# *Yersinia* spp. Identification Using Copy Diversity in the Chromosomal 16S rRNA Gene Sequence

**DOI:** 10.1371/journal.pone.0147639

**Published:** 2016-01-25

**Authors:** Huijing Hao, Junrong Liang, Ran Duan, Yuhuang Chen, Chang Liu, Yuchun Xiao, Xu Li, Mingming Su, Huaiqi Jing, Xin Wang

**Affiliations:** 1 National Institute for Communicable Disease Control and Prevention, Chinese Center for Disease Control and Prevention, State Key Laboratory of Infectious Disease Prevention and Control, Collaborative Innovation Center for Diagnosis and Treatment of Infectious Diseases, Beijing, China; 2 Department of Pathogenic Biology, School of Medical Science, Jiangsu University, Zhenjiang, Jiangsu Province, China; 3 Institute of Biophysics, Chinese Academy of Sciences, Beijing, China; Beijing Institute of Microbiology and Epidemiology, CHINA

## Abstract

API 20E strip test, the standard for Enterobacteriaceae identification, is not sufficient to discriminate some *Yersinia* species for some unstable biochemical reactions and the same biochemical profile presented in some species, e.g. *Yersinia ferderiksenii* and *Yersinia intermedia*, which need a variety of molecular biology methods as auxiliaries for identification. The 16S rRNA gene is considered a valuable tool for assigning bacterial strains to species. However, the resolution of the 16S rRNA gene may be insufficient for discrimination because of the high similarity of sequences between some species and heterogeneity within copies at the intra-genomic level. In this study, for each strain we randomly selected five 16S rRNA gene clones from 768 *Yersinia* strains, and collected 3,840 sequences of the 16S rRNA gene from 10 species, which were divided into 439 patterns. The similarity among the five clones of 16S rRNA gene is over 99% for most strains. Identical sequences were found in strains of different species. A phylogenetic tree was constructed using the five 16S rRNA gene sequences for each strain where the phylogenetic classifications are consistent with biochemical tests; and species that are difficult to identify by biochemical phenotype can be differentiated. Most *Yersinia* strains form distinct groups within each species. However *Yersinia kristensenii*, a heterogeneous species, clusters with some *Yersinia enterocolitica* and *Yersinia ferderiksenii*/*intermedia* strains, while not affecting the overall efficiency of this species classification. In conclusion, through analysis derived from integrated information from multiple 16S rRNA gene sequences, the discrimination ability of *Yersinia* species is improved using our method.

## Introduction

The genus *Yersinia* is widely distributed in nature and currently has 17 species [1–3], three of which are pathogenic (*Y*. *enterocolitica*, *Y*. *pestis*, and *Y*. *pseudotuberculosis*) and have been exhaustively researched. The remaining species are referred to as *Y*. *enterocolitica*-like bacteria where their etiology in disease is not understood [4, 5]. Traditionally, bacteria were classified according to similarities and differences in phenotypes, such as morphology and biochemical reactions. The API 20E strip test is the standard for identifying Enterobacteriaceae; however this test has limitations in identifying *Yersinia* species. Several *Yersinia* species can be identified using API 20E strip test where the accuracy is influenced by passage number, culture conditions, and instability of some biochemical reactions [6]. Therefore, sensitive molecular biology methods are needed to assist the traditional approach to identify the *Yersinia*.

Since the 1980s, the ribosomal RNA gene has been used for phylogenetic studies, and ribosomal RNA-based approaches have been increasingly applied to bacterial classification and identification, especially using the 16S rRNA. The 16S rRNA gene is generally accepted as the best molecular sequence to use for identification because it is functionally constant and shows a mosaic of structure having conserved and variable regions and exists in all organisms; and its length is easily sequenced [7]. These properties make it uniquely well suited for systematics applications. However, the presence of multiple copies of the rRNA operons and intra-genomic heterogeneity in the 16S rRNA gene is regarded as a limiting factor in species identification [8–10]. In bacteria, three rRNA genes (16S, 23S, and 5S) are organized into a gene cluster, which is expressed as a single operon. The total number of rRNA operons in prokaryotes range from one to 15[10].With more and more bacterial genomes being completely sequenced, the heterogeneity of 16S rRNA gene at the intra-genomic level has been discussed. Pei collected 883 prokaryotic genomes from the GenBank database, representing 568 unique species, 235 of which have copy diversity in the 16S rRNA gene within a genome (0.06% to 20.38%)[9]. The diversity in 24 species are close to or over the threshold of the 16S rRNA gene-based operational definition of a species (1% to 1.3% diversity)[9], so these species maybe misclassified into a new species if a different copy is used for identification. Recently, 2,013 genomes were analyzed and 22.5% were found divergence at over 1% in the 16S rRNA gene copies [11]. In addition, the 16S rRNA gene shows high similarity comparing some different species where identical sequences were found even[12]. This limits the use of the 16S rRNA gene in identifying bacterial species. Therefore, sequencing the 16S rRNA gene from multiple operons from isolates is recommended to achieve significant phylogenetic information for species identification [13].

Given the reasons above, we used the copy diversity in the 16S rRNA gene to identify *Yersinia* species by analyzing multiple copies of the 16S rRNA gene from 768 strains within the genus *Yersinia*.

## Materials and Methods

### Source of Strains

We used 768 strains of *Yersinia* including 10 species of *Yersinia*. Seven hundred and eleven-one isolated strains were widely distributed within 21 provinces of China; and thirty-three reference strains were from Japan, France and the National Institutes for Food and Drug Control (NIFDC) in China. The dates of strain isolation encompass 52 years (1962–2014). Biochemical data were determined using API 20E test strips (Biomerieux, France), showing 407 strains of *Y*. *ferderiksenii/intermedia*, 119 strains of *Y*. *kristensenii*, 123 strains of *Y*. *enterocolitica* (72 nonpathogenic and 51 pathogenic), 46 strains of *Y*. *pseudotuberculosis* and 49 strains of *Y*. *pestis*. The source and hosts of strains are shown in Table 1. Twenty-four complete-genome-sequenced strains were selected from the NCBI (http://www.ncbi.nlm.nih.gov/pubmed/) with the GenBank number listed in Table 2.

**Table 1:** Source and host distribution of *Yersinia* strains used.

Source and host		*Y*. *ferderiksenii/intermedia*	*Y*. *kristensenii*	*Y*. *enterocolitica*		*Y*. *pseudotuberculosis*	*Y*. *pestis*
				Non-pathogenic	pathogenic		
Strains isolated in China	Chicken	21	17	6			
	Cattle	6		1			
	Dogs	16	6	3			
	Rats	166	31	43	8	28	2
	Ducks	2	4				
	Goat	4	4	2			
	Mandarin duck	5	7				
	Swines	148	30	10	19	3	
	Marmots						31
	Flies	2		1	1		
	Ticks						2
	Fleas						6
	Diarrhea patients	12	1	4	16		4
	Food	8	9	2			
	Others^e^	9	4		3		4
	Total	407	119	72	51	46	49
Reference strains		8^a^	6^b^		4^c^	15^d^	

^a^All reference strains cited here are from NIFDC, two are *Y*. *ferderiksenii* strains (52235 and 52236) and six are *Y*. *intermedia* strains (52234, 52237, 52244,52248, 52249, and 52250).

^b^Among the reference strains, four are from NIFDC (52232, 52242, 52246, and 52247), and two from Japan.

^c^All reference strains are from Japan.

^d^Among these, six are from NIFDC (53504, 53505, 53510, 53512, 53514, and 53518), eight (PTB3, YP1B, YB2B, YP011, YP014, YP2A, YP15, and YP6) from Japan, and one (YP010) from France.

^e^ All bacteria strains were collected from animals, not human subjects.

**Table 2:** The GenBank numbers of 24 complete-genome-sequenced strains.

Strains	Genbank number	Strains	Genbank number
*Y*.*aldovae* 670–83	CP009781.1	*Y*. *pestis* Pestoides F	CP000668.1
*Y*. *aleksiciae* strain 159	CP011975.1	*Y*. *pseudotuberculosis* IP31758	CP000720.1
*Y*. *ferderiksenii* ATCC33641	KN150731.1	*Y*. *pseudotuberculosis* PB1/+	CP001048.1
*Y*. *ferderiksenii* Y225	CP009364.1	*Y*. *pseudotuberculosis* YPIII	CP000950.1
*Y*. *intermedia* strain Y228	CP009801.1	*Y*. *rohdei* strain YRA	CP009787.1
*Y*. *kristensenii* ATCC33639	CP008955.1	*Y*. *ruckeri* strain YRB	CP009539.1
*Y*. *kristensenii* Y231	CP009997.1	*Y*. *similis* strain 228	CP007230.1
*Y*. *pestis* A1122	CP002956.1	*Y*. *enterocolitica* strain 2516–87	CP009838.1
*Y*. *pestis* D106004	CP001585.1	*Y*. *enterocolitica* strain WA	CP009367.1
*Y*. *pestis* D182038	CP001589.1	*Y*. *enterocolitica subsp*. *enterocolitica* 8081	AM286415.1
*Y*. *pestis* KIM10+	AE009952.1	*Y*. *enterocolitica subsp*. *palearctica* Y11	FR729477.2
*Y*. *pestis* Nepal516	CP000305.1	*Y*. *enterocolitica subsp*. *palearctica* 105.5R(r)	CP002246.1

### Culture and Identification of Strains

Enrichment was performed using phosphate-buffered saline with sorbitol and bile salts (PSB) at 4°C for 21 days. Then strains were inoculated onto *Yersinia*-selective agar (cefsulodin-irgasan-novobiocin [CIN] agar; Difco). Suspected colonies having a typical bull’s-eye appearance (deep red centers surrounded by an outer transparent zone) on CIN agar were selected and allowed to grow on brain heart infusion agar (Beijing Land Bridge Technology Co., Ltd., China) at 25°C for 24 to 48 hours to obtain pure strains[14, 15]. We used the commercial rapid identification system, API 20E strip test (bioMérieux, France), for the identification of presumptive isolates. The biotypes of *Y*. *enterocolitica* strains were identified using the scheme reviewed by Bottone [16].

### T-vectors Cloning and Sequencing of the 16S rRNA Gene

The bacterial genome was extracted using a DNA nucleic acid extraction kit (Tiangen, China). The 16S rRNA gene was amplified using the universal primers 27F (5’-AGAGTTTGATCCTGGCTCAG-3’) and 1492R (5’-GGTTACCTTGTTACGACTT-3’)[17]with the Q5 Hot Start High-Fidelity 2×Master Mix(NEB, U.K.). After gel electrophoresis, specific PCR products were purified using a pEASY purification kit (Transgene, China). Connection and transformation were performed using the pEASY^TM^-Blunt Simple Cloning Kit (Transgene, China). The transformed bacteria were grown at 37°C for 12h on Luria-Bertani agar, containing X-gal (20 mg/ml), IPTG (24 mg/ml), and kanamycin (50 mg/ml). Five white single colonies were randomly selected for sequencing. All were sequenced with an ABI Prism BigDye Terminator cycle sequencing ready reaction kit using AmpliTaq DNA polymerase and an ABI Prism 377xl DNA sequencer (Applied Biosystems, Foster City, CA, USA) according to the instructions of the manufacturer at Tsingke BioTech Co., Ltd., sequencing the amplicons in both directions.

### Sequence Analysis

Sequence alignment was performed using Seqman (Lersergene7.0). A phylogenetic tree based on a single copy of the 16S rRNA gene was constructed using Kimura’s 2-parameter distance and the neighbor-joining method (MEGA 6.0). All of the 16S rRNA gene copies from each complete-genome-sequenced strain were coded with a random number, and then five sequences were selected using a random number table. After elimination of redundancies, all the five sequences in the 16S rRNA gene of each strain were obtained corresponding to a unique pattern number. The minimum distance between species was calculated on the basis of a full array between every two strains and each of the five sequences. A phylogenetic tree based on the multi-copy of the diverse 16S rRNA gene was constructed using a distance matrix. The distance of the five sequences within each strain was also calculated.

### Ethics

The sample collection and detection protocols were approved by the Ethics Review Committee [Institutional Review Board (IRB)] of National Institute for Communicable Disease Control and Prevention, Chinese Center for Disease Control and Prevention. The copy of ethical approval documents were provided comply with PLOS ONE requirements. The authors were involved in sample collections involving human subjects and authors can access to personal information before the authors had access to the data. Signed informed consent was obtained from all study participants. For all the patients under 18 years-old, a written consent form was signed by a parent or legal guardian. The field studies did not involve endangered or protected species, so the locations/activities for which specific permission was not required.

## Results

### 16S rRNA Copies at the Intra-Genomic Level

**Fig 1:**
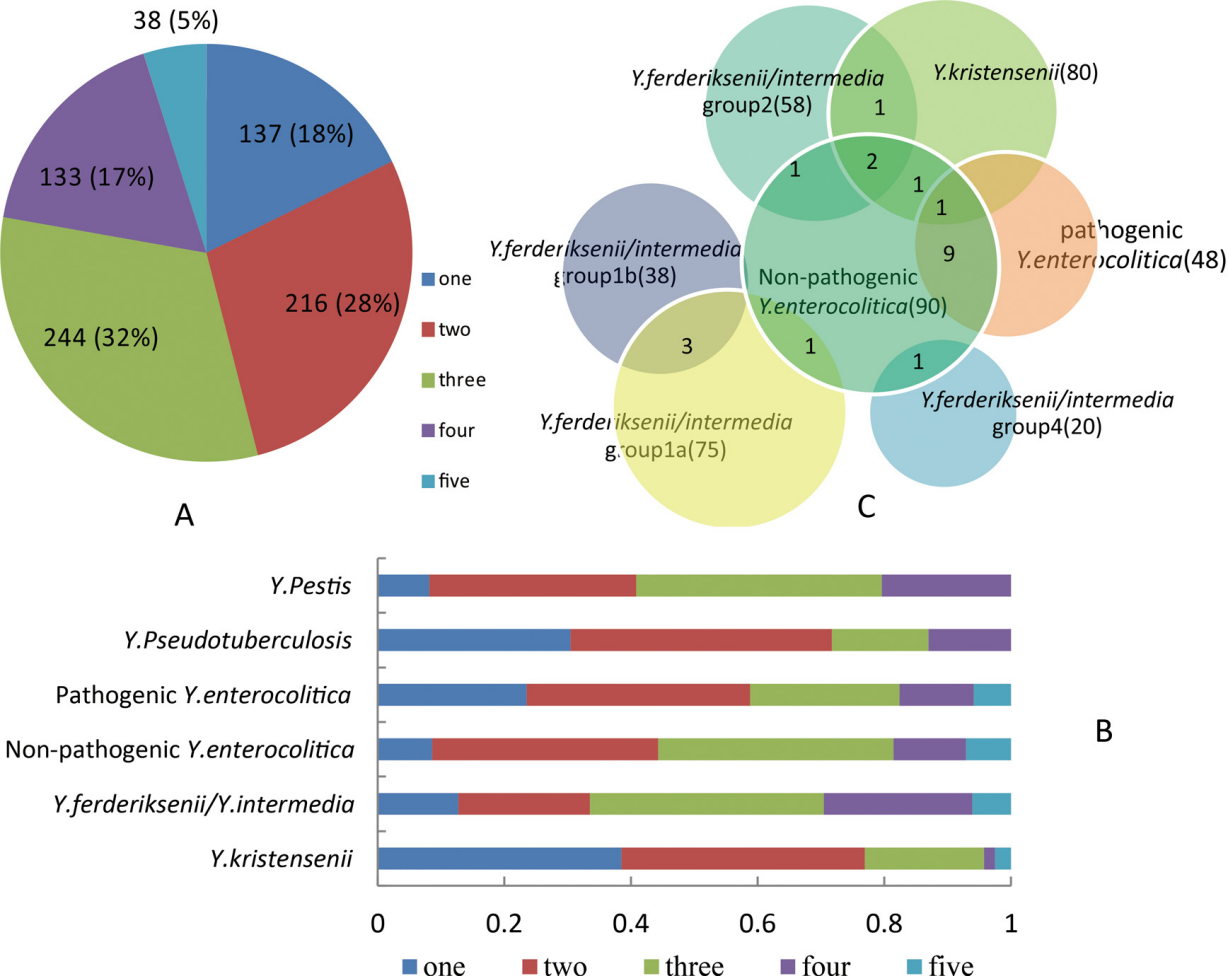
A. Distribution of the type number of 16S rRNA genes in 768 *Yersinia* strains. The colours in different sections of the pie chart represent the type number of 16S rRNA gene in strains, one type, two types, three types, four types, five types, respectively. The number in the pie represents the number of strains that have each kind of copies of 16S rRNA gene, the percentage in parentheses represents the proportion of all strains. B. The proportion of each copy appearing in different *Yersinia* species. C. The identical 16S rRNA patterns that exist in different *Yersinia* species, except for *Y*. *pestis and Y*. *pseudotuberculosis*. Numbers in the crossed circle represent the number of identical patterns in the corresponding *Yersinia* species. Numbers in parentheses represent the amount of total patterns in corresponding species. Specific patterns are not shown.

In total, 3,840 sequences of 16S rRNA gene were obtained from 768 strains used in this study. One or more base difference between two sequences was defined as a different 16S rRNA gene type. The type number of intra-strain 16S rRNA genes is shown in Fig 1A. In general, 60% of the strains have two or three types, 17% have four, and 18% have one. Only 5% of the strains have five totally different 16S rRNA gene types. Fig 1B shows a comparison of the gene type number between the different *Yersinia* species. The number is primarily one or two in *Y*. *pseudotuberculosis* and *Y*. *kristensenii*, and two to four in *Y*. *ferderiksenii/intermedia*. The strains of pathogenic *Y*. *enterocolitica* usually have one to three types, whereas the non-pathogenic strains have two to three types where one strain having five identical copies is uncommon at about 8% of the strains. *Y*. *pestis* and *Y*. *pseudotuberculosis* do not have five different copy sequences.

### Pairwise Comparison of the 16S rRNA Gene at the Intra-Genomic Level

The sequence similarity comparison between pairwise copies of 16S rRNA genes in each strain is shown in Table 3 classified by *Yersinia* species. The sequence similarity of pairwise copies in each *Yersinia* species strain exceeds 99% for most strains. There are only nine *Y*. *ferderiksenii/intermedia* (accounting for 2.2%) of the strains with similarity below 98.7%, and one strain of *Y*. *kristensenii* and non-pathogenic *Y*. *enterocolitica* of relatively low similarity 96.77% and 97.94%, respectively, whose sequence variation are 47 and 30 bases, respectively.

**Table 3:** Pairwise comparison of 16S rRNA gene at the intra-genomic level in each *Yersinia* species.

Species	Similarity*			Minimum value (%)
	100%	100–99%	<98.7%	
*Y*. *ferderiksenii/intermedia*	52(12.7%)	349(85.1%)	9(2.2%)	98.15
*Y*. *kristensenii*	45(37.2%)	75(62.0%)	1(0.8%)	96.77
Pathogenic *Y*. *enterocolitica*	12(22.6%)	40(75.5%)	1(1.9%)	98.22
Non-pathogenic *Y*. *enterocolitica*	6(8.0%)	68(90.7%)	1(1.3%)	97.94
*Y*. *pseudotuberculosis*	15(30.6%)	34(69.4%)		99.45
*Y*. *pestis*	5(9.1%)	50(90.9%)		99.79

^*^ There is not one strain in our study with similarity between 99%-98.7% in different copies of 16S rRNA gene, so the group of <99%, ≥98.7% is not shown in Table 3. The group of 100–99% means the similarity <100% and ≥99%.

### Cluster Analysis Based on 16S rRNA Gene Sequence

**Fig 2:**
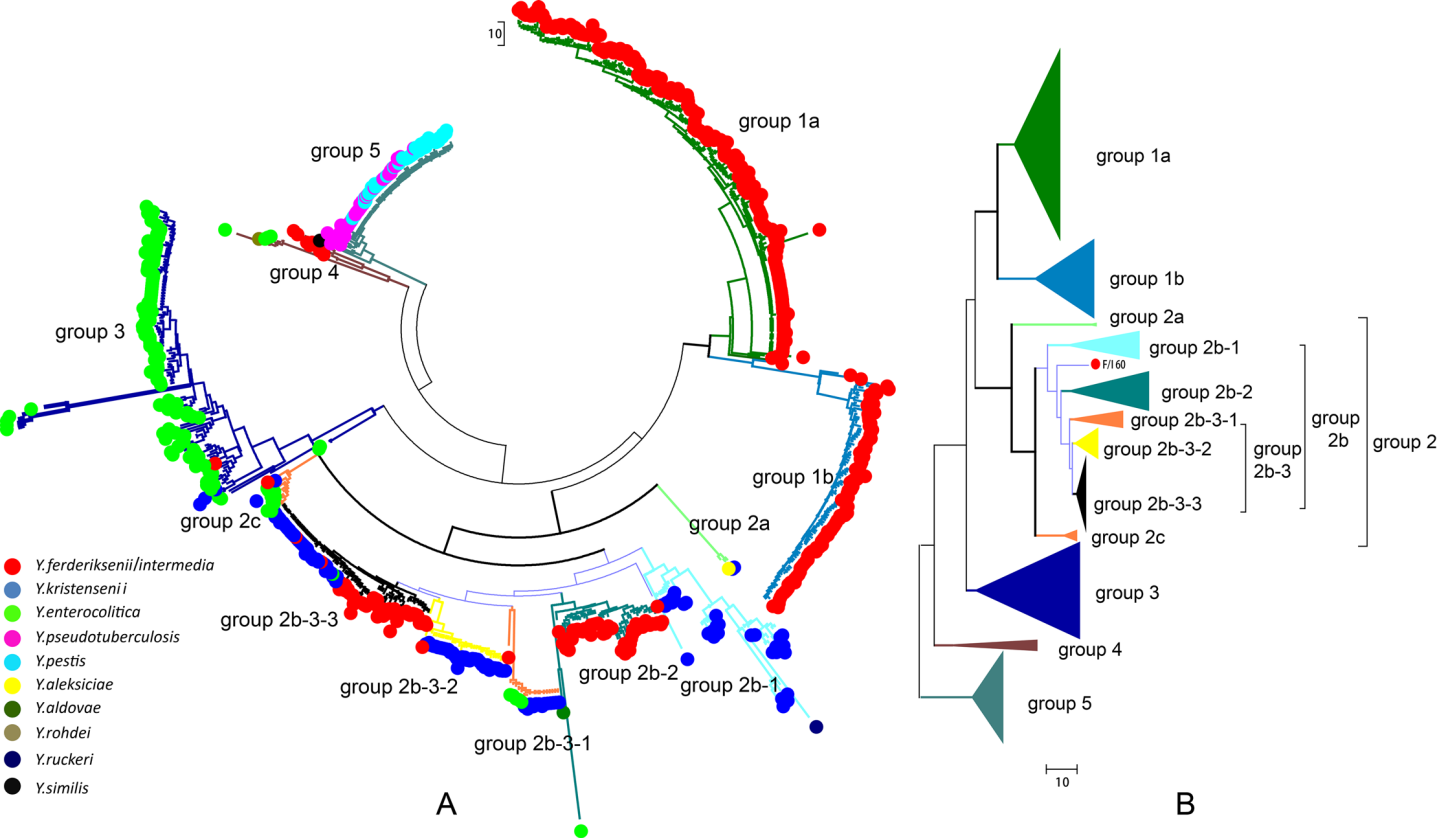
A phylogenetic tree constructed on the basis of the five copies of 16S rRNA gene in each strain using the minimum evolution method. **A.** Dots with different colors represent the corresponding *Yersinia* species; tree branch colors are consistent with triangles in **B.**, which represent different clustering groups.

A phylogenetic tree (Fig 2), constructed on the basis of the five copies of 16S rRNA gene presented in each strain and using the minimum evolution method, can be divided into six groups: 1a, 1b, 2, 3, 4 and 5.

Group 1a and group 1b comprise *Y*. *ferderiksenii/intermedia* strains, 220 and 90 strains, respectively. The similarity between strains of each group is over 90%. Group 3 primarily contains *Y*. *enterocolitica* strains; but clustered with three *Y*. *kristenii* strains and one *Y*. *ferderiksenii* strain. *Y*. *enterocolitica* strains of biotype 1b show a distinct sub-cluster (the dark blue and bold clade in Fig 1A) in group 3, three isolated strains and two complete-genome-sequenced strains WA and 8081. Two sub-clusters comprise group 4: one is a combination of *Y*. *ferderiksenii/intermedia* strains, represented by two reference strains of *Y*. *ferderksenii*, ATCC33641 and 52235; another one contains four *Y*. *enterocolitica* strains and one complete-genome-sequenced strain of *Y*. *rohdei*. Group 5 consists of all the strains of *Y*. *pestis* and *Y*. *pseudotuberculosis* involved in this study, and a complete-genome-sequenced strain of *Y*. *similis*, located outside of the group.

Group 2 contains multiple *Yersinia* species, such as *Y*. *enterocolitica*, *Y*. *ferderiksenii/intermedia* and *Y*. *kristensenii*, which can be divided into three clusters: a, b and c. Group 2a includes two reference strains of *Y*. *kristensenii* and one complete-genome-sequenced strain of *Y*. *aleksiciae*. Group 2b is complicated hence described in three parts. Group 2b-1 is primarily made up of *Y*. *kristenii* strains, next is two reference strains of *Y*. *kristensenii*, 52246 and 52232, and one complete-genome-sequenced strain of *Y*. *ruckeri*. Group 2b-2 has two sub-clusters, one consisting of an *Y*. *enterocolitica* strain and a whole-genome-sequenced strain of *Y*. *aldovae*, and another with 43 *Y*. *ferderiksenii/intermedia* strains of high similarity (>99.6%), two of which are reference strains of *Y*. *intermedia*. Group 2b-3 has three sub-clusters. The first one contains 13 *Y*. *kristensenii* strains, five *Y*. *enterocolitica* strains, and one complete-genome-sequenced strain of *Y*. *ferderiksenii* Y225 that is distant from the rest of the strains in this sub-cluster. The second contains 33 *Y*. *kristensenii* strains of high similarity (>99.6%), except for two *Y*. *ferderiksenii/kristensenii* strains. The third with high similarities between strains (>99.6%), presents diversity, containing 35 *Y*. *kristensenii* (two are complete-genome-sequenced) and 43 *Y*. *ferderiksenii/intermedia* strains (three reference strains of *Y*. *intermedia* and one complete-genome-sequenced strain of *Y*. *intermedia*). There is a single *Y*. *ferderiksenii/intermedia* strain alone between group 2–1 and group 2b-2. Group 2c contains nine *Y*. *enterocolitica* strains, two *Y*. *kristensenii* strains and one complete-genome-sequenced strain of *Y*. *ferderiksenii*.

### The Dominant Patterns of the 16S rRNA Gene in Each *Yersinia* Species

Four hundred thirty-nine patterns of 16S rRNA gene sequences, coded as 0 to 438(S1 File and S1 Table), were obtained after removing the redundant sequences. Table 4 shows each *Yersinia* species has dominant patterns. Though non-dominant patterns are more than dominant patterns, they appear at a low frequency and usually appear only once. There exists the same dominant gene pattern in the strains of *Y*. *kristensenii* and *Y*. *ferderiksenii/intermedia*, and *Y*. *pestis* and *Y*. *pseudotuberculosis*, respectively.

**Table 4:** The dominant patterns of the 16S rRNA genes in each *Yersinia* species.

Species	No. strains	No. sequences of 16S rRNA gene	No. patterns of 16S rRNA gene	Dominant 16S rRNA gene pattern and its percentage
Pathogenic- *Y*. *enterocolitica*	53	265	48	32(51.7%)
Nonpathogenic- *Y*. *enterocolitica*	75	375	90	19(20.8%) 120(10.4%)
*Y*. *ferderiksenii/intermedia* group 1a	220	110	75	11(41.5%) 13(17.5%) 23(10.1%)
*Y*. *ferderiksenii/intermedia* group 1b	91	455	38	142(31.2%) 3(18.7%) 55(12.1%)
*Y*. *ferderiksenii/intermedia* group 2b	88	440	58	10(20.0%) 111(13.2%) 8(13.0%) 9(11.4%)
*Y*. *ferderiksenii/intermedia* group 4	7	35	20	72(14.3%) 98(11.4%)
*Y*. *kristensenii*	121	605	80	10(31.9%) 95(14.7%)
*Y*. *pestis*	55	275	45	1(34.2%)
*Y*.*pseudotuberculosis*	49	245	28	1(64.1%)

### Identical 16S rRNA Gene Sequences from Different *Yersinia* Species

Strains of different *Yersinia* species have identical 16S rRNA gene sequences (Fig 1C). Non-pathogenic *Y*. *enterocolitica* strains (biotype 1A) have the same 16S rRNA gene patterns with many kinds of other *Yersinia* species, with nine same gene patterns with pathogenic *Y*. *enterocolitica*, four with *Y*. *kristensenii* strains and only one with the other *Yersinia* species. *Y*. *kristensenii* strains have three identical gene patterns with *Y*. *ferderiksenii/intermedia* strains from group 2b, one of which is dominant, accounting 20% for *Y*. *kristensenii* and 31.9% for *Y*. *ferderiksenii/intermedia* respectively. There also exist identical 16S rRNA gene patterns among non-pathogenic *Y*. *enterocolitica*, *Y*. *kristensenii*, and *Y*. *ferderiksenii/intermedia* strains from group 2b, and also among non-pathogenic *Y*. *enterocolitica*, *Y*. *kristensenii*, and pathogenic *Y*. *enterocolitica*. Only group 1a and 1b exist three same 16S rRNA gene patterns among the four groups of *Y*. *ferderiksenii/intermedia* (1a, 1b, 2 and 4). *Y*. *pestis* strains have six identical patterns with *Y*. *pseudotuberculosis* (not shown in Fig 1C), one of which is dominant in both species, accounting for 34.2% and 64.1%, respectively.

## Discussion

Compared to bacteria identification methods using phenotype, the approach based on genotype stands out for its consistency. One desirable candidate is the 16S rRNA gene, highly conserved and seldom variable within species, and is becoming an important technique for phylogeny research and species classification[7]. Specifically speaking, for *Yersinia*, the limitation of commercial tests for identification systems such as API 20E strip test has shown a deficiency in distinguishing some species[18, 19], which can be made up through the method based on 16S rRNA gene. However, the similarity of 16S rRNA gene sequence between species is as high as 96.9%-99.8%; it is easy therefore to misclassify species of high homology[20]. Recently, the rRNA operon is shown to have multiple copies with some heterogeneity[17]. In some cases, the diversity between multiple copies of a strain is so high as to be misclassified into different species if a different copy is used to identify a bacterial strain [21, 22]. This is a limiting factor for species identification using a single 16S rRNA gene. The discrimination ability can be improved by integrating multiple 16S rRNA gene copies. Therefore, here we first report species differentiation using five copies of the 16S rRNA gene from 768 strains of *Yersinia*.

Previously, the copy number of *Yersinia*16S rRNA gene was shown to be around seven [11]. According to whole genome sequence strain from NCBI, the number of variable copies is within three. Accordingly, five colonies was randomly selected for 16S rRNA gene sequencing for each strain. Our results show 60% of the strains have two to three genotypes and only 5% have all 5 types, therefore the selection of 5 colonies is reasonable. Though 84% of the strains have more than one sequence type of 16S rRNA gene, the comparison of the intra-genomic 16S rRNA gene showed that the similarity of 98.4% of the strains is above the threshold of species identification (98.7% to 99%)[9]. In other words, for most *Yersinia* strains, the heterogeneity among different copies does not affect species classification. Whereas for 12 strains, the similarity of intra-genomic 16S rRNA gene is below or close to the threshold of classification. When using a single copy for identification, a different result may occur. Hence in this study, with integrated information from multiple 16S rRNA genes and a comprehensive reflection of phylogeny, species identification is more accurate.

A phylogenetic tree (Fig 2) was constructed by combining five 16S rRNA gene sequences for each strain, most clustering of species is accordant with classical identification. *Y*. *pestis* and *Y*. *pseudotuberculosis* share a large number of identical 16S rRNA gene sequences and are close to each other in the phylogenetic tree, which is consistent with the hypothesis that *Y*. *pestis* evolved from O: 1b strains of *Y*. *pseudotuberculosis* about 1,500–20,000 years ago[23, 24], indirectly supporting our method. *Y*. *enterocolitica* strains are in group 3, where *Y*. *enterocolitica* subsp. *enterocolitica* (biotype 1b) and *Y*. *enterocolitica* subsp. *Palearctica* (biotype 1A, 2, 3, 4 and 5) diverge into two clusters, coinciding with previous work[25–27].

The majority of *Y*. *kristensenii* strains are within group 2, spreading widely and having multiple branches. In group2b-3-3, *Y*. *kristensenii* and *Y*. *ferderiksenii/intermedia* strains are closely clustered with each other, sharing high similarity, due to the fact that the consensus sequence of the two species are also dominant in each species. Notably, very few *Y*. *kristensenii* strains cluster with *Y*. *enterocolitica* strains. Compared with other species, *Y*. *kristensenii* presents higher genetic variability. This may be owing to the uncertainty of biochemical identification that these ‘cross-over’ strains may be classified into other *Yersinia* species. Sequencing more than five random selected colonies may solve this problem and needs further investigation.

**Fig 3:**
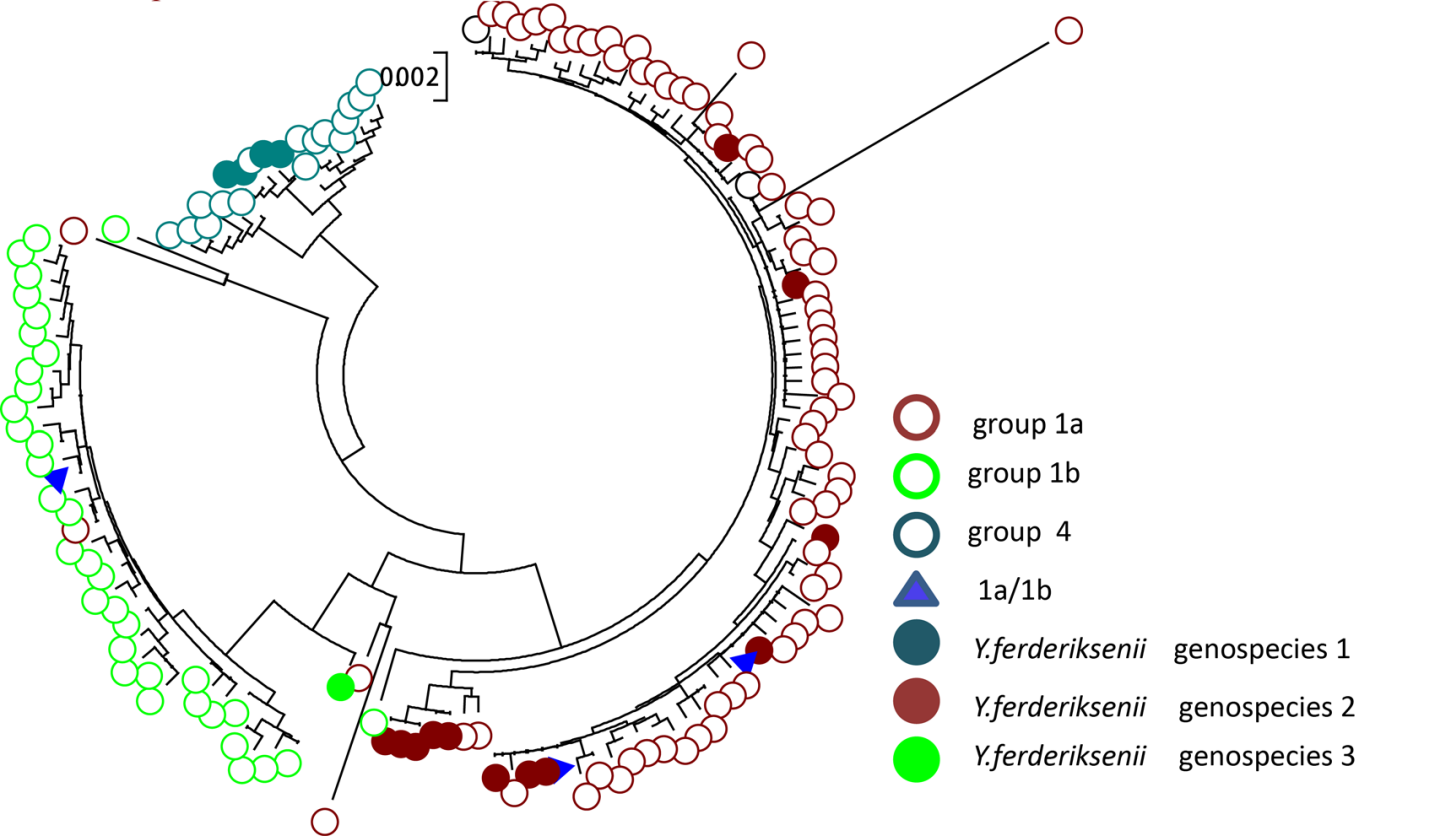
Phylogenetic tree based on single 16S rRNA gene from *Y*. *ferderiksenii*/*intermedia* strains in group 1a, 1b, and 4 and strains of *Y*. *ferderiksenii* belonging to three geno-species. Hollow circles represent all 16S rRNA gene types of *Y*. *ferderiksenii* /*intermedia* strains in group 1a, 1b, and 4; while solid circles represent *Y*. *ferderiksenii*s trains of three geno-species [1]. Triangles represent identical 16S rRNA gene patterns of strains in group 1a and group 1b.

*Y*. *ferderiksenii* and *Y*. *intermedia* strains are indistinguishable using API 20E strip test where they are referred to as *Y*. *ferderiksenii/intermedia*. In this study, they are gathered within group 1a, 1b, 2b and 4. The reference strains of *Y*. *intermedia* were all in group 2b, so strains in this group should be classified to be *Y*. *intermedia* strains. Consequently, strains in group 1a, 1b and 4 belong to *Y*. *ferderiksenii*. Identified in 1980, *Y*. *ferderiksenii* included three geno-species without biochemical differences [28]. Clustering analysis showed sequence types of group 1a, 1b, and 4 clustered with geno-species 2, 3, 1 of *Y*. *ferderiksenii* strains, respectively (Fig 3). Therefore, the 16S rRNA gene sequence types can be used for unclassified strains from API 20E strip test, and also for subtyping strains.

Presently there is divergence in species classification for *Yersinia*. In addition to traditional biochemical reactions, refined molecular biology techniques are needed to compensate for this deficiency. *Y*. *aleksiciae* and *Y*. *kristensenii* have no difference in their biochemical phenotype but differ into distinct species using 16S rRNA gene sequences[29]. In group 2a, *Y*. *kristensenii* reference strains (52242 and 52247) share 99.9% similarity with the genome sequenced strain of *Y*. *aleksiciae*, a recent phylogenetic relationship. A previous study shows *Y*.*similis* is indistinguishable from *Y*. *pseudotuberculosis* through biochemical phenotyping that it was regarded as *Y*. *pseudotuberculosis*; however, their pathogenicity are significantly different and they differed using 16S rRNA gene sequencing [30]. In this study, genome sequenced strain of *Y*. *similis* were inseparably clustered with *Y*. *pseudotuberculosis* and *Y*. *pestis* in group 5, an outlier (black dot in group 5 Fig 2A) with an outermost evolutionary distance. Further, several comparable subspecies clustered respectively in group2b-1 and group 2b-2: genome sequenced *Y*. *ruckeri* strain and four *Y*. *kristensenii* strains formed a sub-branch; *Y*. *aldovae* and one *Y*. *enterocolitica* strain formed another, far away from the rest of the *Y*. *enterocolitica* strains. We suspect this *Y*. *enterocolitica* strain may belong to another undescribed *Yersinia* species. Similarly, in group 4, three *Y*. *enterocolitica* strains formed another sub-branch with one genome sequenced strain *of Y*. *rohdei*. All of these strains show *Yersinia* is a genetically diverse genus; the strains involved here may be classified into other species of more diversity.

Based on integrated information from multiple 16S rRNA gene copies and clustering analysis, this study shows comparable species clusters of *Yersinia* and reduces identification chaos derived from using a single copy of the 16S rRNA gene sequence which is sometimes similar or identical between species, and remarkably increases the discrimination ability of 16S rRNA gene sequence. Further study using our classification method would allow increased differentiation of *Yersinia* species identification.

## Supporting Information

S1 File The 439 Patterns (Coded as 0 to 438) of 16S rRNA Gene Sequences in this study. (PDF)

S1 Table Patterns Code of Five 16S rRNA Gene Sequences for each Strain. (XLSX)
