# Supplementary material for: Yersinia spp. Identification Using Copy Diversity in the Chromosomal 16S rRNA Gene Sequence
Source: PLoS One. 2016 Jan 25;11(1):e0147639. doi: 10.1371/journal.pone.0147639 (PMC4726496; doi:10.1371/journal.pone.0147639)
Supplement: S1 File — (PDF) [file pone.0147639.s001.pdf]

439 patterns of 16S rRNA gene sequences

pattern 0

CGCTGGCGGCAGGCCTAACACATGCAAGTCGAGCGGCAGCGGGAAGTAGTTTACTACT
TTGCCGGCGAGCGGCGGACGGGTGAGTAATGTCTGGGGATCTGCCTGATGGAGGGGGA
TAACTACTGGAAACGGTAGCTAATACCGCATGACCTCGCAAGAGCAAAGTGGGGGACC
TTAGGGCCTCACGCCATCGGATGAACCCAGATGGGATTAGCTAGTAGGTGGGGTAATGG
CTCACCTAGGCGACGATCCCTAGCTGGTCTGAGAGGATGACCAGCCACACTGGAACTG
AGACACGGTCCAGACTCCTACGGGAGGCAGCAGTGGGGAATATTGCACAATGGGCGCA
AGCCTGATGCAGCCATGCCGCGTGTGTGAAGAAGGCCTTCGGGTTGTAAAGCACTTTCA
GCGAGGAGGAAGGGGTTGAGTTTAATACGCTCAATCATTGACGTTACTCGCAGAAGAA
GCACCGGCTAACTCCGTGCCAGCAGCCGCGGTAATACGGAGGGTGCAAGCGTTAATCG
GAATTACTGGGCGTAAAGCGCACGCAGGCGGTTTGTTAAGTCAGATGTGAAATCCCCGC
GCTTAACGTGGGAACTGCATTTGAAACTGGCAAGCTAGAGTCTTGTAGAGGGGGGTAG
AATTCCAGGTGTAGCGGTGAAATGCGTAGAGATCTGGAGGAATACCGGTGGCGAAGGC
GGCCCCCTGGACAAAGACTGACGCTCAGGTGCGAAAGCGTGGGGAGCAAACAGGATT
AGATACCCTGGTAGTCCACGCTGTAAACGATGTCGACTTGGAGGTTGTGCCCTTGAGGC
GTGGCTTCCGGAGCTAACGCGTTAAGTCGACCGCCTGGGGAGTACGGCCGCAAGGTTA
AAACTCAAATGAATTGACGGGGGCCCGCACAAGCGGTGGAGCATGTGGTTTAATTCGAT
GCAACGCGAAGAACCTTACCTACTCTTGACATCCACAGAATTTGGCAGAGATGCTAAAG
TGCCTTCGGGAACTGTGAGACAGGTGCTGCATGGCTGTCGTCAGCTCGTGTTGTGAAAT
GTTGGGTTAAGTCCCGCAACGAGCGCAACCCTTATCCTTTGTTGCCAGCACGTAATGGT
GGGAACTCAAGGGAGACTGCCGGTGACAAACCGGAGGAAGGTGGGGATGACGTCAAG
TCATCATGGCCCTTACGAGTAGGGCTACACACGTGCTACAATGGCAGATACAAAGTGAA
GCGAACTCGCGAGAGTCAGCGGACCACATAAAGTCTGTCGTAGTCCGGATTGGAGTCT
GCAACTCGACTCCATGAAGTCGGAATCGCTAGTAATCGTAGATCAGAATGCTACGGTGA
ATACGTTCCCGGGCCTTGTACACACCGCCCGTCACACCATGGGAGTGGGTTGCAAAAG
AAGTAGGTAGCTTAACCTTCGGGAGGGCGCTTACCACTTTGTGATTCATGACTGGGG

pattern 1

CGCTGGCGGCAGGCCTAACACATGCAAGTCGAGCGGCAGCGGGAAGTAGTTTACTACT
TTGCCGGCGAGCGGCGGACGGGTGAGTAATGTCTGGGGATCTGCCTGATGGAGGGGGA
TAACTACTGGAAACGGTAGCTAATACCGCATGACCTCGCAAGAGCAAAGTGGGGGACC
TTAGGGCCTCACGCCATCGGATGAACCCAGATGGGATTAGCTAGTAGGTGGGGTAATGG
CTCACCTAGGCGACGATCCCTAGCTGGTCTGAGAGGATGACCAGCCACACTGGAACTG
AGACACGGTCCAGACTCCTACGGGAGGCAGCAGTGGGGAATATTGCACAATGGGCGCA
AGCCTGATGCAGCCATGCCGCGTGTGTGAAGAAGGCCTTCGGGTTGTAAAGCACTTTCA
GCGAGGAGGAAGGGGTTGAGTTTAATACGCTCAATCATTGACGTTACTCGCAGAAGAA
GCACCGGCTAACTCCGTGCCAGCAGCCGCGGTAATACGGAGGGTGCAAGCGTTAATCG
GAATTACTGGGCGTAAAGCGCACGCAGGCGGTTTGTTAAGTCAGATGTGAAATCCCCGC
GCTTAACGTGGGAACTGCATTTGAAACTGGCAAGCTAGAGTCTTGTAGAGGGGGGTAG
AATTCCAGGTGTAGCGGTGAAATGCGTAGAGATCTGGAGGAATACCGGTGGCGAAGGC
GGCCCCCTGGACAAAGACTGACGCTCAGGTGCGAAAGCGTGGGGAGCAAACAGGATT
AGATACCCTGGTAGTCCACGCTGTAAACGATGTCGACTTGGAGGTTGTGCCCTTGAGGC
GTGGCTTCCGGAGCTAACGCGTTAAGTCGACCGCCTGGGGAGTACGGCCGCAAGGTTA
AAACTCAAATGAATTGACGGGGGCCCGCACAAGCGGTGGAGCATGTGGTTTAATTCGAT

GCAACGCGAAGAACCTTACCTACTCTTGACATCCACAGAATTTGGCAGAGATGCTAAAG
TGCCTTCGGGAACTGTGAGACAGGTGCTGCATGGCTGTCGTCAGCTCGTGTTGTGAAAT
GTTGGGTTAAGTCCCGCAACGAGCGCAACCCTTATCCTTTGTTGCCAGCACGTAATGGT
GGGAACTCAAGGGAGACTGCCGGTGACAAACCGGAGGAAGGTGGGGATGACGTCAAG
TCATCATGGCCCTTACGAGTAGGGCTACACACGTGCTACAATGGCAGATACAAAGTGAA
GCGAACTCGCGAGAGCCAGCGGACCACATAAAGTCTGTCGTAGTCCGGATTGGAGTCT
GCAACTCGACTCCATGAAGTCGGAATCGCTAGTAATCGTAGATCAGAATGCTACGGTGA
ATACGTTCCCGGGCCTTGTACACACCGCCCGTCACACCATGGGAGTGGGTTGCAAAAG
AAGTAGGTAGCTTAACCTTCGGGAGGGCGCTTACCACTTTGTGATTCATGACTGGGG

pattern 2

CGCTGGCGGCAGGCCTAACACATGCAAGTCGAGCGGCAGCGGGAAGTAGTTTACTACT
TTGCCGGCGAGCGGCGGACGGGTGAGTAATGTCTGGGGATCTGCCTGATGGAGGGGGA
TAACTACTGGAAACGGTGGCTAATACCGCATGACCTCGCAAGAGCAAAGTGGGGGACC
TTAGGGCCTCACGCCATCGGATGAACCCAGATGGGATTAGCTAGTAGGTGGGGTAATGG
CTCACCTAGGCGACGATCCCTAGCTGGTCTGAGAGGATGACCAGCCACACTGGAACTG
AGACACGGTCCAGACTCCTACGGGAGGCAGCAGTGGGGAATATTGCACAATGGGCGCA
AGCCTGATGCAGCCATGCCGCGTGTGTGAAGAAGGCCTTCGGGTTGTAAAGCACTTTCA
GCGAGGAGGAAGGGGTTGAGTTTAATACGCTCAATCATTGACGTTACTCGCAGAAGAA
GCACCGGCTAACTCCGTGCCAGCAGCCGCGGTAATACGGAGGGTGCAAGCGTTAATCG
GAATTACTGGGCGTAAAGCGCACGCAGGCGGTTTGTTAAGTCAGATGTGAAATCCCCGC
GCTTAACGTGGGAACTGCATTTGAAACTGGCAAGCTAGAGTCTTGTAGAGGGGGGTAG
AATTCCAGGTGTAGCGGTGAAATGCGTAGAGATCTGGAGGAATACCGGTGGCGAAGGC
GGCCCCCTGGACAAAGACTGACGCTCAGGTGCGAAAGCGTGGGGAGCAAACAGGATT
AGATACCCTGGTAGTCCACGCTGTAAACGATGTCGACTTGGAGGTTGTGCCCTTGAGGC
GTGGCTTCCGGAGCTAACGCGTTAAGTCGACCGCCTGGGGAGTACGGCCGCAAGGTTA
AAACTCAAATGAATTGACGGGGGCCCGCACAAGCGGTGGAGCATGTGGTTTAATTCGAT
GCAACGCGAAGAACCTTACCTACTCTTGACATCCACAGAATTTGGCAGAGATGCTAAAG
TGCCTTCGGGAACTGTGAGACAGGTGCTGCATGGCTGTCGTCAGCTCGTGTTGTGAAAT
GTTGGGTTAAGTCCCGCAACGAGCGCAACCCTTATCCTTTGTTGCCAGCACGTAATGGT
GGGAACTCAAGGGAGACTGCCGGTGACAAACCGGAGGAAGGTGGGGATGACGTCAAG
TCATCATGGCCCTTACGAGTAGGGCTACACACGTGCTACAATGGCAGATACAAAGTGAA
GCGAACTCGCGAGAGCCAGCGGACCACATAAAGTCTGTCGTAGTCCGGATTGGAGTCT
GCAACTCGACTCCATGAAGTCGGAATCGCTAGTAATCGTAGATCAGAATGCTACGGTGA
ATACGTTCCCGGGCCTTGTACACACCGCCCGTCACACCATGGGAGTGGGTTGCAAAAG
AAGTAGGTAGCTTAACCTTCGGGAGGGCGCTTACCACTTTGTGATTCATGACTGGGG

pattern 3

CGCTGGCGGCAGGCCTAACACATGCAAGTCGAGCGGCAGCGGAAAGTAGCTTGCTACT
TTGCCGGCGAGCGGCGGACGGGTGAGTAATGTCTGGGAAACTGCCTGATGGAGGGGGA
TAACTACTGGAAACGGTAGCTAATACCGCATGACCTCGAAAGAGCAAAGTGGGGGACC
TTCGGGCCTCACGCCATCGGATGTGCCCAGATGGGATTAGCTAGTAGGTGGGGTAATGG
CTCACCTAGGCGACGATCCCTAGCTGGTCTGAGAGGATGACCAGCCACACTGGAACTG
AGACACGGTCCAGACTCCTACGGGAGGCAGCAGTGGGGAATATTGCACAATGGGCGCA
AGCCTGATGCAGCCATGCCGCGTGTGTGAAGAAGGCCTTCGGGTTGTAAAGCACTTTCA
GCGAGGAGGAAGGCATTTCACTTAATACGTGAAGTGATTGACGTTACTCGCAGAAGAA

GCACCGGCTAACTCCGTGCCAGCAGCCGCGGTAATACGGAGGGTGCAAGCGTTAATCG
GAATTACTGGGCGTAAAGCGCACGCAGGCGGTTTGTTAAGTCAGATGTGAAATCCCCGA
GCTTAACTTGGGAACTGCATTTGAAACTGGCAAGCTAGAGTCTTGTAGAGGGGGGTAG
AATTCCAGGTGTAGCGGTGAAATGCGTAGAGATCTGGAGGAATACCGGTGGCGAAGGC
GGCCCCCTGGACAAAGACTGACGCTCAGGTGCGAAAGCGTGGGGAGCAAACAGGATT
AGATACCCTGGTAGTCCACGCTGTAAACGATGTCGACTTGGAGGTTGTGCCCTTGAGGC
GTGGCTTCCGGAGCTAACGCGTTAAGTCGACCGCCTGGGGAGTACGGCCGCAAGGTTA
AAACTCAAATGAATTGACGGGGGCCCGCACAAGCGGTGGAGCATGTGGTTTAATTCGAT
GCAACGCGAAGAACCTTACCTACTCTTGACATCCACAGAACTGAGCAGAGATGCTTAG
GTGCCTTCGGGAACTGTGAGACAGGTGCTGCATGGCTGTCGTCAGCTCGTGTTGTGAAA
TGTTGGGTTAAGTCCCGCAACGAGCGCAACCCTTATCCTTTGTTGCCAGCACGTAATGG
TGGGAACTCAAAGGAGACTGCCGGTGATAAACCGGAGGAAGGTGGGGATGACGTCAA
GTCATCATGGCCCTTACGAGTAGGGCTACACACGTGCTACAATGGCAGATACAAAGTGA
AGCGAACTCGCGAGAGCAAGCGGACCACATAAAGTCTGTCGTAGTCCGGATTGGAGTC
TGCAACTCGACTCCATGAAGTCGGAATCGCTAGTAATCGTAGATCAGAATGCTACGGTG
AATACGTTCCCGGGCCTTGTACACACCGCCCGTCACACCATGGGAGTGGGTTGCAAAA
GAAGTAGGTAGCTTAACCTTCGGGAGGGCGCTTACCACTTTGTGATTCATGACTGGGG
pattern 4
CGCTGGCGGCAGGCCTAACACATGCAAGTCGAGCGGCAGCGGAAAGTAGCTTGCTACT
TTGCCGGCGAGCGGCGGACGGGTGAGTAATGTCTGGGAAACTGCCTGATGGAGGGGGA
TAACTACTGGAAACGGTAGCTAATACCGCATGACCTCGAAAGAGCAAAGTGGGGGACC
TTCGGGCCTCACGCCATCGGATGTGCCCAGATGGGATTAGCTAGTAGGTGAGGTAATGG
CTCACCTAGGCGACGATCCCTAGCTGGTCTGAGAGGATGACCAGCCACACTGGAACTG
AGACACGGTCCAGACTCCTACGGGAGGCAGCAGTGGGGAATATTGCACAATGGGCGCA
AGCCTGATGCAGCCATGCCGCGTGTGTGAAGAAGGCCTTCGGGTTGTAAAGCACTTTCA
GCGAGGAGGAAGGCATTTCACTTAATACGTGAAGTGATTGACGTTACTCGCAGAAGAA
GCACCGGCTAACTCCGTGCCAGCAGCCGCGGTAATACGGAGGGTGCAAGCGTTAATCG
GAATTACTGGGCGTAAAGCGCACGCAGGCGGTTTGTTAAGTCAGATGTGAAATCCCCGA
GCTTAACTTGGGAACTGCATTTGAAACTGGCAAGCTAGAGTCTTGTAGAGGGGGGTAG
AATTCCAGGTGTAGCGGTGAAATGCGTAGAGATCTGGAGGAATACCGGTGGCGAAGGC
GGCCCCCTGGACAAAGACTGACGCTCAGGTGCGAAAGCGTGGGGAGCAAACAGGATT
AGATACCCTGGTAGTCCACGCTGTAAACGATGTCGACTTGGAGGTTGTGCCCTTGAGGC
GTGGCTTCCGGAGCTAACGCGTTAAGTCGACCGCCTGGGGAGTACGGCCGCAAGGTTA
AAACTCAAATGAATTGACGGGGGCCCGCACAAGCGGTGGAGCATGTGGTTTAATTCGAT
GCAACGCGAAGAACCTTACCTACTCTTGACATCCACAGAACTGAGCAGAGATGCTTAG
GTGCCTTCGGGAACTGTGAGACAGGTGCTGCATGGCTGTCGTCAGCTCGTGTTGTGAAA
TGTTGGGTTAAGTCCCGCAACGAGCGCAACCCTTATCCTTTGTTGCCAGCACGTAATGG
TGGGAACTCAAAGGAGACTGCCGGTGATAAACCGGAGGAAGGTGGGGATGACGTCAA
GTCATCATGGCCCTTACGAGTAGGGCTACACACGTGCTACAATGGCAGATACAAAGTGA
AGCGAACTCGCGAGAGCAAGCGGACCACATAAAGTCTGTCGTAGTCCGGATTGGAGTC
TGCAACTCGACTCCATGAAGTCGGAATCGCTAGTAATCGTAGATCAGAATGCTACGGTG
AATACGTTCCCGGGCCTTGTACACACCGCCCGTCACACCATGGGAGTGGGTTGCAAAA
GAAGTAGGTAGCTTAACCTTCGGGAGGGCGCTTACCACTTTGTGATTCATGACTGGGG
pattern 5

CGCTGGCGGCAGGCCTAACACATGCAAGTCGAGCGGCAGCGGAAAGTAGCTTGCTACT
TTGCCGGCGAGCGGCGGACGGGTGAGTAATGTCTGGGAAACTGCCTGATGGAGGGGGA
TAACTACTGGAAACGGTAGCTAATACCGCATGACCTCGAAAGAGCAAAGTGGGGGACC
TTCGGGCCTCACGCCATCGGATGTGCCCAGATGGGATTAGCTAGTAGGTGGGGTAATGG
CTCACCTAGGCGACGATCCCTAGCTGGTCTGAGAGGATGACCAGCCACACTGGAACTG
AGACACGGTCCAGACTCCTACGGGAGGCAGCAGTGGGGAATATTGCACAATGGGCGCA
AGCCTGATGCAGCCATGCCGCGTGTGTGAAGAAGGCCTTCGGGTTGTAAAGCACTTTCA
GCGAGGAGGAAGGCATTTCACTTAATACGTGAAGTGATTGACGTTACTCGCAGAAGAA
GCACCGGCTAACTCCGTGCCAGCAGCCGCGGTAATACGGAGGGTGCAAGCGTTAATCG
GAATTACTGGGCGTAAAGCGCACGCAGGCGGTTTGTTAAGTCAGATGTGAAATCCCCGA
GCTTAACTTGGGAACTGCATTTGAAACTGGCAAGCTAGAGTCTTGTAGAGGGGGGTAG
AATTCCAGGTGTAGCGGTGAAATGCGTAGAGATCTGGAGGAATACCGGTGGCGAAGGC
GGCCCCCTGGACAAAGACTGACGCTCAGGTGCGAAAGCGTGGGGAGCAAACAGGATT
AGATACCCTGGTAGTCCACGCTGTAAACGATGTCGACTTGGAGGTTGTGCCCTTGAGGC
GTGGCTTCCGGAGCTAACGCGTTAAGTCGACCGCCTGGGGAGTACGGCCGCAAGGTTA
AAACTCAAATGAATTGACGGGGGCCCGCACAAGCGGTGGAGCATGTGGTTTAATTCGAT
GCAACGCGAAGAACCTTACCTACTCTTGACATCCACAGAACTGAGCAGAGATGCTTAG
GTGCCTTCGGGAACTGTGAGACAGGTGCTGCATGGCTGTCGTCAGCTCGTGTTGTGAAA
TGTTGGGTTAAGTCCCGCAACGAGCGCAACCCTTATCCTTTGTTGCCAGCACGTGATGG
TGGGAACTCAAAGGAGACTGCCGGTGATAAACCGGAGGAAGGTGGGGATGACGTCAA
GTCATCATGGCCCTTACGAGTAGGGCTACACACGTGCTACAATGGCAGATACAAAGTGA
AGCGAACTCGCGAGAGCAAGCGGACCACATAAAGTCTGTCGTAGTCCGGATTGGAGTC
TGCAACTCGACTCCATGAAGTCGGAATCGCTAGTAATCGTAGATCAGAATGCTACGGTG
AATACGTTCCCGGGCCTTGTACACACCGCCCGTCACACCATGGGAGTGGGTTGCAAAA
GAAGTAGGTAGCTTAACCTTCGGGAGGGCGCTTACCACTTTGTGATTCATGACTGGGG
pattern 6
CGCTGGCGGCAGGCCTAACACATGCAAGTCGAGCGGCAGCGGGAAGTAGTTTACTACT
TTGCCGGCGAGCGGCGGACGGGTGAGTAATGTCTGGGAAACTGCCTGATGGAGGGGGA
TAACTACTGGAAACGGTAGCTAATACCGCATGACCTCGCAAGAGCAAAGTGGGGGACC
TTCGGGCCTCACGCCATCGGATGTGCCCAGATGGGATTAGCTAGTAGGTGGGGTAATGG
CTCACCTAGGCGACGATCCCTAGCTGGTCTGAGAGGATGACCAGCCACACTGGAACTG
AGACACGGTCCAGACTCCTACGGGAGGCAGCAGTGGGGAATATTGCACAATGGGCGCA
AGCCTGATGCAGCCATGCCGCGTGTGTGAAGAAGGCCTTCGGGTTGTAAAGCACTTTCA
GCGAGGAGGAAGGCAGTCGTGTTAATAGCACGATTGATTGACGTTACTCGCAGAAGAA
GCACCGGCTAACTCCGTGCCAGCAGCCGCGGTAATACGGAGGGTGCAAGCGTTAATCG
GAATTACTGGGCGTAAAGCGCACGCAGGCGGTTTGTTAAGTCAGATGTGAAATCCCCGC
GCTTAACGTGGGAACTGCATTTGAAACTGGCAAGCTAGAGTCTTGTAGAGGGGGGTAG
AATTCCAGGTGTAGCGGTGAAATGCGTAGAGATCTGGAGGAATACCGGTGGCGAAGGC
GGCCCCCTGGACAAAGACTGACGCTCAGGTGCGAAAGCGTGGGGAGCAAACAGGATT
AGATACCCTGGTAGTCCACGCTGTAAACGATGTCGACTTGGAGGTTGTGCCCTTGAGGC
ATGGCTTCCGGAGCTAACGCGTTAAGTCGACCGCCTGGGGAGTACGGCCGCAAGGTTA
AAACTCAAATGAATTGACGGGGGCCCGCACAAGCGGTGGAGCATGTGGTTTAATTCGAT
GCAACGCGAAGAACCTTACCTACTCTTGACATCCACAGAACTTAGCAGAGATGCTTCGG
TGCCTTCGGGAACTGTGAGACAGGTGCTGCATGGCTGTCGTCAGCTCGTGTTGTGAAAT

GTTGGGTTAAGTCCCGCAACGAGCGCAACCCTTATCCTTTGTTGCCAGCACGTAATGGT
GGGAACTCAAGGGAGACTGCCGGTGACAAACCGGAGGAAGGTGGGGATGACGTCAAG
TCATCATGGCCCTTACGAGTAGGGCTACACACGTGCTACAATGGCAGATACAAAGTGAA
GCGAACTCGCGAGAGCAAGCGGACCACATAAAGTCTGTCGTAGTCCGGATTGGAGTCT
GCAACTCGACTCCATGAAGTCGGAATCGCTAGTAATCGTAGATCAGAATGCTACGGTGA
ATACGTTCCCGGGCCTTGTACACACCGCCCGTCACACCATGGGAGTGGGTTGCAAAAG
AAGTAGGTAGCTTAACCTTCGGGAGGGCGCTTACCACTTTGTGATTCATGACTGGGG

pattern 7

CGCTGGCGGCAGGCCTAACACATGCAAGTCGAGCGGCAGCGGGAAGTAGTTTACTACT
TTGCCGGCGAGCGGCGGACGGGTGAGTAATGTCTGGGAAACTGCCTGATGGAGGGGGA
TAACTACTGGAAACGGTAGCTAATACCGCATGACCTCGTAAGAGCAAAGTGGGGGACCT
TCGGGCCTCACGCCATCGGATGTGCCCAGATGGGATTAGCTAGTAGGTGGGGTAATGGC
TCACCTAGGCGACGATCCCTAGCTGGTCTGAGAGGATGACCAGCCACACTGGAACTGA
GACACGGTCCAGACTCCTACGGGAGGCAGCAGTGGGGAATATTGCACAATGGGCGCAA
GCCTGATGCAGCCATGCCGCGTGTGTGAAGAAGGCCTTCGGGTTGTAAAGCACTTTCAG
CGAGGAGGAAGGCAGTCGTGTTAATAGCACGATTGATTGACGTTACTCGCAGAAGAAG
CACCGGCTAACTCCGTGCCAGCAGCCGCGGTAATACGGAGGGTGCAAGCGTTAATCGG
AATTACTGGGCGTAAAGCGCACGCAGGCGGTTTGTTAAGTCAGATGTGAAATCCCCGCG
CTTAACGTGGGAACTGCATTTGAAACTGGCAAGCTAGAGTCTTGTAGAGGGGGGTAGA
ATTCCAGGTGTAGCGGTGAAATGCGTAGAGATCTGGAGGAATACCGGTGGCGAAGGCG
GCCCCCTGGACAAAGACTGACGCTCAGGTGCGAAAGCGTGGGGAGCAAACAGGATTA
GATACCCTGGTAGTCCACGCTGTAAACGATGTCGACTTGGAGGTTGTGCCCTTGAGGCG
TGGCTTCCGGAGCTAACGCGTTAAGTCGACCGCCTGGGGAGTACGGCCGCAAGGTTAA
AACTCAAATGAATTGACGGGGGCCCGCACAAGCGGTGGAGCATGTGGTTTAATTCGATG
CAACGCGAAGAACCTTACCTACTCTTGACATCCACGGAATTTAGCAGAGATGCTTTAGT
GCCTTCGGGAACCGTGAGACAGGTGCTGCATGGCTGTCGTCAGCTCGTGTTGTGAAATG
TTGGGTTAAGTCCCGCAACGAGCGCAACCCTTATCCTTTGTTGCCAGCACGTAATGGTG
GGAACTCAAGGGAGACTGCCGGTGACAAACCGGAGGAAGGTGGGGATGACGTCAAGT
CATCATGGCCCTTACGAGTAGGGCTACACACGTGCTACAATGGCAGATACAAAGTGAAG
CGAACTCGCGAGAGCAAGCGGACCACATAAAGTCTGTCGTAGTCCGGATTGGAGTCTG
CAACTCGACTCCATGAAGTCGGAATCGCTAGTAATCGTAGATCAGAATGCTACGGTGAAT
ACGTTCCCGGGCCTTGTACACACCGCCCGTCACACCATGGGAGTGGGTTGCAAAAGAA
GTAGGTAGCTTAACCTTCGGGAGGGCGCTTACCACTTTGTGATTCATGACTGGGG

pattern 8

CGCTGGCGGCAGGCCTAACACATGCAAGTCGAGCGGCAGCGGGAAGTAGTTTACTACT
TTGCCGGCGAGCGGCGGACGGGTGAGTAATGTCTGGGAAACTGCCTGATGGAGGGGGA
TAACTACTGGAAACGGTAGCTAATACCGCATGACCTCGCAAGAGCAAAGTGGGGGACC
TTCGGGCCTCACGCCATCGGATGTGCCCAGATGGGATTAGCTAGTAGGTGGGGTAATGG
CTCACCTAGGCGACGATCCCTAGCTGGTCTGAGAGGATGACCAGCCACACTGGAACTG
AGACACGGTCCAGACTCCTACGGGAGGCAGCAGTGGGGAATATTGCACAATGGGCGCA
AGCCTGATGCAGCCATGCCGCGTGTGTGAAGAAGGCCTTCGGGTTGTAAAGCACTTTCA
GCGAGGAGGAAGGCAGTCGTGTTAATAGCACGATTGATTGACGTTACTCGCAGAAGAA
GCACCGGCTAACTCCGTGCCAGCAGCCGCGGTAATACGGAGGGTGCAAGCGTTAATCG
GAATTACTGGGCGTAAAGCGCACGCAGGCGGTTTGTTAAGTCAGATGTGAAATCCCCGC

GCTTAACGTGGGAACTGCATTTGAAACTGGCAAGCTAGAGTCTTGTAGAGGGGGGTAG
AATTCCAGGTGTAGCGGTGAAATGCGTAGAGATCTGGAGGAATACCGGTGGCGAAGGC
GGCCCCCTGGACAAAGACTGACGCTCAGGTGCGAAAGCGTGGGGAGCAAACAGGATT
AGATACCCTGGTAGTCCACGCTGTAAACGATGTCGACTTGGAGGTTGTGCCCTTGAGGC
GTGGCTTCCGGAGCTAACGCGTTAAGTCGACCGCCTGGGGAGTACGGCCGCAAGGTTA
AAACTCAAATGAATTGACGGGGGCCCGCACAAGCGGTGGAGCATGTGGTTTAATTCGAT
GCAACGCGAAGAACCTTACCTACTCTTGACATCCACGGAATTTAGCAGAGATGCTTTAG
TGCCTTCGGGAACCGTGAGACAGGTGCTGCATGGCTGTCGTCAGCTCGTGTTGTGAAAT
GTTGGGTTAAGTCCCGCAACGAGCGCAACCCTTATCCTTTGTTGCCAGCACGTAATGGT
GGGAACTCAAGGGAGACTGCCGGTGACAAACCGGAGGAAGGTGGGGATGACGTCAAG
TCATCATGGCCCTTACGAGTAGGGCTACACACGTGCTACAATGGCAGATACAAAGTGAA
GCGAACTCGCGAGAGCAAGCGGACCACATAAAGTCTGTCGTAGTCCGGATTGGAGTCT
GCAACTCGACTCCATGAAGTCGGAATCGCTAGTAATCGTAGATCAGAATGCTACGGTGA
ATACGTTCCCGGGCCTTGTACACACCGCCCGTCACACCATGGGAGTGGGTTGCAAAAG
AAGTAGGTAGCTTAACCTTCGGGAGGGCGCTTACCACTTTGTGATTCATGACTGGGG
pattern 9
CGCTGGCGGCAGGCCTAACACATGCAAGTCGAGCGGCAGCGGGAAGTAGTTTACTACT
TTGCCGGCGAGCGGCGGACGGGTGAGTAATGTCTGGGAAACTGCCTGATGGAGGGGGA
TAACTACTGGAAACGGTAGCTAATACCGCATGACCTCGTAAGAGCAAAGTGGGGGACCT
TCGGGCCTCACGCCATCGGATGTGCCCAGATGGGATTAGCTAGTAGGTGGGGTAATGGC
TCACCTAGGCGACGATCCCTAGCTGGTCTGAGAGGATGACCAGCCACACTGGAACTGA
GACACGGTCCAGACTCCTACGGGAGGCAGCAGTGGGGAATATTGCACAATGGGCGCAA
GCCTGATGCAGCCATGCCGCGTGTGTGAAGAAGGCCTTCGGGTTGTAAAGCACTTTCAG
CGAGGAGGAAGGCAGTCGTGTTAATAGCACGATTGATTGACGTTACTCGCAGAAGAAG
CACCGGCTAACTCCGTGCCAGCAGCCGCGGTAATACGGAGGGTGCAAGCGTTAATCGG
AATTACTGGGCGTAAAGCGCACGCAGGCGGTTTGTTAAGTCAGATGTGAAATCCCCGCG
CTTAACGTGGGAACTGCATTTGAAACTGGCAAGCTAGAGTCTTGTAGAGGGGGGTAGA
ATTCCAGGTGTAGCGGTGAAATGCGTAGAGATCTGGAGGAATACCGGTGGCGAAGGCG
GCCCCCTGGACAAAGACTGACGCTCAGGTGCGAAAGCGTGGGGAGCAAACAGGATTA
GATACCCTGGTAGTCCACGCTGTAAACGATGTCGACTTGGAGGTTGTGCCCTTGAGGCG
TGGCTTCCGGAGCTAACGCGTTAAGTCGACCGCCTGGGGAGTACGGCCGCAAGGTTAA
AACTCAAATGAATTGACGGGGGCCCGCACAAGCGGTGGAGCATGTGGTTTAATTCGATG
CAACGCGAAGAACCTTACCTACTCTTGACATCCACAGAACTTAGCAGAGATGCTTCGGT
GCCTTCGGGAACTGTGAGACAGGTGCTGCATGGCTGTCGTCAGCTCGTGTTGTGAAATG
TTGGGTTAAGTCCCGCAACGAGCGCAACCCTTATCCTTTGTTGCCAGCACGTAATGGTG
GGAACTCAAGGGAGACTGCCGGTGACAAACCGGAGGAAGGTGGGGATGACGTCAAGT
CATCATGGCCCTTACGAGTAGGGCTACACACGTGCTACAATGGCAGATACAAAGTGAAG
CGAACTCGCGAGAGCAAGCGGACCACATAAAGTCTGTCGTAGTCCGGATTGGAGTCTG
CAACTCGACTCCATGAAGTCGGAATCGCTAGTAATCGTAGATCAGAATGCTACGGTGAAT
ACGTTCCCGGGCCTTGTACACACCGCCCGTCACACCATGGGAGTGGGTTGCAAAAGAA
GTAGGTAGCTTAACCTTCGGGAGGGCGCTTACCACTTTGTGATTCATGACTGGGG
pattern 10
CGCTGGCGGCAGGCCTAACACATGCAAGTCGAGCGGCAGCGGGAAGTAGTTTACTACT
TTGCCGGCGAGCGGCGGACGGGTGAGTAATGTCTGGGAAACTGCCTGATGGAGGGGGA

TAACTACTGGAAACGGTAGCTAATACCGCATGACCTCGCAAGAGCAAAGTGGGGGACC
TTCGGGCCTCACGCCATCGGATGTGCCCAGATGGGATTAGCTAGTAGGTGGGGTAATGG
CTCACCTAGGCGACGATCCCTAGCTGGTCTGAGAGGATGACCAGCCACACTGGAACTG
AGACACGGTCCAGACTCCTACGGGAGGCAGCAGTGGGGAATATTGCACAATGGGCGCA
AGCCTGATGCAGCCATGCCGCGTGTGTGAAGAAGGCCTTCGGGTTGTAAAGCACTTTCA
GCGAGGAGGAAGGCAGTCGTGTTAATAGCACGATTGATTGACGTTACTCGCAGAAGAA
GCACCGGCTAACTCCGTGCCAGCAGCCGCGGTAATACGGAGGGTGCAAGCGTTAATCG
GAATTACTGGGCGTAAAGCGCACGCAGGCGGTTTGTTAAGTCAGATGTGAAATCCCCGC
GCTTAACGTGGGAACTGCATTTGAAACTGGCAAGCTAGAGTCTTGTAGAGGGGGGTAG
AATTCCAGGTGTAGCGGTGAAATGCGTAGAGATCTGGAGGAATACCGGTGGCGAAGGC
GGCCCCCTGGACAAAGACTGACGCTCAGGTGCGAAAGCGTGGGGAGCAAACAGGATT
AGATACCCTGGTAGTCCACGCTGTAAACGATGTCGACTTGGAGGTTGTGCCCTTGAGGC
GTGGCTTCCGGAGCTAACGCGTTAAGTCGACCGCCTGGGGAGTACGGCCGCAAGGTTA
AAACTCAAATGAATTGACGGGGGCCCGCACAAGCGGTGGAGCATGTGGTTTAATTCGAT
GCAACGCGAAGAACCTTACCTACTCTTGACATCCACAGAACTTAGCAGAGATGCTTCGG
TGCCTTCGGGAACTGTGAGACAGGTGCTGCATGGCTGTCGTCAGCTCGTGTTGTGAAAT
GTTGGGTTAAGTCCCGCAACGAGCGCAACCCTTATCCTTTGTTGCCAGCACGTAATGGT
GGGAACTCAAGGGAGACTGCCGGTGACAAACCGGAGGAAGGTGGGGATGACGTCAAG
TCATCATGGCCCTTACGAGTAGGGCTACACACGTGCTACAATGGCAGATACAAAGTGAA
GCGAACTCGCGAGAGCAAGCGGACCACATAAAGTCTGTCGTAGTCCGGATTGGAGTCT
GCAACTCGACTCCATGAAGTCGGAATCGCTAGTAATCGTAGATCAGAATGCTACGGTGA
ATACGTTCCCGGGCCTTGTACACACCGCCCGTCACACCATGGGAGTGGGTTGCAAAAG
AAGTAGGTAGCTTAACCTTCGGGAGGGCGCTTACCACTTTGTGATTCATGACTGGGG
pattern 11
CGCTGGCGGCAGGCCTAACACATGCAAGTCGAGCGGCAGCGGAAAGTAGCTTGCTACT
TTGCCGGCGAGCGGCGGACGGGTGAGTAATGTCTGGGGATCTGCCTGATGGAGGGGGA
TAACTACTGGAAACGGTAGCTAATACCGCATGACCTCGAAAGAGCAAAGTGGGGGACC
TTCGGGCCTCACGCCATCGGATGAACCCAGATGGGATTAGCTAGTAGGTGGGGTAATGG
CTCACCTAGGCGACGATCCCTAGCTGGTCTGAGAGGATGACCAGCCACACTGGAACTG
AGACACGGTCCAGACTCCTACGGGAGGCAGCAGTGGGGAATATTGCACAATGGGCGCA
AGCCTGATGCAGCCATGCCGCGTGTGTGAAGAAGGCCTTCGGGTTGTAAAGCACTTTCA
GCGAGGAGGAAGGCATTGTGGTTAATAACCGCAGTGATTGACGTTACTCGCAGAAGAA
GCACCGGCTAACTCCGTGCCAGCAGCCGCGGTAATACGGAGGGTGCAAGCGTTAATCG
GAATTACTGGGCGTAAAGCGCACGCAGGCGGTTTGTTAAGTCAGATGTGAAATCCCCGC
GCTTAACGTGGGAACTGCATTTGAAACTGGCAAGCTAGAGTCTTGTAGAGGGGGGTAG
AATTCCAGGTGTAGCGGTGAAATGCGTAGAGATCTGGAGGAATACCGGTGGCGAAGGC
GGCCCCCTGGACAAAGACTGACGCTCAGGTGCGAAAGCGTGGGGAGCAAACAGGATT
AGATACCCTGGTAGTCCACGCTGTAAACGATGTCGACTTGGAGGTTGTGCCCTTGAGGC
GTGGCTTCCGGAGCTAACGCGTTAAGTCGACCGCCTGGGGAGTACGGCCGCAAGGTTA
AAACTCAAATGAATTGACGGGGGCCCGCACAAGCGGTGGAGCATGTGGTTTAATTCGAT
GCAACGCGAAGAACCTTACCTACTCTTGACATCCACAGAACTTAGCAGAGATGCTTCGG
TGCCTTCGGGAACTGTGAGACAGGTGCTGCATGGCTGTCGTCAGCTCGTGTTGTGAAAT
GTTGGGTTAAGTCCCGCAACGAGCGCAACCCTTATCCTTTGTTGCCAGCACGTAATGGT
GGGAACTCAAGGGAGACTGCCGGTGACAAACCGGAGGAAGGTGGGGATGACGTCAAG

TCATCATGGCCCTTACGAGTAGGGCTACACACGTGCTACAATGGCAGATACAAAGTGAA
GCGAACTCGCGAGAGCAAGCGGACCACATAAAGTCTGTCGTAGTCCGGATTGGAGTCT
GCAACTCGACTCCATGAAGTCGGAATCGCTAGTAATCGTAGATCAGAATGCTACGGTGA
ATACGTTCCCGGGCCTTGTACACACCGCCCGTCACACCATGGGAGTGGGTTGCAAAAG
AAGTAGGTAGCTTAACCTTCGGGAGGGCGCTTACCACTTTGTGATTCATGACTGGGG

pattern 12

CGCTGGCGGCAGGCCTAACACATGCAAGTCGAGCGGCAGCGGAAAGTAGCTTGCTACT
TTGCCGGCGAGCGGCGGACGGGTGAGTAATGTCTGGGGATCTGCCTGATGGAGGGGGA
TAACTACTGGAAACGGTAGCTAATACCGCATGACCTCGAAAGAGCAAAGTGGGGGACC
TTCGGGCCTCACGCCATCGGATGAACCCAGATGGGATTAGCTAGTAGGTGGGGTAATGG
CTCACCTAGGCGACGATCCCTAGCTGGTCTGAGAGGATGACCAGCCACACTGGAACTG
AGACACGGTCCAGACTCCTACGGGAGGCAGCAGTGGGGAATATTGCACAATGGGCGCA
AGCCTGATGCAGCCATGCCGCGTGTGTGAAGAAGGCCTTCGGGTTGTAAAGCACTTTCA
GCGAGGAGGAAGGCATTGTGGTTAATAACCGCAGTGATTGACGTTACTCGCAGAAGAA
GCACCGGCTAACTCCGTGCCAGCAGCCGCGGTAATACGGAGGGTGCAAGCGTTAATCG
GAATTACTGGGCGTAAAGCGCACGCAGGCGGTTTGTTAAGTCAGATGTGAAATCCCCGC
GCTTAACGTGGGAACTGCATTTGAAACTGGCAAGCTAGAGTCTTGTAGAGGGGGGTAG
AATTCCAGGTGTAGCGGTGAAATGCGTAGAGATCTGGAGGAATACCGGTGGCGAAGGC
GGCCCCCTGGACAAAGACTGACGCTCAGGTGCGAAAGCGTGGGGAGCAAACAGGATT
AGATACCCTGGTAGTCCACGCTGTAAACGATGTCGACTTGGAGGTTGTGCCCTTGAGGT
GTGGCTTCCGGAGCTAACGCGTTAAGTCGACCGCCTGGGGAGTACGGCCGCAAGGTTA
AAACTCAAATGAATTGACGGGGGCCCGCACAAGCGGTGGAGCATGTGGTTTAATTCGAT
GCAACGCGAAGAACCTTACCTACTCTTGACATCCACAGAACTTAGCAGAGATGCTTCGG
TGCCTTCGGGAACTGTGAGACAGGTGCTGCATGGCTGTCGTCAGCTCGTGTTGTGAAAT
GTTGGGTTAAGTCCCGCAACGAGCGCAACCCTTATCCTTTGTTGCCAGCACGTAATGGT
GGGAACTCAAGGGAGACTGCCGGTGACAAACCGGAGGAAGGTGGGGATGACGTCAAG
TCATCATGGCCCTTACGAGTAGGGCTACACACGTGCTACAATGGCAGATACAAAGTGAA
GCGAACTCGCGAGAGCAAGCGGACCACATAAAGTCTGTCGTAGTCCGGATTGGAGTCT
GCAACTCGACTCCATGAAGTCGGAATCGCTAGTAATCGTAGATCAGAATGCTACGGTGA
ATACGTTCCCGGGCCTTGTACACACCGCCCGTCACACCATGGGAGTGGGTTGCAAAAG
AAGTAGGTAGCTTAACCTTCGGGAGGGCGCTTACCACTTTGTGATTCATGACTGGGG

pattern 13

CGCTGGCGGCAGGCCTAACACATGCAAGTCGAGCGGCAGCGGAAAGTAGCTTGCTACT
TTGCCGGCGAGCGGCGGACGGGTGAGTAATGTCTGGGGATCTGCCTGATGGAGGGGGA
TAACTACTGGAAACGGTAGCTAATACCGCATGACCTCGAAAGAGCAAAGTGGGGGACC
TTCGGGCCTCACGCCATCGGATGAACCCAGATGGGATTAGCTAGTAGGTGGGGTAATGG
CTCACCTAGGCGACGATCCCTAGCTGGTCTGAGAGGATGACCAGCCACACTGGAACTG
AGACACGGTCCAGACTCCTACGGGAGGCAGCAGTGGGGAATATTGCACAATGGGCGCA
AGCCTGATGCAGCCATGCCGCGTGTGTGAAGAAGGCCTTCGGGTTGTAAAGCACTTTCA
GCGAGGAGGAAGGCATTGTGGTTAATAACCGCAGTGATTGACGTTACTCGCAGAAGAA
GCACCGGCTAACTCCGTGCCAGCAGCCGCGGTAATACGGAGGGTGCAAGCGTTAATCG
GAATTACTGGGCGTAAAGCGCACGCAGGCGGTTTGTTAAGTCAGATGTGAAATCCCCGC
GCTTAACGTGGGAACTGCATTTGAAACTGGCAAGCTAGAGTCTTGTAGAGGGGGGTAG
AATTCCAGGTGTAGCGGTGAAATGCGTAGAGATCTGGAGGAATACCGGTGGCGAAGGC

GGCCCCCTGGACAAAGACTGACGCTCAGGTGCGAAAGCGTGGGGAGCAAACAGGATT
AGATACCCTGGTAGTCCACGCTGTAAACGATGTCGACTTGGAGGTTGTGCCCTTGAGGC
GTGGCTTCCGGAGCTAACGCGTTAAGTCGACCGCCTGGGGAGTACGGCCGCAAGGTTA
AAACTCAAATGAATTGACGGGGGCCCGCACAAGCGGTGGAGCATGTGGTTTAATTCGAT
GCAACGCGAAGAACCTTACCTACTCTTGACATCCACGGAATTTAGCAGAGATGCTTTAG
TGCCTTCGGGAACCGTGAGACAGGTGCTGCATGGCTGTCGTCAGCTCGTGTTGTGAAAT
GTTGGGTTAAGTCCCGCAACGAGCGCAACCCTTATCCTTTGTTGCCAGCACGTAATGGT
GGGAACTCAAGGGAGACTGCCGGTGACAAACCGGAGGAAGGTGGGGATGACGTCAAG
TCATCATGGCCCTTACGAGTAGGGCTACACACGTGCTACAATGGCAGATACAAAGTGAA
GCGAACTCGCGAGAGCAAGCGGACCACATAAAGTCTGTCGTAGTCCGGATTGGAGTCT
GCAACTCGACTCCATGAAGTCGGAATCGCTAGTAATCGTAGATCAGAATGCTACGGTGA
ATACGTTCCCGGGCCTTGTACACACCGCCCGTCACACCATGGGAGTGGGTTGCAAAAG
AAGTAGGTAGCTTAACCTTCGGGAGGGCGCTTACCACTTTGTGATTCATGACTGGGG
pattern 14
CGCTGGCGGCAGGCCTAACACATGCAAGTCGAGCGGCAGCGGAAAGTAGCTTGCTACT
TTGCCGGCGAGCGGCGGACGGGTGAGTAATGTCTGGGAAACTGCCTGATGGAGGGGGA
TAACTACTGGAAACGGTAGCTAATACCGCATGACCTCGAAAGAGCAAAGTGGGGGACC
TTCGGGCCTCACGCCATCGGATGTGCCCAGATGGGATTAGCTAGTAGGTGGGGTAATGG
CTCACCTAGGCGACGATCCCTAGCTGGTCTGAGAGGATGACCAGCCACACTGGAACTG
AGACACGGTCCAGACTCCTACGGGAGGCAGCAGTGGGGAATATTGCACAATGGGCGCA
AGCCTGATGCAGCCATGCCGCGTGTGTGAAGAAGGCCTTCGGGTTGTAAAGCACTTTCA
GCGAGGAGGAAGGCATTTCACTTAATACGTGAGGTGATTGACGTTACTCGCAGAAGAA
GCACCGGCTAACTCCGTGCCAGCAGCCGCGGTAATACGGAGGGTGCAAGCGTTAATCG
GAATTACTGGGCGTAAAGCGCACGCAGGCGGTTTGTTAAGTCAGATGTGAAATCCCCGA
GCTTAACTTGGGAACTGCATTTGAAACTGGCAAGCTAGAGTCTTGTAGAGGGGGGTAG
AATTCCAGGTGTAGCGGTGAAATGCGTAGAGATCTGGAGGAATACCGGTGGCGAAGGC
GGCCCCCTGGACAAAGACTGACGCTCAGGTGCGAAAGCGTGGGGAGCAAACAGGATT
AGATACCCTGGTAGTCCACGCTGTAAACGATGTCGACTTGGAGGTTGTGCCCTTGAGGC
GTGGCTTCCGGAGCTAACGCGTTAAGTCGACCGCCTGGGGAGTACGGCCGCAAGGTTA
AAACTCAAATGAATTGACGGGGGCCCGCACAAGCGGTGGAGCATGTGGTTTAATTCGAT
GCAACGCGAAGAACCTTACCTACTCTTGACATCCACAGAACTTAGCAGAGATGCTTAGG
TGCCTTCGGGAACTGTGAGACAGGTGCTGCATGGCTGTCGTCAGCTCGTGTTGTGAAAT
GTTGGGTTAAGTCCCGCAACGAGCGCAACCCTTATCCTTTGTTGCCAGCACGTAATGGT
GGGAACTCAAAGGAGACTGCCGGTGATAAACCGGAGGAAGGTGGGGATGACGTCAAG
TCATCATGGCCCTTACGAGTAGGGCTACACACGTGCTACAATGGCAGATACAAAGTGAA
GCGAACTCGCGAGAGCAAGCGGACCACATAAAGTCTGTCGTAGTCCGGATTGGAGTCT
GCAACTCGACTCCATGAAGTCGGAATCGCTAGTAATCGTAGATCAGAATGCTACGGTGA
ATACGTTCCCGGGCCTTGTACACACCGCCCGTCACACCATGGGAGTGGGTTGCAAAAG
AAGTAGGTAGCTTAACCTTCGGGAGGGCGCTTACCACTTTGTGATTCATGACTGGGg
pattern 15
CGCTGGCGGCAGGCCTAACACATGCAAGTCGAGCGGCAGCGGAAAGTAGCTTGCTACT
TTGCCGGCGAGCGGCGGACGGGTGAGTAATGTCTGGGGATCTGCCTGATGGAGGGGGA
TAACTACTGGAAACGGTAGCTAATACCGCATGACCTCGAAAGAGCAAAGTGGGGGACC
TTCGGGCCTCACGCCATCGGATGAACCCAGATGGGATTAGCTAGTAGGTGAGGTAATGG

CTCACCTAGGCGACGATCCCTAGCTGGTCTGAGAGGATGACCAGCCACACTGGAACTG
AGACACGGTCCAGACTCCTACGGGAGGCAGCAGTGGGGAATATTGCACAATGGGCGCA
AGCCTGATGCAGCCATGCCGCGTGTGTGAAGAAGGCCTTCGGGTTGTAAAGCACTTTCA
GCGAGGAGGAAGGCATTGTGGTTAATAACCGCAGTGATTGACGTTACTCGCAGAAGAA
GCACCGGCTAACTCCGTGCCAGCAGCCGCGGTAATACGGAGGGTGCAAGCGTTAATCG
GAATTACTGGGCGTAAAGCGCACGCAGGCGGTTTGTTAAGTCAGATGTGAAATCCCCGC
GCTTAACGTGGGAACTGCATTTGAAACTGGCAAGCTAGAGTCTTGTAGAGGGGGGTAG
AATTCCAGGTGTAGCGGTGAAATGCGTAGAGATCTGGAGGAATACCGGTGGCGAAGGC
GGCCCCCTGGACAAAGACTGACGCTCAGGTGCGAAAGCGTGGGGAGCAAACAGGATT
AGATACCCTGGTAGTCCACGCTGTAAACGATGTCGACTTGGAGGTTGTGCCCTTGAGGC
GTGGCTTCCGGAGCTAACGCGTTAAGTCGACCGCCTGGGGAGTACGGCCGCAAGGTTA
AAACTCAAATGAATTGACGGGGGCCCGCACAAGCGGTGGAGCATGTGGTTTAATTCGAT
GCAACGCGAAGAACCTTACCTACTCTTGACATCCACGGAATTTAGCAGAGATGCTTTAG
TGCCTTCGGGAACCGTGAGACAGGTGCTGCATGGCTGTCGTCAGCTCGTGTTGTGAAAT
GTTGGGTTAAGTCCCGCAACGAGCGCAACCCTTATCCTTTGTTGCCAGCACGTAATGGT
GGGAACTCAAGGGAGACTGCCGGTGACAAACCGGAGGAAGGTGGGGATGACGTCAAG
TCATCATGGCCCTTACGAGTAGGGCTACACACGTGCTACAATGGCAGATACAAAGTGAA
GCGAACTCGCGAGAGCAAGCGGACCACATAAAGTCTGTCGTAGTCCGGATTGGAGTCT
GCAACTCGACTCCATGAAGTCGGAATCGCTAGTAATCGTAGATCAGAATGCTACGGTGA
ATACGTTCCCGGGCCTTGTACACACCGCCCGTCACACCATGGGAGTGGGTTGCAAAAG
AAGTAGGTAGCTTAACCTTCGGGAGGGCGCTTACCACTTTGTGATTCATGACTGGGG

pattern 16

CGCTGGCGGCAGGCCTAACACATGCAAGTCGAGCGGCAGCGGAAAGTAGCTTGCTACT
TTGCCGGCGAGCGGCGGACGGGTGAGTAATGTCTGGGGATCTGCCTGATGGAGGGGGA
TAACTACTGGAAACGGTAGCTAATACCGCATGACCTCGAAAGAGCAAAGTGGGGGACC
TTCGGGCCTCACGCCATCGGATGAACCCAGATGGGATTAGCTAGTAGGTGAGGTAATGG
CTCACCTAGGCGACGATCCCTAGCTGGTCTGAGAGGATGACCAGCCACACTGGAACTG
AGACACGGTCCAGACTCCTACGGGAGGCAGCAGTGGGGAATATTGCACAATGGGCGCA
AGCCTGATGCAGCCATGCCGCGTGTGTGAAGAAGGCCTTCGGGTTGTAAAGCACTTTCA
GCGAGGAGGAAGGCATTGTGGTTAATAACCGCAGTGATTGACGTTACTCGCAGAAGAA
GCACCGGCTAACTCCGTGCCAGCAGCCGCGGTAATACGGAGGGTGCAAGCGTTAATCG
GAATTACTGGGCGTAAAGCGCACGCAGGCGGTTTGTTAAGTCAGATGTGAAATCCCCGC
GCTTAACGTGGGAACTGCATTTGAAACTGGCAAGCTAGAGTCTTGTAGAGGGGGGTAG
AATTCCAGGTGTAGCGGTGAAATGCGTAGAGATCTGGAGGAATACCGGTGGCGAAGGC
GGCCCCCTGGACAAAGACTGACGCTCAGGTGCGAAAGCGTGGGGAGCAAACAGGATT
AGATACCCTGGTAGTCCACGCTGTAAACGATGTCGACTTGGAGGTTGTGCCCTTGAGGC
GTGGCTTCCGGAGCTAACGCGTTAAGTCGACCGCCTGGGGAGTACGGCCGCAAGGTTA
AAACTCAAATGAATTGACGGGGGCCCGCACAAGCGGTGGAGCATGTGGTTTAATTCGAT
GCAACGCGAAGAACCTTACCTACTCTTGACATCCACAGAACTTAGCAGAGATGCTTCGG
TGCCTTCGGGAACTGTGAGACAGGTGCTGCATGGCTGTCGTCAGCTCGTGTTGTGAAAT
GTTGGGTTAAGTCCCGCAACGAGCGCAACCCTTATCCTTTGTTGCCAGCACGTAATGGT
GGGAACTCAAGGGAGACTGCCGGTGACAAACCGGAGGAAGGTGGGGATGACGTCAAG
TCATCATGGCCCTTACGAGTAGGGCTACACACGTGCTACAATGGCAGATACAAAGTGAA
GCGAACTCGCGAGAGCAAGCGGACCACATAAAGTCTGTCGTAGTCCGGATTGGAGTCT

GCAACTCGACTCCATGAAGTCGGAATCGCTAGTAATCGTAGATCAGAATGCTACGGTGA
ATACGTTCCCGGGCCTTGTACACACCGCCCGTCACACCATGGGAGTGGGTTGCAAAAG
AAGTAGGTAGCTTAACCTTCGGGAGGGCGCTTACCACTTTGTGATTCATGACTGGGG

pattern 17

CGCTGGCGGCAGGCCTAACACATGCAAGTCGAGCGGCAGCGGAAAGTAGCTTGCTACT
TTGCCGGCGAGCGGCGGACGGGTGAGTAATGTCTGGGGATCTGCCTGATGGAGGGGGA
TAACTACTGGAAACGGTAGCTAATACCGCATGACCTCGAAAGAGCAAAGTGGGGGACC
TTCGGGCCTCACGCCATCGGATGAACCCAGATGGGATTAGCTAGTAGGTGAGGTAATGG
CTCACCTAGGCGACGATCCCTAGCTGGTCTGAGAGGATGACCAGCCACACTGGAACTG
AGACACGGTCCAGACTCCTACGGGAGGCAGCAGTGGGGAATATTGCACAATGGGCGCA
AGCCTGATGCAGCCATGCCGCGTGTGTGAAGAAGGCCTTCGGGTTGTAAAGCACTTTCA
GCGAGGAGGAAGGCATTGTGGTTAATAACCGCAGTGATTGACGTTACTCGCAGAAGAA
GCACCGGCTAACTCCGTGCCAGCAGCCGCGGTAATACGGAGGGTGCAAGCGTTAATCG
GAATTACTGGGCGTAAAGCGCACGCAGGCGGTTTGTTAAGTCAGATGTGAAATCCCCGC
GCTTAACGTGGGAACTGCATTTGAAACTGGCAAGCTAGAGTCTTGTAGAGGGGGGTAG
AATTCCAGGTGTAGCGGTGAAATGCGTAGAGATCTGGAGGAATACCGGTGGCGAAGGC
GGCCCCCTGGACAAAGACTGACGCTCAGGTGCGAAAGCGTGGGGAGCAAACAGGATT
AGATACCCTGGTAGTCCACGCTGTAAACGATGTCGACTTGGAGGTTGTGCCCTTGAGGT
GTGGCTTCCGGAGCTAACGCGTTAAGTCGACCGCCTGGGGAGTACGGCCGCAAGGTTA
AAACTCAAATGAATTGACGGGGGCCCGCACAAGCGGTGGAGCATGTGGTTTAATTCGAT
GCAACGCGAAGAACCTTACCTACTCTTGACATCCACAGAACTTAGCAGAGATGCTTCGG
TGCCTTCGGGAACTGTGAGACAGGTGCTGCATGGCTGTCGTCAGCTCGTGTTGTGAAAT
GTTGGGTTAAGTCCCGCAACGAGCGCAACCCTTATCCTTTGTTGCCAGCACGTAATGGT
GGGAACTCAAGGGAGACTGCCGGTGACAAACCGGAGGAAGGTGGGGATGACGTCAAG
TCATCATGGCCCTTACGAGTAGGGCTACACACGTGCTACAATGGCAGATACAAAGTGAA
GCGAACTCGCGAGAGCAAGCGGACCACATAAAGTCTGTCGTAGTCCGGATTGGAGTCT
GCAACTCGACTCCATGAAGTCGGAATCGCTAGTAATCGTAGATCAGAATGCTACGGTGA
ATACGTTCCCGGGCCTTGTACACACCGCCCGTCACACCATGGGAGTGGGTTGCAAAAG
AAGTAGGTAGCTTAACCTTCGGGAGGGCGCTTACCACTTTGTGATTCATGACTGGGG

pattern 18

CGCTGGCGGCAGGCCTAACACATGCAAGTCGAGCGGCAGCGGGAAGTAGTTTACTACT
TTGCCGGCGAGCGGCGGACGGGTGAGTAATGTCTGGGGATCTGCCTGATGGAGGGGGA
TAACTACTGGAAACGGTGGCTAATACCGCGTGACCTCGCAAGAGCAAAGTGGGGGACC
TTAGGGCCTCACGCCATCGGATGAACCCAGATGGGATTAGCTAGTAGGTGGGGTAATGG
CTCACCTAGGCGACGATCCCTAGCTGGTCTGAGAGGATGACCAGCCACACTGGAACTG
AGACACGGTCCAGACTCCTACGGGAGGCAGCAGTGGGGAATATTGCACAATGGGCGCA
AGCCTGATGCAGCCATGCCGCGTGTGTGAAGAAGGCCTTCGGGTTGTAAAGCACTTTCA
GCGAGGAGGAAGGGGTTGAGTTTAATACGCTCAATCATTGACGTTACTCGCAGAAGAA
GCACCGGCTAACTCCGTGCCAGCAGCCGCGGTAATACGGAGGGTGCAAGCGTTAATCG
GAATTACTGGGCGTAAAGCGCACGCAGGCGGTTTGTTAAGTCAGATGTGAAATCCCCGC
GCTTAACGTGGGAACTGCATTTGAAACTGGCAAGCTAGAGTCTTGTAGAGGGGGGTAG
AATTCCAGGTGTAGCGGTGAAATGCGTAGAGATCTGGAGGAATACCGGTGGCGAAGGC
GGCCCCCTGGACAAAGACTGACGCTCAGGTGCGAAAGCGTGGGGAGCAAACAGGATT
AGATACCCTGGTAGTCCACGCTGTAAACGATGTCGACTTGGAGGTTGTGCCCTTGAGGC

GTGGCTTCCGGAGCTAACGCGTTAAGTCGACCGCCTGGGGAGTACGGCCGCAAGGTTA
AAACTCAAATGAATTGACGGGGGCCCGCACAAGCGGTGGAGCATGTGGTTTAATTCGAT
GCAACGCGAAGAACCTTACCTACTCTTGACATCCACAGAATTTGGCAGAGATGCTAAAG
TGCCTTCGGGAACTGTGAGACAGGTGCTGCATGGCTGTCGTCAGCTCGTGTTGTGAAAT
GTTGGGTTAAGTCCCGCAACGAGCGCAACCCTTATCCTTTGTTGCCAGCACGTAATGGT
GGGAACTCAAGGGAGACTGCCGGTGACAAACCGGAGGAAGGTGGGGATGACGTCAAG
TCATCATGGCCCTTACGAGTAGGGCTACACACGTGCTACAATGGCAGATACAAAGTGAA
GCGAACTCGCGAGAGCCAGCGGACCACATAAAGTCTGTCGTAGTCCGGATTGGAGTCT
GCAACTCGACTCCATGAAGTCGGAATCGCTAGTAATCGTAGATCAGAATGCTACGGTGA
ATACGTTCCCGGGCCTTGTACACACCGCCCGTCACACCATGGGAGTGGGTTGCAAAAG
AAGTAGGTAGCTTAACCTTCGGGAGGGCGCTTACCACTTTGTGATTCATGACTGGGG
pattern 19
CGCTGGCGGCAGGCCTAACACATGCAAGTCGAGCGGCAGCGGGAAGTAGTTTACTACT
TTGCCGGCGAGCGGCGGACGGGTGAGTAATGTCTGGGAAACTGCCTGATGGAGGGGGA
TAACTACTGGAAACGGTAGCTAATACCGCATAACGTCTTCGGACCAAAGTGGGGGACCT
TCGGGCCTCACGCCATCGGATGTGCCCAGATGGGATTAGCTAGTAGGTGGGGTAATGGC
TCACCTAGGCGACGATCCCTAGCTGGTCTGAGAGGATGACCAGCCACACTGGAACTGA
GACACGGTCCAGACTCCTACGGGAGGCAGCAGTGGGGAATATTGCACAATGGGCGCAA
GCCTGATGCAGCCATGCCGCGTGTGTGAAGAAGGCCTTCGGGTTGTAAAGCACTTTCAG
CGAGGAGGAAGGCATAAAGGTTAATAACCTTTGTGATTGACGTTACTCGCAGAAGAAG
CACCGGCTAACTCCGTGCCAGCAGCCGCGGTAATACGGAGGGTGCAAGCGTTAATCGG
AATTACTGGGCGTAAAGCGCACGCAGGCGGTTTGTTAAGTCAGATGTGAAATCCCCGCG
CTTAACGTGGGAACTGCATTTGAAACTGGCAAGCTAGAGTCTTGTAGAGGGGGGTAGA
ATTCCAGGTGTAGCGGTGAAATGCGTAGAGATCTGGAGGAATACCGGTGGCGAAGGCG
GCCCCCTGGACAAAGACTGACGCTCAGGTGCGAAAGCGTGGGGAGCAAACAGGATTA
GATACCCTGGTAGTCCACGCTGTAAACGATGTCGACTTGGAGGTTGTGCCCTTGAGGCG
TGGCTTCCGGAGCTAACGCGTTAAGTCGACCGCCTGGGGAGTACGGCCGCAAGGTTAA
AACTCAAATGAATTGACGGGGGCCCGCACAAGCGGTGGAGCATGTGGTTTAATTCGATG
CAACGCGAAGAACCTTACCTACTCTTGACATCCACGGAATTTAGCAGAGATGCTTTAGT
GCCTTCGGGAACCGTGAGACAGGTGCTGCATGGCTGTCGTCAGCTCGTGTTGTGAAATG
TTGGGTTAAGTCCCGCAACGAGCGCAACCCTTATCCTTTGTTGCCAGCACGTCATGGTG
GGAACTCAAAGGAGACTGCCGGTGATAAACCGGAGGAAGGTGGGGATGACGTCAAGT
CATCATGGCCCTTACGAGTAGGGCTACACACGTGCTACAATGGCAGATACAAAGTGAAG
CGAACTCGCGAGAGCAAGCGGACCACATAAAGTCTGTCGTAGTCCGGATTGGAGTCTG
CAACTCGACTCCATGAAGTCGGAATCGCTAGTAATCGTAGATCAGAATGCTACGGTGAAT
ACGTTCCCGGGCCTTGTACACACCGCCCGTCACACCATGGGAGTGGGTTGCAAAAGAA
GTAGGTAGCTTAACCTTCGGGAGGGCGCTTACCACTTTGTGATTCATGACTGGGG
pattern 20
CGCTGGCGGCAGGCCTAACACATGCAAGTCGAGCGGCAGCGGGAAGTAGTTTACTACT
TTGCCGGCGAGCGGCGGACGGGTGAGTAATGTCTGGGAAACTGCCTGATGGAGGGGGA
TAACTACTGGAAACGGTAGCTAATACCGCATAACGTCTACGGACCAAAGTGGGGGACCT
TCGGGCCTCACGCCATCGGATGTGCCCAGATGGGATTAGCTAGTAGGTGGGGTAATGGC
TCACCTAGGCGACGATCCCTAGCTGGTCTGAGAGGATGACCAGCCACACTGGAACTGA
GACACGGTCCAGACTCCTACGGGAGGCAGCAGTGGGGAATATTGCACAATGGGCGCAA

GCCTGATGCAGCCATGCCGCGTGTGTGAAGAAGGCCTTCGGGTTGTAAAGCACTTTCAG
CGAGGAGGAAGGCATAAAGGTTAATAACCTTTGTGATTGACGTTACTCGCAGAAGAAG
CACCGGCTAACTCCGTGCCAGCAGCCGCGGTAATACGGAGGGTGCAAGCGTTAATCGG
AATTACTGGGCGTAAAGCGCACGCAGGCGGTTTGTTAAGTCAGATGTGAAATCCCCGCG
CTTAACGTGGGAACTGCATTTGAAACTGGCAAGCTAGAGTCTTGTAGAGGGGGGTAGA
ATTCCAGGTGTAGCGGTGAAATGCGTAGAGATCTGGAGGAATACCGGTGGCGAAGGCG
GCCCCCTGGACAAAGACTGACGCTCAGGTGCGAAAGCGTGGGGAGCAAACAGGATTA
GATACCCTGGTAGTCCACGCTGTAAACGATGTCGACTTGGAGGTTGTGCCCTTGAGGCG
TGGCTTCCGGAGCTAACGCGTTAAGTCGACCGCCTGGGGAGTACGGCCGCAAGGTTAA
AACTCAAATGAATTGACGGGGGCCCGCACAAGCGGTGGAGCATGTGGTTTAATTCGATG
CAACGCGAAGAACCTTACCTACTCTTGACATCCACAGAACTTAGCAGAGATGCTTCGGT
GCCTTCGGGAACTGTGAGACAGGTGCTGCATGGCTGTCGTCAGCTCGTGTTGTGAAATG
TTGGGTTAAGTCCCGCAACGAGCGCAACCCTTATCCTTTGTTGCCAGCACGTCATGGTG
GGAACTCAAAGGAGACTGCCGGTGATAAACCGGAGGAAGGTGGGGATGACGTCAAGT
CATCATGGCCCTTACGAGTAGGGCTACACACGTGCTACAATGGCAGATACAAAGTGAAG
CGAACTCGCGAGAGCAAGCGGACCACATAAAGTCTGTCGTAGTCCGGATTGGAGTCTG
CAACTCGACTCCATGAAGTCGGAATCGCTAGTAATCGTAGATCAGAATGCTACGGTGAAT
ACGTTCCCGGGCCTTGTACACACCGCCCGTCACACCATGGGAGTGGGTTGCAAAAGAA
GTAGGTAGCTTAACCTTCGGGAGGGCGCTTACCACTTTGTGATTCATGACTGGGG
pattern 21
CGCTGGCGGCAGGCCTAACACATGCAAGTCGAGCGGCAGCGGGAAGTAGTTTACTACT
TTGCCGGCGAGCGGCGGACGGGTGAGTAATGTCTGGGAAACTGCCTGATGGAGGGGGA
TAACTACTGGAAACGGTAGCTAATACCGCATAACGTCTACGGACCAAAGTGGGGGACCT
TCGGGCCTCACGCCATCGGATGTGCCCAGATGGGATTAGCTAGTAGGTGGGGTAATGGC
TCACCTAGGCGACGATCCCTAGCTGGTCTGAGAGGATGACCAGCCACACTGGAACTGA
GACACGGTCCAGACTCCTACGGGAGGCAGCAGTGGGGAATATTGCACAATGGGCGCAA
GCCTGATGCAGCCATGCCGCGTGTGTGAAGAAGGCCTTCGGGTTGTAAAGCACTTTCAG
CGAGGAGGAAGGCATAAAGGTTAATAACCTTTGTGATTGACGTTACTCGCAGAAGAAG
CACCGGCTAACTCCGTGCCAGCAGCCGCGGTAATACGGAGGGTGCAAGCGTTAATCGG
AATTACTGGGCGTAAAGCGCACGCAGGCGGTTTGTTAAGTCAGATGTGAAATCCCCGCG
CTTAACGTGGGAACTGCATTTGAAACTGGCAAGCTAGAGTCTTGTAGAGGGGGGTAGA
ATTCCAGGTGTAGCGGTGAAATGCGTAGAGATCTGGAGGAATACCGGTGGCGAAGGCG
GCCCCCTGGACAAAGACTGACGCTCAGGTGCGAAAGCGTGGGGAGCAAACAGGATTA
GATACCCTGGTAGTCCACGCTGTAAACGATGTCGACTTGGAGGTTGTGCCCTTGAGGCG
TGGCTTCCGGAGCTAACGCGTTAAGTCGACCGCCTGGGGAGTACGGCCGCAAGGTTAA
AACTCAAATGAATTGACGGGGGCCCGCACAAGCGGTGGAGCATGTGGTTTAATTCGATG
CAACGCGAAGAACCTTACCTACTCTTGACATCCACGGAATTTAGCAGAGATGCTTTAGT
GCCTTCGGGAACCGTGAGACAGGTGCTGCATGGCTGTCGTCAGCTCGTGTTGTGAAATG
TTGGGTTAAGTCCCGCAACGAGCGCAACCCTTATCCTTTGTTGCCAGCACGTCATGGTG
GGAACTCAAAGGAGACTGCCGGTGATAAACCGGAGGAAGGTGGGGATGACGTCAAGT
CATCATGGCCCTTACGAGTAGGGCTACACACGTGCTACAATGGCAGATACAAAGTGAAG
CGAACTCGCGAGAGCAAGCGGACCACATAAAGTCTGTCGTAGTCCGGATTGGAGTCTG
CAACTCGACTCCATGAAGTCGGAATCGCTAGTAATCGTAGATCAGAATGCTACGGTGAAT
ACGTTCCCGGGCCTTGTACACACCGCCCGTCACACCATGGGAGTGGGTTGCAAAAGAA

GTAGGTAGCTTAACCTTCGGGAGGGCGCTTACCACTTTGTGATTCATGACTGGGG

pattern 22

CGCTGGCGGCAGGCCTAACACATGCAAGTCGAGCGGCAGCGGAAAGTAGCTTGCTACT
TTGCCGGCGAGCGGCGGACGGGTGAGTAATGTCTGGGGATCTGCCTGATGGAGGGGGA
TAACTACTGGAAACGGTAGCTAATACCGCATGACCTCGAAAGAGCAAAGTGGGGGACC
TTCGGGCCTCACGCCATCGGATGAACCCAGATGGGATTAGCTAGTAGGTGGGGTAATGG
CTCACCTAGGCGACGATCCCTAGCTGGTCTGAGAGGATGACCAGCCACACTGGAACTG
AGACACGGTCCAGACTCCTACGGGAGGCAGCAGTGGGGAATATTGCACAATGGGCGCA
AGCCTGATGCAGCCATGCCGCGTGTGTGAAGAAGGCCTTCGGGTTGTAAAGCACTTTCA
GCGAGGAGGAAGGCATTGTGGTTAATAACCGCAGTGATTGACGTTACTCGCAGAAGAA
GCACCGGCTAACTCCGTGCCAGCAGCCGCGGTAATACGGAGGGTGCAAGCGTTAATCG
GAATTACTGGGCGTAAAGCGCACGCAGGCGGTTTGTTAAGTCAGATGTGAAATCCCCGC
GCTTAACGTGGGAACTGCATTTGAAACTGGCAAGCTAGAGTCTTGTAGAGGGGGGTAG
AATTCCAGGTGTAGCGGTGAAATGCGTAGAGATCTGGAGGAATACCGGTGGCGAAGGC
GGCCCCCTGGACAAAGACTGACGCTCAGGTGCGAAAGCGTGGGGAGCAAACAGGATT
AGATACCCTGGTAGTCCACGCTGTAAACGATGTCGACTTGGAGGTTGTGCCCTTGAGGT
GTGGCTTCCGGAGCTAACGCGTTAAGTCGACCGCCTGGGGAGTACGGCCGCAAGGTTA
AAACTCAAATGAATTGACGGGGGCCCGCACAAGCGGTGGAGCATGTGGTTTAATTCGAT
GCAACGCGAAGAACCTTACCTACTCTTGACATCCACGGAATTTAGCAGAGATGCTTTAG
TGCCTTCGGGAACCGTGAGACAGGTGCTGCATGGCTGTCGTCAGCTCGTGTTGTGAAAT
GTTGGGTTAAGTCCCGCAACGAGCGCAACCCTTATCCTTTGTTGCCAGCACGTAATGGT
GGGAACTCAAGGGAGACTGCCGGTGACAAACCGGAGGAAGGTGGGGATGACGTCAAG
TCATCATGGCCCTTACGAGTAGGGCTACACACGTGCTACAATGGCAGATACAAAGTGAA
GCGAACTCGCGAGAGCAAGCGGACCACATAAAGTCTGTCGTAGTCCGGATTGGAGTCT
GCAACTCGACTCCATGAAGTCGGAATCGCTAGTAATCGTAGATCAGAATGCTACGGTGA
ATACGTTCCCGGGCCTTGTACACACCGCCCGTCACACCATGGGAGTGGGTTGCAAAAG
AAGTAGGTAGCTTAACCTTCGGGAGGGCGCTTACCACTTTGTGATTCATGACTGGGG

pattern 23

CGCTGGCGGCAGGCCTAACACATGCAAGTCGAGCGGCAGCGGAAAGTAGCTTGCTACT
TTGCCGGCGAGCGGCGGACGGGTGAGTAATGTCTGGGGATCTGCCTGATGGAGGGGGA
TAACTACTGGAAACGGTAGCTAATACCGCATGACCTCGAAAGAGCAAAGTGGGGGACC
TTCGGGCCTCACGCCATCGGATGAACCCAGATGGGATTAGCTAGTAGGTGGGGTAATGG
CTCACCTAGGCGACGATCCCTAGCTGGTCTGAGAGGATGACCAGCCACACTGGAACTG
AGACACGGTCCAGACTCCTACGGGAGGCAGCAGTGGGGAATATTGCACAATGGGCGCA
AGCCTGATGCAGCCATGCCGCGTGTGTGAAGAAGGCCTTCGGGTTGTAAAGCACTTTCA
GCGAGGAGGAAGGCATTGTGGTTAATAACCACAGTGATTGACGTTACTCGCAGAAGAA
GCACCGGCTAACTCCGTGCCAGCAGCCGCGGTAATACGGAGGGTGCAAGCGTTAATCG
GAATTACTGGGCGTAAAGCGCACGCAGGCGGTTTGTTAAGTCAGATGTGAAATCCCCGC
GCTTAACGTGGGAACTGCATTTGAAACTGGCAAGCTAGAGTCTTGTAGAGGGGGGTAG
AATTCCAGGTGTAGCGGTGAAATGCGTAGAGATCTGGAGGAATACCGGTGGCGAAGGC
GGCCCCCTGGACAAAGACTGACGCTCAGGTGCGAAAGCGTGGGGAGCAAACAGGATT
AGATACCCTGGTAGTCCACGCTGTAAACGATGTCGACTTGGAGGTTGTGCCCTTGAGGC
GTGGCTTCCGGAGCTAACGCGTTAAGTCGACCGCCTGGGGAGTACGGCCGCAAGGTTA
AAACTCAAATGAATTGACGGGGGCCCGCACAAGCGGTGGAGCATGTGGTTTAATTCGAT

GCAACGCGAAGAACCTTACCTACTCTTGACATCCACAGAACTTAGCAGAGATGCTTCGG
TGCCTTCGGGAACTGTGAGACAGGTGCTGCATGGCTGTCGTCAGCTCGTGTTGTGAAAT
GTTGGGTTAAGTCCCGCAACGAGCGCAACCCTTATCCTTTGTTGCCAGCACGTAATGGT
GGGAACTCAAGGGAGACTGCCGGTGACAAACCGGAGGAAGGTGGGGATGACGTCAAG
TCATCATGGCCCTTACGAGTAGGGCTACACACGTGCTACAATGGCAGATACAAAGTGAA
GCGAACTCGCGAGAGCAAGCGGACCACATAAAGTCTGTCGTAGTCCGGATTGGAGTCT
GCAACTCGACTCCATGAAGTCGGAATCGCTAGTAATCGTAGATCAGAATGCTACGGTGA
ATACGTTCCCGGGCCTTGTACACACCGCCCGTCACACCATGGGAGTGGGTTGCAAAAG
AAGTAGGTAGCTTAACCTTCGGGAGGGCGCTTACCACTTTGTGATTCATGACTGGGG
pattern 24
CGCTGGCGGCAGGCCTAACACATGCAAGTCGAGCGGCAGCGGGAAGTAGTTTACTACT
TTGCCGGCGAGCGGCGGACGGGTGAGTAATGTCTGGGAAACTGCCTGATGGAGGGGGA
TAACTACTGGAAACGGTAGCTAATACCGCATGACCTCGTAAGAGCAAAGTGGGGGACCT
TCGGGCCTCACGCCATCGGATGTGCCCAGATGGGATTAGCTAGTAGGTGGGGTAATGGC
TCACCTAGGCGACGATCCCTAGCTGGTCTGAGAGGATGACCAGCCACACTGGAACTGA
GACACGGTCCAGACTCCTACGGGAGGCAGCAGTGGGGAATATTGCACAATGGGCGCAA
GCCTGATGCAGCCATGCCGCGTGTGTGAAGAAGGCCTTCGGGTTGTAAAGCACTTTCAG
CGAGGAGGAAGGCAGTCGTGTTAATAGCACGGTTGATTGACGTTACTCGCAGAAGAAG
CACCGGCTAACTCCGTGCCAGCAGCCGCGGTAATACGGAGGGTGCAAGCGTTAATCGG
AATTACTGGGCGTAAAGCGCACGCAGGCGGTTTGTTAAGTCAGATGTGAAATCCCCGCG
CTTAACGTGGGAACTGCATTTGAAACTGGCAAGCTAGAGTCTTGTAGAGGGGGGTAGA
ATTCCAGGTGTAGCGGTGAAATGCGTAGAGATCTGGAGGAATACCGGTGGCGAAGGCG
GCCCCCTGGACAAAGACTGACGCTCAGGTGCGAAAGCGTGGGGAGCAAACAGGATTA
GATACCCTGGTAGTCCACGCTGTAAACGATGTCGACTTGGAGGTTGTGCCCTTGAGGCG
TGGCTTCCGGAGCTAACGCGTTAAGTCGACCGCCTGGGGAGTACGGCCGCAAGGTTAA
AACTCAAATGAATTGACGGGGGCCCGCACAAGCGGTGGAGCATGTGGTTTAATTCGATG
CAACGCGAAGAACCTTACCTACTCTTGACATCCACAGAACTTAGCAGAGATGCTTAGGT
GCCTTCGGGAACTGTGAGACAGGTGCTGCATGGCTGTCGTCAGCTCGTGTTGTGAAATG
TTGGGTTAAGTCCCGCAACGAGCGCAACCCTTATCCTTTGTTGCCAGCACGTAATGGTG
GGAACTCAAGGGAGACTGCCGGTGACAAACCGGAGGAAGGTGGGGATGACGTCAAGT
CATCATGGCCCTTACGAGTAGGGCTACACACGTGCTACAATGGCAGATACAAAGTGAAG
CGAACTCGCGAGAGCAAGCGGACCACATAAAGTCTGTCGTAGTCCGGATTGGAGTCTG
CAACTCGACTCCATGAAGTCGGAATCGCTAGTAATCGTAGATCAGAATGCTACGGTGAAT
ACGTTCCCGGGCCTTGTACACACCGCCCGTCACACCATGGGAGTGGGTTGCAAAAGAA
GTAGGTAGCTTAACCTTCGGGAGGGCGCTTACCACTTTGTGATTCATGACTGGGG
pattern 25
CGCTGGCGGCAGGCCTAACACATGCAAGTCGAGCGGCAGCGGAAAGTAGCTTGCTACT
TTGCCGGCGAGCGGCGGACGGGTGAGTAATGTCTGGGAAACTGCCTGATGGAGGGGGA
TAACTACTGGAAACGGTAGCTAATACCGCATGACCTCGAAAGAGCAAAGTGGGGGACC
TTCGGGCCTCACGCCATCGGATGTGCCCAGATGGGATTAGCTAGTAGGTGGGGTAATGG
CTCACCTAGGCGACGATCCCTAGCTGGTCTGAGAGGATGACCAGCCACACTGGAACTG
AGACACGGTCCAGACTCCTACGGGAGGCAGCAGTGGGGAATATTGCACAATGGGCGCA
AGCCTGATGCAGCCATGCCGCGTGTGTGAAGAAGGCCTTCGGGTTGTAAAGCACTTTCA
GCGAGGAGGAAGGCATTTCACTTAATACGTGAAGTGATTGACGTTACTCGCAGAAGAA

GCACCGGCTAACTCCGTGCCAGCAGCCGCGGTAATACGGAGGGTGCAAGCGTTAATCG
GAATTACTGGGCGTAAAGCGCACGCAGGCGGTTTGTTAAGTCAGATGTGAAATCCCCGA
GCTTAACTTGGGAACTGCATTTGAAACTGGCAAGCTAGAGTCTTGTAGAGGGGGGTAG
AATTCCAGGTGTAGCGGTGAAATGCGTAGAGATCTGGAGGAATACCGGTGGCGAAGGC
GGCCCCCTGGACAAAGACTGACGCTCAGGTGCGAAAGCGTGGGGAGCAAACAGGATT
AGATACCCTGGTAGTCCACGCTGTAAACGATGTCGACTTGGAGGTTGTGCCCTTGAGGC
GTGGCTTCCGGAGCTAACGCGTTAAGTCGACCGCCTGGGGAGTACGGCCGCAAGGTTA
AAACTCAAATGAATTGACGGGGGCCCGCACAAGCGGTGGAGCATGTGGTTTAATTCGAT
GCAACGCGAAGAACCTTACCTACTCTTGACATCCACAGAACTTAGCAGAGATGCTTAGG
TGCCTTCGGGAACTGTGAGACAGGTGCTGCATGGCTGTCGTCAGCTCGTGTTGTGAAAT
GTTGGGTTAAGTCCCGCAACGAGCGCAACCCTTATCCTTTGTTGCCAGCACGTAATGGT
GGGAACTCAAAGGAGACTGCCGGTGATAAACTGGAGGAAGGTGGGGATGACGTCAAG
TCATCATGGCCCTTACGAGTAGGGCTACACACGTGCTACAATGGCAGATACAAAGTGAA
GCGAACTCGCGAGAGCAAGCGGACCACATAAAGTCTGTCGTAGTCCGGATTGGAGTCT
GCAACTCGACTCCATGAAGTCGGAATCGCTAGTAATCGTAGATCAGAATGCTACGGTGA
ATACGTTCCCGGGCCTTGTACACACCGCCCGTCACACCATGGGAGTGGGTTGCAAAAG
AAGTAGGTAGCTTAACCTTCGGGAGGGCGCTTACCACTTTGTGATTCATGACTGGGG
pattern 26
CGCTGGCGGCAGGCCTAACACATGCAAGTCGAGCGGCAGCGGAAAGTAGCTTGCTACT
TTGCCGGCGAGCGGCGGACGGGTGAGTAATGTCTGGGGATCTGCCTGATGGAGGGGGA
TAACTACTGGAAACGGTAGCTAATACCGCATGACCTCGAAAGAGCAAAGTGGGGGACC
TTCGGGCCTCACGCCATCGGATGAACCCAGATGGGATTAGCTAGTAGGTGGGGTAATGG
CTCACCTAGGCGACGATCCCTAGCTGGTCTGAGAGGATGACCAGCCACACTGGAACTG
AGACACGGTCCAGACTCCTACGGGAGGCAGCAGTGGGGAATATTGCACAATGGGCGCA
AGCCTGATGCAGCCATGCCGCGTGTGTGAAGAAGGCCTTCGGGTTGTAAAGCACTTTCA
GCGAGGAGGAAGGCATTGTGGTTAATAACCACAGTGATTGACGTTACTCGCAGAAGAA
GCACCGGCTAACTCCGTGCCAGCAGCCGCGGTAATACGGAGGGTGCAAGCGTTAATCG
GAATTACTGGGCGTAAAGCGCACGCAGGCGGTTTGTTAAGTCAGATGTGAAATCCCCGC
GCTTAACGTGGGAACTGCATTTGAAACTGGCAAGCTAGAGTCTTGTAGAGGGGGGTAG
AATTCCAGGTGTAGCGGTGAAATGCGTAGAGATCTGGAGGAATACCGGTGGCGAAGGC
GGCCCCCTGGACAAAGACTGACGCTCAGGTGCGAAAGCGTGGGGAGCAAACAGGATT
AGATACCCTGGTAGTCCACGCTGTAAACGATGTCGACTTGGAGGTTGTGCCCTTGAGGC
GTGGCTTCCGGAGCTAACGCGTTAAGTCGACCGCCTGGGGAGTACGGCCGCAAGGTTA
AAACTCAAATGAATTGACGGGGGCCCGCACAAGCGGTGGAGCATGTGGTTTAATTCGAT
GCAACGCGAAGAACCTTACCTACTCTTGACATCCACAGAACTTAGCAGAGATGCTTTAG
TGCCTTCGGGAACCGTGAGACAGGTGCTGCATGGCTGTCGTCAGCTCGTGTTGTGAAAT
GTTGGGTTAAGTCCCGCAACGAGCGCAACCCTTATCCTTTGTTGCCAGCACGTAATGGT
GGGAACTCAAGGGAGACTGCCGGTGACAAACCGGAGGAAGGTGGGGATGACGTCAAG
TCATCATGGCCCTTACGAGTAGGGCTACACACGTGCTACAATGGCAGATACAAAGTGAA
GCGAACTCGCGAGAGCAAGCGGACCACATAAAGTCTGTCGTAGTCCGGATTGGAGTCT
GCAACTCGACTCCATGAAGTCGGAATCGCTAGTAATCGTAGATCAGAATGCTACGGTGA
ATACGTTCCCGGGCCTTGTACACACCGCCCGTCACACCATGGGAGTGGGTTGCAAAAG
AAGTAGGTAGCTTAACCTTCGGGAGGGCGCTTACCACTTTGTGATTCATGACTGGGG
pattern 27

CGCTGGCGGCAGGCCTAACACATGCAAGTCGAGCGGCAGCGGGAAGTAGTTTACTACT
TTGCCGGCGAGCGGCGGACGGGTGAGTAATGTCTGGGAAACTGCCTGATGGAGGGGGA
TAACTACTGGAAACGGTAGCTAATACCGCATGACCTCGTAAGAGCAAAGTGGGGGACCT
TCGGGCCTCACGCCATCGGATGTGCCCAGATGGGATTAGCTAGTAGGTGGGGTAATGGC
TCACCTAGGCGACGATCCCTAGCTGGTCTGAGAGGATGACCAGCCACACTGGAACTGA
GACACGGTCCAGACTCCTACGGGAGGCAGCAGTGGGGAATATTGCACAATGGGCGCAA
GCCTGATGCAGCCATGCCGCGTGTGTGAAGAAGGCCTTCGGGTTGTAAAGCACTTTCAG
CGAGGAGGAAGGCAGTCGTGTTAATAGCACGGTTGATTGACGTTACTCGCAGAAGAAG
CACCGGCTAACTCCGTGCCAGCAGCCGCGGTAATACGGAGGGTGCAAGCGTTAATCGG
AATTACTGGGCGTAAAGCGCACGCAGGCGGTTTGTTAAGTCAGATGTGAAATCCCCGCG
CTTAACGTGGGAACTGCATTTGAAACTGGCAAGCTAGAGTCTTGTAGAGGGGGGTAGA
ATTCCAGGTGTAGCGGTGAAATGCGTAGAGATCTGGAGGAATACCGGTGGCGAAGGCG
GCCCCCTGGACAAAGACTGACGCTCAGGTGCGAAAGCGTGGGGAGCAAACAGGATTA
GATACCCTGGTAGTCCACGCTGTAAACGATGTCGACTTGGAGGTTGTGCCCTTGAGGCG
TGGCTTCCGGAGCTAACGCGTTAAGTCGACCGCCTGGGGAGTACGGCCGCAAGGTTAA
AACTCAAATGAATTGACGGGGGCCCGCACAAGCGGTGGAGCATGTGGTTTAATTCGATG
CAACGCGAAGAACCTTACCTACTCTTGACATCCACGGAATTTAGCAGAGATGCTTTAGT
GCCTTCGGGAACCGTGAGACAGGTGCTGCATGGCTGTCGTCAGCTCGTGTTGTGAAATG
TTGGGTTAAGTCCCGCAACGAGCGCAACCCTTATCCTTTGTTGCCAGCACGTAATGGTG
GGAACTCAAGGGAGACTGCCGGTGACAAACCGGAGGAAGGTGGGGATGACGTCAAGT
CATCATGGCCCTTACGAGTAGGGCTACACACGTGCTACAATGGCAGATACAAAGTGAAG
CGAACTCGCGAGAGCAAGCGGACCACATAAAGTCTGTCGTAGTCCGGATTGGAGTCTG
CAACTCGACTCCATGAAGTCGGAATCGCTAGTAATCGTAGATCAGAATGCTACGGTGAAT
ACGTTCCCGGGCCTTGTACACACCGCCCGTCACACCATGGGAGTGGGTTGCAAAAGAA
GTAGGTAGCTTAACCTTCGGGAGGGCGCTTACCACTTTGTGATTCATGACTGGGG
pattern 28
CGCTGGCGGCAGGCCTAACACATGCAAGTCGAGCGGCAGCGGGAAGTAGTTTACTACT
TTGCCGGCGAGCGGCGGACGGGTGAGTAATGTCTGGGAAATTGCCTGATGGAGGGGGA
TAACTACTGGAAACGGTAGCTAATACCGCATGACCTCGTAAGAGCAAAGTGGGGGACCT
TCGGGCCTCACGCCATCGGATGTGCCCAGATGGGATTAGCTAGTAGGTGGGGTAATGGC
TCACCTAGGCGACGATCCCTAGCTGGTCTGAGAGGATGACCAGCCACACTGGAACTGA
GACACGGTCCAGACTCCTACGGGAGGCAGCAGTGGGGAATATTGCACAATGGGCGCAA
GCCTGATGCAGCCATGCCGCGTGTGTGAAGAAGGCCTTCGGGTTGTAAAGCACTTTCAG
CGAGGAGGAAGGCAGTCGTGTTAATAGCACGGTTGATTGACGTTACTCGCAGAAGAAG
CACCGGCTAACTCCGTGCCAGCAGCCGCGGTAATACGGAGGGTGCAAGCGTTAATCGG
AATTACTGGGCGTAAAGCGCACGCAGGCGGTTTGTTAAGTCAGATGTGAAATCCCCGCG
CTTAACGTGGGAACTGCATTTGAAACTGGCAAGCTAGAGTCTTGTAGAGGGGGGTAGA
ATTCCAGGTGTAGCGGTGAAATGCGTAGAGATCTGGAGGAATACCGGTGGCGAAGGCG
GCCCCCTGGACAAAGACTGACGCTCAGGTGCGAAAGCGTGGGGAGCAAACAGGATTA
GATACCCTGGTAGTCCACGCTGTAAACGATGTCGACTTGGAGGTTGTGCCCTTGAGGCG
TGGCTTCCGGAGCTAACGCGTTAAGTCGACCGCCTGGGGAGTACGGCCGCAAGGTTAA
AACTCAAATGAATTGACGGGGGCCCGCACAAGCGGTGGAGCATGTGGTTTAATTCGATG
CAACGCGAAGAACCTTACCTACTCTTGACATCCACGGAATTTAGCAGAGATGCTTTAGT
GCCTTCGGGAACCGTGAGACAGGTGCTGCATGGCTGTCGTCAGCTCGTGTTGTGAAATG

TTGGGTTAAGTCCCGCAACGAGCGCAACCCTTATCCTTTGTTGCCAGCACGTAATGGTG
GGAACTCAAGGGAGACTGCCGGTGACAAACCGGAGGAAGGTGGGGATGACGTCAAGT
CATCATGGCCCTTACGAGTAGGGCTACACACGTGCTACAATGGCAGATACAAAGTGAAG
CGAACTCGCGAGAGCAAGCGGACCACATAAAGTCTGTCGTAGTCCGGATTGGAGTCTG
CAACTCGACTCCATGAAGTCGGAATCGCTAGTAATCGTAGATCAGAATGCTACGGTGAAT
ACGTTCCCGGGCCTTGTACACACCGCCCGTCACACCATGGGAGTGGGTTGCAAAAGAA
GTAGGTAGCTTAACCTTCGGGAGGGCGCTTACCACTTTGTGATTCATGACTGGGG
pattern 29
CGCTGGCGGCAGGCCTAACACATGCAAGTCGAGCGGCAGCGGGAAGTAGTTTACTACT
TTGCCGGCGAGCGGCGGACGGGTGAGTAATGTCTGGGAAACTGCCTGATGGAGGGGGA
TAACTACTGGAAACGGTAGCTAATACCGCATGACCTCGCAAGAGCAAAGTGGGGGACC
TTCGGGCCTCACGCCATCGGATGTGCCCAGATGGGATTAGCTAGTAGGTGGGGTAATGG
CTCACCTAGGCGACGATCCCTAGCTGGTCTGAGAGGATGACCAGCCACACTGGAACTG
AGACACGGTCCAGACTCCTACGGGAGGCAGCAGTGGGGAATATTGCACAATGGGCGCA
AGCCTGATGCAGCCATGCCGCGTGTGTGAAGAAGGCCTTCGGGTTGTAAAGCACTTTCA
GCGAGGAGGAAGGCAGTCGTGTTAATAGCACGGTTGATTGACGTTACTCGCAGAAGAA
GCACCGGCTAACTCCGTGCCAGCAGCCGCGGTAATACGGAGGGTGCAAGCGTTAATCG
GAATTACTGGGCGTAAAGCGCACGCAGGCGGTTTGTTAAGTCAGATGTGAAATCCCCGC
GCTTAACGTGGGAACTGCATTTGAAACTGGCAAGCTAGAGTCTTGTAGAGGGGGGTAG
AATTCCAGGTGTAGCGGTGAAATGCGTAGAGATCTGGAGGAATACCGGTGGCGAAGGC
GGCCCCCTGGACAAAGACTGACGCTCAGGTGCGAAAGCGTGGGGAGCAAACAGGATT
AGATACCCTGGTAGTCCACGCTGTAAACGATGTCGACTTGGAGGTTGTGCCCTTGAGGC
GTGGCTTCCGGAGCTAACGCGTTAAGTCGACCGCCTGGGGAGTACGGCCGCAAGGTTA
AAACTCAAATGAATTGACGGGGGCCCGCACAAGCGGTGGAGCATGTGGTTTAATTCGAT
GCAACGCGAAGAACCTTACCTACTCTTGACATCCACGGAATTTAGCAGAGATGCTTTAG
TGCCTTCGGGAACCGTGAGACAGGTGCTGCATGGCTGTCGTCAGCTCGTGTTGTGAAAT
GTTGGGTTAAGTCCCGCAACGAGCGCAACCCTTATCCTTTGTTGCCAGCACGTAATGGT
GGGAACTCAAGGGAGACTGCCGGTGACAAACCGGAGGAAGGTGGGGATGACGTCAAG
TCATCATGGCCCTTACGAGTAGGGCTACACACGTGCTACAATGGCAGATACAAAGTGAA
GCGAACTCGCGAGAGCAAGCGGACCACATAAAGTCTGTCGTAGTCCGGATTGGAGTCT
GCAACTCGACTCCATGAAGTCGGAATCGCTAGTAATCGTAGATCAGAATGCTACGGTGA
ATACGTTCCCGGGCCTTGTACACACCGCCCGTCACACCATGGGAGTGGGTTGCAAAAG
AAGTAGGTAGCTTAACCTTCGGGAGGGCGCTTACCACTTTGTGATTCATGACTGGGG
pattern 30
CGCTGGCGGCAGGCCTAACACATGCAAGTCGAGCGGCAGCGGGAAGTAGTTTACTACT
TTGCCGGCGAGCGGCGGACGGGTGAGTAATGTCTGGGAAACTGCCTGATGGAGGGGGA
TAACTACTGGAAACGGTAGCTAATACCGCATGACCTCGCAAGAGCAAAGTGGGGGACC
TTCGGGCCTCACGCCATCGGATGTGCCCAGATGGGATTAGCTAGTAGGTGGGGTAATGG
CTCACCTAGGCGACGATCCCTAGCTGGTCTGAGAGGATGACCAGCCACACTGGAACTG
AGACACGGTCCAGACTCCTACGGGAGGCAGCAGTGGGGAATATTGCACAATGGGCGCA
AGCCTGATGCAGCCATGCCGCGTGTGTGAAGAAGGCCTTCGGGTTGTAAAGCACTTTCA
GCGAGGAGGAAGGCAGTCGTGTTAATAGCACGATTGATTGACGTTACTCGCAGAAGAA
GCACCGGCTAACTCCGTGCCAGCAGCCGCGGTAATACGGAGGGTGCAAGCGTTAATCG
GAATTACTGGGCGTAAAGCGCACGCAGGCGGTTTGTTAAGTCAGATGTGAAATCCCCGC

GCTTAACGTGGGAACTGCATTTGAAACTGGCAAGCTAGAGTCTTGTAGAGGGGGGTAG
AATTCCAGGTGTAGCGGTGAAATGCGTAGAGATCTGGAGGAATACCGGTGGCGAAGGC
GGCCCCCTGGACAAAGACTGACGCTCAGGTGCGAAAGCGTGGGGAGCAAACAGGATT
AGATACCCTGGTAGTCCACGCTGTAAACGATGTCGACTTGGAGGTTGTGCCCTTGAGGC
GTGGCTTCCGGAGCTAACGCGTTAAGTCGACCGCCTGGGGAGTACGGCCGCAAGGTTA
AAACTCAAATGAATTGACGGGGGCCCGCACAAGCGGTGGAGCATGTGGTTTAATTCGAT
GCAACGCGAAGAACCTTACCTACTCTTGACATCCACAGAACTTAGCAGAGATGCTTAGG
TGCCTTCGGGAACTGTGAGACAGGTGCTGCATGGCTGTCGTCAGCTCGTGTTGTGAAAT
GTTGGGTTAAGTCCCGCAACGAGCGCAACCCTTATCCTTTGTTGCCAGCACGTAATGGT
GGGAACTCAAGGGAGACTGCCGGTGACAAACCGGAGGAAGGTGGGGATGACGTCAAG
TCATCATGGCCCTTACGAGTAGGGCTACACACGTGCTACAATGGCAGATACAAAGTGAA
GCGAACTCGCGAGAGCAAGCGGACCACATAAAGTCTGTCGTAGTCCGGATTGGAGTCT
GCAACTCGACTCCATGAAGTCGGAATCGCTAGTAATCGTAGATCAGAATGCTACGGTGA
ATACGTTCCCGGGCCTTGTACACACCGCCCGTCACACCATGGGAGTGGGTTGCAAAAG
AAGTAGGTAGCTTAACCTTCGGGAGGGCGCTTACCACTTTGTGATTCATGACTGGGG
pattern 31
CGCTGGCGGCAGGCCTAACACATGCAAGTCGAGCGGCAGCGGGAAGTAGTTTACTACT
TTGCCGGCGAGCGGCGGACGGGTGAGTAATGTCTGGGAAACTGCCTGATGGAGGGGGA
TAACTACTGGAAACGGTAGCTAATACCGCATGACCTCGTAAGAGCAAAGTGGGGGACCT
TCGGGCCTCACGCCATCGGATGTGCCCAGATGGGATTAGCTAGTAGGTGGGGTAATGGC
TCACCTAGGCGACGATCCCTAGCTGGTCTGAGAGGATGACCAGCCACACTGGAACTGA
GACACGGTCCAGACTCCTACGGGAGGCAGCAGTGGGGAATATTGCACAATGGGCGCAA
GCCTGATGCAGCCATGCCGCGTGTGTGAAGAAGGCCTTCGGGTTGTAAAGCACTTTCAG
CGAGGAGGAAGGCAGTCGTGTTAATAGCACGATTGATTGACGTTACTCGCAGAAGAAG
CACCGGCTAACTCCGTGCCAGCAGCCGCGGTAATACGGAGGGTGCAAGCGTTAATCGG
AATTACTGGGCGTAAAGCGCACGCAGGCGGTTTGTTAAGTCAGATGTGAAATCCCCGCG
CTTAACGTGGGAACTGCATTTGAAACTGGCAAGCTAGAGTCTTGTAGAGGGGGGTAGA
ATTCCAGGTGTAGCGGTGAAATGCGTAGAGATCTGGAGGAATACCGGTGGCGAAGGCG
GCCCCCTGGACAAAGACTGACGCTCAGGTGCGAAAGCGTGGGGAGCAAACAGGATTA
GATACCCTGGTAGTCCACGCTGTAAACGATGTCGACTTGGAGGTTGTGCCCTTGAGGCG
TGGCTTCCGGAGCTAACGCGTTAAGTCGACCGCCTGGGGAGTACGGCCGCAAGGTTAA
AACTCAAATGAATTGACGGGGGCCCGCACAAGCGGTGGAGCATGTGGTTTAATTCGATG
CAACGCGAAGAACCTTACCTACTCTTGACATCCACAGAACTTAGCAGAGATGCTTAGGT
GCCTTCGGGAACTGTGAGACAGGTGCTGCATGGCTGTCGTCAGCTCGTGTTGTGAAATG
TTGGGTTAAGTCCCGCAACGAGCGCAACCCTTATCCTTTGTTGCCAGCACGTAATGGTG
GGAACTCAAGGGAGACTGCCGGTGACAAACCGGAGGAAGGTGGGGATGACGTCAAGT
CATCATGGCCCTTACGAGTAGGGCTACACACGTGCTACAATGGCAGATACAAAGTGAAG
CGAACTCGCGAGAGCAAGCGGACCACATAAAGTCTGTCGTAGTCCGGATTGGAGTCTG
CAACTCGACTCCATGAAGTCGGAATCGCTAGTAATCGTAGATCAGAATGCTACGGTGAAT
ACGTTCCCGGGCCTTGTACACACCGCCCGTCACACCATGGGAGTGGGTTGCAAAAGAA
GTAGGTAGCTTAACCTTCGGGAGGGCGCTTACCACTTTGTGATTCATGACTGGGG
pattern 32
CGCTGGCGGCAGGCCTAACACATGCAAGTCGAGCGGCAGCGGGAAGTAGTTTACTACT
TTGCCGGCGAGCGGCGGACGGGTGAGTAATGTCTGGGAAACTGCCTGATGGAGGGGGA

TAACTACTGGAAACGGTAGCTAATACCGCATAACGTCTTCGGACCAAAGTGGGGGACCT
TCGGGCCTCACGCCATCGGATGTGCCCAGATGGGATTAGCTAGTAGGTGGGGTAATGGC
TCACCTAGGCGACGATCCCTAGCTGGTCTGAGAGGATGACCAGCCACACTGGAACTGA
GACACGGTCCAGACTCCTACGGGAGGCAGCAGTGGGGAATATTGCACAATGGGCGCAA
GCCTGATGCAGCCATGCCGCGTGTGTGAAGAAGGCCTTCGGGTTGTAAAGCACTTTCAG
CGAGGAGGAAGGCATAAAGGTTAATAACCTTTGTGATTGACGTTACTCGCAGAAGAAG
CACCGGCTAACTCCGTGCCAGCAGCCGCGGTAATACGGAGGGTGCAAGCGTTAATCGG
AATTACTGGGCGTAAAGCGCACGCAGGCGGTTTGTTAAGTCAGATGTGAAATCCCCGCG
CTTAACGTGGGAACTGCATTTGAAACTGGCAAGCTAGAGTCTTGTAGAGGGGGGTAGA
ATTCCAGGTGTAGCGGTGAAATGCGTAGAGATCTGGAGGAATACCGGTGGCGAAGGCG
GCCCCCTGGACAAAGACTGACGCTCAGGTGCGAAAGCGTGGGGAGCAAACAGGATTA
GATACCCTGGTAGTCCACGCTGTAAACGATGTCGACTTGGAGGTTGTGCCCTTGAGGCG
TGGCTTCCGGAGCTAACGCGTTAAGTCGACCGCCTGGGGAGTACGGCCGCAAGGTTAA
AACTCAAATGAATTGACGGGGGCCCGCACAAGCGGTGGAGCATGTGGTTTAATTCGATG
CAACGCGAAGAACCTTACCTACTCTTGACATCCACGGAATTTAGCAGAGATGCTTTAGT
GCCTTCGGGAACCGTGAGACAGGTGCTGCATGGCTGTCGTCAGCTCGTGTTGTGAAATG
TTGGGTTAAGTCCCGCAACGAGCGCAACCCTTATCCTTTGTTGCCAGCACGTGATGGTG
GGAACTCAAAGGAGACTGCCGGTGATAAACCGGAGGAAGGTGGGGATGACGTCAAGT
CATCATGGCCCTTACGAGTAGGGCTACACACGTGCTACAATGGCAGATACAAAGTGAAG
CGAACTCGCGAGAGCAAGCGGACCACATAAAGTCTGTCGTAGTCCGGATTGGAGTCTG
CAACTCGACTCCATGAAGTCGGAATCGCTAGTAATCGTAGATCAGAATGCTACGGTGAAT
ACGTTCCCGGGCCTTGTACACACCGCCCGTCACACCATGGGAGTGGGTTGCAAAAGAA
GTAGGTAGCTTAACCTTCGGGAGGGCGCTTACCACTTTGTGATTCATGACTGGGG
pattern 33
CGCTGGCGGCAGGCCTAACACATGCAAGTCGAGCGGCAGCGGGAAGTAGTTTACTACT
TTGCCGGCGAGCGGCGGACGGGTGAGTAATGTCTGGGAAACTGCCTGATGGAGGGGGA
TAACTACTGGAAACGGTAGCTAATACCGCATAACGTCTTCGGACCAAAGTGGGGGACCT
TCGGGCCTCACGCCATCGGATGTGCCCAGATGGGATTAGCTAGTAGGTGGGGTAATGGC
TCACCTAGGCGACGATCCCTAGCTGGTCTGAGAGGATGACCAGCCACACTGGAACTGA
GACACGGTCCAGACTCCTACGGGAGGCAGCAGTGGGGAATATTGCACAATGGGCGCAA
GCCTGATGCAGCCATGCCGCGTGTGTGAAGAAGGCCTTCGGGTTGTAAAGCACTTTCAG
CGAGGAGGAAGGCATAAAGGTTAATAACCTTTGTGATTGACGTTACTCGCAGAAGAAG
CACCGGCTAACTCCGTGCCAGCAGCCGCGGTAATACGGAGGGTGCAAGCGTTAATCGG
AATTACTGGGCGTAAAGCGCACGCAGGCGGTTTGTTAAGTCAGATGTGAAATCCCCGCG
CTTAACGTGGGAACTGCATTTGAAACTGGCAAGCTAGAGTCTTGTAGAGGGGGGTAGA
ATTCCAGGTGTAGCGGTGAAATGCGTAGAGATCTGGAGGAATACCGGTGGCGAAGGCG
GCCCCCTGGACAAAGACTGACGCTCAGGTGCGAAAGCGTGGGGAGCAAACAGGATTA
GATACCCTGGTAGTCCACGCTGTAAACGATGTCGACTTGGAGGTTGTGCCCTTGAGGCG
TGGCTTCCGGAGCTAACGCGTTAAGTCGACCGCCTGGGGAGTACGGCCGCAAGGTTAA
AACTCAAATGAATTGACGGGGGCCCGCACAAGCGGTGGAGCATGTGGTTTAATTCGATG
CAACGCGAAGAACCTTACCTACTCTTGACATCCACAGAACTTAGCAGAGATGCTTCGGT
GCCTTCGGGAATTGTGAGACAGGTGCTGCATGGCTGTCGTCAGCTCGTGTTGTGAAATG
TTGGGTTAAGTCCCGCAACGAGCGCAACCCTTATCCTTTGTTGCCAGCACGTGATGGTG
GGAACTCAAAGGAGACTGCCGGTGATAAACCGGAGGAAGGTGGGGATGACGTCAAGT

CATCATGGCCCTTACGAGTAGGGCTACACACGTGCTACAATGGCAGATACAAAGTGAAG
CGAACTCGCGAGAGCAAGCGGACCACATAAAGTCTGTCGTAGTCCGGATTGGAGTCTG
CAACTCGACTCCATGAAGTCGGAATCGCTAGTAATCGTAGATCAGAATGCTACGGTGAAT
ACGTTCCCGGGCCTTGTACACACCGCCCGTCACACCATGGGAGTGGGTTGCAAAAGAA
GTAGGTAGCTTAACCTTCGGGAGGGCGCTTACCACTTTGTGATTCATGACTGGGG
pattern 34
CGCTGGCGGCAGGCCTAACACATGCAAGTCGAGCGGCAGCGGGGAGTAGTTTACTACT
TTGCCGGCGAGCGGCGGACGGGTGAGTAATGTCTGGGAAACTGCCTGATGGAGGGGGA
TAACTACTGGAAACGGTAGCTAATACCGCATAACGTCTTCGGACCAAAGTGGGGGACCT
TCGGGCCTCACGCCATCGGATGTGCCCAGATGGGATTAGCTAGTAGGTGGGGTAATGGC
TCACCTAGGCGACGATCCCTAGCTGGTCTGAGAGGATGACCAGCCACACTGGAACTGA
GACACGGTCCAGACTCCTACGGGAGGCAGCAGTGGGGAATATTGCACAATGGGCGCAA
GCCTGATGCAGCCATGCCGCGTGTGTGAAGAAGGCCTTCGGGTTGTAAAGCACTTTCAG
CGAGGAGGAAGGCATAAAGGTTAATAACCTTTGTGATTGACGTTACTCGCAGAAGAAG
CACCGGCTAACTCCGTGCCAGCAGCCGCGGTAATACGGAGGGTGCAAGCGTTAATCGG
AATTACTGGGCGTAAAGCGCACGCAGGCGGTTTGTTAAGTCAGATGTGAAATCCCCGCG
CTTAACGTGGGAACTGCATTTGAAACTGGCAAGCTAGAGTCTTGTAGAGGGGGGTAGA
ATTCCAGGTGTAGCGGTGAAATGCGTAGAGATCTGGAGGAATACCGGTGGCGAAGGCG
GCCCCCTGGACAAAGACTGACGCTCAGGTGCGAAAGCGTGGGGAGCAAACAGGATTA
GATACCCTGGTAGTCCACGCTGTAAACGATGTCGACTTGGAGGTTGTGCCCTTGAGGCG
TGGCTTCCGGAGCTAACGCGTTAAGTCGACCGCCTGGGGAGTACGGCCGCAAGGTTAA
AACTCAAATGAATTGACGGGGGCCCGCACAAGCGGTGGAGCATGTGGTTTAATTCGATG
CAACGCGAAGAACCTTACCTACTCTTGACATCCACGGAATTTAGCAGAGATGCTTTAGT
GCCTTCGGGAACCGTGAGACAGGTGCTGCATGGCTGTCGTCAGCTCGTGTTGTGAAATG
TTGGGTTAAGTCCCGCAACGAGCGCAACCCTTATCCTTTGTTGCCAGCACGTGATGGTG
GGAACTCAAAGGAGACTGCCGGTGATAAACCGGAGGAAGGTGGGGATGACGTCAAGT
CATCATGGCCCTTACGAGTAGGGCTACACACGTGCTACAATGGCAGATACAAAGTGAAG
CGAACTCGCGAGAGCAAGCGGACCACATAAAGTCTGTCGTAGTCCGGATTGGAGTCTG
CAACTCGACTCCATGAAGTCGGAATCGCTAGTAATCGTAGATCAGAATGCTACGGTGAAT
ACGTTCCCGGGCCTTGTACACACCGCCCGTCACACCATGGGAGTGGGTTGCAAAAGAA
GTAGGTAGCTTAACCTTCGGGAGGGCGCTTACCACTTTGTGATTCATGACTGGGG
pattern 35
CGCTGGCGGCAGGCCTAACACATGCAAGTCGAGCGGCAGCGGGAAGTAGTTTACTACT
TTGCCGGCGAGCGGCGGACGGGTGAGTAATGTCTGGGAAACTGCCTGATGGAGGGGGA
TAACTACTGGAAACGGTAGCTAATACCGCATAACGTCTTCGGACCAAAGTGGGGGACCT
TCGGGCCTCACGCCATCGGATGTGCCCAGATGGGATTAGCTAGTAGGTGGGGTAACGGC
TCACCTAGGCGACGATCCCTAGCTGGTCTGAGAGGATGACCAGCCACACTGGAACTGA
GACACGGTCCAGACTCCTACGGGAGGCAGCAGTGGGGAATATTGCACAATGGGCGCAA
GCCTGATGCAGCCATGCCGCGTGTGTGAAGAAGGCCTTCGGGTTGTAAAGCACTTTCAG
CGAGGAGGAAGGCATAAAGGTTAATAACCTTTGTGATTGACGTTACTCGCAGAAGAAG
CACCGGCTAACTCCGTGCCAGCAGCCGCGGTAATACGGAGGGTGCAAGCGTTAATCGG
AATTACTGGGCGTAAAGCGCACGCAGGCGGTTTGTTAAGTCAGATGTGAAATCCCCGCG
CTTAACGTGGGAACTGCATTTGAAACTGGCAAGCTAGAGTCTTGTAGAGGGGGGTAGA
ATTCCAGGTGTAGCGGTGAAATGCGTAGAGATCTGGAGGAATACCGGTGGCGAAGGCG

GCCCCCTGGACAAAGACTGACGCTCAGGTGCGAAAGCGTGGGGAGCAAACAGGATTA
GATACCCTGGTAGTCCACGCTGTAAACGATGTCGACTTGGAGGTTGTGCCCTTGAGGCG
TGGCTTCCGGAGCTAACGCGTTAAGTCGACCGCCTGGGGAGTACGGCCGCAAGGTTAA
AACTCAAATGAATTGACGGGGGCCCGCACAAGCGGTGGAGCATGTGGTTTAATTCGATG
CAACGCGAAGAACCTTACCTACTCTTGACATCCACGGAATTTAGCAGAGATGCTTTAGT
GCCTTCGGGAACCGTGAGACAGGTGCTGCATGGCTGTCGTCAGCTCGTGTTGTGAAATG
TTGGGTTAAGTCCCGCAACGAGCGCAACCCTTATCCTTTGTTGCCAGCACGTGATGGTG
GGAACTCAAAGGAGACTGCCGGTGATAAACCGGAGGAAGGTGGGGATGACGTCAAGT
CATCATGGCCCTTACGAGTAGGGCTACACACGTGCTACAATGGCAGATACAAAGTGAAG
CGAACTCGCGAGAGCAAGCGGACCACATAAAGTCTGTCGTAGTCCGGATTGGAGTCTG
CAACTCGACTCCATGAAGTCGGAATCGCTAGTAATCGTAGATCAGAATGCTACGGTGAAT
ACGTTCCCGGGCCTTGTACACACCGCCCGTCACACCATGGGAGTGGGTTGCAAAAGAA
GTAGGTAGCTTAACCTTCGGGAGGGCGCTTACCACTTTGTGATTCATGACTGGGG
pattern 36
CGCTGGCGGCAGGCCTAACACATGCAAGTCGAGCGGCAGCGGGAAGTAGTTTACTACT
TTGCCGGCGAGCGGCGGACGGGTGAGTAATGTCTGGGAAACTGCCTGATGGAGGGGGA
TAACTACTGGAAACGGTAGCTAATACCGCATAACGTCTTCGGACCAAAGTGGGGGACCT
TCGGGCCTCACGCCATCGGATGTGCCCAGATGGGATTAGCTAGTAGGTGGGGTAATGGC
TCACCTAGGCGACGATCCCTAGCTGGTCTGAGAGGATGACCAGCCACACTGGAACTGA
GACACGGTCCAGACTCCTACGGGAGGCAGCAGTGGGGAATATTGCACAATGGGCGCAA
GCCTGATGCAGCCATGCCGCGTGTGTGAAGAAGGCCTTCGGGTTGTAAAGCACTTTCAG
CGAGGAGGAAGGCATAAAGGTTAATAACCTTTGTGATTGACGTTACTCGCAGAAGAAG
CACCGGCTAACTCCGTGCCAGCAGCCGCGGTAATACGGAGGGTGCAAGCGTTAATCGG
AATTACTGGGCGTAAAGCGCACGCAGGCGGTTTGTTAAGTCAGATGTGAAATCCCCGCG
CTTAACGTGGGAACTGCATTTGAAACTGGCAAGCTAGAGTCTTGTAGAGGGGGGTAGA
ATTCCAGGTGTAGCGGTGAAATGCGTAGAGATCTGGAGGAATACCGGTGGCGAAGGCG
GCCCCCTGGACAAAGACTGACGCTCAGGTGCGAAAGCGTGGGGAGCAAACAGGATTA
GATACCCTGGTAGTCCACGCTGTAAACGATGTCGACTTGGAGGTTGTGCCCTTGAGGCG
TGGCTTCCGGAGCTAACGCGTTAAGTCGACCGCCTGGGGAGTACGGCCGCAAGGTTAA
AACTCAAATGAATTGACGGGGGCCCGCACAAGCGGTGGAGCATGTGGTTTAATTCGATG
CAACGCGAAGAACCTTACCTACTCTTGACATCCACGGAATTTAGCAGAGATGCTTTAGT
GCCTTCGGGAACCGTGAGACAGGTGCTGCATGGCTGTCGTCAGCTCGTGTTGTGAAATG
TTGGGTTAAGTCCCGCAACGAGCGCAACCCTTATCCTTTGTTGCCAGCACGTAATGGTG
GGAACTCAAAGGAGACTGCCGGTGATAAACCGGAGGAAGGTGGGGATGACGTCAAGT
CATCATGGCCCTTACGAGTAGGGCTACACACGTGCTACAATGGCAGATACAAAGTGAAG
CGAACTCGCGAGAGCAAGCGGACCACATAAAGTCTGTCGTAGTCCGGATTGGAGTCTG
CAACTCGACTCCATGAAGTCGGAATCGCTAGTAATCGTAGATCAGAATGCTACGGTGAAT
ACGTTCCCGGGCCTTGTACACACCGCCCGTCACACCATGGGAGTGGGTTGCAAAAGAA
GTAGGTAGCTTAACCTTCGGGAGGGCGCTTACCACTTTGTGATTCATGACTGGGG
pattern 37
CGCTGGCGGCAGGCCTAACACATGCAAGTCGAGCGGCAGCGGGAAGTAGTTTACTACT
TTGCCGGCGAGCGGCGGACGGGTGAGTAATGTCTGGGAAACTGCCTGATGGAGGGGGA
TAACTACTGGAAACGGTAGCTAATACCGCATAACGTCTTCGGACCAAAGTGGGGGACCT
TCGGGCCTCACGCCATCGGATGTGCCCAGATGGGATTAGCTAGTAGGTGGGGTAACGGC

TCACCTAGGCGACGATCCCTAGCTGGTCTGAGAGGATGACCAGCCACACTGGAACTGA
GACACGGTCCAGACTCCTACGGGAGGCAGCAGTGGGGAATATTGCACAATGGGCGCAA
GCCTGATGCAGCCATGCCGCGTGTGTGAAGAAGGCCTTCGGGTTGTAAAGCACTTTCAG
CGAGGAGGAAGGCATAAAGGTTAATAACCTTTGTGATTGACGTTACTCGCAGAAGAAG
CACCGGCTAACTCCGTGCCAGCAGCCGCGGTAATACGGAGGGTGCAAGCGTTAATCGG
AATTACTGGGCGTAAAGCGCACGCAGGCGGTTTGTTAAGTCAGATGTGAAATCCCCGCG
CTTAACGTGGGAACTGCATTTGAAACTGGCAAGCTAGAGTCTTGTAGAGGGGGGTAGA
ATTCCAGGTGTAGCGGTGAAATGCGTAGAGATCTGGAGGAATACCGGTGGCGAAGGCG
GCCCCCTGGACAAAGACTGACGCTCAGGTGCGAAAGCGTGGGGAGCAAACAGGATTA
GATACCCTGGTAGTCCACGCTGTAAACGATGTCGACTTGGAGGTTGTGCCCTTGAGGCG
TGGCTTCCGGAGCTAACGCGTTAAGTCGACCGCCTGGGGAGTACGGCCGCAAGGTTAA
AACTCAAATGAATTGACGGGGGCCCGCACAAGCGGTGGAGCATGTGGTTTAATTCGATG
CAACGCGAAGAACCTTACCTACTCTTGACATCCACGGAATTTAGCAGAGATGCTTTAGT
GCCTTCGGGAACCGTGAGACAGGTGCTGCATGGCTGTCGTCAGCTCGTGTTGTGAAATG
TTGGGTTAAGTCCCGCAACGAGCGCAACCCTTATCCTTTGTTGCCAGCACGTAATGGTG
GGAACTCAAAGGAGACTGCCGGTGATAAACCGGAGGAAGGTGGGGATGACGTCAAGT
CATCATGGCCCTTACGAGTAGGGCTACACACGTGCTACAATGGCAGATACAAAGTGAAG
CGAACTCGCGAGAGCAAGCGGACCACATAAAGTCTGTCGTAGTCCGGATTGGAGTCTG
CAACTCGACTCCATGAAGTCGGAATCGCTAGTAATCGTAGATCAGAATGCTACGGTGAAT
ACGTTCCCGGGCCTTGTACACACCGCCCGTCACACCATGGGAGTGGGTTGCAAAAGAA
GTAGGTAGCTTAACCTTCGGGAGGGCGCTTACCACTTTGTGATTCATGACTGGGG

pattern 38

CGCTGGCGGCAGGCCTAACACATGCAAGTCGAGCGGCAGCGGGAAGTAGTTTACTACT
TTGCCGGCGAGCGGCGGACGGGTGAGTAATGTCTGGGAAACTGCCTGATGGAGGGGGA
TAACTACTGGAAACGGTAGCTAATACCGCATAACGTCTTCGGACCAAAGTGGGGGACCT
TCGGGCCTCACGCCATCGGATGTGCCCAGATGGGATTAGCTAGTAGGTGGGGTAATGGC
TCACCTAGGCGACGATCCCTAGCTGGTCTGAGAGGATGACCAGCCACACTGGAACTGA
GACACGGTCCAGACTCCTACGGGAGGCAGCAGTGGGGAATATTGCACAATGGGCGCAA
GCCTGATGCAGCCATGCCGCGTGTGTGAAGAAGGCCTTCGGGTTGTAAAGCACTTTCAG
CGAGGAGGAAGGCATAAAGGTTAATAACCTTTGTGATTGACGTTACTCGCAGAAGAAG
CACCGGCTAACTCCGTGCCAGCAGCCGCGGTAATACGGAGGGTGCAAGCGTTAATCGG
AATTACTGGGCGTAAAGCGCACGCAGGCGGTTTGTTAAGTCAGATGTGAAATCCCCGCG
CTTAACGTGGGAACTGCATTTGAAACTGGCAAGCTAGAGTCTTGTAGAGGGGGGTAGA
ATTCCAGGTGTAGCGGTGAAATGCGTAGAGATCTGGAGGAATACCGGTGGCGAAGGCG
GCCCCCTGGACAAAGACTGACGCTCAGGTGCGAAAGCGTGGGGAGCAAACAGGATTA
GATACCCTGGTAGTCCACGCTGTAAACGATGTCGACTTGGAGGTTGTGCCCTTGAGGCG
TGGCTTCCGGAGCTAACGCGTTAAGTCGACCGCCTGGGGAGTACGGCCGCAAGGTTAA
AACTCAAATGAATTGACGGGGGCCCGCACAAGCGGTGGAGCATGTGGTTTAATTCGATG
CAACGCGAAGAACCTTACCTACTCTTGACATCCACGGAATTTAGCAGAGATGCTTTAGT
GCCTTCGGGAACCGTGAGACAGGTGCTGCATGGCTGTCGTCAGCTCGTGTTGTGAAATG
TTGGGTTAAGTCCCGCAACGAGCGCAACCCTTATCCTTTGTTGCCAGCACGTAATGGTG
GGAACTCAAAGGAGACTGCCGGTGATAAACCGGAGGAAGGTGGGGATGACGTCAAGT
CATCATGGCCCTTACGAGTAGGGCTACACACGTGCTACAATGGCAGATACAAAGTGAAG
CGAACTCGCGAGAGCAAGCGGACCACATAAAGTCTGTCGTAGTTCGGATTGGAGTCTG

CAACTCGACTCCATGAAGTCGGAATCGCTAGTAATCGTAGATCAGAATGCTACGGTGAAT
ACGTTCCCGGGCCTTGTACACACCGCCCGTCACACCATGGGAGTGGGTTGCAAAAGAA
GTAGGTAGCTTAACCTTCGGGAGGGCGCTTACCACTTTGTGATTCATGACTGGGG

pattern 39

CGCTGGCGGCAGGCCTAACACATGCAAGTCGAGCGGCAGCGGGAAGTAGTTTACTACT
TTGCCGGCGAGCGGCGGACGGGTGAGTAATGTCTGGGAAACTGCCTGATGGAGGGGGA
TAACTACTGGAAACGGTAGCTAATACCGCATAACGTCTTCGGACCAAAGTGGGGGACCT
TCGGGCCTCACGCCATCGGATGTGCCCAGATGGGATTAGCTAGTAGGTGGGGTAATGGC
TCACCTAGGCGACGATCCCTAGCTGGTCTGAGAGGATGACCAGCCACACTGGAACTGA
GACACGGTCCAGACTCCTACGGGAGGCAGCAGTGGGGAATATTGCACAATGGGCGCAA
GCCTGATGCAGCCATGCCGCGTGTGTGAAGAAGGCCTTCGGGTTGTAAAGCACTTTCAG
CGAGGAGGAAGGCATAAAGGTTAATAACCTTTGTGATTGACGTTACTCGCAGAAGAAG
CACCGGCTAACTCCGTGCCAGCAGCCGCGGTAATACGGAGGGTGCAAGCGTTAATCGG
AATTACTGGGCGTAAAGCGCACGCAGGCGGTTTGTTAAGTCAGATGTGAAATCCCCGCG
CTTAACGTGGGAACTGCATTTGAAACTGGCAAGCTAGAGTCTTGTAGAGGGGGGTAGA
ATTCCAGGTGTAGCGGTGAAATGCGTAGAGATCTGGAGGAATACCGGTGGCGAAGGCG
GCCCCCTGGACAAAGACTGACGCTCAGGTGCGAAAGCGTGGGGAGCAAACAGGATTA
GATACCCTGGTAGTCCACGCTGTAAACGATGTCGACTTGGAGGTTGTGCCCTTGAGGCG
TGGCTTCCGGAGCTAACGCGTTAAGTCGACCGCCTGGGGAGTACGGCCGCAAGGTTAA
AACTCAAATGAATTGACGGGGGCCCGCACAAGCGGTGGAGCATGTGGTTTAATTCGATG
CAACGCGAAGAACCTTACCTACTCTTGACATCCACAGAACTTAGCAGAGATGCTTCGGT
GCCTTCGGGAACTGTGAGACAGGTGCTGCATGGCTGTCGTCAGCTCGTGTTGTGAAATG
TTGGGTTAAGTCCCGCAACGAGCGCAACCCTTATCCTTTGTTGCCAGCACGTGATGGTG
GGAACTCAAAGGAGACTGCCGGTGATAAACCGGAGGAAGGTGGGGATGACGTCAAGT
CATCATGGCCCTTACGAGTAGGGCTACACACGTGCTACAATGGCAGATACAAAGTGAAG
CGAACTCGCGAGAGCAAGCGGACCACATAAAGTCTGTCGTAGTCCGGATTGGAGTCTG
CAACTCGACTCCATGAAGTCGGAATCGCTAGTAATCGTAGATCAGAATGCTACGGTGAAT
ACGTTCCCGGGCCTTGTACACACCGCCCGTCACACCATGGGAGTGGGTTGCAAAAGAA
GTAGGTAGCTTAACCTTCGGGAGGGCGCTTACCACTTTGTGATTCATGACTGGGG

pattern 40

CGCTGGCGGCAGGCCTAACACATGCAAGTCGAGCGGCAGCGGGAAGTAGTTTACTACT
TTGCCGGCGAGCGGCGGACGGGTGAGTAATGTCTGGGAAACTGCCTGATGGAGGGGGA
TAACTACTGGAAACGGTAGCTAATACCGCATAACGTCTTCGGACCAAAGTGGGGGACCT
TCGGGCCTCACGCCATCGGATGTGCCCAGATGGGATTAGCTAGTAGGTGGGGTAACGGC
TCACCTAGGCGACGATCCCTAGCTGGTCTGAGAGGATGACCAGCCACACTGGAACTGA
GACACGGTCCAGACTCCTACGGGAGGCAGCAGTGGGGAATATTGCACAATGGGCGCAA
GCCTGATGCAGCCATGCCGCGTGTGTGAAGAAGGCCTTCGGGTTGTAAAGCACTTTCAG
CGAGGAGGAAGGCATAAAGGTTAATAACCTTTGTGATTGACGTTACTCGCAGAAGAAG
CACCGGCTAACTCCGTGCCAGCAGCCGCGGTAATACGGAGGGTGCAAGCGTTAATCGG
AATTACTGGGCGTAAAGCGCACGCAGGCGGTTTGTTAAGTCAGATGTGAAATCCCCGCG
CTTAACGTGGGAACTGCATTTGAAACTGGCAAGCTAGAGTCTTGTAGAGGGGGGTAGA
ATTCCAGGTGTAGCGGTGAAATGCGTAGAGATCTGGAGGAATACCGGTGGCGAAGGCG
GCCCCCTGGACAAAGACTGACGCTCAGGTGCGAAAGCGTGGGGAGCAAACAGGATTA
GATACCCTGGTAGTCCACGCTGTAAACGATGTCGACTTGGAGGTTGTGCCCTTGAGGCG

TGGCTTCCGGAGCTAACGCGTTAAGTCGACCGCCTGGGGAGTACGGCCGCAAGGTTAA
AACTCAAATGAATTGACGGGGGCCCGCACAAGCGGTGGAGCATGTGGTTTAATTCGATG
CAACGCGAAGAACCTTACCTACTCTTGACATCCACGGAATTTAGCAGAGATGCTTTAGT
GCCTTCGGGAACCGTGAGACAGGTGCTGCATGGCCGTCGTCAGCTCGTGTTGTGAAAT
GTTGGGTTAAGTCCCGCAACGAGCGCAACCCTTATCCTTTGTTGCCAGCACGTGATGGT
GGGAACTCAAAGGAGACTGCCGGTGATAAACCGGAGGAAGGTGGGGATGACGTCAAG
TCATCATGGCCCTTACGAGTAGGGCTACACACGTGCTACAATGGCAGATACAAAGTGAA
GCGAACTCGCGAGAGCAAGCGGACCACATAAAGTCTGTCGTAGTCCGGATTGGAGTCT
GCAACTCGACTCCATGAAGTCGGAATCGCTAGTAATCGTAGATCAGAATGCTACGGTGA
ATACGTTCCCGGGCCTTGTACACACCGCCCGTCACACCATGGGAGTGGGTTGCAAAAG
AAGTAGGTAGCTTAACCTTCGGGAGGGCGCTTACCACTTTGTGATTCATGACTGGGG
pattern 41
CGCTGGCGGCAGGCCTAACACATGCAAGTCGAGCGGCAGCGGGAAGTAGTTTACTACT
TTGCCGGCGAGCGGCGGACGGGTGAGTAATGTCTGGGAAACTGCCTGATGGAGGGGGA
TAACTACTGGAAACGGTAGCTAATACCGCATAACGTCTTCGGACCAAAGTGGGGGACCT
TCGGGCCTCACGCCATCGGATGTGCCCAGATGGGATTAGCTAGTAGGTGGGGTAATGGC
TCACCTAGGCGACGATCCCTAGCTGGTCTGAGAGGATGACCAGCCACACTGGAACTGA
GACACGGTCCAGACTCCTACGGGAGGCAGCAGTGGGGAATATTGCACAATGGGCGCAA
GCCTGATGCAGCCATGCCGCGTGTGTGAAGAAGGCCTTCGGGTTGTAAAGCACTTTCAG
CGAGGAGGAAGGCATAAAGGTTAATAACCTTTGTGATTGACGTTACTCGCAGAAGAAG
CACCGGCTAACTCCGTGCCAGCAGCCGCGGTAATACGGAGGGTGCAAGCGTTAATCGG
AATTACTGGGCGTAAAGCGCACGCAGGCGGTTTGTTAAGTCAGATGTGAAATCCCCGCG
CTTAACGTGGGAACTGCATTTGAAACTGGCAAGCTAGAGTCTTGTAGAGGGGGGTAGA
ATTCCAGGTGTAGCGGTGAAATGCGTAGAGATCTGGAGGAATACCGGTGGCGAAGGCG
GCCCCCTGGACAAAGACTGACGCTCAGGTGCGAAAGCGTGGGGAGCAAACAGGATTA
GATACCCTGGTAGTCCACGCTGTAAACGATGTCGACTTGGAGGTTGTGCCCTTGAGGCG
TGGCTTCCGGAGCTAACGCGTTAAGTCGACCGCCTGGGGAGTACGGCCGCAAGGTTAA
AACTCAAATGAATTGACGGGGGCCCGCACAAGCGGTGGAGCATGTGGTTTAATTCGATG
CAACGCGAAGAACCTTACCTACTCTTGACATCCACGGAATTTAGCAGAGATGCTTTAGT
GCCTTCGGGAACCGTGAGACAGGTGCTGCATGGCCGTCGTCAGCTCGTGTTGTGAAAT
GTTGGGTTAAGTCCCGCAACGAGCGCAACCCTTATCCTTTGTTGCCAGCACGTGATGGT
GGGAACTCAAAGGAGACTGCCGGTGATAAACCGGAGGAAGGTGGGGATGACGTCAAG
TCATCATGGCCCTTACGAGTAGGGCTACACACGTGCTACAATGGCAGATACAAAGTGAA
GCGAACTCGCGAGAGCAAGCGGACCACATAAAGTCTGTCGTAGTCCGGATTGGAGTCT
GCAACTCGACTCCATGAAGTCGGAATCGCTAGTAATCGTAGATCAGAATGCTACGGTGA
ATACGTTCCCGGGCCTTGTACACACCGCCCGTCACACCATGGGAGTGGGTTGCAAAAG
AAGTAGGTAGCTTAACCTTCGGGAGGGCGCTTACCACTTTGTGATTCATGACTGGGG
pattern 42
CGCTGGCGGCAGGCCTAACACATGCAAGTCGAGCGGCAGCGGGAAGTAGTTTACTACT
TTGCCGGCGAGCGGCGGACGGGTGAGTAATGTCTGGGAAACTGCCTGATGGAGGGGGA
TAACTACTGGAAACGGTAGCTAATACCGCATAACGTCTTCGGACCAAAGTGGGGGACCT
TCGGGCCTCACGCCATCGGATGTGCCCAGATGGGATTAGCTAGTAGGTGGGGTAATGGC
TCACCTAGGCGACGATCCCTAGCTGGTCTGAGAGGATGACCAGCCACACTGGAACTGA
GACACGGTCCAGACTCCTACGGGAGGCAGCAGTGGGGAATATTGCACAATGGGCGCAA

GCCTGATGCAGCCATGCCGCGTGTGTGAAGAAGGCCTTCGGGTTGTAAAGCACTTTCAG
CGAGGAGGAAGGCATAAAGGTTAATAACCTTTGTGATTGACGTTACTCGCAGAAGAAG
CACCGGCTAACTCCGTGCCAGCAGCCGCGGTAATACGGAGGGTGCAAGCGTTAATCGG
AATTACTGGGCGTAAAGCGCACGCAGGCGGTTTGTTAAGTCAGATGTGAAATCCCCGCG
CTTAACGTGGGAACTGCATTTGAAACTGGCAAGCTAGAGTCTTGTAGAGGGGGGTAGA
ATTCCAGGTGTAGCGGTGAAATGCGTAGAGATCTGGAGGAATACCGGTGGCGAAGGCG
GCCCCCTGGACAAAGACTGACGCTCAGGTGCGAAAGCGTGGGGAGCAAACAGGATTA
GATACCCTGGTAGTCCACGCTGTAAACGATGTCGACTTGGAGGTTGTGCCCTTGAGGCG
TGGCTTCCGGAGCTAACGCGTTAAGTCGACCGCCTGGGGAGTACGGCCGCAAGGTTAA
AACTCAAATGAATTGACGGGGGCCCGCACAAGCGGTGGAGCATGTGGTTTAATTCGATG
CAACGCGAAGAACCTTACCTACTCTTGACATCCACGGAATTTAGCAGAGATGCTTTAGT
GCCTTCGGGAACCGTGAGACAGGTGCTGCATGGCTGTCGTCAGCTCGTGTTGTGAAATG
TTGGGTTAAGTCCCGCAACGAGCGCAACCCTTATCCTTTGTTGCCAGCACGTGATGGTG
GGAACTCAAAGGAGACTGCTGGTGATAAACCGGAGGAAGGTGGGGATGACGTCAAGT
CATCATGGCCCTTACGAGTAGGGCTACACACGTGCTACAATGGCAGATACAAAGTGAAG
CGAACTCGCGAGAGCAAGCGGACCACATAAAGTCTGTCGTAGTCCGGATTGGAGTCTG
CAACTCGACTCCATGAAGTCGGAATCGCTAGTAATCGTAGATCAGAATGCTACGGTGAAT
ACGTTCCCGGGCCTTGTACACACCGCCCGTCACACCATGGGAGTGGGTTGCAAAAGAA
GTAGGTAGCTTAACCTTCGGGAGGGCGCTTACCACTTTGTGATTCATGACTGGGG
pattern 43
CGCTGGCGGCAGGCCTAACACATGCAAGTCGAGCGGCAGCGGGAAGTAGTTTACTACT
TTGCCGGCGAGCGGCGGACGGGTGAGTAATGTCTGGGAAACTGCCTGATGGAGGGGGA
TAACTACTGGAAACGGTAGCTAATACCGCATAACGTCTTCGGACCAAAGTGGGGGACCT
TCGGGCCTCACGCCATCGGATGTGCCCAGATGGGATTAGCTAGTAGGTGGGGTAATGGC
TCACCTAGGCGACGATCCCTAGCTGGCCTGAGAGGATGACCAGCCACACTGGAACTGA
GACACGGTCCAGACTCCTACGGGAGGCAGCAGTGGGGAATATTGCACAATGGGCGCAA
GCCTGATGCAGCCATGCCGCGTGTGTGAAGAAGGCCTTCGGGTTGTAAAGCACTTTCAG
CGAGGAGGAAGGCATAAAGGTTAATAACCTTTGTGATTGACGTTACTCGCAGAAGAAG
CACCGGCTAACTCCGTGCCAGCAGCCGCGGTAATACGGAGGGTGCAAGCGTTAATCGG
AATTACTGGGCGTAAAGCGCACGCAGGCGGTTTGTTAAGTCAGATGTGAAATCCCCGCG
CTTAACGTGGGAACTGCATTTGAAACTGGCAAGCTAGAGTCTTGTAGAGGGGGGTAGA
ATTCCAGGTGTAGCGGTGAAATGCGTAGAGATCTGGAGGAATACCGGTGGCGAAGGCG
GCCCCCTGGACAAAGACTGACGCTCAGGTGCGAAAGCGTGGGGAGCAAACAGGATTA
GATACCCTGGTAGTCCACGCTGTAAACGATGTCGACTTGGAGGTTGTGCCCTTGAGGCG
TGGCTTCCGGAGCTAACGCGTTAAGTCGACCGCCTGGGGAGTACGGCCGCAAGGTTAA
AACTCAAATGAATTGACGGGGGCCCGCACAAGCGGTGGAGCATGTGGTTTAATTCGATG
CAACGCGAAGAACCTTACCTACTCTTGACATCCACGGAATTTAGCAGAGATGCTTTAGT
GCCTTCGGGAACCGTGAGACAGGTGCTGCATGGCTGTCGTCAGCTCGTGTTGTGAAATG
TTGGGTTAAGTCCCGCAACGAGCGCAACCCTTATCCTTTGTTGCCAGCACGTGATGGTG
GGAACTCAAAGGAGACTGCCGGTGATAAACCGGAGGAAGGTGGGGATGACGTCAAGT
CATCATGGCCCTTACGAGTAGGGCTACACACGTGCTACAATGGCAGATACAAAGTGAAG
CGAACTCGCGAGAGCAAGCGGACCACATAAAGTCTGTCGTAGTCCGGATTGGAGTCTG
CAACTCGACTCCATGAAGTCGGAATCGCTAGTAATCGTAGATCAGAATGCTACGGTGAAT
ACGTTCCCGGGCCTTGTACACACCGCCCGTCACACCATGGGAGTGGGTTGCAAAAGAA

GTAGGTAGCTTAACCTTCGGGAGGGCGCTTACCACTTTGTGATTCATGACTGGGG
pattern 44
CGCTGGCGGCAGGCCTAACACATGCAAGTCGAGCGGCAGCGGGGAGTAGTTTACTACT
TTGCCGGCGAGCGGCGGACGGGTGAGTAATGTCTGGGAAACTGCCTGATGGAGGGGGA
TAACTACTGGAAACGGTAGCTAATACCGCATAACGTCTTCGGACCAAAGTGGGGGACCT
TCGGGCCTCACGCCATCGGATGTGCCCAGATGGGATTAGCTAGTAGGTGGGGTAATGGC
TCACCTAGGCGACGATCCCTAGCTGGTCTGAGAGGATGACCAGCCACACTGGAACTGA
GACACGGTCCAGACTCCTACGGGAGGCAGCAGTGGGGAATATTGCACAATGGGCGCAA
GCCTGATGCAGCCATGCCGCGTGTGTGAAGAAGGCCTTCGGGTTGTAAAGCACTTTCAG
CGAGGAGGAAGGCATAAAGGTTAATAACCTTTGTGATTGACGTTACTCGCAGAAGAAG
CACCGGCTAACTCCGTGCCAGCAGCCGCGGTAATACGGAGGGTGCAAGCGTTAATCGG
AATTACTGGGCGTAAAGCGCACGCAGGCGGTTTGTTAAGTCAGATGTGAAATCCCCGCG
CTTAACGTGGGAACTGCATTTGAAACTGGCAAGCTAGAGTCTTGTAGAGGGGGGTAGA
ATTCCAGGTGTAGCGGTGAAATGCGTAGAGATCTGGAGGAATACCGGTGGCGAAGGCG
GCCCCCTGGACAAAGACTGACGCTCAGGTGCGAAAGCGTGGGGAGCAAACAGGATTA
GATACCCTGGTAGTCCACGCTGTAAACGATGTCGACTTGGAGGTTGTGCCCTTGAGGCG
TGGCTTCCGGAGCTAACGCGTTAAGTCGACCGCCTGGGGAGTACGGCCGCAAGGTTAA
AACTCAAATGAATTGACGGGGGCCCGCACAAGCGGTGGAGCATGTGGTTTAATTCGATG
CAACGCGAAGAACCTTACCTACTCTTGACATCCACGGAATTTAGCAGAGATGCTTTAGT
GCCTTCGGGAACCGTGAGACAGGTGCTGCATGGCTGTCGTCAGCTCGTGTTGTGAAATG
TTGGGTTAAGTCCCGCAACGAGCGCAACCCTTATCCTTTGTTGCCAGCACGTGATGGTG
GGAACTCAAAGGAGACTGCTGGTGATAAACCGGAGGAAGGTGGGGATGACGTCAAGT
CATCATGGCCCTTACGAGTAGGGCTACACACGTGCTACAATGGCAGATACAAAGTGAAG
CGAACTCGCGAGAGCAAGCGGACCACATAAAGTCTGTCGTAGTCCGGATTGGAGTCTG
CAACTCGACTCCATGAAGTCGGAATCGCTAGTAATCGTAGATCAGAATGCTACGGTGAAT
ACGTTCCCGGGCCTTGTACACACCGCCCGTCACACCATGGGAGTGGGTTGCAAAAGAA
GTAGGTAGCTTAACCTTCGGGAGGGCGCTTACCACTTTGTGATTCATGACTGGGG
pattern 45
CGCTGGCGGCAGGCCTAACACATGCAAGTCGAGCGGCAGCGGGGAGTAGTTTACTACT
TTGCCGGCGAGCGGCGGACGGGTGAGTAATGTCTGGGAAACTGCCTGATGGAGGGGGA
TAACTACTGGAAACGGTAGCTAATACCGCATAACGTCTTCGGACCAAAGTGGGGGACCT
TCGGGCCTCACGCCATCGGATGTGCCCAGATGGGATTAGCTAGTAGGTGGGGTAATGGC
TCACCTAGGCGACGATCCCTAGCTGGTCTGAGAGGATGACCAGCCACACTGGAACTGA
GACACGGTCCAGACTCCTACGGGAGGCAGCAGTGGGGGATATTGCACAATGGGCGCAA
GCCTGATGCAGCCATGCCGCGTGTGTGAAGAAGGCCTTCGGGTTGTAAAGCACTTTCAG
CGAGGAGGAAGGCATAAAGGTTAATAACCTTTGTGATTGACGTTACTCGCAGAAGAAG
CACCGGCTAACTCCGTGCCAGCAGCCGCGGTAATACGGAGGGTGCAAGCGTTAATCGG
AATTACTGGGCGTAAAGCGCACGCAGGCGGTTTGTTAAGTCAGATGTGAAATCCCCGCG
CTTAACGTGGGAACTGCATTTGAAACTGGCAAGCTAGAGTCTTGTAGAGGGGGGTAGA
ATTCCAGGTGTAGCGGTGAAATGCGTAGAGATCTGGAGGAATACCGGTGGCGAAGGCG
GCCCCCTGGACAAAGACTGACGCTCAGGTGCGAAAGCGTGGGGAGCAAACAGGATTA
GATACCCTGGTAGTCCACGCTGTAAACGATGTCGACTTGGAGGTTGTGCCCTTGAGGCG
TGGCTTCCGGAGCTAACGCGTTAAGTCGACCGCCTGGGGAGTACGGCCGCAAGGTTAA
AACTCAAATGAATTGACGGGGGCCCGCACAAGCGGTGGAGCATGTGGTTTAATTCGATG

CAACGCGAAGAACCTTACCTACTCTTGACATCCACGGAATTTAGCAGAGATGCTTTAGT
GCCTTCGGGAACCGTGAGACAGGTGCTGCATGGCTGTCGTCAGCTCGTGTTGTGAAATG
TTGGGTTAAGTCCCGCAACGAGCGCAACCCTTATCCTTTGTTGCCAGCACGTGATGGTG
GGAACTCAAAGGAGACTGCCGGTGATAAACCGGAGGAAGGTGGGGATGACGTCAAGT
CATCATGGCCCTTACGAGTAGGGCTACACACGTGCTACAATGGCAGATACAAAGTGAAG
CGAACTCGCGAGAGCAAGCGGACCACATAAAGTCTGTCGTAGTCCGGATTGGAGTCTG
CAACTCGACTCCATGAAGTCGGAATCGCTAGTAATCGTAGATCAGAATGCTACGGTGAAT
ACGTTCCCGGGCCTTGTACACACCGCCCGTCACACCATGGGAGTGGGTTGCAAAAGAA
GTAGGTAGCTTAACCTTCGGGAGGGCGCTTACCACTTTGTGATTCATGACTGGGG
pattern 46
CGCTGGCGGCAGGCCTAACACATGCAAGTCGAGCGGCAGCGGGAAGTAGTTTACTACT
TTGCCGGCGAGCGGCGGACGGGTGAGTAATGTCTGGGAAACTGCCTGATGGAGGGGGA
TAACTACTGGAAACGGTAGCTAATACCGCATAACGTCTTCGGACCAAAGTGGGGGACCT
TCGGGCCTCACGCCATCGGATGTGCCCAGATGGGATTAGCTAGTAGGTGGGGTAATGGC
TCACCTAGGCGACGATCCCTAGCTGGTCTGAGAGGATGACCAGCCACACTGGAACTGA
GACACGGTCCAGACTCCTACGGGAGGCAGCAGTGGGGAATATTGCACAATGGGCGCAA
GCCTGATGCAGCCATGCCGCGTGTGTGAAGAAGGCCTTCGGGTTGTAAAGCACTTTCAG
CGAGGAGGAAGGCCAATAACTTAATACGTTGTTGGATTGACGTTACTCGCAGAAGAAGC
ACCGGCTAACTCCGTGCCAGCAGCCGCGGTAATACGGAGGGTGCAAGCGTTAATCGGA
ATTACTGGGCGTAAAGCGCACGCAGGCGGTTTGTTAAGTCAGATGTGAAATCCCCGCGC
TTAACGTGGGAACTGCATTTGAAACTGGCAAGCTAGAGTCTTGTAGAGGGGGGTAGAA
TTCCAGGTGTAGCGGTGAAATGCGTAGAGATCTGGAGGAATACCGGTGGCGAAGGCGA
CCCCCTGGACAAAGACTGACGCTCAGGTGCGAAAGCGTGGGGAGCAAACAGGATTAG
ATACCCTGGTAGTCCACGCTGTAAACGATGTCGACTTGGAGGTTGTGCCCTTGAGGCGT
GGCTTCCGGAGCTAACGCGTTAAGTCGACCGCCTGGGGAGTACGGCCGCAAGGTTAAA
ACTCAAATGAATTGACGGGGGCCCGCACAAGCGGTGGAGCATGTGGTTTAATTCGATGC
AACGCGAAGAACCTTACCTACTCTTGACATCCACGGAATTTAGCAGAGATGCTTTAGTG
CCTTCGGGAACCGTGAGACAGGTGCTGCATGGCTGTCGTCAGCTCGTGTTGTGAAATGT
TGGGTTAAGTCCCGCAACGAGCGCAACCCTTATCCTTTGTTGCCAGCACGTAATGGTGG
GAACTCAAAGGAGACTGCCGGTGATAAACCGGAGGAAGGTGGGGATGACGTCAAGTC
ATCATGGCCCTTACGAGTAGGGCTACACACGTGCTACAATGGCAGATACAAAGTGAAGC
GAACTCGCGAGAGCAAGCGGACCACATAAAGTCTGTCGTAGTCCGGATTGGAGTCTGC
AACTCGACTCCATGAAGTCGGAATCGCTAGTAATCGTAGATCAGAATGCTACGGTGAAT
ACGTTCCCGGGCCTTGTACACACCGCCCGTCACACCATGGGAGTGGGTTGCAAAAGAA
GTAGGTAGCTTAACCTTCGGGAGGGCGCTTACCACTTTGTGATTCATGACTGGGG
pattern 47
CGCTGGCGGCAGGCCTAACACATGCAAGTCGAGCGGCAGCGGGAAGTAGTTTACTACT
TTGCCGGCGAGCGGCGGACGGGTGAGTAATGTCTGGGAAACTGCCTGATGGAGGGGGA
TAACTACTGGAAACGGTAGCTAATACCGCATAATGTCTTCGGACCAAAGTGGGGGACCT
TCGGGCCTCACGCCATCGGATGTGCCCAGATGGGATTAGCTAGTAGGTGGGGTAATGGC
TCACCTAGGCGACGATCCCTAGCTGGTCTGAGAGGATGACCAGCCACACTGGAACTGA
GACACGGTCCAGACTCCTACGGGAGGCAGCAGTGGGGAATATTGCACAATGGGCGCAA
GCCTGATGCAGCCATGCCGCGTGTGTGAAGAAGGCCTTCGGGTTGTAAAGCACTTTCAG
CGAGGAGGAAGGCCAATAACTTAATACGTTGTTGGATTGACGTTACTCGCAGAAGAAGC

ACCGGCTAACTCCGTGCCAGCAGCCGCGGTAATACGGAGGGTGCAAGCGTTAATCGGA
ATTACTGGGCGTAAAGCGCACGCAGGCGGTTTGTTAAGTCAGATGTGAAATCCCCGCGC
TTAACGTGGGAACTGCATTTGAAACTGGCAAGCTAGAGTCTTGTAGAGGGGGGTAGAA
TTCCAGGTGTAGCGGTGAAATGCGTAGAGATCTGGAGGAATACCGGTGGCGAAGGCGG
CCCCCTGGACAAAGACTGACGCTCAGGTGCGAAAGCGTGGGGAGCAAACAGGATTAG
ATACCCTGGTAGTCCACGCTGTAAACGATGTCGACTTGGAGGTTGTGCCCTTGAGGCGT
GGCTTCCGGAGCTAACGCGTTAAGTCGACCGCCTGGGGAGTACGGCCGCAAGGTTAAA
ACTCAAATGAATTGACGGGGGCCCGCACAAGCGGTGGAGCATGTGGTTTAATTCGATGC
AACGCGAAGAACCTTACCTACTCTTGACATCCACGGAATTTAGCAGAGATGCTTTAGTG
CCTTCGGGAACCGTGAGACAGGTGCTGCATGGCTGTCGTCAGCTCGTGTTGTGAAATGT
TGGGTTAAGTCCCGCAACGAGCGCAACCCTTATCCTTTGTTGCCAGCACGTAATGGTGG
GAACTCAAAGGAGACTGCCGGTGATAAACCGGAGGAAGGTGGGGATGACGTCAAGTC
ATCATGGCCCTTACGAGTAGGGCTACACACGTGCTACAATGGCAGATACAAAGTGAAGC
GAACTCGCGAGAGCAAGCGGACCACATAAAGTCTGTCGTAGTCCGGATTGGAGTCTGC
AACTCGACTCCATGAAGTCGGAATCGCTAGTAATCGTAGATCAGAATGCTACGGTGAAT
ACGTTCCCGGGCCTTGTACACACCGCCCGTCACACCATGGGAGTGGGTTGCAAAAGAA
GTAGGTAGCTTAACCTTCGGGAGGGCGCTTACCACTTTGTGATTCATGACTGGGG
pattern 48
CGCTGGCGGCAGGCCTAACACATGCAAGTCGAGCGGCAGCGGGAAGTAGTTTACTACT
TTGCCGGCGAGCGGCGGACGGGTGAGTAATGTCTGGGAAACTGCCTGATGGAGGGGGA
TAACTACTGGAAACGGTAGCTAATACCGCATAACGTCTTCGGACCAAAGTGGGGGACCT
TAGGGCCTCACGCCATCGGATGTGCCCAGATGGGATTAGCTAGTAGGTGGGGTAATGGC
TCACCTAGGCGACGATCCCTAGCTGGTCTGAGAGGATGACCAGCCACACTGGAACTGA
GACACGGTCCAGACTCCTACGGGAGGCAGCAGTGGGGAATATTGCACAATGGGCGCAA
GCCTGATGCAGCCATGCCGCGTGTGTGAAGAAGGCCTTCGGGTTGTAAAGCACTTTCAG
CGAGGAGGAAGGCCAATAACTTAATACGTTGTTGGATTGACGTTACTCGCAGAAGAAGC
ACCGGCTAACTCCGTGCCAGCAGCCGCGGTAATACGGAGGGTGCAAGCGTTAATCGGA
ATTACTGGGCGTAAAGCGCACGCAGGCGGTTTGTTAAGTCAGATGTGAAATCCCCGCGC
TTAACGTGGGAACTGCATTTGAAACTGGCAAGCTAGAGTCTTGTAGAGGGGGGTAGAA
TTCCAGGTGTAGCGGTGAAATGCGTAGAGATCTGGAGGAATACCGGTGGCGAAGGCGG
CCCCCTGGACAAAGACTGACGCTCAGGTGCGAAAGCGTGGGGAGCAAACAGGATTAG
ATACCCTGGTAGTCCACGCTGTAAACGATGTCGACTTGGAGGTTGTGCCCTTGAGGCGT
GGCTTCCGGAGCTAACGCGTTAAGTCGACCGCCTGGGGAGTACGGCCGCAAGGTTAAA
ACTCAAATGAATTGACGGGGGCCCGCACAAGCGGTGGAGCATGTGGTTTAATTCGATGC
AACGCGAAGAACCTTACCTACTCTTGACATCCACGGAATTTAGCAGAGATGCTTTAGTG
CCTTCGGGAACCGTGAGACAGGTGCTGCATGGCTGTCGTCAGCTCGTGTTGTGAAATGT
TGGGTTAAGTCCCGCAACGAGCGCAACCCTTATCCTTTGTTGCCAGCACGTAATGGTGG
GAACTCAAAGGAGACTGCCGGTGATAAACCGGAGGAAGGTGGGGATGACGTCAAGTC
ATCATGGCCCTTACGAGTAGGGCTACACACGTGCTACAATGGCAGATACAAAGTGAAGC
GAACTCGCGAGAGCAAGCGGACCACATAAAGTCTGTCGTAGTCCGGATTGGAGTCTGC
AACTCGACTCCATGAAGTCGGAATCGCTAGTAATCGTAGATCAGAATGCTACGGTGAAT
ACGTTCCCGGGCCTTGTACACACCGCCCGTCACACCATGGGAGTGGGTTGCAAAAGAA
GTAGGTAGCTTAACCTTCGGGAGGGCGCTTACCACTTTGTGATTCATGACTGGGG
pattern 49

CGCTGGCGGCAGGCCTAACACATGCAAGTCGAGCGGCAGCGGGAAGTAGTTTACTACT
TTGCCGGCGAGCGGCGGACGGGTGAGTAATGTCTGGGAAACTGCCTGATGGAGGGGGA
TAACTACTGGAAACGGTAGCTAATACCGCATAACGTCTTCGGACCAAAGTGGGGGACCT
TCGGGCCTCACGCCATCGGATGTGCCCAGATGGGATTAGCTAGTAGGTGGGGTAATGGC
TCACCTAGGCGACGATCCCTAGCTGGTCTGAGAGGATGACCAGCCACACTGGAACTGA
GACACGGTCCAGACTCCTACGGGAGGCAGCAGTGGGGAATATTGCACAATGGGCGCAA
GCCTGATGCAGCCATGCCGCGTGTGTGAAGAAGGCCTTCGGGTTGTAAAGCACTTTCAG
CGAGGAGGAAGGCATAAAGGTTAATAACCTTTGTGATTGACGTTACTCGCAGAAGAAG
CACCGGCTAACTCCGTGCCAGCAGCCGCGGTAATACGGAGGGTGCAAGCGTTAATCGG
AATTACTGGGCGTAAAGCGCACGCAGGCGGTTTGTTAAGTCAGATGTGAAATCCCCGCG
CTTAACGTGGGAACTGCATTTGAAACTGGCAAGCTAGAGTCTTGTAGAGGGGGGTAGA
ATTCCAGGTGTAGCGGTGAAATGCGTAGAGATCTGGAGGAATACCGGTGGCGAAGGCG
GCCCCCTGGACAAAGACTGACGCTCAGGTGCGAAAGCGTGGGGAGCAAACAGGATTA
GATACCCTGGTAGTCCACGCTGTAAACGATGTCGACTTGGAGGTTGTGCCCTTGAGGCG
TGGCTTCCGGAGCTAACGCGTTAAGTCGACCGCCTGGGGAGTACGGCCGCAAGGTTAA
AACTCAAATGAATTGACGGGGGCCCGCACAAGCGGTGGAGCATGTGGTTTAATTCGATG
CAACGCGAAGAACCTTACCTACTCTTGACATCCACGGAATTTAGCAGAGATGCTTTAGT
GCCTTCGGGAACCGTGAGACAGGTGCTGCATGGCTGTCGTCAGCTCGTGTTGTGAAATG
TTGGGTTAAGTCCCGCAACGAGCGCAACCCTTATCCTTTGTTGCCAGCACGTGATGGTG
GGAACTCAAAGGAGACTGCCGGTGATAAACCGGAGGAAGGTGGGGATGACGTCAAGT
CATCATGGCCCTTACGAGTAGGGCTACACACGTGCTACAATGGCAGATACAAAGTGAAG
CGAACTCGCGAGAGCAAGCGGACCACATAAAGTCTGTCGTAGTTCGGATTGGAGTCTG
CAACTCGACTCCATGAAGTCGGAATCGCTAGTAATCGTAGATCAGAATGCTACGGTGAAT
ACGTTCCCGGGCCTTGTACACACCGCCCGTCACACCATGGGAGTGGGTTGCAAAAGAA
GTAGGTAGCTTAACCTTCGGGAGGGCGCTTACCACTTTGTGATTCATGACTGGGG
pattern 50
CGCTGGCGGCAGGCCTAACACATGCAAGTCGAGCGGCAGCGGGAAGTAGTTTACTACT
TTGCCGGCGAGCGGCGGACGGGTGAGTAATGTCTGGGAAACTGCCTGATGGAGGGGGA
TAACTACTGGAAACGGTAGCTAATACCGCATAACGTCTTCGGACCAAAGTGGGGGACCT
TCGGGCCTCACGCCATCGGATGTGCCCAGATGGGATTAGCTAGTAGGTGGGGTAATGGC
TCACCTAGGCGACGATCCCTAGCTGGTCTGAGAGGATGACCAGCCACACTGGAACTGA
GACACGGTCCAGACTCCTACGGGAGGCAGCAGTGGGGAATATTGCACAATGGGCGCAA
GCCTGATGCAGCCATGCCGCGTGTGTGAAGAAGGCCTTCGGGTTGTAAAGCACTTTCAG
CGAGGAGGAAGGCATAAAGGTTAATAACCTTTGTGATTGACGTTACTCGCAGAAGAAG
CACCGGCTAACTCCGTGCCAGCAGCCGCGGTAATACGGAGGGTGCAAGCGTTAATCGG
AATTACTGGGCGTAAAGCGCACGCAGGCGGTTTGTTAAGTCAGATGTGAAATCCCCGCG
CTTAACGTGGGAACTGCATTTGAAACTGGCAAGCTAGAGTCTTGTAGAGGGGGGTAGA
ATTCCAGGTGTAGCGGTGAAATGCGTAGAGATCTGGAGGAATACCGGTGGCGAAGGCG
GCCCCCTGGACAAAGACTGACGCTCAGGTGCGAAAGCGTGGGGAGCAAACAGGATTA
GATACCCTGGTAGTCCACGCTGTAAACGATGTCGACTTGGAGGTTGTGCCCTTGAGGCG
TGGCTTCCGGAGCTAACGCGTTAAGTCGACCGCCTGGGGAGTACGGCCGCAAGGTTAA
AACTCAAATGAATTGACGGGGGCCCGCACAAGCGGTGGAGCATGTGGTTTAATTCGATG
CAACGCGAAGAACCTTACCTACTCTTGACATCCAGGGAATTTAGCAGAGATGCTTTAGT
GCCTTCGGGAACCGTGAGACAGGTGCTGCATGGCTGTCGTCAGCTCGTGTTGTGAAATG

TTGGGTTAAGTCCCGCAACGAGCGCAACCCTTATCCTTTGTTGCCAGCACGTGATGGTG
GGAACTCAAAGGAGACTGCCGGTGATAAACCGGAGGAAGGTGGGGATGACGTCAAGT
CATCATGGCCCTTACGAGTAGGGCTACACACGTGCTACAATGGCAGATACAAAGTGAAG
CGAACTCGCGAGAGCAAGCGGACCACATAAAGTCTGTCGTAGTCCGGATTGGAGTCTG
CAACTCGACTCCATGAAGTCGGAATCGCTAGTAATCGTAGATCAGAATGCTACGGTGAAT
ACGTTCCCGGGCCTTGTACACACCGCCCGTCACACCATGGGAGTGGGTTGCAAAAGAA
GTAGGTAGCTTAACCTTCGGGAGGGCGCTTACCACTTTGTGATTCATGACTGGGG
pattern 51
CGCTGGCGGCAGGCCTAACACATGCAAGTCGAGCGGCAGCGGGAAGTAGTTTACTACT
TTGCCGGCGAGCGGCGGACGGGTGAGTAATGTCTGGGAAACTGCCTGATGGAGGGGGA
TAACTACTGGAAACGGTAGCTAATACCGCATGATCTCGAAAGAGCAAAGTGGGGGACCT
TCGGGCCTCACGCCATCGGATGTGCCCAGATGGGATTAGCTAGTAGGTGGGGTAATGGC
TCACCTAGGCGACGATCCCTAGCTGGTCTGAGAGGATGACCAGCCACACTGGAACTGA
GACACGGTCCAGACTCCTACGGGAGGCAGCAGTGGGGAATATTGCACAATGGGCGCAA
GCCTGATGCAGCCATGCCGCGTGTGTGAAGAAGGCCTTCGGGTTGTAAAGCACTTTCAG
CGAGGAGGAAGGCAATCGTGTTAATAGCACGATTGATTGACGTTACTCGCAGAAGAAG
CACCGGCTAACTCCGTGCCAGCAGCCGCGGTAATACGGAGGGTGCAAGCGTTAATCGG
AATTACTGGGCGTAAAGCGCACGCAGGCGGTTTGTTAAGTCAGATGTGAAATCCCCGCG
CTTAACGTGGGAACTGCATTTGAAACTGGCAAGCTAGAGTCTTGTAGAGGGGGGTAGA
ATTCCAGGTGTAGCGGTGAAATGCGTAGAGATCTGGAGGAATACCGGTGGCGAAGGCG
GCCCCCTGGACAAAGACTGACGCTCAGGTGCGAAAGCGTGGGGAGCAAACAGGATTA
GATACCCTGGTAGTCCACGCTGTAAACGATGTCGACTTGGAGGTTGTGCCCTTGAGGCG
TGGCTTCCGGAGCTAACGCGTTAAGTCGACCGCCTGGGGAGTACGGCCGCAAGGTTAA
AACTCAAATGAATTGACGGGGGCCCGCACAAGCGGTGGAGCATGTGGTTTAATTCGATG
CAACGCGAAGAACCTTACCTACTCTTGACATCCACAGAACTTAGCAGAGATGCTTCGGT
GCCTTCGGGAACTGTGAGACAGGTGCTGCATGGCTGTCGTCAGCTCGTGTTGTGAAATG
TTGGGTTAAGTCCCGCAACGAGCGCAACCCTTATCCTTTGTTGCCAGCGAGTAATGTCG
GGAACTCAAAGGAGACTGCCGGTGATAAACCGGAGGAAGGTGGGGATGACGTCAAGT
CATCATGGCCCTTACGAGTAGGGCTACACACGTGCTACAATGGCAGATACAAAGTGAAG
CGAACTCGCGAGAGCAAGCGGACCACATAAAGTCTGTCGTAGTCCGGATTGGAGTCTG
CAACTCGACTCCATGAAGTCGGAATCGCTAGTAATCGTAGATCAGAATGCTACGGTGAAT
ACGTTCCCGGGCCTTGTACACACCGCCCGTCACACCATGGGAGTGGGTTGCAAAAGAA
GTAGGTAGCTTAACCTTCGGGAGGGCGCTTACCACTTTGTGATTCATGACTGGGG
pattern 52
CGCTGGCGGCAGGCCTAACACATGCAAGTCGAGCGGCAGCGGGAAGTAGTTTACTACT
TTGCCGGCGAGCGGCGGACGGGTGAGTAATGTCTGGGAAACTGCCTGATGGAGGGGGA
TAACTACTGGAAACGGTAGCTAATACCGCATGACCTCGAAAGAGCAAAGTGGGGGACC
TTCGGGCCTCACGCCATCGGATGTGCCCAGATGGGATTAGCTAGTAGGTGGGGTAATGG
CTCACCTAGGCGACGATCCCTAGCTGGTCTGAGAGGATGACCAGCCACACTGGAACTG
AGACACGGTCCAGACTCCTACGGGAGGCAGCAGTGGGGAATATTGCACAATGGGCGCA
AGCCTGATGCAGCCATGCCGCGTGTGTGAAGAAGGCCTTCGGGTTGTAAAGCACTTTCA
GCGAGGAGGAAGGCAGTCGTGTTAATAGCACGATTGATTGACGTTACTCGCAGAAGAA
GCACCGGCTAACTCCGTGCCAGCAGCCGCGGTAATACGGAGGGTGCAAGCGTTAATCG
GAATTACTGGGCGTAAAGCGCACGCAGGCGGTTTGTTAAGTCAGATGTGAAATCCCCGC

GCTTAACGTGGGAACTGCATTTGAAACTGGCAAGCTAGAGTCTTGTAGAGGGGGGTAG
AATTCCAGGTGTAGCGGTGAAATGCGTAGAGATCTGGAGGAATACCGGTGGCGAAGGC
GGCCCCCTGGACAAAGACTGACGCTCAGGTGCGAAAGCGTGGGGAGCAAACAGGATT
AGATACCCTGGTAGTCCACGCTGTAAACGATGTCGACTTGGAGGTTGTGCCCTTGAGGC
GTGGCTTCCGGAGCTAACGCGTTAAGTCGACCGCCTGGGGAGTACGGCCGCAAGGTTA
AAACTCAAATGAATTGACGGGGGCCCGCACAAGCGGTGGAGCATGTGGTTTAATTCGAT
GCAACGCGAAGAACCTTACCTACTCTTGACATCCACAGAACTTAGCAGAGATGCTTCGG
TGCCTTCGGGAACTGTGAGACAGGTGCTGCATGGCTGTCGTCAGCTCGTGTTGTGAAAT
GTTGGGTTAAGTCCCGCAACGAGCGCAACCCTTATCCTTTGTTGCCAGCGAGTAATGTC
GGGAACTCAAAGGAGACTGCCGGTGATAAACCGGAGGAAGGTGGGGATGACGTCAAG
TCATCATGGCCCTTACGAGTAGGGCTACACACGTGCTACAATGGCAGATACAAAGTGAA
GCGAACTCGCGAGAGCAAGCGGACCACATAAAGTCTGTCGTAGTCCGGATTGGAGTCT
GCAACTCGACTCCATGAAGTCGGAATCGCTAGTAATCGTAGATCAGAATGCTACGGTGA
ATACGTTCCCGGGCCTTGTACACACCGCCCGTCACACCATGGGAGTGGGTTGCAAAAG
AAGTAGGTAGCTTAACCTTCGGGAGGGCGCTTACCACTTTGTGATTCATGACTGGGG
pattern 53
CGCTGGCGGCAGGCCTAACACATGCAAGTCGAGCGGCAGCGGGAAGTAGTTTACTACT
TCGCCGGCGAGCGGCGGACGGGTGAGTAATGTCTGGGAAACTGCCTGATGGAGGGGGA
TAACTACTGGAAACGGTAGCTAATACCGCATAACGTCTTCGGACCAAAGTGGGGGACCT
TCGGGCCTCACGCCATCGGATGTGCCCAGATGGGATTAGCTAGTAGGTGGGGTAATGGC
TCACCTAGGCGACGATCCCTAGCTGGTCTGAGAGGATGACCAGCCACACTGGAACTGA
GACACGGTCCAGACTCCTACGGGAGGCAGCAGTGGGGAATATTGCACAATGGGCGCAA
GCCTGATGCAGCCATGCCGCGTGTGTGAAGAAGGCCTTCGGGTTGTAAAGCACTTTCAG
CGAGGAGGAAGGCCAATAACTTAATAGGTTGTTGGATTGACGTTACTCGCAGAAGAAG
CACCGGCTAACTCCGTGCCAGCAGCCGCGGTAATACGGAGGGTGCAAGCGTTAATCGG
AATTACTGGGCGTAAAGCGCACGCAGGCGGTTTGTTAAGTCAGATGTGAAATCCCCGCG
CTTAACGTGGGAACTGCATTTGAAACTGGCAAGCTAGAGTCTTGTAGAGGGGGGTAGA
ATTCCAGGTGTAGCGGTGAAATGCGTAGAGATCTGGAGGAATACCGGTGGCGAAGGCG
GCCCCCTGGACAAAGACTGACGCTCAGGTGCGAAAGCGTGGGGAGCAAACAGGATTA
GATACCCTGGTAGTCCACGCTGTAAACGATGTCGACTTGGAGGTTGTGCCCTTGAGGCG
TGGCTTCCGGAGCTAACGCGTTAAGTCGACCGCCTGGGGAGTACGGCCGCAAGGTTAA
AACTCAAATGAATTGACGGGGGCCCGCACAAGCGGTGGAGCATGTGGTTTAATTCGATG
CAACGCGAAGAACCTTACCTACTCTTGACATCCACAGAACTTAGCAGAGATGCTTCGGT
GCCTTCGGGAACTGTGAGACAGGTGCTGCATGGCTGTCGTCAGCTCGTGTTGTGAAATG
TTGGGTTAAGTCCCGCAACGAGCGCAACCCTTATCCTTTGTTGCCAGCACGTAATGGTG
GGAACTCAAAGGAGACTGCCGGTGATAAACCGGAGGAAGGTGGGGATGACGTCAAGT
CATCATGGCCCTTACGAGTAGGGCTACACACGTGCTACAATGGCAGATACAAAGTGAAG
CGAACTCGCGAGAGCAAGCGGACCACATAAAGTCTGTCGTAGTCCGGATTGGAGTCTG
CAACTCGACTCCATGAAGTCGGAATCGCTAGTAATCGTAGATCAGAATGCTACGGTGAAT
ACGTTCCCGGGCCTTGTACACACCGCCCGTCACACCATGGGAGTGGGTTGCAAAAGAA
GTAGGTAGCTTAACCTTCGGGAGGGCGCTTACCACTTTGTGATTCATGACTGGGG
pattern 54
CGCTGGCGGCAGGCCTAACACATGCAAGTCGAGCGGCAGCGGGAAGTAGTTTACTACT
TTGCCGGCGAGCGGCGGACGGGTGAGTAATGTCTGGGAAACTGCCTGATGGAGGGGGA

TAACTACTGGAAACGGTAGCTAATACCGCATAACGTCTTCGGACCAAAGTGGGGGACCT
TCGGGCCTCACGCCATCGGATGTGCCCAGATGGGATTAGCTAGTAGGTGGGGTAATGGC
TCACCTAGGCGACGATCCCTAGCTGGTCTGAGAGGATGACCAGCCACACTGGAACTGA
GACACGGTCCAGACTCCTACGGGAGGCAGCAGTGGGGAATATTGCACAATGGGCGCAA
GCCTGATGCAGCCATGCCGCGTGTGTGAAGAAGGCCTTCGGGTTGTAAAGCACTTTCAG
CGAGGAGGAAGGCCAATAACTTAATACGTTGTTGGATTGACGTTACTCGCAGAAGAAGC
ACCGGCTAACTCCGTGCCAGCAGCCGCGGTAATACGGAGGGTGCAAGCGTTAATCGGA
ATTACTGGGCGTAAAGCGCACGCAGGCGGTTTGTTAAGTCAGATGTGAAATCCCCGCGC
TTAACGTGGGAACTGCATTTGAAACTGGCAAGCTAGAGTCTTGTAGAGGGGGGTAGAA
TTCCAGGTGTAGCGGTGAAATGCGTAGAGATCTGGAGGAATACCGGTGGCGAAGGCGG
CCCCCTGGACAAAGACTGACGCTCAGGTGCGAAAGCGTGGGGAGCAAACAGGATTAG
ATACCCTGGTAGTCCACGCTGTAAACGATGTCGACTTGGAGGTTGTGCCCTTGAGGCGT
GGCTTCCGGAGCTAACGCGTTAAGTCGACCGCCTGGGGAGTACGGCCGCAAGGTTAAA
ACTCAAATGAATTGACGGGGGCCCGCACAAGCGGTGGAGCATGTGGTTTAATTCGATGC
AACGCGAAGAACCTTACCTACTCTTGACATCCACAGAACTTAGCAGAGATGCTTCGGTG
CCTTCGGGAACTGTGAGACAGGTGCTGCATGGCTGTCGTCAGCTCGTGTTGTGAAATGT
TGGGTTAAGTCCCGCAACGAGCGCAACCCTTATCCTTTGTTGCCAGCACGTAATGGTGG
GAACTCAAAGGAGACTGCCGGTGATAAACCGGAGGAAGGTGGGGATGACGTCAAGTC
ATCATGGCCCTTACGAGTAGGGCTACACACGTGCTACAATGGCAGATACAAAGTGAAGC
GAACTCGCGAGAGCAAGCGGACCACATAAAGTCTGTCGTAGTCCGGATTGGAGTCTGC
AACTCGACTCCATGAAGTCGGAATCGCTAGTAATCGTAGATCAGAATGCTACGGTGAAT
ACGTTCCCGGGCCTTGTACACACCGCCCGTCACACCATGGGAGTGGGTTGCAAAAGAA
GTAGGTAGCTTAACCTTCGGGAGGGCGCTTACCACTTTGTGATTCATGACTGGGG
pattern 55
CGCTGGCGGCAGGCCTAACACATGCAAGTCGAGCGGCAGCGGGAAGTAGCTTGCTACT
TTGCCGGCGAGCGGCGGACGGGTGAGTAATGTCTGGGAAACTGCCTGATGGAGGGGGA
TAACTACTGGAAACGGTAGCTAATACCGCATAACGTCTTCGGACCAAAGTGGGGGACCT
TCGGGCCTCACGCCATCGGATGTGCCCAGATGGGATTAGCTAGTAGGTGGGGTAATGGC
TCACCTAGGCGACGATCCCTAGCTGGTCTGAGAGGATGACCAGCCACACTGGAACTGA
GACACGGTCCAGACTCCTACGGGAGGCAGCAGTGGGGAATATTGCACAATGGGCGCAA
GCCTGATGCAGCCATGCCGCGTGTGTGAAGAAGGCCTTCGGGTTGTAAAGCACTTTCAG
CGAGGAGGAAGGCATAAAGGTTAATAACCTTTGTGATTGACGTTACTCGCAGAAGAAG
CACCGGCTAACTCCGTGCCAGCAGCCGCGGTAATACGGAGGGTGCAAGCGTTAATCGG
AATTACTGGGCGTAAAGCGCACGCAGGCGGTTTGTTAAGTCAGATGTGAAATCCCCGCG
CTTAACGTGGGAACTGCATTTGAAACTGGCAAGCTAGAGTCTTGTAGAGGGGGGTAGA
ATTCCAGGTGTAGCGGTGAAATGCGTAGAGATCTGGAGGAATACCGGTGGCGAAGGCG
GCCCCCTGGACAAAGACTGACGCTCAGGTGCGAAAGCGTGGGGAGCAAACAGGATTA
GATACCCTGGTAGTCCACGCTGTAAACGATGTCGACTTGGAGGTTGTGCCCTTGAGGCG
TGGCTTCCGGAGCTAACGCGTTAAGTCGACCGCCTGGGGAGTACGGCCGCAAGGTTAA
AACTCAAATGAATTGACGGGGGCCCGCACAAGCGGTGGAGCATGTGGTTTAATTCGATG
CAACGCGAAGAACCTTACCTACTCTTGACATCCACGGAATTTAGCAGAGATGCTTTAGT
GCCTTCGGGAACCGTGAGACAGGTGCTGCATGGCTGTCGTCAGCTCGTGTTGTGAAATG
TTGGGTTAAGTCCCGCAACGAGCGCAACCCTTATCCTTTGTTGCCAGCACGTCATGGTG
GGAACTCAAAGGAGACTGCCGGTGATAAACCGGAGGAAGGTGGGGATGACGTCAAGT

CATCATGGCCCTTACGAGTAGGGCTACACACGTGCTACAATGGCAGATACAAAGTGAAG
CGAACTCGCGAGAGCAAGCGGACCACATAAAGTCTGTCGTAGTCCGGATTGGAGTCTG
CAACTCGACTCCATGAAGTCGGAATCGCTAGTAATCGTAGATCAGAATGCTACGGTGAAT
ACGTTCCCGGGCCTTGTACACACCGCCCGTCACACCATGGGAGTGGGTTGCAAAAGAA
GTAGGTAGCTTAACCTTCGGGAGGGCGCTTACCACTTTGTGATTCATGACTGGGG
pattern 56
CGCTGGCGGCAGGCCTAACACATGCAAGTCGAGCGGCAGCGGGAAGTAGCTTGCTACT
TTGCCGGCGAGCGGCGGACGGGTGAGTAATGTCTGGGAAACTGCCTGATGGAGGGGGA
TAACTACTGGAAACGGTAGCTAATACCGCATAACGTCTTCGGACCAAAGTGGGGGACCT
TCGGGCCTCACGCCATCGGATGTGCCCAGATGGGATTAGCTAGTAGGTGGGGTAATGGC
TCACCTAGGCGACGATCCCTAGCTGGTCTGAGAGGATGACCAGCCACACTGGAACTGA
GACACGGTCCAGACTCCTACGGGAGGCAGCAGTGGGGAATATTGCACAATGGGCGCAA
GCCTGATGCAGCCATGCCGCGTGTGTGAAGAAGGCCTTCGGGTTGTAAAGCACTTTCAG
CGAGGAGGAAGGCATAAAGGTTAATAACCTTTGTGATTGACGTTACTCGCAGAAGAAG
CACCGGCTAACTCCGTGCCAGCAGCCGCGGTAATACGGAGGGTGCAAGCGTTAATCGG
AATTACTGGGCGTAAAGCGCACGCAGGCGGTTTGTTAAGTCAGATGTGAAATCCCCGCG
CTTAACGTGGGAACTGCATTTGAAACTGGCAAGCTAGAGTCTTGTAGAGGGGGGTAGA
ATTCCAGGTGTAGCGGTGAAATGCGTAGAGATCTGGAGGAATACCGGTGGCGAAGGCG
GCCCCCTGGACAAAGACTGACGCTCAGGTGCGAAAGCGTGGGGAGCAAACAGGATTA
GATACCCTGGTAGTCCACGCTGTAAACGATGTCGACTTGGAGGTTGTGCCCTTGAGGCG
TGGCTTCCGGAGCTAACGCGTTAAGTCGACCGCCTGGGGAGTACGGCCGCAAGGTTAA
AACTCAAATGAATTGACGGGGGCCCGCACAAGCGGTGGAGCATGTGGTTTAATTCGATG
CAACGCGAAGAACCTTACCTACTCTTGACATCCACAGAACTTAGCAGAGATGCTTCGGT
GCCTTCGGGAACTGTGAGACAGGTGCTGCATGGCTGTCGTCAGCTCGTGTTGTGAAATG
TTGGGTTAAGTCCCGCAACGAGCGCAACCCTTATCCTTTGTTGCCAGCACGTCATGGTG
GGAACTCAAAGGAGACTGCCGGTGATAAACCGGAGGAAGGTGGGGATGACGTCAAGT
CATCATGGCCCTTACGAGTAGGGCTACACACGTGCTACAATGGCAGATACAAAGTGAAG
CGAACTCGCGAGAGCAAGCGGACCACATAAAGTCTGTCGTAGTCCGGATTGGAGTCTG
CAACTCGACTCCATGAAGTCGGAATCGCTAGTAATCGTAGATCAGAATGCTACGGTGAAT
ACGTTCCCGGGCCTTGTACACACCGCCCGTCACACCATGGGAGTGGGTTGCAAAAGAA
GTAGGTAGCTTAACCTTCGGGAGGGCGCTTACCACTTTGTGATTCATGACTGGGG
pattern 57
CGCTGGCGGCAGGCCTAACACATGCAAGTCGAGCGGCAGCGGGAAGTAGCTTGCTACT
TTGCCGGCGAGCGGCGGACGGGTGAGTAATGTCTGGGAAACTGCCTGATGGAGGGGGA
TAACTACTGGAAACGGTAGCTAATACCGCATAACGTCTTCGGACCAAAGTGGGGGACCT
TCGGGCCTCACGCCATCGGATGTGCCCAGATGGGATTAGCTAGTAGGTGGGGTAATGGC
TCACCTAGGCGACGATCCCTAGCTGGTCTGAGAGGATGACCAGCCACACTGGAACTGA
GACACGGTCCAGACTCCTACGGGAGGCAGCAGTGGGGAATATTGCACAATGGGCGCAA
GCCTGATGCAGCCATGCCGCGTGTGTGAAGAAGGCCTTCGGGTTGTAAAGCACTTTCAG
CGAGGAGGAAGGCATAAAGGTTAATAACCTTTGTGATTGACGTTACTCGCAGAAGAAG
CACCGGCTAACTCCGTGCCAGCAGCCGCGGTAATACGGAGGGTGCAAGCGTTAATCGG
AATTACTGGGCGTAAAGCGCACGCAGGCGGTTTGTTAAGTCAGATGTGAAATCCCCGCG
CTTAACGTGGGAACTGCATTTGAAACTGGCAAGCTAGAGTCTTGTAGAGGGGGGTAGA
ATTCCAGGTGTAGCGGTGAAATGCGTAGAGATCTGGAGGAATACCGGTGGCGAAGGCG

GCCCCCTGGACAAAGACTGACGCTCAGGTGCGAAAGCGTGGGGAGCAAACAGGATTA
GATACCCTGGTAGTCCACGCTGTAAACGATGTCGACTTGGAGGTTGTGCCCTTGAGGCG
TGGCTTCCGGAGCTAACGCGTTAAGTCGACCGCCTGGGGAGTACGGCCGCAAGGTTAA
AACTCAAATGAATTGACGGGGGCCCGCACAAGCGGTGGAGCATGTGGTTTAATTCGATG
CAACGCGAAGAACCTTACCTACTCTTGACATCCACGGAATTTAGCAGAGATGCTTTAGT
GCCTTCGGGAACCGTGAGACAGGTGCTGCATGGCTGTCGTCAGCTCGTGTTGTGAAATG
TTGGGTTAAGTCCCGCAACGAGCGCAACCCTTATCCTTTGTTGCCAGCACGTCATGGTG
GGAACTCAAAGGAGACTGCCGGTGATAAACCGGAGGAAGGTGGGGATGACGTCAAGT
CATCATGGCCCTTACGAGTAGGGCTACACACGTGCTACAATGGCAGATACAAAGTGAAG
CAAACTCGCGAGAGCAAGCGGACCACATAAAGTCTGTCGTAGTCCGGATTGGAGTCTG
CAACTCGACTCCATGAAGTCGGAATCGCTAGTAATCGTAGATCAGAATGCTACGGTGAAT
ACGTTCCCGGGCCTTGTACACACCGCCCGTCACACCATGGGAGTGGGTTGCAAAAGAA
GTAGGTAGCTTAACCTTCGGGAGGGCGCTTACCACTTTGTGATTCATGACTGGGG
pattern 58
CGCTGGCGGCAGGCCTAACACATGCAAGTCGAGCGGCAGCGGGAAGTAGTTTACTACT
TTGCCGGCGAGCGGCGGACGGGTGAGTAATGTCTGGGAAACTGCCTGATGGAGGGGGA
TAACTACTGGAAACGGTAGCTAATACCGCATAACGTCTTCGGACCAAAGTGGGGGACCT
TCGGGCCTCACGCCATCGGATGTGCCCAGATGGGATTAGCTAGTAGGTGGGGTAATGGC
TCACCTAGGCGACGATCCCTAGCTGGTCTGAGAGGATGACCAGCCACACTGGAACTGA
GACACGGTCCAGACTCCTACGGGAGGCAGCAGTGGGGAATATTGCACAATGGGCGCAA
GCCTGATGCAGCCATGCCGCGTGTGTGAAGAAGGCCTTCGGGTTGTAAAGCACTTTCAG
CGAGGAGGAAGGCATAAAGGTTAATAACCTTTGTGATTGACGTTACTCGCAGAAGAAG
CACCGGCTAACTCCGTGCCAGCAGCCGCGGTAATACGGAGGGTGCAAGCGTTAATCGG
AATTACTGGGCGTAAAGCGCACGCAGGCGGTTTGTTAAGTCAGATGTGAAATCCCCGCG
CTTAACGTGGGAACTGCATTTGAAACTGGCAAGCTAGAGTCTTGTAGAGGGGGGTAGA
ATTCCAGGTGTAGCGGTGAAATGCGTAGAGATCTGGAGGAATACCGGTGGCGAAGGCG
GCCCCCTGGACAAAGACTGACGCTCAGGTGCGAAAGCGTGGGGAGCAAACAGGATTA
GATACCCTGGTAGTCCACGCTGTAAACGATGTCGACTTGGAGGTTGTGCCCTTGAGGCG
TGGCTTCCGGAGCTAACGCGTTAAGTCGACCGCCTGGGGAGTACGGCCGCAAGGTTAA
AACTCAAATGAATTGACGGGGGCCCGCACAAGCGGTGGAGCATGTGGTTTAATTCGATG
CAACGCGAAGAACCTTACCTACTCTTGACATCCACAGAACTTAGCAGAGATGCTTCGGT
GCCTTCGGGAACTGTGAGACAGGTGCTGCATGGCCGTCGTCAGCTCGTGTTGTGAAATG
TTGGGTTAAGTCCCGCAACGAGCGCAACCCTTATCCTTTGTTGCCAGCACGTCATGGTG
GGAACTCAAAGGAGACTGCCGGTGATAAACCGGAGGAAGGTGGGGATGACGTCAAGT
CATCATGGCCCTTACGAGTAGGGCTACACACGTGCTACAATGGCAGATACAAAGTGAAG
CAAACTCGCGAGAGCAAGCGGACCACATAAAGTCTGTCGTAGTCCGGATTGGAGTCTG
CAACTCGACTCCATGAAGTCGGAATCGCTAGTAATCGTAGATCAGAATGCTACGGTGAAT
ACGTTCCCGGGCCTTGTACACACCGCCCGTCACACCATGGGAGTGGGTTGCAAAAGAA
GTAGGTAGCTTAACCTTCGGGAGGGCGCTTACCACTTTGTGATTCATGACTGGGG
pattern 59
CGCTGGCGGCAGGCCTAACACATGCAAGTCGAGCGGCAGCGGGAAGTAGTTTACTACT
TTGCCGGCGAGCGGCGGACGGGTGAGTAATGTCTGGGAAACTGCCTGATGGAGGGGGA
TAACTACTGGAAACGGTAGCTAATACCGCATGACCTCGCAAGAGCAAAGTGGGGGACC
TTCGGGCCTCACGCCATCGGATGTGCCCAGATGGGATTAGCTAGTAGGTGGGGTAATGG

CTCACCTAGGCGACGATCCCTAGCTGGTCTGAGAGGATGACCAGCCACACTGGAACTG
AGACACGGTCCAGACTCCTACGGGAGGCAGCAGTGGGGAATATTGCACAATGGGCGCA
AGCCTGATGCAGCCATGCCGCGTGTGTGAAGAAGGCCTTCGGGTTGTAAAGCACTTTCA
GCGAGGAGGAAGGCAATCGTGTTAATAGCACGATTGATTGACGTTACTCGCAGAAGAA
GCACCGGCTAACTCCGTGCCAGCAGCCGCGGTAATACGGAGGGTGCAAGCGTTAATCG
GAATTACTGGGCGTAAAGCGCACGCAGGCGGTTTGTTAAGTCAGATGTGAAATCCCCGC
GCTTAACGTGGGAACTGCATTTGAAACTGGCAAGCTAGAGTCTTGTAGAGGGGGGTAG
AATTCCAGGTGTAGCGGTGAAATGCGTAGAGATCTGGAGGAATACCGGTGGCGAAGGC
GGCCCCCTGGACAAAGACTGACGCTCAGGTGCGAAAGCGTGGGGAGCAAACAGGATT
AGATACCCTGGTAGTCCACGCTGTAAACGATGTCGACTTGGAGGTTGTGCCCTTGAGGC
GTGGCTTCCGGAGCTAACGCGTTAAGTCGACCGCCTGGGGAGTACGGCCGCAAGGTTA
AAACTCAAATGAATTGACGGGGGCCCGCACAAGCGGTGGAGCATGTGGTTTAATTCGAT
GCAACGCGAAGAACCTTACCTACTCTTGACATCCACGGAATTTAGCAGAGATGCTTTAG
TGCCTTCGGGAACCGTGAGACAGGTGCTGCATGGCTGTCGTCAGCTCGTGTTGTGAAAT
GTTGGGTTAAGTCCCGCAACGAGCGCAACCCTTATCCTTTGTTGCCAGCACGTAATGGT
GGGAACTCAAGGGAGACTGCCGGTGACAAACCGGAGGAAGGTGGGGATGACGTCAAG
TCATCATGGCCCTTACGAGTAGGGCTACACACGTGCTACAATGGCAGATACAAAGTGAA
GCGAACTCGCGAGAGCAAGCGGACCACATAAAGTCTGTCGTAGTCCGGATTGGAGTCT
GCAACTCGACTCCATGAAGTCGGAATCGCTAGTAATCGTAGATCAGAATGCTACGGTGA
ATACGTTCCCGGGCCTTGTACACACCGCCCGTCACACCATGGGAGTGGGTTGCAAAAG
AAGTAGGTAGCTTAACCTTCGGGAGGGCGCTTACCACTTTGTGATTCATGACTGGGG
pattern 60
CGCTGGCGGCAGGCCTAACACATGCAAGTCGAGCGGCAGCGGGAAGTAGTTTACTACT
TTGCCGGCGAGCGGCGGACGGGTGAGTAATGTCTGGGAAACTGCCTGATGGAGGGGGA
TAACTACTGGAAACGGTAGCTAATACCGCATGACCTCGTAAGAGCAAAGTGGGGGACCT
TCGGGCCTCACGCCATCGGATGTGCCCAGATGGGATTAGCTAGTAGGTGGGGTAATGGC
TCACCTAGGCGACGATCCCTAGCTGGTCTGAGAGGATGACCAGCCACACTGGAACTGA
GACACGGTCCAGACTCCTACGGGAGGCAGCAGTGGGGAATATTGCACAATGGGCGCAA
GCCTGATGCAGCCATGCCGCGTGTGTGAAGAAGGCCTTCGGGTTGTAAAGCACTTTCAG
CGAGGAGGAAGGCAATCGTGTTAATAGCACGATTGATTGACGTTACTCGCAGAAGAAG
CACCGGCTAACTCCGTGCCAGCAGCCGCGGTAATACGGAGGGTGCAAGCGTTAATCGG
AATTACTGGGCGTAAAGCGCACGCAGGCGGTTTGTTAAGTCAGATGTGAAATCCCCGCG
CTTAACGTGGGAACTGCATTTGAAACTGGCAAGCTAGAGTCTTGTAGAGGGGGGTAGA
ATTCCAGGTGTAGCGGTGAAATGCGTAGAGATCTGGAGGAATACCGGTGGCGAAGGCG
GCCCCCTGGACAAAGACTGACGCTCAGGTGCGAAAGCGTGGGGAGCAAACAGGATTA
GATACCCTGGTAGTCCACGCTGTAAACGATGTCGACTTGGAGGTTGTGCCCTTGAGGCG
TGGCTTCCGGAGCTAACGCGTTAAGTCGACCGCCTGGGGAGTACGGCCGCAAGGTTAA
AACTCAAATGAATTGACGGGGGCCCGCACAAGCGGTGGAGCATGTGGTTTAATTCGATG
CAACGCGAAGAACCTTACCTACTCTTGACATCCACAGAACTTAGCAGAGATGCTTCGGT
GCCTTCGGGAACTGTGAGACAGGTGCTGCATGGCTGTCGTCAGCTCGTGTTGTGAAATG
TTGGGTTAAGTCCCGCAACGAGCGCAACCCTTATCCTTTGTTGCCAGCACGTAATGGTG
GGAACTCAAGGGAGACTGCCGGTGACAAACCGGAGGAAGGTGGGGATGACGTCAAGT
CATCATGGCCCTTACGAGTAGGGCTACACACGTGCTACAATGGCAGATACAAAGTGAAG
CGAACTCGCGAGAGCAAGCGGACCACATAAAGTCTGTCGTAGTCCGGATTGGAGTCTG

CAACTCGACTCCATGAAGTCGGAATCGCTAGTAATCGTAGATCAGAATGCTACGGTGAAT
ACGTTCCCGGGCCTTGTACACACCGCCCGTCACACCATGGGAGTGGGTTGCAAAAGAA
GTAGGTAGCTTAACCTTCGGGAGGGCGCTTACCACTTTGTGATTCATGACTGGGG
pattern 61
CGCTGGCGGCAGGCCTAACACATGCAAGTCGAGCGGCAGCGGGAAGTAGTTTACTACT
TTGCCGGCGAGCGGCGGACGGGTGAGTAATGTCTGGGGATCTGCCTGATGGAGGGGGA
TAACTACTGGAAACGGTAGCTAATACCGCGTGACCTCGCAAGAGCAAAGTGGGGGACC
TTAGGGCCTCACGCCATCGGATGAACCCAGATGGGATTAGCTAGTAGGTGGGGTAATGG
CTCACCTAGGCGACGATCCCTAGCTGGTCTGAGAGGATGACCAGCCACACTGGAACTG
AGACACGGTCCAGACTCCTACGGGAGGCAGCAGTGGGGAATATTGCACAATGGGCGCA
AGCCTGATGCAGCCATGCCGCGTGTGTGAAGAAGGCCTTCGGGTTGTAAAGCACTTTCA
GCGAGGAGGAAGGGGTTGAGTTTAATACGCTCAATCATTGACGTTACTCGCAGAAGAA
GCACCGGCTAACTCCGTGCCAGCAGCCGCGGTAATACGGAGGGTGCAAGCGTTAATCG
GAATTACTGGGCGTAAAGCGCACGCAGGCGGTTTGTTAAGTCAGATGTGAAATCCCCGC
GCTTAACGTGGGAACTGCATTTGAAACTGGCAAGCTAGAGTCTTGTAGAGGGGGGTAG
AATTCCAGGTGTAGCGGTGAAATGCGTAGAGATCTGGAGGAATACCGGTGGCGAAGGC
GGCCCCCTGGACAAAGACTGACGCTCAGGTGCGAAAGCGTGGGGAGCAAACAGGATT
AGATACCCTGGTAGTCCACGCTGTAAACGATGTCGACTTGGAGGTTGTGCCCTTGAGGC
GTGGCTTCCGGAGCTAACGCGTTAAGTCGACCGCCTGGGGAGTACGGCCGCAAGGTTA
AAACTCAAATGAATTGACGGGGGCCCGCACAAGCGGTGGAGCATGTGGTTTAATTCGAT
GCAACGCGAAGAACCTTACCTACTCTTGACATCCACAGAATTTGGCAGAGATGCTAAAG
TGCCTTCGGGAACTGTGAGACAGGTGCTGCATGGCTGTCGTCAGCTCGTGTTGTGAAAT
GTTGGGTTAAGTCCCGCAACGAGCGCAACCCTTATCCTTTGTTGCCAGCACGTAATGGT
GGGAACTCAAGGGAGACTGCCGGTGACAAACCGGAGGAAGGTGGGGATGACGTCAAG
TCATCATGGCCCTTACGAGTAGGGCTACACACGTGCTACAATGGCAGATACAAAGTGAA
GCGAACTCGCGAGAGCCAGCGGACCACATAAAGTCTGTCGTAGTCCGGATTGGAGTCT
GCAACTCGACTCCATGAAGTCGGAATCGCTAGTAATCGTAGATCAGAATGCTACGGTGA
ATACGTTCCCGGGCCTTGTACACACCGCCCGTCACACCATGGGAGTGGGTTGCAAAAG
AAGTAGGTAGCTTAACCTTCGGGAGGGCGCTTACCACTTTGTGATTCATGACTGGGG
pattern 62
CGCTGGCGGCAGGCCTAACACATGCAAGTCGAGCGGCAGCGGGAAGTAGTTTACTACT
TTGCCGGCGAGCGGCGGACGGGTGAGTAATGTCTGGGGATCTGCCTGATGGAGGGGGA
TAACTACTGGAAACGGTAGCTAATACCGCATGACCTCGCAAGAGCAAAGTGGGGGACC
TTAGGGCCTCACGCCATCGGATGAACCCAGATGGGATTAGCTAGTAGGTGGGGTAATGG
CTCACCTAGGCGACGATCCCTAGCTGGTCTGAGAGGATGACCAGCCACACTGGAACTG
AGACACGGTCCAGACTCCTACGGGAGGCAGCAGTGGGGAATATTGCACAATGGGCGCA
AGCCTGATGCAGCCATGCCGCGTGTGTGAAGAAGGCCTTCGGGTTGTAAAGCACTTTCA
GCGAGGAGGAAGGGGTTGAGTTTAATACGCTCAATCATTGACGTTACTCGCAGAAGAA
GCACCGGCTAACTCCGTGCCAGCAGCCGCGGTAATACGGAGGGTGCAAGCGTTAATCG
GAATTACTGGGCGTAAAGCGCACGCAGGCGGTTTGTTAAGTCAGATGTGAAATCCCCGC
GCTTAACGTGGGAACTGCATTTGAAACTGGCAAGCTAGAGTCTTGTAGAGGGGGGTAG
AATTCCAGGTGTAGCGGTGAAATGCGTAGAGATCTGGAGGAATACCGGTGGCGAAGGC
GGCCCCCTGGACAAAGACTGACGCTCAGGTGCGAAAGCGTGGGGAGCAAACAGGATT
AGATACCCTGGTAGTCCACGCTGTAAACGATGTCGACTTGGAGGTTGTGCCCTTGAGGC

GTGGCTTCCGGAGCTAACGCGTTAAGTCGACCGCCTGGGGAGTACGGCCGCAAGGTTA
AAACTCAAATGAATTGACGGGGGCCCGCACAAGCGGTGGAGCATGTGGTTTAATTCGAT
GCAACGCGAAGAACCTTACCTACTCTTGACATCCACAGAATTTGGCAGAGATGTTAAAG
TGCCTTCGGGAACTGTGAGACAGGTGCTGCATGGCTGTCGTCAGCTCGTGTTGTGAAAT
GTTGGGTTAAGTCCCGCAACGAGCGCAACCCTTATCCTTTGTTGCCAGCACGTAATGGT
GGGAACTCAAGGGAGACTGCCGGTGACAAACCGGAGGAAGGTGGGGATGACGTCAAG
TCATCATGGCCCTTACGAGTAGGGCTACACACGTGCTACAATGGCAGATACAAAGTGAA
GCGAACTCGCGAGAGCCAGCGGACCACATAAAGTCTGTCGTAGTCCGGATTGGAGTCT
GCAACTCGACTCCATGAAGTCGGAATCGCTAGTAATCGTAGATCAGAATGCTACGGTGA
ATACGTTCCCGGGCCTTGTACACACCGCCCGTCACACCATGGGAGTGGGTTGCAAAAG
AAGTAGGTAGCTTAACCTTCGGGAGGGCGCTTACCACTTTGTGATTCATGACTGGGG
pattern 63
CGCTGGCGGCAGGCCTAACACATGCAAGTCGAGCGGCAGCGGAAAGTAGCTTGCTACT
TTGCCGGCGAGCGGCGGACGGGTGAGTAATGTCTGGGAAACTGCCTGATGGAGGGGGA
TAACTACTGGAAACGGTAGCTAATACCGCATGACCTCGAAAGAGCAAAGTGGGGGACC
TTCGGGCCTCACGCCATCGGATGTGCCCAGATGGGATTAGCTAGTAGGTGAGGTAATGG
CTCACCTAGGCGACGATCCCTAGCTGGTCTGAGAGGATGACCAGCCACACTGGAACTG
AGACACGGTCCAGACTCCTACGGGAGGCAGCAGTGGGGAATATTGCACAATGGGCGCA
AGCCTGATGCAGCCATGCCGCGTGTGTGAAGAAGGCCTTCGGGTTGTAAAGCACTTTCA
GCGAGGAGGAAGGCATTTCACTTAATACGTGAAGTGATTGACGTTACTCGCAGAAGAA
GCACCGGCTAACTCCGTGCCAGCAGCCGCGGTAATACGGAGGGTGCAAGCGTTAATCG
GAATTACTGGGCGTAAAGCGCACGCAGGCGGTTTGTTAAGTCAGATGTGAAATCCCCGA
GCTTAACTTGGGAACTGCATTTGAAACTGGCAAGCTAGAGTCTTGTAGAGGGGGGTAG
AATTCCAGGTGTAGCGGTGAAATGCGTAGAGATCTGGAGGAATACCGGTGGCGAAGGC
GGCCCCCTGGACAAAGACTGACGCTCAGGTGCGAAAGCGTGGGGAGCAAACAGGATT
AGATACCCTGGTAGTCCACGCTGTAAACGATGTCGACTTGGAGGTTGTGCCCTTGAGGC
GTGGCTTCCGGAGCTAACGCGTTAAGTCGACCGCCTGGGGAGTACGGCCGCAAGGTTA
AAACTCAAATGAATTGACGGGGGCCCGCACAAGCGGTGGAGCATGTGGTTTAATTCGAT
GCAACGCGAAGAACCTTACCTACTCTTGACATCCACAGAACTTAGCAGAGATGCTTAGG
TGCCTTCGGGAACTGTGAGACAGGTGCTGCATGGCTGTCGTCAGCTCGTGTTGTGAAAT
GTTGGGTTAAGTCCCGCAACGAGCGCAACCCTTATCCTTTGTTGCCAGCACGTAATGGT
GGGAACTCAAAGGAGACTGCCGGTGATAAACCGGAGGAAGGTGGGGATGACGTCAAG
TCATCATGGCCCTTACGAGTAGGGCTACACACGTGCTACAATGGCAGATACAAAGTGAA
GCGAACTCGCGAGAGCAAGCGGACCACATAAAGTCTGTCGTAGTCCGGATTGGAGTCT
GCAACTCGACTCCATGAAGTCGGAATCGCTAGTAATCGTAGATCAGAATGCTACGGTGA
ATACGTTCCCGGGCCTTGTACACACCGCCCGTCACACCATGGGAGTGGGTTGCAAAAG
AAGTAGGTAGCTTAACCTTCGGGAGGGCGCTTACCACTTTGTGATTCATGACTGGGG
pattern 64
CGCTGGCGGCAGGCCTAACACATGCAAGTCGAGCGGCAGCGGAAAGTAGCTTGCTACT
TTGCCGGCGAGCGGCGGACGGGTGAGTAATGTCTGGGGATCTGCCTGATGGAGGGGGA
TAACTACTGGAAACGGTAGCTAATACCGCATGACCTCGAAAGAGCAAAGTGGGGGACC
TTCGGGCCTCACGCCATCGGATGAACCCAGATGGGATTAGCTAGTAGGTGGGGTAATGG
CTCACCTAGGCGACGATCCCTAGCTGGTCTGAGAGGATGACCAGCCACACTGGAACTG
AGACACGGTCCAGACTCCTACGGGAGGCAGCAGTGGGGAATATTGCACAATGGGCGCA

AGCCTGATGCAGCCATGCCGCGTGTGTGAAGAAGGCCTTCGGGTTGTAAAGCACTTTCA
GCGAGGAGGAAGGCATTGTGGTTAATAACCGCAGTGATTGACGTTACTCGCAGAAGAA
GCACCGGCTAACTCCGTGCCAGCAGCCGCGGTAATACGGAGGGTGCAAGCGTTAATCG
GAATTACTGGGCGTAAAGCGCACGCAGGCGGTTTGTTAAGTCAGATGTGAAATCCCCGC
GCTTAACGTGGGAACTGCATTTGAAACTGGCAAGCTAGAGTCTTGTAGAGGGGGGTAG
AATTCCAGGTGTAGCGGTGAAATGCGTAGAGATCTGGAGGAATACCGGTGGCGAAGGC
GGCCCCCTGGACAAAGACTGACGCTCAGGTGCGAAAGCGTGGGGAGCAAACAGGATT
AGATACCCTGGTAGTCCACGCTGTAAACGATGTCGACTTGGAGGTTGTGCCCTTGAGGC
GTGGCTTCCGGAGCTAACGCGTTAAGTCGACCGCCTGGGGAGTACGGCCGCAAGGTTA
AAACTCAAATGAATTGACGGGGGCCCGCACAAGCGGTGGAGCATGTGGTTTAATTCGAT
GCAACGCGAAGAACCTTACCTACTCTTGACATCCACAGAACTTAGCAGAGATGCTTCGG
TGCCTTCGGGAACTGTGAGACAGGTGCTGCATGGCTGTCGTCAGCTCGTGTTGTGAAAT
GTTGGGTTAAGTCCCGCAACGAGCGCAACCCTTATCCTTTGTTGCCAGCACGTAATGGT
GGGAACTCAAGGGAGACTGCCGGTGACAAACCGGAGGAAGGTGGGGATGACGTCAAG
TCATCATGGCCCTTACGAGTAGGGCTACACACGTGCTACAATGGCAGATACAAAGTGAA
GCGAACTCGCGAGAGCAAGCGGACCACATAAAGTCTGTCGTAGTCCGGATTGGAGTCT
GCAACTCGACTCCATGAAGTCGGAATCGCTAGTAATCGTAGATCAGAATGCTACGGTGA
ATACGTTCCCGGGCCTTGTACACACCGCCCGTCACACCATGGGAGTAGGTTGCAAAAGA
AGTAGGTAGCTTAACCTTCGGGAGGGCGCTTACCACTTTGTGATTCATGACTGGGG
pattern 65
CGCTGGCGGCAGGCCTAACACATGCAAGTCGAGCGGCAGTGGGAAGTAGTTTACTACT
TTGCCGGCGAGCGGCGGACGGGTGAGTAATGTCTGGGAAACTGCCTGATGGAGGGGGA
TAACTACTGGAAACGGTAGCTAATACCGCATGACCTCGTAAGAGCAAAGTGGGGGACCT
TCGGGCCTCACGCCATCGGATGTGCCCAGATGGGATTAGCTAGTAGGTGGGGTAATGGC
TCACCTAGGCGACGATCCCTAGCTGGTCTGAGAGGATGACCAGCCACACTGGAACTGA
GACACGGTCCAGACTCCTACGGGAGGCAGCAGTGGGGAATATTGCACAATGGGCGCAA
GCCTGATGCAGCCATGCCGCGTGTGTGAAGAAGGCCTTCGGGTTGTAAAGCACTTTCAG
CGAGGAGGAAGGCAGTCGTGTTAATAGCACGGTTGATTGACGTTACTCGCAGAAGAAG
CACCGGCTAACTCCGTGCCAGCAGCCGCGGTAATACGGAGGGTGCAAGCGTTAATCGG
AATTACTGGGCGTAAAGCGCACGCAGGCGGTTTGTTAAGTCAGATGTGAAATCCCCGCG
CTTAACGTGGGAACTGCATTTGAAACTGGCAAGCTAGAGTCTTGTAGAGGGGGGTAGA
ATTCCAGGTGTAGCGGTGAAATGCGTAGAGATCTGGAGGAATACCGGTGGCGAAGGCG
GCCCCCTGGACAAAGACTGACGCTCAGGTGCGAAAGCGTGGGGAGCAAACAGGATTA
GATACCCTGGTAGTCCACGCTGTAAACGATGTCGACTTGGAGGTTGTGCCCTTGAGGCG
TGGCTTCCGGAGCTAACGCGTTAAGTCGACCGCCTGGGGAGTACGGCCGCAAGGTTAA
AACTCAAATGAATTGACGGGGGCCCGCACAAGCGGTGGAGCATGTGGTTTAATTCGATG
CAACGCGAAGAACCTTACCTACTCTTGACATCCACGGAATTTAGCAGAGATGCTTTAGT
GCCTTCGGGAACCGTGAGACAGGTGCTGCATGGCTGTCGTCAGCTCGTGTTGTGAAATG
TTGGGTTAAGTCCCGCAACGAGCGCAACCCTTATCCTTTGTTGCCAGCACGTAATGGTG
GGAACTCAAGGGAGACTGCCGGTGACAAACCGGAGGAAGGTGGGGATGACGTCAAGT
CATCATGGCCCTTACGAGTAGGGCTACACACGTGCTACAATGGCAGATACAAAGTGAAG
CGAACTCGCGAGAGCAAGCGGACCACATAAAGTCTGTCGTAGTCCGGATTGGAGTCTG
CAACTCGACTCCATGAAGTCGGAATCGCTAGTAATCGTAGATCAGAATGCTACGGTGAAT
ACGTTCCCGGGCCTTGTACACACCGCCCGTCACACCATGGGAGTGGGTTGCAAAAGAA

GTAGGTAGCTTAACCTTCGGGAGGGCGCTTACCACTTTGTGATTCATGACTGGGG
pattern 66
CGCTGGCGGCAGGCCTAACACATGCAAGTCGAGCGGCAGCGGAAAGTAGCTTGCTACT
TTGCCGGCGAGCGGCGGACGGGTGAGTAATGTCTGGGGATCTGCCTGATGGAGGGGGA
TAACTACTGGAAACGGTAGCTAATACCGCATGACCTCGAAAGAGCAAAGTGGGGGACC
TTCGGGCCTCACGCCATCGGATGAACCCAGATGGGATTAGCTAGTAGGTGGGGTAATGG
CTCACCTAGGCGACGATCCCTAGCTGGTCTGAGAGGATGACCAGCCACACTGGAACTG
AGACACGGTCCAGACTCCTACGGGAGGCAGCAGTGGGGAATATTGCACAATGGGCTCA
AGCCTGATGCAGCCATGCCGCGTGTGTGAAGAAGGCCTTCGGGTTGTAAAGCACTTTCA
GCGAGGAGGAAGGCATTGTGGTTAATAACCGCAGTGATTGACGTTACTCGCAGAAGAA
GCACCGGCTAACTCCGTGCCAGCAGCCGCGGTAATACGGAGGGTGCAAGCGTTAATCG
GAATTACTGGGCGTAAAGCGCACGCAGGCGGTTTGTTAAGTCAGATGTGAAATCCCCGC
GCTTAACGTGGGAACTGCATTTGAAACTGGCAAGCTAGAGTCTTGTAGAGGGGGGTAG
AATTCCAGGTGTAGCGGTGAAATGCGTAGAGATCTGGAGGAATACCGGTGGCGAAGGC
GGCCCCCTGGACAAAGACTGACGCTCAGGTGCGAAAGCGTGGGGAGCAAACAGGATT
AGATACCCTGGTAGTCCACGCTGTAAACGATGTCGACTTGGAGGTTGTGCCCTTGAGGC
GTGGCTTCCGGAGCTAACGCGTTAAGTCGACCGCCTGGGGAGTACGGCCGCAAGGTTA
AAACTCAAATGAATTGACGGGGGCCCGCACAAGCGGTGGAGCATGTGGTTTAATTCGAT
GCAACGCGAAGAACCTTACCTACTCTTGACATCCACAGAACTTAGCAGAGATGCTTCGG
TGCCTTCGGGAACTGTGAGACAGGTGCTGCATGGCTGTCGTCAGCTCGTGTTGTGAAAT
GTTGGGTTAAGTCCCGCAACGAGCGCAACCCTTATCCTTTGTTGCCAGCACGTAATGGT
GGGAACTCAAGGGAGACTGCCGGTGACAAACCGGAGGAAGGTGGGGATGACGTCAAG
TCATCATGGCCCTTACGAGTAGGGCTACACACGTGCTACAATGGCAGATACAAAGTGAA
GCGAACTCGCGAGAGCAAGCGGACCACATAAAGTCTGTCGTAGTCCGGATTGGAGTCT
GCAACTCGACTCCATGAAGTCGGAATCGCTAGTAATCGTAGATCAGAATGCTACGGTGA
ATACGTTCCCGGGCCTTGTACACACCGCCCGTCACACCATGGGAGTGGGTTGCAAAAG
AAGTAGGTAGCTTAACCTTCGGGAGGGCGCTTACCACTTTGTGATTCATGACTGGGG
pattern 67
CGCTGGCGGCAGGCCTAACACATGCAAGTCGAGCGGCAGCGGGAAGTAGTTTACTACT
TTGCCGGCGAGCGGCGGACGGGTGAGTAATGTCTGGGAAACTGCCTGATGGAGGGGGA
TAACTACTGGAAACGGTAGCTAATACCGCATGACCTCGTAAGAGCAAAGTGGGGGACCT
TCGGGCCTCACGCCATCGGATGTGCCCAGATGGGATTAGCTAGTAGGTGGGGTAATGGC
TCACCTAGGCGACGATCCCTAGCTGGTCTGAGAGGATGACCAGCCACACTGGAACTGA
GACACGGTCCAGACTCCTACGGGAGGCAGCAGTGGGGAATATTGCACAATGGGCGCAA
GCCTGATGCAGCCATGCCGCGTGTGTGAAGAAGGCCTTCGGGTTGTAAAGCACTTTCAG
CGAGGAGGAAGGCAGTCGTGTTAATAGCACGGTTGATTGACGTTACTCGCAGAAGAAG
CACCGGCTAACTCCGTGCCAGCAGCCGCGGTAATACGGAGGGTGCAAGCGTTAATCGG
AATTACTGGGCGTAAAGCGCACGCAGGCGGTTTGTTAAGTCAGATGTGAAATCCCCGCG
CTTAACGTGGGAACTGCATTTGAAACTGGCAAGCTAGAGTCTTGTAGAGGGGGGTAGA
ATTCCAGGTGTAGCGGTGAAATGCGTAGAGATCTGGAGGAATACCGGTGGCGAAGGCG
GCCCCCTGGACAAAGACTGACGCTCAGGTGCGAAAGCGTGGGGAGCAAACAGGATTA
GATACCCTGGTAGTCCACGCTGTAAACGATGTCGACTTGGAGGTTGTGCCCTTGAGGCG
TGGCTTCCGGAGCTAACGCGTTAAGTCGACCGCCTGGGGAGTACGGCCGCAAGGTTAA
AACTCAAATGAATTGACGGGGGCCCGCACAAGCGGTGGAGCATGTGGTTTAATTCGATG

CAACGCGAAGAACCTTACCTACTCTTGACATCCACAGAACTTAGCAGAGATGCTTCGGT
GCCTTCGGGAACTGTGAGACAGGTGCTGCATGGCTGTCGTCAGCTCGTGTTGTGAAATG
TTGGGTTAAGTCCCGCAACGAGCGCAACCCTTATCCTTTGTTGCCAGCACGTAATGGTG
GGAACTCAAGGGAGACTGCCGGTGACAAACCGGAGGAAGGTGGGGATGACGTCAAGT
CATCATGGCCCTTACGAGTAGGGCTACACACGTGCTACAATGGCAGATACAAAGTGAAG
CGAACTCGCGAGAGCAAGCGGACCACATAAAGTCTGTCGTAGTCCGGATTGGAGTCTG
CAACTCGACTCCATGAAGTCGGAATCGCTAGTAATCGTAGATCAGAATGCTACGGTGAAT
ACGTTCCCGGGCCTTGTACACACCGCCCGTCACACCATGGGAGTGGGTTGCAAAAGAA
GTAGGTAGCTTAACCTTCGGGAGGGCGCTTACCACTTTGTGATTCATGACTGGGG
pattern 68
CGCTGGCGGCAGGCCTAACACATGCAAGTCGAGCGGCAGCGGAAAGTAGCTTGCTACT
TTGCCGGCGAGCGGCGGACGGGTGAGTAATGTCTGGGAAACTGCCTGATGGAGGGGGA
TAACTACTGGAAACGGTAGCTAATACCGCATGACCTCGAAAGAGCAAAGTGGGGGACC
TTCGGGCCTCACGCCATCGGATGTGCCCAGATGGGATTAGCTAGTAGGTGGGGTAATGG
CTCACCTAGGCGACGATCCCTAGCTGGTCTGAGAGGATGACCAGCCACACTGGAACTG
AGACACGGTCCAGACTCCTACGGGAGGCAGCAGTGGGGAATATTGCACAATGGGCGCA
AGCCTGATGCAGCCATGCCGCGTGTGTGAAGAAGGCCTTCGGGTTGTAAAGCACTTTCA
GCGAGGAGGAAGGCATTTCACTTAATACGTGAAGTGATTGACGTTACTCGCAGAAGAA
GCACCGGCTAACTCCGTGCCAGCAGCCGCGGTAATACGGAGGGTGCAAGCGTTAATCG
GAATTACTGGGCGTAAAGCGCACGCAGGCGGTTTGTTAAGTCAGATGTGAAATCCCCGA
GCTTAACTTGGGAACTGCATTTGAAACTGGCAAGCTAGAGTCTTGTAGAGGGGGGTAG
AATTCCAGGTGTAGCGGTGAAATGCGTAGAGATCTGGAGGAATACCGGTGGCGAAGGC
GGCCCCCTGGACAAAGACTGACGCTCAGGTGCGAAAGCGTGGGGAGCAAACAGGATT
AGATACCCTGGTAGTCCACGCTGTAAACGATGTCGACTTGGAGGTTGTGCCCTTGAGGC
GTGGCTTCCGGAGCTAACGCGTTAAGTCGACCGCCTGGGGAGTACGGCCGCAAGGTTA
AAACTCAAATGAATTGACGGGGGCCCGCACAAGCGGTGGAGCATGTGGTTTAATTCGAT
GCAACGCGAAGAACCTTACCTACTCTTGACATCCACAGAACTTAGCAGAGATGCTTAGG
TGCCTTCGGGAACTGTGAGACAGGTGCTGCATGGCTGTCGTCAGCTCGTGTTGTGAAAT
GTTGGGTTAAGTCCCGCAACGAGCGCAACCCTTATCCTTTGTTGCCAGCACGTAATGGT
GGGAACTCAAAGGAGACTGCCGGTGATAAACCGGAGGAAGGTGGGGATGACGTCAAG
TCATCATGGCCCTTACGAGTAGGGCTACACACGTGCTACAATGGCAGATACAAAGTGAA
GCGAACTCGCGAGAGCAAGCGGACCACATAAAGTCTGTCGTAGTCCGGATTGGAGTCT
GCAACTCGACTCCATGAAGTCGGAATCGCTAGTAATCGTAGATCAGAATGCTACGGTGA
ATACGTTCCCGGGCCTTGTACACACCGCCCGTCACACCATGGGAGTGGGTTGCAAAAG
AAGTAGGTAGCTTAACCTTCGGGAGGGCGCTTACCACTTTGTGATTCATGACTGGGG
pattern 69
CGCTGGCGGCAGGCCTAACACATGCAAGTCGAGCGGCAGCGGGAAGTAGCTTGCTACT
TTGCCGGCGAGCGGCGGACGGGTGAGTAATGTCTGGGAAACTGCCTGATGGAGGGGGA
TAACTACTGGAAACGGTAGCTAATACCGCATGACCTCGAAAGAGCAAAGTGGGGGACC
TTCGGGCCTCACGCCATCGGATGTGCCCAGATGGGATTAGCTAGTAGGTGGGGTAATGG
CTCACCTAGGCGACGATCCCTAGCTGGTCTGAGAGGATGACCAGCCACACTGGAACTG
AGACACGGTCCAGACTCCTACGGGAGGCAGCAGTGGGGAATATTGCACAATGGGCGCA
AGCCTGATGCAGCCATGCCGCGTGTGTGAAGAAGGCCTTCGGGTTGTAAAGCACTTTCA
GCGAGGAGGAAGGCATTTCACTTAATACGTGAAGTGATTGACGTTACTCGCAGAAGAA

GCACCGGCTAACTCCGTGCCAGCAGCCGCGGTAATACGGAGGGTGCAAGCGTTAATCG
GAATTACTGGGCGTAAAGCGCACGCAGGCGGTTTGTTAAGTCAGATGTGAAATCCCCGA
GCTTAACTTGGGAACTGCATTTGAAACTGGCAAGCTAGAGTCTTGTAGAGGGGGGTAG
AATTCCAGGTGTAGCGGTGAAATGCGTAGAGATCTGGAGGAATACCGGTGGCGAAGGC
GGCCCCCTGGACAAAGACTGACGCTCAGGTGCGAAAGCGTGGGGAGCAAACAGGATT
AGATACCCTGGTAGTCCACGCTGTAAACGATGTCGACTTGGAGGTTGTGCCCTTGAGGC
GTGGCTTCCGGAGCTAACGCGTTAAGTCGACCGCCTGGGGAGTACGGCCGCAAGGTTA
AAACTCAAATGAATTGACGGGGGCCCGCACAAGCGGTGGAGCATGTGGTTTAATTCGAT
GCAACGCGAAGAACCTTACCTACTCTTGACATCCACAGAACTTAGCAGAGATGCTTAGG
TGCCTTCGGGAACTGTGAGACAGGTGCTGCATGGCTGTCGTCAGCTCGTGTTGTGAAAT
GTTGGGTTAAGTCCCGCAACGAGCGCAACCCTTATCCTTTGTTGCCAGCACGTAATGGT
GGGAACTCAAAGGAGACTGCCGGTGATAAACCGGAGGAAGGTGGGGATGACGTCAAG
TCATCATGGCCCTTACGAGTAGGGCTACACACGTGCTACAATGGCAGATACAAAGTGAA
GCGAACTCGCGAGAGCAAGCGGACCACATAAAGTCTGTCGTAGTCCGGATTGGAGTCT
GCAACTCGACTCCATGAAGTCGGAATCGCTAGTAATCGTAGATCAGAATGCTACGGTGA
ATACGTTCCCGGGCCTTGTACACACCGCCCGTCACACCATGGGAGTGGGTTGCAAAAG
AAGTAGGTAGCTTAACCTTCGGGAGGGCGCTTACCACTTTGTGATTCATGACTGGGG
pattern 70
CGCTGGCGGCAGGCCTAACACATGCAAGTCGAGCGGCAGCGGGAAGTAGCTTGCTACT
TTGCCGGCGAGCGGCGGACGGGTGAGTAATGTCTGGGAAACTGCCTGATGGAGGGGGA
TAACTACTGGAAACGGTAGCTAATACCGCATGACCTCGAAAGAGCAAAGTGGGGGACC
TTCGGGCCTCACGCCATCGGATGTGCCCAGATGGGATTAGCTAGTAGGTGGGGTAATGG
CTCACCTAGGCGACGATCCCTAGCTGGTCTGAGAGGATGACCAGCCACACTGGAACTG
AGACACGGTCCAGACTCCTACGGGAGGCAGCAGTGGGGAATATTGCACAATGGGCGCA
AGCCTGATGCAGCCATGCCGCGTGTGTGAAGAAGGCCTTCGGGTTGTAAAGCACTTTCA
GCGAGGAGGAAGGCATTTCACTTAATACGTGAGGTGATTGGCGTTACTCGCAGAAGAA
GCACCGGCTAACTCCGTGCCAGCAGCCGCGGTAATACGGAGGGTGCAAGCGTTAATCG
GAATTACTGGGCGTAAAGCGCACGCAGGCGGTTTGTTAAGTCAGATGTGAAATCCCCGA
GCTTAACTTGGGAACTGCATTTGAAACTGGCAAGCTAGAGTCTTGTAGAGGGGGGTAG
AATTCCAGGTGTAGCGGTGAAATGCGTAGAGATCTGGAGGAATACCGGTGGCGAAGGC
GGCCCCCTGGACAAAGACTGACGCTCAGGTGCGAAAGCGTGGGGAGCAAACAGGATT
AGATACCCTGGTAGTCCACGCTGTAAACGATGTCGACTTGGAGGTTGTGCCCTTGAGGC
GTGGCTTCCGGAGCTAACGCGTTAAGTCGACCGCCTGGGGAGTACGGCCGCAAGGTTA
AAACTCAAATGAATTGACGGGGGCCCGCACAAGCGGTGGAGCATGTGGTTTAATTCGAT
GCAACGCGAAGAACCTTACCTACTCTTGACATCCACAGAACTTAGCAGAGATGCTTAGG
TGCCTTCGGGAACTGTGAGACAGGTGCTGCATGGCTGTCGTCAGCTCGTGTTGTGAAAT
GTTGGGTTAAGTCCCGCAACGAGCGCAACCCTTATCCTTTGTTGCCAGCACGTAATGGT
GGGAACTCAAAGGAGACTGCCGGTGATAAACCGGAGGAAGGTGGGGATGACGTCAAG
TCATCATGGCCCTTACGAGTAGGGCTACACACGTGCTACAATGGCAGATACAAAGTGAA
GCGAACTCGCGAGAGCAAGCGGACCACATAAAGTCTGTCGTAGTCCGGATTGGAGTCT
GCAACTCGACTCCATGAAGTCGGAATCGCTAGTAATCGTAGATCAGAATGCTACGGTGA
ATACGTTCCCGGGCCTTGTACACACCGCCCGTCACACCATGGGAGTGGGTTGCAAAAG
AAGTAGGTAGCTTAACCTTCGGGAGGGCGCTTACCACTTTGTGATTCATGACTGGGG
pattern 71

CGCTGGCGGCAGGCCTAACACATGCAAGTCGAGCGGCAGCGGGAAGTAGCTTGCTACT
TTGCCGGCGAGCGGCGGACGGGTGAGTAATGTCTGGGAAACTGCCTGATGGAGGGGGA
TAACTACTGGAAACGGTAGCTAATACCGCATGACCTCGCAAGAGCAAAGTGGGGGACC
TTCGGGCCTCACGCCATCGGATGTGCCCAGATGGGATTAGCTAGTAGGTGGGGTAATGG
CTCACCTAGGCGACGATCCCTAGCTGGTCTGAGAGGATGACCAGCCACACTGGAACTG
AGACACGGTCCAGACTCCTACGGGAGGCAGCAGTGGGGAATATTGCACAATGGGCGCA
AGCCTGATGCAGCCATGCCGCGTGTGTGAAGAAGGCCTTCGGGTTGTAAAGCACTTTCA
GCGAGGAGGAAGGGGTTGAGTTTAATACGCTTAATCATTGACGTTACTCGCAGAAGAAG
CACCGGCTAACTCCGTGCCAGCAGCCGCGGTAATACGGAGGGTGCAAGCGTTAATCGG
AATTACTGGGCGTAAAGCGCACGCAGGCGGTTTGTTAAGTCAGATGTGAAATCCCCGCG
CTTAACGTGGGAACTGCATTTGAAACTGGCAAGCTAGAGTCTTGTAGAGGGGGGTAGA
ATTCCAGGTGTAGCGGTGAAATGCGTAGAGATCTGGAGGAATACCGGTGGCGAAGGCG
GCCCCCTGGACAAAGACTGACGCTCAGGTGCGAAAGCGTGGGGAGCAAACAGGATTA
GATACCCTGGTAGTCCACGCTGTAAACGATGTCGACTTGGAGGTTGTGCCCTTGAGGCG
TGGCTTCCGGAGCTAACGCGTTAAGTCGACCGCCTGGGGAGTACGGCCGCAAGGTTAA
AACTCAAATGAATTGACGGGGGCCCGCACAAGCGGTGGAGCATGTGGTTTAATTCGATG
CAACGCGAAGAACCTTACCTACTCTTGACATCCACAGAACTTAGCAGAGATGCTTCGGT
GCCTTCGGGAACTGTGAGACAGGTGCTGCATGGCTGTCGTCAGCTCGTGTTGTGAAATG
TTGGGTTAAGTCCCGCAACGAGCGCAACCCTTATCCTTTGTTGCCAGCACGTAATGGTG
GGAACTCAAAGGAGACTGCCGGTGATAAACCGGAGGAAGGTGGGGATGACGTCAAGT
CATCATGGCCCTTACGAGTAGGGCTACACACGTGCTACAATGGCAGATACAAAGTGAAG
CGAACTCGCGAGAGCAAGCGGACCACATAAAGTCTGTCGTAGTCCGGATTGGAGTCTG
CAACTCGACTCCATGAAGTCGGAATCGCTAGTAATCGTAGATCAGAATGCTACGGTGAAT
ACGTTCCCGGGCCTTGTACACACCGCCCGTCACACCATGGGAGTGGGTTGCAAAAGAA
GTAGGTAGCTTAACCTTCGGGAGGGCGCTTACCACTTTGTGATTCATGACTGGGG
pattern 72
CGCTGGCGGCAGGCCTAACACATGCAAGTCGAGCGGCAGCGGAAAGTAGCTTGCTACT
TTGCCGGCGAGCGGCGGACGGGTGAGTAATGTCTGGGAAACTGCCTGATGGAGGGGGA
TAACTACTGGAAACGGTAGCTAATACCGCATGACCTCGCAAGAGCAAAGTGGGGGACC
TTCGGGCCTCACGCCATCGGATGTGCCCAGATGGGATTAGCTAGTAGGTGGGGTAATGG
CTCACCTAGGCGACGATCCCTAGCTGGTCTGAGAGGATGACCAGCCACACTGGAACTG
AGACACGGTCCAGACTCCTACGGGAGGCAGCAGTGGGGAATATTGCACAATGGGCGCA
AGCCTGATGCAGCCATGCCGCGTGTGTGAAGAAGGCCTTCGGGTTGTAAAGCACTTTCA
GCGAGGAGGAAGGGGTTGAGTTTAATACGCTCAATCATTGACGTTACTCGCAGAAGAA
GCACCGGCTAACTCCGTGCCAGCAGCCGCGGTAATACGGAGGGTGCAAGCGTTAATCG
GAATTACTGGGCGTAAAGCGCACGCAGGCGGTTTGTTAAGTCAGATGTGAAATCCCCGC
GCTTAACGTGGGAACTGCATTTGAAACTGGCAAGCTAGAGTCTTGTAGAGGGGGGTAG
AATTCCAGGTGTAGCGGTGAAATGCGTAGAGATCTGGAGGAATACCGGTGGCGAAGGC
GGCCCCCTGGACAAAGACTGACGCTCAGGTGCGAAAGCGTGGGGAGCAAACAGGATT
AGATACCCTGGTAGTCCACGCTGTAAACGATGTCGACTTGGAGGTTGTGCCCTTGAGGC
GTGGCTTCCGGAGCTAACGCGTTAAGTCGACCGCCTGGGGAGTACGGCCGCAAGGTTA
AAACTCAAATGAATTGACGGGGGCCCGCACAAGCGGTGGAGCATGTGGTTTAATTCGAT
GCAACGCGAAGAACCTTACCTACTCTTGACATCCACAGAACTTAGCAGAGATGCTTAGG
TGCCTTCGGGAACTGTGAGACAGGTGCTGCATGGCTGTCGTCAGCTCGTGTTGTGAAAT

GTTGGGTTAAGTCCCGCAACGAGCGCAACCCTTATCCTTTGTTGCCAGCACGTCATGGT
GGGAACTCAAAGGAGACTGCCGGTGATAAACCGGAGGAAGGTGGGGATGACGTCAAG
TCATCATGGCCCTTACGAGTAGGGCTACACACGTGCTACAATGGCAGATACAAAGTGAA
GCGAACTCGCGAGAGCAAGCGGACCACATAAAGTCTGTCGTAGTCCGGATTGGAGTCT
GCAACTCGACTCCATGAAGTCGGAATCGCTAGTAATCGTAGATCAGAATGCTACGGTGA
ATACGTTCCCGGGCCTTGTACACACCGCCCGTCACACCATGGGAGTGGGTTGCAAAAG
AAGTAGGTAGCTTAACCTTCGGGAGGGCGCTTACCACTTTGTGATTCATGACTGGGG
pattern 73
CGCTGGCGGCAGGCCTAACACATGCAAGTCGAGCGGCAGCGGAAAGTAGCTTGCTACT
TTGCCGGCGAGCGGCGGACGGGTGAGTAATGTCTGGGAAACTGCCTGATGGAGGGGGA
TAACTACTGGAAACGGTAGCTAATACCGCATGACCTCGCAAGAGCAAAGTGGGGGACC
TTCGGGCCTCACGCCATCGGATGTGCCCAGATGGGATTAGCTAGTAGGTGGGGTAATGG
CTCACCTAGGCGACGATCCCTAGCTGGTCTGAGAGGATGACCAGCCACACTGGAACTG
AGACACGGTCCAGACTCCTACGGGAGGCAGCAGTGGGGAATATTGCACAATGGGCGCA
AGCCTGATGCAGCCATGCCGCGTGTGTGAAGAAGGCCTTCGGGTTGTAAAGCACTTTCA
GCGAGGAGGAAGGGGTTGAGTTTAATACGCTCAATCATTGACGTTACTCGCAGAAGAA
GCACCGGCTAACTCCGTGCCAGCAGCCGCGGTAATACGGAGGGTGCAAGCGTTAATCG
GAATTACTGGGCGTAAAGCGCACGCAGGCGGTTTGTTAAGTCAGATGTGAAATCCCCGC
GCTTAACGTGGGAACTGCATTTGAAACTGGCAAGCTAGAGTCTTGTAGAGGGGGGTAG
AATTCCAGGTGTAGCGGTGAAATGCGTAGAGATCTGGAGGAATACCGGTGGCGAAGGC
GGCCCCCTGGACAAAGACTGACGCTCAGGTGCGAAAGCGTGGGGAGCAAACAGGATT
AGATACCCTGGTAGTCCACGCTGTAAACGATGTCGACTTGGAGGTTGTGCCCTTGAGGC
GTGGCTTCCGGAGCTAACGCGTTAAGTCGACCGCCTGGGGAGTACGGCCGCAAGGTTA
AAACTCAAATGAATTGACGGGGGCCCGCACAAGCGGTGGAGCATGTGGTTTAATTCGAT
GCAACGCGAAGAACCTTACCTACTCTTGACATCCACAGAACTTAGCAGAGATGCTTCGG
TGCCTTCGGGAACTGTGAGACAGGTGCTGCATGGCTGTCGTCAGCTCGTGTTGTGAAAT
GTTGGGTTAAGTCCCGCAACGAGCGCAACCCTTATCCTTTGTTGCCAGCACGTAATGGT
GGGAACTCAAAGGAGACTGCCGGTGATAAACCGGAGGAAGGTGGGGATGACGTCAAG
TCATCATGGCCCTTACGAGTAGGGCTACACACGTGCTACAATGGCAGATACAAAGTGAA
GCGAACTCGCGAGAGCAAGCGGACCACATAAAGTCTGTCGTAGTCCGGATTGGAGTCT
GCAACTCGACTCCATGAAGTCGGAATCGCTAGTAATCGTAGATCAGAATGCTACGGTGA
ATACGTTCCCGGGCCTTGTACACACCGCCCGTCACACCATGGGAGTGGGTTGCAAAAG
AAGTAGGTAGCTTAACCTTCGGGAGGGCGCTTACCACTTTGTGATTCATGACTGGGG
pattern 74
CGCTGGCGGCAGGCCTAACACATGCAAGTCGAGCGGCAGCGGGAAGTAGCTTGCTACT
TTGCCGGCGAGCGGCGGACGGGTGAGTAATGTCTGGGAAACTGCCTGATGGAGGGGGA
TAACTACTGGAAACGGTAGCTAATACCGCATGACCTCGCAAGAGCAAAGTGGGGGACC
TTCGGGCCTCACGCCATCGGATGTGCCCAGATGGGATTAGCTAGTAGGTGGGGTAATGG
CTCACCTAGGCGACGATCCCTAGCTGGTCTGAGAGGATGACCAGCCACACTGGAACTG
AGACACGGTCCAGACTCCTACGGGAGGCAGCAGTGGGGAATATTGCACAATGGGCGCA
AGCCTGATGCAGCCATGCCGCGTGTGTGAAGAAGGCCTTCGGGTTGTAAAGCACTTTCA
GCGAGGAGGAAGGGGTTGAGTTTAATACGCTTAATCATTGACGTTACTCGCAGAAGAAG
CACCGGCTAACTCCGTGCCAGCAGCCGCGGTAATACGGAGGGTGCAAGCGTTAATCGG
AATTACTGGGCGTAAAGCGCACGCAGGCGGTTTGTTAAGTCAGATGTGAAATCCCCGCG

CTTAACGTGGGAACTGCATTTGAAACTGGCAAGCTAGAGTCTTGTAGAGGGGGGTAGA
ATTCCAGGTGTAGCGGTGAAATGCGTAGAGATCTGGAGGAATACCGGTGGCGAAGGCG
GCCCCCTGGACAAAGACTGACGCTCAGGTGCGAAAGCGTGGGGAGCAAACAGGATTA
GATACCCTGGTAGTCCACGCTGTAAACGATGTCGACTTGGAGGTTGTGCCCTTGAGGCG
TGGCTTCCGGAGCTAACGCGTTAAGTCGACCGCCTGGGGAGTACGGCCGCAAGGTTAA
AACTCAAATGAATTGACGGGGGCCCGCACAAGCGGTGGAGCATGTGGTTTAATTCGATG
CAACGCGAAGAACCTTACCTACTCTTGACATCCACGGAATTTAGCAGAGATGCTTTAGT
GCCTTCGGGAACTGTGAGACAGGTGCTGCATGGCTGTCGTCAGCTCGTGTTGTGAAATG
TTGGGTTAAGTCCCGCAACGAGCGCAACCCTTATCCTTTGTTGCCAGCACGTCATGGTG
GGAACTCAAAGGAGACTGCCGGTGATAAACCGGAGGAAGGTGGGGATGACGTCAAGT
CATCATGGCCCTTACGAGTAGGGCTACACACGTGCTACAATGGCAGATACAAAGTGAAG
CGAACTCGCGAGAGCAAGCGGACCACATAAAGTCTGTCGTAGTCCGGATTGGAGTCTG
CAACTCGACTCCATGAAGTCGGAATCGCTAGTAATCGTAGATCAGAATGCTACGGTGAAT
ACGTTCCCGGGCCTTGTACACACCGCCCGTCACACCATGGGAGTGGGTTGCAAAAGAA
GTAGGTAGCTTAACCTTCGGGAGGGCGCTTACCACTTTGTGATTCATGACTGGGG
pattern 75
CGCTGGCGGCAGGCCTAACACATGCAAGTCGAGCGGCAGCGGGAAGTAGTTTACTACT
TTGCCGGCGAGCGGCGGACGGGTGAGTAATGTCTGGGAAACTGCCTGATGGAGGGGGA
TAACTACTGGAAACGGTAGCTAATACCGCATGACCTCGCAAGAGCAAAGTGGGGGACC
TTCGGGCCTCACGCCATCGGATGTGCCCAGATGGGATTAGTTAGTAGGTGGGGTAATGG
CTCACCTAGGCGACGATCCCTAGCTGGTCTGAGAGGATGACCAGCCACACTGGAACTG
AGACACGGTCCAGACTCCTACGGGAGGCAGCAGTGGGGAATATTGCACAATGGGCGCA
AGCCTGATGCAGCCATGCCGCGTGTGTGAAGAAGGCCTTCGGGTTGTAAAGCACTTTCA
GCGAGGAGGAAGGCAGTCGTGTTAATAGCACGATTGATTGACGTTACTCGCAGAAGAA
GCACCGGCTAACTCCGTGCCAGCAGCCGCGGTAATACGGAGGGTGCAAGCGTTAATCG
GAATTACTGGGCGTAAAGCGCACGCAGGCGGTTTGTTAAGTCAGATGTGAAATCCCCGC
GCTTAACGTGGGAACTGCATTTGAAACTGGCAAGCTAGAGTCTTGTAGAGGGGGGTAG
AATTCCAGGTGTAGCGGTGAAATGCGTAGAGATCTGGAGGAATACCGGTGGCGAAGGC
GGCCCCCTGGACAAAGACTGACGCTCAGGTGCGAAAGCGTGGGGAGCAAACAGGATT
AGATACCCTGGTAGTCCACGCTGTAAACGATGTCGACTTGGAGGTTGTGCCCTTGAGGC
GTGGCTTCCGGAGCTAACGCGTTAAGTCGACCGCCTGGGGAGTACGGCCGCAAGGTTA
AAACTCAAATGAATTGACGGGGGCCCGCACAAGCGGTGGAGCATGTGGTTTAATTCGAT
GCAACGCGAAGAACCTTACCTACTCTTGACATCCACAGAACTTAGCAGAGATGCTTCGG
TGCCTTCGGGAACTGTGAGACAGGTGCTGCATGGCTGTCGTCAGCTCGTGTTGTGAAAT
GTTGGGTTAAGTCCCGCAACGAGCGCAACCCTTATCCTTTGTTGCCAGCACGTAATGGT
GGGAACTCAAGGGAGACTGCCGGTGACAAACCGGAGGAAGGTGGGGATGACGTCAAG
TCATCATGGCCCTTACGAGTAGGGCTACACACGTGCTACAATGGCAGATACAAAGTGAA
GCGAACTCGCGAGAGCAAGCGGACCACATAAAGTCTGTCGTAGTCCGGATTGGAGTCT
GCAACTCGACTCCATGAAGTCGGAATCGCTAGTAATCGTAGATCAGAATGCTACGGTGA
ATACGTTCCCGGGCCTTGTACACACCGCCCGTCACACCATGGGAGTGGGTTGCAAAAG
AAGTAGGTAGCTTAACCTTCGGGAGGGCGCTTACCACTTTGTGATTCATGACTGGGG
pattern 76
CGCTGGCGGCAGGCCTAACACATGCAAGTCGAGCGGCAGCGGGAAGTAGTTTACTACT
TTGCCGGCGAGCGGCGGACGGGTGAGTAATGTCTGGGGAACTGCCTGATGGAGGGGGA

TAACTACTGGAAACGGTAGCTAATACCGCATGACCTCGCAAGAGCAAAGTGGGGGACC
TTCGGGCCTCACGCCATCGGATGTGCCCAGATGGGATTAGCTAGTAGGTGGGGTAATGG
CTCACCTAGGCGACGATCCCTAGCTGGTCTGAGAGGATGACCAGCCACACTGGAACTG
AGACACGGTCCAGACTCCTACGGGAGGCAGCAGTGGGGAATATTGCACAATGGGCGCA
AGCCTGATGCAGCCATGCCGCGTGTGTGAAGAAGGCCTTCGGGTTGTAAAGCACTTTCA
GCGAGGAGGAAGGCAGTCGTGTTAATAGCACGATTGATTGACGTTACTCGCAGAAGAA
GCACCGGCTAACTCCGTGCCAGCAGCCGCGGTAATACGGAGGGTGCAAGCGTTAATCG
GAATTACTGGGCGTAAAGCGCACGCAGGCGGTTTGTTAAGTCAGATGTGAAATCCCCGC
GCTTAACGTGGGAACTGCATTTGAAACTGGCAAGCTAGAGTCTTGTAGAGGGGGGTAG
AATTCCAGGTGTAGCGGTGAAATGCGTAGAGATCTGGAGGAATACCGGTGGCGAAGGC
GGCCCCCTGGACAAAGACTGACGCTCAGGTGCGAAAGCGTGGGGAGCAAACAGGATT
AGATACCCTGGTAGTCCACGCTGTAAACGATGTCGACTTGGAGGTTGTGCCCTTGAGGC
GTGGCTTCCGGAGCTAACGCGTTAAGTCGACCGCCTGGGGAGTACGGCCGCAAGGTTA
AAACTCAAATGAATTGACGGGGGCCCGCACAAGCGGTGGAGCATGTGGTTTAATTCGAT
GCAACGCGAAGAACCTTACCTACTCTTGACATCCACGGAATTTAGCAGAGATGCTTTAG
TGCCTTCGGGAACCGTGAGACAGGTGCTGCATGGCTGTCGTCAGCTCGTGTTGTGAAAT
GTTGGGTTAAGTCCCGCAACGAGCGCAACCCTTATCCTTTGTTGCCAGCACGTAATGGT
GGGAACTCAAGGGAGACTGCCGGTGACAAACCGGAGGAAGGTGGGGATGACGTCAAG
TCATCATGGCCCTTACGAGTAGGGCTACACACGTGCTACAATGGCAGATACAAAGTGAA
GCGAACTCGCGAGAGCAAGCGGACCACATAAAGTCTGTCGTAGTCCGGATTGGAGTCT
GCAACTCGACTCCATGAAGTCGGAATCGCTAGTAATCGTAGATCAGAATGCTACGGTGA
ATACGTTCCCGGGCCTTGTACACACCGCCCGTCACACCATGGGAGTGGGTTGCAAAAG
AAGTAGGTAGCTTAACCTTCGGGAGGGCGCTTACCACTTTGTGATTCATGACTGGGG
pattern 77
CGCTGGCGGCAGGCCTAACACATGCAAGTCGAGCGGCAGTGGGAAGTAGTTTACTACT
TTGCCGGCGAGCGGCGGACGGGTGAGTAATGTCTGGGAAACTGCCTGATGGAGGGGGA
TAACTACTGGAAACGGTAGCTAATACCGCATAACGTCTTCGGACCAAAGTGGGGGACCT
TCGGGCCTCACGCCATCGGATGTGCCCAGATGGGATTAGCTAGTAGGTGGGGTAATGGC
TCACCTAGGCGACGATCCCTAGCTGGTCTGAGAGGATGACCAGCCACACTGGAACTGA
GACACGGTCCAGACTCCTACGGGAGGCAGCAGTGGGGAATATTGCACAATGGGCGCAA
GCCTGATGCAGCCATGCCGCGTGTGTGAAGAAGGCCTTCGGGTTGTAAAGCACTTTCAG
CGAGGAGGAAGGCATAAAGGTTAATAACCTTTGTGATTGACGTTACTCGCAGAAGAAG
CACCGGCTAACTCCGTGCCAGCAGCCGCGGTAATACGGAGGGTGCAAGCGTTAATCGG
AATTACTGGGCGTAAAGCGCACGCAGGCGGTTTGTTAAGTCAGATGTGAAATCCCCGCG
CTTAACGTGGGAACTGCATTTGAAACTGGCAAGCTAGAGTCTTGTAGAGGGGGGTAGA
ATTCCAGGTGTAGCGGTGAAATGCGTAGAGATCTGGAGGAATACCGGTGGCGAAGGCG
GCCCCCTGGACAAAGACTGACGCTCAGGTGCGAAAGCGTGGGGAGCAAACAGGATTA
GATACCCTGGTAGTCCACGCTGTAAACGATGTCGACTTGGAGGTTGTGCCCTTGAGGCG
TGGCTTCCGGAGCTAACGCGTTAAGTCGACCGCCTGGGGAGTACGGCCGCAAGGTTAA
AACTCAAATGAATTGACGGGGGCCCGCACAAGCGGTGGAGCATGTGGTTTAATTCGATG
CAACGCGAAGAACCTTACCTACTCTTGACATCCACGGAATTTAGCAGAGATGCTTTAGT
GCCTTCGGGAACCGTGAGACAGGTGCTGCATGGCTGTCGTCAGCTCGTGTTGTGAAATG
TTGGGTTAAGTCCCGCAACGAGCGCAACCCTTATCCTTTGTTGCCAGCACGTGATGGTG
GGAACTCAAAGGAGACTGCCGGTGATAAACCGGAGGAAGGTGGGGATGACGTCAAGT

CATCATGGCCCTTACGAGTAGGGCTACACACGTGCTACAATGGCAGATACAAAGTGAAG
CGAACTCGCGAGAGCAAGCGGACCACATAAAGTCTGTCGTAGTCCGGATTGGAGTCTG
CAACTCGACTCCATGAAGTCGGAATCGCTAGTAATCGTAGATCAGAATGCTACGGTGAAT
ACGTTCCCGGGCCTTGTACACACCGCCCGTCACACCATGGGAGTGGGTTGCAAAAGAA
GTAGGTAGCTTAACCTTCGGGAGGGCGCTTACCACTTTGTGATTCATGACTGGGG
pattern 78
CGCTGGCGGCAGGCCTAACACATGCAAGTCGAGCGGCAGTGGGAAGTAGTTTACTACT
TTGCCGGCGAGCGGCGGACGGGTGAGTAATGTCTGGGAAACTGCCTGATGGAGGGGGA
TAACTACTGGAAACGGTAGCTAATACCGCATAACGTCTTCGGACCAAAGTGGGGGACCT
TCGGGCCTCACGCCATCGGATGTGCCCAGATGGGATTAGCTAGTAGGTGGGGTAATGGC
TCACCTAGGCGACGATCCCTAGCTGGTCTGAGAGGATGACCAGCCACACTGGAACTGA
GACACGGTCCAGACTCCTACGGGAGGCAGCAGTGGGGAATATTGCACAATGGGCGCAA
GCCTGATGCAGCCATGCCGCGTGTGTGAAGAAGGCCTTCGGGTTGTAAAGCACTTTCAG
CGAGGAGGAAGGCATAAAGGTTAATAACCTTTGTGATTGACGTTACTCGCAGAAGAAG
CACCGGCTAACTCCGTGCCAGCAGCCGCGGTAATACGGAGGGTGCAAGCGTTAATCGG
AATTACTGGGCGTAAAGCGCACGCAGGCGGTTTGTTAAGTCAGATGTGAAATCCCCGCG
CTTAACGTGGGAACTGCATTTGAAACTGGCAAGCTAGAGTCTTGTAGAGGGGGGTAGA
ATTCCAGGTGTAGCGGTGAAATGCGTAGAGATCTGGAGGAATACCGGTGGCGAAGGCG
GCCCCCTGGACAAAGACTGACGCTCAGGTGCGAAAGTGTGGGGAGCAAACAGGATTA
GATACCCTGGTAGTCCACGCTGTAAACGATGTCGACTTGGAGGTTGTGCCCTTGAGGCG
TGGCTTCCGGAGCTAACGCGTTAAGTCGACCGCCTGGGGAGTACGGCCGCAAGGTTAA
AACTCAAATGAATTGACGGGGGCCCGCACAAGCGGTGGAGCATGTGGTTTAATTCGATG
CAACGCGAAGAACCTTACCTACTCTTGACATCCACGGAATTTAGCAGAGATGCTTTAGT
GCCTTCGGGAACCGTGAGACAGGTGCTGCATGGCTGTCGTCAGCTCGTGTTGTGAAATG
TTGGGTTAAGTCCCGCAACGAGCGCAACCCTTATCCTTTGTTGCCAGCACGTGATGGTG
GGAACTCAAAGGAGACTGCCGGTGATAAACCGGAGGAAGGTGGGGATGACGTCAAGT
CATCATGGCCCTTACGAGTAGGGCTACACACGTGCTACAATGGCAGATACAAAGTGAAG
CGAACTCGCGAGAGCAAGCGGACCACATAAAGTCTGTCGTAGTCCGGATTGGAGTCTG
CAACTCGACTCCATGAAGTCGGAATCGCTAGTAATCGTAGATCAGAATGCTACGGTGAAT
ACGTTCCCGGGCCTTGTACACACCGCCCGTCACACCATGGGAGTGGGTTGCAAAAGAA
GTAGGTAGCTTAACCTTCGGGAGGGCGCTTACCACTTTGTGATTCATGACTGGGG
pattern 79
CGCTGGCGGCAGGCCTAACACATGCAAGTCGAGCGGCAGCGGGAAGTAGTTTACTACT
TTGCCGGCGAGCGGCGGACGGGTGAGTAATGTCTGGGAAACTGCCTGATGGAGGGGGA
TAACTACTGGAAACGGTAGCTAATACCGCATGACCTCGCAAGAGCAAAGTGGGGGACC
TTCGGGCCTCACGCCATCGGATGTGCCCAGATGGGATTAGCTAGTAGGTGGGGTAATGG
CTCACCTAGGCGACGATCCCTAGCTGGTCTGAGAGGATGACCAGCCACACTGGAACTG
AGACACGGTCCAGACTCCTACGGGAGGCAGCAGTGGGGAATATTGCACAATGGGCGCA
AGCCTGATGCAGCCATGCCGCGTGTGTGAAGAAGGCCTTCGGGTTGTAAAGCACTTTCA
GCGAGGAGGAAGGCAGTCGTGTTAATAGCACTGTTCATTGACGTTACTCGCAGAAGAA
GCACCGGCTAACTCCGTGCCAGCAGCCGCGGTAATACGGAGGGTGCAAGCGTTAATCG
GAATTACTGGGCGTAAAGCGCACGCAGGCGGTTTGTTAAGTCAGATGTGAAATCCCCGC
GCTTAACGTGGGAACTGCATTTGAAACTGGCAAGCTAGAGTCTTGTAGAGGGGGGTAG
AATTCCAGGTGTAGCGGTGAAATGCGTAGAGATCTGGAGGAATACCGGTGGCGAAGGC

GGCCCCCTGGACAAAGACTGACGCTCAGGTGCGAAAGCGTGGGGAGCAAACAGGATT
AGATACCCTGGTAGTCCACGCTGTAAACGATGTCGACTTGGAGGTTGTGCCCTTGAGGC
GTGGCTTCCGGAGCTAACGCGTTAAGTCGACCGCCTGGGGAGTACGGCCGCAAGGTTA
AAACTCAAATGAATTGACGGGGGCCCGCACAAGCGGTGGAGCATGTGGTTTAATTCGAT
GCAACGCGAAGAACCTTACCTACTCTTGACATCCACAGAACTTAGCAGAGATGCTTCGG
TGCCTTCGGGAACTGTGAGACAGGTGCTGCATGGCTGTCGTCAGCTCGTGTTGTGAAAT
GTTGGGTTAAGTCCCGCAACGAGCGCAACCCTTATCCTTTGTTGCCAGCACGTAATGGT
GGGAACTCAAGGGAGACTGCCGGTGACAAACCGGAGGAAGGTGGGGATGACGTCAAG
TCATCATGGCCCTTACGAGTAGGGCTACACACGTGCTACAATGGCAGATACAAAGTGAA
GCGAACTCGCGAGAGCAAGCGGACCACATAAAGTCTGTCGTAGTCCGGATTGGAGTCT
GCAACTCGACTCCATGAAGTCGGAATCGCTAGTAATCGTAGATCAGAATGCTACGGTGA
ATACGTTCCCGGGCCTTGTACACACCGCCCGTCACACCATGGGAGTGGGTTGCAAAAG
AAGTAGGTAGCTTAACCTTCGGGAGGGCGCTTACCACTTTGTGATTCATGACTGGGG
pattern 80
CGCTGGCGGCAGGCCTAACACATGCAAGTCGAGCGGCAGCGGAAAGTAGCTTGCTACT
TTGCCGGCGAGCGGCGGACGGGTGAGTAATGTCTGGGAAATTGCCTGATGGAGGGGGA
TAACTACTGGAAACGGTAGCTAATACCGCATGACCTCGAAAGAGCAAAGTGGGGGACC
TTCGGGCCTCACGCCATCGGATGTGCCCAGATGGGATTAGCTAGTAGGTGGGGTAATGG
CTCACCTAGGCGACGATCCCTAGCTGGTCTGAGAGGATGACCAGCCACACTGGAACTG
AGACACGGTCCAGACTCCTACGGGAGGCAGCAGTGGGGAATATTGCACAATGGGCGCA
AGCCTGATGCAGCCATGCCGCGTGTGTGAAGAAGGCCTTCGGGTTGTAAAGCACTTTCA
GCGAGGAGGAAGGCATTTCACTTAATACGTGAAGTGATTGACGTTACTCGCAGAAGAA
GCACCGGCTAACTCCGTGCCAGCAGCCGCGGTAATACGGAGGGTGCAAGCGTTAATCG
GAATTACTGGGCGTAAAGCGCACGCAGGCGGTTTGTTAAGTCAGATGTGAAATCCCCGA
GCTTAACTTGGGAACTGCATTTGAAACTGGCAAGCTAGAGTCTTGTAGAGGGGGGTAG
AATTCCAGGTGTAGCGGTGAAATGCGTAGAGATCTGGAGGAATACCGGTGGCGAAGGC
GGCCCCCTGGACAAAGACTGACGCTCAGGTGCGAAAGCGTGGGGAGCAAACAGGATT
AGATACCCTGGTAGTCCACGCTGTAAACGATGTCGACTTGGAGGTTGTGCCCTTGAGGC
GTGGCTTCCGGAGCTAACGCGTTAAGTCGACCGCCTGGGGAGTACGGCCGCAAGGTTA
AAACTCAAATGAATTGACGGGGGCCCGCACAAGCGGTGGAGCATGTGGTTTAATTCGAT
GCAACGCGAAGAACCTTACCTACTCTTGACATCCACAGAACTTAGCAGAGATGCTTAGG
TGCCTTCGGGAACTGTGAGACAGGTGCTGCATGGCTGTCGTCAGCTCGTGTTGTGAAAT
GTTGGGTTAAGTCCCGCAACGAGCGCAACCCTTATCCTTTGTTGCCAGCACGTAATGGT
GGGAACTCAAAGGAGACTGCCGGTGATAAACCGGAGGAAGGTGGGGATGACGTCAAG
TCATCATGGCCCTTACGAGTAGGGCTACACACGTGCTACAATGGCAGATACAAAGTGAA
GCGAACTCGCGAGAGCAAGCGGACCACATAAAGTCTGTCGTAGTCCGGATTGGAGTCT
GCAACTCGACTCCATGAAGTCGGAATCGCTAGTAATCGTAGATCAGAATGCTACGGTGA
ATACGTTCCCGGGCCTTGTACACACCGCCCGTCACACCATGGGAGTGGGTTGCAAAAG
AAGTAGGTAGCTTAACCTTCGGGAGGGCGCTTACCACTTTGTGATTCATGACTGGGG
pattern 81
CGCTGGCGGCAGGCCTAACACATGCAAGTCGAGCGGCAGCGGAAAGTAGCTTGCTACT
TTGCCGGCGAGCGGCGGACGGGTGAGTAATGTCTGGGGATCTGCCTGATGGAGGGGGA
TAACTACTGGAAACGGTAGCTAATACCGCATGACCTCGAAAGAGCAAAGTGGGGGACC
TTCGGGCCTCACGCCATCGGATGAACCCAGATGGGATTAGCTAGTAGGTGAGGTAATGG

CTCACCTAGGCGACGATCCCTAGCTGGTCTGAGAGGATGACCAGCCACACTGGAACTG
AGACACGGTCCAGACTCCTACGGGAGGCAGCAGTGGGGAATATTGCACAATGGGCGCA
AGCCTGATGCAGCCATGCCGCGTGTGTGAAGAAGGCCTTCGGGTTGTAAAGCACTTTCA
GCGAGGAGGAAGGCATTGTGGTTAATAACCACAGTGATTGACGTTACTCGCAGAAGAA
GCACCGGCTAACTCCGTGCCAGCAGCCGCGGTAATACGGAGGGTGCAAGCGTTAATCG
GAATTACTGGGCGTAAAGCGCACGCAGGCGGTTTGTTAAGTCAGATGTGAAATCCCCGC
GCTTAACGTGGGAACTGCATTTGAAACTGGCAAGCTAGAGTCTTGTAGAGGGGGGTAG
AATTCCAGGTGTAGCGGTGAAATGCGTAGAGATCTGGAGGAATACCGGTGGCGAAGGC
GGCCCCCTGGACAAAGACTGACGCTCAGGTGCGAAAGCGTGGGGAGCAAACAGGATT
AGATACCCTGGTAGTCCACGCTGTAAACGATGTCGACTTGGAGGTTGTGCCCTTGAGGC
GTGGCTTCCGGAGCTAACGCGTTAAGTCGACCGCCTGGGGAGTACGGCCGCAAGGTTA
AAACTCAAATGAATTGACGGGGGCCCGCACAAGCGGTGGAGCATGTGGTTTAATTCGAT
GCAACGCGAAGAACCTTACCTACTCTTGACATCCACGGAATTTAGCAGAGATGCTTTAG
TGCCTTCGGGAACCGTGAGACAGGTGCTGCATGGCTGTCGTCAGCTCGTGTTGTGAAAT
GTTGGGTTAAGTCCCGCAACGAGCGCAACCCTTATCCTTTGTTGCCAGCACGTAATGGT
GGGAACTCAAGGGAGACTGCCGGTGACAAACCGGAGGAAGGTGGGGATGACGTCAAG
TCATCATGGCCCTTACGAGTAGGGCTACACACGTGCTACAATGGCAGATACAAAGTGAA
GCGAACTCGCGAGAGCAAGCGGACCACATAAAGTCTGTCGTAGTCCGGATTGGAGTCT
GCAACTCGACTCCATGAAGTCGGAATCGCTAGTAATCGTAGATCAGAATGCTACGGTGA
ATACGTTCCCGGGCCTTGTACACACCGCCCGTCACACCATGGGAGTGGGTTGCAAAAG
AAGTAGGTAGCTTAACCTTCGGGAGGGCGCTTACCACTTTGTGATTCATGACTGGGG
pattern 82
CGCTGGCGGCAGGCCTAACACATGCAAGTCGAGCGGCAGCGGAAAGTAGCTTGCTACT
TTGCCGGCGAGCGGCGGACGGGTGAGTAATGTCTGGGAAACTGCCTGATGGAGGGGGA
TAACTACTGGAAACGGTAGCTAATACCGCATGACCTCGAAAGAGCAAAGTGGGGGACC
TTCGGGCCTCACGCCATCGGATGTGCCCAGATGGGATTAGCTAGTAGGTGAGGTAATGG
CTCACCTAGGCGACGATCCCTAGCTGGTCTGAGAGGATGACCAGCCACACTGGAACTG
AGACACGGTCCAGACTCCTACGGGAGGCAGCAGTGGGGAATATTGCACAATGGGCGCA
AGCCTGATGCAGCCATGCCGCGTGTGTGAAGAAGGCCTTCGGGTTGTAAAGCACTTTCA
GCGAGGAGGAAGGCATTTCACTTAATACGTGAAGTGATTGACGTTACTCGCAGAAGAA
GCACCGGCTAACTCCGTGCCAGCAGCCGCGGTAATACGGAGGGTGCAAGCGTTAATCG
GAATTACTGGGCGTAAAGCGCACGCAGGCGGTTTGTTAAGTCAGATGTGAAATCCCCGA
GCTTAACTTGGGAACTGCATTTGAAACTGGCAAGCTAGAGTCTTGTAGAGGGGGGTAG
AATTCCAGGTGTAGCGGTGAAATGCGTAGAGATCTGGAGGAATACCGGTGGCGAAGGC
GGCCCCCTGGACAAAGACTGACGCTCAGGTGCGAAAGCGTGGGGAGCAAACAGGATT
AGATACCCTGGTAGTCCACGCTGTAAACGATGTCGACTTGGAGGTTGTGCCCTTGAGGC
GTGGCTTCCGGAGCTAACGCGTTAAGTCGACCGCCTGGGGAGTACGGCCGCAAGGTTA
AAACTCAAATGAATTGACGGGGGCCCGCACAAGCGGTGGAGCATGTGGTTTAATTCGAT
GCAACGCGAAGAACCTTACCTACTCTTGACATCCACAGAACTGAGCAGAGATGCTTAG
GTGCCTTCGGGAACTGTGAGACAGGTGCTGCATGGCTGTCGTCAGCTCGTGTTGTGAAA
TGTTGGGTTAAGTCCCGCAACGAGCGCAACCCTTATCCTTTGTTGCCAGCACGTGATGG
TGGGAACTCAAAGGAGACTGCCGGTGATAAACCGGAGGAAGGTGGGGATGACGTCAA
GTCATCATGGCCCTTACGAGTAGGGCTACACACGTGCTACAATGGCAGATACAAAGTGA
AGCGAACTCGCGAGAGCAAGCGGACCACATAAAGTCTGTCGTAGTCCGGATTGGAGTC

TGCAACTCGACTCCATGAAGTCGGAATCGCTAGTAATCGTAGATCAGAATGCTACGGTG
AATACGTTCCCGGGCCTTGTACACACCGCCCGTCACACCATGGGAGTGGGTTGCAAAA
GAAGTAGGTAGCTTAACCTTCGGGAGGGCGCTTACCACTTTGTGATTCATGACTGGGG
pattern 83
CGCTGGCGGCAGGCCTAACACATGCAAGTCGAGCGGCAGCGGAAAGTAGCTTGCTACT
TTGCCGGCGAGCGGCGGACGGGTGAGTAATGTCTGGGGATCTGCCTGATGGAGGGGGA
TAACTACTGGAAACGGTAGCTAATACCGCATGACCTCGAAAGAGCAAAGTGGGGGACC
TTCGGGCCTCACGCCATCGGATGAACCCAGATGGGATTAGCTAGTAGGTGGGGTAATGG
CTCACCTAGGCGACGATCCCTAGCTGGTCTGAGAGGATGACCAGCCACACTGGAACTG
AGACACGGTCCAGACTCCTACGGGAGGCAGCAGTGGGGAATATTGCACAATGGGCGCA
AGCCTGATGCAGCCATGCCGCGTGTGTGAAGAAGGCCTTCGGGTTGTAAAGCACTTTCA
GCGAGGAGGAAGGCATTGTGGTTAATAACCACAGTGATTGACGTTACTCGCAGAAGAA
GCACCGGCTAACTCCGTGCCAGCAGCCGCGGTAATACGGAGGGTGCAAGCGTTAATCG
GAATTACTGGGCGTAAAGCGCACGCAGGCGGTTTGTTAAGTCAGATGTGAAATCCCCGC
GCTTAACGTGGGAACTGCATTTGAAACTGGCAAGCTAGAGTCTTGTAGAGGGGGGTAG
AATTCCAGGTGTAGCGGTGAAATGCGTAGAGATCTGGAGGAATACCGGTGGCGAAGGC
GGCCCCCTGGACAAAGACTGACGCTCAGGTGCGAAAGCGTGGGGAGCAAACAGGATT
AGATACCCTGGTAGTCCACGCTGTAAACGATGTCGACTTGGAGGTTGTGCCCTTGAGGC
GTGGCTTCCGGAGCTAACGCGTTAAGTCGACCGCCTGGGGAGTACGGCCGCAAGGTTA
AAACTCAAATGAATTGACGGGGGCCCGCACAAGCGGTGGAGCATGTGGTTTAATTCGAT
GCAACGCGAAGAACCTTACCTACTCTTGACATCCACGGAATTTAGCAGAGATGCTTTAG
TGCCTTCGGGAACCGTGAGACAGGTGCTGCATGGCTGTCGTCAGCTCGTGTTGTGAAAT
GTTGGGTTAAGTCCCGCAACGAGCGCAACCCTTATCCTTTGTTGCCAGCACGTAATGGT
GGGAACTCAAGGGAGACTGCCGGTGACAAACCGGAGGAAGGTGGGGATGACGTCAAG
TCATCATGGCCCTTACGAGTAGGGCTACACACGTGCTACAATGGCAGATACAAAGTGAA
GCGAACTCGCGAGAGCAAGCGGACCACATAAAGTCTGTCGTAGTCCGGATTGGAGTCT
GCAACTCGACTCCATGAAGTCGGAATCGCTAGTAATCGTAGATCAGAATGCTACGGTGA
ATACGTTCCCGGGCCTTGTACACACCGCCCGTCACACCATGGGAGTGGGTTGCAAAAG
AAGTAGGTAGCTTAACCTTCGGGAGGGCGCTTACCACTTTGTGATTCATGACTGGGG
pattern 84
CGCTGGCGGCAGGCCTAACACATGCAAGTCGAGCGGCAGCGGAAAGTAGCTTGCTACT
TTGCCGGCGAGCGGCGGACGGGTGAGTAATGTCTGGGGATCTGCCTGATGGAGGGGGA
TAACTACTGGAAACGGTAGCTAATACCGCATGACCTCGAAAGAGCAAAGTGGGGGACC
TTCGGGCCTCACGCCATCGGATGAACCCAGATGGGATTAGCTAGTAGGTGGGGTAATGG
CTCACCTAGGCGACGATCCCTAGCTGGTCTGAGAGGATGACCAGCCACACTGGAACTG
AGACACGGTCCAGACTCCTACGGGAGGCAGCAGTGGGGAATATTGCACAATGGGCGCA
AGCCTGATGCAGCCATGCCGCGTGTGTGAAGAAGGCCTTCGGGTTGTAAAGCACTTTCA
GCGAGGAGGAAGGCATTGTGGTTAATAACCGCAGTGATTGACGTTACTCGCAGAAGAA
GCACCGGCTAACTCCGTGCCAGCAGCCGCGGTAATACGGAGGGTGCAAGCGTTAATCG
GAATTACTGGGCGTAAAGCGCACGCAGGCGGTTTGTTAAGTCAGATGTGAAATCCCCGC
GCTTAACGTGGGAACTGCATTTGAAACTGGCAAGCTAGAGTCTTGTAGAGGGGGGTAG
AATTCCAGGTGTAGCGGTGAAATGCGTAGAGATCTGGAGGAATACCGGTGGCGAAGGC
GGCCCCCTGGACAAAGACTGACGCTCAGGTGCGAAAGCGTGGGGAGCAAACAGGATT
AGATACCCTGGTAGTCCACGCTGTAAACGATGTCGACTTGGAGGTTGTGCCCTTGAGGT

GTGGCTTCCGGAGCTAACGCGTTAAGTCGACCGCCTGGGGAGTACGGCCGCAAGGTTA
AAACTCAAATGAATTGACGGGGGCCCGCACAAGCGGTGGAGCATGTGGTTTAATTCGAT
GCAACGCGAAGAACCTTACCTACTCTTGACATCCACGGAATTTAGCAGAGATGCTTTAG
TGCCTTCGGGAACTGTGAGACAGGTGCTGCATGGCTGTCGTCAGCTCGTGTTGTGAAAT
GTTGGGTTAAGTCCCGCAACGAGCGCAACCCTTATCCTTTGTTGCCAGCACGTAATGGT
GGGAACTCAAGGGAGACTGCCGGTGACAAACCGGAGGAAGGTGGGGATGACGTCAAG
TCATCATGGCCCTTACGAGTAGGGCTACACACGTGCTACAATGGCAGATACAAAGTGAA
GCGAACTCGCGAGAGCAAGCGGACCACATAAAGTCTGTCGTAGTCCGGATTGGAGTCT
GCAACTCGACTCCATGAAGTCGGAATCGCTAGTAATCGTAGATCAGAATGCTACGGTGA
ATACGTTCCCGGGCCTTGTACACACCGCCCGTCACACCATGGGAGTGGGTTGCAAAAG
AAGTAGGTAGCTTAACCTTCGGGAGGGCGCTTACCACTTTGTGATTCATGACTGGGG
pattern 85
CGCTGGCGGCAGGCCTAACACATGCAAGTCGAGCGGCAGCGGAAAGTAGCTTGCTACT
TTGCCGGCGAGCGGCGGACGGGTGAGTAATGTCTGGGGATCTGCCTGATGGAGGGGGA
TAACTACTGGAAACGGTAGCTAATACCGCATGACCTCGAAAGAGCAAAGTGGGGGACC
TTCGGGCCTCACGCCATCGGATGAACCCAGATGGGATTAGCTAGTAGGTGAGGTAATGG
CTCACCTAGGCGACGATCCCTAGCTGGTCTGAGAGGATGACCAGCCACACTGGAACTG
AGACACGGTCCAGACTCCTACGGGAGGCAGCAGTGGGGAATATTGCACAATGGGCGCA
AGCCTGATGCAGCCATGCCGCGTGTGTGAAGAAGGCCTTCGGGTTGTAAAGCACTTTCA
GCGAGGAGGAAGGCATTGTGGTTAATAACCGCAGTGATTGACGTTACTCGCAGAAGAA
GCACCGGCTAACTCCGTGCCAGCAGCCGCGGTAATACGGAGGGTGCAAGCGTTAATCG
GAATTACTGGGCGTAAAGCGCACGCAGGCGGTTTGTTAAGTCAGATGTGAAATCCCCGC
GCTTAACGTGGGAACTGCATTTGAAACTGGCAAGCTAGAGTCTTGTAGAGGGGGGTGG
AATTCCAGGTGTAGCGGTGAAATGCGTAGAGATCTGGAGGAATACCGGTGGCGAAGGC
GGCCCCCTGGACAAAGACTGACGCTCAGGTGCGAAAGCGTGGGGAGCAAACAGGATT
AGATACCCTGGTAGTCCACGCTGTAAACGATGTCGACTTGGAGGTTGTGCCCTTGAGGC
GTGGCTTCCGGAGCTAACGCGTTAAGTCGACCGCCTGGGGAGTACGGCCGCAAGGTTA
AAACTCAAATGAATTGACGGGGGCCCGCACAAGCGGTGGAGCATGTGGTTTAATTCGAT
GCAACGCGAAGAACCTTACCTACTCTTGACATCCACAGAACTTAGCAGAGATGCTTCGG
TGCCTTCGGGAACTGTGAGACAGGTGCTGCATGGCTGTCGTCAGCTCGTGTTGTGAAAT
GTTGGGTTAAGTCCCGCAACGAGCGCAACCCTTATCCTTTGTTGCCAGCACGTAATGGT
GGGAACTCAAGGGAGACTGCCGGTGACAAACCGGAGGAAGGTGGGGATGACGTCAAG
TCATCATGGCCCTTACGAGTAGGGCTACACACGTGCTACAATGGCAGATACAAAGTGAA
GCGAACTCGCGAGAGCAAGCGGACCACATAAAGTCTGTCGTAGTCCGGATTGGAGTCT
GCAACTCGACTCCATGAAGTCGGAATCGCTAGTAATCGTAGATCAGAATGCTACGGTGA
ATACGTTCCCGGGCCTTGTACACACCGCCCGTCACACCATGGGAGTGGGTTGCAAAAG
AAGTAGGTAGCTTAACCTTCGGGAGGGCGCTTACCACTTTGTGATTCATGACTGGGG
pattern 86
CGCTGGCGGCAGGCCTAACACATGCAAGTCGAGCGGCAGCGGAAAGTAGCTTGCTACT
TTGCCGGCGAGCGGCGGACGGGTGAGTAATGTCTGGGAAACTGCCTGATGGAGGGGGA
TAACTACTGGAAACGGTAGCTAATACCGCATGACCTCGAAAGAGCAAAGTGGGGGACC
TTCGGGCCTCACGCCATCGGATGTGCCCAGATGGGATTAGCTAGTAGGTGAGGTAATGG
CTCACCTAGGCGACGATCCCTAGCTGGTCTGAGAGGATGACCAGCCACACTGGAACTG
AGACACGGTCCAGACTCCTACGGGAGGCAGCAGTGGGGAATATTGCACAATGGGCGCA

AGCCTGATGCAGCCATGCCGCGTGTGTGAAGAAGGCCTTCGGGTTGTAAAGCACTTTCA
GCGAGGAGGAAGGCATTTCACTTAATACGTGAAGTGATTGACGTTACTCGCAGAAGAA
GCACCGGCTAACTCCGTGCCAGCAGCCGCGGTAATACGGAGGGTGCAAGCGTTAATCG
GAATTACTGGGCGTAAAGCGCACGCAGGCGGTTTGTTAAGTCAGATGTGAAATCCCCGA
GCTTAACTTGGGAACTGCATTTGAAACTGGCAAGCTAGAGTCTTGTAGAGGGGGGTAG
AATTCCAGGTGTAGCGGTGAAATGCGTAGAGATCTGGAGGAATACCGGTGGCGAAGGC
GGCCCCCTGGACAAAGACTGACGCTCAGGTGCGAAAGCGTGGGGAGCAAACAGGATT
AGATACCCTGGTAGTCCACGCTGTAAACGATGTCGACTTGGAGGTTGTGCCCTTGAGGC
GTGGCTTCCGGAGCTAACGCGTTAAGTCGACCGCCTGGGGAGTACGGCCGCAAGGTTA
AAACTCAAATGAATTGACGGGGGCCCGCACAAGCGGTGGAGCATGTGGTTTAATTCGAT
GCAACGCGAAGAACCTTACCTACTCTTGACATCCACAGAACTTAGCAGAGATGCTTAGG
TGCCTTCGGGAACTGTGAGACAGGTGCTGCATGGCTGTCGTCAGCTCGTGTTGTGAAAT
GTTGGGTTAAGTCCCGCAACGAGCGCAACCCTTATCCTTTGTTGCCAGCACGTGATGGT
GGGAACTCAAAGGAGACTGCCGGTGATAAACCGGAGGAAGGTGGGGATGACGTCAAG
TCATCATGGCCCTTACGAGTAGGGCTACACACGTGCTACAATGGCAGATACAAAGTGAA
GCGAACTCGCGAGAGCAAGCGGACCACATAAAGTCTGTCGTAGTCCGGATTGGAGTCT
GCAACTCGACTCCATGAAGTCGGAATCGCTAGTAATCGTAGATCAGAATGCTACGGTGA
ATACGTTCCCGGGCCTTGTACACACCGCCCGTCACACCATGGGAGTGGGTTGCAAAAG
AAGTAGGTAGCTTAACCTTCGGGAGGGCGCTTACCACTTTGTGATTCATGACTGGGG
pattern 87
CGCTGGCGGCAGGCCTAACACATGCAAGTCGAGCGGCAGCGGAAAGTAGCTTGCTACT
TTGCCGGCGAGCGGCGGACGGGTGAGTAATGTCTGGGGATCTGCCTAATGGAGGGGGA
TAACTACTGGAAACGGTAGCTAATACCGCATGACCTCGAAAGAGCAAAGTGGGGGACC
TTCGGGCCTCACGCCATCGGATGAACCCAGATGGGATTAGCTAGTAGGTGGGGTAATGG
CTCACCTAGGCGACGATCCCTAGCTGGTCTGAGAGGATGACCAGCCACACTGGAACTG
AGACACGGTCCAGACTCCTACGGGAGGCAGCAGTGGGGAATATTGCACAATGGGCGCA
AGCCTGATGCAGCCATGCCGCGTGTGTGAAGAAGGCCTTCGGGTTGTAAAGCACTTTCA
GCGAGGAGGAAGGCATTGTGGTTAATAACCACAGTGATTGACGTTACTCGCAGAAGAA
GCACCGGCTAACTCCGTGCCAGCAGCCGCGGTAATACGGAGGGTGCAAGCGTTAATCG
GAATTACTGGGCGTAAAGCGCACGCAGGCGGTTTGTTAAGTCAGATGTGAAATCCCCGC
GCTTAACGTGGGAACTGCATTTGAAACTGGCAAGCTAGAGTCTTGTAGAGGGGGGTAG
AATTCCAGGTGTAGCGGTGAAATGCGTAGAGATCTGGAGGAATACCGGTGGCGAAGGC
GGCCCCCTGGACAAAGACTGACGCTCAGGTGCGAAAGCGTGGGGAGCAAACAGGATT
AGATACCCTGGTAGTCCACGCTGTAAACGATGTCGACTTGGAGGTTGTGCCCTTGAGGT
GTGGCTTCCGGAGCTAACGCGTTAAGTCGACCGCCTGGGGAGTACGGCCGCAAGGTTA
AAACTCAAATGAATTGACGGGGGCCCGCACAAGCGGTGGAGCATGTGGTTTAATTCGAT
GCAACGCGAAGAACCTTACCTACTCTTGACATCCACGGAATTTAGCAGAGATGCTTTAG
TGCCTTCGGGAACCGTGAGACAGGTGCTGCATGGCTGTCGTCAGCTCGTGTTGTGAAAT
GTTGGGTTAAGTCCCGCAACGAGCGCAACCCTTATCCTTTGTTGCCAGCACGTAATGGT
GGGAACTCAAGGGAGACTGCCGGTGACAAACCGGAGGAAGGTGGGGATGACGTCAAG
TCATCATGGCCCTTACGAGTAGGGCTACACACGTGCTACAATGGCAGATACAAAGTGAA
GCGAACTCGCGAGAGCAAGCGGACCACATAAAGTCTGTCGTAGTCCGGATTGGAGTCT
GCAACTCGACTCCATGAAGTCGGAATCGCTAGTAATCGTAGATCAGAATGCTACGGTGA
ATACGTTCCCGGGCCTTGTACACACCGCCCGTCACACCATGGGAGTGGGTTGCAAAAG

AAGTAGGTAGCTTAACCTTCGGGAGGGCGCTTACCACTTTGTGATTCATGACTGGGG
pattern 88
CGCTGGCGGCAGGCCTAACACATGCAAGTCGAGCGGCAGCGGAAAGTAGCTTGCTACT
TTGCCGGCGAGCGGCGGACGGGTGAGTAATGTCTGGGGATCTGCCTGATGGAGGGGGA
TAACTACTGGAAACGGTAGCTAATACCGCATGACCTCGAAAGAGCAAAGTGGGGGACC
TTCGGGCCTCACGCCATCGGATGAACCCAGATGGGATTAGCTAGTAGGTGGGGTAATGG
CTCACCTAGGCGACGATCCCTAGCTGGTCTGAGAGGATGACCAGCCACACTGGAACTG
AGACACGGTCCAGACTCCTACGGGAGGCAGCAGTGGGGAATATTGCACAATGGGCGCA
AGCCTGATGCAGCCATGCCGCGTGTGTGAAGAAGGCCTTCGGGTTGTAAAGCACTTTCA
GCGAGGAGGAAGGCATTGTGGTTAATAACCGCAGTGATTGACGTTACTCGCAGAAGAA
GCACCGGCTAACTCCGTGCCAGCAGCCGCGGTAATACGGAGGGTGCAAGCGTTAATCG
GAATTACTGGGCGTAAAGCGCACGCAGGCGGTTTGTTAAGTCAGATGTGAAATCCCCGC
GCTTAACGTGGGAACTGCATTTGAAACTGGCAAGCTAGAGTCTTGTAGAGGGGGGTAG
AATTCCAGGTGTAGCGGTGAAATGCGTAGAGATCTGGAGGAATACCGGTGGCGAAGGC
GGCCCCCTGGACAAAGACTGACGCTCAGGTGCGAAAGCGTGGGGAGCAAACAGGATT
AGATACCCTGGTAGTCCACGCTGTAAACGATGTCGACTTGGAGGTTGTGCCCTTGAGGT
GTGGCTTCCGGAGCTAACGCGTTAAGTCGACCGCCTGGGGAGTACGGCCGCAAGGTTA
AAACTCAAATGAATTGACGGGGGCCCGCACAAGCGGTGGAGCATGTGGTTTAATTCGAT
GCAACGCGAAGAACCTTACCTACTCTTGACATCCACAGAACTTAGCAGAGATGCTTTAG
TGCCTTCGGGAACCGTGAGACAGGTGCTGCATGGCTGTCGTCAGCTCGTGTTGTGAAAT
GTTGGGTTAAGTCCCGCAACGAGCGCAACCCTTATCCTTTGTTGCCAGCACGTAATGGT
GGGAACTCAAGGGAGACTGCCGGTGACAAACCGGAGGAAGGTGGGGATGACGTCAAG
TCATCATGGCCCTTACGAGTAGGGCTACACACGTGCTACAATGGCAGATACAAAGTGAA
GCGAACTCGCGAGAGCAAGCGGACCACATAAAGTCTGTCGTAGTCCGGATTGGAGTCT
GCAACTCGACTCCATGAAGTCGGAATCGCTAGTAATCGTAGATCAGAATGCTACGGTGA
ATACGTTCCCGGGCCTTGTACACACCGCCCGTCACACCATGGGAGTGGGTTGCAAAAG
AAGTAGGTAGCTTAACCTTCGGGAGGGCGCTTACCACTTTGTGATTCATGACTGGGG
pattern 89
CGCTGGCGGCAGGCCTAACACATGCAAGTCGAGCGGCAGCGGAAAGTAGCTTGCTACT
TTGCCGGCGAGCGGCGGACGGATGAGTAATGTCTGGGGATCTGCCTGATGGAGGGGGA
TAACTACTGGAAACGGTAGCTAATACCGCATGACCTCGAAAGAGCAAAGTGGGGGACC
TTCGGGCCTCACGCCATCGGATGAACCCAGATGGGATTAGCTAGTAGGTGGGGTAATGG
CTCACCTAGGCGACGATCCCTAGCTGGTCTGAGAGGATGACCAGCCACACTGGAACTG
AGACACGGTCCAGACTCCTACGGGAGGCAGCAGTGGGGAATATTGCACAATGGGCGCA
AGCCTGATGCAGCCATGCCGCGTGTGTGAAGAAGGCCTTCGGGTTGTAAAGCACTTTCA
GCGAGGAGGAAGGCATTGTGGTTAATAACCACAGTGATTGACGTTACTCGCAGAAGAA
GCACCGGCTAACTCCGTGCCAGCAGCCGCGGTAATACGGAGGGTGCAAGCGTTAATCG
GAATTACTGGGCGTAAAGCGCACGCAGGCGGTTTGTTAAGTCAGATGTGAAATCCCCGC
GCTTAACGTGGGAACTGCATTTGAAACTGGCAAGCTAGAGTCTTGTAGAGGGGGGTAG
AATTCCAGGTGTAGCGGTGAAATGCGTAGAGATCTGGAGGAATACCGGTGGCGAAGGC
GGCCCCCTGGACAAAGACTGACGCTCAGGTGCGAAAGCGTGGGGAGCAAACAGGATT
AGATACCCTGGTAGTCCACGCTGTAAACGATGTCGACTTGGAGGTTGTGCCCTTGAGGC
GTGGCTTCCGGAGCTAACGCGTTAAGTCGACCGCCTGGGGAGTACGGCCGCAAGGTTA
AAACTCAAATGAATTGACGGGGGCCCGCACAAGCGGTGGAGCATGTGGTTTAATTCGAT

GCAACGCGAAGAACCTTACCTACTCTTGACATCCACAGAACTTAGCAGAGATGCTTCGG
TGCCTTCGGGAACTGTGAGACAGGTGCTGCATGGCTGTCGTCAGCTCGTGTTGTGAAAT
GTTGGGTTAAGTCCCGCAACGAGCGCAACCCTTATCCTTTGTTGCCAGCACGTAATGGT
GGGAACTCAAGGGAGACTGCCGGTGACAAACCGGAGGAAGGTGGGGATGACGTCAAG
TCATCATGGCCCTTACGAGTAGGGCTACACACGTGCTACAATGGCAGATACAAAGTGAA
GCGAACTCGCGAGAGCAAGCGGACCACATAAAGTCTGTCGTAGTCCGGATTGGAGTCT
GCAACTCGACTCCATGAAGTCGGAATCGCTAGTAATCGTAGATCAGAATGCTACGGTGA
ATACGTTCCCGGGCCTTGTACACACCGCCCGTCACACCATGGGAGTGGGTTGCAAAAG
AAGTAGGTAGCTTAACCTTCGGGAGGGCGCTTACCACTTTGTGATTCATGACTGGGG

pattern 90

CGCTGGCGGCAGGCCTAACACATGCAAGTCGAGCGGCAGCGGAAAGTAGCTTGCTACT
TTGCCGGCGAGCGGCGGACGGGTGAGTAATGTCTGGGAAATTGCCTGATGGAGGGGGA
TAACTACTGGAAACGGTAGCTAATACCGCATGACCTCGAAAGAGCAAAGTGGGGGACC
TTCGGGCCTCACGCCATCGGATGTGCCCAGATGGGATTAGCTAGTAGGTGAGGTAATGG
CTCACCTAGGCGACGATCCCTAGCTGGTCTGAGAGGATGACCAGCCACACTGGAACTG
AGACACGGTCCAGACTCCTACGGGAGGCAGCAGTGGGGAATATTGCACAATGGGCGCA
AGCCTGATGCAGCCATGCCGCGTGTGTGAAGAAGGCCTTCGGGTTGTAAAGCACTTTCA
GCGAGGAGGAAGGCATTTCACTTAATACGTGAAGTGATTGACGTTACTCGCAGAAGAA
GCACCGGCTAACTCCGTGCCAGCAGCCGCGGTAATACGGAGGGTGCAAGCGTTAATCG
GAATTACTGGGCGTAAAGCGCACGCAGGCGGTTTGTTAAGTCAGATGTGAAATCCCCGA
GCTTAACTTGGGAACTGCATTTGAAACTGGCAAGCTAGAGTCTTGTAGAGGGGGGTAG
AATTCCAGGTGTAGCGGTGAAATGCGTAGAGATCTGGAGGAATACCGGTGGCGAAGGC
GGCCCCCTGGACAAAGACTGACGCTCAGGTGCGAAAGCGTGGGGAGCAAACAGGATT
AGATACCCTGGTAGTCCACGCTGTAAACGATGTCGACTTGGAGGTTGTGCCCTTGAGGC
GTGGCTTCCGGAGCTAACGCGTTAAGTCGACCGCCTGGGGAGTACGGCCGCAAGGTTA
AAACTCAAATGAATTGACGGGGGCCCGCACAAGCGGTGGAGCATGTGGTTTAATTCGAT
GCAACGCGAAGAACCTTACCTACTCTTGACATCCACAGAACTTAGCAGAGATGCTTAGG
TGCCTTCGGGAACTGTGAGACAGGTGCTGCATGGCTGTCGTCAGCTCGTGTTGTGAAAT
GTTGGGTTAAGTCCCGCAACGAGCGCAACCCTTATCCTTTGTTGCCAGCACGTAATGGT
GGGAACTCAAAGGAGACTGCCGGTGATAAACCGGAGGAAGGTGGGGATGACGTCAAG
TCATCATGGCCCTTACGAGTAGGGCTACACACGTGCTACAATGGCAGATACAAAGTGAA
GCGAACTCGCGAGAGCAAGCGGACCACATAAAGTCTGTCGTAGTCCGGATTGGAGTCT
GCAACTCGACTCCATGAAGTCGGAATCGCTAGTAATCGTAGATCAGAATGCTACGGTGA
ATACGTTCCCGGGCCTTGTACACACCGCCCGTCACACCATGGGAGTGGGTTGCAAAAG
AAGTAGGTAGCTTAACCTTCGGGAGGGCGCTTACCACTTTGTGATTCATGACTGGGG

pattern 91

CGCTGGCGGCAGGCCTAACACATGCAAGTCGAGCGGCAGCGGAAAGTAGCTTGCTACT
TTGCCGGCGAGCGGCGGACGGGTGAGTAATGTCTGGGGATCTGCCTGATGGAGGGGGA
TAACTACTGGAAACGGTAGCTAATACCGCATGACCTCGAAAGAGCAAAGTGGGGGACC
TTCGGGCCTCACGCCATCGGATGAACCCAGATGGGATTAGCTAGTAGGTGGGGTAATGG
CTCACCTAGGCGACGATCCCTAGCTGGTCTGAGAGGATGACCAGCCACACTGGAACTG
AGACACGGTCCAGACTCCTACGGGAGGCAGCAGTGGGGAATATTGCACAATGGGCGCA
AGCCTGATGCAGCCATGCCGCGTGTGTGAAGAAGGCCTTCGGGTTGTAAAGCACTTTCA
GCGAGGAGGAAGGCATTGTGGTTAATAACCGCAGTGATTGACGTTACTCGCAGAAGAA

GCACCGGCTAACTCCGTGCCAGCAGCCGCGGTAATACGGAGGGTGCAAGCGTTAATCG
GAATTACTGGGCGTAAAGCGCACGCAGGCGGTTTGTTAAGTCAGATGTGAAATCCCCGC
GCTTAACGTGGGAACTGCATTTGAAACTGGCAAGCTAGAGTCTTGTAGAGGGGGGTGG
AATTCCAGGTGTAGCGGTGAAATGCGTAGAGATCTGGAGGAATACCGGTGGCGAAGGC
GGCCCCCTGGACAAAGACTGACGCTCAGGTGCGAAAGCGTGGGGAGCAAACAGGATT
AGATACCCTGGTAGTCCACGCTGTAAACGATGTCGACTTGGAGGTTGTGCCCTTGAGGC
GTGGCTTCCGGAGCTAACGCGTTAAGTCGACCGCCTGGGGAGTACGGCCGCAAGGTTA
AAACTCAAATGAATTGACGGGGGCCCGCACAAGCGGTGGAGCATGTGGTTTAATTCGAT
GCAACGCGAAGAACCTTACCTACTCTTGACATCCACAGAACTTAGCAGAGATGCTTCGG
TGCCTTCGGGAACTGTGAGACAGGTGCTGCATGGCTGTCGTCAGCTCGTGTTGTGAAAT
GTTGGGTTAAGTCCCGCAACGAGCGCAACCCTTATCCTTTGTTGCCAGCACGTAATGGT
GGGAACTCAAGGGAGACTGCCGGTGACAAACCGGAGGAAGGTGGGGATGACGTCAAG
TCATCATGGCCCTTACGAGTAGGGCTACACACGTGCTACAATGGCAGATACAAAGTGAA
GCGAACTCGCGAGAGCAAGCGGACCACATAAAGTCTGTCGTAGTCCGGATTGGAGTCT
GCAACTCGACTCCATGAAGTCGGAATCGCTAGTAATCGTAGATCAGAATGCTACGGTGA
ATACGTTCCCGGGCCTTGTACACACCGCCCGTCACACCATGGGAGTGGGTTGCAAAAG
AAGTAGGTAGCTTAACCTTCGGGAGGGCGCTTACCACTTTGTGATTCATGACTGGGG
pattern 92
CGCTGGCGGCAGGCCTAACACATGCAAGTCGAGCGGCAGCGGGAGGTAGTTTACTACT
TTGCCGGCGAGCGGCGGACGGGTGAGTAATGTCTGGGAAACTGCCTGATGGAGGGGGA
TAACTACTGGAAACGGTAGCTAATACCGCATGACCTCGCAAGAGCAAAGTGGGGGACC
TTCGGGCCTCACGCCATCGGATGTGCCCAGATGGGATTAGCTAGTAGGTGGGGTAATGG
CTCACCTAGGCGACGATCCCTAGCTGGTCTGAGAGGATGACCAGCCACACTGGAACTG
AGACACGGTCCAGACTCCTACGGGAGGCAGCAGTGGGGAATATTGCACAATGGGCGCA
AGCCTGATGCAGCCATGCCGCGTGTGTGAAGAAGGCCTTCGGGTTGTAAAGCACTTTCA
GCGAGGAGGAAGGCAGTCGTGTTAATAGCACGATTGATTGACGTTACTCGCAGAAGAA
GCACCGGCTAACTCCGTGCCAGCAGCCGCGGTAATACGGAGGGTGCAAGCGTTAATCG
GAATTACTGGGCGTAAAGCGCACGCAGGCGGTTTGTTAAGTCAGATGTGAAATCCCCGC
GCTTAACGTGGGAACTGCATTTGAAACTGGCAAGCTAGAGTCTTGTAGAGGGGGGTAG
AATTCCAGGTGTAGCGGTGAAATGCGTAGAGATCTGGAGGAATACCGGTGGCGAAGGC
GGCCCCCTGGACAAAGACTGACGCTCAGGTGCGAAAGCGTGGGGAGCAAACAGGATT
AGATACCCTGGTAGTCCACGCTGTAAACGATGTCGACTTGGAGGTTGTGCCCTTGAGGC
GTGGCTTCCGGAGCTAACGCGTTAAGTCGACCGCCTGGGGAGTACGGCCGCAAGGTTA
AAACTCAAATGAATTGACGGGGGCCCGCACAAGCGGTGGAGCATGTGGTTTAATTCGAT
GCAACGCGAAGAACCTTACCTACTCTTGACATCCACAGAACTTAGCAGAGATGCTTCGG
TGCCTTCGGGAACTGTGAGACAGGTGCTGCATGGCTGTCGTCAGCTCGTGTTGTGAAAT
GTTGGGTTAAGTCCCGCAACGAGCGCAACCCTTATCCTTTGTTGCCAGCACGTAATGGT
GGGAACTCAAGGGAGACTGCCGGTGACAAACCGGAGGAAGGTGGGGATGACGTCAAG
TCATCATGGCCCTTACGAGTAGGGCTACACACGTGCTACAATGGCAGATACAAAGTGAA
GCGAACTCGCGAGAGCAAGCGGACCACATAAAGTCTGTCGTAGTCCGGATTGGAGTCT
GCAACTCGACTCCATGAAGTCGGAATCGCTAGTAATCGTAGATCAGAATGCTACGGTGA
ATACGTTCCCGGGCCTTGTACACACCGCCCGTCACACCATGGGAGTGGGTTGCAAAAG
AAGTAGGTAGCTTAACCTTCGGGAGGGCGCTTACCACTTTGTGATTCATGACTGGGG
pattern 93

CGCTGGCGGCAGGCCTAACACATGCAAGTCGAGCGGCAGCGGGAAGTAGTTTACTACT
TCGCCGGCGAGCGGCGGACGGGTGAGTAATGTCTGGGAAACTGCCTGATGGAGGGGGA
TAACTACTGGAAACGGTAGCTAATACCGCATGACCTCGCAAGAGCAAAGTGGGGGACC
TTAGGGCCTCACGCCATCGGATGTGCCCAGATGGGATTAGCTAGTAGGTGGGGTAATGG
CTCACCTAGGCGACGATCCCTAGCTGGTCTGAGAGGATGACCAGCCACACTGGAACTG
AGACACGGTCCAGACTCCTACGGGAGGCAGCAGTGGGGAATATTGCACAATGGGCGCA
AGCCTGATGCAGCCATGCCGCGTGTGTGAAGAAGGCCTTCGGGTTGTAAAGCACTTTCA
GCGAGGAGGAAGGCAATCGTGTTAATAGCACGGTTGATTGACGTTACTCGCAGAAGAA
GCACCGGCTAACTCCGTGCCAGCAGCCGCGGTAATACGGAGGGTGCAAGCGTTAATCG
GAATTACTGGGCGTAAAGCGCACGCAGGCGGTTTGTTAAGTCAGATGTGAAATCCCCGC
GCTTAACGTGGGAACTGCATTTGAAACTGGCAAGCTAGAGTCTTGTAGAGGGGGGTAG
AATTCCAGGTGTAGCGGTGAAATGCGTAGAGATCTGGAGGAATACCGGTGGCGAAGGC
GGCCCCCTGGACAAAGACTGACGCTCAGGTGCGAAAGCGTGGGGAGCAAACAGGATT
AGATACCCTGGTAGTCCACGCTGTAAACGATGTCGACTTGGAGGTTGTGCCCTTGAGGC
GTGGCTTCCGGAGCTAACGCGTTAAGTCGACCGCCTGGGGAGTACGGCCGCAAGGTTA
AAACTCAAATGAATTGACGGGGGCCCGCACAAGCGGTGGAGCATGTGGTTTAATTCGAT
GCAACGCGAAGAACCTTACCTACTCTTGACATCCACAGAACTTAGCAGAGATGCTTCGG
TGCCTTCGGGAACTGTGAGACAGGTGCTGCATGGCTGTCGTCAGCTCGTGTTGTGAAAT
GTTGGGTTAAGTCCCGCAACGAGCGCAACCCTTATCCTTTGTTGCCAGCACGTAATGGT
GGGAACTCAAGGGAGACTGCCGGTGACAAACCGGAGGAAGGTGGGGATGACGTCAAG
TCATCATGGCCCTTACGAGTAGGGCTACACACGTGCTACAATGGCAGATACAAAGTGAA
GCGAACTCGCGAGAGCAAGCGGACCACATAAAGTCTGTCGTAGTCCGGATTGGAGTCT
GCAACTCGACTCCATGAAGTCGGAATCGCTAGTAATCGTAGATCAGAATGCTACGGTGA
ATACGTTCCCGGGCCTTGTACACACCGCCCGTCACACCATGGGAGTGGGTTGCAAAAG
AAGTAGGTAGCTTAACCTTCGGGAGGGCGCTTACCACTTTGTGATTCATGACTGGGG
pattern 94
CGCTGGCGGCAGGCCTAACACATGCAAGTCGAGCGGCAGCGGGAAGTAGTTTACTACT
TCGCCGGCGAGCGGCGGACGGGTGAGTAATGTCTGGGAAACTGCCTGATGGAGGGGGA
TAACTACTGGAAACGGTAGCTAATACCGCATGACCTCGCAAGAGCAAAGTGGGGGACC
TTAGGGCCTCACGCCATCGGATGTGCCCAGATGGGATTAGCTAGTAGGTGGGGTAATGG
CTCACCTAGGCGACGATCCCTAGCTGGTCTGAGAGGATGACCAGCCACACTGGAACTG
AGACACGGTCCAGACTCCTACGGGAGGCAGCAGTGGGGAATATTGCACAATGGGCGCA
AGCCTGATGCAGCCATGCCGCGTGTGTGAAGAAGGCCTTCGGGTTGTAAAGCACTTTCA
GCGAGGAGGAAGGCAATCGTGTTAATAGCACGGTTGATTGACGTTACTCGCAGAAGAA
GCACCGGCTAACTCCGTGCCAGCAGCCGCGGTAATACGGAGGGTGCAAGCGTTAATCG
GAATTACTGGGCGTAAAGCGCACGCAGGCGGTTTGTTAAGTCAGATGTGAAATCCCCGC
GCTTAACGTGGGAACTGCATTTGAAACTGGCAAGCTAGAGTCTTGTAGAGGGGGGTAG
AATTCCAGGTGTAGCGGTGAAATGCGTAGAGATCTGGAGGAATACCGGTGGCGAAGGC
GGCCCCCTGGACAAAGACTGACGCTCAGGTGCGAAAGCGTGGGGAGCAAACAGGATT
AGATACCCTGGTAGTCCACGCTGTAAACGATGTCGACTTGGAGGTTGTGCCCTTGAGGC
GTGGCTTCCGGAGCTAACGCGTTAAGTCGACCGCCTGGGGAGTACGGCCGCAAGGTTA
AAACTCAAATGAATTGACGGGGGCCCGCACAAGCGGTGGAGCATGTGGTTTAATTCGAT
GCAACGCGAAGAACCTTACCTACTCTTGACATCCACAGAACTTAGCAGAGATGCTTCGG
TGCCTTCGGGAACTGTGAGACAGGTGCTGCATGGCCGTCGTCAGCTCGTGTTGTGAAAT

GTTGGGTTAAGTCCCGCAACGAGCGCAACCCTTATCCTTTGTTGCCAGCACGTAATGGT
GGGAACTCAAGGGAGACTGCCGGTGACAAACCGGAGGAAGGTGGGGATGACGTCAAG
TCATCATGGCCCTTACGAGTAGGGCTACACACGTGCTACAATGGCAGATACAAAGTGAA
GCGAACTCGCGAGAGCAAGCGGACCACATAAAGTCTGTCGTAGTCCGGATTGGAGTCT
GCAACTCGACTCCATGAAGTCGGAATCGCTAGTAATCGTAGATCAGAATGCTACGGTGA
ATACGTTCCCGGGCCTTGTACACACCGCCCGTCACACCATGGGAGTGGGTTGCAAAAG
AAGTAGGTAGCTTAACCTTCGGGAGGGCGCTTACCACTTTGTGATTCATGACTGGGG

pattern 95

CGCTGGCGGCAGGCCTAACACATGCAAGTCGAGCGGCAGCGGGAAGTAGTTTACTACT
TTGCCGGCGAGCGGCGGACGGGTGAGTAATGTCTGGGAAACTGCCTGATGGAGGGGGA
TAACTACTGGAAACGGTAGCTAATACCGCATGACCTCGCAAGAGCAAAGTGGGGGACC
TTAGGGCCTCACGCCATCGGATGTGCCCAGATGGGATTAGCTAGTAGGTGGGGTAATGG
CTCACCTAGGCGACGATCCCTAGCTGGTCTGAGAGGATGACCAGCCACACTGGAACTG
AGACACGGTCCAGACTCCTACGGGAGGCAGCAGTGGGGAATATTGCACAATGGGCGCA
AGCCTGATGCAGCCATGCCGCGTGTGTGAAGAAGGCCTTCGGGTTGTAAAGCACTTTCA
GCGAGGAGGAAGGCAATCGTGTTAATAGCACGGTTGATTGACGTTACTCGCAGAAGAA
GCACCGGCTAACTCCGTGCCAGCAGCCGCGGTAATACGGAGGGTGCAAGCGTTAATCG
GAATTACTGGGCGTAAAGCGCACGCAGGCGGTTTGTTAAGTCAGATGTGAAATCCCCGC
GCTTAACGTGGGAACTGCATTTGAAACTGGCAAGCTAGAGTCTTGTAGAGGGGGGTAG
AATTCCAGGTGTAGCGGTGAAATGCGTAGAGATCTGGAGGAATACCGGTGGCGAAGGC
GGCCCCCTGGACAAAGACTGACGCTCAGGTGCGAAAGCGTGGGGAGCAAACAGGATT
AGATACCCTGGTAGTCCACGCTGTAAACGATGTCGACTTGGAGGTTGTGCCCTTGAGGC
GTGGCTTCCGGAGCTAACGCGTTAAGTCGACCGCCTGGGGAGTACGGCCGCAAGGTTA
AAACTCAAATGAATTGACGGGGGCCCGCACAAGCGGTGGAGCATGTGGTTTAATTCGAT
GCAACGCGAAGAACCTTACCTACTCTTGACATCCACAGAACTTAGCAGAGATGCTTCGG
TGCCTTCGGGAACTGTGAGACAGGTGCTGCATGGCTGTCGTCAGCTCGTGTTGTGAAAT
GTTGGGTTAAGTCCCGCAACGAGCGCAACCCTTATCCTTTGTTGCCAGCACGTAATGGT
GGGAACTCAAGGGAGACTGCCGGTGACAAACCGGAGGAAGGTGGGGATGACGTCAAG
TCATCATGGCCCTTACGAGTAGGGCTACACACGTGCTACAATGGCAGATACAAAGTGAA
GCGAACTCGCGAGAGCAAGCGGACCACATAAAGTCTGTCGTAGTCCGGATTGGAGTCT
GCAACTCGACTCCATGAAGTCGGAATCGCTAGTAATCGTAGATCAGAATGCTACGGTGA
ATACGTTCCCGGGCCTTGTACACACCGCCCGTCACACCATGGGAGTGGGTTGCAAAAG
AAGTAGGTAGCTTAACCTTCGGGAGGGCGCTTACCACTTTGTGATTCATGACTGGGG

pattern 96

CGCTGGCGGCAGGCCTAACACATGCAAGTCGAGCGGCAGCGGGAAGTAGTTTACTACT
TTGCCGGCGAGCGGCGGACGGGTGAGTAATGTCTGGGAAACTGCCTGATGGAGGGGGA
TAACTACTGGAAACGGTAGCTAATACCGCATGACCTCGCAAGAGCAAAGTGGGGGACC
TTAGGGCCTCACGCCATCGGATGTGCCCAGATGGGATTAGCTAGTAGGTGGGGTAATGG
CTCACCTAGGCGACGATCCCTAGCTGGTCTGAGAGGATGACCAGCCACACTGGAACTG
AGACACGGTCCAGACTCCTACGGGAGGCAGCAGTGGGGAATATTGCACAATGGGCGCA
AGCCTGATGCAGCCATGCCGCGTGTGTGAAGAAGGCCTTCGGGTTGTAAAGCACTTTCA
GCGAGGAGGAAGGCAATCGTGTTAATAGCACGGTTGATTGACGTTACTCGCAGAAGAA
GCACCGGCTAACTCCGTGCCAGCAGCCGCGGTAATACGGAGGGTGCAAGCGTTAATCG
GAATTACTGGGCGTAAAGCGCACGCAGGCGGTTTGTTAAGTCAGATGTGAAATCCCCGC

GCTTAACGTGGGAACTGCATTTGAAACTGGCAAGCTAGAGTCTTGTAGAGGGGGGTAG
AATTCCAGGTGTAGCGGTGAAATGCGTAGAGATCTGGAGGAATACCGGTGGCGAAGGC
GGCCCCCTGGACAAAGACTGACGCTCAGGTGCGAAAGCGTGGGGAGCAAACAGGATT
AGATACCCTGGTAGTCCACGCTGTAAACGATGTCGACTTGGAGGTTGTGCCCTTGAGGC
GTGGCTTCCGGAGCTAACGCGTTAAGTCGACCGCCTGGGGAGTACGGCCGCAAGGTTA
AAACTCAAATGAATTGACGGGGGCCCGCACAAGCGGTGGAGCATGTGGTTTAATTCGAT
GCAACGCGAAGAACCTTACCTACTCTTGACATCCACAGAACTTAGCAGAGATGCTTCGG
TGCCTTCGGGAACTGTGAGACAGGTGCTGCATGGCCGTCGTCAGCTCGTGTTGTGAAAT
GTTGGGTTAAGTCCCGCAACGAGCGCAACCCTTATCCTTTGTTGCCAGCACGTAATGGT
GGGAACTCAAGGGAGACTGCCGGTGACAAACCGGAGGAAGGTGGGGATGACGTCAAG
TCATCATGGCCCTTACGAGTAGGGCTACACACGTGCTACAATGGCAGATACAAAGTGAA
GCGAACTCGCGAGAGCAAGCGGACCACATAAAGTCTGTCGTAGTCCGGATTGGAGTCT
GCAACTCGACTCCATGAAGTCGGAATCGCTAGTAATCGTAGATCAGAATGCTACGGTGA
ATACGTTCCCGGGCCTTGTACACACCGCCCGTCACACCATGGGAGTGGGTTGCAAAAG
AAGTAGGTAGCTTAACCTTCGGGAGGGCGCTTACCACTTTGTGATTCATGACTGGGG
pattern 97
CGCTGGCGGCAGGCCTAACACATGCAAGTCGAGCGGCAGCGGGAAGTAGTTTACTACT
TCGCCGGCGAGCGGCGGACGGGTGAGTAATGTCTGGGAAACTGCCTGATGGAGGGGGA
TAACTACTGGAAACGGTAGCTAATACCGCATGACCTCGCAAGAGCAAAGTGGGGGACC
TTAGGGCCTCACGCCATCGGATGTGCCCAGATGGGATTAGCTAGTAGGTGGGGTAATGG
CTCACCTAGGCGACGATCCCTAGCTGGTCTGAGAGGATGACCAGCCACACTGGAACTG
AGACACGGTCCAGACTCCTACGGGAGGCAGCAGTGGGGAATATTGCACAATGGGCGCA
AGCCTGATGCAGCCATGCCGCGTGTGTGAAGAAGGCCTTCGGGTTGTAAAGCACTTTCA
GCGAGGAGGAAAGCAATCGTGTTAATAGCACGGTTGATTGACGTTACTCGCAGAAGAA
GCACCGGCTAACTCCGTGCCAGCAGCCGCGGTAATACGGAGGGTGCAAGCGTTAATCG
GAATTACTGGGCGTAAAGCGCACGCAGGCGGTTTGTTAAGTCAGATGTGAAATCCCCGC
GCTTAACGTGGGAACTGCATTTGAAACTGGCAAGCTAGAGTCTTGTAGAGGGGGGTAG
AATTCCAGGTGTAGCGGTGAAATGCGTAGAGATCTGGAGGAATACCGGTGGCGAAGGC
GGCCCCCTGGACAAAGACTGACGCTCAGGTGCGAAAGCGTGGGGAGCAAACAGGATT
AGATACCCTGGTAGTCCACGCTGTAAACGATGTCGACTTGGAGGTTGTGCCCTTGAGGC
GTGGCTTCCGGAGCTAACGCGTTAAGTCGACCGCCTGGGGAGTACGGCCGCAAGGTTA
AAACTCAAATGAATTGACGGGGGCCCGCACAAGCGGTGGAGCATGTGGTTTAATTCGAT
GCAACGCGAAGAACCTTACCTACTCTTGACATCCACAGAACTTAGCAGAGATGCTTCGG
TGCCTTCGGGAACTGTGAGACAGGTGCTGCATGGCTGTCGTCAGCTCGTGTTGTGAAAT
GTTGGGTTAAGTCCCGCAACGAGCGCAACCCTTATCCTTTGTTGCCAGCACGTAATGGT
GGGAACTCAAGGGAGACTGCCGGTGACAAACCGGAGGAAGGTGGGGATGACGTCAAG
TCATCATGGCCCTTACGAGTAGGGCTACACACGTGCTACAATGGCAGATACAAAGTGAA
GCGAACTCGCGAGAGCAAGCGGACCACATAAAGTCTGTCGTAGTCCGGATTGGAGTCT
GCAACTCGACTCCATGAAGTCGGAATCGCTAGTAATCGTAGATCAGAATGCTACGGTGA
ATACGTTCCCGGGCCTTGTACACACCGCCCGTCACACCATGGGAGTGGGTTGCAAAAG
AAGTAGGTAGCTTAACCTTCGGGAGGGCGCTTACCACTTTGTGATTCATGACTGGGG
pattern 98
CGCTGGCGGCAGGCCTAACACATGCAAGTCGAGCGGCAGCGGGAAGTAGTTTACTACT
TTGCCGGCGAGCGGCGGACGGGTGAGTAATGTCTGGGAAACTGCCTGATGGAGGGGGA

TAACTACTGGAAACGGTAGCTAATACCGCATGACCTCGCAAGAGCAAAGTGGGGGACC
TTCGGGCCTCACGCCATCGGATGTGCCCAGATGGGATTAGCTAGTAGGTGAGGTAATGG
CTCACCTAGGCGACGATCCCTAGCTGGTCTGAGAGGATGACCAGCCACACTGGAACTG
AGACACGGTCCAGACTCCTACGGGAGGCAGCAGTGGGGAATATTGCACAATGGGCGCA
AGCCTGATGCAGCCATGCCGCGTGTGTGAAGAAGGCCTTCGGGTTGTAAAGCACTTTCA
GCGAGGAGGAAGGGGTTGAGTTTAATACGCTCAATCATTGACGTTACTCGCAGAAGAA
GCACCGGCTAACTCCGTGCCAGCAGCCGCGGTAATACGGAGGGTGCAAGCGTTAATCG
GAATTACTGGGCGTAAAGCGCACGCAGGCGGTTTGTTAAGTCAGATGTGAAATCCCCGC
GCTTAACGTGGGAACTGCATTTGAAACTGGCAAGCTAGAGTCTTGTAGAGGGGGGTAG
AATTCCAGGTGTAGCGGTGAAATGCGTAGAGATCTGGAGGAATACCGGTGGCGAAGGC
GGCCCCCTGGACAAAGACTGACGCTCAGGTGCGAAAGCGTGGGGAGCAAACAGGATT
AGATACCCTGGTAGTCCACGCTGTAAACGATGTCGACTTGGAGGTTGTGCCCTTGAGGC
GTGGCTTCCGGAGCTAACGCGTTAAGTCGACCGCCTGGGGAGTACGGCCGCAAGGTTA
AAACTCAAATGAATTGACGGGGGCCCGCACAAGCGGTGGAGCATGTGGTTTAATTCGAT
GCAACGCGAAGAACCTTACCTACTCTTGACATCCACAGAACTTAGCAGAGATGCTTCGG
TGCCTTCGGGAACTGTGAGACAGGTGCTGCATGGCTGTCGTCAGCTCGTGTTGTGAAAT
GTTGGGTTAAGTCCCGCAACGAGCGCAACCCTTATCCTTTGTTGCCAGCACGTAATGGT
GGGAACTCAAAGGAGACTGCCGGTGACAAACCGGAGGAAGGTGGGGATGACGTCAAG
TCATCATGGCCCTTACGAGTAGGGCTACACACGTGCTACAATGGCAGATACAAAGTGAA
GCGAACTCGCGAGAGCAAGCGGACCACATAAAGTCTGTCGTAGTCCGGATTGGAGTCT
GCAACTCGACTCCATGAAGTCGGAATCGCTAGTAATCGTAGATCAGAATGCTACGGTGA
ATACGTTCCCGGGCCTTGTACACACCGCCCGTCACACCATGGGAGTGGGTTGCAAAAG
AAGTAGGTAGCTTAACCTTCGGGAGGGCGCTTACCACTTTGTGATTCATGACTGGGG
pattern 99
CGCTGGCGGCAGGCCTAACACATGCAAGTCGAGCGGCAGCGGGAAGTAGTTTACTACT
TTGCCGGCGAGCGGCGGACGGGTGAGTAATGTCTGGGAAACTGCCTGATGGAGGGGGA
TAACTACTGGAAACGGTAGCTAATACCGCATGACCTCGCAAGAGCAAAGTGGGGGACC
TTCGGGCCTCACGCCATCGGATGTGCCCAGATGGGATTAGCTAGTAGGTGGGGTAATGG
CTCACCTAGGCGACGATCCCTAGCTGGTCTGAGAGGATGACCAGCCACACTGGAACTG
AGACACGGTCCAGACTCCTACGGGAGGCAGCAGTGGGGAATATTGCACAATGGGCGCA
AGCCTGATGCAGCCATGCCGCGTGTGTGAAGAAGGCCTTCGGGTTGTAAAGCACTTTCA
GCGAGGAGGAAGGGGTTGAGTTTAATACGCTCAATCATTGACGTTACTCGCAGAAGAA
GCACCGGCTAACTCCGTGCCAGCAGCCGCGGTAATACGGAGGGTGCAAGCGTTAATCG
GAATTACTGGGCGTAAAGCGCACGCAGGCGGTTTGTTAAGTCAGATGTGAAATCCCCGC
GCTTAACGTGGGAACTGCATTTGAAACTGGCAAGCTAGAGTCTTGTAGAGGGGGGTAG
AATTCCAGGTGTAGCGGTGAAATGCGTAGAGATCTGGAGGAATACCGGTGGCGAAGGC
GGCCCCCTGGACAAAGACTGACGCTCAGGTGCGAAAGCGTGGGGAGCAAACAGGATT
AGATACCCTGGTAGTCCACGCTGTAAACGATGTCGACTTGGAGGTTGTGCCCTTGAGGC
GTGGCTTCCGGAGCTAACGCGTTAAGTCGACCGCCTGGGGAGTACGGCCGCAAGGTTA
AAACTCAAATGAATTGACGGGGGCCCGCACAAGCGGTGGAGCATGTGGTTTAATTCGAT
GCAACGCGAAGAACCTTACCTACTCTTGACATCCACAGAACTTAGCAGAGATGCTTCGG
TGCCTTCGGGAACTGTGAGACAGGTGCTGCATGGCTGTCGTCAGCTCGTGTTGTGAAAT
GTTGGGTTAAGTCCCGCAACGAGCGCAACCCTTATCCTTTGTTGCCAGCACGTAATGGT
GGGAACTCAAAGGAGACTGCCGGTGACAAACCGGAGGAAGGTGGGGATGACGTCAAG

TCATCATGGCCCTTACGAGTAGGGCTACACACGTGCTACAATGGCAGATACAAAGTGAA
GCGAACTCGCGAGAGCAAGCGGACCACATAAAGTCTGTCGTAGTCCGGATTGGAGTCT
GCAACTCGACTCCATGAAGTCGGAATCGCTAGTAATCGTAGATCAGAATGCTACGGTGA
ATACGTTCCCGGGCCTTGTACACACCGCCCGTCACACCATGGGAGTGGGTTGCAAAAG
AAGTAGGTAGCTTAACCTTCGGGAGGGCGCTTACCACTTTGTGATTCATGACTGGGG
pattern 100
CGCTGGCGGCAGGCCTAACACATGCAAGTCGAGCGGCAGCGGAAAGTAGTTTACTACT
TTGCCGGCGAGCGGCGGACGGGTGAGTAATGTCTGGGAAACTGCCTGATGGAGGGGGA
TAACTACTGGAAACGGTAGCTAATACCGCATGACCTCGCAAGAGCAAAGTGGGGGACC
TTCGGGCCTCACGCCATCGGATGTGCCCAGATGGGATTAGCTAGTAGGTGAGGTAATGG
CTCACCTAGGCGACGATCCCTAGCTGGTCTGAGAGGATGACCAGCCACACTGGAACTG
AGACACGGTCCAGACTCCTACGGGAGGCAGCAGTGGGGAATATTGCACAATGGGCGCA
AGCCTGATGCAGCCATGCCGCGTGTGTGAAGAAGGCCTTCGGGTTGTAAAGCACTTTCA
GCGAGGAGGAAGGGGTTGAGTTTAATACGCTCAATCATTGACGTTACTCGCAGAAGAA
GCACCGGCTAACTCCGTGCCAGCAGCCGCGGTAATACGGAGGGTGCAAGCGTTAATCG
GAATTACTGGGCGTAAAGCGCACGCAGGCGGTTTGTTAAGTCAGATGTGAAATCCCCGC
GCTTAACGTGGGAACTGCATTTGAAACTGGCAAGCTAGAGTCTTGTAGAGGGGGGTAG
AATTCCAGGTGTAGCGGTGAAATGCGTAGAGATCTGGAGGAATACCGGTGGCGAAGGC
GGCCCCCTGGACAAAGACTGACGCTCAGGTGCGAAAGCGTGGGGAGCAAACAGGATT
AGATACCCTGGTAGTCCACGCTGTAAACGATGTCGACTTGGAGGTTGTGCCCTTGAGGC
GTGGCTTCCGGAGCTAACGCGTTAAGTCGACCGCCTGGGGAGTACGGCCGCAAGGTTA
AAACTCAAATGAATTGACGGGGGCCCGCACAAGCGGTGGAGCATGTGGTTTAATTCGAT
GCAACGCGAAGAACCTTACCTACTCTTGACATCCACAGAACTTAGCAGAGATGCTTCGG
TGCCTTCGGGAACTGTGAGACAGGTGCTGCATGGCTGTCGTCAGCTCGTGTTGTGAAAT
GTTGGGTTAAGTCCCGCAACGAGCGCAACCCTTATCCTTTGTTGCCAGCACGTAATGGT
GGGAACTCAAAGGAGACTGCCGGTGACAAACCGGAGGAAGGTGGGGATGACGTCAAG
TCATCATGGCCCTTACGAGTAGGGCTACACACGTGCTACAATGGCAGATACAAAGTGAA
GCGAACTCGCGAGAGCAAGCGGACCACATAAAGTCTGTCGTAGTCCGGATTGGAGTCT
GCAACTCGACTCCATGAAGTCGGAATCGCTAGTAATCGTAGATCAGAATGCTACGGTGA
ATACGTTCCCGGGCCTTGTACACACCGCCCGTCACACCATGGGAGTGGGTTGCAAAAG
AAGTAGGTAGCTTAACCTTCGGGAGGGCGCTTACCACTTTGTGATTCATGACTGGGG
pattern 101
CGCTGGCGGCAGGCCTAACACATGCAAGTCGAGCGGCAGCGGAAAGTAGTTTACTACT
TTGCCGGCGAGCGGCGGACGGGTGAGTAATGTCTGGGAAACTGCCTGATGGAGGGGGA
TAACTACTGGAAACGGTAGCTAATACCGCATGACCTCGCAAGAGCAAAGTGGGGGACC
TTCGGGCCTCACGCCATCGGATGTGCCCAGATGGGATTAGCTAGTAGGTGGGGTAATGG
CTCACCTAGGCGACGATCCCTAGCTGGTCTGAGAGGATGACCAGCCACACTGGAACTG
AGACACGGTCCAGACTCCTACGGGAGGCAGCAGTGGGGAATATTGCACAATGGGCGCA
AGCCTGATGCAGCCATGCCGCGTGTGTGAAGAAGGCCTTCGGGTTGTAAAGCACTTTCA
GCGAGGAGGAAGGGGTTGAGTTTAATACGCTCAATCATTGACGTTACTCGCAGAAGAA
GCACCGGCTAACTCCGTGCCAGCAGCCGCGGTAATACGGAGGGTGCAAGCGTTAATCG
GAATTACTGGGCGTAAAGCGCACGCAGGCGGTTTGTTAAGTCAGATGTGAAATCCCCGC
GCTTAACGTGGGAACTGCATTTGAAACTGGCAAGCTAGAGTCTTGTAGAGGGGGGTAG
AATTCCAGGTGTAGCGGTGAAATGCGTAGAGATCTGGAGGAATACCGGTGGCGAAGGC

GGCCCCCTGGACAAAGACTGACGCTCAGGTGCGAAAGCGTGGGGAGCAAACAGGATT
AGATACCCTGGTAGTCCACGCTGTAAACGATGTCGACTTGGAGGTTGTGCCCTTGAGGC
GTGGCTTCCGGAGCTAACGCGTTAAGTCGACCGCCTGGGGAGTACGGCCGCAAGGTTA
AAACTCAAATGAATTGACGGGGGCCCGCACAAGCGGTGGAGCATGTGGTTTAATTCGAT
GCAACGCGAAGAACCTTACCTACTCTTGACATCCACAGAACTTAGCAGAGATGCTTCGG
TGCCTTCGGGAACTGTGAGACAGGTGCTGCATGGCTGTCGTCAGCTCGTGTTGTGAAAT
GTTGGGTTAAGTCCCGCAACGAGCGCAACCCTTATCCTTTGTTGCCAGCACGTAATGGT
GGGAACTCAAAGGAGACTGCCGGTGACAAACCGGAGGAAGGTGGGGATGACGTCAAG
TCATCATGGCCCTTACGAGTAGGGCTACACACGTGCTACAATGGCAGATACAAAGTGAA
GCGAACTCGCGAGAGCAAGCGGACCACATAAAGTCTGTCGTAGTCCGGATTGGAGTCT
GCAACTCGACTCCATGAAGTCGGAATCGCTAGTAATCGTAGATCAGAATGCTACGGTGA
ATACGTTCCCGGGCCTTGTACACACCGCCCGTCACACCATGGGAGTGGGTTGCAAAAG
AAGTAGGTAGCTTAACCTTCGGGAGGGCGCTTACCACTTTGTGATTCATGACTGGGG
pattern 102
CGCTGGCGGCAGGCCTAACACATGCAAGTCGAGCGGCAGCGGAAAGTAGCTTGCTACT
TTGCCGGCGAGCGGCGGACGGGTGAGTAATGTCTGGGGATCTGCCTGATGGAGGGGGA
TAACTACTGGAAACGGTAGCTAATACCGCATGACCTCGAAAGAGCAAAGTGGGGGACC
TTCGGGCCTCACGCCATCGGATGAACCCAGATGGGATTAGCTAGTAGGTGGGGTAATGG
CTCACCTAGGCGACGATCCCTAGCTGGTCTGAGAGGATGACCAGCCACACTGGAACTG
AGACACGGTCCAGACTCCTACGGGAGGCAGCAGTGGGGAATATTGCACAATGGGCGCA
AGCCTGATGCAGCCATGCCGCGTGTGTGAAGAAGGCCTTCGGGTTGTAAAGCACTTTCA
GCGAGGAGGAAAGCATTGTGGTTAATAACCGCAGTGATTGACGTTACTCGCAGAAGAA
GCACCGGCTAACTCCGTGCCAGCAGCCGCGGTAATACGGAGGGTGCAAGCGTTAATCG
GAATTACTGGGCGTAAAGCGCACGCAGGCGGTTTGTTAAGTCAGATGTGAAATCCCCGC
GCTTAACGTGGGAACTGCATTTGAAACTGGCAAGCTAGAGTCTTGTAGAGGGGGGTAG
AATTCCAGGTGTAGCGGTGAAATGCGTAGAGATCTGGAGGAATACCGGTGGCGAAGGC
GGCCCCCTGGACAAAGACTGACGCTCAGGTGCGAAAGCGTGGGGAGCAAACAGGATT
AGATACCCTGGTAGTCCACGCTGTAAACGATGTCGACTTGGAGGTTGTGCCCTTGAGGC
GTGGCTTCCGGAGCTAACGCGTTAAGTCGACCGCCTGGGGAGTACGGCCGCAAGGTTA
AAACTCAAATGAATTGACGGGGGCCCGCACAAGCGGTGGAGCATGTGGTTTAATTCGAT
GCAACGCGAAGAACCTTACCTACTCTTGACATCCACAGAACTTAGCAGAGATGCTTCGG
TGCCTTCGGGAACTGTGAGACAGGTGCTGCATGGCTGTCGTCAGCTCGTGTTGTGAAAT
GTTGGGTTAAGTCCCGCAACGAGCGCAACCCTTATCCTTTGTTGCCAGCACGTAATGGT
GGGAACTCAAGGGAGACTGCCGGTGACAAACCGGAGGAAGGTGGGGATGACGTCAAG
TCATCATGGCCCTTACGAGTAGGGCTACACACGTGCTACAATGGCAGATACAAAGTGAA
GCGAACTCGCGAGAGCAAGCGGACCACATAAAGTCTGTCGTAGTCCGGATTGGAGTCT
GCAACTCGACTCCATGAAGTCGGAATCGCTAGTAATCGTAGATCAGAATGCTACGGTGA
ATACGTTCCCGGGCCTTGTACACACCGCCCGTCACACCATGGGAGTGGGTTGCAAAAG
AAGTAGGTAGCTTAACCTTCGGGAGGGCGCTTACCACTTTGTGATTCATGACTGGGG
pattern 103
CGCTGGCGGCAGGCCTAACACATGCAAGTCGAGCGGCAGCGGAAAGTAGCTTGCTACT
TTGCCGGCGAGCGGCGGACGGGTGAGTAATGTCTGGGGATCTGCCTGATGGAGGGGGA
TAACTACTGGAAACGGTAGCTAATACCGCATGACCTCGAAAGAGCAAAGTGGGGGACC
TTCGGGCCTCACGCCATCGGATGAACCCAGATGGGATTAGCTAGTAGGTGGGGTAATGG

CTCACCTAGGCGACGATCCCTAGCTGGTCTGAGAGGATGACCAGCCACACTGGAACTG
AGACACGGTCCAGACTCCTACGGGAGGCAGCAGTGGGGAATATTGCACAATGGGCGCA
AGCCTGATGCAGCCATGCCGCGTGTGTGAAGAAGGCCTTCGGGTTGTAAAGCACTTTCA
GCGAGGAGGAAAGCATTGTGGTTAATAACCGCAGTGATTGACGTTACTCGCAGAAGAA
GCACCGGCTAACTCCGTGCCAGCAGCCGCGGTAATACGGAGGGTGCAAGCGTTAATCG
GAATTACTGGGCGTAAAGCGCACGCAGGCGGTTTGTTAAGTCAGATGTGAAATCCCCGC
GCTTAACGTGGGAACTGCATTTGAAACTGGCAAGCTAGAGTCTTGTAGAGGGGGGTAG
AATTCCAGGTGTAGCGGTGAAATGCGTAGAGATCTGGAGGAATACCGGTGGCGAAGGC
GGCCCCCTGGACAAAGACTGACGCTCAGGTGCGAAAGCGTGGGGAGCAAACAGGATT
AGATACCCTGGTAGTCCACGCTGTAAACGATGTCGACTTGGAGGTTGTGCCCTTGAGGC
GTGGCTTCCGGAGCTAACGCGTTAAGTCGACCGCCTGGGGAGTACGGCCGCAAGGTTA
AAACTCAAATGAATTGACGGGGGCCCGCACAAGCGGTGGAGCATGTGGTTTAATTCGAT
GCAACGCGAAGAACCTTACCTACTCTTGACATCCACAGAACTTAGCAGAGATGCTTCGG
TGCCTTCGGGAACTGTGAGACAGGTGCTGCATGGCTGTCGTCAGCTCGTGTTGTGAAAT
GTTGGGTTAAGTCCCGCAACGAGCGCAACCCTTATCCTTTGTTGCCAGCACGTAATGGT
GGGAACTCAAGGGAGACTGCCGGTGACAAACCGGAGGAAGGTGGGGATGACGTCAAG
TCATCATGGCCCTTACGAGTAGGGCTACACACGTGCTACAATGGCAGATACAAAGTGAA
GCGAACTCGCGAGAGCAAGCGGACCACATAAAGTCTGTCGTAGTCCGGATTGGAGTCT
GCAACTCGACTCCATGAAGTCGGAATCGCTAGTAATCGTAGATCAGAATGCTACGGTGA
ATACGTTCCCGGGCCTTGTACACACCGCCCGTCACACCATGGGAGTAGGTTGCAAAAGA
AGTAGGTAGCTTAACCTTCGGGAGGGCGCTTACCACTTTGTGATTCATGACTGGGG
pattern 104
CGCTGGCGGCAGGCCTAACACATGCAAGTCGAGCGGCAGCGGAAAGTAGCTTGCTACT
TTGCCGGCGAGCGGCGGACGGGTGAGTAATGTCTGGGAAACTGCCTGATGGAGGGGGA
TAACTACTGGAAACGGTAGCTAATACCGCATGACCTCGAAAGAGCAAAGTGGGGGACC
TTCGGGCCTCACGCCATCGGATGTGCCCAGATGGGATTAGCTAGTAGGTGGGGTAATGG
CTCACCTAGGCGACGATCCCTAGCTGGTCTGAGAGGATGACCAGCCACACTGGAACTG
AGACACGGTCCAGACTCCTACGGGAGGCAGCAGTGGGGAATATTGCACAATGGGCGCA
AGCCTGATGCAGCCATGCCGCGTGTGTGAAGAAGGCCTTCGGGTTGTAAAGCACTTTCA
GCGAGGAGGAAGGCATTTCACTTAATACGTGAAGTGATTGACGTTACTCGCAGAAGAA
GCACCGGCTAACTCCGTGCCAGCAGCCGCGGTAATACGGAGGGTGCAAGCGTTAATCG
GAATTACTGGGCGTAAAGCGCACGCAGGCGGTTTGTTAAGTCAGATGTGAAATCCCCGA
GCTTAACTTGGGAACTGCATTTGAAACTGGCAAGCTAGAGTCTTGTAGAGGGGGGTAG
AATTCCAGGTGTAGCGGTGAAATGCGTAGAGATCTGGAGGAATACCGGTGGCGAAGGC
GGCCCCCTGGACAAAGACTGACGCTCAGGTGCGAAAGCGTGGGGAGCAAACAGGATT
AGATACCCTGGTAGTCCACGCTGTAAACGATGTCGACTTGGAGGTTGTGCCCTTGAGGC
GTGGCTTCCGGAGCTAACGCGTTAAGTCGACCGCCTGGGGAGTACGGCCGCAAGGTTA
AAACTCAAATGAATTGACGGGGGCCCGCACAAGCGGTGGAGCATGTGGTTTAATTCGAT
GCAACGCGAAGAACCTTACCTACTCTTGACATCCACAGAACTTAGCAGAGATGCTTAGG
TGCCTTCGGGAACTGTGAGACAGGTGCTGCATGGCTGTCGTCAGCTCGTGTTGTGAAAT
GTTGGGTTAAGTCCCGCAACGAGCGCAACCCTTATCCTTTGTTGCCAGCACGTGATGGT
GGGAACTCAAAGGAGACTGCCGGTGATAAACCGGAGGAAGGTGGGGATGACGTCAAG
TCATCATGGCCCTTACGAGTAGGGCTACACACGTGCTACAATGGCAGATACAAAGTGAA
GCGAACTCGCGAGAGCAAGCGGACCACATAAAGTCTGTCGTAGTCCGGATTGGAGTCT

GCAACTCGACTCCATGAAGTCGGAATCGCTAGTAATCGTAGATCAGAATGCTACGGTGA
ATACGTTCCCGGGCCTTGTACACACCGCCCGTCACACCATGGGAGTGGGTTGCAAAAG
AAGTAGGTAGCTTAACCTTCGGGAGGGCGCTTACCACTTTGTGATTCATGACTGGGG
pattern 105
CGCTGGCGGCAGGCCTAACACATGCAAGTCGAGCGGCAGCGGAAAGTAGCTTGCTACT
TTGCCGGCGAGCGGCGGACGGGTGAGTAATGTCTGGGAAACTGCCTGATGGAGGGGGA
TAACTACTGGAAACGGTAGCTAATACCGCATGACCTCGAAAGAGCAAAGTGGGGGACC
TTCGGGCCTCACGCCATCGGATGTGCCCAGATGGGATTAGCTAGTAGGTGGGGTAATGG
CTCACCTAGGCGACGATCCCTAGCTGGTCTGAGAGGATGACCAGCCACACTGGAACTG
AGACACGGTCCAGACTCCTACGGGAGGCAGCAGTGGGGAATATTGCACAATGGGCGCA
AGCCTGATGCAGCCATGCCGCGTGTGTGAAGAAGGCCTTCGGGTTGTAAAGCACTTTCA
GCGAGGAGGAAGGCATTTCACTTAATACGTGAGGTGATTGACGTTACTCGCAGAAGAA
GCACCGGCTAACTCCGTGCCAGCAGCCGCGGTAATACGGAGGGTGCAAGCGTTAATCG
GAATTACTGGGCGTAAAGCGCACGCAGGCGGTTTGTTAAGTCAGATGTGAAATCCCCGA
GCTTAACTTGGGAACTGCATTTGAAACTGGCAAGCTAGAGTCTTGTAGAGGGGGGTAG
AATTCCAGGTGTAGCGGTGAAATGCGTAGAGATCTGGAGGAATACCGGTGGCGAAGGC
GGCCCCCTGGACAAAGACTGACGCTCAGGTGCGAAAGCGTGGGGAGCAAACAGGATT
AGATACCCTGGTAGTCCACGCTGTAAACGATGTCGACTTGGAGGTTGTGCCCTTGAGGC
GTGGCTTCCGGAGCTAACGCGTTAAGTCGACCGCCTGGGGAGTACGGCCGCAAGGTTA
AAACTCAAATGAATTGACGGGGGCCCGCACAAGCGGTGGAGCATGTGGTTTAATTCGAT
GCAACGCGAAGAACCTTACCTACTCTTGACATCCACAGAACTTAGCAGAGATGCTTAGG
TGCCTTCGGGAACTGTGAGACAGGTGCTGCATGGCTGTCGTCAGCTCGTGTTGTGAAAT
GTTGGGTTAAGTCCCGCAACGAGCGCAACCCTTATCCTTTGTTGCCAGCACGTGATGGT
GGGAACTCAAAGGAGACTGCCGGTGATAAACCGGAGGAAGGTGGGGATGACGTCAAG
TCATCATGGCCCTTACGAGTAGGGCTACACACGTGCTACAATGGCAGATACAAAGTGAA
GCGAACTCGCGAGAGCAAGCGGACCACATAAAGTCTGTCGTAGTCCGGATTGGAGTCT
GCAACTCGACTCCATGAAGTCGGAATCGCTAGTAATCGTAGATCAGAATGCTACGGTGA
ATACGTTCCCGGGCCTTGTACACACCGCCCGTCACACCATGGGAGTGGGTTGCAAAAG
AAGTAGGTAGCTTAACCTTCGGGAGGGCGCTTACCACTTTGTGATTCATGACTGGGG
pattern 106
CGCTGGCGGCAGGCCTAACACATGCAAGTCGAGCGGCAGCGGAAAGTAGCTTGCTACT
TTGCCGGCGAGCGGCGGACGGGTGAGTAATGTCTGGGAAACTGCCTGATGGAGGGGGA
TAACTACTGGAAACGGTAGCTAATACCGCATGACCTCGAAAGAGCAAAGTGGGGGACC
TTCGGGCCTCACGCCATCGGATGTGCCCAGATGGGATTAGCTAGTAGGTGGGGTAATGG
CTCACCTAGGCGACGATCCCTAGCTGGTCTGAGAGGATGACCAGCCACACTGGAACTG
AGACACGGTCCAGACTCCTACGGGAGGCAGCAGTGGGGAATATTGCACAATGGGCGCA
AGCCTGATGCAGCCATGCCGCGTGTGTGAAGAAGGCCTTCGGGTTGTAAAGCACTTTCA
GCGAGGAGGAAGGCATTTCACTTAATACGTGAGGTGATTGACGTTACTCGCAGAAGAA
GCACCGGCTAACTCCGTGCCAGCAGCCGCGGTAATACGGAGGGTGCAAGCGTTAATCG
GAATTACTGGGCGTAAAGCGCACGCAGGCGGTTTGTTAAGTCAGATGTGAAATCCCCGA
GCTTAACTTGGGAACTGCATTTGAAACTGGCAAGCTAGAGTCTTGTAGAGGGGGGTAG
AATTCCAGGTGTAGCGGTGAAATGCGTAGAGATCTGGAGGAATACCGGTGGCGAAGGC
GGCCCCCTGGACAAAGACTGACGCTCAGGTGCGAAAGCGTGGGGAGCAAACAGGATT
AGATACCCTGGTAGTCCACGCTGTAAACGATGTCGACTTGGAGGTTGTGCCCTTGAGGC

GTGGCTTCCGGAGCTAACGCGTTAAGTCGACCGCCTGGGGAGTACGGCCGCAAGGTTA
AAACTCAAATGAATTGACGGGGGCCCGCACAAGCGGTGGAGCATGTGGTTTAATTCGAT
GCAACGCGAAGAACCTTACCTACTCTTGACATCCACAGAACTGAGCAGAGATGCTTAG
GTGCCTTCGGGAACTGTGAGACAGGTGCTGCATGGCTGTCGTCAGCTCGTGTTGTGAAA
TGTTGGGTTAAGTCCCGCAACGAGCGCAACCCTTATCCTTTGTTGCCAGCACGTAATGG
TGGGAACTCAAAGGAGACTGCCGGTGATAAACCGGAGGAAGGTGGGGATGACGTCAA
GTCATCATGGCCCTTACGAGTAGGGCTACACACGTGCTACAATGGCAGATACAAAGTGA
AGCGAACTCGCGAGAGCAAGCGGACCACATAAAGTCTGTCGTAGTCCGGATTGGAGTC
TGCAACTCGACTCCATGAAGTCGGAATCGCTAGTAATCGTAGATCAGAATGCTACGGTG
AATACGTTCCCGGGCCTTGTACACACCGCCCGTCACACCATGGGAGTGGGTTGCAAAA
GAAGTAGGTAGCTTAACCTTCGGGAGGGCGCTTACCACTTTGTGATTCATGACTGGGG
pattern 107
CGCTGGCGGCAGGCCTAACACATGCAAGTCGAGCGGCAGCGGAAAGTAGCTTGCTACT
TTGCCGGCGAGCGGCGGACGGGTGAGTAATGTCTGGGAAACTGCCTGATGGAGGGGGA
TAACTACTGGAAACGGTAGCTAATACCGCATGACCTCGAAAGAGCAAAGTGGGGGACC
TTCGGGCCTCACGCCATCGGATGTGCCCAGATGGGATTAGCTAGTAGGTGGGGTAATGG
CTCACCTAGGCGACGATCCCTAGCTGGTCTGAGAGGATGACCAGCCACACTGGAACTG
AGACACGGTCCAGACTCCTACGGGAGGCAGCAGTGGGGAATATTGCACAATGGGCGCA
AGCCTGATGCAGCCATGCCGCGTGTGTGAAGAAGGCCTTCGGGTTGTAAAGCACTTTCA
GCGAGGAGGAAGGCATTTCACTTAATACGTGAGGTGATTGACGTTACTCGCAGAAGAA
GCACCGGCTAACTCCGTGCCAGCAGCCGCGGTAATACGGAGGGTGCAAGCGTTAATCG
GAATTACTGGGCGTAAAGCGCACGCAGGCGGTTTGTTAAGTCAGATGTGAAATCCCCGA
GCTTAACTTGGGAACTGCATTTGAAACTGGCAAGCTAGAGTCTTGTAGAGGGGGGTAG
AATTCCAGGTGTAGCGGTGAAATGCGTAGAGATCTGGAGGAATACCGGTGGCGAAGGC
GGCCCCCTGGACAAAGACTGACGCTCAGGTGCGAAAGCGTGGGGAGCAAACAGGATT
AGATACCCTGGTAGTCCACGCTGTAAACGATGTCGACTTGGAGGTTGTGCCCTTGAGGC
GTGGCTTCCGGAGCTAACGCGTTAAGTCGACCGCCTGGGGAGTACGGCCGCAAGGTTA
AAACTCAAATGAATTGACGGGGGCCCGCACAAGCGGTGGAGCATGTGGTTTAATTCGAT
GCAACGCGAAGAACCTTACCTACTCTTGACATCCACAGAACTTAGCAGAGATGCTTAGG
TGCCTTCGGGAACTGTGAGACAGGTGCTGCATGGCTGTCGTCAGCTCGTGTTGTGAAAT
GTTGGGTTAAGTCCCGCAACGAGCGCAACCCTTATCCTTTGTTGCCAGCACGTAATGGT
GGGAACTCAAAGGAGACTGCCGGTGATAAACCGGAGGAAGGTGGGGATGACGTCAAG
TCATCATGGCCCTTACGAGTAGGGCTACACACGTGCTACAATGGCAGATACAAAGTGAA
GCGAACTCGCGAGAGCAAGCGGACCACATAAAGTCTGTCGTAGTCCGGATTGGAGTCT
GCAACTCGACTCCATGAAGTCGGAATCGCTAGTAATCGTAGATCAGAATGCTACGGTGA
ATACGTTCCCGGGCCTTGTACACACCGCCCGTCACACCATGGGAGTGGGTTGCAAAAG
AAGTAGGTAGCTTAACCTTCGGGAGGGCGCTTACCACTTTGTGATTCATGACTGGGG
pattern 108
CGCTGGCGGCAGGCCTAACACATGCAAGTCGAGCGGCAGTGGGAAGTAGTTTACTACT
TTGCCGGCGAGCGGCGGACGGGTGAGTAATGTCTGGGAAACTGCCTGATGGAGGGGGA
TAACTACTGGAAACGGTAGCTAATACCGCATAACGTCTTCGGACCAAAGTGGGGGACCT
TCGGGCCTCACGCCATCGGATGTGCCCAGATGGGATTAGCTAGTAGGTGGGGTAATGGC
TCACCTAGGCGACGATCCCTAGCTGGTCTGAGAGGATGACCAGCCACACTGGAACTGA
GACACGGTCCAGACTCCTACGGGAGGCAGCAGTGGGGAATATTGCACAATGGGCGCAA

GCCTGATGCAGCCATGCCGCGTGTGTGAAGAAGGCCTTCGGGTTGTAAAGCACTTTCAG
CGAGGAGGAAGGCATAAAGGTTAATAACCTTTGTGATTGACGTTACTCGCAGAAGAAG
CACCGGCTAACTCCGTGCCAGCAGCCGCGGTAATACGGAGGGTGCAAGCGTTAATCGG
AATTACTGGGCGTAAAGCGCACGCAGGCGGTTTGTTAAGTCAGATGTGAAATCCCCGCG
CTTAACGTGGGAACTGCATTTGAAACTGGCAAGCTAGAGTCTTGTAGAGGGGGGTAGA
ATTCCAGGTGTAGCGGTGAAATGCGTAGAGATCTGGAGGAATACCGGTGGCGAAGGCG
GCCCCCTGGACAAAGACTGACGCTCAGGTGCGAAAGCGTGGGGAGCAAACAGGATTA
GATACCCTGGTAGTCCACGCTGTAAACGATGTCGACTTGGAGGTTGTGCCCTTGAGGCG
TGGCTTCCGGAGCTAACGCGTTAAGTCGACCGCCTGGGGAGTACGGCCGCAAGGTTAA
AACTCAAATGAATTGACGGGGGCCCGCACAAGCGGTGGAGCATGTGGTTTAATTCGATG
CAACGCGAAGAACCTTACCTACTCTTGACATCCACGGAATTTAGCAGAGATGCTTTAGT
GCCTTCGGGAACCGTGAGACAGGTGCTGCATGGCTGTCGTCAGCTCGTGTTGTGAAATG
TTGGGTTAAGTCCCGCAACGAGCGCAACCCTTATCCTTTGTTGCCAGCACGTGATGGTG
GGAACTCAAAGGAGACTGCCGGTGATAAACCGGAGGAAGGTGGGGATGACGTCAAGT
CATCATGGCCCTTACGAGTAGGGCTACACACGTGCTACAATGGCAGATACAAAGTGAAG
CGAACTCGCGAGAGCAAGCGGACCACATAAAGTCTGTCGTAGTCCGGATTGGAGTCTG
CAACTCGACTCCATGAAGTCGGAATCGCTAGTAATCGTAGATCAGAATGCTACGGTGAAT
ACGTTCCCGGGCCTTGTACACACCGCCCGTCACACCATGGGAGTGGGTTGCAAAAGAA
GTAGGTAGCTTAACCTTCGGGAGGGCGCTTACCCCTTTGTGATTCATGACTGGGG
pattern 109
CGCTGGCGGCAGGCCTAACACATGCAAGTCGAGCGGCAGCGGGAAGTAGTTTACTACT
TTGCCGGCGAGCGGCGGACGGGTGAGTAATGTCTGGGAAACTGCCTGATGGAGGGGGA
TAAACTACTGGAAACGGTAGCTAATACCGCATAACGTCTTCGGACCAAAGTGGGGGACC
TTCGGGCCTCACGCCATCGGATGTGCCCAGATGGGATTAGCTAGTAGGTGGGGTAATGG
CTCACCTAGGCGACGATCCCTAGCTGGTCTGAGAGGATGACCAGCCACACTGGAACTG
AGACACGGTCCAGACTCCTACGGGAGGCAGCAGTGGGGAATATTGCACAATGGGCGCA
AGCCTGATGCAGCCATGCCGCGTGTGTGAAGAAGGCCTTCGGGTTGTAAAGCACTTTCA
GCGAGGAGGAAGGCATAAAGGTTAATAACCTTTGTGATTGACGTTACTCGCAGAAGAA
GCACCGGCTAACTCCGTGCCAGCAGCCGCGGTAATACGGAGGGTGCAAGCGTTAATCG
GAATTACTGGGCGTAAAGCGCACGCAGGCGGTTTGTTAAGTCAGATGTGAAATCCCCGC
GCTTAACGTGGGAACTGCATTTGAAACTGGCAAGCTAGAGTCTTGTAGAGGGGGGTAG
AATTCCAGGTGTAGCGGTGAAATGCGTAGAGATCTGGAGGAATACCGGTGGCGAAGGC
GGCCCCCTGGACAAAGACTGACGCTCAGGTGCGAAAGCGTGGGGAGCAAACAGGATT
AGATACCCTGGTAGTCCACGCTGTAAACGATGTCGACTTGGAGGTTGTGCCCTTGAGGC
GTGGCTTCCGGAGCTAACGCGTTAAGTCGACCGCCTGGGGAGTACGGCCGCAAGGTTA
AAACTCAAATGAATTGACGGGGGCCCGCACAAGCGGTGGAGCATGTGGTTTAATTCGAT
GCAACGCGAAGAACCTTACCTACTCTTGACATCCACGGAATTTAGCAGAGATGCTTTAG
TGCCTTCGGGAACCGTGAGACAGGTGCTGCATGGCTGTCGTCAGCTCGTGTTGTGAAAT
GTTGGGTTAAGTCCCGCAACGAGCGCAACCCTTATCCTTTGTTGCCAGCACGTGATGGT
GGGAACTCAAAGGAGACTGCCGGTGATAAACCGGAGGAAGGTGGGGATGACGTCAAG
TCATCATGGCCCTTACGAGTAGGGCTACACACGTGCTACAATGGCAGATACAAAGTGAA
GCGAACTCGCGAGAGCAAGCGGACCACATAAAGTCTGTCGTAGTCCGGATTGGAGTCT
GCAACTCGACTCCATGAAGTCGGAATCGCTAGTAATCGTAGATCAGAATGCTACGGTGA
ATACGTTCCCGGGCCTTGTACACACCGCCCGTCACACCATGGGAGTGGGTTGCAAAAG

AAGTAGGTAGCTTAACCTTCGGGAGGGCGCTTACCACTTTGTGATTCATGACTGGGG
pattern 110
CGCTGGCGGCAGGCCTAACACATGCAAGTCGAGCGGCAGCGGGAAGTAGTTTACTACT
TTGCCGGCGAGCGGCGGACGGGTGAGTAATGTCTGGGAAACTGCCTGATGGAGGGGGA
TAACTACTGGAAACGGTAGCTAATACCGCATAACGTCTTCGGACCAAAGTGGGGGACCT
TCGGGCCTCACGCCATCGGATGTGCCCAGATGGGATTAGCTAGTAGGTGGGGGTAATGG
CTCACCTAGGCGACGATCCCTAGCTGGTCTGAGAGGATGACCAGCCACACTGGAACTG
AGACACGGTCCAGACTCCTACGGGAGGCAGCAGTGGGGAATATTGCACAATGGGCGCA
AGCCTGATGCAGCCATGCCGCGTGTGTGAAGAAGGCCTTCGGGTTGTAAAGCACTTTCA
GCGAGGAGGAAGGCATAAAGGTTAATAACCTTTGTGATTGACGTTACTCGCAGAAGAA
GCACCGGCTAACTCCGTGCCAGCAGCCGCGGTAATACGGAGGGTGCAAGCGTTAATCG
GAATTACTGGGCGTAAAGCGCACGCAGGCGGTTTGTTAAGTCAGATGTGAAATCCCCGC
GCTTAACGTGGGAACTGCATTTGAAACTGGCAAGCTAGAGTCTTGTAGAGGGGGGTAG
AATTCCAGGTGTAGCGGTGAAATGCGTAGAGATCTGGAGGAATACCGGTGGCGAAGGC
GGCCCCCTGGACAAAGACTGACGCTCAGGTGCGAAAGCGTGGGGAGCAAACAGGATT
AGATACCCTGGTAGTCCACGCTGTAAACGATGTCGACTTGGAGGTTGTGCCCTTGAGGC
GTGGCTTCCGGAGCTAACGCGTTAAGTCGACCGCCTGGGGAGTACGGCCGCAAGGTTA
AAACTCAAATGAATTGACGGGGGCCCGCACAAGCGGTGGAGCATGTGGTTTAATTCGAT
GCAACGCGAAGAACCTTACCTACTCTTGACATCCACGGAATTTAGCAGAGATGCTTTAG
TGCCTTCGGGAACCGTGAGACAGGTGCTGCATGGCTGTCGTCAGCTCGTGTTGTGAAAT
GTTGGGTTAAGTCCCGCAACGAGCGCAACCCTTATCCTTTGTTGCCAGCACGTGATGGT
GGGAACTCAAAGGAGACTGCCGGTGATAAACCGGAGGAAGGTGGGGATGACGTCAAG
TCATCATGGCCCTTACGAGTAGGGCTACACACGTGCTACAATGGCAGATACAAAGTGAA
GCGAACTCGCGAGAGCAAGCGGACCACATAAAGTCTGTCGTAGTCCGGATTGGAGTCT
GCAACTCGACTCCATGAAGTCGGAATCGCTAGTAATCGTAGATCAGAATGCTACGGTGA
ATACGTTCCCGGGCCTTGTACACACCGCCCGTCACACCATGGGAGTGGGTTGCAAAAG
AAGTAGGTAGCTTAACCTTCGGGAGGGCGCTTACCACTTTGTGATTCACGACTGGGG
pattern 111
CGCTGGCGGCAGGCCTAACACATGCAAGTCGAGCGGCAGCGGGAAGTAGTTTACTACT
TTGCCGGCGAGCGGCGGACGGGTGAGTAATGTCTGGGAAACTGCCTGATGGAGGGGGA
TAACTACTGGAAACGGTAGCTAATACCGCATGACCTCGCAAGAGCAAAGTGGGGGACC
TTCGGGCCTCACGCCATCGGATGTGCCCAGATGGGATTAGCTAGTAGGTGGGGTAATGG
CTCACCTAGGCGACGATCCCTAGCTGGTCTGAGAGGATGACCAGCCACACTGGAACTG
AGACACGGTCCAGACTCCTACGGGAGGCAGCAGTGGGGAATATTGCACAATGGGCGCA
AGCCTGATGCAGCCATGCCGCGTGTGTGAAGAAGGCCTTCGGGTTGTAAAGCACTTTCA
GCGAGGAGGAAGGCAGTCGTGTTAATAGCACGGTTGATTGACGTTACTCGCAGAAGAA
GCACCGGCTAACTCCGTGCCAGCAGCCGCGGTAATACGGAGGGTGCAAGCGTTAATCG
GAATTACTGGGCGTAAAGCGCACGCAGGCGGTTTGTTAAGTCAGATGTGAAATCCCCGC
GCTTAACGTGGGAACTGCATTTGAAACTGGCAAGCTAGAGTCTTGTAGAGGGGGGTAG
AATTCCAGGTGTAGCGGTGAAATGCGTAGAGATCTGGAGGAATACCGGTGGCGAAGGC
GGCCCCCTGGACAAAGACTGACGCTCAGGTGCGAAAGCGTGGGGAGCAAACAGGATT
AGATACCCTGGTAGTCCACGCTGTAAACGATGTCGACTTGGAGGTTGTGCCCTTGAGGC
GTGGCTTCCGGAGCTAACGCGTTAAGTCGACCGCCTGGGGAGTACGGCCGCAAGGTTA
AAACTCAAATGAATTGACGGGGGCCCGCACAAGCGGTGGAGCATGTGGTTTAATTCGAT

GCAACGCGAAGAACCTTACCTACTCTTGACATCCACAGAACTTAGCAGAGATGCTTCGG
TGCCTTCGGGAACTGTGAGACAGGTGCTGCATGGCTGTCGTCAGCTCGTGTTGTGAAAT
GTTGGGTTAAGTCCCGCAACGAGCGCAACCCTTATCCTTTGTTGCCAGCACGTAATGGT
GGGAACTCAAGGGAGACTGCCGGTGACAAACCGGAGGAAGGTGGGGATGACGTCAAG
TCATCATGGCCCTTACGAGTAGGGCTACACACGTGCTACAATGGCAGATACAAAGTGAA
GCGAACTCGCGAGAGCAAGCGGACCACATAAAGTCTGTCGTAGTCCGGATTGGAGTCT
GCAACTCGACTCCATGAAGTCGGAATCGCTAGTAATCGTAGATCAGAATGCTACGGTGA
ATACGTTCCCGGGCCTTGTACACACCGCCCGTCACACCATGGGAGTGGGTTGCAAAAG
AAGTAGGTAGCTTAACCTTCGGGAGGGCGCTTACCACTTTGTGATTCATGACTGGGG

pattern 112

CGCTGGCGGCAGGCCTAACACATGCAAGTCGAGCGGCAGCGGAAAGTAGCTTGCTACT
TTGCCGGCGAGCGGCGGACGGGTGAGTAATGTCTGGGGATCTGCCTGATGGAGGGGGA
TAACTACTGGAAACGGTAGCTAATACCGCATGACCTCGAAAGAGCAAAGTGGGGGACC
TTCGGGCCTCACGCCATCGGATGAACCCAGATGGGATTAGCTAGTAGGTGGGGTAATGG
CTCACCTAGGCGACGATCCCTAGCTGGTCTGAGAGGATGACCAGCCACACTGGAACTG
AGACACGGTCCAGACTCCTACGGGAGGCAGCAGTGGGGAATATTGCACAATGGGCGCA
AGCCTGATGCAGCCATGCCGCGTGTGTGAAGAAGGCCTTCGGGTTGTAAAGCACTTTCA
GCGAGGAGGAAGGCATTGTGGTTAATAACTGCAGTGATTGACGTTACTCGCAGAAGAA
GCACCGGCTAACTCCGTGCCAGCAGCCGCGGTAATACGGAGGGTGCAAGCGTTAATCG
GAATTACTGGGCGTAAAGCGCACGCAGGCGGTTTGTTAAGTCAGATGTGAAATCCCCGC
GCTTAACGTGGGAACTGCATTTGAAACTGGCAAGCTAGAGTCTTGTAGAGGGGGGTAG
AATTCCAGGTGTAGCGGTGAAATGCGTAGAGATCTGGAGGAATACCGGTGGCGAAGGC
GGCCCCCTGGACAAAGACTGACGCTCAGGTGCGAAAGCGTGGGGAGCAAACAGGATT
AGATACCCTGGTAGTCCACGCTGTAAACGATGTCGACTTGGAGGTTGTGCCCTTGAGGC
GTGGCTTCCGGAGCTAACGCGTTAAGTCGACCGCCTGGGGAGTACGGCCGCAAGGTTA
AAACTCAAATGAATTGACGGGGGCCCGCACAAGCGGTGGAGCATGTGGTTTAATTCGAT
GCAACGCGAAGAACCTTACCTACTCTTGACATCCACGGAATTTAGCAGAGATGCTTTAG
TGCCTTCGGGAACCGTGAGACAGGTGCTGCATGGCTGTCGTCAGCTCGTGTTGTGAAAT
GTTGGGTTAAGTCCCGCAACGAGCGCAACCCTTATCCTTTGTTGCCAGCACGTAATGGT
GGGAACTCAAGGGAGACTGCCGGTGACAAACCGGAGGAAGGTGGGGATGACGTCAAG
TCATCATGGCCCTTACGAGTAGGGCTACACACGTGCTACAATGGCAGATACAAAGTGAA
GCGAACTCGCGAGAGCAAGCGGACCACATAAAGTCTGTCGTAGTCCGGATTGGAGTCT
GCAACTCGACTCCATGAAGTCGGAATCGCTAGTAATCGTAGATCAGAATGCTACGGTGA
ATACGTTCCCGGGCCTTGTACACACCGCCCGTCACACCATGGGAGTGGGTTGCAAAAG
AAGTAGGTAGCTTAACCTTCGGGAGGGCGCTTACCACTTTGTGATTCATGACTGGGG

pattern 113

CGCTGGCGGCAGGCCTAACACATGCAAGTCGAGCGGCAGCGGGAAGTAGTTTACTACT
TTGCCGGCGAGCGGCGGACGGGTGAGTAATGTCTGGGAAACTGCCTGATGGAGGGGGA
TAACTACTGGAAACGGTAGCTAATACCGCATAACGTCTTCGGACCAAAGTGGGGGACCT
TCGGGCCTCACGCCATCGGATGTGCCCAGATGGGATTAGCTAGTAGGTGGGGTAATGGC
TCACCTAGGCGACGATCCCTAGCTGGTCTGAGAGGATGACCAGCCACACTGGAACTGA
GACACGGTCCAGACTCCTACGGGAGGCAGCAGTGGGGAATATTGCACAATGGGCGCAA
GCCTGATGCAGCCATGCCGCGTGTGTGAAGAAGGCCTTCGGGTTGTAAAGCACTTTCAG
CGAGGAGGAAGGCATAAAGGTTAATAACCTTTGTGATTGACGTTACTCGCAGAAGAAG

CACCGGCTAACTCCGTGCCAGCAGCCGCGGTAATACGGAGGGTGCAAGCGTTAATCGG
AATTACTGGGCGTAAAGCGCACGCAGGCGGTTTGTTAAGTCAGATGTGAAATCCCCGCG
CTTAACGTGGGAACTGCATTTGAAACTGGCAAGCTAGAGTCTTGTAGAGGGGGGTAGA
ATTCCAGGTGTAGTGGTGAAATGCGTAGAGATCTGGAGGAATACCGGTGGCGAAGGCG
GCCCCCTGGACAAAGACTGACGCTCAGGTGCGAAAGCGTGGGGAGCAAACAGGATTA
GATACCCTGGTAGTCCACGCTGTAAACGATGTCGACTTGGAGGTTGTGCCCTTGAGGCG
TGGCTTCCGGAGCTAACGCGTTAAGTCGACCGCCTGGGGAGTACGGCCGCAAGGTTAA
AACTCAAATGAATTGACGGGGGCCCGCACAAGCGGTGGAGCATGTGGTTTAATTCGATG
CAACGCGAAGAACCTTACCTACTCTTGACATCCACAGAACTTAGCAGAGATGCTTCGGT
GCCTTCGGGAATTGTGAGACAGGTGCTGCATGGCTGTCGTCAGCTCGTGTTGTGAAATG
TTGGGTTAAGTCCCGCAACGAGCGCAACCCTTATCCTTTGTTGCCAGCACGTGATGGTG
GGAACTCAAAGGAGACTGCCGGTGATAAACCGGAGGAAGGTGGGGATGACGTCAAGT
CATCATGGCCCTTACGAGTAGGGCTACACACGTGCTACAATGGCAGATACAAAGTGAAG
CGAACTCGCGAGAGCAAGCGGACCACATAAAGTCTGTCGTAGTCCGGATTGGAGTCTG
CAACTCGACTCCATGAAGTCGGAATCGCTAGTAATCGTAGATCAGAATGCTACGGTGAAT
ACGTTCCCGGGCCTTGTACACACCGCCCGTCACACCATGGGAGTGGGTTGCAAAAGAA
GTAGGTAGCTTAACCTTCGGGAGGGCGCTTACCACTTTGTGATTCATGACTGGGG
pattern 114
CGCTGGCGGCAGGCCTAACACATGCAAGTCGAGCGGCAGCGGGAAGTAGTTTACTACT
TTGCCGGCGAGCGGCGGACGGGTGAGTAATGTCTGGGAAACTGCCTGATGGAGGGGGA
TAACTACTGGAAACGGTAGCTAATACCGCATAACGTCTTCGGACCAAAGTGGGGGACCT
TCGGGCCTCACGCCATCGGATGTGCCCAGATGGGATTAGCTAGTAGGTGGGGTAATGGC
TCACCTAGGCGACGATCCCTAGCTGGTCTGAGAGGATGACCAGCCACACTGGAACTGA
GACACGGTCCAGACTCCTACGGGAGGCAGCAGTGGGGAATATTGCACAATGGGCGCAA
GCCTGATGCAGCCATGCCGCGTGTGTGAAGAAGGCCTTCGGGTTGTAAAGCACTTTCAG
CGAGGAGGAAGGCATAAAGGTTAATAACCTTTGTGATTGACGTTACTCGCAGAAGAAG
CACCGGCTAACTCCGTGCCAGCAGCCGCGGTAATACGGAGGGTGCAAGCGTTAATCGG
AATTACTGGGCGTAAAGCGCACGCAGGCGGTTTGTTAAGTCAGATGTGAAATCCCCGCG
CTTAACGTGGGAACTGCATTTGAAACTGGCAAGCTAGAGTCTTGTAGAGGGGGGTAGA
ATTCCAGGTGTAGCGGTGAAATGCGTAGAGATCTGGAGGAATACCGGTGGCGAAGGCG
GCCCCCTGGACAAAGACTGACGCTCAGGTGCGAAAGCGTGGGGAGCAAACAGGATTA
GATACCCTGGTAGTCCACGCTGTAAACGATGTCGACTTGGAGGTTGTGCCCTTGAGGCG
TGGCTTCCGGAGCTAACGCGTTAAGTCGACCGCCTGGGGAGTACGGCCGCAAGGTTAA
AACTCAAATGAATTGACGGGGGCCCGCACAAGCGGTGGAGCATGTGGTTTAATTCGATG
CAACGCGAAGAACCTTACCTACTCTTGACATCCACGGAATTTAGCAGAGATGCTTTAGT
GCCTTCGGGAACCGTGAGACAGGTGCTGCATGGCTGTCGCCAGCTCGTGTTGTGAAAT
GTTGGGTTAAGTCCCGCAACGAGCGCAACCCTTATCCTTTGTTGCCAGCACGTGATGGT
GGGAACTCAAAGGAGACTGCTGGTGATAAACCGGAGGAAGGTGGGGATGACGTCAAG
TCATCATGGCCCTTACGAGTAGGGCTACACACGTGCTACAATGGCAGATACAAAGTGAA
GCGAACTCGCGAGAGCAAGCGGACCACATAAAGTCTGTCGTAGTCCGGATTGGAGTCT
GCAACTCGACTCCATGAAGTCGGAATCGCTAGTAATCGTAGATCAGAATGCTACGGTGA
ATACGTTCCCGGGCCTTGTACACACCGCCCGTCACACCATGGGAGTGGGTTGCAAAAG
AAGTAGGTAGCTTAACCTTCGGGAGGGCGCTTACCACTTTGTGATTCATGACTGGGG
pattern 115

CGCTGGCGGCAGGCCTAACACATGCAAGTCGAGCGGCAGCGGGAAGTAGTTTACTACT
TTGCCGGCGAGCGGCGGACGGGTGAGTAATGTCTGGGAAACTGCCTGATGGAGGGGGA
TAACTACTGGAAACGGTAGCTAATACCGCATGACCTCGCAAGAGCAAAGTGGGGGACC
TTCGGGCCTCACGCCATCGGATGTGCCCAGATGGGATTAGCTAGTAGGTGAGGTAATGG
CTCACCTAGGCGACGATCCCTAGCTGGTCTGAGAGGATGACCAGCCACACTGGAACTG
AGACACGGTCCAGACTCCTACGGGAGGCAGCAGTGGGGAATATTGCACAATGGGCGCA
AGCCTGATGCAGCCATGCCGCGTGTGTGAAGAAGGCCTTCGGGTTGTAAAGCACTTTCA
GCGAGGAGGAAGGCAGTCGTGTTAATAGCACGGTTGATTGACGTTACTCGCAGAAGAA
GCACCGGCTAACTCCGTGCCAGCAGCCGCGGTAATACGGAGGGTGCAAGCGTTAATCG
GAATTACTGGGCGTAAAGCGCACGCAGGCGGTTTGTTAAGTCAGATGTGAAATCCCCGC
GCTTAACGTGGGAACTGCATTTGAAACTGGCAAGCTAGAGTCTTGTAGAGGGGGGTAG
AATTCCAGGTGTAGCGGTGAAATGCGTAGAGATCTGGAGGAATACCGGTGGCGAAGGC
GGCCCCCTGGACAAAGACTGACGCTCAGGTGCGAAAGCGTGGGGAGCAAACAGGATT
AGATACCCTGGTAGTCCACGCTGTAAACGATGTCGACTTGGAGGTTGTGCCCTTGAGGC
GTGGCTTCCGGAGCTAACGCGTTAAGTCGACCGCCTGGGGAGTACGGCCGCAAGGTTA
AAACTCAAATGAATTGACGGGGGCCCGCACAAGCGGTGGAGCATGTGGTTTAATTCGAT
GCAACGCGAAGAACCTTACCTACTCTTGACATCCACAGAACTTAGCAGAGATGCTTCGG
TGCCTTCGGGAACTGTGAGACAGGTGCTGCATGGCTGTCGTCAGCTCGTGTTGTGAAAT
GTTGGGTTAAGTCCCGCAACGAGCGCAACCCTTATCCTTTGTTGCCAGCACGTAATGGT
GGGAACTCAAGGGAGACTGCCGGTGACAAACCGGAGGAAGGTGGGGATGACGTCAAG
TCATCATGGCCCTTACGAGTAGGGCTACACACGTGCTACAATGGCAGATACAAAGTGAA
GCGAACTCGCGAGAGCAAGCGGACCACATAAAGTCTGTCGTAGTCCGGATTGGAGTCT
GCAACTCGACTCCATGAAGTCGGAATCGCTAGTAATCGTAGATCAGAATGCTACGGTGA
ATACGTTCCCGGGCCTTGTACACACCGCCCGTCACACCATGGGAGTGGGTTGCAAAAG
AAGTAGGTAGCTTAACCTTCGGGAGGGCGCTTACCACTTTGTGATTCATGACTGGGG
pattern 116
CGCTGGCGGCAGGCCTAACACATGCAAGTCGAGCGGCAGCGGGAAGTAGTTTACTACT
TTGCCGGCGAGCGGCGGACGGGTGAGTAATGTCTGGGGATCTGCCTGATGGAGGGGGA
TAACTACTGGAAACGGTAGCTAATACCGCGTGACCTCGCAAGAGCAAAGTGGGGGACC
TTAGGGCCTCACGCCATCGGATGAACCCAGATGGGATTAGCTAGTAGGTGGGGTAATGG
CTCACCTAGGCGACGATCCCTAGCTGGTCTGAGAGGATGACCAGCCACACTGGAACTG
AGACACGGTCCAGACTCCTACGGGAGGCAGCAGTGGGGAATATTGCACAATGGGCGCA
AGCCTGATGCAGCCATGCCGCGTGTGTGAAGAAGGCCTTCGGGTTGTAAAGCACTTTCA
GCGAGGAGGAAGGGGTTGAGTTTAATACGCTCAATCATTGACGTTACTCGCAGAAGAA
GCACCGGCTAACTCCGTGCCAGCAGCCGCGGTAATACGGAGGGTGCAAGCGTTAATCG
GAATTACTGGGCGTAAAGCGCACGCAGGCGGTTTGTTAAGTCAGATGTGAAATCCCCGC
GCTTAACGTGGGAACTGCATTTGAAACTGGCAAGCTAGAGTCTTGTAGAGGGGGGTAG
AATTCCAGGTGTAGCGGTGAAATGCGTAGAGATCTGGAGGAATACCGGTGGCGAAGGC
GGCCCCCTGGACAAAGACTGACGCTCAGGTGCGAAAGCGTGGGGAGCAAACAGGATT
AGATACCCTGGTAGTCCACGCTGTAAACGATGTCGACTTGGAGGTTGTGCCCTTGAGGC
GTGGCTTCCGGAGCTAACGCGTTAAGTCGACCGCCTGGGGAGTACGGCCGCAAGGTTA
AAACTCAAATGAATTGACGGGGGCCCGCACAAGCGGTGGAGCATGTGGTTTAATTCGAT
GCAACGCGAAGAACCTTACCTACTCTTGACATCCACAGAATTTGGCAGAGATGCTAAAG
TGCCTTCGGGAACTGTGAGACAGGTGCTGCATGGCTGTCGTCAGCTCGTGTTGTGAAAT

GTTGGGTTAAGTCCCGCAACGAGCGCAACCCTTATCCTTTGTTGCCAGCACGTAATGGT
GGGAACTCAAGGGAGACTGCCGGTGACAAACCGGAGGAAGGTGGGGATGACGTCAAG
TCATCATGGCCCTTACGAGTAGGGCTACACACGTGCTACAATGGCAGATACAAAGTGAA
GCGAACTCGCGAGAGTCAGCGGACCACATAAAGTCTGTCGTAGTCCGGATTGGAGTCT
GCAACTCGACTCCATGAAGTCGGAATCGCTAGTAATCGTAGATCAGAATGCTACGGTGA
ATACGTTCCCGGGCCTTGTACACACCGCCCGTCACACCATGGGAGTGGGTTGCAAAAG
AAGTAGGTAGCTTAACCTTCGGGAGGGCGCTTACCACTTTGTGATTCATGACTGGGG
pattern 117
CGCTGGCGGCAGGCCTAACACATGCAAGTCGAGCGGCAGCGGAAAGTAGCTTGCTACT
TTGCCGGCGAGCGGCGGACGGGTGAGTAATGTCTGGGAAACTGCCTGATGGAGGGGGA
TAACTACTGGAAACGGTAGCTAATACCGCATAACGTCTTCGGACCAAAGTGGGGGACCT
TCGGGCCTCACGCCATCGGATGTGCCCAGATGGGATTAGCTAGTAGGTGGGGTAATGGC
TCACCTAGGCGACGATCCCTAGCTGGTCTGAGAGGATGACCAGCCACACTGGAACTGA
GACACGGTCCAGACTCCTACGGGAGGCAGCAGTGGGGAATATTGCACAATGGGCGCAA
GCCTGATGCAGCCATGCCGCGTGTGTGAAGAAGGCCTTCGGGTTGTAAAGCACTTTCAG
CGAGGAGGAAGGCATAAAGGTTAATAACCTTTGTGATTGACGTTACTCGCAGAAGAAG
CACCGGCTAACTCCGTGCCAGCAGCCGCGGTAATACGGAGGGTGCAAGCGTTAATCGG
AATTACTGGGCGTAAAGCGCACGCAGGCGGTTTGTTAAGTCAGATGTGAAATCCCCGCG
CTTAACGTGGGAACTGCATTTGAAACTGGCAAGCTAGAGTCTTGTAGAGGGGGGTAGA
ATTCCAGGTGTAGCGGTGAAATGCGTAGAGATCTGGAGGAATACCGGTGGCGAAGGCG
GCCCCCTGGACAAAGACTGACGCTCAGGTGCGAAAGCGTGGGGAGCAAACAGGATTA
GATACCCTGGTAGTCCACGCTGTAAACGATGTCGACTTGGAGGTTGTGCCCTTGAGGCG
TGGCTTCCGGAGCTAACGCGTTAAGTCGACCGCCTGGGGAGTACGGCCGCAAGGTTAA
AACTCAAATGAATTGACGGGGGCCCGCACAAGCGGTGGAGCATGTGGTTTAATTCGATG
CAACGCGAAGAACCTTACCTACTCTTGACATCCACAGAACTTAGCAGAGATGCTTCGGT
GCCTTCGGGAACTGTGAGACAGGTGCTGCATGGCTGTCGTCAGCTCGTGTTGTGAAATG
TTGGGTTAAGTCCCGCAACGAGCGCAACCCTTATCCTTTGTTGCCAGCACGTCATGGTG
GGAACTCAAAGGAGACTGCCGGTGATAAACCGGAGGAAGGTGGGGATGACGTCAAGT
CATCATGGCCCTTACGAGTAGGGCTACACACGTGCTACAATGGCAGATACAAAGTGAAG
CGAACTCGCGAGAGCAAGCGGACCACATAAAGTCTGTCGTAGTCCGGATTGGAGTCTG
CAACTCGACTCCATGAAGTCGGAATCGCTAGTAATCGTAGATCAGAATGCTACGGTGAAT
ACGTTCCCGGGCCTTGTACACACCGCCCGTCACACCATGGGAGTGGGTTGCAAAAGAA
GTAGGTAGCTTAACCTTCGGGAGGGCGCTTACCACTTTGTGATTCATGACTGGGG
pattern 118
CGCTGGCGGCAGGCCTAACACATGCAAGTCGAGCGGCAGCGGAAAGTAGCTTGCTACT
TTGCCGGCGAGCGGCGGACGGGTGAGTAATGTCTGGGAAACTGCCTGATGGAGGGGGA
TAACTACTGGAAACGGTAGCTAATACCGCATAACGTCTTCGGACCAAAGTGGGGGACCT
TCGGGCCTCACGCCATCGGATGTGCCCAGATGGGATTAGCTAGTAGGTGGGGTAATGGC
TCACCTAGGCGACGATCCCTAGCTGGTCTGAGAGGATGACCAGCCACACTGGAACTGA
GACACGGTCCAGACTCCTACGGGAGGCAGCAGTGGGGAATATTGCACAATGGGCGCAA
GCCTGATGCAGCCATGCCGCGTGTGTGAAGAAGGCCTTCGGGTTGTAAAGCACTTTCAG
CGAGGAGGAAGGCATAAAGGTTAATAACCTTTGTGATTGACGTTACTCGCAGAAGAAG
CACCGGCTAACTCCGTGCCAGCAGCCGCGGTAATACGGAGGGTGCAAGCGTTAATCGG
AATTACTGGGCGTAAAGCGCACGCAGGCGGTTTGTTAAGTCAGATGTGAAATCCCCGCG

CTTAACGTGGGAACTGCATTTGAAACTGGCAAGCTAGAGTCTTGTAGAGGGGGGTAGA
ATTCCAGGTGTAGCGGTGAAATGCGTAGAGATCTGGAGGAATACCGGTGGCGAAGGCG
GCCCCCTGGACAAAGACTGACGCTCAGGTGCGAAAGCGTGGGGAGCAAACAGGATTA
GATACCCTGGTAGTCCACGCTGTAAACGATGTCGACTTGGAGGTTGTGCCCTTGAGGCG
TGGCTTCCGGAGCTAACGCGTTAAGTCGACCGCCTGGGGAGTACGGCCGCAAGGTTAA
AACTCAAATGAATTGACGGGGGCCCGCACAAGCGGTGGAGCATGTGGTTTAATTCGATG
CAACGCGAAGAACCTTACCTACTCTTGACATCCACGGAATTTAGCAGAGATGCTTTAGT
GCCTTCGGGAACCGTGAGACAGGTGCTGCATGGCTGTCGTCAGCTCGTGTTGTGAAATG
TTGGGTTAAGTCCCGCAACGAGCGCAACCCTTATCCTTTGTTGCCAGCACGTCATGGTG
GGAACTCAAAGGAGACTGCCGGTGATAAACCGGAGGAAGGTGGGGATGACGTCAAGT
CATCATGGCCCTTACGAGTAGGGCTACACACGTGCTACAATGGCAGATACAAAGTGAAG
CGAACTCGCGAGAGCAAGCGGACCACATAAAGTCTGTCGTAGTCCGGATTGGAGTCTG
CAACTCGACTCCATGAAGTCGGAATCGCTAGTAATCGTAGATCAGAATGCTACGGTGAAT
ACGTTCCCGGGCCTTGTACACACCGCCCGTCACACCATGGGAGTGGGTTGCAAAAGAA
GTAGGTAGCTTAACCTTCGGGAGGGCGCTTACCACTTTGTGATTCATGACTGGGG
pattern 119
CGCTGGCGGCAGGCCTAACACATGCAAGTCGAGCGGCAGCGGGAAGTAGTTTACTACT
TCGCCGGCGAGCGGCGGACGGGTGAGTAATGTCTGGGAAACTGCCTGATGGAGGGGGA
TAACTACTGGAAACGGTAGCTAATACCGCATAACGTCTTCGGACCAAAGTGGGGGACCT
TCGGGCCTCACGCCATCGGATGTGCCCAGATGGGATTAGCTAGTAGGTGGGGTAATGGC
TCACCTAGGCGACGATCCCTAGCTGGTCTGAGAGGATGACCAGCCACACTGGAACTGA
GACACGGTCCAGACTCCTACGGGAGGCAGCAGTGGGGAATATTGCACAATGGGCGCAA
GCCTGATGCAGCCATGCCGCGTGTGTGAAGAAGGCCTTCGGGTTGTAAAGCACTTTCAG
CGAGGAGGAAGGCATAAAGGTTAATAACCTTTGTGATTGACGTTACTCGCAGAAGAAG
CACCGGCTAACTCCGTGCCAGCAGCCGCGGTAATACGGAGGGTGCAAGCGTTAATCGG
AATTACTGGGCGTAAAGCGCACGCAGGCGGTTTGTTAAGTCAGATGTGAAATCCCCGCG
CTTAACGTGGGAACTGCATTTGAAACTGGCAAGCTAGAGTCTTGTAGAGGGGGGTAGA
ATTCCAGGTGTAGCGGTGAAATGCGTAGAGATCTGGAGGAATACCGGTGGCGAAGGCG
GCCCCCTGGACAAAGACTGACGCTCAGGTGCGAAAGCGTGGGGAGCAAACAGGATTA
GATACCCTGGTAGTCCACGCTGTAAACGATGTCGACTTGGAGGTTGTGCCCTTGAGGCG
TGGCTTCCGGAGCTAACGCGTTAAGTCGACCGCCTGGGGAGTACGGCCGCAAGGTTAA
AACTCAAATGAATTGACGGGGGCCCGCACAAGCGGTGGAGCATGTGGTTTAATTCGATG
CAACGCGAAGAACCTTACCTACTCTTGACATCCACGGAATTTAGCAGAGATGCTTTAGT
GCCTTCGGGAACCGTGAGACAGGTGCTGCATGGCTGTCGTCAGCTCGTGTTGTGAAATG
TTGGGTTAAGTCCCGCAACGAGCGCAACCCTTATCCTTTGTTGCCAGCACGTCATGGTG
GGAACTCAAAGGAGACTGCCGGTGATAAACCGGAGGAAGGTGGGGATGACGTCAAGT
CATCATGGCCCTTACGAGTAGGGCTACACACGTGCTACAATGGCAGATACAAAGTGAAG
CGAACTCGCGAGAGCAAGCGGACCACATAAAGTCTGTCGTAGTCCGGATTGGAGTCTG
CAACTCGACTCCATGAAGTCGGAATCGCTAGTAATCGTAGATCAGAATGCTACGGTGAAT
ACGTTCCCGGGCCTTGTACACACCGCCCGTCACACCATGGGAGTGGGTTGCAAAAGAA
GTAGGTAGCTTAACCTTCGGGAGGGCGCTTACCACTTTGTGATTCATGACTGGGG
pattern 120
CGCTGGCGGCAGGCCTAACACATGCAAGTCGAGCGGCAGCGGGAAGTAGTTTACTACT
TTGCCGGCGAGCGGCGGACGGGTGAGTAATGTCTGGGAAACTGCCTGATGGAGGGGGA

TAACTACTGGAAACGGTAGCTAATACCGCATAACGTCTTCGGACCAAAGTGGGGGACCT
TCGGGCCTCACGCCATCGGATGTGCCCAGATGGGATTAGCTAGTAGGTGGGGTAATGGC
TCACCTAGGCGACGATCCCTAGCTGGTCTGAGAGGATGACCAGCCACACTGGAACTGA
GACACGGTCCAGACTCCTACGGGAGGCAGCAGTGGGGAATATTGCACAATGGGCGCAA
GCCTGATGCAGCCATGCCGCGTGTGTGAAGAAGGCCTTCGGGTTGTAAAGCACTTTCAG
CGAGGAGGAAGGCATAAAGGTTAATAACCTTTGTGATTGACGTTACTCGCAGAAGAAG
CACCGGCTAACTCCGTGCCAGCAGCCGCGGTAATACGGAGGGTGCAAGCGTTAATCGG
AATTACTGGGCGTAAAGCGCACGCAGGCGGTTTGTTAAGTCAGATGTGAAATCCCCGCG
CTTAACGTGGGAACTGCATTTGAAACTGGCAAGCTAGAGTCTTGTAGAGGGGGGTAGA
ATTCCAGGTGTAGCGGTGAAATGCGTAGAGATCTGGAGGAATACCGGTGGCGAAGGCG
GCCCCCTGGACAAAGACTGACGCTCAGGTGCGAAAGCGTGGGGAGCAAACAGGATTA
GATACCCTGGTAGTCCACGCTGTAAACGATGTCGACTTGGAGGTTGTGCCCTTGAGGCG
TGGCTTCCGGAGCTAACGCGTTAAGTCGACCGCCTGGGGAGTACGGCCGCAAGGTTAA
AACTCAAATGAATTGACGGGGGCCCGCACAAGCGGTGGAGCATGTGGTTTAATTCGATG
CAACGCGAAGAACCTTACCTACTCTTGACATCCACAGAACTTAGCAGAGATGCTTCGGT
GCCTTCGGGAACTGTGAGACAGGTGCTGCATGGCTGTCGTCAGCTCGTGTTGTGAAATG
TTGGGTTAAGTCCCGCAACGAGCGCAACCCTTATCCTTTGTTGCCAGCACGTCATGGTG
GGAACTCAAAGGAGACTGCCGGTGATAAACCGGAGGAAGGTGGGGATGACGTCAAGT
CATCATGGCCCTTACGAGTAGGGCTACACACGTGCTACAATGGCAGATACAAAGTGAAG
CGAACTCGCGAGAGCAAGCGGACCACATAAAGTCTGTCGTAGTCCGGATTGGAGTCTG
CAACTCGACTCCATGAAGTCGGAATCGCTAGTAATCGTAGATCAGAATGCTACGGTGAAT
ACGTTCCCGGGCCTTGTACACACCGCCCGTCACACCATGGGAGTGGGTTGCAAAAGAA
GTAGGTAGCTTAACCTTCGGGAGGGCGCTTACCACTTTGTGATTCATGACTGGGG
pattern 121
CGCTGGCGGCAGGCCTAACACATGCAAGTCGAGCGGCAGCGGAAAGTAGCTTGCTACT
TTGCCGGCGAGCGGCGGACGGGTGAGTAATGTCTGGGAAACTGCCTGATGGAGGGGGA
TAACTACTGGAAACGGTAGCTAATACCGCATGACCTCGCAAGAGCAAAGTGGGGGACC
TTCGGGCCTCACGCCATCGGATGTGCCCAGATGGGATTAGCTAGTAGGTGGGGTAATGG
CTCACCTAGGCGACGATCCCTAGCTGGTCTGAGAGGATGACCAGCCACACTGGAACTG
AGACACGGTCCAGACTCCTACGGGAGGCAGCAGTGGGGAATATTGCACAATGGGCGCA
AGCCTGGTGCAGCCATGCCGCGTGTGTGAAGAAGGCCTTCGGGTTGTAAAGCACTTTC
AGCGAGGAGGAAGGCAGTCGTGTTAATAGCACGATTGATTGACGTTACTCGCAGAAGA
AGCACCGGCTAACTCCGTGCCAGCAGCCGCGGTAATACGGAGGGTGCAAGCGTTAATC
GGAATTACTGGGCGTAAAGCGCACGCAGGCGGTTTGTTAAGTCAGATGTGAAATCCCCG
CGCTTAACGTGGGAACTGCATTTGAAACTGGCAAGCTAGAGTCTTGTAGAGGGGGGTA
GAATTCCAGGTGTAGCGGTGAAATGCGTAGAGATCTGGAGGAATACCGGTGGCGAGGG
CGGCCCCCTGGACAAAGACTGACGCTCAGGTGCGAAAGCGTGGGGAGCAAACAGGAT
TAGATACCCTGGTAGTCCACGCTGTAAACGATGTCGACTTGGAGGTTGTGCCCTTGAGG
CGTGGCTTCCGGAGCTAACGCGTTAAGTCGACCGCCTGGGGAGTACGGCCGCAAGGTT
AAAACTCAAATGAATTGACGGGGGCCCGCACAAGCGGTGGAGCATGTGGTTTAATTCG
ATGCAACGCGAAGAACCTTACCTACTCTTGACATCCACAGAACTGAGCAGAGATGCTTA
GGTGCCTTCGGGAACTGTGAGACAGGTGCTGCATGGCTGTCGTCAGCTCGTGTTGTGA
AATGTTGGGTTAAGTCCCGCAACGAGCGCAACCCTTATCCTTTGTTGCCAGCACGTAAT
GGTGGGAACTCAAAGGAGACTGCCGGTGATAAACCGGAGGAAGGTGGGGATGACGTC

AAGTCATCATGGCCCTTACGAGTAGGGCTACACACGTGCTACAATGGCAGATACTAAGT
GAAGCGAACTCGCGAGAGCAAGCGGACCACATAAAGTCTGTCGTAGTCCGGATTGGAG
TCTGCAACTCGACTCCATGAAGTCGGAATCGCTAGTAATCGTAGATCAGAATGCTACGG
TGAATACGTTCCCGGGCCTTGTACACACCGCCCGTCACACCATGGGAGTGGGTTGCAAA
AGAAGTAGGTAGCTTAACCTTCGGGAGGGCGCTTACCACTTTGTGATTCATGACTGGGG
pattern 122
CGCTGGCGGCAGGCCTAACACATGCAAGTCGAGCGGCAGCGGGAAGTAGTTTACTACT
TTGCCGGCGAGCGGCGGACGGGTGAGTAATGTCTGGGAAACTGCCTGATGGAGGGGGA
TAACTACTGGAAACGGTAGCTAATACCGCATGACCTCGCAAGAGCAAAGTGGGGGACC
TTCGGGCCTCACGCCATCGGATGTGCCCAGATGGGATTAGCTAGTAGGTGGGGTAATGG
CTCACCTAGGCGACGATCCCTAGCTGGTCTGAGAGGATGACCAGCCACACTGGAACTG
AGACACGGTCCAGACTCCTACGGGAGGCAGCAGTGGGGAATATTGCACAATGGGCGCA
AGCCTGATGCAGCCATGCCGCGTGTGTGAAGAAGGCCTTCGGGTTGTAAAGCACTTTCA
GCGAGGAGGAAGGCAGTCGCGTTAATAGCACGATTGATTGACGTTACTCGCAGAAGAA
GCACCGGCTAACTCCGTGCCAGCAGCCGCGGTAATACGGAGGGTGCAAGCGTTAATCG
GAATTACTGGGCGTAAAGCGCACGCAGGCGGTTTGTTAAGTCAGATGTGAAATCCCCGC
GCTTAACGTGGGAACTGCATTTGGAACTGGCAAGCTAGAGTCTTGTAGAGGGGGGTAG
AATTCCAGGTGTAGCGGTGAAATGCGTAGAGATCTGGAGGAATACCGGTGGCGAAGGC
GGCCCCCTGGACAAAGACTGACGCTCAGGTGCGAAAGCGTGGGGAGCAAACAGGATT
AGATACCCTGGTAGTCCACGCTGTAAACGATGTCGACTTGGAGGTTGTGCCCTTGAGGC
GTGGCTTCCGGAGCTAACGCGTTAAGTCGACCGCCTGGGGAGTACGGCCGCAAGGTTA
AAACTCAAATGAATTGACGGGGGCCCGCACAAGCGGTGGAGCATGTGGTTTAATTCGAT
GCAACGCGAAGAACCTTACCTACTCTTGACATCCACAGAACTGAGCAGAGATGCTTAG
GTGCCTTCGGGAACTGTGAGACAGGTGCTGCATGGCTGTCGTCAGCTCGTGTTGTGAAA
TGTTGGGTTAAGTCCCGCAACGAGCGCAACCCTTATCCTTTGTTGCCAGCACGTAATGG
TGGGAACTCAAAGGAGACTGCCGGTGATAAACCGGAGGAAGGTGGGGATGACGTCAA
GTCATCATGGCCCTTACGAGTAGGGCTACACACGTGCTACAATGGCAGATACAAAGTGA
AGCGAACTCGCGAGAGCAAGCGGACCACATAAAGTCTGTCGTAGTCCGGATTGGAGTC
TGCAACTCGACTCCATGAAGTCGGAATCGCTAGTAATCGTAGATCAGAATGCTACGGTG
AATACGTTCCCGGGCCTTGTACACACCGCCCGTCACACCATGGGAGTGGGTTGCAAAA
GAAGTAGGTAGCTTAACCTTCGGGAGGGCGCTTACCACTTTGTGATTCATGACTGGGG
pattern 123
CGCTGGCGGCAGGCCTAACACATGCAAGTCGAGCGGCAGCGGGAAGTAGTTTACTACT
TTGCCGGCGAGCGGCGGACGGGTGAGTAATGTCTGGGAAACTGCCTGATGGAGGGGGA
TAACTACTGGAAACGGTAGCTAATACCGCATGACCTCGAAGAGCAAAGTGGGGGACCT
TCGGGCCTCACGCCATCGGATGTGCCCAGATGGGATTAGCTAGTAGGTGGGGTAATGGC
TCACCTAGGCGACGATCCCTAGCTGGTCTGAGAGGATGACCAGCCACACTGGAACTGA
GACACGGTCCAGACTCCTACGGGAGGCAGCAGTGGGGAATATTGCACAATGGGCGCAA
GCCTGATGCAGCCATGCCGCGTGTGTGAAGAAGGCCTTCGGGTTGTAAAGCACTTTCAG
CGAGGAGGAAGGCAGTCGTGTTAATAGCACGATTGATTGACGTTACTCGCAGAAGAAG
CACCGGCTAACTCCGTGCCAGCAGCCGCGGTAATACGGAGGGTGCAAGCGTTAATCGG
AATTACTGGGCGTAAAGCGCACGCAGGCGGTTTGTTAAGTCAGATGTGAAATCCCCGCG
CTTAACGTGGGAACTGCATTTGAAACTGGCAAGCTAGAGTCTTGTAGAGGGGGGTAGA
ATTCCAGGTGTAGCGGTGAAATGCGTAGAGATCTGGAGGAATACCGGTGGCGAAGGCG

GCCCCCTGGACAAAGACTGACGCTCAGGTGCGAAAGCGTGGGGAGCAAACAGGATTA
GATACCCTGGTAGTCCACGCTGTAAACGATGTCGACTTGGAGGTTGTGCCCTTGAGGCG
TGGCTTCCGGAGCTAACGCGTTAAGTCGACCGCCTGGGGAGTACGGCCGCAAGGTTAA
AACTCAAATGAATTGACGGGGGCCCGCACAAGCGGTGGAGCATGTGGTTTAATTCGATG
CAACGCGAAGAACCTTACCTACTCTTGACATCCACAGAACTTAGCAGAGATGCTTCGGT
GCCTTCGGGAACTGTGAGACAGGTGCTGCATGGCTGTCGTCAGCTCGTGTTGTGAAATG
TTGGGTTAAGTCCCGCAACGAGCGCAACCCTTATCCTTTGTTGCCAGCACGTAATGGTG
GGAACTCAAGGGAGACTGCCGGTGACAAACCGGAGGAAGGTGGGGATGACGTCAAGT
CATCATGGCCCTTACGAGTAGGGCTACACACGTGCTACAATGGCAGATACAAAGTGAAG
CGAACTCGCGAGAGCAAGCGGACCACATAAAGTCTGTCGTAGTCCGGATTGGAGTCTG
CAACTCGACTCCATGAAGTCGGAATCGCTAGTAATCGTAGATCAGAATGCTACGGTGAAT
ACGTTCCCGGGCCTTGTACACACCGCCCGTCACACCATGGGAGTGGGTTGCAAAAGAA
GTAGGTAGCTTAACCTTCGGGAGGGCGCTTACCACTTTGTGATTCATGACTGGGG
pattern 124
CGCTGGCGGCAGGCCTAACACATGCAAGTCGAGCGGCAGCGGGAAGTAGTTTACTACT
TTGCCGGCGAGCGGCGGACGGGTGAGTAATGTCTGGGAAACTGCCTGATGGAGGGGGA
TAACTACTGGAAACGGTAGCTAATACCGCATGACCTCGCAAGAGCAAAGTGGGGGACC
TTCGGGCCTCACGCCATCGGATGTGCCCAGATGGGATTAGCTAGTAGGTGGGGTAATGG
CTCACCTAGGCGACGATCCCTAGCTGGTCTGAGAGGATGACCAGCCACACTGGAACTG
AGACACGGTCCAGACTCCTACGGGAGGCAGCAGTGGGGAATATTGCACAATGGGCGCA
AGCCTGATGCAGCCATGCCGCGTGTGTGAAGAAGGCCTTCGGGTTGTAAAGCACTTTCA
GCGAGGAGGAAGGCAGTCGTGTTAATAGCACGATTGATTGACGTTACTCGCAGAAGAA
GCACCGGCTAACTCCGTGCCAGCAGCCGCGGTAATACGGAGGGTGCAAGCGTTAATCG
GAATTACTGGGCGTAAAGCGCACGCAGGCGGTTTGTTAAGTCAGATGTGAAATCCCCGC
GCTTAACGTGGGAACTGCATTTGAAACTGGCAAGCTAGAGTCTTGTAGAGGGGGGTAG
AATTCCAGGTGTAGCGGTGAAATGCGTAGAGATCTGGAGGAATACCGGTGGCGAAGGC
GGCCCCCTGGACAAAGACTGACGCTCAGGTGCGAAAGCGTGGGGAGCAAACAGGATT
AGATACCCTGGTAGTCCACGCTGTAAACGATGTCGACTTGGAGGTTGTGCCCTTGAGGC
GTGGCTTCCGGAGCTAACGCGTTAAGTCGACCGCCTGGGGAGTACGGCCGCAGGTTAA
AACTCAAATGAATTGACGGGGGCCCGCACAAGCGGTGGAGCATGTGGTTTAATTCGATG
CAACGCGAAGAACCTTACCTACTCTTGACATCCACAGAACTTAGCAGAGATGCTTCGGT
GCCTTCGGGAACTGTGAGACAGGTGCTGCATGGCTGTCGTCAGCTCGTGTTGTGAAATG
TTGGGTTAAGTCCCGCAACGAGCGCAACCCTTATCCTTTGTTGCCAGCACGTAATGGTG
GGAACTCAAGGGAGACTGCCGGTGACAAACCGGAGGAAGGTGGGGATGACGTCAAGT
CATCATGGCCCTTACGAGTAGGGCTACACACGTGCTACAATGGCAGATACAAAGTGGAG
CGAACTCGCGAGAGCAAGCGGACCACATAAAGTCTGTCGTAGTCCGGATTGGAGTCTG
CAACTCGACTCCATGAAGTCGGAATCGCTAGTAATCGTAGATCAGAATGCTACGGTGAAT
ACGTTCCCGGGCCTTGTACACACCGCCCGTCACACCATGGGAGTGGGTTGCAAAAGAA
GTAGGTAGCTTAACCTTCGGGAGGGCGCTTACCACTTTGTGATTCATGACTGGGG
pattern 125
CGCTGGCGGCAGGCCTAACACATGCAAGTCGAGCGGCAGCGGAAAGTAGCTTGCTACT
TTGCCGGCGAGCGGCGGACGGGTGAGTAATGTCTGGGAAACTGCCTGATGGAGGGGGA
TAACTACTGGAAACGGTAGCTAATACCGCATAACGTCTTCGGACCAAAGTGGGGGACCT
TCGGGCCTCACGCCATCGGATGTGCCCAGATGGGATTAGCTAGTAGGTGGGGTAATGGC

TCACCTAGGCGACGATCCCTAGCTGGTCTGAGAGGATGACCAGCCACACTGGAACTGA
GACACGGTCCAGACTCCTACGGGAGGCAGCAGTGGGGAATATTGCACAATGGGCGCAA
GCCTGATGCAGCCATGCCGCGTGTGTGAAGAAGGCCTTCGGGTTGTAAAGCACTTTCAG
CGAGGAGGAAGGCATAAAGGTTAATAACCTTTGTGATTGACGTTACTCGCAGAAGAAG
CACCGGCTAACTCCGTGCCAGCAGCCGCGGTAATACGGAGGGTGCAAGCGTTAATCGG
AATTACTGGGCGTAAAGCGCACGCAGGCGGTTTGTTAAGTCAGATGTGAAATCCCCGCG
CTTAACGTGGGAACTGCATTTGAAACTGGCAAGCTAGAGTCTTGTAGAGGGGGGTAGA
ATTCCAGGTGTAGCGGTGAAATGCGTAGAGATCTGGAGGAATACCGGTGGCGAAGGCG
GCCCCCTGGACAAAGACTGACGCTCAGGTGCGAAAGCGTGGGGAGCAAACAGGATTA
GATACCCTGGTAGTCCACGCTGTAAACGATGTCGACTTGGAGGTTGTGCCCTTGAGGCG
TGGCTTCCGGAGCTAACGCGTTAAGTCGACCGCCTGGGGAGTACGGCCGCAAGGTTAA
AACTCAAATGAATTGACGGGGGCCCGCACAAGCGGTGGAGCATGTGGTTTAATTCGATG
CAACGCGAAGAACCTTACCTACTCTTGACATCCACGGAATTTAGCAGAGATGCTTTAGT
GCCTTCGGGAACTGTGAGACAGGTGCTGCATGGCTGTCGTCAGCTCGTGTTGTGAAATG
TTGGGTTAAGTCCCGCAACGAGCGCAACCCTTATCCTTTGTTGCCAGCACGTCATGGTG
GGAACTCAAAGGAGACTGCCGGTGATAAACCGGAGGAAGGTGGGGATGACGTCAAGT
CATCATGGCCCTTACGAGTAGGGCTACACACGTGCTACAATGGCAGATACAAAGTGAAG
CGAACTCGCGAGAGCAAGCGGACCACATAAAGTCTGTCGTAGTCCGGATTGGAGTCTG
CAACTCGACTCCATGAAGTCGGAATCGCTAGTAATCGTAGATCAGAATGCTACGGTGAAT
ACGTTCCCGGGCCTTGTACACACCGCCCGTCACACCATGGGAGTGGGTTGCAAAAGAA
GTAGGTAGCTTAACCTTCGGGAGGGCGCTTACCACTTTGTGATTCATGACTGGGG
pattern 126
CGCTGGCGGCAGGCCTAACACATGCAAGTCGAGCGGCAGCGGGAAGTAGTTTACTACT
TCGCCGGCGAGCGGCGGACGGGTGAGTAATGTCTGGGAAACTGCCTGATGGAGGGGGA
TAACTACTGGAAACGGTAGCTAATACCGCATAACGTCTTCGGACCAAAGTGGGGGACCT
TCGGGCCTCACGCCATCGGATGTGCCCAGATGGGATTAGCTAGTAGGTGGGGTAATGGC
TCACCTAGGCGACGATCCCTAGCTGGTCTGAGAGGATGACCAGCCACACTGGAACTGA
GACACGGTCCAGACTCCTACGGGAGGCAGCAGTGGGGAATATTGCACAATGGGCGCAA
GCCTGATGCAGCCATGCCGCGTGTGTGAAGAAGGCCTTCGGGTTGTAAAGCACTTTCAG
CGAGGAGGAAGGCATAAAGGTTAATAACCTTTGTGATTGACGTTACTCGCAGAAGAAG
CACCGGCTAACTCCGTGCCAGCAGCCGCGGTAATACGGAGGGTGCAAGCGTTAATCGG
AATTACTGGGCGTAAAGCGCACGCAGGCGGTTTGTTAAGTCAGATGTGAAATCCCCGCG
CTTAACGTGGGAACTGCATTTGAAACTGGCAAGCTAGAGTCTTGTAGAGGGGGGTAGA
ATTCCAGGTGTAGCGGTGAAATGCGTAGAGATCTGGAGGAATACCGGTGGCGAAGGCG
GCCCCCTGGACAAAGACTGACGCTCAGGTGCGAAAGCGTGGGGAGCAAACAGGATTA
GATACCCTGGTAGTCCACGCTGTAAACGATGTCGACTTGGAGGTTGTGCCCTTGAGGCG
TGGCTTCCGGAGCTAACGCGTTAAGTCGACCGCCTGGGGAGTACGGCCGCAAGGTTAA
AACTCAAATGAATTGACGGGGGCCCGCACAAGCGGTGGAGCATGTGGTTTAATTCGATG
CAACGCGAAGAACCTTACCTACTCTTGACATCCACGGAATTTAGCAGAGATGCTTTAGT
GCCTTCGGGAACTGTGAGACAGGTGCTGCATGGCTGTCGTCAGCTCGTGTTGTGAAATG
TTGGGTTAAGTCCCGCAACGAGCGCAACCCTTATCCTTTGTTGCCAGCACGTCATGGTG
GGAACTCAAAGGAGACTGCCGGTGATAAACCGGAGGAAGGTGGGGATGACGTCAAGT
CATCATGGCCCTTACGAGTAGGGCTACACACGTGCTACAATGGCAGATACAAAGTGAAG
CGAACTCGCGAGAGCAAGCGGACCACATAAAGTCTGTCGTAGTCCGGATTGGAGTCTG

CAACTCGACTCCATGAAGTCGGAATCGCTAGTAATCGTAGATCAGAATGCTACGGTGAAT
ACGTTCCCGGGCCTTGTACACACCGCCCGTCACACCATGGGAGTGGGTTGCAAAAGAA
GTAGGTAGCTTAACCTTCGGGAGGGCGCTTACCACTTTGTGATTCATGACTGGGG
pattern 127
CGCTGGCGGCAGGCCTAACACATGCAAGTCGAGCGGCAGCGGGAAGTAGCTTGCTACT
TTGCCGGCGAGCGGCGGACGGGTGAGTAATGTCTGGGAAACTGCCTGATGGAGGGGGA
TAACTACTGGAAACGGTAGCTAATACCGCATGACCTCGAAAGAGCAAAGTGGGGGACC
TTCGGGCCTCACGCCATCGGATGTGCCCAGATGGGATTAGCTAGTAGGTGGGGTAATGG
CTCACCTAGGCGACGATCCCTAGCTGGTCTGAGAGGATGACCAGCCACACTGGAACTG
AGACACGGTCCAGACTCCTACGGGAGGCAGCAGTGGGGAATATTGCACAATGGGCGCA
AGCCTGATGCAGCCATGCCGCGTGTGTGAAGAAGGCCTTCGGGTTGTAAAGCACTTTCA
GCGAGGAGGAAGGCATTTCACTTAATACGTGAGGTGATTGACGTTACTCGCAGAAGAA
GCACCGGCTAACTCCGTGCCAGCAGCCGCGGTAATACGGAGGGTGCAAGCGTTAATCG
GAATTACTGGGCGTAAAGCGCACGCAGGCGGTTTGTTAAGTCAGATGTGAAATCCCCGA
GCTTAACTTGGGAACTGCATTTGAAACTGGCAAGCTAGAGTCTTGTAGAGGGGGGTAG
AATTCCAGGTGTAGCGGTGAAATGCGTAGAGATCTGGAGGAATACCGGTGGCGAAGGC
GGCCCCCTGGACAAAGACTGACGCTCAGGTGCGAAAGCGTGGGGAGCAAACAGGATT
AGATACCCTGGTAGTCCACGCTGTAAACGATGTCGACTTGGAGGTTGTGCCCTTGAGGC
GTGGCTTCCGGAGCTAACGCGTTAAGTCGACCGCCTGGGGAGTACGGCCGCAAGGTTA
AAACTCAAATGAATTGACGGGGGCCCGCACAAGCGGTGGAGCATGTGGTTTAATTCGAT
GCAACGCGAAGAACCTTACCTACTCTTGACATCCACAGAACTTAGCAGAGATGCTTAGG
TGCCTTCGGGAACTGTGAGACAGGTGCTGCATGGCTGTCGTCAGCTCGTGTTGTGAAAT
GTTGGGTTAAGTCCCGCAACGAGCGCAACCCTTATCCTTTGTTGCCAGCACGTAATGGT
GGGAACTCAAAGGAGACTGCCGGTGATAAACCGGAGGAAGGTGGGGATGACGTCAAG
TCATCATGGCCCTTACGAGTAGGGCTACACACGTGCTACAATGGCAGATACAAAGTGAA
GCGAACTCGCGAGAGCAAGCGGACCACATAAAGTCTGTCGTAGTCCGGATTGGAGTCT
GCAACTCGACTCCATGAAGTCGGAATCGCTAGTAATCGTAGATCAGAATGCTACGGTGA
ATACGTTCCCGGGCCTTGTACACACCGCCCGTCACACCATGGGAGTGGGTTGCAAAAG
AAGTAGGTAGCTTAACCTTCGGGAGGGCGCTTACCACTTTGTGATTCATGACTGGGG
pattern 128
CGCTGGCGGCAGGCCTAACACATGCAAGTCGAGCGGCAGCGGAAAGTAGCTTGCTACT
TTGCCGGCGAGCGGCGGACGGGTGAGTAATGTCTGGGAAACTGCCTGATGGAGGGGGA
TAACTACTGGAAACGGTAGCTAATACCGCATGACCTCGAAAGAGCAAAGTGGGGGACC
TTCGGGCCTCACGCCATCGGATGTGCCCAGATGGGATTAGCTAGTAGGTGGGGTAATGG
CTCACCTAGGCGACGATCCCTAGCTGGTCTGAGAGGATGACCAGCCACACTGGAACTG
AGACACGGTCCAGACTCCTACGGGAGGCAGCAGTGGGGAATATTGCACAATGGGCGCA
AGCCTGATGCAGCCATGCCGCGTGTGTGAAGAAGGCCTTCGGGTTGTAAAGCACTTTCA
GCGAGGAGGAAGGCATCTTACTTAATACGTGAAGTGATTGACGTTACTCGCAGAAGAA
GCACCGGCTAACTCCGTGCCAGCAGCCGCGGTAATACGGAGGGTGCAAGCGTTAATCG
GAATTACTGGGCGTAAAGCGCACGCAGGCGGTTTGTTAAGTCAGATGTGAAATCCCCGA
GCTTAACTTGGGAACTGCATTTGAAACTGGCAAGCTAGAGTCTTGTAGAGGGGGGTAG
AATTCCAGGTGTAGCGGTGAAATGCGTAGAGATCTGGAGGAATACCGGTGGCGAAGGC
GGCCCCCTGGACAAAGACTGACGCTCAGGTGCGAAAGCGTGGGGAGCAAACAGGATT
AGATACCCTGGTAGTCCACGCTGTAAACGATGTCGACTTGGAGGTTGTGCCCTTGAGGC

GTGGCTTCCGGAGCTAACGCGTTAAGTCGACCGCCTGGGGAGTACGGCCGCAAGGTTA
AAACTCAAATGAATTGACGGGGGCCCGCACAAGCGGTGGAGCATGTGGTTTAATTCGAT
GCAACGCGAAGAACCTTACCTACTCTTGACATCCACAGAACTTAGCAGAGATGCTTAGG
TGCCTTCGGGAACTGTGAGACAGGTGCTGCATGGCTGTCGTCAGCTCGTGTTGTGAAAT
GTTGGGTTAAGTCCCGCAACGAGCGCAACCCTTATCCTTTGTTGCCAGCACGTAATGGT
GGGAACTCAAAGGAGACTGCCGGTGATAAACCGGAGGAAGGTGGGGATGACGTCAAG
TCATCATGGCCCTTACGAGTAGGGCTACACACGTGCTACAATGGCAGATACAAAGTGAA
GCGAACTCGCGAGAGCAAGCGGACCACATAAAGTCTGTCGTAGTCCGGATTGGAGTCT
GCAACTCGACTCCATGAAGTCGGAATCGCTAGTAATCGTAGATCAGAATGCTACGGTGA
ATACGTTCCCGGGCCTTGTACACACCGCCCGTCACACCATGGGAGTGGGTTGCAAAAG
AAGTAGGTAGCTTAACCTTCGGGAGGGCGCTTACCACTTTGTGATTCATGACTGGGG
pattern 129
CGCTGGCGGCAGGCCTAACACATGCAAGTCGAGCGGCAGCGGAAAGTAGCTTGCTACT
TTGCCGGCGAGCGGCGGACGGGTGAGTAATGTCTGGGAAACTGCCTGATGGAGGGGGA
TAACTACTGGAAACGGTAGCTAATACCGCATGACCTCGAAAGAGCAAAGTGGGGGACC
TTCGGGCCTCACGCCATCGGATGTGCCCAGATGGGATTAGCTAGTAGGTGGGGTAATGG
CTCACCTAGGCGACGATCCCTAGCTGGTCTGAGAGGATGACCAGCCACACTGGAACTG
AGACACGGTCCAGACTCCTACGGGAGGCAGCAGTGGGGAATATTGCACAATGGGCGCA
AGCCTGATGCAGCCATGCCGCGTGTGTGAAGAAGGCCTTCGGGTTGTAAAGCACTTTCA
GCGAGGAGGAAGGCATCTTACTTAATACGTGAGGTGATTGACGTTACTCGCAGAAGAA
GCACCGGCTAACTCCGTGCCAGCAGCCGCGGTAATACGGAGGGTGCAAGCGTTAATCG
GAATTACTGGGCGTAAAGCGCACGCAGGCGGTTTGTTAAGTCAGATGTGAAATCCCCGA
GCTTAACTTGGGAACTGCATTTGAAACTGGCAAGCTAGAGTCTTGTAGAGGGGGGTAG
AATTCCAGGTGTAGCGGTGAAATGCGTAGAGATCTGGAGGAATACCGGTGGCGAAGGC
GGCCCCCTGGACAAAGACTGACGCTCAGGTGCGAAAGCGTGGGGAGCAAACAGGATT
AGATACCCTGGTAGTCCACGCTGTAAACGATGTCGACTTGGAGGTTGTGCCCTTGAGGC
GTGGCTTCCGGAGCTAACGCGTTAAGTCGACCGCCTGGGGAGTACGGCCGCAAGGTTA
AAACTCAAATGAATTGACGGGGGCCCGCACAAGCGGTGGAGCATGTGGTTTAATTCGAT
GCAACGCGAAGAACCTTACCTACTCTTGACATCCACAGAACTTAGCAGAGATGCTTAGG
TGCCTTCGGGAACTGTGAGACAGGTGCTGCATGGCTGTCGTCAGCTCGTGTTGTGAAAT
GTTGGGTTAAGTCCCGCAACGAGCGCAACCCTTATCCTTTGTTGCCAGCACGTAATGGT
GGGAACTCAAAGGAGACTGCCGGTGATAAACCGGAGGAAGGTGGGGATGACGTCAAG
TCATCATGGCCCTTACGAGTAGGGCTACACACGTGCTACAATGGCAGATACAAAGTGAA
GCGAACTCGCGAGAGCAAGCGGACCACATAAAGTCTGTCGTAGTCCGGATTGGAGTCT
GCAACTCGACTCCATGAAGTCGGAATCGCTAGTAATCGTAGATCAGAATGCTACGGTGA
ATACGTTCCCGGGCCTTGTACACACCGCCCGTCACACCATGGGAGTGGGTTGCAAAAG
AAGTAGGTAGCTTAACCTTCGGGAGGGCGCTTACCACTTTGTGATTCATGACTGGGG
pattern 130
CGCTGGCGGCAGGCCTAACACATGCAAGTCGAGCGGCAGCGGGAAGTAGTTTACTACT
TTGCCGGCGAGCGGCGGACGGGTGAGTAATGTCTGGGAAACTGCCTGATGGAGGGGGA
TAACTACTGGAAACGGTAGCTAATACCGCATAACGTCTTCGGACCAAAGTGGGGGACCT
TAGGGCCTCACGCCATCGGATGTGCCCAGATGGGATTAGCTAGTAGGTGGGGTAATGGC
TCACCTAGGCGACGATCCCTAGCTGGTCTGAGAGGATGACCAGCCACACTGGAACTGA
GACACGGTCCAGACTCCTACGGGAGGCAGCAGTGGGGAATATTGCACAATGGGCGCAA

GCCTGATGCAGCCATGCCGCGTGTGTGAAGAAGGCCTTCGGGTTGTAAAGCACTTTCAG
CGAGGAGGAAGGCCAATAACTTAATACGTTGTTGGATTGACGTTACTCGCAGAAGAAGC
ACCGGCTAACTCCGTGCCAGCAGCCGCGGTAATACGGAGGGTGCAAGCGTTAATCGGA
ATTACTGGGCGTAAAGCGCACGCAGGCGGTTTGTTAAGTCAGATGTGAAATCCCCGCGC
TTAACATGGGAACTGCATTTGAAACTGGCAAGCTAGAGTCTTGTAGAGGGGGGTAGAAT
TCCAGGTGTAGCGGCGAAATGCGTAGAGATCTGGAGGAATACCGGTGGCGAAGGCGGC
CCCCTGGACAAAGACTGACGCTCAGGTGCGAAAGCGTGGGGAGCAAACAGGATTAGAT
ACCCTGGTAGTCCACGCTGTAAACGATGTCGACTTGGAGGTTGTGCCCTTGAGGCGTGG
CTTCCGGAGCTAACGCGTTAAGTCGACCGCCTGGGGAGTACGGCCGCAAGGTTAAAAC
TCAAATGAATTGACGGGGGCCCGCACAAGCGGTGGAGCATGTGGTTTAATTCGATGCAA
CGCGAAGAACCTTACCTACTCTTGACATCCACGGAATTTAGCAGAGATGCTTTAGTGCC
TTCGGGAACCGTGAGACAGGTGCTGCATGGCTGTCGTCAGCTCGTGTTGTGAAATGTTG
GGTTAAGTCCCGCAACGAGCGCAACCCTTATCCTTTGTTGCCAGCACGTAATGGTGGGA
ACTCAAAGGAGACTGCCGGTGATAAACCGGAGGAAGGTGGGGATGACGTCAAGTCATC
ATGGCCCTTACGAGTAGGGCTACACACGTGCTACAATGGCAGATACAAAGTGAAGCGA
ACTCGCGAGAGCAAGCGGACCACATAAAGTCTGTCGTAGTCCGGATTGGAGTCTGCAA
CTCGACTCCATGAAGTCGGAATCGCTAGTAATCGTAGATCAGAATGCTACGGTGAATACG
TTCCCGGGCCTTGTACACACCGCCCGTCACACCATGGGAGTGGGTTGCAAAAGAAGTA
GGTAGCTTAACCTTCGGGAGGGCGCTTACCACTTTGTGATTCATGACTGGGG
pattern 131
CGCTGGCGGCAGGCCTAACACATGCAAGTCGAGCGGCAGCGGGAAGTAGTTTACTACT
TTGCCGGCGAGCGGCGGACGGGTGAGTAATGTCTGGGAAACTGCCTGATGGAGGGGGA
TAACTACTGGAAACGGTAGCTAATACCGCATAACGTCTTCGGACCAAAGTGGGGGACCT
TAGGGCCTCACGCCATCGGATGTGCCCAGATGGGATTAGCTAGTAGGTGGGGTAATGGC
TCACCTAGGCGACGATCCCTAGCTGGTCTGAGAGGATGACCAGCCACACTGGAACTGA
GACACGGTCCAGACTCCTACGGGAGGCAGCAGTGGGGAATATTGCACAATGGGCGCAA
GCCTGATGCAGCCATGCCGCGTGTGTGAAGAAGGCCTTCGGGTTGTAAAGCACTTTCAG
CGAGGAGGAAGGCCAATAACTTAATACGTTGTTGGATTGACGTTACTCGCAGAAGAAGC
ACCGGCTAACTCCGTGCCAGCAGCCGCGGTAATACGGAGGGTGCAAGCGTTAATCGGA
ATTACTGGGCGTAAAGCGCACGCAGGCGGTTTGTTAAGTCAGATGTGAAATCCCCGCGC
TTAACGTGGGAACTGCATTTGAAACTGGCAAGCTAGAGTCTTGTAGAGGGGGGTAGAA
TTCCAGGTGTAGCGGTGAAATGCGTAGAGATCTGGAGGAATACCGGTGGCGAAGGCGG
CCCCCTGGACAAAGACTGACGCTCAGGTGCGAAAGCGTGGGGAGCAAACAGGATTAG
ATACCCTGGTAGTCCACGCTGTAAACGATGTCGACTTGGAGGTTGTGCCCTTGAGGCGT
GGCTTCCGGAGCTAACGCGTTAAGTCGACCGCCTGGGGAGTACGGCCGCAAGGTTAAA
ACTCAAATGAATTGACGGGGGCCCGCACAAGCGGTGGAGCATGTGGTTTAATTCGATGC
AACGCGAAGAACCTTACCTACTCTTGACATCCACAGAACTTAGCAGAGATGCTTCGGTG
CCTTCGGGAACTGTGAGACAGGTGCTGCATGGCTGTCGTCAGCTCGTGTTGTGAAATGT
TGGGTTAAGTCCCGCAACGAGCGCAACCCTTATCCTTTGTTGCCAGCACGTAATGGTGG
GAACTCAAAGGAGACTGCCGGTGATAAACCGGAGGAAGGTGGGGATGACGTCAAGTC
ATCATGGCCCTTACGAGTAGGGCTACACACGTGCTACAATGGCAGATACAAAGTGAAGC
GAACTCGCGAGAGCAAGCGGACCACATAAAGTCTGTCGTAGTCCGGATTGGAGTCTGC
AACTCGACTCCATGAAGTCGGAATCGCTAGTAATCGTAGATCAGAATGCTACGGTGAAT
ACGTTCCCGGGCCTTGTACACACCGCCCGTCACACCATGGGAGTGGGTTGCAAAAGAA

GTAGGTAGCTTAACCTTCGGGAGGGCGCTTACCACTTTGTGATTCATGACTGGGG
pattern 132
CGCTGGCGGCAGGCCTAACACATGCAAGTCGAGCGGCAGCGGGAAGTAGTTTACTACT
TTGCCGGCGAGCGGCGGACGGGTGAGTAATGTCTGGGAAACTGCCTGATGGAGGGGGA
TAACTACTGGAAACGGTAGCTAATACCGCATGATCTCGAAAGAGCAAAGTGGGGGACCT
TCGGGCCTCACGCCATCGGATGTGCCCAGATGGGATTAGCTAGTAGGTGGGGTAATGGC
TCACCTAGGCGACGATCCCTAGCTGGTCTGAGAGGATGACCAGCCACACTGGAACTGA
GACACGGTCCAGACTCCTACGGGAGGCAGCAGTGGGGAATATTGCACAATGGGCGCAA
GCCTGATGCAGCCATGCCGCGTGTGTGAAGAAGGCCTTCGGGTTGTAAAGCACTTTCAG
CGAGGAGGAAGGCAGTCGTGTTAATAGCACGATTGATTGACGTTACTCGCAGAAGAAG
CACCGGCTAACTCCGTGCCAGCAGCCGCGGTAATACGGAGGGTGCAAGCGTTAATCGG
AATTACTGGGCGTAAAGCGCACGCAGGCGGTTTGTTAAGTCAGATGTGAAATCCCCGCG
CTTAACGTGGGAACTGCATTTGAAACTGGCAAGCTAGAGTCTTGTAGAGGGGGGTAGA
ATTCCAGGTGTAGCGGTGAAATGCGTAGAGATCTGGAGGAATACCGGTGGCGAAGGCG
GCCCCCTGGACAAAGACTGACGCTCAGGTGCGAAAGCGTGGGGAGCAAACAGGATTA
GATACCCTGGTAGTCCACGCTGTAAACGATGTCGACTTGGAGGTTGTGCCCTTGAGGCG
TGGCTTCCGGAGCTAACGCGTTAAGTCGACCGCCTGGGGAGTACGGCCGCAAGGTTAA
AACTCAAATGAATTGACGGGGGCCCGCACAAGCGGTGGAGCATGTGGTTTAATTCGATG
CAACGCGAAGAACCTTACCTACTCTTGACATCCACAGAACTTAGCAGAGATGCTTCGGT
GCCTTCGGGAACTGTGAGACAGGTGCTGCATGGCTGTCGTCAGCTCGTGTTGTGAAATG
TTGGGTTAAGTCCCGCAACGAGCGCAACCCTTATCCTTTGTTGCCAGCGAGTAATGTCG
GGAACTCAAAGGAGACTGCCGGTGATAAACCGGAGGAAGGTGGGGATGACGTCAAGT
CATCATGGCCCTTACGAGTAGGGCTACACACGTGCTACAATGGCAGATACAAAGTGAAG
CGAACTCGCGAGAGCAAGCGGACCACATAAAGTCTGTCGTAGTCCGGATTGGAGTCTG
CAACTCGACTCCATGAAGTCGGAATCGCTAGTAATCGTAGATCAGAATGCTACGGTGAAT
ACGTTCCCGGGCCTTGTACACACCGCCCGTCACACCATGGGAGTGGGTTGCAAAAGAA
GTAGGTAGCTTAACCTTCGGGAGGGCGCTTACCACTTTGTGATTCATGACTGGGG
pattern 133
CGCTGGCGGCAGGCCTAACACATGCAAGTCGAGCGGCAGCGGGAAGTAGTTTACTACT
TTGCCGGCGAGCGGCGGACGGGTGAGTAATGTCTGGGAAACTGCCTGATGGAGGGGGA
TAACTACTGGAAACGGTAGCTAATACCGCATGACCTCGCAAGAGCAAAGTGGGGGACC
TTCGGGCCTCACGCCATCGGATGTGCCCAGATGGGATTAGCTAGTAGGTGGGGTAATGG
CTCACCTAGGCGACGATCCCTAGCTGGTCTGAGAGGATGACCAGCCACACTGGAACTG
AGACACGGTCCAGACTCCTACGGGAGGCAGCAGTGGGGAATATTGCACAATGGGCGCA
AGCCTGATGCAGCCATGCCGCGTGTGTGAAGAAGGCCTTCGGGTTGTAAAGCACTTTCA
GCGAGGAGGAAGGGTTCAGTGTTAATAGCACTGTGCATTGACGTTACTCGCAGAAGAA
GCACCGGCTAACTCCGTGCCAGCAGCCGCGGTAATACGGAGGGTGCAAGCGTTAATCG
GAATTACTGGGCGTAAAGCGCACGCAGGCGGTTTGTTAAGTCAGATGTGAAATCCCCGC
GCTTAACGTGGGAACTGCATTTGAAACTGGCAAGCTAGAGTCTTGTAGAGGGGGGTAG
AATTCCAGGTGTAGCGGTGAAATGCGTAGAGATCTGGAGGAATACCGGTGGCGAAGGC
GGCCCCCTGGACAAAGACTGACGCTCAGGTGCGAAAGCGTGGGGAGCAAACAGGATT
AGATACCCTGGTAGTCCACGCTGTAAACGATGTCGACTTGGAGGTTGTGCCCTTGAGGC
GTGGCTTCCGGAGCTAACGCGTTAAGTCGACCGCCTGGGGAGTACGGCCGCAAGGTTA
AAACTCAAATGAATTGACGGGGGCCCGCACAAGCGGTGGAGCATGTGGTTTAATTCGAT

GCAACGCGAAGAACCTTACCTACTCTTGACATCCACAGAACTTAGCAGAGATGCTTAGG
TGCCTTCGGGAACTGTGAGACAGGTGCTGCATGGCTGTCGTCAGCTCGTGTTGTGAAAT
GTTGGGTTAAGTCCCGCAACGAGCGCAACCCTTATCCTTTGTTGCCAGCACGTAATGGT
GGGAACTCAAGGGAGACTGCCGGTGACAAACCGGAGGAAGGTGGGGATGACGTCAAG
TCATCATGGCCCTTACGAGTAGGGCTACACACGTGCTACAATGGCAGATACAAAGTGAA
GCGAACTCGCGAGAGCAAGCGGACCACATAAAGTCTGTCGTAGTCCGGATTGGAGTCT
GCAACTCGACTCCATGAAGTCGGAATCGCTAGTAATCGTAGATCAGAATGCTACGGTGA
ATACGTTCCCGGGCCTTGTACACACCGCCCGTCACACCATGGGAGTGGGTTGCAAAAG
AAGTAGGTAGCTTAACCTTCGGGAGGGCGCTTACCACTTTGTGATTCATGACTGGGG
pattern 134
CGCTGGCGGCAGGCCTAACACATGCAAGTCGAGCGGCAGCGGGAAGTAGTTTACTACT
TTGCCGGCGAGCGGCGGACGGGTGAGTAATGTCTGGGAAACTGCCTGATGGAGGGGGA
TAACTACTGGAAACGGTAGCTAATACCGCATGACCTCGCAAGAGCAAAGTGGGGGACC
TTCGGGCCTCACGCCATCGGATGTGCCCAGATGGGATTAGCTAGTAGGTGGGGTAATGG
CTCACCTAGGCAACGATCCCTAGCTGGTCTGAGAGGATGACCAGCCACACTGGAACTG
AGACACGGTCCAGACTCCTACGGGAGGCAGCAGTGGGGAATATTGCACAATGGGCGCA
AGCCTGATGCAGCCATGCCGCGTGTGTGAAGAAGGCCTTCGGGTTGTAAAGCACTTTCA
GCGAGGAGGAAGGGTTCAGTGTTAATAGCACTGTGCATTGACGTTACTCGCAGAAGAA
GCACCGGCTAACTCCGTGCCAGCAGCCGCGGTAATACGGAGGGTGCAAGCGTTAATCG
GAATTACTGGGCGTAAAGCGCACGCAGGCGGTTTGTTAAGTCAGATGTGAAATCCCCGC
GCTTAACGTGGGAACTGCATTTGAAACTGGCAAGCTAGAGTCTTGTAGAGGGGGGTAG
AATTCCAGGTGTAGCGGTGAAATGCGTAGAGATCTGGAGGAATACCGGTGGCGAAGGC
GGCCCCCTGGACAAAGACTGACGCTCAGGTGCGAAAGCGTGGGGAGCAAACAGGATT
AGATACCCTGGTAGTCCACGCTGTAAACGATGTCGACTTGGAGGTTGTGCCCTTGAGGC
GTGGCTTCCGGAGCTAACGCGTTAAGTCGACCGCCTGGGGAGTACGGCCGCAAGGTTA
AAACTCAAATGAATTGACGGGGGCCCGCACAAGCGGTGGAGCATGTGGTTTAATTCGAT
GCAACGCGAAGAACCTTACCTACTCTTGACATCCACAGAACTTAGCAGAGATGCTTAGG
TGCCTTCGGGAACTGTGAGACAGGTGCTGCATGGCTGTCGTCAGCTCGTGTTGTGAAAT
GTTGGGTTAAGTCCCGCAACGAGCGCAACCCTTATCCTTTGTTGCCAGCACGTAATGGT
GGGAACTCAAGGGAGACTGCCGGTGACAAACCGGAGGAAGGTGGGGATGACGTCAAG
TCATCATGGCCCTTACGAGTAGGGCTACACACGTGCTACAATGGCAGATACAAAGTGAA
GCGAACTCGCGAGAGCAAGCGGACCACATAAAGTCTGTCGTAGTCCGGATTGGAGTCT
GCAACTCGACTCCATGAAGTCGGAATCGCTAGTAATCGTAGATCAGAATGCTACGGTGA
ATACGTTCCCGGGCCTTGTACACACCGCCCGTCACACCATGGGAGTGGGTTGCAAAAG
AAGTAGGTAGCTTAACCTTCGGGAGGGCGCTTACCACTTTGTGATTCATGACTGGGG
pattern 135
CGCTGGCGGCAGGCCTAACACATGCAAGTCGAGCGGCAGCGGGAAGTAGTTTACTACT
TTGCCGGCGAGCGGCGGACGGGTGAGTAATGTCTGGGAAACTGCCTGATGGAGGGGGA
TAACTACTGGAAACGGTAGCTAATACCGCATGATCTCGAAAGAGCAAAGTGGGGGACCT
TCGGGCCTCACGCCATCGGATGTGCCCAGATGGGATTAGCTAGTAGGTGGGGTAACGGC
TCACCTAGGCGACGATCCCTAGCTGGTCTGAGAGGATGACCAGCCACACTGGAACTGA
GACACGGTCCAGACTCCTACGGGAGGCAGCAGTGGGGAATATTGCACAATGGGCGCAA
GCCTGATGCAGCCATGCCGCGTGTGTGAAGAAGGCCTTCGGGTTGTAAAGCACTTTCAG
CGAGGAGGAAGGCAGTCGTGTTAATAGCACGATTGATTGACGTTACTCGCAGAAGAAG

CACCGGCTAACTCCGTGCCAGCAGCCGCGGTAATACGGAGGGTGCAAGCGTTAATCGG
AATTACTGGGCGTAAAGCGCACGCAGGCGGTTTGTTAAGTCAGATGTGAAATCCCCGCG
CTTAACGTGGGAACTGCATTTGAAACTGGCAAGCTAGAGTCTTGTAGAGGGGGGTAGA
ATTCCAGGTGTAGCGGTGAAATGCGTAGAGATCTGGAGGAATACCGGTGGCGAAGGCG
GCCCCCTGGACAAAGACTGACGCTCAGGTGCGAAAGCGTGGGGAGCAAACAGGATTA
GATACCCTGGTAGTCCACGCTGTAAACGATGTCGACTTGGAGGTTGTGCCCTTGAGGCG
TGGCTTCCGGAGCTAACGCGTTAAGTCGACCGCCTGGGGAGTACGGCCGCAAGGTTAA
AACTCAAATGAATTGACGGGGGCCCGCACAAGCGGTGGAGCATGTGGTTTAATTCGATG
CAACGCGAAGAACCTTACCTACTCTTGACATCCACAGAACTTAGCAGAGATGCTTCGGT
GCCTTCGGGAACTGTGAGACAGGTGCTGCATGGCTGTCGTCAGCTCGTGTTGTGAAATG
TTGGGTTAAGTCCCGCAACGAGCGCAACCCTTATCCTTTGTTGCCAGCGAGTAATGTCG
GGAACTCAAAGGAGACTGCCGGTGATAAACCGGAGGAAGGTGGGGATGACGTCAAGT
CATCATGGCCCTTACGAGTAGGGCTACACACGTGCTACAATGGCAGATACAAAGTGAAG
CGAACTCGCGAGAGCAAGCGGACCACATAAAGTCTGTCGTAGTCCGGATTGGAGTCTG
CAACTCGACTCCATGAAGTCGGAATCGCTAGTAATCGTAGATCAGAATGCTACGGTGAAT
ACGTTCCCGGGCCTTGTACACACCGCCCGTCACACCATGGGAGTGGGTTGCAAAAGAA
GTAGGTAGCTTAACCTTCGGGAGGGCGCTTACCACTTTGTGATTCATGACTGGGG
pattern 136
CGCTGGCGGCAGGCCTAACACATGCAAGTCGAGCGGCAGCGGGAAGTAGTTTACTACT
TTGCCGGCGAGCGGCGGACGGGTGAGTAATGTCTGGGAAACTGCCTGATGGAGGGGGA
TAACTACTGGAAACGGTAGCTAATACCGCATGATCTCGAAAGAGCAAAGTGGGGGACCT
TCGGGCCTCACGCCATCGGATGTGCCCAGATGGGATTAGCTAGTAGGTGGGGTAATGGC
TCACCTAGGCGACGATCCCTAGCTGGTCTGAGAGGATGACCAGCCACACTGGAACTGA
GACACGGTCCAGACTCCTACGGGAGGCAGCAGTGGGGAATATTGCACAATGGGCGCAA
GCCTGATGCAGCCATGCCGCGTGTGTGAAGAAGGCCTTTGGGTTGTAAAGCACTTTCAG
CGAGGAGGAAGGCAGTCGTGTTAATAGCACGATTGATTGACGTTACTCGCAGAAGAAG
CACCGGCTAACTCCGTGCCAGCAGCCGCGGTAATACGGAGGGTGCAAGCGTTAATCGG
AATTACTGGGCGTAAAGCGCACGCAGGCGGTTTGTTAAGTCAGATGTGAAATCCCCGCG
CTTAACGTGGGAACTGCATTTGAAACTGGCAAGCTAGAGTCTTGTAGAGGGGGGTAGA
ATTCCAGGTGTAGCGGTGAAATGCGTAGAGATCTGGAGGAATACCGGTGGCGAAGGCG
GCCCCCTGGACAAAGACTGACGCTCAGGTGCGAAAGCGTGGGGAGCAAACAGGATTA
GATACCCTGGTAGTCCACGCTGTAAACGATGTCGACTTGGAGGTTGTGCCCTTGAGGCG
TGGCTTCCGGAGCTAACGCGTTAAGTCGACCGCCTGGGGAGTACGGCCGCAAGGTTAA
AACTCAAATGAATTGACGGGGGCCCGCACAAGCGGTGGAGCATGTGGTTTAATTCGATG
CAACGCGAAGAACCTTACCTACTCTTGACATCCACAGAACTTAGCAGAGATGCTTCGGT
GCCTTCGGGAACTGTGAGACAGGTGCTGCATGGCTGTCGTCAGCTCGTGTTGTGAAATG
TTGGGTTAAGTCCCGCAACGAGCGCAACCCTTATCCTTTGTTGCCAGCGAGTAATGTCG
GGAACTCAAAGGAGACTGCCGGTGATAAACCGGAGGAAGGTGGGGATGACGTCAAGT
CATCATGGCCCTTACGAGTAGGGCTACACACGTGCTACAATGGCAGATACAAAGTGAAG
CGAACTCGCGAGAGCAAGCGGACCACATAAAGTCTGTCGTAGTCCGGATTGGAGTCTG
CAACTCGACTCCATGAAGTCGGAATCGCTAGTAATCGTAGATCAGAATGCTACGGTGAAT
ACGTTCCCGGGCCTTGTACACACCGCCCGTCACACCATGGGAGTGGGTTGCAAAAGAA
GTAGGTAGCTTAACCTTCGGGAGGGCGCTTACCACTTTGTGATTCATGACTGGGG
pattern 137

CGCTGGCGGCAGGCCTAACACATGCAAGTCGAGCGGCAGCGGGAAGTAGTTTACTACT
TTGCCGGCGAGCGGCGGACGGGTGAGTAATGTCTGGGAAACTGCCTGATGGAGGGGGA
TAACTACTGGAAACGGTAGCTAATACCGCATGACCTCGCAAGAGCAAAGTGGGGGACC
TTCGGGCCTCACGCCATCGGATGTGCCCAGATGGGATTAGCTAGTAGGTGGGGTAATGG
CTCACCTAGGCGACGATCCCTAGCTGGTCTGAGAGGATGACCAGCCACACTGGAACTG
AGACACGGTCCAGACTCCTACGGGAGGCAGCAGTGGGGAATATTGCACAATGGGCGCA
AGCCTGATGCAGCCATGCCGCGTGTGTGAAGAAGGCCTTCGGGTTGTAAAGCACTTTCA
GCGAGGAGGAAGGGTTCAGTGTTAATAGCACTGTGCATTGACGTTACTCGCAGAAGAA
GCACCGGCTAACTCCGTGCCAGCAGCCGCGGTAATACGGAGGGTGCAAGCGTTAATCG
GAATTACTGGGCGTAAAGCGCACGCAGGCGGTTTGTTAAGTCAGATGTGAAATCCCCGC
GCTTAACGTGGGAACTGCATTTGAAACTGGCAAGCTAGAGTCTTGTAGAGGGGGGTAG
AATTCCAGGTGTAGCGGTGAAATGCGTAGAGATCTGGAGGAATACCGGTGGCGAAGGC
GGCCCCCTGGACAAAGACTGACGCTCAGGTGCGAAAGCGTGGGGAGCAAACAGGATT
AGATACCCTGGTAGTCCACGCTGTAAACGATGTCGACTTGGAGGTTGTGCCCTTGAGGC
GTGGCTTCCGGAGCTAACGCGTTAAGTCGACCGCCTGGGGAGTACGGCCGCAAGGTTA
AAACTCAAATGAATTGACGGGGGCCCGCACAAGCGGTGGAGCATGTGGTTTAATTCGAT
GCAACGCGAAGAACCTTACCTACTCTTGACATCCACAGAACTTAGCAGAGATGCTTCGG
TGCCTTCGGGAACTGTGAGACAGGTGCTGCATGGCTGTCGTCAGCTCGTGTTGTGAAAT
GTTGGGTTAAGTCCCGCAACGAGCGCAACCCTTATCCTTTGTTGCCAGCACGTAATGGT
GGGAACTCAAGGGAGACTGCCGGTGACAAACCGGAGGAAGGTGGGGATGACGTCAAG
TCATCATGGCCCTTACGAGTAGGGCTACACACGTGCTACAATGGCAGATACAAAGTGAA
GCGAACTCGCGAGAGCAAGCGGACCACATAAAGTCTGTCGTAGTCCGGATTGGAGTCT
GCAACTCGACTCCATGAAGTCGGAATCGCTAGTAATCGTAGATCAGAATGCTACGGTGA
ATACGTTCCCGGGCCTTGTACACACCGCCCGTCACACCATGGGAGTGGGTTGCAAAAG
AAGTAGGTAGCTTAACCTTCGGGAGGGCGCTTACCACTTTGTGATTCATGACTGGGG
pattern 138
CGCTGGCGGCAGGCCTAACACATGCAAGTCGAGCGGCAGCGGGAAGTAGTTTACTACT
TTGCCGGCGAGCGGCGGACGGGTGAGTAATGTCTGGGAAACTGCCTGATGGAGGGGGA
TAACTACTGGAAACGGTAGCTAATACCGCATGACCTCGCAAGAGCAAAGTGGGGGACC
TTCGGGCCTCACGCCATCGGATGTGCCCAGATGGGATTAGCTAGTAGGTGGGGTAATGG
CTCACCTAGGCGACGATCCCTAGCTGGTCTGAGAGGATGACCAGCCACACTGGAACTG
AGACACGGTCCAGACTCCTACGGGAGGCAGCAGTGGGGAATATTGCACAATGGGCGCA
AGCCTGATGCAGCCATGCCGCGTGTGTGAAGAAGGCCTTCGGGTTGTAAAGCACTTTCA
GCGAGGAGGAAGGGTTCAGTGTTAATAGCACTGTTCATTGACGTTACTCGCAGAAGAA
GCACCGGCTAACTCCGTGCCAGCAGCCGCGGTAATACGGAGGGTGCAAGCGTTAATCG
GAATTACTGGGCGTAAAGCGCACGCAGGCGGTTTGTTAAGTCAGATGTGAAATCCCCGC
GCTTAACGTGGGAACTGCATTTGAAACTGGCAAGCTAGAGTCTTGTAGAGGGGGGTAG
AATTCCAGGTGTAGCGGTGAAATGCGTAGAGATCTGGAGGAATACCGGTGGCGAAGGC
GGCCCCCTGGACAAAGACTGACGCTCAGGTGCGAAAGCGTGGGGAGCAAACAGGATT
AGATACCCTGGTAGTCCACGCTGTAAACGATGTCGACTTGGAGGTTGTGCCCTTGAGGC
GTGGCTTCCGGAGCTAACGCGTTAAGTCGACCGCCTGGGGAGTACGGCCGCAAGGTTA
AAACTCAAATGAATTGACGGGGGCCCGCACAAGCGGTGGAGCATGTGGTTTAATTCGAT
GCAACGCGAAGAACCTTACCTACTCTTGACATCCACAGAACTTAGCAGAGATGCTTCGG
TGCCTTCGGGAACTGTGAGACAGGTGCTGCATGGCTGTCGTCAGCTCGTGTTGTGAAAT

GTTGGGTTAAGTCCCGCAACGAGCGCAACCCTTATCCTTTGTTGCCAGCACGTAATGGT
GGGAACTCAAGGGAGACTGCCGGTGACAAACCGGAGGAAGGTGGGGATGACGTCAAG
TCATCATGGCCCTTACGAGTAGGGCTACACACGTGCTACAATGGCAGATACAAAGTGAA
GCGAACTCGCGAGAGCAAGCGGACCACATAAAGTCTGTCGTAGTCCGGATTGGAGTCT
GCAACTCGACTCCATGAAGTCGGAATCGCTAGTAATCGTAGATCAGAATGCTACGGTGA
ATACGTTCCCGGGCCTTGTACACACCGCCCGTCACACCATGGGAGTGGGTTGCAAAAG
AAGTAGGTAGCTTAACCTTCGGGAGGGCGCTTACCACTTTGTGATTCATGACTGGGG

pattern 139

CGCTGGCGGCAGGCCTAACACATGCAAGTCGAGCGGCAGCGGGAAGTAGTTTACTACT
TTGTCGGCGAGCGGCGGACGGGTGAGTAATGTCTGGGAAACTGCCTGATGGAGGGGGA
TAACTACTGGAAACGGTAGCTAATACCGCATGACCTCGCAAGAGCAAAGTGGGGGACC
TTCGGGCCTCACGCCATCGGATGTGCCCAGATGGGATTAGCTAGTAGGTGGGGTAATGG
CTCACCTAGGCGACGATCCCTAGCTGGTCTGAGAGGATGACCAGCCACACTGGAACTG
AGACACGGTCCAGACTCCTACGGGAGGCAGCAGTGGGGAATATTGCACAATGGGCGCA
AGCCTGATGCAGCCATGCCGCGTGTGTGAAGAAGGCCTTCGGGTTGTAAAGCACTTTCA
GCGAGGAGGAAGGGTTCAGTGTTAATAGCACTGTTCATTGACGTTACTCGCAGAAGAA
GCACCGGCTAACTCCGTGCCAGCAGCCGCGGTAATACGGAGGGTGCAAGCGTTAATCG
GAATTACTGGGCGTAAAGCGCACGCAGGCGGTTTGTTAAGTCAGATGTGAAATCCCCGC
GCTTAACGTGGGAACTGCATTTGAAACTGGCAAGCTAGAGTCTTGTAGAGGGGGGTAG
AATTCCAGGTGTAGCGGTGAAATGCGTAGAGATCTGGAGGAATACCGGTGGCGAAGGC
GGCCCCCTGGACAAAGACTGACGCTCAGGTGCGAAAGCGTGGGGAGCAAACAGGATT
AGATACCCTGGTAGTCCACGCTGTAAACGATGTCGACTTGGAGGTTGTGCCCTTGAGGC
GTGGCTTCCGGAGCTAACGCGTTAAGTCGACCGCCTGGGGAGTACGGCCGCAAGGTTA
AAACTCAAATGAATTGACGGGGGCCCGCACAAGCGGTGGAGCATGTGGTTTAATTCGAT
GCAACGCGAAGAACCTTACCTACTCTTGACATCCACAGAACTTAGCAGAGATGCTTCGG
TGCCTTCGGGAACTGTGAGACAGGTGCTGCATGGCTGTCGTCAGCTCGTGTTGTGAAAT
GTTGGGTTAAGTCCCGCAACGAGCGCAACCCTTATCCTTTGTTGCCAGCACGTAATGGT
GGGAACTCAAGGGAGACTGCCGGTGACAAACCGGAGGAAGGTGGGGATGACGTCAAG
TCATCATGGCCCTTACGAGTAGGGCTACACACGTGCTACAATGGCAGATACAAAGTGAA
GCGAACTCGCGAGAGCAAGCGGACCACATAAAGTCTGTCGTAGTCCGGATTGGAGTCT
GCAACTCGACTCCATGAAGTCGGAATCGCTAGTAATCGTAGATCAGAATGCTACGGTGA
ATACGTTCCCGGGCCTTGTACACACCGCCCGTCACACCATGGGAGTGGGTTGCAAAAG
AAGTAGGTAGCTTAACCTTCGGGAGGGCGCTTACCACTTTGTGATTCATGACTGGGG

pattern 140

CGCTGGCGGCAGGCCTAACACATGCAAGTCGAGCGGCAGCGGGAAGTAGTTTACTACT
TTGCCGGCGAGCGGCGGACGGGTGAGTAATGTCTGGGAAACTGCCTGATGGAGGGGGA
TAACTACTGGAAACGGTAGCTAATACCGCATGATCTCGAAAGAGCAAAGTGGGGGACTT
TCGGGCCTCACGCCATCGGATGTGCCCAGATGGGATTAGCTAGTAGGTGGGGTAATGGC
TCACCTAGGCGACGATCCCTAGCTGGTCTGAGAGGATGACCAGCCACACTGGAACTGA
GACACGGTCCAGACTCCTACGGGAGGCAGCAGTGGGGAATATTGCACAATGGGCGCAA
GCCTGATGCAGCCATGCCGCGTGTGTGAAGAAGGCCTTCGGGTTGTAAAGCACTTTCAG
CGAGGAGGAAGGCAGTCGTGTTAATAGCACGATTGATTGACGTTACTCGCAGAAGAAG
CACCGGCTAACTCCGTGCCAGCAGCCGCGGTAATACGGAGGGTGCAAGCGTTAATCGG
AATTACTGGGCGTAAAGCGCACGCAGGCGGTTTGTTAAGTCAGATGTGAAATCCCCGCG

CTTAACGTGGGAACTGCATTTGAAACTGGCAAGCTAGAGTCTTGTAGAGGGGGGTAGA
ATTCCAGGTGTAGCGGTGAAATGCGTAGAGATCTGGAGGAATACCGGTGGCGAAGGCG
GCCCCCTGGACAAAGACTGACGCTCAGGTGCGAAAGCGTGGGGAGCAAACAGGATTA
GATACCCTGGTAGTCCACGCTGTAAACGATGTCGACTTGGAGGTTGTGCCCTTGAGGCG
TGGCTTCCGGAGCTAACGCGTTAAGTCGACCGCCTGGGGAGTACGGCCGCAAGGTTAA
AACTCAAATGAATTGACGGGGGCCCGCACAAGCGGTGGAGCATGTGGTTTAATTCGATG
CAACGCGAAGAACCTTACCTACTCTTGACATCCACAGAACTTAGCAGAGATGCTTCGGT
GCCTTCGGGAACTGTGAGACAGGTGCTGCATGGCTGTCGTCAGCTCGTGTTGTGAAATG
TTGGGTTAAGTCCCGCAACGAGCGCAACCCTTATCCTTTGTTGCCAGCGAGTAATGTCG
GGAACTCAAAGGAGACTGCCGGTGATAAACCGGAGGAAGGTGGGGATGACGTCAAGT
CATCATGGCCCTTACGAGTAGGGCTACACACGTGCTACAATGGCAGATACAAAGTGAAG
CGAACTCGCGAGAGCAAGCGGACCACATAAAGTCTGTCGTAGTCCGGATTGGAGTCTG
CAACTCGACTCCATGAAGTCGGAATCGCTAGTAATCGTAGATCAGAATGCTACGGTGAAT
ACGTTCCCGGGCCTTGTACACACCGCCCGTCACACCATGGGAGTGGGTTGCAAAAGAA
GTAGGTAGCTTAACCTTCGGGAGGGCGCTTACCACTTTGTGATTCATGACTGGGG
pattern 141
CGCTGGCGGCAGGCCTAACACATGCAAGTCGAGCGGCAGCGGGAAGTAGTTTACTACT
TTGCCGGCGAGCGGCGGACGGGTGAGTAATGTCTGGGGATCTGCCTGATGGAGGGGGA
TAACTACTGGAAACGGTAGCTAATACCGCATGACCTCGCAAGAGCAAAGTGGGGGACC
TTAGGGCCTCACGCCATCGGATGAACCCAGATGGGATTAGCTAGTAGGTGGGGTAATGG
CTCACCTAGGCGACGATCCCTAGCTGGTCTGAGAGGATGACCAGCCACACTGGAACTG
AGACACGGTCCAGACTCCTACGGGAGGCAGCAGTGGGGAATATTGCACAATGGGCGCA
AGCCTGATGCAGCCATGCCGCGTGTGTGAAGAAGGCCTTCGGGTTGTAAAGCACTTTCA
GCGAGGAGGAAGGGGTTGAGTTTAATACGCTCAATCATTGACGTTACTCGCAGAAGAA
GCACCGGCTAACTCCGTGCCAGCAGCCGCGGTAATACGGAGGGTGCGAGCGTTAATCG
GAATTACTGGGCGTAAAGCGCACGCAGGCGGTTTGTTAAGTCAGATGTGAAATCCCCGC
GCTTAACGTGGGAACTGCATTTGAAACTGGCAAGCTAGAGTCTTGTAGAGGGGGGTAG
AATTCCAGGTGTAGCGGTGAAATGCGTAGAGATCTGGAGGAATACCGGTGGCGAAGGC
GGCCCCCTGGACAAAGACTGACGCTCAGGTGCGAAAGCGTGGGGAGCAAACAGGATT
AGATACCCTGGTAGTCCACGCTGTAAACGATGTCGACTTGGAGGTTGTGCCCTTGAGGC
GTGGCTTCCGGAGCTAACGCGTTAAGTCGACCGCCTGGGGAGTACGGCCGCAAGGTTA
AAACTCAAATGAATTGACGGGGGCCCGCACAAGCGGTGGAGCATGTGGTTTAATTCGAT
GCAACGCGAAGAACCTTACCTACTCTTGACATCCACAGAATTTGGCAGAGATGCTAAAG
TGCCTTCGGGAACTGTGAGACAGGTGCTGCATGGCTGTCGTCAGCTCGTGTTGTGAAAT
GTTGGGTTAAGTCCCGCAACGAGCGCAACCCTTATCCTTTGTTGCCAGCACGTAATGGT
GGGAACTCAAGGGAGACTGCCGGTGACAAACCGGAGGAAGGTGGGGATGACGTCAAG
TCATCATGGCCCTTACGAGTAGGGCTACACACGTGCTACAATGGCAGATACAAAGTGAA
GCGAACTCGCGAGAGCCAGCGGACCACATAAAGTCTGTCGTAGTCCGGATTGGAGTCT
GCAACTCGACTCCATGAAGTCGGAATCGCTAGTAATCGTAGATCAGAATGCTACGGTGA
ATACGTTCCCGGGCCTTGTACACACCGCCCGTCACACCATGGGAGTGGGTTGCAAAAG
AAGTAGGTAGCTTAACCTTCGGGAGGGCGCTTACCACTTTGTGATTCATGACTGGGG
pattern 142
CGCTGGCGGCAGGCCTAACACATGCAAGTCGAGCGGCAGCGGGAAGTAGTTTACTACT
TTGCCGGCGAGCGGCGGACGGGTGAGTAATGTCTGGGAAACTGCCTGATGGAGGGGGA

TAACTACTGGAAACGGTAGCTAATACCGCATGACCTCGCAAGAGCAAAGTGGGGGACC
TTCGGGCCTCACGCCATCGGATGTGCCCAGATGGGATTAGCTAGTAGGTGGGGTAATGG
CTCACCTAGGCGACGATCCCTAGCTGGTCTGAGAGGATGACCAGCCACACTGGAACTG
AGACACGGTCCAGACTCCTACGGGAGGCAGCAGTGGGGAATATTGCACAATGGGCGCA
AGCCTGATGCAGCCATGCCGCGTGTGTGAAGAAGGCCTTCGGGTTGTAAAGCACTTTCA
GCGAGGAGGAAGGCAGTCGTGTTAATAGCACGATTGATTGACGTTACTCGCAGAAGAA
GCACCGGCTAACTCCGTGCCAGCAGCCGCGGTAATACGGAGGGTGCAAGCGTTAATCG
GAATTACTGGGCGTAAAGCGCACGCAGGCGGTTTGTTAAGTCAGATGTGAAATCCCCGC
GCTTAACGTGGGAACTGCATTTGAAACTGGCAAGCTAGAGTCTTGTAGAGGGGGGTAG
AATTCCAGGTGTAGCGGTGAAATGCGTAGAGATCTGGAGGAATACCGGTGGCGAAGGC
GGCCCCCTGGACAAAGACTGACGCTCAGGTGCGAAAGCGTGGGGAGCAAACAGGATT
AGATACCCTGGTAGTCCACGCTGTAAACGATGTCGACTTGGAGGTTGTGCCCTTGAGGC
GTGGCTTCCGGAGCTAACGCGTTAAGTCGACCGCCTGGGGAGTACGGCCGCAAGGTTA
AAACTCAAAATGAATTGACGGGGGCCCGCACAAGCGGTGGAGCATGTGGCTTAATTCG
ATGCAACGCGAAGAACCTTACCTACTCTTGACATCCACAGAACTTAGCAGAGATGCTTC
GGTGCCTTCGGGAACTGTGAGACAGGTGCTGCATGGCTGTCGTCAGCTCGTGTTGTGA
AATGTTGGGTTAAGTCCCGCAACGAGCGCAACCCTTATCCTTTGTTGCCAGCACGTAAT
GGTGGGAACTCAAGGGAGACTGCCGGTGACAAACCGGAGGAAGGTGGGGATGACGTC
AAGTCATCATGGCCCTTACGAGTAGGGCTACACACGTGCTACAATGGCAGATACAAAGT
GAAGCGAACTCGCGAGAGCAAGCGGACCACATAAAGTCTGTCGTAGTCCGGATTGGAG
TCTGCAACTCGACTCCATGAAGTCGGAATCGCTAGTAATCGTAGATCAGAATGCTACGG
TGAATACGTTCCCGGGCCTTGTACACACCGCCCGTCACACCATGGGAGTGGGTTGCAAA
AGAAGTAGGTAGCTTAACCTTCGGGAGGGCGCTTACCACTTTGTGATTCATGACTGGGG
pattern 143
CGCTGGCGGCAGGCCTAACACATGCAAGTCGAGCGGCAGCGGGAAGTAGTTTACTACT
TTGCCGGCGAGCGGCGGACGGGTGAGTAATGTCTGGGAAACTGCCTGATGGAGGGGGA
TAACTACTGGAAACGGTAGCTAATACCGCATGACCTCGCAAGAGCAAAGTGGGGGACC
TTCGGGCCTCACGCCATCGGATGTGCCCAGATGGGATTAGCTAGTAGGTGGGGTAATGG
CTCACCTAGGCGACGATCCCTAGCTGGTCTGAGAGGATGACCAGCCACACTGGAACTG
AGACACGGTCCAGACTCCTACGGGAGGCAGCAGTGGGGAATATTGCACAATGGGCGCA
AGCCTGATGCAGCCATGCCGCGTGTGTGAAGAAGGCCTTCGGGTTGTAAAGCACTTTCA
GCGAGGAGGAAAGCAGTCGTGTTAATAGCACGATTGATTGACGTTACTCGCAGAAGAA
GCACCGGCTAACTCCGTGCCAGCAGCCGCGGTAATACGGAGGGTGCAAGCGTTAATCG
GAATTACTGGGCGTAAAGCGCACGCAGGCGGTTTGTTAAGTCAGATGTGAAATCCCCGC
GCTTAACGTGGGAACTGCATTTGAAACTGGCAAGCTAGAGTCTTGTAGAGGGGGGTAG
AATTCCAGGTGTAGCGGTGAAATGCGTAGAGATCTGGAGGAATACCGGTGGCGAAGGC
GGCCCCCTGGACAAAGACTGACGCTCAGGTGCGAAAGCGTGGGGAGCAAACAGGATT
AGATACCCTGGTAGTCCACGCTGTAAACGATGTCGACTTGGAGGTTGTGCCCTTGAGGC
GTGGCTTCCGGAGCTAACGCGTTAAGTCGACCGCCTGGGGAGTACGGCCGCAAGGTTA
AAACTCAAATGAATTGACGGGGGCCCGCACAAGCGGTGGAGCATGTGGTTTAATTCGAT
GCAACGCGAAGAACCTTACCTACTCTTGACATCCACAGAACTTAGCAGAGATGCTTCGG
TGCCTTCGGGAACTGTGAGACAGGTGCTGCATGGCTGTCGTCAGCTCGTGTTGTGAAAT
GTTGGGTTAAGTCCCGCAACGAGCGCAACCCTTATCCTTTGTTGCCAGCACGTAATGGT
GGGAACTCAAGGGAGACTGCCGGTGACAAACCGGAGGAAGGTGGGGATGACGTCAAG

TCATCATGGCCCTTACGAGTAGGGCTACACACGTGCTACAATGGCAGATACAAAGTGAA
GCGAACTCGCGAGAGCAAGCGGACCACATAAAGTCTGTCGTAGTCCGGATTGGAGTCT
GCAACTCGACTCCATGAAGTCGGAATCGCTAGTAATCGTAGATCAGAATGCTACGGTGA
ATACGTTCCCGGGCCTTGTACACACCGCCCGTCACACCATGGGAGTGGGTTGCAAAAG
AAGTAGGTAGCTTAACCTTCGGGAGGGCGCTTACCACTTTGTGATTCATGACTGGGG

pattern 144

CGCTGGCGGCAGGCCTAACACATGCAAGTCGAGCGGCAGCGGGAAGTAGTTTACTACT
TTGCCGGCGAGCGGCGGACGGGTGAGTAATGTCTGGGAAACTGCCTGATGGAGGGGGA
TAACTACTGGAAACGGTAGCTAATACCGCATGACCTCGCAAGAGCAAAGTGGGGGACC
TTAGGGCCTCACGCCATCGGATGTGCCCAGATGGGATTAGCTAGTAGGTGGGGTAATGG
CTCACCTAGGCGACGATCCCTAGCTGGTCTGAGAGGATGACCAGCCACACTGGAACTG
AGACACGGTCCAGACTCCTACGGGAGGCAGCAGTGGGGAATATTGCACAATGGGCGCA
AGCCTGATGCAGCCATGCCGCGTGTGTGAAGAAGGCCTTCGGGTTGTAAAGCACTTTCA
GCGAGGAGGAAGGGTTCAGTGTTAATAGCACTGTTCATTGACGTTACTCGCAGAAGAA
GCACCGGCTAACTCCGTGCCAGCAGCCGCGGTAATACGGAGGGTGCAAGCGTTAATCG
GAATTACTGGGCGTAAAGCGCACGCAGGCGGTTTGTTAAGTCAGATGTGAAATCCCCGC
GCTTAACGTGGGAACTGCATTTGAAACTGGCAAGCTAGAGTCTTGTAGAGGGGGGTAG
AATTCCAGGTGTAGCGGTGAAATGCGTAGAGATCTGGAGGAATACCGGTGGCGAAGGC
GGCCCCCTGGACAAAGACTGACGCTCAGGTGCGAAAGCGTGGGGAGCAAACAGGATT
AGATACCCTGGTAGTCCACGCTGTAAACGATGTCGACTTGGAGGTTGTGCCCTTGAGGC
GTGGCTTCCGGAGCTAACGCGTTAAGTCGACCGCCTGGGGAGTACGGCCGCAAGGTTA
AAACTCAAATGAATTGACGGGGGCCCGCACAAGCGGTGGAGCATGTGGTTTAATTCGAT
GCAACGCGAAGAACCTTACCTACTCTTGACATCCACAGAACTTAGCAGAGATGCTTCGG
TGCCTTCGGGAACTGTGAGACAGGTGCTGCATGGCTGTCGTCAGCTCGTGTTGTGAAAT
GTTGGGTTAAGTCCCGCAACGAGCGCAACCCTTATCCTTTGTTGCCAGCACGTAATGGT
GGGAACTCAAGGGAGACTGCCGGTGACAAACCGGAGGAAGGTGGGGATGACGTCAAG
TCATCATGGCCCTTACGAGTAGGGCTACACACGTGCTACAATGGCAGATACAAAGTGAA
GCGAACTCGCGAGAGCAAGCGGACCACATAAAGTCTGTCGTAGTCCGGATTGGAGTCT
GCAACTCGACTCCATGAAGTCGGAATCGCTAGTAATCGTAGATCAGAATGCTACGGTGA
ATACGTTCCCGGGCCTTGTACACACCGCCCGTCACACCATGGGAGTGGGTTGCAAAAG
AAGTAGGTAGCTTAACCTTCGGGAGGGCGCTTACCACTTTGTGATTCATGACTGGGG

pattern 145

CGCTGGCGGCAGGCCTAACACATGCAAGTCGAGCGGCAGCGGAAAGTAGCTTGCTACT
TTGCCGGCGAGCGGCGGACGGGTGAGTAATGTCTGGGAAACTGCCTGATGGAGGGGGA
TAACTACTGGAAACGGTAGCTAATACCGCATGACCTCGCAAGAGCAAAGTGGGGGACC
TTAGGGCCTCACGCCATCGGATGTGCCCAGATGGGATTAGCTAGTAGGTGGGGTAATGG
CTCACCTAGGCGACGATCCCTAGCTGGTCTGAGAGGATGACCAGCCACACTGGAACTG
AGACACGGTCCAGACTCCTACGGGAGGCAGCAGTGGGGAATATTGCACAATGGGCGCA
AGCCTGATGCAGCCATGCCGCGTGTGTGAAGAAGGCCTTCGGGTTGTAAAGCACTTTCA
GCGAGGAGGAAGGCAATCGTGTTAATAGCACGGTTGATTGACGTTACTCGCAGAAGAA
GCACCGGCTAACTCCGTGCCAGCAGCCGCGGTAATACGGAGGGTGCAAGCGTTAATCG
GAATTACTGGGCGTAAAGCGCACGCAGGCGGTTTGTTAAGTCAGATGTGAAATCCCCGC
GCTTAACGTGGGAACTGCATTTGAAACTGGCAAGCTAGAGTCTTGTAGAGGGGGGTAG
AATTCCAGGTGTAGCGGTGAAATGCGTAGAGATCTGGAGGAATACCGGTGGCGAAGGC

GGCCCCCTGGACAAAGACTGACGCTCAGGTGCGAAAGCGTGGGGAGCAAACAGGATT
AGATACCCTGGTAGTCCACGCTGTAAACGATGTCGACTTGGAGGTTGTGCCCTTGAGGC
GTGGCTTCCGGAGCTAACGCGTTAAGTCGACCGCCTGGGGAGTACGGCCGCAAGGTTA
AAACTCAAATGAATTGACGGGGGCCCGCACAAGCGGTGGAGCATGTGGTTTAATTCGAT
GCAACGCGAAGAACCTTACCTACTCTTGACATCCACAGAACTTAGCAGAGATGCTTCGG
TGCCTTCGGGAACTGTGAGACAGGTGCTGCATGGCTGTCGTCAGCTCGTGTTGTGAAAT
GTTGGGTTAAGTCCCGCAACGAGCGCAACCCTTATCCTTTGTTGCCAGCACGTAATGGT
GGGAACTCAAGGGAGACTGCCGGTGACAAACCGGAGGAAGGTGGGGATGACGTCAAG
TCATCATGGCCCTTACGAGTAGGGCTACACACGTGCTACAATGGCAGATACAAAGTGAA
GCGAACTCGCGAGAGCAAGCGGACCACATAAAGTCTGTCGTAGTCCGGATTGGAGTCT
GCAACTCGACTCCATGAAGTCGGAATCGCTAGTAATCGTAGATCAGAATGCTACGGTGA
ATACGTTCCCGGGCCTTGTACACACCGCCCGTCACACCATGGGAGTGGGTTGCAAAAG
AAGTAGGTAGCTTAACCTTCGGGAGGGCGCTTACCACTTTGTGATTCATGACTGGGG
pattern 146
CGCTGGCGGCAGGCCTAACACATGCAAGTCGAGCGGCAGCGGGAAGTAGTTTACTACT
TTGCCGGCGAGCGGCGGACGGGTGAGTAATGTCTGGGAAACTGCCTGATGGAGGGGGA
TAACTACTGGAAACGGTAGCTAATACCGCATGACCTCGCAAGAGCAAAGTGGGGGACC
TTCGGGCCTCACGCCATCGGATGTGCCCAGATGGGATTAGCTAGTAGGTGGGGTAATGG
CTCACCTAGGCGACGATCCCTAGCTGGTCTGAGAGGATGACCAGCCACACTGGAACTG
AGACACGGTCCAGACTCCTACGGGAGGCAGCAGTGGGGAATATTGCACAATGGGCGCA
AGCCTGATGCAGCCATGCCGCGTGTGTGAAGAAGGCCTTCGGGTTGTAAAGCACTTTCA
GCGAGGAGGAAGGCAGTCGTGTTAATAGCACGATTGATTGACGTTACTCGCAGAAGAA
GCACCGGCTAACTCCGTGCCAGCAGCCGCGGTAATACGGAGGGTGCAAGCGTTAATCG
GAATTACTGGGCGTAAAGCGCACGCAGGCGGTTTGTTAAGTCAGATGTGAAATCCCCGC
GCTTAACGTGGGAACTGCATTTGAAACTGGCAAGCTAGAGTCTTGTAGAGGGGGGTAG
AATTCCAGGTGTAGCGGTGAAATGCGTAGAGATCTGGAGGAATACCGGTGGCGAAGGC
GGCCCCCTGGACAAAGACTGACGCTCAGGTGCGAAAGCGTGGGGAGCAAACAGGATT
AGATACCCTGGTAGTCCACGCTGTAAACGATGTCGACTTGGAGGTTGTGCCCTTGAGGT
GTGGCTTCCGGAGCTAACGCGTTAAGTCGACCGCCTGGGGAGTACGGCCGCAAGGTTA
AAACTCAAATGAATTGACGGGGGCCCGCACAAGCGGTGGAGCATGTGGTTTAATTCGAT
GCAACGCGAAGAACCTTACCTACTCTTGACATCCACAGAACTTAGCAGAGATGCTTCGG
TGCCTTCGGGAACTGTGAGACAGGTGCTGCATGGCTGTCGTCAGCTCGTGTTGTGAAAT
GTTGGGTTAAGTCCCGCAACGAGCGCAACCCTTATCCTTTGTTGCCAGCACGTAATGGT
GGGAACTCAAGGGAGACTGCCGGTGACAAACCGGAGGAAGGTGGGGATGACGTCAAG
TCATCATGGCCCTTACGAGTAGGGCTACACACGTGCTACAATGGCAGATACAAAGTGAA
GCGAACTCGCGAGAGCAAGCGGACCACATAAAGTCTGTCGTAGTCCGGATTGGAGTCT
GCAACTCGACTCCATGAAGTCGGAATCGCTAGTAATCGTAGATCAGAATGCTACGGTGA
ATACGTTCCCGGGCCTTGTACACACCGCCCGTCACACCATGGGAGTGGGTTGCAAAAG
AAGTAGGTAGCTTAACCTTCGGGAGGGCGCTTACCACTTTGTGATTCATGACTGGGG
pattern 147
CGCTGGCGGCAGGCCTAACACATGCAAGTCGAGCGGCAGCGGGAAGTAGTTTACTACT
TTGCCGGCGAGCGGCGGACGGGTGAGTAATGTCTGGGAAACTGCCTGATGGAGGGGGA
TAACTACTGGAAACGGTAGCTAATACCGCAGGACCTCGCAAGAGCAAAGTGGGGGACC
TTCGGGCCTCACGCCATCGGATGTGCCCAGATGGGATTAGCTAGTAGGTGGGGTAATGG

CTCACCTAGGCGACGATCCCTAGCTGGTCTGAGAGGATGACCAGCCACACTGGAACTG
AGACACGGTCCAGACTCCTACGGGAGGCAGCAGTGGGGAATATTGCACAATGGGCGCA
AGCCTGATGCAGCCATGCCGCGTGTGTGAAGAAGGCCTTCGGGTTGTAAAGCACTTTCA
GCGAGGAGGAAGGCAGTCGTGTTAATAGCACGATTGATTGACGTTACTCGCAGAAGAA
GCACCGGCTAACTCCGTGCCAGCAGCCGCGGTAATACGGAGGGTGCAAGCGTTAATCG
GAATTACTGGGCGTAAAGCGCACGCAGGCGGTTTGTTAAGTCAGATGTGAAATCCCCGC
GCTTAACGTGGGAACTGCATTTGAAACTGGCAAGCTAGAGTCTTGTAGAGGGGGGTAG
AATTCCAGGTGTAGCGGTGAAATGCGTAGAGATCTGGAGGAATACCGGTGGCGAAGGC
GGCCCCCTGGACAAAGACTGACGCTCAGGTGCGAAAGCGTGGGGAGCAAACAGGATT
AGATACCCTGGTAGTCCACGCTGTAAACGATGTCGACTTGGAGGTTGTGCCCTTGAGGC
GTGGCTTCCGGAGCTAACGCGTTAAGTCGACCGCCTGGGGAGTACGGCCGCAAGGTTA
AAACTCAAATGAATTGACGGGGGCCCGCACAAGCGGTGGAGCATGTGGTTTAATTCGAT
GCAACGCGAAGAACCTTACCTACTCTTGACATCCACAGAACTTAGCAGAGATGCTTCGG
TGCCTTCGGGAACTGTGAGACAGGTGCTGCATGGCTGTCGTCAGCTCGTGTTGTGAAAT
GTTGGGTTAAGTCCCGCAACGAGCGCAACCCTTATCCTTTGTTGCCAGCACGTAATGGT
GGGAACTCAAGGGAGACTGCCGGTGACAAACCGGAGGAAGGTGGGGATGACGTCAAG
TCATCATGGCCCTTACGAGTAGGGCTACACACGTGCTACAATGGCAGATACAAAGTGAA
GCGAACTCGCGAGAGCAAGCGGACCACATAAAGTCTGTCGTAGTCCGGATTGGAGTCT
GCAACTCGACTCCATGAAGTCGGAATCGCTAGTAATCGTAGATCAGAATGCTACGGTGA
ATACGTTCCCGGGCCTTGTACACACCGCCCGTCACACCATGGGAGTGGGTTGCAAAAG
AAGTAGGTAGCTTAACCTTCGGGAGGGCGCTTACCACTTTGTGATTCATGACTGGGG
pattern 148
CGCTGGCGGCAGGCCTAACACATGCAAGTCGAGCGGCAGCGGAAAGTAGCTTGCTACT
TTGCCGGCGAGCGGCGGACGGGTGAGTAATGTCTGGGAAACTGCCTGATGGAGGGGGA
TAACTACTGGAAACGGTAGCTAATACCGCATAACCTCGCAAGAGCAAAGTGGGGGACC
TTCGGGCCTCACGCCATCGGATGTGCCCAGATGGGATTAGCTAGTAGGTGGGGTAATGG
CTCACCTAGGCGACGATCCCTAGCTGGTCTGAGAGGATGACCAGCCACACTGGAACTG
AGACACGGTCCAGACTCCTACGGGAGGCAGCAGTGGGGAATATTGCACAATGGGCGCA
AGCCTGATGCAGCCATGCCGCGTGTGTGAAGAAGGCCTTCGGGTTGTAAAGCACTTTCA
GCGAGGAGGAAGGGTTGAGTGTTAATAGCACTGAACATTGACGTTACTCGCAGAAGAA
GCACCGGCTAACTCCGTGCCAGCAGCCGCGGTAATACGGAGGGTGCAAGCGTTAATCG
GAATTACTGGGCGTAAAGCGCACGCAGGCGGTTTGTTAAGTCAGATGTGAAATCCCCGA
GCTTAACTTGGGAACTGCATTTGAAACTGGCAAGCTAGAGTCTTGTAGAGGGGGGTAG
AATTCCAGGTGTAGCGGTGAAATGCGTAGAGATCTGGAGGAATACCGGTGGCGAAGGC
GGCCCCCTGGACAAAGACTGACGCTCAGGTGCGAAAGCGTGGGGAGCAAACAGGATT
AGATACCCTGGTAGTCCACGCTGTAAACGATGTCGACTTGGAGGTTGTGCCCTTGAGGC
GTGGCTTCCGGAGCTAACGCGTTAAGTCGACCGCCTGGGGAGTACGGCCGCAAGGTTA
AAACTCAAATGAATTGACGGGGGCCCGCACAAGCGGTGGAGCATGTGGTTTAATTCGAT
GCAACGCGAAGAACCTTACCTACTCTTGACATCCACAGAACTTGGCAGAGATGCCTTGG
TGCCTTCGGGACCTGTGAGACAGGTGCTGCATGGCTGTCGTCAGCTCGTGTTGTGAAAT
GTTGGGTTAAGTCCCGCAACGAGCGCAACCCTTATCCTTTGTTGCCAGCACGTCATGGT
GGGAACTCAAGGGAGACTGCCGGTGACAAACCGGAGGAAGGTGGGGATGACGTCAAG
TCATCATGGCCCTTACGAGTAGGGCTACACACGTGCTACAATGGCAGATACAAAGTGAA
GCGAACTCGCGAGAGCAAGCGGACCACATAAAGTCTGTCGTAGTCCGGATTGGAGTCT

GCAACTCGACTCCATGAAGTCGGAATCGCTAGTAATCGTAGATCAGAATGCTACGGTGA
ATACGTTCCCGGGCCTTGTACACACCGCCCGTCACACCATGGGAGTGGGTTGCAAAAG
AAGTAGGTAGCTTAACCTTCGGGAGGGCGCTTACCACTTTGTGATTCATGACTGGGG
pattern 149
CGCTGGCGGCAGGCCTAACACATGCAAGTCGAGCGGCAGCGGAAAGTAGCTTGCTACT
TTGCCGGCGAGCGGCGGACGGGTGAGTAATGTCTGGGAAACTGCCTGATGGAGGGGGA
TAACTACTGGAAACGGTAGCTAATACCGCATAACCTCGCAAGAGCAAAGTGGGGGACC
TTCGGGCCTCACGCCATCGGATGTGCCCAGATGGGATTAGCTAGTAGGTGGGGTAATGG
CTCACCTAGGCGACGATCCCTAGCTGGTCTGAGAGGATGACCAGCCACACTGGAACTG
AGACACGGTCCAGACTCCTACGGGAGGCAGCAGTGGGGAATATTGCACAATGGGCGCA
AGCCTGATGCAGCCATGCCGCGTGTGTGAAGAAGGCCTTCGGGTTGTAAAGCACTTTCA
GCGAGGAGGAAGGGTTGAGTGTTAATAGCACTGAACATTGACGTTACTCGCAGAAGAA
GCACCGGCTAACTCCGTGCCAGCAGCCGCGGTAATACGGAGGGTGCAAGCGTTAATCG
GAATTACTGGGCGTAAAGCGCACGCAGGCGGTTTGTTAAGTCAGATGTGAAATCCCCGA
GCTTAACTTGGGAACTGCATTTGAAACTGGCAAGCTAGAGTCTTGTAGAGGGGGGTAGAA
TTCCAGGTGTAGCGGTGAAATGCGTAGAGATCTGGAGGAATACCGGTGGCGAAGGCGG
CCCCCTGGACAAAGACTGACGCTCAGGTGCGAAAGCGTGGGGAGCAAACAGGATTAG
ATACCCTGGTAGTCCACGCTGTAAACGATGTCGACTTGGAGGTTGTGCCCTTGAGGCGT
GGCTTCCGGAGCTAACGCGTTAAGTCGACCGCCTGGGGAGTACGGCCGCAAGGTTAAA
ACTCAAATGAATTGACGGGGGCCCGCACAAGCGGTGGAGCATGTGGTTTAATTCGATGC
AACGCGAAGAACCTTACCTACTCTTGACATCCACAGAACTTGGCAGAGATGCCTTGGTG
CCTTCGGGACCTGTGAGACAGGTGCTGCATGGCTGTCGTCAGCTCGTGTTGTGAAATGT
TGGGTTAAGTCCCGCAACGAGCGCAACCCTTATCCTTGTTGCCAGCACGTCATGGTGGG
AACTCAAGGGAGACTGCCGGCGACAAACCGGAGGAAGGTGGGGATGACGTCAAGTCA
TCATGGCCCTTACGAGTAGGGCTACACACGTGCTACAATGGCAGATACAAAGTGAAGCG
AACTCGCGAGAGCAAGCGGACCACATAAAGTCTGTCGTAGTCCGGATTGGAGTCTGCA
ACTCGACTCCATGAAGTCGGAATCGCTAGTAATCGTAGATCAGAATGCTACGGTGAATAC
GTTCCCGGGCCTTGTACACACCGCCCGTCACACCATGGGAGTGGGTTGCAAAAGAAGT
AGGTAGCTTAACCTTCGGGAGGGCGCTTACCACTTTGTGATTCATGACTGGGG
pattern 150
CGCTGGCGGCAGGCCTAACACATGCAAGTCGAGCGGCAGCGGAAAGTAGCTTGCTACT
TTGCCGGCGAGCGGCGGACGGGTGAGTAATGTCTGGGAAACTGCCTGATGGAGGGGGA
TAACTACTGGAAACGGTAGCTAATACCGCATAACCTCGCAAGAGCAAAGTGGGGGACC
TTCGGGCCTCACGCCATCGGATGTGCCCAGATGGGATTAGCTAGTAGGTGGGGTAATGG
CTCACCTAGGCGACGATCCCTAGCTGGTCTGAGAGGATGACCAGCCACACTGGAACTG
AGACACGGTCCAGACTCCTACGGGAGGCAGCAGTGGGGAATATTGCACAATGGGCGCA
AGCCTGATGCAGCCATGCCGCGTGTGTGAAGAAGGCCTTCGGGTTGTAAAGCACTTTCA
GCGAGGAGGAAGGGTTGAGTGTTAATAGCACTGAACATTGACGTTACTCGCAGAAGAA
GCACCGGCTAACTCCGTGCCAGCAGCCGCGGTAATACGGAGGGTGCAAGCGTTAATCG
GAATTACTGGGCGTAAAGCGCACGCAGGCGGTTTGTAAGTCAGATGTGAAATCCCCGA
GCTTAACTTGGGAACTGCATTTGAAACTGGCAAGCTAGAGTCTTGTAGAGGGGGGTAG
AATTCCAGGTGTAGCGGTGAAATGCGTAGAGATCTGGAGGAATACCGGTGGCGAAGGC
GGCCCCCTGGACAAAGACTGACGCTCAGGTGCGAAAGCGTGGGGAGCAAACAGGATT
AGATACCCTGGTAGTCCACGCTGTAAACGATGTCGACTTGGAGGTTGTGCCCTTGAGGC

GTGGCTTCCGGAGCTAACGCGTTAAGTCGACCGCCTGGGGAGTACGGCCGCAAGGTTA
AAACTCAAATGAATTGACGGGGGCCCGCACAAGCGGTGGAGCATGTGGTTTAATTCGAT
GCAACGCGAAGAACCTTACCTACTCTTGACATCCACAGAACTTGGCAGAGATGCCTTGG
TGCCTTCGGGACCTGTGAGACAGGTGCTGCATGGCTGTCGTCAGCTCGTGTTGTGAAAT
GTTGGGTTAAGTCCCGCAACGAGCGCAACCCTTATCCTTTGTTGCCAGCACGTCATGGT
GGGAACTCAAGGGAGACTGCCGGTGACAAACCGGAGGAAGGTGGGATGACGTCAAGT
CATCATGGCCCTTACGAGTAGGGCTACACACGTGCTACAATGGCAGATACAAAGTGAAG
CGAACTCGCGAGAGCAAGCGGACCACATAAAGTCTGTCGTAGTCCGGATTGGAGTCTG
CAACTCGACTCCATGAAGTCGGAATCGCTAGTAATCGTAGATCAGAATGCTACGGTGAAT
ACGTTCCCGGGCCTTGTACACACCGCCCGTCACACCATGGGAGTGGGTTGCAAAAGAA
GTAGGTAGCTTAACCTTCGGGAGGGCGCTTACCACTTTGTGATTCATGACTGGGG

pattern 151

CGCTGGCGGCAGGCCTAACACATGCAAGTCGAGCGGCAGCGGAAAGTAGCTTGCTACT
TTGCCGGCGAGCGGCGGACGGGTGAGTAATGTCTGGGAAACTGCCTGATGGAGGGGGA
TAACTACTGGAAACGGTAGCTAATACCGCATAACCTCGCAAGAGCAAAGTGGGGGACC
TTCGGGCCTCACGCCATCGGATGTGCCCAGATGGGATTAGCTAGTAGGTGGGGTAATGG
CTCACCTAGGCGACGATCCCTAGCTGGTCTGAGAGGATGACCAGCCACACTGGAACTG
AGACACGGTCCAGACTCCTACGGGAGGCAGCAGTGGGGAATATTGCACAATGGGCGCA
AGCCTGATGCAGCCATGCCGCGTGTGTGAAGAAGGCCTTCGGGTTGTAAAGCACTTTCA
GCGAGGAGGAAGGGTTGAGTGTTAATAGCACTGAACATTGACGTTACTCGCAGAAGAA
GCACCGGCTAACTCCGTGCCAGCAGCCGCGGTAATACGGAGGGTGCAAGCGTTAATCG
GAATTACTGGGCGTAAAGCGCACGCAGGCGGTTTGTTAAGTCAGATGTGAAATCCCCGA
GCTTAACTTGGGAACTGCATTTGAAACTGGCAAGCTAGAGTCTTGTAGAGGGGGGTAG
AATTCCAGGTGTAGCGGTGAAATGCGTAGAGATCTGGAGGAATACCGGTGGCGAAGGC
GGCCCCCTGGACAAAGACTGACGCTCAGGTGCGAAAGCGTGGGGAGCAAACAGGATT
AGATACCCTGGTAGTCCACGCTGTAAACGATGTCGACTTGGAGGTTGTGCCCTTGAGGC
GTGGCTTCCGGAGCTAACGCGTTAAGTCGACCGCCTGGGGAGTACGGCCGCAAGGTTA
AAACTCAAATGAATTGACGGGGGCCCGCACAAGCGGTGGAGCATGTGGTTTAATTCGAT
GCAACGCGAAGAACCTTACCTACTCTTGACATCCACAGAACTTGGCAGAGATGCCTTGG
TGCCTTCGGGACCTGTGAGACAGGTGCTGCATGGCTGTCGTCAGCTCGTGTTGTGAAAT
GTTGGGTTAAGTCCCGCAACGAGCGCAACCCTTATCCTTTGTTGCCAGCACGTCATGGT
GGGAACTCAAGGGAGACTGCCGGTGACAAACCGGAGGAAGGTGGGGATGACGTCAAG
TCATCATGGCCCTTACGAGTAGGGCTACACACGTGCTACAATGGCAGATACAAAGTGAA
GCGAACTCGCGAGAGCAAGCGGACCACATAAAGTCTGTCGTAGTCCGGATTGGAGTCT
GCAACTCGACTCCATGAAGTCGGAATCGCTAGTAATCGTAGATCAGAATGCTACGGTGA
ATACGTTCCCGGGCCTTGTACACACCGCCCGTCACACCATGGGAGTGGGTTGCAAAGA
AGTAGGTAGCTTAACCTTCGGGAGGGCGCTTACCACTTTGTGATTCATGACTGGGG

pattern 152

CGCTGGCGGCAGGCCTAACACATGCAAGTCGAGCGGCAGCGGAAAGTAGCTTGCTACT
TTGCCGGCGAGCGGCGGACGGGTGAGTAATGTCTGGGGATCTGCCTGATGGAGGGGGA
TAACTACTGGAAACGGTAGCTAATACCGCATGACCTCGAAAGAGCAAAGTGGGGGACC
TTCGGGCCTCACGCCATCGGATGAACCCAGATGGGATTAGCTAGTAGGTGGGGTAATGG
CTCACCTAGGCGACGATCCCTAGCTGGTCTGAGAGGATGACCAGCCACACTGGAACTG
AGACACGGTCCAGACTCCTACGGGAGGCAGCAGTGGGGAATATTGCACAATGGGCGCA

AGCCTGATGCAGCCATGCCGCGTGTGTGAAGAAGGCCTTCGGGTTGTAAAGCACTTTCA
GCGAGGAGGAAGGCATTGTGGTTAATAACCACAGTGATTGACGTTACTCGCAGAAGAA
GCACCGGCTAACTCCGTGCCAGCAGCCGCGGTAATACGGAGGGTGCAAGCGTTAATCG
GAATTACTGGGCGTAAAGCGCACGCAGGCGGTTTGTTAAGTCAGATGTGAAATCCCCGC
GCTTAACGTGGGAACTGCATTTGAAACTGGCAAGCTAGAGTCTTGTAGAGGGGGGTAG
AATTCCAGGTGTAGCGGTGAAATGCGTAGAGATCTGGAGGAATACCGGTGGCGAAGGC
GGCCCCCTGGACAAAGACTGACGCTCAGGTGCGAAAGCGTGGGGAGCAAACAGGATT
AGATACCCTGGTAGTCCACGCTGTAAACGATGTCGACTTGGAGGTTGTGCCCTTGAGGT
GTGGCTTCCGGAGCTAACGCGTTAAGTCGACCGCCTGGGGAGTACGGCCGCAAGGTTA
AAACTCAAATGAATTGACGGGGGCCCGCACAAGCGGTGGAGCATGTGGTTTAATTCGAT
GCAACGCGAAGAACCTTACCTACTCTTGACATCCACAGAACTTAGCAGAGATGCTTCGG
TGCCTTCGGGAACTGTGAGACAGGTGCTGCATGGCTGTCGTCAGCTCGTGTTGTGAAAT
GTTGGGTTAAGTCCCGCAACGAGCGCAACCCTTATCCTTTGTTGCCAGCACGTAATGGT
GGGAACTCAAGGGAGACTGCCGGTGACAAACCGGAGGAAGGTGGGGATGACGTCAAG
TCATCATGGCCCTTACGAGTAGGGCTACACACGTGCTACAATGGCAGATACAAAGTGAA
GCGAACTCGCGAGAGCAAGCGGACCACATAAAGTCTGTCGTAGTCCGGATTGGAGTCT
GCAACTCGACTCCATGAAGTCGGAATCGCTAGTAATCGTAGATCAGAATGCTACGGTGA
ATACGTTCCCGGGCCTTGTACACACCGCCCGTCACACCATGGGAGTGGGTTGCAAAAG
AAGTAGGTAGCTTAACCTTCGGGAGGGCGCTTACCACTTTGTGATTCATGACTGGGG
pattern 153
CGCTGGCGGCAGGCCTAACACATGCAAGTCGAGCGGCAGCGGAAAGTAGCTTGCTACT
TTGCCGGCGAGCGGCGGACGGGTGAGTAATGTCTGGGGATCTGCCTGATGGAGGGGGA
TAACTACTGGAAACGGTAGCTAATACCGCATGACCTCGAAAGAGCAAAGTGGGGGACC
TTCGGGCCTCACGCCATCGGATGAACCCAGATGGGATTAGCTAGTAGGTGGGGTAATGG
CTCACCTAGGCGACGATCCCTAGCTGGTCTGAGAGGATGACCAGCCACACTGGAACTG
AGACACGGTCCAGACTCCTACGGGAGGCAGCAGTGGGGAATATTGCACAATGGGCGCA
AGCCTGATGCAGCCATGCCGCGTGTGTGAAGAAGGCCTTCGGGTTGTAAAGCACTTTCA
GCGAGGAGGAAGGCATTGTGGTTAATAACCGCAGTGATTGACGTTACTCGCAGAAGAA
GCACCGGCTAACTCCGTGCCAGCAGCCGCGGTAATACGGAGGGTGCAAGCGTTAATCG
GAATTACTGGGCGTAAAGCGCACGCAGGCGGTTTGTTAAGTCAGATGTGAAATCCCCGC
GCTTAACGTGGGAACTGCATTTGAAACTGGCAAGCTAGAGTCTTGTAGAGGGGGGTAG
AATTCCAGGTGTAGCGGTGAAATGCGTAGAGATCTGGAGGAATACCGGTGGCGAAGGC
GGCCCCCTGGACAAAGACTGACGCTCAGGTGCGAAAGCGTGGGGAGCAAACAGGATT
AGATACCCTGGTAGTCCACGCTGTAAACGATGTCGACTTGGAGGTTGTGCCCTTGAGGC
GTGGCTTCCGGAGCTAACGCGTTAAGTCGACCGCCTGGGGAGTACGGCCGCAAGGTTA
AAACTCAAATGAATTGACGGGGGCCCGCACAAGCGGTGGAGCATGTGGTTTAATTCGAT
GCAACGCGAAGAACCTTACCTACTCTTGACATCCACAGAACTTAGCAGAGATGCTTCGG
TGCCTTCGGGAACTGTGAGACAGGTGCTGCATGGCTGTCGTCAGCTCGTGTTGTGAAAT
GTTGGGTTAAGTCCCGCAACGAGCGCAACCCTTATCCTTTGTTGCCAGCACGTAATGGT
GGGAACTCAAGGGAGACTGCCGGTGACAAACCGGAGGAAGGTGGGGATGACGTCAAG
TCATCATGGCCCTTACGAGTAGGGCTACACACGTGCTACAATGGCAGATACAAAGTGAA
GCGAACTCGCGAGAGCAAGCGGACCACATAAAGTCTGTCGTAGTCCGGATTGGAGTCT
GCAACTCGACTCCATGAAGTCGGAATCGCTAGTAATCGTAGATCAGAATGCTACGGTGA
ATACGTTCCCGGGCCTTGTACACACCGCCCGTCACACCATGGGAGTGGGTTGCAAAAG

AAGTAGGTAGCTTAACCTTCGGGAGGGCGTTTACCACTTTGTGATTCATGACTGGGG
pattern 154
CGCTGGCGGCAGGCCTAACACATGCAAGTCGAGCGGCAGCGGAAAGTAGCTTGCTACT
TTGCCGGCGAGCGGCGGACGGGTGAGTAATGTCTGGGGATCTGCCTGATGGAGGGGGA
TAACTACTGGAAACGGTAGCTAATACCGCATGACCTCGAAAGAGCAAAGTGGGGGACC
TTCGGGCCTCACGCCATCGGATGAACCCAGATGGGATTAGCTAGTAGGTGGGGTAATGG
CTCACCTAGGCGACGATCCCTAGCTGGTCTGAGAGGATGACCAGCCACACTGGAACTG
AGACACGGTCCAGACTCCTACGGGAGGCAGCAGTGGGGAATATTGCACAATGGGCGCA
AGCCTGATGCAGCCATGCCGCGTGTGTGAAGAAGGCCTTCGGGTTGTAAAGCACTTTCA
GCGAGGAGGAAGGCATTGTGGTTAATAACCGCAGTGATTGACGTTACTCGCAGAAGAA
GCACCGGCTAACTCCGTGCCAGCAGCCGCGGTAATACGGAGGGTGCAAGCGTTAATCG
GAATTACTGGGCGTAAAGCGCACGCAGGCGGTTTGTTAAGTCAGATGTGAAATCCCCGC
GCTTAACGTGGGAACTGCATTTGAAACTGGCAAGCTAGAGTCTTGTAGAGGGGGGTAG
AATTCCAGGTGTAGCGGTGAAATGCGTAGAGATCTGGAGGAATACCGGTGGCGAAGGC
GGCCCCCTGGACAAAGACTGACGCTCAGGTGCGAAAGCGTGGGGAGCAAACAGGATT
AGATACCCTGGTAGTCCACGCTGTAAACGATGTCGACTTGGAGGTTGTGCCCTTGAGGC
GTGGCTTCCGGAGCTAACGCGTTAAGTCGACCGCCTGGGGAGTACGGCCGCAAGGTTA
AAACTCAAATGAATTGACGGGGGCCCGCACAAGCGGTGGAGCATGTGGTTTAATTCGAT
GCAACGCGAAGAACCTTACCTACTCTTGACATCCACAGAACTTAGCAGAGATGCTTCAG
TGCCTTCGGGAACCGTGAGACAGGTGCTGCATGGCTGTCGTCAGCTCGTGTTGTGAAAT
GTTGGGTTAAGTCCCGCAACGAGCGCAACCCTTATCCTTTGTTGCCAGCACGTAATGGT
GGGAACTCAAGGGAGACTGCCGGTGACAAACCGGAGGAAGGTGGGGATGACGTCAAG
TCATCATGGCCCTTACGAGTAGGGCTACACACGTGCTACAATGGCAGATACAAAGTGAA
GCGAACTCGCGAGAGCAAGCGGACCACATAAAGTCTGTCGTAGTCCGGATTGGAGTCT
GCAACTCGACTCCATGAAGTCGGAATCGCTAGTAATCGTAGATCAGAATGCTACGGTGA
ATACGTTCCCGGGCCTTGTACACACCGCCCGTCACACCATGGGAGTGGGTTGCAAAAG
AAGTAGGTAGCTTAACCTTCGGGAGGGCGCTTACCACTTTGTGATTCATGACTGGGG
pattern 155
CGCTGGCGGCAGGCCTAACACATGCAAGTCGAGCGGCAGCGGGAAGTAGTTTACTACT
TTGCCGGCGAGCGGCGGACGGGTGAGTAATGTCTGGGAAACTGCCTGATGGAGGGGGA
TAACTACTGGAAACGGTAGCTAATACCGCATGACCTCGTAAGAGCAAAGTGGGGGACCT
TCGGGCCTCACGCCATCGGATGTGCCCAGATGGGATTAGCTAGTAGGTGAGGTAATGGC
TCACCTAGGCGACGATCCCTAGCTGGTCTGAGAGGATGACCAGCCACACTGGAACTGA
GACACGGTCCAGACTCCTACGGGAGGCAGCAGTGGGGAATATTGCACAATGGGCGCAA
GCCTGATGCAGCCATGCCGCGTGTGTGAAGAAGGCCTTCGGGTTGTAAAGCACTTTCAG
CGAGGAGGAAGGCAGTCGTGTTAATAGCACGATTGATTGACGTTACTCGCAGAAGAAG
CACCGGCTAACTCCGTGCCAGCAGCCGCGGTAATACGGAGGGTGCAAGCGTTAATCGG
AATTACTGGGCGTAAAGCGCACGCAGGCGGTTTGTTAAGTCAGATGTGAAATCCCCGCG
CTTAACGTGGGAACTGCATTTGAAACTGGCAAGCTAGAGTCTTGTAGAGGGGGGTAGA
ATTCCAGGTGTAGCGGTGAAATGCGTAGAGATCTGGAGGAATACCGGTGGCGAAGGCG
GCCCCCTGGACAAAGACTGACGCTCAGGTGCGAAAGCGTGGGGAGCAAACAGGATTA
GATACCCTGGTAGTCCACGCTGTAAACGATGTCGACTTGGAGGTTGTGCCCTTGAGGCG
TGGCTTCCGGAGCTAACGCGTTAAGTCGACCGCCTGGGGAGTACGGCCGCAAGGTTAA
AACTCAAATGAATTGACGGGGGCCCGCACAAGCGGTGGAGCATGTGGTTTAATTCGATG

CAACGCGAAGAACCTTACCTACTCTTGACATCCACGGAATTTAGCAGAGATGCTTTAGT
GCCTTCGGGAACCGTGAGACAGGTGCTGCATGGCTGTCGTCAGCTCGTGTTGTGAAATG
TTGGGTTAAGTCCCGCAACGAGCGCAACCCTTATCCTTTGTTGCCAGCACGTAATGGTG
GGAACTCAAGGGAGACTGCCGGTGACAAACCGGAGGAAGGTGGGGATGACGTCAAGT
CATCATGGCCCTTACGAGTAGGGCTACACACGTGCTACAATGGCAGATACAAAGTGAAG
CGAACTCGCGAGAGCAAGCGGACCACATAAAGTCTGTCGTAGTCCGGATTGGAGTCTG
CAACTCGACTCCATGAAGTCGGAATCGCTAGTAATCGTAGATCAGAATGCTACGGTGAAT
ACGTTCCCGGGCCTTGTACACACCGCCCGTCACACCATGGGAGTGGGTTGCAAAAGAA
GTAGGTAGCTTAACCTTCGGGAGGGCGCTTACCACTTTGTGATTCATGACTGGGG
pattern 156
CGCTGGCGGCAGGCCTAACACATGCAAGTCGAGCGGCAGCGGGAAGTAGTTTACTACT
TTGCCGGCGAGCGGCGGACGGGTGAGTAATGTCTGGGAAACTGCCTGATGGAGGGGGA
TAACTACTGGAAACGGTAGCTAATACCGCATGACCTCGTAAGAGCAAAGTGGGGGACCT
TCGGGCCTCACGCCATCGGATGTGCCCAGATGGGATTAGCTAGTAGGTGGGGTAATGGC
TCACCTAGGCGACGATCCCTAGCTGGTCTGAGAGGATGACCAGCCACACTGGAACTGA
GACACGGTCCAGACTCCTACGGGAGGCAGCAGTGGGGAATATTGCACAATGGGCGCAA
GCCTGATGCAGCCATGCCGCGTGTGTGAAGAAGGCCTTCGGGTTGTAAAGCACTTTCAG
CGAGGAGGAAGGCAGTCGTGTTAATAGCACGATTGATTGACGTTACTCGCAGAAGAAG
CACCGGCTAACTCCGTGCCAGCAGCCGCGGTAATACGGAGGGTGCAAGCGTTAATCGG
AATTACTGGGCGTAAAGCGCACGCAGGCGGTTTGTTAAGTCAGATGTGAAATCCCCGCG
CTTAACGTGGGAACTGCATTTGAAACTGGCAAGCTAGAGTCTTGTAGAGGGGGGTAGA
ATTCCAGGTGTAGCGGTGAAATGCGTAGAGATCTGGAGGAATACCGGTGGCGAAGGCG
GCCCCCTGGACAAAGACTGACGCTCAGGTGCGAAAGCGTGGGGAGCAAACAGGATTA
GATACCCTGGTAGTCCACGCTGTAAACGATGTCGACTTGGAGGTTGTGCCCTTGAGGCG
TGGCTTCCGGAGCTAACGCGTTAAGTCGACCGCCTGGGGAGTACGGCCGCAAGGTTAA
AACTCAAATGAATTGACGGGGGCCCGCACAAGCGGTGGAGCATGTGGTTTAATTCGATG
CAACGCGAAGAACCTTACCTACTCTTGACATCCACGGAATTTAGCAGAGATGCTTTAGT
GCCTTCGGGAACTGTGAGACAGGTGCTGCATGGCTGTCGTCAGCTCGTGTTGTGAAATG
TTGGGTTAAGTCCCGCAACGAGCGCAACCCTTATCCTTTGTTGCCAGCACGTAATGGTG
GGAACTCAAGGGAGACTGCCGGTGACAAACCGGAGGAAGGTGGGGATGACGTCAAGT
CATCATGGCCCTTACGAGTAGGGCTACACACGTGCTACAATGGCAGATACAAAGTGAAG
CGAACTCGCGAGAGCAAGCGGACCACATAAAGTCTGTCGTAGTCCGGATTGGAGTCTG
CAACTCGACTCCATGAAGTCGGAATCGCTAGTAATCGTAGATCAGAATGCTACGGTGAAT
ACGTTCCCGGGCCTTGTACACACCGCCCGTCACACCATGGGAGTGGGTTGCAAAAGAA
GTAGGTAGCTTAACCTTCGGGAGGGCGCTTACCACTTTGTGATTCATGACTGGGG
pattern 157
CGCTGGCGGCAGGCCTAACACATGCAAGTCGAGCGGCAGCGGGAAGTAGTTTACTACT
TTGCCGGCGAGCGGCGGACGGGTGAGTAATGTCTGGGAAACTGCCTGATGGAGGGGGA
TAACTACTGGAAACGGTAGCTAATACCGCATGACCTCGTAAGAGCAAAGTGGGGGACCT
TCGGGCCTCACGCCATCGGATGTGCCCAGATGGGATTAGCTAGTAGGTGGGGTAATGGC
TCACCTAGGCGACGATCCCTAGCTGGTCTGAGAGGATGACCAGCCACACTGGAACTGA
GACACGGTCCAGACTCCTACGGGAGGCAGCAGTGGGGAATATTGCACAATGGGCGCAA
GCCTGATGCAGCCATGCCGCGTGTGTGAAGAAGGCCTTCGGGTTGTAAAGCACTTTCAG
CGAGGAGGAAGGCAGTCGTGTTAATAGCACGATTGATTGACGTTACTCGCAGAAGAAG

CACCGGCTAACTCCGTGCCAGCAGCCGCGGTAATACGGAGGGTGCAAGCGTTAATCGG
AATTACTGGGCGTAAAGCGCACGCAGGCGGTTTGTTAAGTCAGATGTGAAATCCCCGCG
CTTAACGTGGGAACTGCATTTGAAACTGGCAAGCTAGAGTCTTGTAGAGGGGGGTAGA
ATTCCAGGTGTAGCGGTGAAATGCGTAGAGATCTGGAGGAATACCGGTGGCGAAGGCG
GCCCCCTGGACAAAGACTGACGCTCAGGTGCGAAAGCGTGGGGAGCAAACAGGATTA
GATACCCTGGTAGTCCACGCTGTAAACGATGTCGACTTGGAGGTTGTGCCCTTGAGGCG
TGGCTTCCGGAGCTAACGCGTTAAGTCGACCGCCTGGGGAGTACGACCGCAAGGTTAA
AACTCAAATGAATTGACGGGGGCCCGCACAAGCGGTGGAGCATGTGGTTTAATTCGATG
CAACGCGAAGAACCTTACCTACTCTTGACATCCACGGAATTTAGCAGAGATGCTTTAGT
GCCTTCGGGAACCGTGAGACAGGTGCTGCATGGCTGTCGTCAGCTCGTGTTGTGAAATG
TTGGGTTAAGTCCCGCAACGAGCGCAACCCTTATCCTTTGTTGCCAGCACGTAATGGTG
GGAACTCAAGGGAGACTGCCGGTGACAAACCGGAGGAAGGTGGGGATGACGTCAAGT
CATCATGGCCCTTACGAGTAGGGCTACACACGTGCTACAATGGCAGATACAAAGTGAAG
CGAACTCGCGAGAGCAAGCGGACCACATAAAGTCTGTCGTAGTCCGGATTGGAGTCTG
CAACTCGACTCCATGAAGTCGGAATCGCTAGTAATCGTAGATCAGAATGCTACGGTGAAT
ACGTTCCCGGGCCTTGTACACACCGCCCGTCACACCATGGGAGTGGGTTGCAAAAGAA
GTAGGTAGCTTAACCTTCGGGAGGGCGCTTACCACTTTGTGATTCATGACTGGGG
pattern 158
CGCTGGCGGCAGGCCTAACACATGCAAGTCGAGCGGCAGCGGGAAGTAGTTTACTACT
TTGCCGGCGAGCGGCGGACGGGTGAGTAATGTCTGGGAAACTGCCTGATGGAGGGGGA
TAACTACTGGAAACGGTAGCTAATACCGCATGACCTCGTAAGAGCAAAGTGGGGGACCT
TCGGGCCTCACGCCATCGGATGTGCCCAGATGGGATTAGCTAGTAGGTGGGGTAATGGC
TCACCTAGGCGACGATCCCTAGCTGGTCTGAGAGGATGACCAGCCACACTGGAACTGA
GACACGGTCCAGACTCCTACGGGAGGCAGCAGTGGGGAATATTGCACAATGGGCGCAA
GCCTGATGCAGCCATGCCGCGTGTGTGAAGAAGGCCTTCGGGTTGTAAAGCACTTTCAG
CGAGGAGGAAGGCAGTCGTGTTAATAGCACGATTGATTGACGTTACTCGCAGAAGAAG
CACCGGCTAACTCCGTGCCAGCAGCCGCGGTAATACGGAGGGTGCAAGCGTTAATCGG
AATTACTGGGCGTAAAGCGCACGCAGGCGGTTTGTTAAGTCAGATGTGAAATCCCCGCG
CTTAACGTGGGAACTGCATTTGAAACTGGCAAGCTAGAGTCTTGTAGAGGGGGGTAGA
ATTCCAGGTGTAGCGGTGAAATGCGTAGAGATCTGGAGGAATACCGGTGGCGAAGGCG
GCCCCCTGGACAAAGACTGACGCTCAGGTGCGAAAGCGTGGGGAGCAAACAGGATTA
GATACCCTGGTAGTCCACGCTGTAAACGATGTCGACTTGGAGGTTGTGCCCTTGAGGCG
TGGCTTCCGGAGCTAACGCGTTAAGTCGACCGCCTGGGGAGTACGGCCGCAAGGTTAA
AACTCAAATGAATTGACGGGGGCCCGCACAAGCGGTGGAGCATGTGGTTTAATTCGATG
CAACGCGAAGAACCTTACCTACTCTTGACATCCACGGAATTTAGCAGAGATGCTTTAGT
GCCTTCGGGAACCGTGAGACAGGTGCTGCATGGCTGTCGTCAGCTCGTGTTGTGAAATG
TTGGGTTAAGTCCCGCAACGAGCGCAACCCTTATCCTTTGTTGCCAGCACGTAATGGTG
GGAACTCAAGGGAGACTGCCGGTGACAAACCGGAGGAAGGTGGGGATGACGTCAAGT
CATCATGGCCCTTACGAGTAGGGCTACACACGTGCTACAATGGCAGATACAAAGTGAAG
CGAACTCGCGAGAGCAAGCGGACCACATAAAGTCTGTCGTAGTCCGGATTGGAGTCTA
CAACTCGACTCCATGAAGTCGGAATCGCTAGTAATCGTAGATCAGAATGCTACGGTGAAT
ACGTTCCCGGGCCTTGTACACACCGCCCGTCACACCATGGGAGTGGGTTGCAAAAGAA
GTAGGTAGCTTAACCTTCGGGAGGGCGCTTACCACTTTGTGATTCATGACTGGGG
pattern 159

CGCTGGCGGCAGGCCTAACACATGCAAGTCGAGCGGCAGCGGGAAGTAGTTTACTACT
TTGCCGGCGAGCGGCGGACGGGTGAGTAATGTCTGGGAAACTGCCTGATGGAGGGGGA
TAACTACTGGAAACGGTAGCTAATACCGCATGACCTCGTAAGAGCAAAGTGGGGGACCT
TCGGGCCTCACGCCATCGGATGTGCCCAGATGGGATTAGCTAGTAGGTGAGGTAATGGC
TCACCTAGGCGACGATCCCTAGCTGGTCTGAGAGGATGACCAGCCACACTGGAACTGA
GACACGGTCCAGACTCCTACGGGAGGCAGCAGTGGGGAATATTGCACAATGGGCGCAA
GCCTGATGCAGCCATGCCGCGTGTGTGAAGAAGGCCTTCGGGTTGTAAAGCACTTTCAG
CGAGGAGGAAGGCAGTCGTGTTAATAGCACGATTGATTGACGTTACTCGCAGAAGAAG
CACCGGCTAACTCCGTGCCAGCAGCCGCGGTAATACGGAGGGTGCAAGCGTTAATCGG
AATTACTGGGCGTAAAGCGCACGCAGGCGGTTTGTTAAGTCAGATGTGAAATCCCCGCG
CTTAACGTGGGAACTGCATTTGAAACTGGCAAGCTAGAGTCTTGTAGAGGGGGGTAGA
ATTCCAGGTGTAGCGGTGAAATGCGTAGAGATCTGGAGGAATACCGGTGGCGAAGGCG
GCCCCCTGGACAAAGACTGACGCTCAGGTGCGAAAGCGTGGGGAGCAAACAGGATTA
GATACCCTGGTAGTCCACGCTGTAAACGATGTCGACTTGGAGGTTGTGCCCTTGAGGCG
TGGCTTCCGGAGCTAACGCGTTAAGTCGACCGCCTGGGGAGTACGACCGCAAGGTTAA
AACTCAAATGAATTGACGGGGGCCCGCACAAGCGGTGGAGCATGTGGTTTAATTCGATG
CAACGCGAAGAACCTTACCTACTCTTGACATCCACGGAATTTAGCAGAGATGCTTTAGT
GCCTTCGGGAACCGTGAGACAGGTGCTGCATGGCTGTCGTCAGCTCGTGTTGTGAAATG
TTGGGTTAAGTCCCGCAACGAGCGCAACCCTTATCCTTTGTTGCCAGCACGTAATGGTG
GGAACTCAAGGGAGACTGCCGGTGACAAACCGGAGGAAGGTGGGGATGACGTCAAGT
CATCATGGCCCTTACGAGTAGGGCTACACACGTGCTACAATGGCAGATACAAAGTGAAG
CGAACTCGCGAGAGCAAGCGGACCACATAAAGTCTGTCGTAGTCCGGATTGGAGTCTG
CAACTCGACTCCATGAAGTCGGAATCGCTAGTAATCGTAGATCAGAATGCTACGGTGAAT
ACGTTCCCGGGCCTTGTACACACCGCCCGTCACACCATGGGAGTGGGTTGCAAAAGAA
GTAGGTAGCTTAACCTTCGGGAGGGCGCTTACCACTTTGTGATTCATGACTGGGG
pattern 160
CGCTGGCGGCAGGCCTAACACATGCAAGTCGAGCGGCAGCGGGAAGTAGTTTACTACT
TTGCCGGCGAGCGGCGGACGGGTGAGTAATGTCTGGGAAACTGCCTGATGGAGGGGGA
TAACTACTGGAAACGGTAGCTAATACCGCATGACCTCGTAAGAGCAAAGTGGGGGACCT
TCGGGCCTCACGCCATCGGATGTGCCCATATGGGATTAGCTAGTAGGTGGGGTAATGGCT
CACCTAGGCGACGATCCCTAGCTGGTCTGAGAGGATGACCAGCCACACTGGAACTGAG
ACACGGTCCAGACTCCTACGGGAGGCAGCAGTGGGGAATATTGCACAATGGGCGCAAG
CCTGATGCAGCCATGCCGCGTGTGTGAAGAAGGCCTTCGGGTTGTAAAGCACTTTCAGC
GAGGAGGAAGGCAGTCGTGTTAATAGCACGATTGATTGACGTTACTCGCAGAAGAAGC
ACCGGCTAACTCCGTGCCAGCAGCCGCGGTAATACGGAGGGTGCAAGCGTTAATCGGA
ATTACTGGGCGTAAAGCGCACGCAGGCGGTTTGTTAAGTCAGATGTGAAATCCCCGCGC
TTAACGTGGGAACTGCATTTGAAACTGGCAAGCTAGAGTCTTGTAGAGGGGGGTAGAA
TTCCAGGTGTAGCGGTGAAATGCGTAGAGATCTGGAGGAATACCGGTGGCGAAGGCGG
CCCCCTGGACAAAGACTGACGCTCAGGTGCGAAAGCGTGGGGAGCAAACAGGATTAG
ATACCCTGGTAGTCCACGCTGTAAACGATGTCGACTTGGAGGTTGTGCCCTTGAGGCGT
GGCTTCCGGAGCTAACGCGTTAAGTCGACCGCCTGGGGAGTACGGCCGCAAGGTTAAA
ACTCAAATGAATTGACGGGGGCCCGCACAAGCGGTGGAGCATGTGGTTTAATTCGATGC
AACGCGAAGAACCTTACCTACTCTTGACATCCACGGAATTTAGCAGAGATGCTTTAGTG
CCTTCGGGAACCGTGAGACAGGTGCTGCATGGCTGTCGTCAGCTCGTGTTGTGAAATGT

TGGGTTAAGTCCCGCAACGAGCGCAACCCTTATCCTTTGTTGCCAGCACGTAATGGTGG
GAACTCAAGGGAGACTGCCGGTGACAAACCGGAGGAAGGTGGGGATGACGTCAAGTC
ATCATGGCCCTTACGAGTAGGGCTACACACGTGCTACAATGGCAGATACAAAGTGAAGC
GAACTCGCGAGAGCAAGCGGACCACATAAAGTCTGTCGTAGTCCGGATTGGAGTCTGC
AACTCGACTCCATGAAGTCGGAATCGCTAGTAATCGTAGATCAGAATGCTACGGTGAAT
ACGTTCCCGGGCCTTGTACACACCGCCCGTCACACCATGGGAGTGGGTTGCAAAAGAA
GTAGGTAGCTTAACCTTCGGGAGGGCGCTTACCACTTTGTGATTCATGACTGGGG
pattern 161
CGCTGGCGGCAGGCCTAACACATGCAAGTCGAGCGGCAGCGGAAAGTAGCTTGCTACT
TTGCCGGCGAGCGGCGGACGGGTGAGTAATGTCTGGGAAACTGCCTGATGGAGGGGGA
TAACTACTGGAAACGGTAGCTAATACCGCATGACCTCGCAAGAGCAAAGTGGGGGACC
TTCGGGCCTCACGCCATCGGATGTGCCCAGATGGGATTAGCTAGTAGGTGGGGTAATGG
CTCACCTAGGCGACGATCCCTAGCTGGTCTGAGAGGATGACCAGCCACACTGGAACTG
AGACACGGTCCAGACTCCTACGGGAGGCAGCAGTGGGGAATATTGCACAATGGGCGCA
AGCCTGATGCAGCCATGCCGCGTGTGTGAAGAAGGCCTTCGGGTTGTAAAGCACTTTCA
GCGAGGAGGAAGGCAGTCGTGTTAATAGCACGATTGATTGACGTTACTCGCAGAAGAA
GCACCGGCTAACTCCGTGCCAGCAGCCGCGGTAATACGGAGGGTGCAAGCGTTAATCG
GAATTACTGGGCGTAAAGCGCACGCAGGCGGTTTGTTAAGTCAGATGTGAAATCCCCGC
GCTTAACGTGGGAACTGCATTTGAAACTGGCAAGCTAGAGTCTTGTAGAGGGGGGTAG
AATTCCAGGTGTAGCGGTGAAATGCGTAGAGATCTGGAGGAATACCGGTGGCGAAGGC
GGCCCCCTGGACAAAGACTGACGCTCAGGTGCGAAAGCGTGGGGAGCAAACAGGATT
AGATACCCTGGTAGTCCACGCTGTAAACGATGTCGACTTGGAGGTTGTGCCCTTGAGGC
GTGGCTTCCGGAGCTAACGCGTTAAGTCGACCGCCTGGGGAGTACGGCCGCAAGGTTA
AAACTCAAATGAATTGACGGGGGCCCGCACAAGCGGTGGAGCATGTGGTTTAATTCGAT
GCAACGCGAAGAACCTTACCTACTCTTGACATCCACAGAACTTAGCAGAGATGCTTCGG
TGCCTTCGGGAACTGTGAGACAGGTGCTGCATGGCCGTCGTCAGCTCGTGTTGTGAAAT
GTTGGGTTAAGTCCCGCAACGAGCGCAACCCTTATCCTTTGTTGCCAGCACGTAATGGT
GGGAACTCAAGGGAGACTGCCGGTGACAAACCGGAGGAAGGTGGGGATGACGTCAAG
TCATCATGGCCCTTACGAGTAGGGCTACACACGTGCTACAATGGCAGATACAAAGTGAA
GCGAGCTCGCGAGAGTCAGCGGACCACATAAAGTCTGTCGTAGTCCGGATTGGAGTCT
GCAACTCGACTCCATGAAGTCGGAATCGCTAGTAATCGTAGATCAGAATGCTACGGTGA
ATACGTTCCCGGGCCTTGTACACACCGCCCGTCACACCATGGGAGTGGGTTGCAAAAG
AAGTAGGTAGCTTAACCTTCGGGAGGGCGCTTACCACTTTGTGATTCATGACTGGGG
pattern 162
CGCTGGCGGCAGGCCTAACACATGCAAGTCGAGCGGCAGCGGAAAGTAGCTTGCTACT
TTGCCGGCGAGCGGCGGACGGGTGAGTAATGTCTGGGAAACTGCCTGATGGAGGGGGA
TAACTACTGGAAACGGTAGCTAATACCGCATGACCTCGCAAGAGCAAAGTGGGGGACC
TTCGGGCCTCACGCCATCGGATGTGCCCAGATGGGATTAGCTAGTAGGTGGGGTAATGG
CTCACCTAGGCGACGATCCCTAGCTGGTCTGAGAGGATGACCAGCCACACTGGAACTG
AGACACGGTCCAGACTCCTACGGGAGGCAGCAGTGGGGAATATTGCACAATGGGCGCA
AGCCTGATGCAGCCATGCCGCGTGTGTGAAGAAGGCCTTCGGGTTGTAAAGCACTTTCA
GCGAGGAGGAAGGCAGTCGTGTTAATAGCACGATTGATTGACGTTACTCGCAGAAGAA
GCACCGGCTAACTCCGTGCCAGCAGCCGCGGTAATACGGAGGGTGCAAGCGTTAATCG
GAATTACTGGGCGTAAAGCGCACGCAGGCGGTTTGTTAAGTCAGATGTGAAATCCCCGC

GCTTAACGTGGGAACTGCATTTGAAACTGGCAAGCTAGAGTCTTGTAGAGGGGGGTAG
AATTCCAGGTGTAGCGGTGAAATGCGTAGAGATCTGGAGGAATACCGGTGGCGAAGGC
GGCCCCCTGGACAAAGACTGACGCTCAGGTGCGAAAGCGTGGGGAGCAAACAGGATT
AGATACCCTGGTAGTCCACGCTGTAAACGATGTCGACTTGGAGGTTGTGCCCTTGAGGC
GTGGCTTCCGGAGCTAACGCGTTAAGTCGACCGCCTGGGGAGTACGGCCGCAAGGTTA
AAACTCAAATGAATTGACGGGGGCCCGCACAAGCGGTGGAGCATGTGGTTTAATTCGAT
GCAACGCGAAGAACCTTACCTACTCTTGACATCCACAGAACTTAGCAGAGATGCTTCGG
TGCCTTCGGGAACTGTGAGACAGGTGCTGCATGGCTGTCGTCAGCTCGTGTTGTGAAAT
GTTGGGTTAAGTCCCGCAACGAGCGCAACCCTTATCCTTTGTTGCCAGCACGTAATGGT
GGGAACTCAAGGGAGACTGCCGGTGACAAACCGGAGGAAGGTGGGGATGACGTCAAG
TCATCATGGCCCTTACGAGTAGGGCTACACACGTGCTACAATGGCAGATACAAAGTGAA
GCGAGCTCGCGAGAGTCAGCGGACCACATAAAGTCTGTCGTAGTCCGGATTGGAGTCT
GCAACTCGACTCCATGAAGTCGGAATCGCTAGTAATCGTAGATCAGAATGCTACGGTGA
ATACGTTCCCGGGCCTTGTACACACCGCCCGTCACACCATGGGAGTGGGTTGCAAAAG
AAGTAGGTAGCTTAACCTTCGGGAGGGCGCTTACCACTTTGTGATTCATGACTGGGG
pattern 163
CGCTGGCGGCAGGCCTAACACATGCAAGTCGAGCGGCAGCGGAAAGTAGCTTGCTACT
TTGCCGGCGAGCGGCGGACGGGTGAGTAATGTCTGGGAAACTGCCTGATGGAGGGGGA
TAACTACTGGAAACGGTAGCTAATACCGCATGACCTCGCAAGAGCAAAGTGGGGGACC
TTCGGGCCTCACGCCATCGGATGTGCCCAGATGGGATTAGCTAGTAGGTGGGGTAATGG
CTCACCTAGGCGACGATCCCTAGCTGGTCTGAGAGGATGACCAGCCACACTGGAACTG
AGACACGGTCCAGACTCCTACGGGAGGCAGCAGTGGGGAATATTGCACAATGGGCGCA
AGCCTGATGCAGCCATGCCGCGTGTGTGAAGAAGGCCTTCGGGTTGTAAAGCACTTTCA
GCGAGGAGGAAGGCAGTCGTGTTAATAGCACGATTGATTGACGTTACTCGCAGAAGAA
GCACCGGCTAACTCCGTGCCAGCAGCCGCGGTAATACGGAGGGTGCAAGCGTTAATCG
GAATTACTGGGCGTAAAGCGCACGCAGGCGGTTTGTTAAGTCAGATGTGAAATCCCCGC
GCTTAACGTGGGAACTGCATTTGAAACTGGCAAGCTAGAGTCTTGTAGAGGGGGGTAG
AATTCCAGGTGTAGCGGTGAAATGCGTAGAGATCTGGAGGAATACCGGTGGCGAAGGC
GGCCCCCTGGACAAAGACTGACGCTCAGGTGCGAAAGCGTGGGGAGCAAACAGGATT
AGATACCCTGGTAGTCCACGCTGTAAACGATGTCGACTTGGAGGTTGTGCCCTTGAGGC
GTGGCTTCCGGAGCTAACGCGTTAAGTCGACCGCCTGGGGAGTACGGCCGCAAGGTTA
AAACTCAAATGAATTGACGGGGGCCCGCACAAGCGGTGGAGCATGTGGTTTAATTCGAT
GCAACGCGAAGAACCTTACCTACTCTTGACATCCACAGAACTTAGCAGAGATGCTTCGG
TGCCTTCGGGAACTGTGAGACAGGTGCTGCATGGCTGTCGTCAGCTCGTGTTGTGAAAT
GTTGGGTTAAGTCCCGCAACGAGCGCAACCCTTATCCTTTGTTGCCAGCACGTAATGGT
GGGAACTCAAGGGAGACTGCCGGTGACAAACCGGAGGAAGGTGGGGATGACGTCAAG
TCATCATGGCCCTTACGAGTAGGGCTACACACGTGCTACAATGGCAGATACAAAGTGAA
GCGAACTCGCGAGAGTCAGCGGACCACATAAAGTCTGTCGTAGTCCGGATTGGAGTCT
GCAACTCGACTCCATGAAGTCGGAATCGCTAGTAATCGTAGATCAGAATGCTACGGTGA
ATACGTTCCCGGGCCTTGTACACACCGCCCGTCACACCATGGGAGTGGGTTGCAAAAG
AAGTAGGTAGCTTAACCTTCGGGAGGGCGCTTACCACTTTGTGATTCATGACTGGGG
pattern 164
CGCTGGCGGCAGGCCTAACACATGCAAGTCGAGCGGCAGCGGAAAGTAGCTTGCTACT
TTGCCGGCGAGCGGCGGACGGGTGAGTAATGTCTGGGGATCTGCCTGATGGAGGGGGA

TAACTACTGGAAACGGTAGCTAATACCGCATGACCTCGAAAGAGCAAAGTGGGGGACC
TTCGGGCCTCACGCCATCGGATGAACCCAGATGGGATTAGCTAGTAGGTGAGGTAATGG
CTCACCTAGGCGACGATCCCTAGCTGGTCTGAGAGGATGACCAGCCACACTGGAACTG
AGACACGGTCCAGACTCCTACGGGAGGCAGCAGTGGGGAATATTGCACAATGGGCGCA
AGCCTGATGCAGCCATGCCGCGTGTGTGAAGAAGGCCTTCGGGTTGTAAAGCACTTTCA
GCGAGGAGGAAGGCATTGTGGTTAATAACCACAGTGATTGACGTTACTCGCAGAAGAA
GCACCGGCTAACTCCGTGCCAGCAGCCGCGGTAATACGGAGGGTGCAAGCGTTAATCG
GAATTACTGGGCGTAAAGCGCACGCAGGCGGTTTGTTAAGTCAGATGTGAAATCCCCGC
GCTTAACGTGGGAACTGCATTTGAAACTGGCAAGCTAGAGTCTTGTAGAGGGGGGTAG
AATTCCAGGTGTAGCGGTGAAATGCGTAGAGATCTGGAGGAATACCGGTGGCGAAGGC
GGCCCCCTGGACAAAGACTGACGCTCAGGTGCGAAAGCGTGGGGAGCAAACAGGATT
AGATACCCTGGTAGTCCACGCTGTAAACGATGTCGACTTGGAGGTTGTGCCCTTGAGGC
GTGGCTTCCGGAGCTAACGCGTTAAGTCGACCGCCTGGGGAGTACGGCCGCAAGGTTA
AAACTCAAATGAATTGACGGGGGCCCGCACAAGCGGTGGAGCATGTGGTTTAATTCGAT
GCAACGCGAAGAACCTTACCTACTCTTGACATCCACAGAACTTAGCAGAGATGCTTCGG
TGCCTTCGGGAACTGTGAGACAGGTGCTGCATGGCTGTCGTCAGCTCGTGTTGTGAAAT
GTTGGGTTAAGTCCCGCAACGAGCGCAACCCTTATCCTTTGTTGCCAGCACGTAATGGT
GGGAACTCAAGGGAGACTGCCGGTGACAAACCGGAGGAAGGTGGGGATGACGTCAAG
TCATCATGGCCCTTACGAGTAGGGCTACACACGTGCTACAATGGCAGATACAAAGTGAA
GCGAACTCGCGAGAGCAAGCGGACCACATAAAGTCTGTCGTAGTCCGGATTGGAGTCT
GCAACTCGACTCCATGAAGTCGGAATCGCTAGTAATCGTAGATCAGAATGCTACGGTGA
ATACGTTCCCGGGCCTTGTACACACCGCCCGTCACACCATGGGAGTGGGTTGCAAAAG
AAGTAGGTAGCTTAACCTTCGGGAGGGCGCTTACCACTTTGTGATTCATGACTGGGG
pattern 165
CGCTGGCGGCAGGCCTAACACATGCAAGTCGAGCGGCAGCGGAAAGTAGCTTGCTACT
TTGCCGGCGAGCGGCGGACGGGTGAGTAATGTCTGGGGATCTGCCTGATGGAGGGGGA
TAACTACTGGAAACGGTAGCTAATACCGCATGACCTCGAAAGAGCAAAGTGGGGGACC
TTCGGGCCTCACGCCATCGGATGAACCCAGATGGGATTAGCTAGTAGGTGGGGTAATGG
CTCACCTAGGCGACGATCCCTAGCTGGTCTGAGAGGATGACCAGCCACACTGGAACTG
AGACACGGTCCAGACTCCTACGGGAGGCAGCAGTGGGGAATATTGCACAATGGGCGCA
AGCCTGATGCAGCCATGCCGCGTGTGTGAAGAAGGCCTTCGGGTTGTAAAGCACTTTCA
GCGAGGAGGAAGGCATTGTGGTTAATAACCACAGTGATTGACGTTACTCGCAGAAGAA
GCACCGGCTAACTCCGTGCCAGCAGCCGCGGTAATACGGAGGGTGCAAGCGTTAATCG
GAATTACTGGGCGTAAAGCGCACGCAGGCGGTTTGTTAAGTCAGATGTGAAATCCCCGC
GCTTAACGTGGGAACTGCATTTGAAACTGGCAAGCTAGAGTCTTGTAGAGGGGGGTAG
AATTCCAGGTGTAGCGGTGAAATGCGTAGAGATCTGGAGGAATACCGGTGGCGAAGGC
GGCCCCCTGGACAAAGACTGACGCTCAGGTGCGAAAGCGTGGGGAGCAAACAGGATT
AGATACCCTGGTAGTCCACGCTGTAAACGATGTCGACTTGGAGGTTGTGCCCTTGAGGC
GTGGCTTCCGGAGCTAACGCGTTAAGTCGACCGCCTGGGGAGTACGGCCGCAAGGTTA
AAACTCAAATGAATTGACGGGGGCCCGCACAAGCGGTGGAGCATGTGGTTTAATTCGAT
GCAACGCGAAGAACCTTACCTACTCTTGACATCCGCGGAATTTAGCAGAGATGCTTTAG
TGCCTTCGGGAACCGTGAGACAGGTGCTGCATGGCTGTCGTCAGCTCGTGTTGTGAAAT
GTTGGGTTAAGTCCCGCAACGAGCGCAACCCTTATCCTTTGTTGCCAGCACGTAATGGT
GGGAACTCAAGGGAGACTGCCGGTGACAAACCGGAGGAAGGTGGGGATGACGTCAAG

TCATCATGGCCCTTACGAGTAGGGCTACACACGTGCTACAATGGCAGATACAAAGTGAA
GCGAACTCGCGAGAGCAAGCGGACCACATAAAGTCTGTCGTAGTCCGGATTGGAGTCT
GCAACTCGACTCCATGAAGTCGGAATCGCTAGTAATCGTAGATCAGAATGCTACGGTGA
ATACGTTCCCGGGCCTTGTACACACCGCCCGTCACACCATGGGAGTGGGTTGCAAAAG
AAGTAGGTAGCTTAACCTTCGGGAGGGCGCTTACCACTTTGTGATTCATGACTGGGG
pattern 166
CGCTGGCGGCAGGCCTAACACATGCAAGTCGAGCGGCAGCGGAAAGTAGCTTGCTACT
TTGCCGGCGAGCGGCGGACGGGTGAGTAATGTCTGGGAAACTGCCTGATGGAGGGGGA
TAACTACTGGAAACGGTAGCTAATACCGCATGACCTCGAAAGAGCAAAGTGGGGGACC
TTCGGGCCTCACGCCATCGGATGTGCCCAGATGGGATTAGCTAGTAGGTGAGGTAATGG
CTCACCTAGGCGACGATCCCTAGCTGGTCTGAGAGGATGACCAGCCACACTGGAACTG
AGACACGGTCCAGACTCCTACGGGAGGCAGCAGTGGGGAATATTGCACAATGGGCGCA
AGCCTGATGCAGCCATGCCGCGTGTGTGAAGAAGGCCTTCGGGTTGTAAAGCACTTTCA
GCGAGGAGGAAGGCATTTCACTTAATACGTGAGGTGATTGACGTTACTCGCAGAAGAA
GCACCGGCTAACTCCGTGCCAGCAGCCGCGGTAATACGGAGGGTGCAAGCGTTAATCG
GAATTACTGGGCGTAAAGCGCACGCAGGCGGTTTGTTAAGTCAGATGTGAAATCCCCGA
GCTTAACTTGGGAACTGCATTTGAAACTGGCAAGCTAGAGTCTTGTAGAGGGGGGTAG
AATTCCAGGTGTAGCGGTGAAATGCGTAGAGATCTGGAGGAATACCGGTGGCGAAGGC
GGCCCCCTGGACAAAGACTGACGCTCAGGTGCGAAAGCGTGGGGAGCAAACAGGATT
AGATACCCTGGTAGTCCACGCTGTAAACGATGTCGACTTGGAGGTTGTGCCCTTGAGGC
GTGGCTTCCGGAGCTAACGCGTTAAGTCGACCGCCTGGGGAGTACGGCCGCAAGGTTA
AAACTCAAATGAATTGACGGGGGCCCGCACAAGCGGTGGAGCATGTGGTTTAATTCGAT
GCAACGCGAAGAACCTTACCTACTCTTGACATCCACAGAACTGAGCAGAGATGCTTAG
GTGCCTTCGGGAACTGTGAGACAGGTGCTGCATGGCTGTCGTCAGCTCGTGTTGTGAAA
TGTTGGGTTAAGTCCCGCAACGAGCGCAACCCTTATCCTTTGTTGCCAGCACGTGATGG
TGGGAACTCAAAGGAGACTGCCGGTGATAAACCGGAGGAAGGTGGGGATGACGTCAA
GTCATCATGGCCCTTACGAGTAGGGCTACACACGTGCTACAATGGCAGATACAAAGTGA
AGCGAACTCGCGAGAGCAAGCGGACCACATAAAGTCTGTCGTAGTCCGGATTGGAGTC
TGCAACTCGACTCCATGAAGTCGGAATCGCTAGTAATCGTAGATCAGAATGCTACGGTG
AATACGTTCCCGGGCCTTGTACACACCGCCCGTCACACCATGGGAGTGGGTTGCAAAA
GAAGTAGGTAGCTTAACCTTCGGGAGGGCGCTTACCACTTTGTGATTCATGACTGGGG
pattern 167
CGCTGGCGGCAGGCCTAACACATGCAAGTCGAGCGGCAGCGGGAAGTAGTTTACTACT
TCGCCGGCGAGCGGCGGACGGGTGAGTAATGTCTGGGAAACTGCCTGATGGAGGGGGA
TAACTACTGGAAACGGTAGCTAATACCGCATGACCTCGCAAGAGCAAAGTGGGGGACC
TTCGGGCCTCACGCCATCGGATGTGCCCAGATGGGATTAGCTAGTAGGTGGGGTAATGG
CTCACCTAGGCGACGATCCCTAGCTGGTCTGAGAGGATGACCAGCCACACTGGAACTG
AGACACGGTCCAGACTCCTACGGGAGGCAGCAGTGGGGAATATTGCACAATGGGCGCA
AGCCTGATGCAGCCATGCCGCGTGTGTGAAGAAGGCCTTCGGGTTGTAAAGCACTTTCA
GCGAGGAGGAAGGGTTCAGTGTTAATAGCACTGAGCATTGACGTTACTCGCAGAAGAA
GCACCGGCTAACTCCGTGCCAGCAGCCGCGGTAATACGGAGGGTGCAAGCGTTAATCG
GAATTACTGGGCGTAAAGCGCACGCAGGCGGTTTGTTAAGTCAGATGTGAAATCCCCGC
GCTTAACGTGGGAACTGCATTTGAAACTGGCAAGCTAGAGTCTTGTAGAGGGGGGTAG
AATTCCAGGTGTAGCGGTGAAATGCGTAGAGATCTGGAGGAATACCGGTGGCGAAGGC

GGCCCCCTGGACAAAGACTGACGCTCAGGTGCGAAAGCGTGGGGAGCAAACAGGATT
AGATACCCTGGTAGTCCACGCTGTAAACGATGTCGACTTGGAGGTTGTGCCCTTGAGGC
GTGGCTTCCGGAGCTAACGCGTTAAGTCGACCGCCTGGGGAGTACGGCCGCAAGGTTA
AAACTCAAATGAATTGACGGGGGCCCGCACAAGCGGTGGAGCATGTGGTTTAATTCGAT
GCAACGCGAAGAACCTTACCTACTCTTGACATCCACAGAACTTAGCAGAGATGCTTCGG
TGCCTTCGGGAACTGTGAGACAGGTGCTGCATGGCTGTCGTCAGCTCGTGTTGTGAAAT
GTTGGGTTAAGTCCCGCAACGAGCGCAACCCTTATCCTTTGTTGCCAGCACGTAATGGT
GGGAACTCAAGGGAGACTGCCGGTGACAAACCGGAGGAAGGTGGGGATGACGTCAAG
TCATCATGGCCCTTACGAGTAGGGCTACACACGTGCTACAATGGCAGATACAAAGTGAA
GCGAACTCGCGAGAGCAAGCGGACCACATAAAGTCTGTCGTAGTCCGGATTGGAGTCT
GCAACTCGACTCCATGAAGTCGGAATCGCTAGTAATCGTAGATCAGAATGCTACGGTGA
ATACGTTCCCGGGCCTTGTACACACCGCCCGTCACACCATGGGAGTGGGTTGCAAAAG
AAGTAGGTAGCTTAACCTTCGGGAGGGCGCTTACCACTTTGTGATTCATGACTGGGG

pattern 168

CGCTGGCGGCAGGCCTAACACATGCAAGTCGAGCGGCAGCGGGAAGTAGTTTACTACT
TTGCCGGCGAGCGGCGGACGGGTGAGTAATGTCTGGGAAACTGCCTGATGGAGGGGGA
TAACTACTGGAAACGGTAGCTAATACCGCATGACCTCGCAAGAGCAAAGTGGGGGACC
TTCGGGCCTCACGCCATCGGATGTGCCCAGATGGGATTAGCTAGTAGGTGGGGTAATGG
CTCACCTAGGCGACGATCCCTAGCTGGTCTGAGAGGATGACCAGCCACACTGGAACTG
AGACACGGTCCAGACTCCTACGGGAGGCAGCAGTGGGGAATATTGCACAATGGGCGCA
AGCCTGATGCAGCCATGCCGCGTGTGTGAAGAAGGCCTTCGGGTTGTAAAGCACTTTCA
GCGAGGAGGAAGGGTTCAGTGTTAATAGCACTGAGCATTGACGTTACTCGCAGAAGAA
GCACCGGCTAACTCCGTGCCAGCAGCCGCGGTAATACGGAGGGTGCAAGCGTTAATCG
GAATTACTGGGCGTAAAGCGCACGCAGGCGGTTTGTTAAGTCAGATGTGAAATCCCCGC
GCTTAACGTGGGAACTGCATTTGAAACTGGCAAGCTAGAGTCTTGTAGAGGGGGGTAG
AATTCCAGGTGTAGCGGTGAAATGCGTAGAGATCTGGAGGAATACCGGTGGCGAAGGC
GGCCCCCTGGACAAAGACTGACGCTCAGGTGCGAAAGCGTGGGGAGCAAACAGGATT
AGATACCCTGGTAGTCCACGCTGTAAACGATGTCGACTTGGAGGTTGTGCCCTTGAGGC
GTGGCTTCCGGAGCTAACGCGTTAAGTCGACCGCCTGGGGAGTACGGCCGCAAGGTTA
AAACTCAAATGAATTGACGGGGGCCCGCACAAGCGGTGGAGCATGTGGTTTAATTCGAT
GCAACGCGAAGAACCTTACCTACTCTTGACATCCACAGAACTTAGCAGAGATGCTTCGG
TGCCTTCGGGAACTGTGAGACAGGTGCTGCATGGCTGTCGTCAGCTCGTGTTGTGAAAT
GTTGGGTTAAGTCCCGCAACGAGCGCAACCCTTATCCTTTGTTGCCAGCACGTAATGGT
GGGAACTCAAGGGAGACTGCCGGTGACAAACCGGAGGAAGGTGGGGATGACGTCAAG
TCATCATGGCCCTTACGAGTAGGGCTACACACGTGCTACAATGGCAGATACAAAGTGAA
GCGAACTCGCGAGAGCAAGCGGACCACATAAAGTCTGTCGTAGTCCGGATTGGAGTCT
GCAACTCGACTCCATGAAGTCGGAATCGCTAGTAATCGTAGATCAGAATGCTACGGTGA
ATACGTTCCCGGGCCTTGTACACACCGCCCGTCACACCATGGGAGTGGGTTGCAAAAG
AAGTAGGTAGCTTAACCTTCGGGAGGGCGCTTACCACTTTGTGATTCATGACTGGGG

pattern 169

CGCTGGCGGCAGGCCTAACACATGCAAGTCGAGCGGCAGCGGGAAGTAGTTTACTACT
TTGCCGGCGAGCGGCGGACGGGTGAGTAATGTCTGGGAAACTGCCTGATGGAGGGGGA
TAACTACTGGAAACGGTAGCTAATACCGCATGACCTCGAAAGAGCAAAGTGGGGGACC
TTCGGGCCTCACGCCATCGGATGTGCCCAGATGGGATTAGCTAGTAGGTGGGGTAATGG

CTCACCTAGGCGACGATCCCTAGCTGGTCTGAGAGGATGACCAGCCACACTGGAACTG
AGACACGGTCCAGACTCCTACGGGAGGCAGCAGTGGGGAATATTGCACAATGGGCGCA
AGCCTGATGCAGCCATGCCGCGTGTGTGAAGAAGGCCTTCGGGTTGTAAAGCACTTTCA
GCGAGGAGGAAGGCATAAAGGTTAATAACCTTTGTGATTGACGTTACTCGCAGAAGAA
GCACCGGCTAACTCCGTGCCAGCAGCCGCGGTAATACGGAGGGTGCAAGCGTTAATCG
GAATTACTGGGCGTAAAGCGCACGCAGGCGGTTTGTTAAGTCAGATGTGAAATCCCCGC
GCTTAACGTGGGAACTGCATTTGAAACTGGCAAGCTAGAGTCTTGTAGAGGGGGGTAG
AATTCCAGGTGTAGCGGTGAAATGCGTAGAGATCTGGAGGAATACCGGTGGCGAAGGC
GGCCCCCTGGACAAAGACTGACGCTCAGGTGCGAAAGCGTGGGGAGCAAACAGGATT
AGATACCCTGGTAGTCCACGCTGTAAACGATGTCGACTTGGAGGTTGTGCCCTTGAGGC
GTGGCTTCCGGAGCTAACGCGTTAAGTCGACCGCCTGGGGAGTACGGCCGCAAGGTTA
AAACTCAAATGAATTGACGGGGGCCCGCACAAGCGGTGGAGCATGTGGTTTAATTCGAT
GCAACGCGAAGAACCTTACCTACTCTTGACATCCACGGAATTTAGCAGAGATGCTTTAG
TGCCTTCGGGAACCGTGAGACAGGTGCTGCATGGCTGTCGTCAGCTCGTGTTGTGAAAT
GTTGGGTTAAGTCCCGCAACGAGCGCAACCCTTATCCTTTGTTGCCAGCACGTGATGGT
GGGAACTCAAAGGAGACTGCCGGTGATAAACCGGAGGAAGGTGGGGATGACGTCAAG
TCATCATGGCCCTTACGAGTAGGGCTACACACGTGCTACAATGGCAGATACAAAGTGAA
GCGAACTCGCGAGAGCAAGCGGACCACATAAAGTCTGTCGTAGTCCGGATTGGAGTCT
GCAACTCGACTCCATGAAGTCGGAATCGCTAGTAATCGTAGATCAGAATGCTACGGTGA
ATACGTTCCCGGGCCTTGTACACACCGCCCGTCACACCATGGGAGTGGGTTGCAAAAG
AAGTAGGTAGCTTAACCTTCGGGAGGGCGCTTACCACTTTGTGATTCATGACTGGGG
pattern 170
CGCTGGCGGCAGGCCTAACACATGCAAGTCGAGCGGCAGCGGGAAGTAGTTTACTACT
TTGCCGGCGAGCGGCGGACGGGTGAGTAATGTCTGGGAAACTGCCTGGTGGAGGGGGA
TAACTACTGGAAACGGTAGCTAATACCGCATGACCTCGAAAGAGCAAAGTGGGGGACC
TTCGGGCCTCACGCCATCGGATGTGCCCAGATGGGATTAGCTAGTAGGTGGGGTAATGG
CTCACCTAGGCGACGATCCCTAGCTGGTCTGAGAGGATGACCAGCCACACTGGAACTG
AGACACGGTCCAGACTCCTACGGGAGGCAGCAGTGGGGAATATTGCACAATGGGCGCA
AGCCTGATGCAGCCATGCCGCGTGTGTGAAGAAGGCCTTCGGGTTGTAAAGCACTTTCA
GCGAGGAGGAAGGCATAAAGGTTAATAACCTTTGTGATTGACGTTACTCGCAGAAGAA
GCACCGGCTAACTCCGTGCCAGCAGCCGCGGTAATACGGAGGGTGCAAGCGTTAATCG
GAATTACTGGGCGTAAAGCGCACGCAGGCGGTTTGTTAAGTCAGATGTGAAATCCCCGC
GCTTAACGTGGGAACTGCATTTGAAACTGGCAAGCTAGAGTCTTGTAGAGGGGGGTAG
AATTCCAGGTGTAGCGGTGAAATGCGTAGAGATCTGGAGGAATACCGGTGGCGAAGGC
GGCCCCCTGGACAAAGACTGACGCTCAGGTGCGAAAGCGTGGGGAGCAAACAGGATT
AGATACCCTGGTAGTCCACGCTGTAAACGATGTCGACTTGGAGGTTGTGCCCTTGAGGC
GTGGCTTCCGGAGCTAACGCGTTAAGTCGACCGCCTGGGGAGTACGGCCGCAAGGTTA
AAACTCAAATGAATTGACGGGGGCCCGCACAAGCGGTGGAGCATGTGGTTTAATTCGAT
GCAACGCGAAGAACCTTACCTACTCTTGACATCCACAGAACTTAGCAGAGATGCTTCGG
TGCCTTCGGGAACTGTGAGACAGGTGCTGCATGGCTGTCGTCAGCTCGTGTTGTGAAAT
GTTGGGTTAAGTCCCGCAACGAGCGCAACCCTTATCCTTTGTTGCCAGCGAGTAATGTC
GGGAACTCAAAGGAGACTGCCGGTGATAAACCGGAGGAAGGTGGGGATGACGTCAAG
TCATCATGGCCCTTACGAGTAGGGCTACACACGTGCTACAATGGCAGATACAAAGTGAA
GCGAACTCGCGAGAGCAAGCGGACCACATAAAGTCTGTCGTAGTCCGGATTGGAGTCT

GCAACTCGACTCCATGAAGTCGGAATCGCTAGTAATCGTAGATCAGAATGCTACGGTGA
ATACGTTCCCGGGCCTTGTACACACCGCCCGTCACACCATGGGAGTGGGTTGCAAAAG
AAGTAGGTAGCTTAACCTTCGGGAGGGCGCTTACCACTTTGTGATTCATGACTGGGG
pattern 171
CGCTGGCGGCAGGCCTAACACATGCAAGTCGAGCGGCAACGGGAAGTAGTTTACTACT
TTGCCGGCGAGCGGCGGACGGGTGAGTAATGTCTGGGAAACTGCCTGATGGAGGGGGA
TAACTACTGGAAACGGTAGCTAATACCGCATGACCTCGTAAGAGCAAAGTGGGGGACCT
TCGGGCCTCACGCCATCGGATGTGCCCAGATGGGATTAGCTAGTAGGTGGGGTAATGGC
TCACCTAGGCGACGATCCCTAGCTGGTCTGAGAGGATGACCAGCCACACTGGAACTGA
GACACGGTCCAGACTCCTACGGGAGGCAGCAGTGGGGAATATTGCACAATGGGCGCAA
GCCTGATGCAGCCATGCCGCGTGTGTGAAGAAGGCCTTCGGGTTGTAAAGCACTTTCAG
CGAGGAGGAAGGCAGTCGTGTTAATAGCACGGTTGATTGACGTTACTCGCAGAAGAAG
CACCGGCTAACTCCGTGCCAGCAGCCGCGGTAATACGGAGGGTGCAAGCGTTAATCGG
AATTACTGGGCGTAAAGCGCACGCAGGCGGTTTGTTAAGTCAGATGTGAAATCCCCGCG
CTTAACGTGGGAACTGCATTTGAAACTGGCAAGCTAGAGTCTTGTAGAGGGGGGTAGA
ATTCCAGGTGTAGCGGTGAAATGCGTAGAGATCTGGAGGAATACCGGTGGCGAAGGCG
GCCCCCTGGACAAAGACTGACGCTCAGGTGCGAAAGCGTGGGGAGCAAACAGGATTA
GATACCCTGGTAGTCCACGCTGTAAACGATGTCGACTTGGAGGTTGTGCCCTTGAGGCG
TGGCTTCCGGAGCTAACGCGTTAAGTCGACCGCCTGGGGAGTACGGCCGCAAGGTTAA
AACTCAAATGAATTGACGGGGGCCCGCACAAGCGGTGGAGCATGTGGTTTAATTCGATG
CAACGCGAAGAACCTTACCTACTCTTGACATCCACGGAATTTAGCAGAGATGCTTTAGT
GCCTTCGGGAACTGTGAGACAGGTGCTGCATGGCTGTCGTCAGCTCGTGTTGTGAAATG
TTGGGTTAAGTCCCGCAACGAGCGCAACCCTTATCCTTTGTTGCCAGCACGTAATGGTG
GGAACTCAAGGGAGACTGCCGGTGACAAACCGGAGGAAGGTGGGGATGACGTCAAGT
CATCATGGCCCTTACGAGTAGGGCTACACACGTGCTACAATGGCAGATACAAAGTGAAG
CGAACTCGCGAGAGCAAGCGGACCACATAAAGTCTGTCGTAGTCCGGATTGGAGTCTG
CAACTCGACTCCATGAAGTCGGAATCGCTAGTAATCGTAGATCAGAATGCTACGGTGAAT
ACGTTCCCGGGCCTTGTACACACCGCCCGTCACACCATGGGAGTGGGTTGCAAAAGAA
GTAGGTAGCTTAACCTTCGGGAGGGCGCTTACCACTTTGTGATTCATGACTGGGG
pattern 172
CGCTGGCGGCAGGCCTAACACATGCAAGTCGAGCGGCAGCGGAAAGTAGCTTGCTACT
TTGCCGGCGAGCGGCGGACGGGTGAGTAATGTCTGGGGATCTGCCTGATGGAGGGGGA
TAACTACTGGAAACGGTAGCTAATACCGCATGACCTCGAAAGAGCAAAGTGGGGGACC
TTAGGGCCTCACGCCATCGGATGAACCCAGATGGGATTAGCTAGTAGGTGGGGTAATGG
CTCACCTAGGCGACGATCCCTAGCTGGTCTGAGAGGATGACCAGCCACACTGGAACTG
AGACACGGTCCAGACTCCTACGGGAGGCAGCAGTGGGGAATATTGCACAATGGGCGCA
AGCCTGATGCAGCCATGCCGCGTGTGTGAAGAAGGCCTTCGGGTTGTAAAGCACTTTCA
GCGAGGAGGAAGGCATTGTGGTTAATAACCGCAGTGATTGACGTTACTCGCAGAAGAA
GCACCGGCTAACTCCGTGCCAGCAGCCGCGGTAATACGGAGGGTGCAAGCGTTAATCG
GAATTACTGGGCGTAAAGCGCACGCAGGCGGTTTGTTAAGTCAGATGTGAAATCCCCGC
GCTTAACGTGGGAACTGCATTTGAAACTGGCAAGCTAGAGTCTTGTAGAGGGGGGTAG
AATTCCAGGTGTAGCGGTGAAATGCGTAGAGATCTGGAGGAATACCGGTGGCGAAGGC
GGCCCCCTGGACAAAGACTGACGCTCAGGTGCGAAAGCGTGGGGAGCAAACAGGATT
AGATACCCTGGTAGTCCACGCTGTAAACGATGTCGACTTGGAGGTTGTGCCCTTGAGGC

GTGGCTTCCGGAGCTAACGCGTTAAGTCGACCGCCTGGGGAGTACGGCCGCAAGGTTA
AAACTCAAATGAATTGACGGGGGCCCGCACAAGCGGTGGAGCATGTGGTTTAATTCGAT
GCAACGCGAAGAACCTTACCTACTCTTGACATCCACAGAACTTAGCAGAGATGCTTCGG
TGCCTTCGGGAACTGTGAGACAGGTGCTGCATGGCTGTCGTCAGCTCGTGTTGTGAAAT
GTTGGGTTAAGTCCCGCAACGAGCGCAACCCTTATCCTTTGTTGCCAGCACGTAATGGT
GGGAACTCAAGGGAGACTGCCGGTGACAAACCGGAGGAAGGTGGGGATGACGTCAAG
TCATCATGGCCCTTACGAGTAGGGCTACACACGTGCTACAATGGCAGATACAAAGTGAA
GCGAACTCGCGAGAGCAAGCGGACCACATAAAGTCTGTCGTAGTCCGGATTGGAGTCT
GCAACTCGACTCCATGAAGTCGGAATCGCTAGTAATCGTAGATCAGAATGCTACGGTGA
ATACGTTCCCGGGCCTTGTACACACCGCCCGTCACACCATGGGAGTGGGTTGCAAAAG
AAGTAGGTAGCTTAACCTTCGGGAGGGCGCTTACCACTTTGTGATTCATGACTGGGG
pattern 173
CGCTGGCGGCAGGCCTAACACATGCAAGTCGAGCGGCAGCGGAAAGTAGCTTGCTACT
TTGCCGGCGAGCGGCGGACGGGTGAGTAATGTCTGGGGATCTGCCTGATGGAGGGGGA
TAACTACTGGAAACGGTAGCTAATACCGCATGACCTCGAAAGAGCAAAGTGGGGGACC
TTAGGGCCTCACGCCATCGGATGAACCCAGATGGGATTAGCTAGTAGGTGGGGTAATGG
CTCACCTAGGCGACGATCCCTAGCTGGTCTGAGAGGATGACCAGCCACACTGGAACTG
AGACACGGTCCAGACTCCTACGGGAGGCAGCAGTGGGGAATATTGCACAATGGGCGCA
AGCCTGATGCAGCCATGCCGCGTGTGTGAAGAAGGCCTTCGGGTTGTAAAGCACTTTCA
GCGAGGAGGAAGGCATTGTGGTTAATAACCACAGTGATTGACGTTACTCGCAGAAGAA
GCACCGGCTAACTCCGTGCCAGCAGCCGCGGTAATACGGAGGGTGCAAGCGTTAATCG
GAATTACTGGGCGTAAAGCGCACGCAGGCGGTTTGTTAAGTCAGATGTGAAATCCCCGC
GCTTAACGTGGGAACTGCATTTGAAACTGGCAAGCTAGAGTCTTGTAGAGGGGGGTAG
AATTCCAGGTGTAGCGGTGAAATGCGTAGAGATCTGGAGGAATACCGGTGGCGAAGGC
GGCCCCCTGGACAAAGACTGACGCTCAGGTGCGAAAGCGTGGGGAGCAAACAGGATT
AGATACCCTGGTAGTCCACGCTGTAAACGATGTCGACTTGGAGGTTGTGCCCTTGAGGC
GTGGCTTCCGGAGCTAACGCGTTAAGTCGACCGCCTGGGGAGTACGGCCGCAAGGTTA
AAACTCAAATGAATTGACGGGGGCCCGCACAAGCGGTGGAGCATGTGGTTTAATTCGAT
GCAACGCGAAGAACCTTACCTACTCTTGACATCCACAGAACTTAGCAGAGATGCTTCGG
TGCCTTCGGGAACTGTGAGACAGGTGCTGCATGGCTGTCGTCAGCTCGTGTTGTGAAAT
GTTGGGTTAAGTCCCGCAACGAGCGCAACCCTTATCCTTTGTTGCCAGCACGTAATGGT
GGGAACTCAAGGGAGACTGCCGGTGACAAACCGGAGGAAGGTGGGGATGACGTCAAG
TCATCATGGCCCTTACGAGTAGGGCTACACACGTGCTACAATGGCAGATACAAAGTGAA
GCGAACTCGCGAGAGCAAGCGGACCACATAAAGTCTGTCGTAGTCCGGATTGGAGTCT
GCAACTCGACTCCATGAAGTCGGAATCGCTAGTAATCGTAGATCAGAATGCTACGGTGA
ATACGTTCCCGGGCCTTGTACACACCGCCCGTCACACCATGGGAGTGGGTTGCAAAAG
AAGTAGGTAGCTTAACCTTCGGGAGGGCGCTTACCACTTTGTGATTCATGACTGGGG
pattern 174
CGCTGGCGGCAGGCCTAACACATGCAAGTCGAGCGGCAGCGGGAAGTAGTTTACTACT
TTGCCGGCGAGCGGCGGACGGGTGAGTAATGTCTGGGGAACTGCCTGATGGAGGGGGA
TAACTACTGGAAACGGTAGCTAATACCGCATAACGTCTTCGGACCAAAGTGGGGGACCT
TCGGGCCTCACGCCATCGGATGTGCCCAGATGGGATTAGCTAGTAGGTGGGGTAATGGC
TCACCTAGGCGACGATCCCTAGCTGGTCTGAGAGGATGACCAGCCACACTGGAACTGA
GACACGGTCCAGACTCCTACGGGAGGCAGCAGTGGGGAATATTGCACAATGGGCGCAA

GCCTGATGCAGCCATGCCGCGTGTGTGAAGAAGGCCTTCGGGTTGTAAAGCACTTTCAG
CGAGGAGGAAGGCATAAAGGTTAATAACCTTTGTGATTGACGTTACTCGCAGAAGAAG
CACCGGCTAACTCCGTGCCAGCAGCCGCGGTAATACGGAGGGTGCAAGCGTTAATCGG
AATTACTGGGCGTAAAGCGCACGCAGGCGGTTTGTTAAGTCAGATGTGAAATCCCCGCG
CTTAACGTGGGAACTGCATTTGAAACTGGCAAGCTAGAGTCTTGTAGAGGGGGGTAGA
ATTCCAGGTGTAGCGGTGAAATGCGTAGAGATCTGGAGGAATACCGGTGGCGAAGGCG
GCCCCCTGGACAAAGACTGACGCTCAGGTGCGAAAGCGTGGGGAGCAAACAGGATTA
GATACCCTGGTAGTCCACGCTGTAAACGATGTCGACTTGGAGGTTGTGCCCTTGAGGCG
TGGCTTCCGGAGCTAACGCGTTAAGTCGACCGCCTGGGGAGTACGGCCGCAAGGTTAA
AACTCAAATGAATTGACGGGGGCCCGCACAAGCGGTGGAGCATGTGGTTTAATTCGATG
CAACGCGAAGAACCTTACCTACTCTTGACATCCACAGAACTTAGCAGAGATGCTTCGGT
GCCTTCGGGAACTGTGAGACAGGTGCTGCATGGCTGTCGTCAGCTCGTGTTGTGAAATG
TTGGGTTAAGTCCCGCAACGAGCGCAACCCTTATCCTTTGTTGCCAGCACGTCATGGTG
GGAACTCAAAGGAGACTGCCGGTGATAAACCGGAGGAAGGTGGGGATGACGTCAAGT
CATCATGGCCCTTACGAGTAGGGCTACACACGTGCTACAATGGCAGATACAAAGTGAAG
CGAACTCGCGAGAGCAAGCGGACCACATAAAGTCTGTCGTAGTCCGGATTGGAGTCTG
CAACTCGACTCCATGAAGTCGGAATCGCTAGTAATCGTAGATCAGAATGCTACGGTGAAT
ACGTTCCCGGGCCTTGTACACACCGCCCGTCACACCATGGGAGTGGGTTGCAAAAGAA
GTAGGTAGCTTAACCTTCGGGAGGGCGCTTACCACTTTGTGATTCATGACTGGGG
pattern 175
CGCTGGCGGCAGGCCTAACACATGCAAGTCGAGCGGCAGCGGGAAGTAGTTTACTACT
TTGCCGGCGAGCGGCGGACGGGTGAGTAATGTCTGGGAAACTGCCTGATGGAGGGGGA
TAACTACTGGAAACGGTAGCTAATACCGCATAACGTCTTCGGACCAAAGTGGGGGACCT
TCGGGCCTCACGCCATCGGATGTGCCCAGATGGGATTAGCTAGTAGGTGGGGTAATGGC
TCACCTAGGCGACGATCCCTAGCTGGTCTGAGAGGATGACCAGCCACACTGGAACTGA
GACACGGTCCAGACTCCTACGGGAGGCAGCAGTGGGGAATATTGCACAATGGGCGCAA
GCCTGATGCAGCCATGCCGCGTGTGTGAAGAAGGCCTTCGGGTTGTAAAGCACTTTCAG
CGAGGAGGAAGGCATAAAGGTTAATAACCTTTGTGATTGACGTTACTCGCAGAAGAAG
CACCGGCTAACTCCGTGCCAGCAGCCGCGGTAATACGGAGGGTGCAAGCGTTAATCGG
AATTACTGGGCGTAAAGCGCACGCAGGCGGTTTGTTAAGTCAGATGTGAAATCCCCGCG
CTTAACGTGGGAACTGCATTTGAAACTGGCAAGCTAGAGTCTTGTAGAGGGGGGTAGA
ATTCCAGGTGTAGCGGTGAAATGCGTAGAGATCTGGAGGAATACCGGTGGCGAAGGCG
GCCCCCTGGACAAAGACTGACGCTCAGGTGCGAAAGCGTGGGGAGCAAACAGGATTA
GATACCCTGGTAGTCCACGCTGTAAACGATGTCGACTTGGAGGTTGTGCCCTTGAGGCG
TGGCTTCCGGAGCTAACGCGTTAAGTCGACCGCCTGGGGAGTACGGCCGCAAGGTTAA
AACTCAAATGAATTGACGGGGGCCCGCACAAGCGGTGGAGCATGTGGTTTAATTCGATG
CAACGCGAAGAACCTTACCTACTCTTGACATCCACAGAACTTAGCAGAGATGCTTCGGT
GCCTTCGGGAACTGTGAGACAGGTGCTGCATGGCTGTCGTCAGCTCGTGTTGTGAAATG
TTGGGTTAAGTCCCGCAACGAGCGCAACCCTTATCCTTTGTTGCCAGCGAGTAATGTCG
GGAACTCAAAGGAGACTGCCGGTGATAAACCGGAGGAAGGTGGGGATGACGTCAAGT
CATCATGGCCCTTACGAGTAGGGCTACACACGTGCTACAATGGCAGATACAAAGTGAAG
CGAACTCGCGAGAGCAAGCGGACCACATAAAGTCTGTCGTAGTCCGGATTGGAGTCTG
CAACTCGACTCCATGAAGTCGGAATCGCTAGTAATCGTAGATCAGAATGCTACGGTGAAT
ACGTTCCCGGGCCTTGTACACACCGCCCGTCACACCATGGGAGTGGGTTGCAAAAGAA

GTAGGTAGCTTAACCTTCGGGAGGGCGCTTACCACTTTGTGATTCATGACTGGGG
pattern 176
CGCTGGCGGCAGGCCTAACACATGCAAGTCGAGCGGCAGCGGGAAGTAGTTTACTACT
TTGCCGGCGAGCGGCGGACGGGTGAGTAATGTCTGGGAAACTGCCTGATGGAGGGGGA
TAACTACTGGAAACGGTAGCTAATACCGCATAACGTCTACGGACCAAAGTGGGGGACCT
TCGGGCCTCACGCCATCGGATGTGCCCAGATGGGATTAGCTAGTAGGTGGGGTAATGGC
TCACCTAGGCGACGATCCCTAGCTGGTCTGAGAGGATGACCAGCCACACTGGAACTGA
GACACGGTCCAGACTCCTACGGGAGGCAGCAGTGGGGAATATTGCACAATGGGCGCAA
GCCTGATGCAGCCATGCCGCGTGTGTGAAGAAGGCCTTCGGGTTGTAAAGCACTTTCAG
CGAGGAGGAAGGCAGTCGTGTTAATAGCACGATTGATTGACGTTACTCGCAGAAGAAG
CACCGGCTAACTCCGTGCCAGCAGCCGCGGTAATACGGAGGGTGCAAGCGTTAATCGG
AATTACTGGGCGTAAAGCGCACGCAGGCGGTTTGTTAAGTCAGATGTGAAATCCCCGCG
CTTAACGTGGGAACTGCATTTGAAACTGGCAAGCTAGAGTCTTGTAGAGGGGGGTAGA
ATTCCAGGTGTAGCGGTGAAATGCGTAGAGATCTGGAGGAATACCGGTGGCGAAGGCG
GCCCCCTGGACAAAGACTGACGCTCAGGTGCGAAAGCGTGGGGAGCAAACAGGATTA
GATACCCTGGTAGTCCACGCTGTAAACGATGTCGACTTGGAGGTTGTGCCCTTGAGGCG
TGGCTTCCGGAGCTAACGCGTTAAGTCGACCGCCTGGGGAGTACGGCCGCAAGGTTAA
AACTCAAATGAATTGACGGGGGCCCGCACAAGCGGTGGAGCATGTGGTTTAATTCGATG
CAACGCGAAGAACCTTACCTACTCTTGACATCCACGGAATTTAGCAGAGATGCTTTAGT
GCCTTCGGGAACCGTGAGACAGGTGCTGCATGGCTGTCGTCAGCTCGTGTTGTGAAATG
TTGGGTTAAGTCCCGCAACGAGCGCAACCCTTATCCTTTGTTGCCAGCACATAATGGTG
GGAACTCAAAGGAGACTGCCGGTGATAAACCGGAGGAAGGTGGGGATGACGTCAAGT
CATCATGGCCCTTACGAGTAGGGCTACACACGTGCTACAATGGCATATACAAAGAGAAG
CAAACTCGCGAGAGCCAGCGGACCTCATAAAGTATGTCGTAGTCCGGATTGGAGTCTGC
AACTCGACTCCATGAAGTCGGAATCGCTAGTAATCGTAGATCAGAATGCTACGGTGAAT
ACGTTCCCGGGCCTTGTACACACCGCCCGTCACACCATGGGAGTGGGTTGCAAAAGAA
GTAGGTAGCTTAACCTTCGGGAGGGCGCTTACCACTTTGTGATTCATGACTGGGG
pattern 177
CGCTGGCGGCAGGCCTAACACATGCAAGTCGAGCGGCAGCGGGAAGTAGTTTACTACT
TTGCCGGCGAGCGGCGGACGGGTGAGTAATGTCTGGGAAACTGCCTGATGGAGGGGGA
TAACTACTGGAAACGGTAGCTAATACCGCATAACGTCTTCGGACCAAAGTGGGGGACCT
TCGGGCCTCACGCCATCGGATGTGCCCAGATGGGATTAGCTAGTAGGTGGGGTAATGGC
TCACCTAGGCGACGATCCCTAGCTGGTCTGAGAGGATGACCAGCCACACTGGAACTGA
GACACGGTCCAGACTCCTACGGGAGGCAGCAGTGGGGAATATTGCACAATGGGCGCAA
GCCTGATGCAGCCATGCCGCGTGTGTGAAGAAGGCCTTCGGGTTGTAAAGCACTTTCAG
CGAGGAGGAAGGCATAAAGGTTAATAACCTTTGTGATTGACGTTACTCGCAGAAGAAG
CACCGGCTAACTCCGTGCCAGCAGCCGCGGTAATACGGAGGGTGCAAGCGTTAATCGG
AATTACTGGGCGTAAAGCGCACGCAGGCGGTTTGTTAAGTCAGATGTGAAATCCCCGCG
CTTAACGTGGGAACTGCATTTGAAACTGGCAAGCTAGAGTCTTGTAGAGGGGGGTAGA
ATTCCAGGTGTAGCGGTGAAATGCGTAGAGATCTGGAGGAATACCGGTGGCGAAGGCG
GCCCCCTGGACAAAGACTGACGCTCAGGTGCGAAAGCGTGGGGAGCAAACAGGATTA
GATACCCTGGTAGTCCACGCTGTAAACGATGTCGACTTGGAGGTTGTGCCCTTGAGGCG
TGGCTTCCGGAGCTAACGCGTTAAGTCGACCGCCTGGGGAGTACGGCCGCAAGGTTAA
AACTCAAATGAATTGACGGGGGCCCGCACAAGCGGTGGAGCATGTGGTTTAATTCGATG

CAACGCGAAGAACCTTACCTACTCTTGACATCCACGGAATTTAGCAGAGATGCTTTAGT
GCCTTCGGGAACCGTGAGACAGGTGCTGCATGGCTGTCGTCAGCTCGTGTTGTGAAATG
TTGGGTTAAGTCCCGCAACGAGCGCAACCCTTATCCTTTGTTGCCAGCACATAATGGTG
GGAACTCAAAGGAGACTGCCGGTGATAAACCGGAGGAAGGTGGGGATGACGTCAAGT
CATCATGGCCCTTACGAGTAGGGCTACACACGTGCTACAATGGCATATACAAAGAGAAG
CAAACTCGCGAGAGCTAGCGGACCTCATAAAGTATGTCGTAGTCCGGATTGGAGTCTGC
AACTCGACTCCATGAAGTCGGAATCGCTAGTAATCGTAGATCAGAATGCTACGGTGAAT
ACGTTCCCGGGCCTTGTACACACCGCCCGTCACACCATGGGAGTGGGTTGCAAAAGAA
GTAGGTAGCTTAACCTTCGGGAGGGCGCTTACCACTTTGTGATTCATGACTGGGG

pattern 178

CGCTGGCGGCAGGCCTAACACATGCAAGTCGAGCGGCAGCGGGAAGTAGTTTACTACT
TTGCCGGCGAGCGGCGGACGGGTGAGTAATGTCTGGGAAACTGCCTGATGGAGGGGGA
TAACTACTGGAAACGGTAGCTAATACCGCATAACGTCTTCGGACCAAAGTGGGGGACCT
TCGGGCCTCACGCCATTGGATGTGCCCAGATGGGATTAGCTAGTAGGTGGGGTAATGGC
TCACCTAGGCGACGATCCCTAGCTGGTCTGAGAGGATGACCAGCCACACTGGAACTGA
GACACGGTCCAGACTCCTACGGGAGGCAGCAGTGGGGAATATTGCACAATGGGCGCAA
GCCTGATGCAGCCATGCCGCGTGTGTGAAGAAGGCCTTCGGGTTGTAAAGCACTTTCAG
CGAGGAGGAAGGCAGTCGTGTTAATAGCACGATTGATTGACGTTACTCGCAGAAGAAG
CACCGGCTAACTCCGTGCCAGCAGCCGCGGTAATACGGAGGGTGCAAGCGTTAATCGG
AATTACTGGGCGTAAAGCGCACGCAGGCGGTTTGTTAAGTCAGATGTGAAATCCCCGCG
CTTAACGTGGGAACTGCATTTGAAACTGGCAAGCTAGAGTCTTGTAGAGGGGGGTAGA
ATTCCAGGTGTAGCGGTGAAATGCGTAGAGATCTGGAGGAATACCGGTGGCGAAGGCG
GCCCCCTGGACAAAGACTGACGCTCAGGTGCGAAAGCGTGGGGAGCAAACAGGATTA
GATACCCTGGTAGTCCACGCTGTAAACGATGTCGACTTGGAGGTTGTGCCCTTGAGGCG
TGGCTTCCGGAGCTAACGCGTTAAGTCGACCGCCTGGGGAGTACGGCCGCAAGGTTAA
AACTCAAATGAATTGACGGGGGCCCGCACAAGCGGTGGAGCATGTGGTTTAATTCGATG
CAACGCGAAGAACCTTACCTACTCTTGACATCCACGGAATTTAGCAGAGATGCTTTAGT
GCCTTCGGGAACCGTGAGACAGGTGCTGCATGGCTGTCGTCAGCTCGTGTTGTGAAATG
TTGGGTTAAGTCCCGCAACGAGCGCAACCCTTATCCTTTGTTGCCAGCACATAATGGTG
GGAACTCAAAGGAGACTGCCGGTGATAAACCGGAGGAAGGTGGGGATGACGTCAAGT
CATCATGGCCCTTACGAGTAGGGCTACACACGTGCTACAATGGCATATACAAAGAGAAG
CAAACTCGCGAGAGCCAGCGGACCTCATAAAGTATGTCGTAGTCCGGATTGGAGTCTGC
AACTCGACTCCATGAAGTCGGAATCGCTAGTAATCGTAGATCAGAATGCTACGGTGAAT
ACGTTCCCGGGCCTTGTACACACCGCCCGTCACACCATGGGAGTGGGTTGCAAAAGAA
GTAGGTAGCTTAACCTTCGGGAGGGCGCTTACCACTTTGTGATTCATGACTGGGG

pattern 179

CGCTGGCGGCAGGCCTAACACATGCAAGTCGAGCGGCAGCGGGAAGTAGTTTACTACT
TTGCCGGCGAGCGGCGGACGGGTGAGTAATGTCTGGGAAACTGCCTGATGGAGGGGGA
TAACTACTGGAAACGGTAGCTAATACCGCATAACGTCTTCGGACCAAAGTGGGGGACCT
TCGGGCCTCACGCCATCGGATGTGCCCAGATGGGATTAGCTAGTAGGTGGGGTAATGGC
TCACCTAGGCGACGATCCCTAGCTGGTCTGAGAGGATGACCAGCCACACTGGAACTGA
GACACGGTCCAGACTCCTACGGGAGGCAGCAGTGGGGAATATTGCACAATGGGCGCAA
GCCTGATGCAGCCATGCCGCGTGTGTGAAGAAGGCCTTCGGGTTGTAAAGCACTTTCAG
CGAGGAGGAAGGCAGTCGTGTTAATAGCACGATTGATTGACGTTACTCGCAGAAGAAG

CACCGGCTAACTCCGTGCCAGCAGCCGCGGTAATACGGAGGGTGCAAGCGTTAATCGG
AATTACTGGGCGTAAAGCGCACGCAGGCGGTTTGTTAAGTCAGATGTGAAATCCCCGCG
CTTAACGTGGGAACTGCATTTGAAACTGGCAAGCTAGAGTCTTGTAGAGGGGGGTAGA
ATTCCAGGTGTAGCGGTGAAATGCGTAGAGATCTGGAGGAATACCGGTGGCGAAGGCG
GCCCCCTGGACAAAGACTGACGCTCAGGTGCGAAAGCGTGGGGAGCAAACAGGATTA
GATACCCTGGTAGTCCACGCTGTAAACGATGTCGACTTGGAGGTTGTGCCCTTGAGGCG
TGGCTTCCGGAGCTAACGCGTTAAGTCGACCGCCTGGGGAGTACGGCCGCAAGGTTAA
AACTCAAATGAATTGACGGGGGCCCGCACAAGCGGTGGAGCATGTGGTTTAATTCGATG
CAACGCGAAGAACCTTACCTACTCTTGACATCCACGGAATTTAGCAGAGATGCTTTAGT
GCCTTCGGGAACCGTGAGACAGGTGCTGCATGGCTGTCGTCAGCTCGTGTTGTGAAATG
TTGGGTTAAGTCCCGCAACGAGCGCAACCCTTATCCTTTGTTGCCAGCACATAATGGTG
GGAACTCAAAGGAGACTGCCGGTGATAAACCGGAGGAAGGTGGGGATGACGTCAAGT
CATCATGGCCCTTACGAGTAGGGCTACACACGTGCTACAATGGCATATACAAAGAGAAG
CAAACTCGCGAGAGCTAGCGGACCTCATAAAGTATGTCGTAGTCCGGATTGGAGTCTGC
AACTCGACTCCATGAAGTCGGAATCGCTAGTAATCGTAGATCAGAATGCTACGGTGAAT
ACGTTCCCGGGCCTTGTACACACCGCCCGTCACACCATGGGAGTGGGTTGCAAAAGAA
GTAGGTAGCTTAACCTTCGGGAGGGCGCTTACCACTTTGTGATTCATGACTGGGG
pattern 180
CGCTGGCGGCAGGCCTAACACATGCAAGTCGAGCGGCAGCGGGAAGTAGTTTACTACT
TTGCCGGCGAGCGGCGGACGGGTGAGTAATGTCTGGGAAACTGCCTGATGGAGGGGGA
TAACTACTGGAAACGGTAGCTAATACCGCATAACGTCTTCGGACCAAAGTGGGGGACCT
TCGGGCCTCACGCCATCGGATGTGCCCAGATGGGATTAGCTAGTAGGTGGGGTAATGGC
TCACCTAGGCGACGATCCCTAGCTGGTCTGAGAGGATGACCAGCCACACTGGAACTGA
GACACGGTCCAGACTCCTACGGGAGGCAGCAGTGGGGAATATTGCACAATGGGCGCAA
GCCTGATGCAGCCATGCCGCGTGTGTGAAGAAGGCCTTCGGGTTGTAAAGCACTTTCAG
CGAGGAGGAAGGCAGTCGTGTTAATAGCACGATTGATTGACGTTACTCGCAGAAGAAG
CACCGGCTAACTCCGTGCCAGCAGCCGCGGTAATACGGAGGGTGCAAGCGTTAATCGG
AATTACTGGGCGTAAAGCGCACGCAGGCGGTTTGTTAAGTCAGATGTGAAATCCCCGCG
CTTAACGTGGGAACTGCATTTGAAACTGGCAAGCTAGAGTCTTGTAGAGGGGGGTAGA
ATTCCAGGTGTAGCGGTGAAATGCGTAGAGATCTGGAGGAATACCGGTGGCGAAGGCG
GCCCCCTGGACAAAGACTGACGCTCAGGTGCGAAAGCGTGGGGAGCAAACAGGATTA
GATACCCTGGTAGTCCACGCTGTAAACGATGTCGACTTGGAGGTTGTGCCCTTGAGGCG
TGGCTTCCGGAGCTAACGCGTTAAGTCGACCGCCTGGGGAGTACGGCCGCAAGGTTAA
AACTCAAATGAATTGACGGGGGCCCGCACAAGCGGTGGAGCATGTGGTTTAATTCGATG
CAACGCGAAGAACCTTACCTACTCTTGACATCCACGGAATTTAGCAGAGATGCTTTAGT
GCCTTCGGGAACCGTGAGACAGGTGCTGCATGGCTGTCGTCAGCTCGTGTTGTGAAATG
TTGGGTTAAGTCCCGCAACGAGCGCAACCCTTATCCTTTGTTGCCAGCACATAATGGTG
GGAACTCAAAGGAGACTGCCGGTGATAAACCGGAGGAAGGTGGGGATGACGTCAAGT
CATCATGGCCCTTACGAGTAGGGCTACACACGTGCTACAATGGCATATACAAAGAGAAG
CAAACTCGCGAGAGCCAGCGGACCTCATAAAGTATGTCGTAGTCCGGATTGGAGTCTGC
AACTCGACTCCATGAAGTCGGAATCGCTAGTAATCGTAGATCAGAATGCTACGGTGAAT
ACGTTCCCGGGCCTTGTACACACCGCCCGTCACACCATGGGAGTGGGTTGCAAAAGAA
GTAGGTAGCTTAACCTTCGGGAGGGCGCTTACCACTTTGTGATTCATGACTGGGG
pattern 181

CGCTGGCGGCAGGCCTAACACATGCAAGTCGAGCGGCAGCGGGAAGTAGTTTACTACT
TTGCCGGCGAGCGGCGGACGGGTGAGTAATGTCTGGGAAACTGCCTGATGGAGGGGGA
TAACTACTGGAAACGGTAGCTAATACCGCATGACCTCGAAAGAGCAAAGTGGGGGACC
TTCGGGCCTCACGCCATCGGATGTGCCCAGATGGGATTAGCTAGTAGGTGGGGTAATGG
CTCACCTAGGCGACGATCCCTAGCTGGTCTGAGAGGATGACCAGCCACACTGGAACTG
AGACACGGTCCAGACTCCTACGGGAGGCAGCAGTGGGGAATATTGCACAATGGGCGCA
AGCCTGATGCAGCCATGCCGCGTGTGTGAAGAAGGCCTTCGGGTTGTAAAGCACTTTCA
GCGAGGAGGAAGGCAATCGTGTTAATAGCACGATTGATTGACGTTACTCGCAGAAGAA
GCACCGGCTAACTCCGTGCCAGCAGCCGCGGTAATACGGAGGGTGCAAGCGTTAATCG
GAATTACTGGGCGTAAAGCGCACGCAGGCGGTTTGTTAAGTCAGATGTGAAATCCCCGC
GCTTAACGTGGGAACTGCATTTGAAACTGGCAAGCTAGAGTCTTGTAGAGGGGGGTAG
AATTCCAGGTGTAGCGGTGAAATGCGTAGAGATCTGGAGGAATACCGGTGGCGAAGGC
GGCCCCCTGGACAAAGACTGACGCTCAGGTGCGAAAGCGTGGGGAGCAAACAGGATT
AGATACCCTGGTAGTCCACGCTGTAAACGATGTCGACTTGGAGGTTGTGCCCTTGAGGC
GTGGCTTCCGGAGCTAACGCGTTAAGTCGACCGCCTGGGGAGTACGGCCGCAAGGTTA
AAACTCAAATGAATTGACGGGGGCCCGCACAAGCGGTGGAGCATGTGGTTTAATTCGAT
GCAACGCGAAGAACCTTACCTACTCTTGACATCCACAGAACTTAGCAGAGATGCTTCGG
TGCCTTCGGGAACTGTGAGACAGGTGCTGCATGGCTGTCGTCAGCTCGTGTTGTGAAAT
GTTGGGTTAAGTCCCGCAACGAGCGCAACCCTTATCCTTTGTTGCCAGCGAGTAATGTC
GGGAACTCAAAGGAGACTGCCGGTGATAAACCGGAGGAAGGTGGGGATGACGTCAAG
TCATCATGGCCCTTACGAGTAGGGCTACACACGTGCTACAATGGCAGATACAAAGTGAA
GCGAACTCGCGAGAGCAAGCGGACCACATAAAGTCTGTCGTAGTCCGGATTGGAGTCT
GCAACTCGACTCCATGAAGTCGGAATCGCTAGTAATCGTAGATCAGAATGCTACGGTGA
ATACGTTCCCGGGCCTTGTACACACCGCCCGTCACACCATGGGAGTGGGTTGCAAAAG
AAGTAGGTAGCTTAACCTTCGGGAGGGCGCTTACCACTTTGTGATTCATGACTGGGG
pattern 182
CGCTGGCGGCAGGCCTAACACATGCAAGTCGAGCGGCAGCGGAAAGTAGCTTGCTACT
TTGCCGGCGAGCGGCGGACGGGTGAGTAATGTCTGGGAAACTGCCTGATGGAGGGGGA
TAACTACTGGAAACGGTAGCTAATACCGCATGACCTCGCAAGAGCAAAGTGGGGGACC
TTCGGGCCTCACGCCATCGGATGTGCCCAGATGGGATTAGCTAGTAGGTGGGGTAATGG
CTCACCTAGGCGACGATCCCTAGCTGGTCTGAGAGGATGACCAGCCACACTGGAACTG
AGACACGGTCCAGACTCCTACGGGAGGCAGCAGTGGGGAATATTGCACAATGGGCGCA
AGCCTGATGCAGCCATGCCGCGTGTGTGAAGAAGGCCTTCGGGTTGTAAAGCACTTTCA
GCGAGGAGGAAGGCAGTCGTGTTAATAGCACGGTTGATTGACGTTACTCGCAGAAGAA
GCACCGGCTAACTCCGTGCCAGCAGCCGCGGTAATACGGAGGGTGCAAGCGTTAATCG
GAATTACTGGGCGTAAAGCGCACGCAGGCGGTTTGTTAAGTCAGATGTGAAATCCCCGC
GCTTAACGTGGGAACTGCATTTGAAACTGGCAAGCTAGAGTCTTGTAGAGGGGGGTAG
AATTCCAGGTGTAGCGGTGAAATGCGTAGAGATCTGGAGGAATACCGGTGGCGAAGGC
GGCCCCCTGGACAAAGACTGACGCTCAGGTGCGAAAGCGTGGGGAGCAAACAGGATT
AGATACCCTGGTAGTCCACGCTGTAAACGATGTCGACTTGGAGGTTGTGCCCTTGAGGC
GTGGCTTCCGGAGCTAACGCGTTAAGTCGACCGCCTGGGGAGTACGGCCGCAAGGTTA
AAACTCAAATGAATTGACGGGGGCCCGCACAAGCGGTGGAGCATGTGGTTTAATTCGAT
GCAACGCGAAGAACCTTACCTACTCTTGACATCCACAGAACTTAGCAGAGATGCTTCGG
TGCCTTCGGGAACTGTGAGACAGGTGCTGCATGGCTGTCGTCAGCTCGTGTTGTGAAAT

GTTGGGTTAAGTCCCGCAACGAGCGCAACCCTTATCCTTTGTTGCCAGCACGTAATGGT
GGGAACTCAAGGGAGACTGCCGGTGACAAACCGGAGGAAGGTGGGGATGACGTCAAG
TCATCATGGCCCTTACGAGTAGGGCTACACACGTGCTACAATGGCAGATACAAAGTGAA
GCGAGCTCGCGAGAGTCAGCGGACCACATAAAGTCTGTCGTAGTCCGGATTGGAGTCT
GCAACTCGACTCCATGAAGTCGGAATCGCTAGTAATCGTAGATCAGAATGCTACGGTGA
ATACGTTCCCGGGCCTTGTACACACCGCCCGTCACACCATGGGAGTGGGTTGCAAAAG
AAGTAGGTAGCTTAACCTTCGGGAGGGCGCTTACCACTTTGTGATTCATGACTGGGG
pattern 183
CGCTGGCGGCAGGCCTAACACATGCAAGTCGAGCGGCAGCGGGAAGTAGCTTGCTACT
TTGCCGGCGAGCGGCGGACGGGTGAGTAATGTCTGGGAAACTGCCTGATGGAGGGGGA
TAACTACTGGAAACGGTAGCTAATACCGCATAACGTCTTCGGACCAAAGTGGGGGACCT
TCGGGCCTCACGCCATCGGATGTGCCCAGATGGGATTAGCTAGTAGGTGGGGTAATGGC
TCACCTAGGCGACGATCCCTAGCTGGTCTGAGAGGATGACCAGCCACACTGGAACTGA
GACACGGTCCAGACTCCTACGGGAGGCAGCAGTGGGGAATATTGCACAATGGGCGCAA
GCCTGATGCAGCCATGCCGCGTGTGTGAAGAAGGCCTTCGGGTTGTAAAGCACTTTCAG
CGAGGAGGAAGGCATAAAGGTTAATAACCTTTGTGATTGACGTTACTCGCAGAAGAAG
CACCGGCTAACTCCGTGCCAGCAGCCGCGGTAATACGGAGGGTGCAAGCGTTAATCGG
AATTACTGGGCGTAAAGCGCACGCAGGCGGTTTGTTAAGTCAGATGTGAAATCCCCGCG
CTTAACGTGGGAACTGCATTTGAAACTGGCAAGCTAGAGTCTTGTAGAGGGGGGTAGA
ATTCCAGGTGTAGCGGTGAAATGCGTAGAGATCTGGAGGAATACCGGTGGCGAAGGCG
GCCCCCTGGACAAAGACTGACGCTCAGGTGCGAAAGCGTGGGGAGCAAACAGGATTA
GATACCCTGGTAGTCCACGCTGTAAACGATGTCGACTTGGAGGTTGTGCCCTTGAGGCG
TGGCTTCCGGAGCTAACGCGTTAAGTCGACCGCCTGGGGAGTACGGCCGCAAGGTTAA
AACTCAAATGAATTGACGGGGGCCCGCACAAGCGGTGGAGCATGTGGTTTAATTCGATG
CAACGCGAAGAACCTTACCTACTCTTGACATCCACAGAACTTAGCAGAGATGCTTCGGT
GCCTTCGGGAACTGTGAGACAGGTGCTGCATGGCTGTCGTCAGCTCGTGTTGTGAAATG
TTGGGTTAAGTCCCGCAACGAGCGCAACCCTTATCCTTTGTTGCCAGCGAGTAATGTCG
GGAACTCAAAGGAGACTGCCGGTGATAAACCGGAGGAAGGTGGGGATGACGTCAAGT
CATCATGGCCCTTACGAGTAGGGCTACACACGTGCTACAATGGCAGATACAAAGTGAAG
CGAACTCGCGAGAGCAAGCGGACCACATAAAGTCTGTCGTAGTCCGGATTGGAGTCTG
CAACTCGACTCCATGAAGTCGGAATCGCTAGTAATCGTAGATCAGAATGCTACGGTGAAT
ACGTTCCCGGGCCTTGTACACACCGCCCGTCACACCATGGGAGTGGGTTGCAAAAGAA
GTAGGTAGCTTAACCTTCGGGAGGGCGCTTACCACTTTGTGATTCATGACTGGGG
pattern 184
CGCTGGCGGCAGGCCTAACACATGCAAGTCGAGCGGCAGCGGAAAGTAGCTTGCTACT
TTGCCGGCGAGCGGCGGACGGGTGAGTAATGTCTGGGAAACTGCCTGATGGAGGGGGA
TAACTACTGGAAACGGTAGCTAATACCGCATGACCTCGCAAGAGCAAAGTGGGGGACC
TTCGGGCCTCACGCCATCGGATGTGCCCAGATGGGATTAGCTAGTAGGTGGGGTAATGG
CTCACCTAGGCGACGATCCCTAGCTGGTCTGAGAGGATGACCAGCCACACTGGAACTG
AGACACGGTCCAGACTCCTACGGGAGGCAGCAGTGGGGAATATTGCACAATGGGCGCA
AGCCTGATGCAGCCATGCCGCGTGTGTGAAGAAGGCCTTCGGGTTGTAAAGCACTTTCA
GCGAGGAGGAAGGCAGTCGTGTTAATAGCACGATTGATTGACGTTACTCGCAGAAGAA
GCACCGGCTAACTCCGTGCCAGCAGCCGCGGTAATACGGAGGGTGCAAGCGTTAATCG
GAATTACTGGGCGTAAAGCGCACGCAGGCGGTTTGTTAAGTCAGATGTGAAATCCCCGC

GCTTAACGTGGGAACTGCATTTGAAACTGGCAAGCTAGAGTCTTGTAGAGGGGGGTAG
AATTCCAGGTGTAGCGGTGAAATGCGTAGAGATCTGGAGGAATACCGGTGGCGAAGGC
GGCCCCCTGGACAAAGACTGACGCTCAGGTGCGAAAGCGTGGGGAGCAAACAGGATT
AGATACCCTGGTAGTCCACGCTGTAAACGATGTCGACTTGGAGGTTGTGCCCTTGAGGC
GTGGCTTCCGGAGCTAACGCGTTAAGTCGACCGCCTGGGGAGTACGGCCGCAAGGTTA
AAACTCAAATGAATTGACGGGGGCCCGCACAAGCGGTGGAGCATGTGGTTTAATTCGAT
GCAACGCGAAGAACCTTACCTACTCTTGACATCCACAGAACTTAGCAGAGATGCTTCGG
TGCCTTCGGGAACTGTGAGACAGGTGCTGCATGGCTGTCGTCAGCTCGTGTTGTGAAAT
GTTGGGTTAAGTCCCGCAACGAGCGCAACCCTTATCCTTTGTTGCCAGCACGTAATGGT
GGGAACTCAAGGGAGACTGCCGGTGACAAACCGGAGGAAGGTGGGGATGACGTCAAG
TCATCATGGCCCTTACGAGTAGGGCTACACACGTGCTACAATGGCAGATACAAAGTGAA
GCGAACTCGCGAGAGCCAGCGGACCACATAAAGTCTGTCGTAGTCCGGATTGGAGTCT
GCAACTCGACTCCATGAAGTCGGAATCGCTAGTAATCGTAGATCAGAATGCTACGGTGA
ATACGTTCCCGGGCCTTGTACACACCGCCCGTCACACCATGGGAGTGGGTTGCAAAAG
AAGTAGGTAGCTTAACCTTCGGGAGGGCGCTTACCACTTTGTGATTCATGACTGGGG
pattern 185
CGCTGGCGGCAGGCCTAACACATGCAAGTCGAGCGGCAGCGGAAAGTAGCTTGCTACT
TTGCCGGCGAGCGGCGGACGGGTGAGTAATGTCTGGGAAACTGCCTGATGGAGGGGGA
TAACTACTGGAAACGGTAGCTAATACCGCATGACCTCGCAAGAGCAAAGTGGGGGACC
TTCGGGCCTCACGCCATCGGATGTGCCCAGATGGGATTAGCTAGTAGGTGAGGTAATGG
CTCACCTAGGCGACGATCCCTAGCTGGTCTGAGAGGATGACCAGCCACACTGGAACTG
AGACACGGTCCAGACTCCTACGGGAGGCAGCAGTGGGGAATATTGCACAATGGGCGCA
AGCCTGATGCAGCCATGCCGCGTGTGTGAAGAAGGCCTTCGGGTTGTAAAGCACTTTCA
GCGAGGAGGAAGGCAGTCGTGTTAATAGCACGGTTGATTGACGTTACTCGCAGAAGAA
GCACCGGCTAACTCCGTGCCAGCAGCCGCGGTAATACGGAGGGTGCAAGCGTTAATCG
GAATTACTGGGCGTAAAGCGCACGCAGGCGGTTTGTTAAGTCAGATGTGAAATCCCCGC
GCTTAACGTGGGAACTGCATTTGAAACTGGCAAGCTAGAGTCTTGTAGAGGGGGGTAG
AATTCCAGGTGTAGCGGTGAAATGCGTAGAGATCTGGAGGAATACCGGTGGCGAAGGC
GGCCCCCTGGACAAAGACTGACGCTCAGGTGCGAAAGCGTGGGGAGCAAACAGGATT
AGATACCCTGGTAGTCCACGCTGTAAACGATGTCGACTTGGAGGTTGTGCCCTTGAGGC
GTGGCTTCCGGAGCTAACGCGTTAAGTCGACCGCCTGGGGAGTACGGCCGCAAGGTTA
AAACTCAAATGAATTGACGGGGGCCCGCACAAGCGGTGGAGCATGTGGTTTAATTCGAT
GCAACGCGAAGAACCTTACCTACTCTTGACATCCACAGAACTTAGCAGAGATGCTTCGG
TGCCTTCGGGAACTGTGAGACAGGTGCTGCATGGCTGTCGTCAGCTCGTGTTGTGAAAT
GTTGGGTTAAGTCCCGCAACGAGCGCAACCCTTATCCTTTGTTGCCAGCACGTAATGGT
GGGAACTCAAGGGAGACTGCCGGTGACAAACCGGAGGAAGGTGGGGATGACGTCAAG
TCATCATGGCCCTTACGAGTAGGGCTACACACGTGCTACAATGGCAGATACAAAGTGAA
GCGAACTCGCGAGAGTCAGCGGACCACATAAAGTCTGTCGTAGTCCGGATTGGAGTCT
GCAACTCGACTCCATGAAGTCGGAATCGCTAGTAATCGTAGATCAGAATGCTACGGTGA
ATACGTTCCCGGGCCTTGTACACACCGCCCGTCACACCATGGGAGTGGGTTGCAAAAG
AAGTAGGTAGCTTAACCTTCGGGAGGGCGCTTACCACTTTGTGATTCATGACTGGGG
pattern 186
CGCTGGCGGCAGGCCTAACACATGCAAGTCGAGCGGCAGCGGAAAGTAGCTTGCTACT
TTGCCGGCGAGCGGCGGACGGGTGAGTAATGTCTGGGAAACTGCCTGATGGAGGGGGA

TAACTACTGGAAACGGTAGCTAATACCGCATGACCTCGCAAGAGCAAAGTGGGGGACC
TTCGGGCCTCACGCCATCGGATGTGCCCAGATGGGATTAGCTAGTAGGTGGGGTAATGG
CTCACCTAGGCGACGATCCCTAGCTGGTCTGAGAGGATGACCAGCCACACTGGAACTG
AGACACGGTCCAGACTCCTACGGGAGGCAGCAGTGGGGAATATTGCACAATGGGCGCA
AGCCTGATGCAGCCATGCCGCGTGTGTGAAGAAGGCCTTCGGGTTGTAAAGCACTTTCA
GCGAGGAGGAAGGCAGTCGTGTTAATAGCACGGTTGATTGACGTTACTCGCAGAAGAA
GCACCGGCTAACTCCGTGCCAGCAGCCGCGGTAATACGGAGGGTGCAAGCGTTAATCG
GAATTACTGGGCGTAAAGCGCACGCAGGCGGTTTGTTAAGTCAGATGTGAAATCCCCGC
GCTTAACGTGGGAACTGCATTTGAAACTGGCAAGCTAGAGTCTTGTAGAGGGGGGTAG
AATTCCAGGTGTAGCGGTGAAATGCGTAGAGATCTGGAGGAATACCGGTGGCGAAGGC
GGCCCCCTGGACAAAGACTGACGCTCAGGTGCGAAAGCGTGGGGAGCAAACAGGATT
AGATACCCTGGTAGTCCACGCTGTAAACGATGTCGACTTGGAGGTTGTGCCCTTGAGGC
GTGGCTTCCGGAGCTAACGCGTTAAGTCGACCGCCTGGGGAGTACGGCCGCAAGGTTA
AAACTCAAATGAATTGACGGGGGCCCGCACAAGCGGTGGAGCATGTGGTTTAATTCGAT
GCAACGCGAAGAACCTTACCTACTCTTGACATCCACAGAACTTAGCAGAGATGCTTCGG
TGCCTTCGGGAACTGTGAGACAGGTGCTGCATGGCTGTCGTCAGCTCGTGTTGTGAAAT
GTTGGGTTAAGTCCCGCAACGAGCGCAACCCTTATCCTTTGTTGCCAGCACGTAATGGT
GGGAACTCAAGGGAGACTGCCGGTGACAAACCGGAGGAAGGTGGGGATGACGTCAAG
TCATCATGGCCCTTACGAGTAGGGCTACACACGTGCTACAATGGCAGATACAAAGTGAA
GCGAACTCGCGAGAGCCAGCGGACCACATAAAGTCTGTCGTAGTCCGGATTGGAGTCT
GCAACTCGACTCCATGAAGTCGGAATCGCTAGTAATCGTAGATCAGAATGCTACGGTGA
ATACGTTCCCGGGCCTTGTACACACCGCCCGTCACACCATGGGAGTGGGTTGCAAAAG
AAGTAGGTAGCTTAACCTTCGGGAGGGCGCTTACCACTTTGTGATTCATGACTGGGG
pattern 187
CGCTGGCGGCAGGCCTAACACATGCAAGTCGAGCGGCAGCGGGAAGTAGCTTGCTACT
TTGCCGGCGAGCGGCGGACGGGTGAGTAATGTCTGGGAAACTGCCTGATGGAGGGGGA
TAACTACTGGAAACGGTAGCTAATACCGCATAACGTCTACGGACCAAAGTGGGGGACCT
TCGGGCCTCACGCCATCGGATGTGCCCAGATGGGATTAGCTAGTAGGTGGGGTAATGGC
TCACCTAGGCGACGATCCCTAGCTGGTCTGAGAGGATGACCAGCCACACTGGAACTGA
GACACGGTCCAGACTCCTACGGGAGGCAGCAGTGGGGAATATTGCACAATGGGCGCAA
GCCTGATGCAGCCATGCCGCGTGTGTGAAGAAGGCCTTCGGGTTGTAAAGCACTTTCAG
CGAGGAGGAAGGCATAAAGGTTAATAACCTTTGTGATTGACGTTACTCGCAGAAGAAG
CACCGGCTAACTCCGTGCCAGCAGCCGCGGTAATACGGAGGGTGCAAGCGTTAATCGG
AATTACTGGGCGTAAAGCGCACGCAGGCGGTTTGTTAAGTCAGATGTGAAATCCCCGCG
CTTAACGTGGGAACTGCATTTGAAACTGGCAAGCTAGAGTCTTGTAGAGGGGGGTAGA
ATTCCAGGTGTAGCGGTGAAATGCGTAGAGATCTGGAGGAATACCGGTGGCGAAGGCG
GCCCCCTGGACAAAGACTGACGCTCAGGTGCGAAAGCGTGGGGAGCAAACAGGATTA
GATACCCTGGTAGTCCACGCTGTAAACGATGTCGACTTGGAGGTTGTGCCCTTGAGGCG
TGGCTTCCGGAGCTAACGCGTTAAGTCGACCGCCTGGGGAGTACGGCCGCAAGGTTAA
AACTCAAATGAATTGACGGGGGCCCGCACAAGCGGTGGAGCATGTGGTTTAATTCGATG
CAACGCGAAGAACCTTACCTACTCTTGACATCCACGGAATTTAGCAGAGATGCTTTAGT
GCCTTCGGGAACTGTGAGACAGGTGCTGCATGGCTGTCGTCAGCTCGTGTTGTGAAATG
TTGGGTTAAGTCCCGCAACGAGCGCAACCCTTATCCTTTGTTGCCAGCGAGTAATGTCG
GGAACTCAAAGGAGACTGCCGGTGATAAACCGGAGGAAGGTGGGGATGACGTCAAGT

CATCATGGCCCTTACGAGTAGGGCTACACACGTGCTACAATGGCAGATACAAAGTGAAG
CGAACTCGCGAGAGCAAGCGGACCACATAAAGTCTGTCGTAGTCCGGATTGGAGTCTG
CAACTCGACTCCATGAAGTCGGAATCGCTAGTAATCGTAGATCAGAATGCTACGGTGAAT
ACGTTCCCGGGCCTTGTACACACCGCCCGTCACACCATGGGAGTGGGTTGCAAAAGAA
GTAGGTAGCTTAACCTTCGGGAGGGCGCTTACCACTTTGTGATTCATGACTGGGG
pattern 188
CGCTGGCGGCAGGCCTAACACATGCAAGTCGAGCGGCAGCGGGAAGTAGCTTGCTACT
TTGCCGGCGAGCGGCGGACGGGTGAGTAATGTCTGGGAAACTGCCTGATGGAGGGGGA
TAACTACTGGAAACGGTAGCTAATACCGCATAACGTCTACGGACCAAAGTGGGGGACCT
TCGGGCCTCACGCCATCGGATGTGCCCAGATGGGATTAGCTAGTAGGTGGGGTAATGGC
TCACCTAGGCGACGATCCCTAGCTGGTCTGAGAGGATGACCAGCCACACTGGAACTGA
GACACGGTCCAGACTCCTACGGGAGGCAGCAGTGGGGAATATTGCACAATGGGCGCAA
GCCTGATGCAGCCATGCCGCGTGTGTGAAGAAGGCCTTCGGGTTGTAAAGCACTTTCAG
CGAGGAGGAAGGCATAAAGGTTAATAACCTTTGTGATTGACGTTACTCGCAGAAGAAG
CACCGGCTAACTCCGTGCCAGCAGCCGCGGTAATACGGAGGGTGCAAGCGTTAATCGG
AATTACTGGGCGTAAAGCGCACGCAGGCGGTTTGTTAAGTCAGATGTGAAATCCCCGCG
CTTAACGTGGGAACTGCATTTGAAACTGGCAAGCTAGAGTCTTGTAGAGGGGGGTAGA
ATTCCAGGTGTAGCGGTGAAATGCGTAGAGATCTGGAGGAATACCGGTGGCGAAGGCG
GCCCCCTGGACAAAGACTGACGCTCAGGTGCGAAAGCGTGGGGAGCAAACAGGATTA
GATACCCTGGTAGTCCACGCTGTAAACGATGTCGACTTGGAGGTTGTGCCCTTGAGGCG
TGGCTTCCGGAGCTAACGCGTTAAGTCGACCGCCTGGGGAGTACGGCCGCAAGGTTAA
AACTCAAATGAATTGACGGGGGCCCGCACAAGCGGTGGAGCATGTGGTTTAATTCGATG
CAACGCGAAGAACCTTACCTACTCTTGACATCCACAGAACTTAGCAGAGATGCTTCGGT
GCCTTCGGGAACTGTGAGACAGGTGCTGCATGGCTGTCGTCAGCTCGTGTTGTGAAATG
TTGGGTTAAGTCCCGCAACGAGCGCAACCCTTATCCTTTGTTGCCAGCGAGTAATGTCG
GGAACTCAAAGGAGACTGCCGGTGATAAACCGGAGGAAGGTGGGGATGACGTCAAGT
CATCATGGCCCTTACGAGTAGGGCTACACACGTGCTACAATGGCAGATACAAAGTGAAG
CGAACTCGCGAGAGCAAGCGGACCACATAAAGTCTGTCGTAGTCCGGATTGGAGTCTG
CAACTCGACTCCATGAAGTCGGAATCGCTAGTAATCGTAGATCAGAATGCTACGGTGAAT
ACGTTCCCGGGCCTTGTACACACCGCCCGTCACACCATGGGAGTGGGTTGCAAAAGAA
GTAGGTAGCTTAACCTTCGGGAGGGCGCTTACCACTTTGTGATTCATGACTGGGG
pattern 189
CGCTGGCGGCAGGCCTAACACATGCAAGTCGAGCGGCAGCGGAAAGTAGCTTGCTACT
TTGCCGGCGAGCGGCGGACGGGTGAGTAATGTCTGGGGATCTGCCTGATGGAGGGGGA
TAACTACTGGAAACGGTAGCTAATACCGCATGACCTCGAAAGAGCAAAGTGGGGGACC
TTCGGGCCTCACGCCATCGGATGAACCCAGATGGGATTAGCTAGTAGGTGAGGTAATGG
CTCACCTAGGCGACGATCCCTAGCTGGTCTGAGAGGATGACCAGCCACACTGGAACTG
AGACACGGTCCAGACTCCTACGGGAGGCAGCAGTGGGGAATATTGCACAATGGGCGCA
AGCCTGATGCAGCCATGCCGCGTGTGTGAAGAAGGCCTTCGGGTTGTAAAGCACTTTCA
GCGAGGAGGAAGGCATTGTGGTTAATAACCACAGTGATTGACGTTACTCGCAGAAGAA
GCACCGGCTAACTCCGTGCCAGCAGCCGCGGTAATACGGAGGGTGCAAGCGTTAATCG
GAATTACTGGGCGTAAAGCGCACGCAGGCGGTTTGTTAAGTCAGATGTGAAATCCCCGC
GCTTAACGTGGGAACTGCATTTGAAACTGGCAAGCTAGAGTCTTGTAGAGGGGGGTAG
AATTCCAGGTGTAGCGGTGAAATGCGTAGAGATCTGGAGGAATACCGGTGGCGAAGGC

GGCCCCCTGGACAAAGACTGACGCTCAGGTGCGAAAGCGTGGGGAGCAAACAGGATT
AGATACCCTGGTAGTCCACGCTGTAAACGATGTCGACTTGGAGGTTGTGCCCTTGAGGT
GTGGCTTCCGGAGCTAACGCGTTAAGTCGACCGCCTGGGGAGTACGGCCGCAAGGTTA
AAACTCAAATGAATTGACGGGGGCCCGCACAAGCGGTGGAGCATGTGGTTTAATTCGAT
GCAACGCGAAGAACCTTACCTACTCTTGACATCCACGGAATTTAGCAGAGATGCTTTAG
TGCCTTCGGGAACCGTGAGACAGGTGCTGCATGGCTGTCGTCAGCTCGTGTTGTGAAAT
GTTGGGTTAAGTCCCGCAACGAGCGCAACCCTTATCCTTTGTTGCCAGCACGTAATGGT
GGGAACTCAAGGGAGACTGCCGGTGACAAACCGGAGGAAGGTGGGGATGACGTCAAG
TCATCATGGCCCTTACGAGTAGGGCTACACACGTGCTACAATGGCAGATACAAAGTGAA
GCGAACTCGCGAGAGCAAGCGGACCACATAAAGTCTGTCGTAGTCCGGATTGGAGTCT
GCAACTCGACTCCATGAAGTCGGAATCGCTAGTAATCGTAGATCAGAATGCTACGGTGA
ATACGTTCCCGGGCCTTGTACACACCGCCCGTCACACCATGGGAGTGGGTTGCAAAAG
AAGTAGGTAGCTTAACCTTCGGGAGGGCGCTTACCACTTTGTGATTCATGACTGGGG
pattern 190
CGCTGGCGGCAGGCCTAACACATGCAAGTCGAGCGGCAGCGGAAAGTAGCTTGCTACT
TTGCCGGCGAGCGGCGGACGGGTGAGTAATGTCTGGGGATCTGCCTGATGGAGGGGGA
TAACTACTGGAAACGGTAGCTAATACCGCATGACCTCGAAAGAGCAAAGTGGGGGACC
TTCGGGCCTCACGCCATCGGATGAACCCAGATGGGATTAGCTAGTAGGTGGGGTAATGG
CTCACCTAGGCGACGATCCTTAGCTGGTCTGAGAGGATGACCAGCCACACTGGAACTG
AGACACGGTCCAGACTCCTACGGGAGGCAGCAGTGGGGAATATTGCACAATGGGCGCA
AGCCTGATGCAGCCATGCCGCGTGTGTGAAGAAGGCCTTCGGGTTGTAAAGCACTTTCA
GCGAGGAGGAAGGCATTGTGGTTAATAACCGCAGTGATTGACGTTACTCGCAGAAGAA
GCACCGGCTAACTCCGTGCCAGCAGCCGCGGTAATACGGAGGGTGCAAGCGTTAATCG
GAATTACTGGGCGTAAAGCGCACGCAGGCGGTTTGTTAAGTCAGATGTGAAATCCCCGC
GCTTAACGTGGGAACTGCATTTGAAACTGGCAAGCTAGAGTCTTGTAGAGGGGGGTAG
AATTCCAGGTGTAGCGGTGAAATGCGTAGAGATCTGGAGGAATACCGGTGGCGAAGGC
GGCCCCCTGGACAAAGACTGACGCTCAGGTGCGAAAGCGTGGGGAGCAAACAGGATT
AGATACCCTGGTAGTCCACGCTGTAAACGATGTCGACTTGGAGGTTGTGCCCTTGAGGC
GTGGCTTCCGGAGCTAACGCGTTAAGTCGACCGCCTGGGGAGTACGGCCGCAAGGTTA
AAACTCAAATGAATTGACGGGGGCCCGCACAAGCGGTGGAGCATGTGGTTTAATTCGAT
GCAACGCGAAGAACCTTACCTACTCTTGACATCCACAGAACTTAGCAGAGATGCTTCGG
TGCCTTCGGGAACTGTGAGACAGGTGCTGCATGGCTGTCGTCAGCTCGTGTTGTGAAAT
GTTGGGTTAAGTCCCGCAACGAGCGCAACCCTTATCCTTTGTTGCCAGCACGTAATGGT
GGGAACTCAAGGGAGACTGCCGGTGACAAACCGGAGGAAGGTGGGGATGACGTCAAG
TCATCATGGCCCTTACGAGTAGGGCTACACACGTGCTACAATGGCAGATACAAAGTGAA
GCGAACTCGCGAGAGCAAGCGGACCACATAAAGTCTGTCGTAGTCCGGATTGGAGTCT
GCAACTCGACTCCATGAAGTCGGAATCGCTAGTAATCGTAGATCAGAATGCTACGGTGA
ATACGTTCCCGGGCCTTGTACACACCGCCCGTCACACCATGGGAGTGGGTTGCAAAAG
AAGTAGGTAGCTTAACCTTCGGGAGGGCGCTTACCACTTTGTGATTCATGACTGGGG
pattern 191
CGCTGGCGGCAGGCCTAACACATGCAAGTCGAGCGGCAGCGGGAAGTAGTTTACTACT
TTGCCGGCGAGCGGCGGACGGGTGAGTAATGTCTGGGAAACTGCCTGATGGAGGGGGA
TAACTACTGGAAACGGTAGCTAATACCGCATGACCTCGTAAGAGCAAAGTGGGGGACCT
TCGGGCCTCACGCCATCGGATGTGCCCAGATGGGATTAGCTAGTAGGTGGGGTAATGGC

TCACCTAGGCGACGATCCCTAGCTGGTCTGAGAGGATGACCAGCCACACTGGAACTGA
GACACGGTCCAGACTCCTACGGGAGGCAGCAGTGGGGAATATTGCACAATGGGCGCAA
GCCTGATGCAGCCATGCCGCGTGTGTGAAGAAGGCCTTCGGGTTGTAAAGCACTTTCAG
CGAGGAGGAAGGCAGTCGTGTTAATAACACGGTTGATTGACGTTACTCGCAGAAGAAG
CACCGGCTAACTCCGTGCCAGCAGCCGCGGTAATACGGAGGGTGCAAGCGTTAATCGG
AATTACTGGGCGTAAAGCGCACGCAGGCGGTTTGTTAAGTCAGATGTGAAATCCCCGCG
CTTAACGTGGGAACTGCATTTGAAACTGGCAAGCTAGAGTCTTGTAGAGGGGGGTAGA
ATTCCAGGTGTAGCGGTGAAATGCGTAGAGATCTGGAGGAATACCGGTGGCGAAGGCG
GCCCCCTGGACAAAGACTGACGCTCAGGTGCGAAAGCGTGGGGAGCAAACAGGATTA
GATACCCTGGTAGTCCACGCTGTAAACGATGTCGACTTGGAGGTTGTGCCCTTGAGGCG
TGGCTTCCGGAGCTAACGCGTTAAGTCGACCGCCTGGGGAGTACGGCCGCAAGGTTAA
AACTCAAATGAATTGACGGGGGCCCGCACAAGCGGTGGAGCATGTGGTTTAATTCGATG
CAACGCGAAGAACCTTACCTACTCTTGACATCCACAGAACTTAGCAGAGATGCTTCGGT
GCCTTCGGGAACTGTGAGACAGGTGCTGCATGGCTGTCGTCAGCTCGTGTTGTGAAATG
TTGGGTTAAGTCCCGCAACGAGCGCAACCCTTATCCTTTGTTGCCAGCACGTAATGGTG
GGAACTCAAGGGAGACTGCCGGTGACAAACCGGAGGAAGGTGGGGATGACGTCAAGT
CATCATGGCCCTTACGAGTAGGGCTACACACGTGCTACAATGGCAGATACAAAGTGAAG
CGAACTCGCGAGAGCAAGCGGACCACATAAAGTCTGTCGTAGTCCGGATTGGAGTCTG
CAACTCGACTCCATGAAGTCGGAATCGCTAGTAATCGTAGATCAGAATGCTACGGTGAAT
ACGTTCCCGGGCCTTGTACACACCGCCCGTCACACCATGGGAGTGGGTTGCAAAAGAA
GTAGGTAGCTTAACCTTCGGGAGGGCGCTTACCACTTTGTGATTCATGACTGGGG
pattern 192
CGCTGGCGGCAGGCCTAACACATGCAAGTCGAGCGGCAGCGGGAAGTAGTTTACTACT
TTGCCGGCGAGCGGCGGACGGGTGAGTAATGTCTGGGAAACTGCCTGATGGAGGGGGA
TAACTACTGGAAACGGTAGCTAATACCGCATAACGTCTACGGACCAAAGTGGGGGACCT
TCGGGCCTCACGCCATCGGATGTGCCCAGATGGGATTAGCTAGTAGGTGGGGTAATGGC
TCACCTAGGCGACGATCCCTAGCTGGTCTGAGAGGATGACCAGCCACACTGGAACTGA
GACACGGTCCAGACTCCTACGGGAGGCAGCAGTGGGGAATATTGCACAATGGGCGCAA
GCCTGATGCAGCCATGCCGCGTGTGTGAAGAAGGCCTTCGGGTTGTAAAGCACTTTCAG
CGAGGAGGAAGGCATGAAGGTTAATAACCTTTGTGATTGACGTTACTCGCAGAAGAAG
CACCGGCTAACTCCGTGCCAGCAGCCGCGGTAATACGGAGGGTGCAAGCGTTAATCGG
AATTACTGGGCGTAAAGCGCACGCAGGCGGTTTGTTAAGTCAGATGTGAAATCCCCGCG
CTTAACGTGGGAACTGCATTTGAAACTGGCAAGCTAGAGTCTTGTAGAGGGGGGTAGA
ATTCCAGGTGTAGCGGTGAAATGCGTAGAGATCTGGAGGAATACCGGTGGCGAAGGCG
GCCCCCTGGACAAAGACTGACGCTCAGGTGCGAAAGCGTGGGGAGCAAACAGGATTA
GATACCCTGGTAGTCCACGCTGTAAACGATGTCGACTTGGAGGTTGTGCCCTTGAGGCG
TGGCTTCCGGAGCTAACGCGTTAAGTCGACCGCCTGGGGAGTACGGCCGCAAGGTTAA
AACTCAAATGAATTGACGGGGGCCCGCACAAGCGGTGGAGCATGTGGTTTAATTCGATG
CAACGCGAAGAACCTTACCTACTCTTGACATCCACAGAACTTAGCAGAGATGCTTCGGT
GCCTTCGGGAACTGTGAGACAGGTGCTGCATGGCTGTCGTCAGCTCGTGTTGTGAAATG
TTGGGTTAAGTCCCGCAACGAGCGCAACCCTTATCCTTTGTTGCCAGCGAGTAATGTCG
GGAACTCAAAGGAGACTGCCGGTGATAAACCGGAGGAAGGTGGGGATGACGTCAAGT
CATCATGGCCCTTACGAGTAGGGCTACACACGTGCTACAATGGCAGATACAAAGTGAAG
CGAACTCGCGAGAGCAAGCGGACCACATAAAGTCTGTCGTAGTCCGGATTGGAGTCTG

CAACTCGACTCCATGAAGTCGGAATCGCTAGTAATCGTAGATCAGAATGCTACGGTGAAT
ACGTTCCCGGGCCTTGTACACACCGCCCGTCACACCATGGGAGTGGGTTGCAAAAGAA
GTAGGTAGCTTAACCTTCGGGAGGGCGCTTACCACTTTGTGATTCATGACTGGGG
pattern 193
CGCTGGCGGCAGGCCTAACACATGCAAGTCGAGCGGCAGCGGGAAGTAGCTTGCTACT
TTGCCGGCGAGCGGCGGACGGGTGAGTAATGTCTGGGAAACTGCCTGATGGAGGGGGA
TAACTACTGGAAACGGTAGCTAATACCGCATAACGTCTACGGACCAAAGTGGGGGACCT
TCGGGCCTCACGCCATCGGATGTGCCCAGATGGGATTAGCTAGTAGGTGGGGTAATGGC
TCACCTAGGCGACGATCCCTAGCTGGTCTGAGAGGATGACCAGCCACACTGGAACTGA
GACACGGTCCAGACTCCTACGGGAGGCAGCAGTGGGGAATATTGCACAATGGGCGCAA
GCCTGATGCAGCCATGCCGCGTGTGTGAAGAAGGCCTTCGGGTTGTAAAGCACTTTCAG
CGAGGAGGAAGGCATGAAGGTTAATAACCTTTGTGATTGACGTTACTCGCAGAAGAAG
CACCGGCTAACTCCGTGCCAGCAGCCGCGGTAATACGGAGGGTGCAAGCGTTAATCGG
AATTACTGGGCGTAAAGCGCACGCAGGCGGTTTGTTAAGTCAGATGTGAAATCCCCGCG
CTTAACGTGGGAACTGCATTTGAAACTGGCAAGCTAGAGTCTTGTAGAGGGGGGTAGA
ATTCCAGGTGTAGCGGTGAAATGCGTAGAGATCTGGAGGAATACCGGTGGCGAAGGCG
GCCCCCTGGACAAAGACTGACGCTCAGGTGCGAAAGCGTGGGGAGCAAACAGGATTA
GATACCCTGGTAGTCCACGCTGTAAACGATGTCGACTTGGAGGTTGTGCCCTTGAGGCG
TGGCTTCCGGAGCTAACGCGTTAAGTCGACCGCCTGGGGAGTACGGCCGCAAGGTTAA
AACTCAAATGAATTGACGGGGGCCCGCACAAGCGGTGGAGCATGTGGTTTAATTCGATG
CAACGCGAAGAACCTTACCTACTCTTGACATCCACAGAACTTAGCAGAGATGCTTCGGT
GCCTTCGGGAACTGTGAGACAGGTGCTGCATGGCTGTCGTCAGCTCGTGTTGTGAAATG
TTGGGTTAAGTCCCGCAACGAGCGCAACCCTTATCCTTTGTTGCCAGCGAGTAATGTCG
GGAACTCAAAGGAGACTGCCGGTGATAAACCGGAGGAAGGTGGGGATGACGTCAAGT
CATCATGGCCCTTACGAGTAGGGCTACACACGTGCTACAATGGCAGATACAAAGTGAAG
CGAACTCGCGAGAGCAAGCGGACCACATAAAGTCTGTCGTAGTCCGGATTGGAGTCTG
CAACTCGACTCCATGAAGTCGGAATCGCTAGTAATCGTAGATCAGAATGCTACGGTGAAT
ACGTTCCCGGGCCTTGTACACACCGCCCGTCACACCATGGGAGTGGGTTGCAAAAGAA
GTAGGTAGCTTAACCTTCGGGAGGGCGCTTACCACTTTGTGATTCATGACTGGGG
pattern 194
CGCTGGCGGCAGGCCTAACACATGCAAGTCGAGCGGCAGCGGAAAGTAGCTTGCTACT
TTGCCGGCGAGCGGCGGACGGGTGAGTAATGTCTGGGGATCTGCCTGATGGAGGGGGA
TAACTACTGGAAACGGTAGCTAATACCGCATGACCTCGAAAGAGCAAAGTGGGGGACC
TTCGGGCCTCACGCCATCGGATGAACCCAGATGGGATTAGCTAGTAGGTGGGGTAATGG
CTCACCTAGGCGACGATCCCTAGCTGGCCTGAGAGGATGACCAGCCACACTGGAACTG
AGACACGGTCCAGACTCCTACGGGAGGCAGCAGTGGGGAATATTGCACAATGGGCGCA
AGCCTGATGCAGCCATGCCGCGTGTGTGAAGAAGGCCTTCGGGTTGTAAAGCACTTTCA
GCGAGGAGGAAGGCATTGTGGTTAATAACCACAGTGATTGACGTTACTCGCAGAAGAA
GCACCGGCTAACTCCGTGCCAGCAGCCGCGGTAATACGGAGGGTGCAAGCGTTAATCG
GAATTACTGGGCGTAAAGCGCACGCAGGCGGTTTGTTAAGTCAGATGTGAAATCCCCGC
GCTTAACGTGGGAACTGCATTTGAAACTGGCAAGCTAGAGTCTTGTAGAGGGGGGTAG
AATTCCAGGTGTAGCGGTGAAATGCGTAGAGATCTGGAGGAATACCGGTGGCGAAGGC
GGCCCCCTGGACAAAGACTGACGCTCAGGTGCGAAAGCGTGGGGAGCAAACAGGATT
AGATACCCTGGTAGTCCACGCTGTAAACGATGTCGACTTGGAGGTTGTGCCCTTGAGGC

GTGGCTTCCGGAGCTAACGCGTTAAGTCGACCGCCTGGGGAGTACGGCCGCAAGGTTA
AAACTCAAATGAATTGACGGGGGCCCGCACAAGCGGTGGAGCATGTGGTTTAATTCGAT
GCAACGCGAAGAACCTTACCTACTCTTGACATCCACAGAACTTAGCAGAGATGCTTCGG
TGCCTTCGGGAACTGTGAGACAGGTGCTGCATGGCTGTCGTCAGCTCGTGTTGTGAAAT
GTTGGGTTAAGTCCCGCAACGAGCGCAACCCTTATCCTTTGTTGCCAGCACGTAATGGT
GGGAACTCAAGGGAGACTGCCGGTGACAAACCGGAGGAAGGTGGGGATGACGTCAAG
TCATCATGGCCCTTACGAGTAGGGCTACACACGTGCTACAATGGCAGATACAAAGTGAA
GCGAACTCGCGAGAGCAAGCGGACCACATAAAGTCTGTCGTAGTCCGGATTGGAGTCT
GCAACTCGACTCCATGAAGTCGGAATCGCTAGTAATCGTAGATCAGAATGCTACGGTGA
ATACGTTCCCGGGCCTTGTACACACCGCCCGTCACACCATGGGAGTGGGTTGCAAAAG
AAGTAGGTAGCTTAACCTTCGGGAGGGCGCTTACCACTTTGTGATTCATGACTGGGG
pattern 195
CGCTGGCGGCAGGCCTAACACATGCAAGTCGAGCGGCAGCGGGAAGTAGTTTACTACT
TTGTCGGCGAGCGGCGGACGGGTGAGTAATGTCTGGGGATCTGCCTGATGGAGGGGGA
TAACTACTGGAAACGGTAGCTAATACCGCGTGACCTCGCAAGAGCAAAGTGGGGGACC
TTAGGGCCTCACGCCATCGGATGAACCCAGATGGGATTAGCTAGTAGGTGGGGTAATGG
CTCACCTAGGCGACGATCCCTAGCTGGTCTGAGAGGATGACCAGCCACACTGGAACTG
AGACACGGTCCAGACTCCTACGGGAGGCAGCAGTGGGGAATATTGCACAATGGGCGCA
AGCCTGATGCAGCCATGCCGCGTGTGTGAAGAAGGCCTTCGGGTTGTAAAGCACTTTCA
GCGAGGAGGAAGGGGTTGAGTTTAATACGCTCAATCATTGACGTTACTCGCAGAAGAA
GCACCGGCTAACTCCGTGCCAGCAGCCGCGGTAATACGGAGGGTGCAAGCGTTAATCG
GAATTACTGGGCGTAAAGCGCACGCAGGCGGTTTGTTAAGTCAGATGTGAAATCCCCGC
GCTTAACGTGGGAACTGCATTTGAAACTGGCAAGCTAGAGTCTTGTAGAGGGGGGTAG
AATTCCAGGTGTAGCGGTGAAATGCGTAGAGATCTGGAGGAATACCGGTGGCGAAGGC
GGCCCCCTGGACAAAGACTGACGCTCAGGTGCGAAAGCGTGGGGAGCAAACAGGATT
AGATACCCTGGTAGTCCACGCTGTAAACGATGTCGACTTGGAGGTTGTGCCCTTGAGGC
GTGGCTTCCGGAGCTAACGCGTTAAGTCGACCGCCTGGGGAGTACGGCCGCAAGGTTA
AAACTCAAATGAATTGACGGGGGCCCGCACAAGCGGTGGAGCATGTGGTTTAATTCGAT
GCAACGCGAAGAACCTTACCTACTCTTGACATCCACAGAATTTGGCAGAGATGCTAAAG
TGCCTTCGGGAACTGTGAGACAGGTGCTGCATGGCTGTCGTCAGCTCGTGTTGTGAAAT
GTTGGGTTAAGTCCCGCAACGAGCGCAACCCTTATCCTTTGTTGCCAGCACGTAATGGT
GGGAACTCAAGGGAGACTGCCGGTGACAAACCGGAGGAAGGTGGGGATGACGTCAAG
TCATCATGGCCCTTACGAGTAGGGCTACACACGTGCTACAATGGCAGATACAAAGTGAA
GCGAACTCGCGAGAGCCAGCGGACCACATAAAGTCTGTCGTAGTCCGGATTGGAGTCT
GCAACTCGACTCCATGAAGTCGGAATCGCTAGTAATCGTAGATCAGAATGCTACGGTGA
ATACGTTCCCGGGCCTTGTACACACCGCCCGTCACACCATGGGAGTGGGTTGCAAAAG
AAGTAGGTAGCTTAACCTTCGGGAGGGCGCTTACCACTTTGTGATTCATGACTGGGG
pattern 196
CGCTGGCGGCAGGCCTAACACATGCAAGTCGAGCGGCAGCGGGAAGTAGTTTACTACT
TTGCCGGCGAGCGGCGGACGGGTGAGTAATGTCTGGGGATCTGCCTGATGGAGGGGGA
TAACTACTGGAAACGGTAGCTAATACCGCATGACCTCGCAAGAGCAAAGTGGGGGACC
TTAGGGCCTCACGCCATCGGATGAACCCAGATGGGATTAGCTAGTAGGTGGGGTAATGG
CTCACCTAGGCGACGATCCCTAGCTGGTCTGAGAGGATGACCAGCCACACTGGAACTG
AGACACGGTCCAGACTCCTACGGGAGGCAGCAGTGGGGAATATTGCACAATGGGCGCA

AGCCTGATGCAGCCATGCCGCGTGTGTGAAGAAGGCCTTCGGGTTGTAAAGCACTTTCA
GCGAGGAGGAAGGGGTTGAGTTTAATACGCTCAATCATTGACGTTACTCGCAGAAGAA
GCACCGGCTAACTCCGTGCCAGCAGCCGCGGTAATACGGAGGGTGCAAGCGTTAATCG
GAATTACTGGGCGTAAAGCGCACGCAGGCGGTTTGTTAAGTCAGATGTGAAATCCCCGC
GCTTAACGTGGGAACTGCATTTGAAACTGGCAAGCTAGAGTCTTGTAGAGGGGGGTAG
AATTCCAGGTGTAGCGGTGAAATGCGTAGAGATCTGGAGGAATACCGGTGGCGAAGGC
GGCCCCCTGGACAAAGACTGACGCTCAGGTGCGAAAGCGTGGGGAGCAAACAGGATT
AGATACCCTGGTAGTCCACGCTGTAAACGATGTCGACTTGGAGGTTGTGCCCTTGAGGC
GTGGCTTCCGGAGCTAACGCGTTAAGTCGACCGCCTGGGGAGTACGGCCGCAAGGTTA
AAACTCAAATGAATTGACGGGGGCCCGCACAAGCGGTGGAGCATGTGGTTTAATTCGAT
GCAACGCGAAGAACCTTACCTACTCTTGACATCCACGGAATTTAGCAGAGATGCTTTAG
TGCCTTCGGGAACCGTGAGACAGGTGCTGCATGGCTGTCGTCAGCTCGTGTTGTGAAAT
GTTGGGTTAAGTCCCGCAACGAGCGCAACCCTTATCCTTTGTTGCCAGCACGTAATGGT
GGGAACTCAAGGGAGACTGCCGGTGACAAACCGGAGGAAGGTGGGGATGACGTCAAG
TCATCATGGCCCTTACGAGTAGGGCTACACACGTGCTACAATGGCAGATACAAAGTGAA
GCGAACTCGCGAGAGCCAGCGGACCACATAAAGTCTGTCGTAGTCCGGATTGGAGTCT
GCAACTCGACTCCATGAAGTCGGAATCGCTAGTAATCGTAGATCAGAATGCTACGGTGA
ATACGTTCCCGGGCCTTGTACACACCGCCCGTCACACCATGGGAGTGGGTTGCAAAAG
AAGTAGGTAGCTTAACCTTCGGGAGGGCGCTTACCACTTTGTGATTCATGACTGGGG
pattern 197
CGCTGGCGGCAGGCCTAACACATGCAAGTCGAGCGGCAGCGGGAAGTAGTTTACTACT
TTGCCGGCGAGCGGCGGACGGGTGAGTAATGTCTGGGGATCTGCCTGATGGAGGGGGA
TAACTACTGGAAACGGTGGCTAATACCGCATGACCTCGCAAGAGCAAAGTGGGGGACC
TTAGGGCCTCACGCCATCGGATGAACCCAGATGGGATTAGCTAGTAGGTGGGGTAATGG
CTCACCTAGGCGACGATCCCTAGCTGGTCTGAGAGGATGACCAGCCACACTGGAACTG
AGACACGGTCCAGACTCCTACGGGAGGCAGCAGTGGGGAATATTGCACAATGGGCGCA
AGCCTGATGCAGCCATGCCGCGTGTGTGAAGAAGGCCTTCGGGTTGTAAAGCACTTTCA
GCGAGGAGGAAGGGGTTGAGTTTAATACGCTCAATCATTGACGTTACTCGCAGAAGAA
GCACCGGCTAACTCCGTGCCAGCAGCCGCGGTAATACGGAGGGTGCAAGCGTTAATCG
GAATTACTGGGCGTAAAGCGCACGCAGGCGGTTTGTTAAGTCAGATGTGAAATCCCCGC
GCTTAACGTGGGAACTGCATTTGAAACTGGCAAGCTAGAGTCTTGTAGAGGGGGGTAG
AATTCCAGGTGTAGCGGTGAAATGCGTAGAGATCTGGAGGAATACCGGTGGCGAAGGC
GGCCCCCTGGACAAAGACTGACGCTCAGGTGCGAAAGCGTGGGGAGCAAACAGGATT
AGATACCCTGGTAGTCCACGCTGTAAACGATGTCGACTTGGAGGTTGTGCCCTTGAGGC
GTGGCTTCCGGAGCTAACGCGTTAAGTCGACCGCCTGGGGAGTACGGCCGCAAGGTTA
AAACTCAAATGAATTGACGGGGGCCCGCACAAGCGGTGGAGCATGTGGTTTAATTCGAT
GCAACGCGAAGAACCTTACCTACTCTTGACATCCACAGAATTTGGCAGAGATGCTAAAG
TGCCTTCGGGAACTGTGAGACAGGTGCTGCATGGCTGTCGTCAGCTCGTGTTGTGAAAT
GTTGGGTTAAGTCCCGCAACGAGCGCAACCCTTATCCTTTGTTGCCAGCACGTAATGGT
GGGAACTCAAGGGAGACTGCCGGTGACAAACCGGAGGAAGGTGGGGATGACGTCAAG
TCATCATGGCCCTTACGAGTAGGGCTACACACGTGCTACAATGGCAGATACAAAGTGAA
GCGAACTCGCGAGAGTCAGCGGACCACATAAAGTCTGTCGTAGTCCGGATTGGAGTCT
GCAACTCGACTCCATGAAGTCGGAATCGCTAGTAATCGTAGATCAGAATGCTACGGTGA
ATACGTTCCCGGGCCTTGTACACACCGCCCGTCACACCATGGGAGTGGGTTGCAAAAG

AAGTAGGTAGCTTAACCTTCGGGAGGGCGCTTACCACTTTGTGATTCATGACTGGGG
pattern 198
CGCTGGCGGCAGGCCTAACACATGCAAGTCGAGCGGCAGCGGGAAGTAGTTTACTACT
TTGCCGGCGAGCGGCGGACGGGTGAGTAATGTCTGGGGATCTGCCTGATGGAGGGGGA
TAACTACTGGAAACGGTAGCTAATACCGCATGACCTCGCAAGAGCAAAGTGGGGGACC
TTAGGGCCTCACGCCATCGGATGAACCCAGATGGGATTAGCTAGTAGGTGGGGTAATGG
CTCACCTAGGCGACGATCCCTAGCTGGTCTGAGAGGATGACCAGCCACACTGGAACTG
AGACACGGTCCAGACTCCTACGGGAGGCAGCAGTGGGGAATATTGCACAATGGGCGCA
AGCCTGATGCAGCCATGCCGCGTGTGTGAAGAAGGCCTTCGGGTTGTAAAGCACTTTCA
GCGAGGAGGAAGGGGTTGAGTTTAATACGCTCAATCATTGACGTTACTCGCAGAAGAA
GCACCGGCTAACTCCGTGCCAGCAGCCGCGGTAATACGGAGGGTGCAAGCGTTAATCG
GAATTACTGGGCGTAAAGCGCACGCAGGCGGTTTGTTAAGTCAGATGTGAAATCCCCGC
GCTTAACGTGGGAACTGCATTTGAAACTGGCAAGCTAGAGTCTTGTAGAGGGGGGTAG
AATTCCAGGTGTAGCGGTGAAATGCGTAGAGATCTGGAGGAATACCGGTGGCGAAGGC
GGCCCCCTGGACAAAGACTGACGCTCAGGTGCGAAAGCGTGGGGAGCAAACAGGATT
AGATACCCTGGTAGTCCACGCTGTAAACGATGTCGACTTGGAGGTTGTGCCCTTGAGGC
GTGGCTTCCGGAGCTAACGCGTTAAGTCGACCGCCTGGGGAGTACGGCCGCAAGGTTA
AAACTCAAATGAATTGACGGGGGCCCGCACAAGCGGTGGAGCATGTGGTTTAATTCGAT
GCAACGCGAAGAACCTTACCTACTCTTGACATCCACAGAATTTGGCAGAGATGCTAAAG
TGCCTTCGGGAACTGTGAGACAGGTGCTGCATGGCTGTCGTCAGCTCGTGTTGTGAAAT
GTTGGGTTAAGTCCCGCAACGAGCGCAACCCTTATCCTTTGTTGCCAGCACGTAATGGT
GGGAACTCAAGGGAGACTGCCGGTGACAAACCGGAGGAAGGTGGGGATGACGTCAAG
TCATCATGGCCCTTACGAGTAGGGCTACACACGTGCTACAATGGCAGATACAAAGTGAA
GCGAACTCGCGAGAGCCAGCGGACCACATAAAGTCTGTCGTAGTCCGGATTGGAGTCT
GCAACTCGACTCCATGAAGTCGGAATCGCTAGTAATCGTAGATCAGAATGCTACGGTGA
ATACGTTCCCGGGCCTTGTACACACCGCCCGTCACACCATGGGAGTGGGTTGCAAAAG
AAGTAGGTAGCTTAACCTTCAGGAGGGCGCTTACCACTTTGTGATTCATGACTGGGG
pattern 199
CGCTGGCGGCAGGCCTAACACATGCAAGTCGAGCGGCAGCGGGAAGTAGTTTACTACT
TTGCCGGCGAGCGGCGGACGGGTGAGTAATGTCTGGGGATCTGCCTGATGGAGGGGGA
TAACTACTGGAAACGGTAGCTAATACCGCATGACCTCGCAAGAGCAAAGTGGGGGACC
TTAGGGCCTCACGCCATCGGATGAACCCAGATGGGATTAGCTAGTAGGTGGGGTAATGG
CTCACCTAGGCGACGATCCCTAGCTGGTCTGAGAGGATGACCAGCCACACTGGAACTG
AGACACGGTCCAGACTCCTACGGGAGGCAGCAGTGGGGAATATTGCACAATGGGCGCA
AGCCTGATGCAGCCATGCCGCGTGTGTGAAGAAGGCCTTCGGGTTGTAAAGCACTTTCA
GCGAGGAGGAAGGGGTTGAGTTTAATACGCTCAATCATTGACGTTACTCGCAGAAGAA
GCACCGGCTAACTCCGTGCCAGCAGCCGCGGTAATACGGAGGGTGCAAGCGTTAATCG
GAATTACTGGGCGTAAAGCGCACGCAGGCGGTTTGTTAAGTCAGATGTGAAATCCCCGC
GCTTAACGTGGGAACTGCATTTGAAACTGGCAAGCTAGAGTCTTGTAGAGGGGGGTAG
AATTCCAGGTGTAGCGGTGAAATGCGTAGAGATCTGGAGGAATACCGGTGGCGAAGGC
GGCCCCCTGGACAAAGACTGACGCTCAGGTGCGAAAGCGTGGGGAGCAAACAGGATT
AGATACCCTGGTAGTCCACGCTGTAAACGATGTCGACTTGGAGGTTGTGCCCTTGAGGC
GTGGCTTCCGGAGCTAACGCGTTAAGTCGACCGCCTGGGGAGTACGGCCGCAAGGTTA
AAACTCAAATGAATTGACGGGGGCCCGCACAAGCGGTGGAGCATGTGGTTTAATTCGAT

GCAACGCGAAGAACCTTACCTACTCTTGACATCCACAGAATTTGGCAGAGATGCTAAAG
TGCCTTCGGGAACTGTGAGACAGGTGCTGCATGGCTGTCGTCAGCTCGTGTTGTGAAAT
GTTGGGTTAAGTCCCGCAACGAGCGCAACCCTTATCCTTTGTTGCCAGCACGTAATGGT
GGGAACTCAAGGGAGACTGCCGGTGACAAACCGGAGGAAGGTGGGGATGACGTCAAG
TCATCATGGCCCTTACGAGTAGGGCTACACACGTGCTACAATGGCAGATACAAAGTGAA
GCGAACTCGCGAGAGCCAGCGGACCAAATAAAGTCTGTCGTAGTCCGGATTGGAGTCT
GCAACTCGACTCCATGAAGTCGGAATCGCTAGTAATCGTAGATCAGAATGCTACGGTGA
ATACGTTCCCGGGCCTTGTACACACCGCCCGTCACACCATGGGAGTGGGTTGCAAAAG
AAGTAGGTAGCTTAACCTTCGGGAGGGCGCTTACCACTTTGTGATTCATGACTGGGG
pattern 200
CGCTGGCGGCAGGCCTAACACATGCAAGTCGAGCGGCAGCGGGAAGTAGTTTACTACT
TTGCCGGCGAGCGGCGGACGGGTGAGTAATGTCTGGGGATCTGCCTGATGGAGGGGGA
TAACTACTGGAAACGGTGGCTAATACCGCATGACCTCGCAAGAGCAAAGTGGGGGACC
TTAGGGCCTCACGCCATCGGATGAACCCAGATGGGATTAGCTAGTAGGTGGGGTAATGG
CTCACCTAGGCGACGATCCCTAGCTGGTCTGAGAGGATGACCAGCCACACTGGAACTG
AGACACGGTCCAGACTCCTACGGGAGGCAGCAGTGGGGAATATTGCACAATGGGCGCA
AGCCTGATGCAGCCATGCCGCGTGTGTGAAGAAGGCCTTCGGGTTGTAAAGCACTTTCA
GCGAGGAGGAAGGGGTTGAGTTTAATACGCTCAATCATTGACGTTACTCGCAGAAGAA
GCACCGGCTAACTCCGTGCCAGCAGCCGCGGTAATACGGAGGGTGCAAGCGTTAATCG
GAATTACTGGGCGTAAAGCGCACGCAGGCGGTTTGTTAAGTCAGATGTGAAATCCCCGC
GCTTAACGTGGGAACTGCATTTGAAACTGGCAAGCTAGAGTCTTGTAGAGGGGGGTAG
AATTCCAGGTGTAGCGGTGAAATGCGTAGAGATCTGGAGGAATACCGGTGGCGAAGGC
GGCCCCCTGGACAAAGACTGACGCTCAGGTGCGAAAGCGTGGGGAGCAAACAGGATT
AGATACCCTGGTAGTCCACGCTGTAAACGATGTCGACTTGGAGGTTGTGCCCTTGAGGC
GTGGCTTCCGGAGCTAACGCGTTAAGTCGACCGCCTGGGGAGTACGGCCGCAAGGTTA
AAACTCAAATGAATTGACGGGGGCCCGCACAAGCGGTGGAGCATGTGGTTTAATTCGAT
GCAACGCGAAGAACCTTACCTACTCTTGACATCCACGGAATTTAGCAGAGATGCTTTAG
TGCCTTCGGGAACCGTGAGACAGGTGCTGCATGGCTGTCGTCAGCTCGTGTTGTGAAAT
GTTGGGTTAAGTCCCGCAACGAGCGCAACCCTTATCCTTTGTTGCCAGCACGTAATGGT
GGGAACTCAAGGGAGACTGCCGGTGACAAACCGGAGGAAGGTGGGGATGACGTCAAG
TCATCATGGCCCTTACGAGTAGGGCTACACACGTGCTACAATGGCAGATACAAAGTGAA
GCGAACTCGCGAGAGCCAGCGGACCACATAAAGTCTGTCGTAGTCCGGATTGGAGTCT
GCAACTCGACTCCATGAAGTCGGAATCGCTAGTAATCGTAGATCAGAATGCTACGGTGA
ATACGTTCCCGGGCCTTGTACACACCGCCCGTCACACCATGGGAGTGGGTTGCAAAAG
AAGTAGGTAGCTTAACCTTCGGGAGGGCGCTTACCACTTTGTGATTCATGACTGGGG
pattern 201
CGCTGGCGGCAGGCCTAACACATGCAAGTCGAGCGGCAGCGGGAAGTAGTTTACTACT
TCGCCGGCGAGCGGCGGACGGGTGAGTAATGTCTGGGAAACTGCCTGATGGAGGGGGA
TAACTACTGGAAACGGTAGCTAATACCGCATGACCTCGCAAGAGCAAAGTGGGGGACC
TTCGGGCCTCACGCCATCGGATGTGCCCAGATGGGATTAGCTAGTAGGTGGGGTAATGG
CTCACCTAGGCGACGATCCCTAGCTGGTCTGAGAGGATGACCAGCCACACTGGAACTG
AGACACGGTCCAGACTCCTACGGGAGGCAGCAGTGGGGAATATTGCACAATGGGCGCA
AGCCTGATGCAGCCATGCCGCGTGTGTGAAGAAGGCCTTCGGGTTGTAAAGCACTTTCA
GCGAGGAGGAAGGGTTCAGTGTTAATAGCACTGAGCATTGACGTTACTCGCAGAAGAA

GCACCGGCTAACTCCGTGCCAGCAGCCGCGGTAATACGGAGGGTGCAAGCGTTAATCG
GAATTACTGGGCGTAAAGCGCACGCAGGCGGTTTGTTAAGTCAGATGTGAAATCCCCGC
GCTTAACGTGGGAACTGCATTTGAAACTGGCAAGCTAGAGTCTTGTAGAGGGGGGTAG
AATTCCAGGTGTAGCGGTGAAATGCGTAGAGATCTGGAGGAATACCGGTGGCGAAGGC
GGCCCCCTGGACAAAGACTGACGCTCAGGTGCGAAAGCGTGGGGAGCAAACAGGATT
AGATACCCTGGTAGTCCACGCTGTAAACGATGTCGACTTGGAGGTTGTGCCCTTGAGGC
GTGGCTTCCGGAGCTAACGCGTTAAGTCGACCGCCTGGGGAGTACGGCCGCAAGGTTA
AAACTCAAATGAATTGACGGGGGCCCGCACAAGCGGTGGAGCATGTGGTTTAATTCGAT
GCAACGCGAAGAACCTTACCTACTCTTGACATCCACAGAACTTAGCAGAGATGCTTCGG
TGCCTTCGGGAACTGTGAGACAGGTGCTGCATGGCTGTCGTCAGCTCGTGTTGTGAAAT
GTTGGGTTAAGTCCCGCAACGAGCGCAACCCTTATCCTTTGTTACCAGCACGTAATGGT
GGGAACTCAAGGGAGACTGCCGGTGACAAACCGGAGGAAGGTGGGGATGACGTCAAG
TCATCATGGCCCTTACGAGTAGGGCTACACACGTGCTACAATGGCAGATACAAAGTGAA
GCGAACTCGCGAGAGCAAGCGGACCACATAAAGTCTGTCGTAGTCCGGATTGGAGTCT
GCAACTCGACTCCATGAAGTCGGAATCGCTAGTAATCGTAGATCAGAATGCTACGGTGA
ATACGTTCCCGGGCCTTGTACACACCGCCCGTCACACCATGGGAGTGGGTTGCAAAAG
AAGTAGGTAGCTTAACCTTCGGGAGGGCGCTTACCACTTTGTGATTCATGACTGGGG
pattern 202
CGCTGGCGGCAGGCCTAACACATGCAAGTCGAGCGGCAGCGGGAAGTAGTTTACTACT
TCGCCGGCGAGCGGCGGACGGGTGAGTAATGTCTGGGAAACTGCCTGATGGAGGGGGA
TAACTACTGGAAACGGTAGCTAATACCGCATGACCTCGCAAGAGCAAAGTGGGGGACC
TTCGGGCCTCACGCCATCGGATGTGCCCAGATGGGATTAGCTAGTAGGTGGGGTAATGG
CTCACCTAGGCGACGATCCCTAGCTGGTCTGAGAGGATGACCAGCCACACTGGAACTG
AGACACGGTCCAGACTCCTACGGGAGGCAGCAGTGGGGAATATTGCACAATGGGCGCA
AGCCTGATGCAGCCATGCCGCGTGTGTGAAGAAGGCCTTCGGGTTGTAAAGCACTTTCA
GCGAGGAGGAAGGGTTCAGTGTTAATAGCACTGTACATTGACGTTACTCGCAGAAGAA
GCACCGGCTAACTCCGTGCCAGCAGCCGCGGTAATACGGAGGGTGCAAGCGTTAATCG
GAATTACTGGGCGTAAAGCGCACGCAGGCGGTTTGTTAAGTCAGATGTGAAATCCCCGC
GCTTAACGTGGGAACTGCATTTGAAACTGGCAAGCTAGAGTCTTGTAGAGGGGGGTAG
AATTCCAGGTGTAGCGGTGAAATGCGTAGAGATCTGGAGGAATACCGGTGGCGAAGGC
GGCCCCCTGGACAAAGACTGACGCTCAGGTGCGAAAGCGTGGGGAGCAAACAGGATT
AGATACCCTGGTAGTCCACGCTGTAAACGATGTCGACTTGGAGGTTGTGCCCTTGAGGC
GTGGCTTCCGGAGCTAACGCGTTAAGTCGACCGCCTGGGGAGTACGGCCGCAAGGTTA
AAACTCAAATGAATTGACGGGGGCCCGCACAAGCGGTGGAGCATGTGGTTTAATTCGAT
GCAACGCGAAGAACCTTACCTACTCTTGACATCCACAGAACTTAGCAGAGATGCTTCGG
TGCCTTCGGGAACTGTGAGACAGGTGCTGCATGGCTGTCGTCAGCTCGTGTTGTGAAAT
GTTGGGTTAAGTCCCGCAACGAGCGCAACCCTTATCCTTTGTTGCCAGCACGTAATGGT
GGGAACTCAAGGGAGACTGCCGGTGACAAACCGGAGGAAGGTGGGGATGACGTCAAG
TCATCATGGCCCTTACGAGTAGGGCTACACACGTGCTACAATGGCAGATACAAAGTGAA
GCGAACTCGCGAGAGCAAGCGGACCACATAAAGTCTGTCGTAGTCCGGATTGGAGTCT
GCAACTCGACTCCATGAAGTCGGAATCGCTAGTAATCGTAGATCAGAATGCTACGGTGA
ATACGTTCCCGGGCCTTGTACACACCGCCCGTCACACCATGGGAGTGGGTTGCAAAAG
AAGTAGGTAGCTTAACCTTCGGGAGGGCGCTTACCACTTTGTGATTCATGACTGGGG
pattern 203

CGCTGGCGGCAGGCCTAACACATGCAAGTCGAGCGGCAGCGGAAAGTAGCTTGCTACT
TTGCCGGCGAGCGGCGGACGGGTGAGTAATGTCTGGGGATCTGCCTGATGGAGGGGGA
TAACTACTGGAAACGGTAGCTAATACCGCATGACCTCGAAAGAGCAAAGTGGGGGACC
TTCGGGCCTCACGCCATCGGATGAACCCAGATGGGATTAGCTAGTAGGTGAGGTAATGG
CTCACCTAGGCGACGATCCCTAGCTGGTCTGAGAGGATGACCAGCCACACTGGAACTG
AGACACGGTCCAGACTCCTACGGGAGGCAGCAGTGGGGAATATTGCACAATGGGCGCA
AGCCTGATGCAGCCATGCCGCGTGTGTGAAGAAGGCCTTCGGGTTGTAAAGCACTTTCA
GCGAGGAGGAAGGCATTGTGGTTAATAACCACAGTGATTGACGTTACTCGCAGAAGAA
GCACCGGCTAACTCCGTGCCAGCAGCCGCGGTAATACGGAGGGTGCAAGCGTTAATCG
GAATTACTGGGCGTAAAGCGCACGCAGGCGGTTTGTTAAGTCAGATGTGAAATCCCCGC
GCTTAACGTGGGAACTGCATTTGAAACTGGCAAGCTAGAGTCTTGTAGAGGGGGGTAG
AATTCCAGGTGTAGCGGTGAAATGCGTAGAGATCTGGAGGAATACCGGTGGCGAAGGC
GGCCCCCTGGACAAAGACTGACGCTCAGGTGCGAAAGCGTGGGGAGCAAACAGGATT
AGATACCCTGGTAGTCCACGCTGTAAACGATGTCGACTTGGAGGTTGTGCCCTTGAGGT
GTGGCTTCCGGAGCTAACGCGTTAAGTCGACCGCCTGGGGAGTACGGCCGCAAGGTTA
AAACTCAAATGAATTGACGGGGGCCCGCACAAGCGGTGGAGCATGTGGTTTAATTCGAT
GCAACGCGAAGAACCTTACCTACTCTTGACATCCACAGAACTTAGCAGAGATGCTTCGG
TGCCTTCGGGAACTGTGAGACAGGTGCTGCATGGCTGTCGTCAGCTCGTGTTGTGAAAT
GTTGGGTTAAGTCCCGCAACGAGCGCAACCCTTATCCTTTGTTGCCAGCACGTAATGGT
GGGAACTCAAGGGAGACTGCCGGTGACAAACCGGAGGAAGGTGGGGATGACGTCAAG
TCATCATGGCCCTTACGAGTAGGGCTACACACGTGCTACAATGGCAGATACAAAGTGAA
GCGAACTCGCGAGAGCAAGCGGACCACATAAAGTCTGTCGTAGTCCGGATTGGAGTCT
GCAACTCGACTCCATGAAGTCGGAATCGCTAGTAATCGTAGATCAGAATGCTACGGTGA
ATACGTTCCCGGGCCTTGTACACACCGCCCGTCACACCATGGGAGTGGGTTGCAAAAG
AAGTAGGTAGCTTAACCTTCGGGAGGGCGCTTACCACTTTGTGATTCATGACTGGGG

pattern 204

CGCTGGCGGCAGGCCTAACACATGCAAGTCGAGCGGCAGCGGGAAGTAGTTTACTACT
TTGCCGGCGAGCGGCGGACGGGTGAGTAATGTCTGGGAAACTGCCTGATGGAGGGGGA
TAACTACTGGAAACGGTAGCTAATACCGCATGACCTCGCAAGAGCAAAGTGGGGGACC
TTAGGGCCTCACGCCATCGGATGTGCCCAGATGGGATTAGCTAGTAGGTGGGGTAACGG
CTCACCTAGGCGACGATCCCTAGCTGGTCTGAGAGGATGACCAGCCACACTGGAACTG
AGACACGGTCCAGACTCCTACGGGAGGCAGCAGTGGGGAATATTGCACAATGGGCGCA
AGCCTGATGCAGCCATGCCGCGTGTGTGAAGAAGGCCTTCGGGTTGTAAAGCACTTTCA
GCGAGGAGGAAGGCATTGTGGTTAATAACCGCAGTGATTGACGTTACTCGCAGAAGAA
GCACCGGCTAACTCCGTGCCAGCAGCCGCGGTAATACGGAGGGTGCAAGCGTTAATCG
GAATTACTGGGCGTAAAGCGCACGCAGGCGGTTTGTTAAGTCAGATGTGAAATCCCCGC
GCTTAACGTGGGAACTGCATTTGAAACTGGCAAGCTAGAGTCTTGTAGAGGGGGGTAG
AATTCCAGGTGTAGCGGTGAAATGCGTAGAGATCTGGAGGAATACCGGTGGCGAAGGC
GGCCCCCTGGACAAAGACTGACGCTCAGGTGCGAAAGCGTGGGGAGCAAACAGGATT
AGATACCCTGGTAGTCCACGCTGTAAACGATGTCGACTTGGAGGTTGTGCCCTTGAGGC
GTGGCTTCCGGAGCTAACGCGTTAAGTCGACCGCCTGGGGAGTACGGCCGCAAGGTTA
AAACTCAAATGAATTGACGGGGGCCCGCACAAGCGGTGGAGCATGTGGTTTAATTCGAT
GCAACGCGAAGAACCTTACCTACTCTTGACATCCACAGAACTTAGCAGAGATGCTTCGG
TGCCTTCGGGAACTGTGAGACAGGTGCTGCATGGCTGTCGTCAGCTCGTGTTGTGAAAT

GTTGGGTTAAGTCCCGCAACGAGCGCAACCCTTATCCTTTGTTGCCAGCACGTAATGGT
GGGAACTCAAAGGAGACTGCCGGTGATAAACCGGAGGAAGGTGGGGATGACGTCAAG
TCATCATGGCCCTTACGAGTAGGGCTACACACGTGCTACAATGGCAGATACAAAGTGAA
GCGAACTCGCGAGAGCAAGCGGACCACATAAAGTCTGTCGTAGTCCGGATTGGAGTCT
GCAACTCGACTCCATGAAGTCGGAATCGCTAGTAATCGTAGATCAGAATGCTACGGTGA
ATACGTTCCCGGGCCTTGTACACACCGCCCGTCACACCATGGGAGTGGGTTGCAAAAG
AAGTAGGTAGCTTAACCTTCGGGAGGGCGCTTACCACTTTGTGATTCATGACTGGGG
pattern 205
CGCTGGCGGCAGGCCTAACACATGCAAGTCGAGCGGCAGCGGGAAGTAGTTTACTACT
TCGCCGGCGAGCGGCGGACGGGTGAGTAATGTCTGGGAAACTGCCTGATGGAGGGGGA
TAACTACTGGAAACGGTAGCTAATACCGCATGACCTCGCAAGAGCAAAGTGGGGGACC
TTAGGGCCTCACGCCATCGGATGTGCCCAGATGGGATTAGCTAGTAGGTGGGGTAACGG
CTCACCTAGGCGACGATCCCTAGCTGGTCTGAGAGGATGACCAGCCACACTGGAACTG
AGACACGGTCCAGACTCCTACGGGAGGCAGCAGTGGGGAATATTGCACAATGGGCGCA
AGCCTGATGCAGCCATGCCGCGTGTGTGAAGAAGGCCTTCGGGTTGTAAAGCACTTTCA
GCGAGGAGGAAGGCATTGTGGTTAATAACCGCAGTGATTGACGTTACTCGCAGAAGAA
GCACCGGCTAACTCCGTGCCAGCAGCCGCGGTAATACGGAGGGTGCAAGCGTTAATCG
GAATTACTGGGCGTAAAGCGCACGCAGGCGGTTTGTTAAGTCAGATGTGAAATCCCCGC
GCTTAACGTGGGAACTGCATTTGAAACTGGCAAGCTAGAGTCTTGTAGAGGGGGGTAG
AATTCCAGGTGTAGCGGTGAAATGCGTAGAGATCTGGAGGAATACCGGTGGCGAAGGC
GGCCCCCTGGACAAAGACTGACGCTCAGGTGCGAAAGCGTGGGGAGCAAACAGGATT
AGATACCCTGGTAGTCCACGCTGTAAACGATGTCGACTTGGAGGTTGTGCCCTTGAGGC
GTGGCTTCCGGAGCTAACGCGTTAAGTCGACCGCCTGGGGAGTACGGCCGCAAGGTTA
AAACTCAAATGAATTGACGGGGGCCCGCACAAGCGGTGGAGCATGTGGTTTAATTCGAT
GCAACGCGAAGAACCTTACCTACTCTTGACATCCACAGAACTTAGCAGAGATGCTTCGG
TGCCTTCGGGAACTGTGAGACAGGTGCTGCATGGCTGTCGTCAGCTCGTGTTGTGAAAT
GTTGGGTTAAGTCCCGCAACGAGCGCAACCCTTATCCTTTGTTGCCAGCACGTAATGGT
GGGAACTCAAAGGAGACTGCCGGTGATAAACCGGAGGAAGGTGGGGATGACGTCAAG
TCATCATGGCCCTTACGAGTAGGGCTACACACGTGCTACAATGGCAGATACAAAGTGAA
GCGAACTCGCGAGAGCAAGCGGACCACATAAAGTCTGTCGTAGTCCGGATTGGAGTCT
GCAACTCGACTCCATGAAGTCGGAATCGCTAGTAATCGTAGATCAGAATGCTACGGTGA
ATACGTTCCCGGGCCTTGTACACACCGCCCGTCACACCATGGGAGTGGGTTGCAAAAG
AAGTAGGTAGCTTAACCTTCGGGAGGGCGCTTACCACTTTGTGATTCATGACTGGGG
pattern 206
CGCTGGCGGCAGGCCTAACACATGCAAGTCGAGCGGCAGCGGAAAGTAGCTTGCTACT
TTGCCGGCGAGCGGCGGACGGGTGAGTAATGTCTGGGGATCTGCCTGATGGAGGGGGA
TAACTACTGGAAACGGTAGCTAATACCGCATGACCTCGAAAGAGCAAAGTGGGGGACC
TTCGGGCCTCACGCCATCGGATGAACCCAGATGGGATTAGCTAGTAGGTGGGGTAATGG
CTCACCTAGGCGACGATCCCTAGCTGGTCTGAGAGGATGACCAGCCACACTGGAACTG
AGACACGGTCCAGACTCCTACGGGAGGCAGCAGTGGGGAATATTGCACAATGGGCGCA
AGCCTGATGCAGCCATGCCGCGTGTGTGAAGAAGGCCTTCGGGTTGTAAAGCACTTTCA
GCGAGGAGGAAGGCATTGTGGTTAATAACCACAGTGATTGACGTTACTCGCAGAAGAA
GCACCGGCTAACTCCGTGCCAGCAGCCGCGGTAATACGGAGGGTGCAAGCGTTAATCG
GAATTACTGGGCGTAAAGCGCACGCAGGCGGTTTGTTAAGTCAGATGTGAAATCCCCGC

GCTTAACGTGGGAACTGCATTTGAAACTGGCAAGCTAGAGTCTTGTAGAGGGGGGTGG
AATTCCAGGTGTAGCGGTGAAATGCGTAGAGATCTGGAGGAATACCGGTGGCGAAGGC
GGCCCCCTGGACAAAGACTGACGCTCAGGTGCGAAAGCGTGGGGAGCAAACAGGATT
AGATACCCTGGTAGTCCACGCTGTAAACGATGTCGACTTGGAGGTTGTGCCCTTGAGGC
GTGGCTTCCGGAGCTAACGCGTTAAGTCGACCGCCTGGGGAGTACGGCCGCAAGGTTA
AAACTCAAATGAATTGACGGGGGCCCGCACAAGCGGTGGAGCATGTGGTTTAATTCGAT
GCAACGCGAAGAACCTTACCTACTCTTGACATCCACAGAACTTAGCAGAGATGCTTCGG
TGCCTTCGGGAACTGTGAGACAGGTGCTGCATGGCTGTCGTCAGCTCGTGTTGTGAAAT
GTTGGGTTAAGTCCCGCAACGAGCGCAACCCTTATCCTTTGTTGCCAGCACGTAATGGT
GGGAACTCAAGGGAGACTGCCGGTGACAAACCGGAGGAAGGTGGGGATGACGTCAAG
TCATCATGGCCCTTACGAGTAGGGCTACACACGTGCTACAATGGCAGATACAAAGTGAA
GCGAACTCGCGAGAGCAAGCGGACCACATAAAGTCTGTCGTAGTCCGGATTGGAGTCT
GCAACTCGACTCCATGAAGTCGGAATCGCTAGTAATCGTAGATCAGAATGCTACGGTGA
ATACGTTCCCGGGCCTTGTACACACCGCCCGTCACACCATGGGAGTGGGTTGCAAAAG
AAGTAGGTAGCTTAACCTTCGGGAGGGCGCTTACCACTTTGTGATTCATGACTGGGG
pattern 207
CGCTGGCGGCAGGCCTAACACATGCAAGTCGAGCGGCAGCGGAAAGTAGCTTGCTACT
TTGCCGGCGAGCGGCGGACGGGTGAGTAATGTCTGGGGATCTGCCTGATGGAGGGGGA
TAACTACTGGAAACGGTAGCTAATACCGCATGACCTCGAAAGAGCAAAGTGGGGGACC
TTCGGGCCTCACGCCATCGGATGAACCCAGATGGGATTAGCTAGTAGGTGGGGTAATGG
CTCACCTAGGCGACGATCCCTAGCTGGTCTGAGAGGATGACCAGCCACACTGGAACTG
AGACACGGTCCAGACTCCTACGGGAGGCAGCAGTGGGGAATATTGCACAATGGGCGCA
AGCCTGATGCAGCCATGCCGCGTGTGTGAAGAAGGCCTTCGGGTTGTAAAGCACTTTCA
GCGAGGAGGAAGGCATTGTGGTTAATAACCGCAGTGATTGACGTTACTCGCAGAAGAA
GCACCGGCTAACTCCGTGCCAGCAGCCGCGGTAATACGGAGGGTGCAAGCGTTAATCG
GAATTACTGGGCGTAAAGCGCACGCAGGCGGTTTGTTAAGTCAGATGTGAAATCCCCGC
GCTTAACGTGGGAACTGCATTTGAAACTGGCAAGCTAGAGTCTTGTAGAGGGGGGTGG
AATTCCAGGTGTAGCGGTGAAATGCGTAGAGATCTGGAGGAATACCGGTGGCGAAGGC
GGCCCCCTGGACAAAGACTGACGCTCAGGTGCGAAAGCGTGGGGAGCAAACAGGATT
AGATACCCTGGTAGTCCACGCTGTAAACGATGTCGACTTGGAGGTTGTGCCCTTGAGGC
GTGGCTTCCGGAGCTAACGCGTTAAGTCGACCGCCTGGGGAGTACGGCCGCAAGGTTA
AAACTCAAATGAATTGACGGGGGCCCGCACAAGCGGTGGAGCATGTGGTTTAATTCGAT
GCAACGCGAAGAACCTTACCTACTCTTGACATCCACAGAACTTAGCAGAGATGCTTTAG
TGCCTTCGGGAACCGTGAGACAGGTGCTGCATGGCTGTCGTCAGCTCGTGTTGTGAAAT
GTTGGGTTAAGTCCCGCAACGAGCGCAACCCTTATCCTTTGTTGCCAGCACGTAATGGT
GGGAACTCAAGGGAGACTGCCGGTGACAAACCGGAGGAAGGTGGGGATGACGTCAAG
TCATCATGGCCCTTACGAGTAGGGCTACACACGTGCTACAATGGCAGATACAAAGTGAA
GCGAACTCGCGAGAGCAAGCGGACCACATAAAGTCTGTCGTAGTCCGGATTGGAGTCT
GCAACTCGACTCCATGAAGTCGGAATCGCTAGTAATCGTAGATCAGAATGCTACGGTGA
ATACGTTCCCGGGCCTTGTACACACCGCCCGTCACACCATGGGAGTGGGTTGCAAAAG
AAGTAGGTAGCTTAACCTTCGGGAGGGCGCTTACCACTTTGTGATTCATGACTGGGG
pattern 208
CGCTGGCGGCAGGCCTAACACATGCAAGTCGAGCGGCAGCGGAAAGTAGCTTGCTACT
TTGCCGGCGAGCGGCGGACGGGTGAGTAATGTCTGGGGATCTGCCTGATGGAGGGGGA

TAACTACTGGAAACGGTAGCTAATACCGCATGACCTCGAAAGAGCAAAGTGGGGGACC
TTCGGGCCTCACGCCATCGGATGAACCCAGATGGGATTAGCTAGTAGGTGGGGTAATGG
CTCACCTAGGCGACGATCCCTAGCTGGTCTGAGAGGATGACCAGCCACACTGGAACTG
AGACACGGTCCAGACTCCTACGGGAGGCAGCAGTGGGGAATATTGCACAATGGGCGCA
AGCCTGATGCAGCCATGCCGCGTGTGTGAAGAAGGCCTTCGGGTTGTAAAGCACTTTCA
GCGAGGAGGAAGGCATTGTGGTTAATAACCGCAGTGATTGACGTTACTCGCAGAAGAA
GCACCGGCTAACTCCGTGCCAGCAGCCGCGGTAATACGGAGGGTGCAAGCGTTAATCG
GAATTACTGGGCGTAAAGCGCACGCAGGCGGTTTGTTAAGTCAGATGTGAAATCCCCGC
GCTTAACGTGGGAACTGCATTTGAAACTGGCAAGCTAGAGTCTTGTAGAGGGGGGTAG
AATTCCAGGTGTAGCGGTGAAATGCGTAGAGATCTGGAGGAATACCGGTGGCGAAGGC
GGCCCCCTGGACAAAGACTGACGCTCAGGTGCGAAAGCGTGGGGAGCAAACAGGATT
AGATACCCTGGTAGTCCACGCTGTAAACGATGTCGACTTGGAGGTTGTGCCCTTGAGGC
GTGGCTTCCGGAGCTAACGCGTTAAGTCGACCGCCTGGGGAGTACGGCCGCAAGGTTA
AAACTCAAATGAATTGACGGGGGCCCGCACAAGCGGTGGAGCATGTGGTTTAATTCGAT
GCAACGCGAAGAACCTTACCTACTCTTGACATCCACAGAACTTAGCAGAGATGCTTTAG
TGCCTTCGGGAACTGTGAGACAGGTGCTGCATGGCTGTCGTCAGCTCGTGTTGTGAAAT
GTTGGGTTAAGTCCCGCAACGAGCGCAACCCTTATCCTTTGTTGCCAGCACGTAATGGT
GGGAACTCAAGGGAGACTGCCGGTGACAAACCGGAGGAAGGTGGGGATGACGTCAAG
TCATCATGGCCCTTACGAGTAGGGCTACACACGTGCTACAATGGCAGATACAAAGTGAA
GCGAACTCGCGAGAGCAAGCGGACCACATAAAGTCTGTCGTAGTCCGGATTGGAGTCT
GCAACTCGACTCCATGAAGTCGGAATCGCTAGTAATCGTAGATCAGAATGCTACGGTGA
ATACGTTCCCGGGCCTTGTACACACCGCCCGTCACACCATGGGAGTGGGTTGCAAAAG
AAGTAGGTAGCTTAACCTTCGGGAGGGCGCTTACCACTTTGTGATTCATGACTGGGG
pattern 209
CGCTGGCGGCAGGCCTAACACATGCAAGTCGAGCGGCAGCGGGAAGTAGTTTACTACT
TTGCCGGCGAGCGGCGGACGGGTGAGTAATGTCTGGGAAACTGCCTGATGGAGGGGGA
TAACTACTGGAAACGGTAGCTAATACCGCATGACCTCGCAAGAGCAAAGTGGGGGACC
TTCGGGCCTCACGCCATCGGATGTGCCCAGATGGGATTAGCTAGTAGGTGGGGTAATGG
CTCACCTAGGCGACGATCCCTAGCTGGTCTGAGAGGATGACCAGCCACACTGGAACTG
AGACACGGTCCAGACTCCTACGGGAGGCAGCAGTGGGGAATATTGCACAATGGGCGCA
AGCCTGATGCAGCCATGCCGCGTGTGTGAAGAAGGCCTTCGGGTTGTAAAGCACTTTCA
GCGAGGAGGAAGGCAGTCGTGTTAATAGCACGATTGATTGACGTTACTCGCAGAAGAA
GCACCGGCTAACTCCGTGCCAGCAGCCGCGGTAATACGGAGGGTGCAAGCGTTAATCG
GAATTACTGGGCGTAAAGCGCACGCAGGCGGTTTGTTAAGTCAGATGTGAAATCCCCGC
GCTTAACGTGGGAACTGCATTTGAAACTGGCAAGCTAGAGTCTTGTAGAGGGGGGTAG
AATTCCAGGTGTAGCGGTGAAATGCGTAGAGATCTGGAGGAATACCGGTGGCGAAGGC
GGCCCCCTGGACAAAGACTGACGCTCAGGTGCGAAAGCGTGGGGAGCAAACAGGATT
AGATACCCTGGTAGTCCACGCTGTAAACGATGTCGACTTGGAGGTTGTGCCCTTGAGGC
GTGGCTTCCGGAGCTAACGCGTTAAGTCGACCGCCTGGGGAGTACGGCCGCAAGGTTA
AAACTCAAATGAATTGACGGGGGCCCGCACAAGCGGTGGAGCATGTGGTTTAATTCGAT
GCAACGCGAAGAACCTTACCTACTCTTGACATCCACAGAACTTAGCAGAGATGCTTCGG
TGCCTTCGGGAACTGTGAGACAGGTGCTGCATGGCCGTCGTCAGCTCGTGTTGTGAAAT
GTTGGGTTAAGTCCCGCAACGAGCGCAACCCTTATCCTTTGTTGCCAGCACGTAATGGT
GGGAACTCAAGGGAGACTGCCGGTGACAAACCGGAGGAAGGTGGGGATGACGTCAAG

TCATCATGGCCCTTACGAGTAGGGCTACACACGTGCTACAATGGCAGATACAAAGTGAA
GCGAACTCGCGAGAGCAAGCGGACCACATAAAGTCTGTCGTAGTCCGGATTGGAGTCT
GCAACTCGACTCCATGAAGTCGGAATCGCTAGTAATCGTAGATCAGAATGCTACGGTGA
ATACGTTCCCGGGCCTTGTACACACCGCCCGTCACACCATGGGAGTGGGTTGCAAAAG
AAGTAGGTAGCTTAACCTTCGGGAGGGCGCTTACCACTTTGTGATTCATGACTGGGG
pattern 210
CGCTGGCGGCAGGCCTAACACATGCAAGTCGAGCGGCAGCGGGAAGTAGTTTACTACT
TCGCCGGCGAGCGGCGGACGGGTGAGTAATGTCTGGGAAACTGCCTGATGGAGGGGGA
TAACTACTGGAAACGGTAGCTAATACCGCATGACCTCGCAAGAGCAAAGTGGGGGACC
TTAGGGCCTCACGCCATCGGATGTGCCCAGATGGGATTAGCTAGTAGGTGGGGTAATGG
CTCACCTAGGCGACGATCCCTAGCTGGTCTGAGAGGATGACCAGCCACACTGGAACTG
AGACACGGTCCAGACTCCTACGGGAGGCAGCAGTGGGGAATATTGCACAATGGGCGCA
AGCCTGATGCAGCCATGCCGCGTGTGTGAAGAAGGCCTTCGGGTTGTAAAGCACTTTCA
GCGAGGAGGAAGGCAATCGTGTTAATAGCACGGTTGATTGACGTTACTCGCAGAAGAA
GCACCGGCTAACTCCGTGCCAGCAGCCGCGGTAATACGGAGGGTGCAAGCGTTAATCG
GAATTACTGGGCGTAAAGCGCACGCAGGCGGTTTGTTAAGTCAGATGTGAAATCCCCGC
GCTTAACGTGGGAACTGCATTTGAAACTGGCAAGCTAGAGTCTTGTAGAGGGGGGTAG
AATTCCAGGTGTAGCGGTGAAATGCGTAGAGATCTGGAGGAATACCGGTGGCGAAGGC
GGCCCCCTGGACAAAGACTGACGCTCAGGTGCGAAAGCGTGGGGAGCAAACAGGATT
AGATACCCTGGTAGTCCACGCTGTAAACGATGTCGACTTGGAGGTTGTGCCCTTGAGGC
GTGGCTTCCGGAGCTAACGCGTTAAGTCGACCGCCTGGGGAGTACGGCCGCAAGGTTA
AAACTCAAATGAATTGACGGGGGCCCGCACAAGCGGTGGAGCATGTGGTTTAATTCGAT
GCAACGCGAAGAACCTTACCTACTCTTGACATCCACAGAACTTAGCAGAGATGCTTCGG
TGCCTTCGGGAACTGTGAGACAGGTGCTGCATGGCTGTCGTCAGCTCGTGTTGTGAAAT
GTTGGGTTAAGTCCCGCAACGAGCGCAACCCTTATCCTTTGTTGCCAGCACGTAATGGT
GGGAACTCAAGGGAGACTGCCGGTGACAAACCGGAGGAAGGTGGGGATGACGTCAAG
TCATCATGGCCCTTACGAGTAGGGCTACACACGTGCTACAATGGCAGATACAAAGTGAA
GCGAACTCGCGAGAGCAAGCGGACCACATAAAGTCTGTCGTAGTCCGGATTGGAGTCT
GCAACTCGACTCCATGAAGTCGGAATCGCTAGTAATCGTAGATCAGAATGCTACGGTGA
ATACGTTCCCGGGCCTTGTACACACCGCCCGTCACACCATGGGAGTGGGTTGCAAAAG
AAGTAGGTAGCTTAACCTCCGGGAGGGCGCTTACCACTTTGTGATTCATGACTGGGG
pattern 211
CGCTGGCGGCAGGCCTAACACATGCAAGTCGAGCGGCAGCGGAAAGTAGCTTGCTACT
TTGCCGGCGAGCGGCGGACGGGTGAGTAATGTCTGGGAAACTGCCTGATGGAGGGGGA
TAACTACTGGAAACGGTAGCTAATACCGCATGACCTCGCAAGAGCAAAGTGGGGGACC
TTCGGGCCTCACGCCATCGGATGTGCCCAGATGGGATTAGCTAGTAGGTGAGGTAATGG
CTCACCTAGGCGACGATCCCTAGCTGGTCTGAGAGGATGACCAGCCACACTGGAACTG
AGACACGGTCCAGACTCCTACGGGAGGCAGCAGTGGGGAATATTGCACAATGGGCGCA
AGCCTGATGCAGCCATGCCGCGTGTGTGAAGAAGGCCTTCGGGTTGTAAAGCACTTTCA
GCGAGGAGGAAGGCAGTCGTGTTAATAGCACGGTTGATTGACGTTACTCGCAGAAGAA
GCACCGGCTAACTCCGTGCCAGCAGCCGCGGTAATACGGAGGGTGCAAGCGTTAATCG
GAATTACTGGGCGTAAAGCGCACGCAGGCGGTTTGTTAAGTCAGATGTGAAATCCCCGC
GCTTAACGTGGGAACTGCATTTGAAACTGGCAAGCTAGAGTCTTGTAGAGGGGGGTAG
AATTCCAGGTGTAGCGGTGAAATGCGTAGAGATCTGGAGGAATACCGGTGGCGAAGGC

GGCCCCCTGGACAAAGACTGACGCTCAGGTGCGAAAGCGTGGGGAGCAAACAGGATT
AGATACCCTGGTAGTCCACGCTGTAAACGATGTCGACTTGGAGGTTGTGCCCTTGAGGC
GTGGCTTCCGGAGCTAACGCGTTAAGTCGACCGCCTGGGGAGTACGGCCGCAAGGTTA
AAACTCAAATGAATTGACGGGGGCCCGCACAAGCGGTGGAGCATGTGGTTTAATTCGAT
GCAACGCGAAGAACCTTACCTACTCTTGACATCCACAGAACTTAGCAGAGATGCTTCGG
TGCCTTCGGGAACTGTGAGACAGGTGCTGCATGGCTGTCGTCAGCTCGTGTTGTGAAAT
GTTGGGTTAAGTCCCGCAACGAGCGCAACCCTTATCCTTTGTTGCCAGCACGTAATGGT
GGGAACTCAAGGGAGACTGCCGGTGACAAACCGGAGGAAGGTGGGGATGACGTCAAG
TCATCATGGCCCTTACGAGTAGGGCTACACACGTGCTACAATGGCAGATACAAAGTGAA
GCGAACTCGCGAGAGCCAGCGGACCACATAAAGTCTGTCGTAGTCCGGATTGGAGTCT
GCAACTCGACTCCATGAAGTCGGAATCGCTAGTAATCGTAGATCAGAATGCTACGGTGA
ATACGTTCCCGGGCCTTGTACACACCGCCCGTCACACCATGGGAGTGGGTTGCAAAAG
AAGTAGGTAGCTTAACCTTCGGGAGGGCGCTTACCACTTTGTGATTCATGACTGGGG
pattern 212
CGCTGGCGGCAGGCCTAACACATGCAAGTCGAGCGGCAGCGGGAAGTAGTTTACTACT
TTGCCGGCGAGCGGCGGACGGGTGAGTAATGTCTGGGAAACTGCCTGATGGAGGGGGA
TAACTACTGGAAACGGTAGCTAATACCGCATGACCTCGCAAGAGCAAAGTGGGGGACC
TTCGGGCCTCACGCCATCGGATGTGCCCAGATGGGATTAGCTAGTAGGTGGGGTAATGG
CTCACCTAGGCGACGATCCCTAGCTGGTCTGAGAGGATGACCAGCCACACTGGAACTG
AGACACGGTCCAGACTCCTACGGGAGGCAGCAGTGGGGAATATTGCACAATGGGCGCA
AGCCTGATGCAGCCATGCCGCGTGTGTGAAGAAGGCCTTCGGGTTGTAAAGCACTTTCA
GCGAGGAGGAAGGCAGTCGTGTTAATAGCACGATTGATTGACGTTACTCGCAGAAGAA
GCACCGGCTAACTCCGTGCCAGCAGCCGCGGTAATACGGAGGGTGCAAGCGTTAATCG
GAATTACTGGGCGTAAAGCGCACGCAGGCGGTTTGTTAAGTCAGATGTGAAATCCCCGC
GCTTAACGTGGGAACTGCATTTGAAACTGGCAAGCTAGAGTCTTGTAGAGGGGGGTAG
AATTCCAGGTGTAGCGGTGAAATGCGTAGAGATCTGGAGGAATACCGGTGGCGAAGGC
GGCCCCCTGGACAAAGACTGACGCTCAGGTGCGAAAGCGTGGGGAGCAAACAGGATT
AGATACCCTGGTAGTCCACGCTGTAAACGATGTCGACTTGGAGGTTGTGCCCTTGAGGT
GTGGCTTCCGGAGCTAACGCGTTAAGTCGACCGCCTGGGGAGTACGGCCGCAAGGTTA
AAACTCAAATGAATTGACGGGGGCCCGCACAAGCGGTGGAGCATGTGGTTTAATTCGAT
GCAACGCGAAGAACCTTACCTACTCTTGACATCCACAGAACTTAGCAGAGATGCTTCGG
TGCCTTCGGGAACTGTGAGACAGGTGCTGCATGGCTGTCGTCAGCTCGTGTTGTGAAAT
GTTGGGTTAAGTCCCGCAACGATCGCAACCCTTATCCTTTGTTGCCAGCACGTAATGGTG
GGAACTCAAGGGAGACTGCCGGTGACAAACCGGAGGAAGGTGGGGATGACGTCAAGT
CATCATGGCCCTTACGAGTAGGGCTACACACGTGCTACAATGGCAGATACAAAGTGAAG
CGAACTCGCGAGAGCAAGCGGACCACATAAAGTCTGTCGTAGTCCGGATTGGAGTCTG
CAACTCGACTCCATGAAGTCGGAATCGCTAGTAATCGTAGATCAGAATGCTACGGTGAAT
ACGTTCCCGGGCCTTGTACACACCGCCCGTCACACCATGGGAGTGGGTTGCAAAAGAA
GTAGGTAGCTTAACCTTCGGGAGGGCGCTTACCACTTTGTGATTCATGACTGGGG
pattern 213
CGCTGGCGGCAGGCCTAACACATGCAAGTCGAGCGGCAGCGGGAAGTAGTTTACTACT
TTGCCGGCGAGCGGCGGACGGGTGAGTAATGTCTGGGAAACTGCCTGATGGAGGGGGA
TAACTACTGGAAACGGTAGCTAATACCGCATGACCTCGCAAGAGCAAAGTGGGGGACC
TTCGGGCCTCACGCCATCGGATGTGCCCAGATGGGATTAGCTAGTAGGTGGGATAATGG

CTCACCTAGGCGACGATCCCTAGCTGGTCTGAGAGGATGACCAGCCACACTGGAACTG
AGACACGGTCCAGACTCCTACGGGAGGCAGCAGTGGGGAATATTGCACAATGGGCGCA
AGCCTGATGCAGCCATGCCGCGTGTGTGAAGAAGGCCTTCGGGTTGTAAAGCACTTTCA
GCGAGGAGGAAGGCAGTCGTGTTAATAGCACGATTGATTGACGTTACTCGCAGAAGAA
GCACCGGCTAACTCCGTGCCAGCAGCCGCGGTAATACGGAGGGTGCAAGCGTTAATCG
GAATTACTGGGCGTAAAGCGCACGCAGGCGGTTTGTTAAGTCAGATGTGAAATCCCCGC
GCTTAACGTGGGAACTGCATTTGAAACTGGCAAGCTAGAGTCTTGTAGAGGGGGGTAG
AATTCCAGGTGTAGCGGTGAAATGCGTAGAGATCTGGAGGAATACCGGTGGCGAAGGC
GGCCCCCTGGACAAAGACTGACGCTCAGGTGCGAAAGCGTGGGGAGCAAACAGGATT
AGATACCCTGGTAGTCCACGCTGTAAACGATGTCGACTTGGAGGTTGTGCCCTTGAGGC
GTGGCTTCCGGAGCTAACGCGTTAAGTCGACCGCCTGGGGAGTACGGCCGCAAGGTTA
AAACTCAAATGAATTGACGGGGGCCCGCACAAGCGGTGGAGCATGTGGTTTAATTCGAT
GCAACGCGAAGAACCTTACCTACTCTTGACATCCACAGAACTTAGCAGAGATGCTTCGG
TGCCTTCGGGAACTGTGAGACAGGTGCTGCATGGCTGTCGTCAGCTCGTGTTGTGAAAT
GTTGGGTTAAGTCCCGCAACGAGCGCAACCCTTATCCTTTGTTGCCAGCACGTAATGGT
GGGAACTCAAGGGAGACTGCCGGTGACAAACCGGAGGAAGGTGGGGATGACGTCAAG
TCATCATGGCCCTTACGAGTAGGGCTACACACGTGCTACAATGGCAGATACAAAGTGAA
GCGAACTCGCGAGAGCAAGCGGACCACATAAAGTCTGTCGTAGTCCGGATTGGAGTCT
GCAACTCGACTCCATGAAGTCGGAATCGCTAGTAATCGTAGATCAGAATGCTACGGTGA
ATACGTTCCCGGGCCTTGTACACACCGCCCGTCACACCATGGGAGTGGGTTGCAAAAG
AAGTAGGTAGCTTAACCTTCGGGAGGGCGCTTACCACTTTGTGATTCATGACTGGGG
pattern 214
CGCTGGCGGCAGGCCTAACACATGCAAGTCGAGCGGCAGCGGGAAGTAGTTTACTACT
TTGCCGGCGAGCGGCGGACGGGTGAGTAATGTCTGGGAAACTGCCTGATGGAGGGGGA
TAACTACTGGAAACGGTAGCTAATACCGCATGACCTCGTAAGAGCAAAGTGGGGGACCT
TCGGGCCTCACGCCATCGGATGTGCCCAGATGGGATTAGCTAGTAGGTGAGGTAATGGC
TCACCTAGGCGACGATCCCTAGCTGGTCTGAGAGGATGACCAGCCACACTGGAACTGA
GACACGGTCCAGACTCCTACGGGAGGCAGCAGTGGGGAATATTGCACAATGGGCGCAA
GCCTGATGCAGCCATGCCGCGTGTGTGAAGAAGGCCTTCGGGTTGTAAAGCACTTTCAG
CGAGGAGGAAGGCAGTCGTGTTAATAGCACGGTTGATTGACGTTACTCGCAGAAGAAG
CACCGGCTAACTCCGTGCCAGCAGCCGCGGTAATACGGAGGGTGCAAGCGTTAATCGG
AATTACTGGGCGTAAAGCGCACGCAGGCGGTTTGTTAAGTCAGATGTGAAATCCCCGCG
CTTAACGTGGGAACTGCATTTGAAACTGGCAAGCTAGAGTCTTGTAGAGGGGGGTAGA
ATTCCAGGTGTAGCGGTGAAATGCGTAGAGATCTGGAGGAATACCGGTGGCGAAGGCG
GCCCCCTGGACAAAGACTGACGCTCAGGTGCGAAAGCGTGGGGAGCAAACAGGATTA
GATACCCTGGTAGTCCACGCTGTAAACGATGTCGACTTGGAGGTTGTGCCCTTGAGGCG
TGGCTTCCGGAGCTAACGCGTTAAGTCGACCGCCTGGGGAGTACGGCCGCAAGGTTAA
AACTCAAATGAATTGACGGGGGCCCGCACAAGCGGTGGAGCATGTGGTTTAATTCGATG
CAACGCGAAGAACCTTACCTACTCTTGACATCCACGGAATTTAGCAGAGATGCTTTAGT
GCCTTCGGGAACCGTGAGACAGGTGCTGCATGGCTGTCGTCAGCTCGTGTTGTGAAATG
TTGGGTTAAGTCCCGCAACGAGCGCAACCCTTATCCTTTGTTGCCAGCACGTAATGGTG
GGAACTCAAGGGAGACTGCCGGTGACAAACCGGAGGAAGGTGGGGATGACGTCAAGT
CATCATGGCCCTTACGAGTAGGGCTACACACGTGCTACAATGGCAGATACAAAGTGAAG
CGAACTCGCGAGAGCAAGCGGACCACATAAAGTCTGTCGTAGTCCGGATTGGAGTCTG

CAACTCGACTCCATGAAGTCGGAATCGCTAGTAATCGTAGATCAGAATGCTACGGTGAAT
ACGTTCCCGGGCCTTGTACACACCGCCCGTCACACCATGGGAGTGGGTTGCAAAAGAA
GTAGGTAGCTTAACCTTCGGGAGGGCGCTTACCACTTTGTGATTCATGACTGGGG
pattern 215
CGCTGGCGGCAGGCCTAACACATGCAAGTCGAGCGGCAACGGGAAGTAGTTTACTACT
TTGCCGGCGAGCGGCGGACGGGTGAGTAATGTCTGGGAAACTGCCTGATGGAGGGGGA
TAACTACTGGAAACGGTAGCTAATACCGCATGACCTCGTAAGAGCAAAGTGGGGGACCT
TCGGGCCTCACGCCATCGGATGTGCCCAGATGGGATTAGCTAGTAGGTGGGGTAATGGC
TCACCTAGGCGACGATCCCTAGCTGGTCTGAGAGGATGACCAGCCACACTGGAACTGA
GACACGGTCCAGACTCCTACGGGAGGCAGCAGTGGGGAATATTGCACAATGGGCGCAA
GCCTGATGCAGCCATGCCGCGTGTGTGAAGAAGGCCTTCGGGTTGTAAAGCACTTTCAG
CGAGGAGGAAGGCAGTCGTGTTAATAGCACGGTTGATTGACGTTACTCGCAGAAGAAG
CACCGGCTAACTCCGTGCCAGCAGCCGCGGTAATACGGAGGGTGCAAGCGTTAATCGG
AATTACTGGGCGTAAAGCGCACGCAGGCGGTTTGTTAAGTCAGATGTGAAATCCCCGCG
CTTAACGTGGGAACTGCATTTGAAACTGGCAAGCTAGAGTCTTGTAGAGGGGGGTAGA
ATTCCAGGTGTAGCGGTGAAATGCGTAGAGATCTGGAGGAATACCGGTGGCGAAGGCG
GCCCCCTGGACAAAGACTGACGCTCAGGTGCGAAAGCGTGGGGAGCAAACAGGATTA
GATACCCTGGTAGTCCACGCTGTAAACGATGTCGACTTGGAGGTTGTGCCCTTGAGGCG
TGGCTTCCGGAGCTAACGCGTTAAGTCGACCGCCTGGGGAGTACGGCCGCAAGGTTAA
AACTCAAATGAATTGACGGGGGCCCGCACAAGCGGTGGAGCATGTGGTTTAATTCGATG
CAACGCGAAGAACCTTACCTACTCTTGACATCCACAGAACTTAGCAGAGATGCTTCGGT
GCCTTCGGGAACTGTGAGACAGGTGCTGCATGGCTGTCGTCAGCTCGTGTTGTGAAATG
TTGGGTTAAGTCCCGCAACGAGCGCAACCCTTATCCTTTGTTGCCAGCACGTAATGGTG
GGAACTCAAGGGAGACTGCCGGTGACAAACCGGAGGAAGGTGGGGATGACGTCAAGT
CATCATGGCCCTTACGAGTAGGGCTACACACGTGCTACAATGGCAGATACAAAGTGAAG
CGAACTCGCGAGAGCAAGCGGACCACATAAAGTCTGTCGTAGTCCGGATTGGAGTCTG
CAACTCGACTCCATGAAGTCGGAATCGCTAGTAATCGTAGATCAGAATGCTACGGTGAAT
ACGTTCCCGGGCCTTGTACACACCGCCCGTCACACCATGGGAGTGGGTTGCAAAAGAA
GTAGGTAGCTTAACCTTCGGGAGGGCGCTTACCACTTTGTGATTCATGACTGGGG
pattern 216
CGCTGGCGGCAGGCCTAACACATGCAAGTCGAGCGGCAACGGGAAGTAGTTTACTACT
TTGCCGGCGAGCGGCGGACGGGTGAGTAATGTCTGGGAAACTGCCTGATGGAGGGGGA
TAACTACTGGAAACGGTAGCTAATACCGCATGACCTCGTAAGAGCAAAGTGGGGGACCT
TCGGGCCTCACGCCATCGGATGTGCCCAGATGGGATTAGCTAGTAGGTGGGGTAATGGC
TCACCTAGGCGACGATCCCTAGCTGGTCTGAGAGGATGACCAGCCACACTGGAACTGA
GACACGGTCCAGACTCCTACGGGAGGCAGCAGTGGGGAATATTGCACAATGGGCGCAA
GCCTGATGCAGCCATGCCGCGTGTGTGAAGAAGGCCTTCGGGTTGTAAAGCACTTTCAG
CGAGGAGGAAGGCAGTCGTGTTAATAGCACGATTGATTGACGTTACTCGCAGAAGAAG
CACCGGCTAACTCCGTGCCAGCAGCCGCGGTAATACGGAGGGTGCAAGCGTTAATCGG
AATTACTGGGCGTAAAGCGCACGCAGGCGGTTTGTTAAGTCAGATGTGAAATCCCCGCG
CTTAACGTGGGAACTGCATTTGAAACTGGCAAGCTAGAGTCTTGTAGAGGGGGGTAGA
ATTCCAGGTGTAGCGGTGAAATGCGTAGAGATCTGGAGGAATACCGGTGGCGAAGGCG
GCCCCCTGGACAAAGACTGACGCTCAGGTGCGAAAGCGTGGGGAGCAAACAGGATTA
GATACCCTGGTAGTCCACGCTGTAAACGATGTCGACTTGGAGGTTGTGCCCTTGAGGCG

TGGCTTCCGGAGCTAACGCGTTAAGTCGACCGCCTGGGGAGTACGGCCGCAAGGTTAA
AACTCAAATGAATTGACGGGGGCCCGCACAAGCGGTGGAGCATGTGGTTTAATTCGATG
CAACGCGAAGAACCTTACCTACTCTTGACATCCACAGAACTTAGCAGAGATGCTTCGGT
GCCTTCGGGAACTGTGAGACAGGTGCTGCATGGCTGTCGTCAGCTCGTGTTGTGAAATG
TTGGGTTAAGTCCCGCAACGAGCGCAACCCTTATCCTTTGTTGCCAGCACGTAATGGTG
GGAACTCAAGGGAGACTGCCGGTGACAAACCGGAGGAAGGTGGGGATGACGTCAAGT
CATCATGGCCCTTACGAGTAGGGCTACACACGTGCTACAATGGCAGATACAAAGTGAAG
CGAACTCGCGAGAGCAAGCGGACCACATAAAGTCTGTCGTAGTCCGGATTGGAGTCTG
CAACTCGACTCCATGAAGTCGGAATCGCTAGTAATCGTAGATCAGAATGCTACGGTGAAT
ACGTTCCCGGGCCTTGTACACACCGCCCGTCACACCATGGGAGTGGGTTGCAAAAGAA
GTAGGTAGCTTAACCTTCGGGAGGGCGCTTACCACTTTGTGATTCATGACTGGGG
pattern 217
CGCTGGCGGCAGGCCTAACACATGCAAGTCGAGCGGCAACGGGAAGTAGTTTACTACT
TTGCCGGCGAGCGGCGGACGGGTGAGTAATGTCTGGGAAACTGCCTGATGGAGGGGGA
TAACTACTGGAAACGGTAGCTAATACCGCATGACCTCGTAAGAGCAAAGTGGGGGACCT
TCGGGCCTCACGCCATCGGATGTGCCCAGATGGGATTAGCTAGTAGGTGGGGTAATGGC
TCACCTAGGCGACGATCCCTAGCTGGTCTGAGAGGATGACCAGCCACACTGGAACTGA
GACACGGTCCAGACTCCTACGGGAGGCAGCAGTGGGGAATATTGCACAATGGGCGCAA
GCCTGATGCAGCCATGCCGCGTGTGTGAAGAAGGCCTTCGGGTTGTAAAGCACTTTCAG
CGAGGAGGAAGGCAGTCGTGTTAATAGCACGGTTGATTGACGTTACTCGCAGAAGAAG
CACCGGCTAACTCCGTGCCAGCAGCCGCGGTAATACGGAGGGTGCAAGCGTTAATCGG
AATTACTGGGCGTAAAGCGCACGCAGGCGGTTTGTTAAGTCAGATGTGAAATCCCCGCG
CTTAACGTGGGAACTGCATTTGAAACTGGCAAGCTAGAGTCTTGTAGAGGGGGGTAGA
ATTCCAGGTGTAGCGGTGAAATGCGTAGAGATCTGGAGGAATACCGGTGGCGAAGGCG
GCCCCCTGGACAAAGACTGACGCTCAGGTGCGAAAGCGTGGGGAGCAAACAGGATTA
GATACCCTGGTAGTCCACGCTGTAAACGATGTCGACTTGGAGGTTGTGCCCTTGAGGCG
TGGCTTCCGGAGCTAACGCGTTAAGTCGACCGCCTGGGGAGTACGGCCGCAAGGTTAA
AACTCAAATGAATTGACGGGGGCCCGCACAAGCGGTGGAGCATGTGGTTTAATTCGATG
CAACGCGAAGAACCTTACCTACTCTTGACATCCACAGAACTTAGCAGAGATGCTTCGGT
GCCTTCGGGAACTGTGAGACAGGTGCTGCATGGCTGTCGTCAGCTCGTGTTGTGAAATG
TTGGGTTAAGTCCCGCAACGAGCGCAACCCTTATCCTTTGTTGCCAGCACGTAATGGTG
GGAACTCAAGGGAGACTGCCGGTGACAAACCGGAGGAAGGTGGGGATGACGTCAAGT
CATCATGGCCCTTACGAGTAGGGCTACACACGTGCTACAATGGCAGATACAAAGTGAAG
CGAACTCGCGAGAGCAAGCGGACCACATAAAGTCTGTCGTAGTCCGGATTGGAGTCTG
CAACTCGACTCCATGAAGTCGGAATCGCTAGTAATCGTAGATCAGAATGCTACGGTGAAT
ATGTTCCCGGGCCTTGTACACACCGCCCGTCACACCATGGGAGTGGGTTGCAAAAGAA
GTAGGTAGCTTAACCTTCGGGAGGGCGCTTACCACTTTGTGATTCATGACTGGGG
pattern 218
CGCTGGCGGCAGGCCTAACACATGCAAGTCGAGCGGCAGCGGGAAGTAGTTTACTACT
TTGCCGGCGAGCGGCGGACGGGTGAGTAATGTCTGGGAAACTGCCTGATGGAGGGGGA
TAACTACTGGAAACGGTAGCTAATACCGCATGACCTCGTAAGAGCAAAGTGGGGGACCT
TCGGGCCTCACGCCATCGGATGTGCCCAGATGGGATTAGCTAGTAGGTGGGGTAATGGC
TCACCTAGGCGACGATCCCTAGCTGGTCTGAGAGGATGACCAGCCACACTGGAACTGA
GACACGGTCCAGACTCCTACGGGAGGCAGCAGTGGGGAATATTGCACAATGGGCGCAA

GCCTGATGCAGCCATGCCGCGTGTGTGAAGAAGGCCTTCGGGTTGTAAAGCACTTTCAG
CGAGGAGGAAGGCAGTCGTGTTAATAGCACGATTGATTGACGTTACTCGCAGAAGAAG
CACCGGCTAACTCCGTGCCAGCAGCCGCGGTAATACGGAGGGTGCAAGCGTTAATCGG
AATTACTGGGCGTAAAGCGCACGCAGGCGGTTTGTTAAGTCAGATGTGAAATCCCCGCG
CTTAACGTGGGAACTGCATTTGAAACTGGCAAGCTAGAGTCTTGTAGAGGGGGGTAGA
ATTCCAGGTGTAGCGGTGAAATGCGTAGAGATCTGGAGGAATACCGGTGGCGAAGGCG
GCCCCCTGGACAAAGACTGACGCTCAGGTGCGAAAGCGTGGGGAGCAAACAGGATTA
GATACCCTGGTAGTCCACGCTGTAAACGATGTCGACTTGGAGGTTGTGCCCTTGAGGCG
TGGCTTCCGGAGCTAACGCGTTAAGTCGACCGCCTGGGGAGTACGGCCGCAAGGTTAA
AACTCAAATGAATTGACGGGGGCCCGCACAAGCGGTGGAGCATGTGGTTTAATTCGATG
CAACGCGAAGAACCTTACCTACTCTTGACATCCACGGAATTTAGCAGAGATGCTTTAGT
GCCTTCGGGAACCGTGAGACAGGTGCTGCATGGCTGTCGTCAGCTCGTGTTGTGAAATG
TTGGGTTAAGTCCCGCAACGAGCGCAACCCTTATCCTTTGTTGCCAGCACGTAATGGTG
GGAACTCAAGGGAGACTGCCGGTGACAAACCGGAGGAAGGTGGGGATGACGTCAAGT
CATCATGGCCCTTACGAGTAGGGCTACACACGTGCTACAATGGCAAATACAAAGTGAAG
CGAACTCGCGAGAGCAAGCGGACCACATAAAGTCTGTCGTAGTCCGGATTGGAGTCTG
CAACTCGACTCCATGAAGTCGGAATCGCTAGTAATCGTAGATCAGAATGCTACGGTGAAT
ACGTTCCCGGGCCTTGTACACACCGCCCGTCACACCATGGGAGTGGGTTGCAAAAGAA
GTAGGTAGCTTAACCTTCGGGAGGGCGCTTACCACTTTGTGATTCATGACTGGGG
pattern 219
CGCTGGCGGCAGGCCTAACACATGCAAGTCGAGCGGCAGCGGGAAGTAGTTTACTACT
TTGCCGGCGAGCGGCGGACGGGTGAGTAATGTCTGGGAAACTGCCTGATGGAGGGGGA
TAACTACTGGAAACGGTAGCTAATACCGCATGACCTCGTAAGAGCAAAGTGGGGGACCT
TCGGGCCTCACGCCATCGGATGTGCCCAGATGGGATTAGCTAGTAGGTGGGGTAATGGC
TCACCTAGGCGACGATCCCTAGCTGGTCTGAGAGGATGACCAGCCACACTGGAACTGA
GACACGGTCCAGACTCCTACGGGAGGCAGCAGTGGGGAATATTGCACAATGGGCGCAA
GCCTGATGCAGCCATGCCGCGTGTGTGAAGAAGGCCTTCGGGTTGTAAAGCACTTTCAG
CGAGGAGGAAGGCAGTCGTGTTAATAGCACGATTGATTGACGTTACTCGCAGAAGAAG
CACCGGCTAACTCCGTGCCAGCAGCCGCGGTAATACGGAGGGTGCAAGCGTTAATCGG
AATTACTGGGCGTAAAGCGCACGCAGGCGGTTTGTTAAGTCAGATGTGAAATCCCCGCG
CTTAACGTGGGAACTGCATTTGAAACTGGCAAGCTAGAGTCTTGTAGAGGGGGGTAGA
ATTCCAGGTGTAGCGGTGAAATGCGTAGAGATCTGGAGGAATACCGGTGGCGAAGGCG
GCCCCCTGGACAAAGACTGACGCTCAGGTGCGAAAGCGTGGGGAGCAAACAGGATTA
GATACCCTGGTAGTCCACGCTGTAAACGATGTCGACTTGGAGGTTGTGCCCTTGAGGCG
TGGCTTCCGGAGCTAACGCGTTAAGTCGACCGCCTGGGGAGTACGGCCGCAAGGTTAA
AACTCAAATGAATTGACGGGGGCCCGCACAAGCGGTGGAGCATGTGGTTTAATTCGATG
CAACGCGAAGAACCTTACCTACTCTTGACATCCACAGAACTTAGCAGAGATGCTTCGGT
GCCTTCGGGAACTGTGAGACAGGTGCTGCATGGCTGTCGTCAGCTCGTGTTGTGAAATG
TTGGGTTAAGTCCCGCAACGAGCGCAACCCTTATCCTTTGTTGCCAGCACGTAATGGTG
GGAACTCAAGGGAGACTGCCGGTGACAAACCGGAGGAAGGTGGGGATGACGTCAAGT
CATCATGGCCCTTACGAGTAGGGCTACACACGTGCTACAATGGCAAATACAAAGTGAAG
CGAACTCGCGAGAGCAAGCGGACCACATAAAGTCTGTCGTAGTCCGGATTGGAGTCTG
CAACTCGACTCCATGAAGTCGGAATCGCTAGTAATCGTAGATCAGAATGCTACGGTGAAT
ACGTTCCCGGGCCTTGTACACACCGCCCGTCACACCATGGGAGTGGGTTGCAAAAGAA

GTAGGTAGCTTAACCTTCGGGAGGGCGCTTACCACTTTGTGATTCATGACTGGGG
pattern 220
CGCTGGCGGCAGGCCTAACACATGCAAGTCGAGCGGCAGCGGAAAGTAGCTTGCTACT
TTGCCGGCGAGCGGCGGACGGGTGAGTAATGTCTGGGAAACTGCCTGATGGAGGGGGA
TAACTACTGGAAACGGTAGCTAATACCGCATGACCTCGAAAGAGCAAAGTGGGGGACC
TTCGGGCCTCACGCCATCGGATGTGCCCAGATGGGATTAGCTAGTAGGTGGGGTAATGG
CTCACCTAGGCGACGATCCCTAGCTGGTCTGAGAGGATGACCAGCCACACTGGAACTG
AGACACGGTCCAGACTCCTACGGGAGGCAGCAGTGGGGAATATTGCACAATGGGCGCA
AGCCTGATGCAGCCATGCCGCGTGTGTGAAGAAGGCCTTCGGGTTGTAAAGCACTTTCA
GCGAGGAGGAAGGCATTTCACTTAATACGTGAGGTGATTGACGTTACTCGCAGAAGAA
GCACCGGCTAACTCCGTGCCAGCAGCCGCGGTAATACGGAGGGTGCAAGCGTTAATCG
GAATTACTGGGCGTAAAGCGCACGCAGGCGGTTTGTTAAGTCAGATGTGAAATCCCCGA
GCTTAACTTGGGAACTGCATTTGAAACTGGCAAGCTAGAGTCTTGTAGAGGGGGGTAG
AATTCCAGGTGTAGCGGTGAAATGCGTAGAGATCTGGAGGAATACCGGTGGCGAAGGC
GGCCCCCTGGACAAAGACTGACGCTCAGGTGCGAAAGCGTGGGGAGCAAACAGGATT
AGATACCCTGGTAGTCCACGCTGTAAACGATGTCGACTTGGAGGTTGTGCCCTTGAGGC
GTGGCTTCCGGAGCTAACGCGTTAAGTCGACCGCCTGGGGAGTACGGCCGCAAGGTTA
AAACTCAAATGAATTGACGGGGGCCCGCACAAGCGGTGGAGCATGTGGTTTAATTCGAT
GCAACGCGAAGAACCTTACCTACTCTTGACATCCACAGAACTGAGCAGAGATGCTTAG
GTGCCTTCGGGAACTGTGAGACAGGTGCTGCATGGCTGTCGTCAGCTCGTGTTGTGAAA
TGTTGGGTTAAGTCCCGCAACGAGCGCAACCCTTATCCTTTGTTGCCAGCACGTGATGG
TGGGAACTCAAAGGAGACTGCCGGTGATAAACCGGAGGAAGGTGGGGATGACGTCAA
GTCATCATGGCCCTTACGAGTAGGGCTACACACGTGCTACAATGGCAGATACAAAGTGA
AGCGAACTCGCGAGAGCAAGCGGACCACATAAAGTCTGTCGTAGTCCGGATTGGAGTC
TGCAACTCGACTCCATGAAGTCGGAATCGCTAGTAATCGTAGATCAGAATGCTACGGTG
AATACGTTCCCGGGCCTTGTACACACCGCCCGTCACACCATGGGAGTGGGTTGCAAAA
GAAGTAGGTAGCTTAACCTTCGGGAGGGCGCTTACCACTTTGTGATTCATGACTGGGG
pattern 221
CGCTGGCGGCAGGCCTAACACATGCAAGTCGAGCGGCAGCGGAAAGTAGCTTGCTACT
TTGCCGGCGAGCGGCGGACGGGTGAGTAATGTCTGGGAAACTGCCTGATGGAGGGGGA
TAACTACTGGAAACGGTAGCTAATACCGCATGACCTCGAAAGAGCAAAGTGGGGGACC
TTCGGGCCTCACGCCATCGGATGTGCCCAGATGGGATTAGCTAGTAGGTGGGGTAATGG
CTCACCTAGGCGACGATCCCTAGCTGGTCTGAGAGGATGACCAGCCACACTGGAACTG
AGACACGGTCCAGACTCCTACGGGAGGCAGCAGTGGGGAATATTGCACAATGGGCGCA
AGCCTGATGCAGCCATGCCGCGTGTGTGAAGAAGGCCTTCGGGTTGTAAAGCACTTTCA
GCGAGGAGGAAGGCATTTCACTTAATACGTGAAGTGATTGACGTTACTCGCAGAAGAA
GCACCGGCTAACTCCGTGCCAGCAGCCGCGGTAATACGGAGGGTGCAAGCGTTAATCG
GAATTACTGGGCGTAAAGCGCACGCAGGCGGTTTGTTAAGTCAGATGTGAAATCCCCGA
GCTTAACTTGGGAACTGTATTTGAAACTGGCAAGCTAGAGTCTTGTAGAGGGGGGTAGA
ATTCCAGGTGTAGCGGTGAAATGCGTAGAGATCTGGAGGAATACCGGTGGCGAAGGCG
GCCCCCTGGACAAAGACTGACGCTCAGGTGCGAAAGCGTGGGGAGCAAACAGGATTA
GATACCCTGGTAGTCCACGCTGTAAACGATGTCGACTTGGAGGTTGTGCCCTTGAGGCG
TGGCTTCCGGAGCTAACGCGTTAAGTCGACCGCCTGGGGAGTACGGCCGCAAGGTTAA
AACTCAAATGAATTGACGGGGGCCCGCACAAGCGGTGGAGCATGTGGTTTAATTCGATG

CAACGCGAAGAACCTTACCTACTCTTGACATCCACAGAACTGAGCAGAGATGCTTAGGT
GCCTTCGGGAACTGTGAGACAGGTGCTGCATGGCTGTCGTCAGCTCGTGTTGTGAAATG
TTGGGTTAAGTCCCGCAACGAGCGCAACCCTTATCCTTTGTTGCCAGCACGTGATGGTG
GGAACTCAAAGGAGACTGCCGGTGATAAACCGGAGGAAGGTGGGGATGACGTCAAGT
CATCATGGCCCTTACGAGTAGGGCTACACACGTGCTACAATGGCAGATACAAAGTGAAG
CGAACTCGCGAGAGCAAGCGGACCACATAAAGTCTGTCGTAGTCCGGATTGGAGTCTG
CAACTCGACTCCATGAAGTCGGAATCGCTAGTAATCGTAGATCAGAATGCTACGGTGAAT
ACGTTCCCGGGCCTTGTACACACCGCCCGTCACACCATGGGAGTGGGTTGCAAAAGAA
GTAGGTAGCTTAACCTTCGGGAGGGCGCTTACCACTTTGTGATTCATGACTGGGG
pattern 222
CGCTGGCGGCAGGCCTAACACATGCAAGTCGAGCGGCAGCGGAAAGTAGCTTGCTACT
TTGCCGGCGAGCGGCGGACGGGTGAGTAATGTCTGGGAAACTGCCTGATGGAGGGGGA
TAACTACTGGAAACGGTAGCTAATACCGCATGACCTCGAAAGAGCAAAGTGGGGGACC
TTCGGGCCTCACGCCATCGGATGTGCCCAGATGGGATTAGCTAGTAGGTGAGGTAATGG
CTCACCTAGGCGACGATCCCTAGCTGGTCTGAGAGGATGACCAGCCACACTGGAACTG
AGACACGGTCCAGACTCCTACGGGAGGCAGCAGTGGGGAATATTGCACAATGGGCGCA
AGCCTGATGCAGCCATGCCGCGTGTGTGAAGAAGGCCTTCGGGTTGTAAAGCACTTTCA
GCGAGGAGGAAGGCATTTCACTTAATACGTGAGGTGATTGACGTTACTCGCAGAAGAA
GCACCGGCTAACTCCGTGCCAGCAGCCGCGGTAATACGGAGGGTGCAAGCGTTAATCG
GAATTACTGGGCGTAAAGCGCACGCAGGCGGTTTGTTAAGTCAGATGTGAAATCCCCGA
GCTTAACTTGGGAACTGCATTTGAAACTGGCAAGCTAGAGTCTTGTAGAGGGGGGTAG
AATTCCAGGTGTAGCGGTGAAATGCGTAGAGATCTGGAGGAATACCGGTGGCGAAGGC
GGCCCCCTGGACAAAGACTGACGCTCAGGTGCGAAAGCGTGGGGAGCAAACAGGATT
AGATACCCTGGTAGTCCACGCTGTAAACGATGTCGACTTGGAGGTTGTGCCCTTGAGGC
GTGGCTTCCGGAGCTAACGCGTTAAGTCGACCGCCTGGGGAGTACGGCCGCAAGGTTA
AAACTCAAATGAATTGACGGGGGCCCGCACAAGCGGTGGAGCATGTGGTTTAATTCGAT
GCAACGCGAAGAACCTTACCTACTCTTGACATCCACAGAACTTAGCAGAGATGCTTAGG
TGCCTTCGGGAACTGTGAGACAGGTGCTGCATGGCTGTCGTCAGCTCGTGTTGTGAAAT
GTTGGGTTAAGTCCCGCAACGAGCGCAACCCTTATCCTTTGTTGCCAGCACGTAATGGT
GGGAACTCAAAGGAGACTGCCGGTGATAAACCGGAGGAAGGTGGGGATGACGTCAAG
TCATCATGGCCCTTACGAGTAGGGCTACACACGTGCTACAATGGCAGATACAAAGTGAA
GCGAACTCGCGAGAGCAAGCGGACCACATAAAGTCTGTCGTAGTCCGGATTGGAGTCT
GCAACTCGACTCCATGAAGTCGGAATCGCTAGTAATCGTAGATCAGAATGCTACGGTGA
ATACGTTCCCGGGCCTTGTACACACCGCCCGTCACACCATGGGAGTGGGTTGCAAAAG
AAGTAGGTAGCTTAACCTTCGGGAGGGCGCTTACCACTTTGTGATTCATGACTGGGG
pattern 223
CGCTGGCGGCAGGCCTAACACATGCAAGTCGAGCGGCAGCGGGAAGTAGCTTGCTACT
TTGCCGGCGAGCGGCGGACGGGTGAGTAATGTCTGGGAAACTGCCTGATGGAGGGGGA
TAACTACTGGAAACGGTAGCTAATACCGCATGACCTCGAAAGAGCAAAGTGGGGGACC
TTCGGGCCTCACGCCATCGGATGTGCCCAGATGGGATTAGCTAGTAGGTGGGGTAATGG
CTCACCTAGGCGACGATCCCTAGCTGGTCTGAGAGGATGACCAGCCACACTGGAACTG
AGACACGGTCCAGACTCCTACGGGAGGCAGCAGTGGGGAATATTGCACAATGGGCGCA
AGCCTGATGCAGCCATGCCGCGTGTGTGAAGAAGGCCTTCGGGTTGTAAAGCACTTTCA
GCGAGGAGGAAGGCATCTTACTTAATACGTGAGGTGATTGACGTTACTCGCAGAAGAA

GCACCGGCTAACTCCGTGCCAGCAGCCGCGGTAATACGGAGGGTGCAAGCGTTAATCG
GAATTACTGGGCGTAAAGCGCACGCAGGCGGTTTGTTAAGTCAGATGTGAAATCCCCGA
GCTTAACTTGGGAACTGCATTTGAAACTGGCAAGCTAGAGTCTTGTAGAGGGGGGTAG
AATTCCAGGTGTAGCGGTGAAATGCGTAGAGATCTGGAGGAATACCGGTGGCGAAGGC
GGCCCCCTGGACAAAGACTGACGCTCAGGTGCGAAAGCGTGGGGAGCAAACAGGATT
AGATACCCTGGTAGTCCACGCTGTAAACGATGTCGACTTGGAGGTTGTGCCCTTGAGGC
GTGGCTTCCGGAGCTAACGCGTTAAGTCGACCGCCTGGGGAGTACGGCCGCAAGGTTA
AAACTCAAATGAATTGACGGGGGCCCGCACAAGCGGTGGAGCATGTGGTTTAATTCGAT
GCAACGCGAAGAACCTTACCTACTCTTGACATCCACAGAACTTAGCAGAGATGCTTAGG
TGCCTTCGGGAACTGTGAGACAGGTGCTGCATGGCTGTCGTCAGCTCGTGTTGTGAAAT
GTTGGGTTAAGTCCCGCAACGAGCGCAACCCTTATCCTTTGTTGCCAGCACGTAATGGT
GGGAACTCAAAGGAGACTGCCGGTGATAAACCGGAGGAAGGTGGGGATGACGTCAAG
TCATCATGGCCCTTACGAGTAGGGCTACACACGTGCTACAATGGCAGATACAAAGTGAA
GCGAACTCGCGAGAGCAAGCGGACCACATAAAGTCTGTCGTAGTCCGGATTGGAGTCT
GCAACTCGACTCCATGAAGTCGGAATCGCTAGTAATCGTAGATCAGAATGCTACGGTGA
ATACGTTCCCGGGCCTTGTACACACCGCCCGTCACACCATGGGAGTGGGTTGCAAAAG
AAGTAGGTAGCTTAACCTTCGGGAGGGCGCTTACCACTTTGTGATTCATGACTGGGG
pattern 224
CGCTGGCGGCAGGCCTAACACATGCAAGTCGAGCGGCAGCGGAAAGTAGCTTGCTACT
TTGCCGGCGAGCGGCGGACGGGTGAGTAATGTCTGGGGATCTGCCTGATGGAGGGGGA
TAACTACTGGAAACGGTAGCTAATACCGCATGACCTCGAAAGAGCAAAGTGGGGGACC
TTCGGGCCTCACGCCATCGGATGAACCCAGATGGGATTAGCTAGTAGGTGGGGTAATGG
CTCACCTAGGCGACGATCCCTAGCTGGTCTGAGAGGATGACCAGCCACACTGGAACTG
AGACACGGTCCAGACTCCTACGGGAGGCAGCAGTGGGGAATATTGCACAATGGGCGCA
AGCCTGATGCAGCCATGCCGCGTGTGTGAAGAAGGCCTTCGGGTTGTAAAGCACTTTCA
GCGAGGAGGAAGGCATTGTGGTTAATAACCGCAGTGATTGACGTTACTCGCAGAAGAA
GCACCGGCTAACTCCGTGCCAGCAGCCGCGGTAATACGGAGGGTGCAAGCGTTAATCG
GAATTACTGGGCGTAAAGCGCACGCAGGCGGTTTGTTAAGTCAGATGTGAAATCCCCGC
GCTTAACGTGGGAACTGCATTTGAAACTGGCAAGCTAGAGTCTTGTAGAGGGGGGTAG
AATTCCAGGTGTAGCGGTGAAATGCGTAGAGATCTGGAGGAATACCGGTGGCGAAGGC
GGCCCCCTGGACAAAGACTGACGCTCAGGTGCGAAAGCGTGGGGAGCAAACAGGATT
AGATACCCTGGTAGTCCACGCTGTAAACGATGTCGACTTGGAGGTTGTGCCCTTGAGGT
GTGGCTTCCGGAGCTAACGCGTTAAGTCGACCGCCTGGGGAGTACGGCCGCAAGGTTA
AAACTCAAATGAATTGACGGGGGCCCGCACAAGCGGTGGAGCATGTGGTTTAATTCGAT
GCAACGCGAAGAACCTTACCTACTCTTGACATCCACGGAATTTAGCAGAGATGCTTCGG
TGCCTTCGGGAACTGTGAGACAGGTGCTGCATGGCTGTCGTCAGCTCGTGTTGTGAAAT
GTTGGGTTAAGTCCCGCAACGAGCGCAACCCTTATCCTTTGTTGCCAGCACGTAATGGT
GGGAACTCAAGGGAGACTGCCGGTGACAAACCGGAGGAAGGTGGGGATGACGTCAAG
TCATCATGGCCCTTACGAGTAGGGCTACACACGTGCTACAATGGCAGATACAAAGTGAA
GCGAACTCGCGAGAGCAAGCGGACCACATAAAGTCTGTCGTAGTCCGGATTGGAGTCT
GCAACTCGACTCCATGAAGTCGGAATCGCTAGTAATCGTAGATCAGAATGCTACGGTGA
ATACGTTCCCGGGCCTTGTACACACCGCCCGTCACACCATGGGAGTGGGTTGCAAAAG
AAGTAGGTAGCTTAACCTTCGGGAGGGCGCTTACCACTTTGTGATTCATGACTGGGG
pattern 225

CGCTGGCGGCAGGCCTAACACATGCAAGTCGAGCGGCAGCGGGAAGTAGCTTGCTACT
TTGCCGGCGAGCGGCGGACGGGTGAGTAATGTCTGGGAAACTGCCTGATGGAGGGGGA
TAACTACTGGAAACGGTAGCTAATACCGCATGACCTCGAAAGAGCAAAGTGGGGGACC
TTCGGGCCTCACGCCATCGGATGTGCCCAGATGGGATTAGCTAGTAGGTGGGGTAATGG
CTCACCTAGGCGACGATCCCTAGCTGGTCTGAGAGGATGACCAGCCACACTGGAACTG
AGACACGGTCCAGACTCCTACGGGAGGCAGCAGTGGGGAATATTGCACAATGGGCGCA
AGCCTGATGCAGCCATGCCGCGTGTGTGAAGAAGGCCTTCGGGTTGTAAAGCACTTTCA
GCGAGGAGGAAGGCATTTCACTTAATACGTGGGGTGATTGACGTTACTCGCAGAAGAA
GCACCGGCTAACTCCGTGCCAGCAGCCGCGGTAATACGGAGGGTGCAAGCGTTAATCG
GAATTACTGGGCGTAAAGCGCACGCAGGCGGTTTGTTAAGTCAGATGTGAAATCCCCGA
GCTTAACTTGGGAACTGCATTTGAAACTGGCAAGCTAGAGTCTTGTAGAGGGGGGTAG
AATTCCAGGTGTAGCGGTGAAATGCGTAGAGATCTGGAGGAATACCGGTGGCGAAGGC
GGCCCCCTGGACAAAGACTGACGCTCAGGTGCGAAAGCGTGGGGAGCAAACAGGATT
AGATACCCTGGTAGTCCACGCTGTAAACGATGTCGACTTGGAGGTTGTGCCCTTGAGGC
GTGGCTTCCGGAGCTAACGCGTTAAGTCGACCGCCTGGGGAGTACGGCCGCAAGGTTA
AAACTCAAATGAATTGACGGGGGCCCGCACAAGCGGTGGAGCATGTGGTTTAATTCGAT
GCAACGCGAAGAACCTTACCTACTCTTGACATCCACAGAACTTAGCAGAGATGCTTAGG
TGCCTTCGGGAACTGTGAGACAGGTGCTGCATGGCTGTCGTCAGCTCGTGTTGTGAAAT
GTTGGGTTAAGTCCCGCAACGAGCGCAACCCTTATCCTTTGTTGCCAGCACGTAATGGT
GGGAACTCAAAGGAGACTGCCGGTGATAAACCGGAGGAAGGTGGGGATGACGTCAAG
TCATCATGGCCCTTACGAGTAGGGCTACACACGTGCTACAATGGCAGATACAAAGTGAA
GCGAACTCGCGAGAGCAAGCGGACCACATAAAGTCTGTCGTAGTCCGGATTGGAGTCT
GCAACTCGACTCCATGAAGTCGGAATCGCTAGTAATCGTAGATCAGAATGCTACGGTGA
ATACGTTCCCGGGCCTTGTACACACCGCCCGTCACACCATGGGAGTGGGTTGCAAAAG
AAGTAGGTAGCTTAACCTTCGGGAGGGCGCTTACCACTTTGTGATTCATGACTGGGG
pattern 226
CGCTGGCGGCAGGCCTAACACATGCAAGTCGAGCGGCAGCGGGAAGTAGTTTACTACT
TTGCCGGCGAGCGGCGGACGGGTGAGTAATGTCTGGGGATCTGCCTGATGGAGGGGGA
TAACTACTGGAAACGGTAGCTAATACCGCATGACCTCGCAAGAGCAAAGTGGGGGACC
TTAGGGCCTCACGCCATCGGATGAACCCAGATGGGATTAGCTAGTAGGTGGGGTAATGG
CTCACCTAGGCGACGATCCCTAGCTGGTCTGAGAGGATGACCAGCCACACTGGAACTG
AGACACGGTCCAGACTCCTACGGGAGGCAGCAGTGGGGAATATTGCACAATGGGCGCA
AGCCTGATGCAGCCATGCCGCGTGTGTGAAGAAGGCCTTCGGGTTGTAAAGCACTTTCA
GCGAGGAGGAAGGGGTTGAGTTTAATACGCTCAATCATTGACGTTACTCGCAGAAGAA
GCACCGGCTAACTCCGTGCCAGCAGCCGCGGTAATACGGAGGGTGCAAGCGTTAATCG
GAATTACTGGGCGTAAAGCGCACGCAGGCGGTTTGTTAAGTCAGATGTGAAATCCCCGC
GCTTAACGTGGGAACTGCATTTGAAACTGGCAAGCTAGAGTCTTGTAGAGGGGGGTAG
AATTCCAGGTGTAGCGGTGAAATGCGTAGAGATCTGGAGGAATACCGGTGGCGAAGGC
GGCCCCCTGGACAAAGACTGACGCTCAGGTGCGAAAGCGTGGGGAGCAAACAGGATT
AGATACCCTGGTAGTCCACGCTGTAAACGATGTCGACTTGGAGGTTGTGCCCTTGAGGT
GTGGCTTCCGGAGCTAACGCGTTAAGTCGACCGCCTGGGGAGTACGGCCGCAAGGTTA
AAACTCAAATGAATTGACGGGGGCCCGCACAAGCGGTGGAGCATGTGGTTTAATTCGAT
GCAACGCGAAGAACCTTACCTACTCTTGACATCCACAGAATTTGGCAGAGATGCTAAAG
TGCCTTCGGGAACTGTGAGACAGGTGCTGCATGGCTGTCGTCAGCTCGTGTTGTGAAAT

GTTGGGTTAAGTCCCGCAACGAGCGCAACCCTTATCCTTTGTTGCCAGCACGTAATGGT
GGGAACTCAAGGGAGACTGCCGGTGACAAACCGGAGGAAGGTGGGGATGACGTCAAG
TCATCATGGCCCTTACGAGTAGGGCTACACACGTGCTACAATGGCAGATACAAAGTGAA
GCGAACTCGCGAGAGCCAGCGGACCACATAAAGTCTGTCGTAGTCCGGATTGGAGTCT
GCAACTCGACTCCATGAAGTCGGAATCGCTAGTAATCGTAGATCAGAATGCTACGGTGA
ATACGTTCCCGGGCCTTGTACACACCGCCCGTCACACCATGGGAGTGGGTTGCAAAAG
AAGTAGGTAGCTTAACCTTCGGGAGGGCGCTTACCACTTTGTGATTCATGACTGGGG
pattern 227
CGCTGGCGGCAGGCCTAACACATGCAAGTCGAGCGGCAGCGGGAAGTAGTTTACTACT
TTGCCGGCGAGCGGCGGACGGGTGAGTAATGTCTGGGGATCTGCCTGATGGAGGGGGA
TAACTACTGGAAACGGTAGCTAATACCGCATAACCTCGCAAGAGCAAAGTGGGGGACC
TTAGGGCCTCACGCCATCGGATGAACCCAGATGGGATTAGCTAGTAGGTGGGGTAATGG
CTCACCTAGGCGACGATCCCTAGCTGGTCTGAGAGGATGACCAGCCACACTGGAACTG
AGACACGGTCCAGACTCCTACGGGAGGCAGCAGTGGGGAATATTGCACAATGGGCGCA
AGCCTGATGCAGCCATGCCGCGTGTGTGAAGAAGGCCTTCGGGTTGTAAAGCACTTTCA
GCGAGGAGGAAGGGGTTGAGTTTAATACGCTCAATCATTGACGTTACTCGCAGAAGAA
GCACCGGCTAACTCCGTGCCAGCAGCCGCGGTAATACGGAGGGTGCAAGCGTTAATCG
GAATTACTGGGCGTAAAGCGCACGCAGGCGGTTTGTTAAGTCAGATGTGAAATCCCCGC
GCTTAACGTGGGAACTGCATTTGAAACTGGCAAGCTAGAGTCTTGTAGAGGGGGGTAG
AATTCCAGGTGTAGCGGTGAAATGCGTAGAGATCTGGAGGAATACCGGTGGCGAAGGC
GGCCCCCTGGACAAAGACTGACGCTCAGGTGCGAAAGCGTGGGGAGCAAACAGGATT
AGATACCCTGGTAGTCCACGCTGTAAACGATGTCGACTTGGAGGTTGTGCCCTTGAGGC
GTGGCTTCCGGAGCTAACGCGTTAAGTCGACCGCCTGGGGAGTACGGCCGCAAGGTTA
AAACTCAAATGAATTGACGGGGGCCCGCACAAGCGGTGGAGCATGTGGTTTAATTCGAT
GCAACGCGAAGAACCTTACCTACTCTTGACATCCACAGAATTTGGCAGAGATGCTAAAG
TGCCTTCGGGAACTGTGAGACAGGTGCTGCATGGCTGTCGTCAGCTCGTGTTGTGAAAT
GTTGGGTTAAGTCCCGCAACGAGCGCAACCCTTATCCTTTGTTGCCAGCACGTAATGGT
GGGAACTCAAGGGAGACTGCCGGTGACAAACCGGAGGAAGGTGGGGATGACGTCAAG
TCATCATGGCCCTTACGAGTAGGGCTACACACGTGCTACAATGGCAGATACAAAGTGAA
GCGAACTCGCGAGAGCCAGCGGACCACATAAAGTCTGTCGTAGTCCGGATTGGAGTCT
GCAACTCGACTCCATGAAGTCGGAATCGCTAGTAATCGTAGATCAGAATGCTACGGTGA
ATACGTTCCCGGGCCTTGTACACACCGCCCGTCACACCATGGGAGTGGGTTGCAAAAG
AAGTAGGTAGCTTAACCTTCGGGAGGGCGCTTACCACTTTGTGATTCATGACTGGGG
pattern 228
CGCTGGCGGCAGGCCTAACACATGCAAGTCGAGCGGCAGCGGGAAGTAGTTTACTACT
TTGCCGGCGAGCGGCGGACGGGTGAGTAATGTCTGGGGATCTGCCTGATGGAGGGGGA
TAACTACTGGAAACGGTAGCTAATACCGCATGACCTCGCGAGAGCAAAGTGGGGGACC
TTAGGGCCTCACGCCATCGGATGAACCCAGATGGGATTAGCTAGTAGGTGGGGTAATGG
CTCACCTAGGCGACGATCCCTAGCTGGTCTGAGAGGATGACCAGCCACACTGGAACTG
AGACACGGTCCAGACTCCTACGGGAGGCAGCAGTGGGGAATATTGCACAATGGGCGCA
AGCCTGATGCAGCCATGCCGCGTGTGTGAAGAAGGCCTTCGGGTTGTAAAGCACTTTCA
GCGAGGAGGAAGGGGTTGAGTTTAATACGCTCAATCATTGACGTTACTCGCAGAAGAA
GCACCGGCTAACTCCGTGCCAGCAGCCGCGGTAATACGGAGGGTGCAAGCGTTAATCG
GAATTACTGGGCGTAAAGCGCACGCAGGCGGTTTGTTAAGTCAGATGTGAAATCCCCGC

GCTTAACGTGGGAACTGCATTTGAAACTGGCAAGCTAGAGTCTTGTAGAGGGGGGTAG
AATTCCAGGTGTAGCGGTGAAATGCGTAGAGATCTGGAGGAATACCGGTGGCGAAGGC
GGCCCCCTGGACAAAGACTGACGCTCAGGTGCGAAAGCGTGGGGAGCAAACAGGATT
AGATACCCTGGTAGTCCACGCTGTAAACGATGTCGACTTGGAGGTTGTGCCCTTGAGGC
GTGGCTTCCGGAGCTAACGCGTTAAGTCGACCGCCTGGGGAGTACGGCCGCAAGGTTA
AAACTCAAATGAATTGACGGGGGCCCGCACAAGCGGTGGAGCATGTGGTTTAATTCGAT
GCAGCGCGAAGAACCTTACCTACTCTTGACATCCACAGAATTTGGCAGAGATGCTAAAG
TGCCTTCGGGAACTGTGAGACAGGTGCTGCATGGCTGTCGTCAGCTCGTGTTGTGAAAT
GTTGGGTTAAGTCCCGCAACGAGCGCAACCCTTATCCTTTGTTGCCAGCACGTAATGGT
GGGAACTCAAGGGAGACTGCCGGTGACAAACCGGAGGAAGGTGGGGATGACGTCAAG
TCATCATGGCCCTTACGAGTAGGGCTACACACGTGCTACAATGGCAGATACAAAGTGAA
GCGAACTCGCGAGAGCCAGCGGACCACATAAAGTCTGTCGTAGTCCGGATTGGAGTCT
GCAACTCGACTCCATGAAGTCGGAATCGCTAGTAATCGTAGATCAGAATGCTACGGTGA
ATACGTTCCCGGGCCTTGTACACACCGCCCGTCACACCATGGGAGTGGGTTGCAAAAG
AAGTAGGTAGCTTAACCTTCGGGAGGGCGCTTACCACTTTGTGATTCATGACTGGGG
pattern 229
CGCTGGCGGCAGGCCTAACACATGCAAGTCGAGCGGCAGCGGGAAGTAGTTTACTACT
TTGCCGGCGAGCGGCGGACGGGTGAGTAATGTCTGGGGATCTGCCTGATGGAGGGGGA
TAACTACTGGAAACGGTAGCTAATACCGCATGACCTCGCGAGAGCAAAGTGGGGGACC
TTAGGGCCTCACGCCATCGGATGAACCCAGATGGGATTAGCTAGTAGGTGGGGTAATGG
CTCACCTAGGCGACGATCCCTAGCTGGTCTGAGAGGATGACCAGCCACACTGGAACTG
AGACACGGTCCAGACTCCTACGGGAGGCAGCAGTGGGGAATATTGCACAATGGGCGCA
AGCCTGATGCAGCCATGCCGCGTGTGTGAAGAAGGCCTTCGGGTTGTAAAGCACTTTCA
GCGAGGAGGAAGGGGTTGAGTTTAATACGCTCAATCATTGACGTTACTCGCAGAAGAA
GCACCGGCTAACTCCGTGCCAGCAGCCGCGGTAATACGGAGGGTGCAAGCGTTAATCG
GAATTACTGGGCGTAAAGCGCACGCAGGCGGTTTGTTAAGTCAGATGTGAAATCCCCGC
GCTTAACGTGGGAACTGCATTTGAAACTGGCAAGCTAGAGTCTTGTAGAGGGGGGTAG
AATTCCAGGTGTAGCGGTGAAATGCGTAGAGATCTGGAGGAATACCGGTGGCGAAGGC
GGCCCCCTGGACAAAGACTGACGCTCAGGTGCGAAAGCGTGGGGAGCAAACAGGATT
AGATACCCTGGTAGTCCACGCTGTAAACGATGTCGACTTGGAGGTTGTGCCCTTGAGGC
GTGGCTTCCGGAGCTAACGCGTTAAGTCGACCGCCTGGGGAGTACGGCCGCAAGGTTA
AAACTCAAATGAATTGACGGGGGCCCGCACAAGCGGTGGAGCATGTGGTTTAATTCGAT
GCAACGCGAAGAACCTTACCTACTCTTGACATCCACAGAATTTGGCAGAGATGCTAAAG
TGCCTTCGGGAACTGTGAGACAGGTGCTGCATGGCTGTCGTCAGCTCGTGTTGTGAAAT
GTTGGGTTAAGTCCCGCAACGAGCGCAACCCTTATCCTTTGTTGCCAGCACGTAATGGT
GGGAACTCAAGGGAGACTGCCGGTGACAAACCGGAGGAAGGTGGGGATGACGTCAAG
TCATCATGGCCCTTACGAGTAGGGCTACACACGTGCTACAATGGCAGATACAAAGTGAA
GCGAACTCGCGAGAGCCAGCGGACCACATAAAGTCTGTCGTAGTCCGGATTGGAGTCT
GCAACTCGACTCCATGAAGTCGGAATCGCTAGTAATCGTAGATCAGAATGCTACGGTGA
ATACGTTCCCGGGCCTTGTACACACCGCCCGTCACACCATGGGAGTGGGTTGCAAAAG
AAGTAGGTAGCTTAACCTTCGGGAGGGCGCTTACCACTTTGTGATTCATGACTGGGG
pattern 230
CGCTGGCGGCAGGCCTAACACATGCAAGTCGAGCGGCAGCGGAAAGTAGCTTGCTACT
TTGCCGGCGAGCGGCGGACGGGTGAGTAATGTCTGGGAAACTGCCTGATGGAGGGGGA

TAACTACTGGAAACGGTAGCTAATACCGCATGACCTCGCAAGAGCAAAGTGGGGGACC
TTCGGGCCTCACGCCATCGGATGTGCCCAGATGGGATTAGCTAGTAGGTGGGGTAATGG
CTCACCTAGGCGACGATCCCTAGCTGGTCTGAGAGGATGACCAGCCACACTGGAACTG
AGACACGGTCCAGACTCCTACGGGAGGCAGCAGTGGGGAATATTGCACAATGGGCGCA
AGCCTGATGCAGCCATGCCGCGTGTGTGAAGAAGGCCTTCGGGTTGTAAAGCACTTTCA
GCGAGGAGGAAGGCAGTCGTGTTAATAGCACGGTTGATTGACGTTACTCGCAGAAGAA
GCACCGGCTAACTCCGTGCCAGCAGCCGCGGTAATACGGAGGGTGCAAGCGTTAATCG
GAATTACTGGGCGTAAAGCGCACGCAGGCGGTTTGTTAAGTCAGATGTGAAATCCCCGC
GCTTAACGTGGGAACTGCATTTGAAACTGGCAAGCTAGAGTCTTGTAGAGGGGGGTAG
AATTCCAGGTGTAGCGGTGAAATGCGTAGAGATCTGGAGGAATACCGGTGGCGAAGGC
GGCCCCCTGGACAAAGACTGACGCTCAGGTGCGAAAGCGTGGGGAGCAAACAGGATT
AGATACCCTGGTAGTCCACGCTGTAAACGATGTCGACTTGGAGGTTGTGCCCTTGAGGC
GTGGCTTCCGGAGCTAACGCGTTAAGTCGACCGCCTGGGGAGTACGGCCGCAAGGTTA
AAACTCAAATGAATTGACGGGGGCCCGCACAAGCGGTGGAGCATGTGGTTTAATTCGAT
GCAACGCGAAGAACCTTACCTACTCTTGACATCCACAGAACTTAGCAGAGATGCTTCGG
TGCCTTCGGGAACTGTGAGACAGGTGCTGCATGGCTGTCGTCAGCTCGTGTTGTGAAAT
GTTGGGTTAAGTCCCGCAACGAGCGCAACCCTTATCCTTTGTTGCCAGCACGTAATGGT
GGGAACTCAAGGGAGACTGCCGGTGACAAACCGGAGGAAGGTGGGGATGACGTCAAG
TCATCATGGCCCTTACGAGTAGGGCTACACACGTGCTACAATGGCAGATACAAAGTGAA
GCGAACTCGCGAGAGTCAGCGGACCACATAAAGTCTGTCGTAGTCCGGATTGGAGTCT
GCAACTCGACTCCATGAAGTCGGAATCGCTAGTAATCGTAGATCAGAATGCTACGGTGA
ATACGTTCCCGGGCCTTGTACACACCGCCCGTCACACCATGGGAGTGGGTTGCAAAAG
AAGTAGGTAGCTTAACCTTCGGGAGGGCGCTTACCACTTTGTGATTCATGACTGGGG
pattern 231
CGCTGGCGGCAGGCCTAACACATGCAAGTCGAGCGGCAGCGGGAAGTAGTTTACTACT
TTGCCGGCGAGCGGCGGACGGGTGAGTAATGTCTGGGGATCTGCCTGATGGAGGGGGA
TAACTACTGGAAACGGTAGCTAATACCGCATGACCTCGCAAGAGCAAAGTGGGGGACC
TTAGGGCCTCACGCCATCGGATGAACCCAGATGGGATTAGCTAGTAGGTGGGGTAATGG
CTCACCTAGGCGACGATCCCTAGCTGGTCTGAGAGGATGAGCAGCCACACTGGAACTG
AGACACGGTCCAGACTCCTACGGGAGGCAGCAGTGGGGAATATTGCACAATGGGCGCA
AGCCTGATGCAGCCATGCCGCGTGTGTGAAGAAGGCCTTCGGGTTGTAAAGCACTTTCA
GCGAGGAGGAAGGGGTTGAGTTTAATACGCTCAATCATTGACGTTACTCGCAGAAGAA
GCACCGGCTAACTCCGTGCCAGCAGCCGCGGTAATACGGAGGGTGCAAGCGTTAATCG
GAATTACTGGGCGTAAAGCGCACGCAGGCGGTTTGTTAAGTCAGATGTGAAATCCCCGC
GCTTAACGTGGGAACTGCATTTGAAACTGGCAAGCTAGAGTCTTGTAGAGGGGGGTAG
AATTCCAGGTGTAGCGGTGAAATGCGTAGAGATCTGGAGGAATACCGGTGGCGAAGGC
GGCCCCCTGGACAAAGACTGACGCTCAGGTGCGAAAGCGTGGGGAGCAAACAGGATT
AGATACCCTGGTAGTCCACGCTGTAAACGATGTCGACTTGGAGGTTGTGCCCTTGAGGC
GTGGCTTCCGGAGCTAACGCGTTAAGTCGACCGCCTGGGGAGTACGGCCGCAAGGTTA
AAACTCAAATGAATTGACGGGGGCCCGCACAAGCGGTGGAGCATGTGGTTTAATTCGAT
GCAACGCGAAGAACCTTACCTACTCTTGACATCCACAGAATTTGGCAGAGATGCTAAAG
TGCCTTCGGGAACTGTGAGACAGGTGCTGCATGGCTGTCGTCAGCTCGTGTTGTGAAAT
GTTGGGTTAAGTCCCGCAACGAGCGCAACCCTTATCCTTTGTTGCCAGCACGTAATGGT
GGGAACTCAAGGGAGACTGCCGGTGACAAACCGGAGGAAGGTGGGGATGACGTCAAG

TCATCATGGCCCTTACGAGTAGGGCTACACACGTGCTACAATGGCAGATACAAAGTGAA
GCGAACTCGCGAGAGCCAGCGGACCACATAAAGTCTGTCGTAGTCCGGATTGGAGTCT
GCAACTCGACTCCATGAAGTCGGAATCGCTAGTAATCGTAGATCAGAATGCTACGGTGA
ATACGTTCCCGGGCCTTGTACACACCGCCCGTCACACCATGGGAGTGGGTTGCAAAAG
AAGTAGGTAGCTTAACCTTCGGGAGGGCGCTTACCACTTTGTGATTCATGACTGGGG
pattern 232
CGCTGGCGGCAGGCCTAACACATGCAAGTCGAGCGGCAGCGGGAAGTAGTTTACTACT
TTGCCGGCGAGCGGCGGACGGGTGAGTAATGTCTGGGAAGCTGCCTGATGGAGGGGGA
TAACTACTGGAAACGGTAGCTAATACCGCATAACGTCTTCGGACCAAAGTGGGGGACCT
TCGGGCCTCACGCCATCGGATGTGCCCAGATGGGATTAGCTAGTAGGTGGGGTAATGGC
TCACCTAGGCGACGATCCCTAGCTGGTCTGAGAGGATGACCAGCCACACTGGAACTGA
GACACGGTCCAGACTCCTACGGGAGGCAGCAGTGGGGAATATTGCACAATGGGCGCAA
GCCTGATGCAGCCATGCCGCGTGTGTGAAGAAGGCCTTCGGGTTGTAAAGCACTTTCAG
CGAGGAGGAAGGCATAAAGGTTAATAACCTTTGTGATTGACGTTACTCGCAGAAGAAG
CACCGGCTAACTCCGTGCCAGCAGCCGCGGTAATACGGAGGGTGCAAGCGTTAATCGG
AATTACTGGGCGTAAAGCGCACGCAGGCGGTTTGTTAAGTCAGATGTGAAATCCCCGCG
CTTAACGTGGGAACTGCATTTGAAACTGGCAAGCTAGAGTCTTGTAGAGGGGGGTAGA
ATTCCAGGTGTAGCGGTGAAATGCGTAGAGATCTGGAGGAATACCGGTGGCGAAGGCG
GCCCCCTGGACAAAGACTGACGCTCAGGTGCGAAAGCGTGGGGAGCAAACAGGATTA
GATACCCTGGTAGTCCACGCTGTAAACGATGTCGACTTGGAGGTTGTGCCCTTGAGGCG
TGGCTTCCGGAGCTAACGCGTTAAGTCGACCGCCTGGGGAGTACGGCCGCAAGGTTAA
AACTCAAATGAATTGACGGGGGCCCGCACAAGCGGTGGAGCATGTGGTTTAATTCGATG
CAACGCGAAGAACCTTACCTACTCTTGACATCCACAGAACTTAGCAGAGATGCTTCGGT
GCCTTCGGGAACTGTGAGACAGGTGCTGCATGGCTGTCGTCAGCTCGTGTTGTGAAATG
TTGGGTTAAGTCCCGCAACGAGCGCAACCCTTATCCTTTGTTGCCAGCACGTCATGGTG
GGAACTCAAAGGAGACTGCCGGTGATAAACCGGAGGAAGGTGGGGATGACGTCAAGT
CATCATGGCCCTTACGAGTAGGGCTACACACGTGCTACAATGGCAGATACAAAGTGAAG
CGAACTCGCGAGAGCAAGCGGACCACATAAAGTCTGTCGTAGTCCGGATTGGAGTCTG
CAACTCGACTCCATGAAGTCGGAATCGCTAGTAATCGTAGATCAGAATGCTACGGTGAAT
ACGTTCCCGGGCCTTGTACACACCGCCCGTCACACCATGGGAGTGGGTTGCAAAAGAA
GTAGGTAGCTTAACCTTCGGGAGGGCGCTTACCACTTTGTGATTCATGACTGGGG
pattern 233
CGCTGGCGGCAGGCCTAACACATGCAAGTCGAGCGGCAGCGGGAAGTAGTTTACTACT
TTGCCGGCGAGCGGCGGACGGGTGAGTAATGTCTGGGAAACTGCCTGATGGGGGGGGA
TAACTACTGGAAACGGTAGCTAATACCGCATAACGTCTTCGGACCAAAGTGGGGGACCT
TCGGGCCTCACGCCATCGGATGTGCCCAGATGGGATTAGCTAGTAGGTGGGGTAATGGC
TCACCTAGGCGACGATCCCTAGCTGGTCTGAGAGGATGACCAGCCACACTGGAACTGA
GACACGGTCCAGACTCCTACGGGAGGCAGCAGTGGGGAATATTGCACAATGGGCGCAA
GCCTGATGCAGCCATGCCGCGTGTGTGAAGAAGGCCTTCGGGTTGTAAAGCACTTTCAG
CGAGGAGGAAGGCATAAAGGTTAATAACCTTTGTGATTGACGTTACTCGCAGAAGAAG
CACCGGCTAACTCCGTGCCAGCAGCCGCGGTAATACGGAGGGTGCAAGCGTTAATCGG
AATTACTGGGCGTAAAGCGCACGCAGGCGGTTTGTTAAGTCAGATGTGAAATCCCCGCG
CTTAACGTGGGAACTGCATTTGAAACTGGCAAGCTAGAGTCTTGTAGAGGGGGGTAGA
ATTCCAGGTGTAGCGGTGAAATGCGTAGAGATCTGGAGGAATACCGGTGGCGAAGGCG

GCCCCCTGGACAAAGACTGACGCTCAGGTGCGAAAGCGTGGGGAGCAAACAGGATTA
GATACCCTGGTAGTCCACGCTGTAAACGATGTCGACTTGGAGGTTGTGCCCTTGAGGCG
TGGCTTCCGGAGCTAACGCGTTAAGTCGACCGCCTGGGGAGTACGGCCGCAAGGTTAA
AACTCAAATGAATTGACGGGGGCCCGCACAAGCGGTGGAGCATGTGGTTTAATTCGATG
CAACGCGAAGAACCTTACCTACTCTTGACATCCACGGAATTTAGCAGAGATGCTTTAGT
GCCTTCGGGAACCGTGAGACAGGTGCTGCATGGCTGTCGTCAGCTCGTGTTGTGAAATG
TTGGGTTAAGTCCCGCAACGAGCGCAACCCTTATCCTTTGTTGCCAGCACGTCATGGTG
GGAACTCAAAGGAGACTGCCGGTGATAAACCGGAGGAAGGTGGGGATGACGTCAAGT
CATCATGGCCCTTACGAGTAGGGCTACACACGTGCTACAATGGCAGATACAAAGTGAAG
CGAACTCGCGAGAGCAAGCGGACCACATAAAGTCTGTCGTAGTCCGGATTGGAGTCTG
CAACTCGACTCCATGAAGTCGGAATCGCTAGTAATCGTAGATCAGAATGCTACGGTGAAT
ACGTTCCCGGGCCTTGTACACACCGCCCGTCACACCATGGGAGTGGGTTGCAAAAGAA
GTAGGTAGCTTAACCTTCGGGAGGGCGCTTACCACTTTGTGATTCATGACTGGGG
pattern 234
CGCTGGCGGCAGGCCTAACACATGCAAGTCGAGCGGCAGCGGGAAGTAGTTTACTACT
TTGCCGGCGAGCGGCGGACGGGTGAGTAATGTCTGGGAAACTGCCTGATGGGGGGGA
TAACTACTGGAAACGGTAGCTAATACCGCATAACGTCTTCGGACCAAAGTGGGGGACCT
TCGGGCCTCACGCCATCGGATGTGCCCAGATGGGATTAGCTAGTAGGTGGGGTAATGGC
TCACCTAGGCGACGATCCCTAGCTGGTCTGAGAGGATGACCAGCCACACTGGAACTGA
GACACGGTCCAGACTCCTACGGGAGGCAGCAGTGGGGAATATTGCACAATGGGCGCAA
GCCTGATGCAGCCATGCCGCGTGTGTGAAGAAGGCCTTCGGGTTGTAAAGCACTTTCAG
CGAGGAGGAAGGCATAAAGGTTAATAACCTTTGTGATTGACGTTACTCGCAGAAGAAG
CACCGGCTAACTCCGTGCCAGCAGCCGCGGTAATACGGAGGGTGCAAGCGTTAATCGG
AATTACTGGGCGTAAAGCGCACGCAGGCGGTTTGTTAAGTCAGATGTGAAATCCCCGCG
CTTAACGTGGGAACTGCATTTGAAACTGGCAAGCTAGAGTCTTGTAGAGGGGGGTAGA
ATTCCAGGTGTAGCGGTGAAATGCGTAGAGATCTGGAGGAATACCGGTGGCGAAGGCG
GCCCCCTGGACAAAGACTGACGCTCAGGTGCGAAAGCGTGGGGAGCAAACAGGATTA
GATACCCTGGTAGTCCACGCTGTAAACGATGTCGACTTGGAGGTTGTGCCCTTGAGGCG
TGGCTTCCGGAGCTAACGCGTTAAGTCGACCGCCTGGGGAGTACGGCCGCAAGGTTAA
AACTCAAATGAATTGACGGGGGCCCGCACAAGCGGTGGAGCATGTGGTTTAATTCGATG
CAACGCGAAGAACCTTACCTACTCTTGACATCCACAGAACTTAGCAGAGATGCTTCGGT
GCCTTCGGGAACTGTGAGACAGGTGCTGCATGGCTGTCGTCAGCTCGTGTTGTGAAATG
TTGGGTTAAGTCCCGCAACGAGCGCAACCCTTATCCTTTGTTGCCAGCACGTCATGGTG
GGAACTCAAAGGAGACTGCCGGTGATAAACCGGAGGAAGGTGGGGATGACGTCAAGT
CATCATGGCCCTTACGAGTAGGGCTACACACGTGCTACAATGGCAGATACAAAGTGAAG
CGAACTCGCGAGAGCAAGCGGACCACATAAAGTCTGTCGTAGTCCGGATTGGAGTCTG
CAACTCGACTCCATGAAGTCGGAATCGCTAGTAATCGTAGATCAGAATGCTACGGTGAAT
ACGTTCCCGGGCCTTGTACACACCGCCCGTCACACCATGGGAGTGGGTTGCAAAAGAA
GTAGGTAGCTTAACCTTCGGGAGGGCGCTTACCACTTTGTGATTCATGACTGGGG
pattern 235
CGCTGGCGGCAGGCCTAACACATGCAAGTCGAGCGGCAGCGGGAAGTAGTTTACTACT
TTGCCGGCGAGCGGCGGACGGGTGAGTAATGTCTGGGAAACTGCCTGATGGGGGGGA
TAACTACTGGAAACGGTAGCTAATACCGCATAACGTCTTCGGACCAAAGTGGGGGACCT
TCGGGCCTCACGCCATCGGATGTGCCCAGATGGGATTAGCTAGTAGGTGGGGTAATGGC

TCACCTAGGCGACGATCCCTAGCTGGTCTGAGAGGATGACCAGCCACACTGGAACTGA
GACACGGTCCAGACTCCTACGGGAGGCAGCAGTGGGGAATATTGCACAATGGGCGCAA
GCCTGATGCAGCCATGCCGCGTGTGTGAAGAAGGCCTTCGGGTTGTAAAGCACTTTCAG
CGAGGAGGAAGGCATAAAGGTTAATAACCTTTGTGATTGACGTTACTCGCAGAAGAAG
CACCGGCTAACTCCGTGCCAGCAGCCGCGGTAATACGGAGGGTGCAAGCGTTAATCGG
AATTACTGGGCGTAAAGCGCACGCAGGCGGTTTGTTAAGTCAGATGTGAAATCCCCGCG
CTTAACGTGGGAACTGCATTTGAAACTGGCAAGCTAGAGTCTTGTAGAGGGGGGTAGA
ATTCCAGGTGTAGCGGTGAAATGCGTAGAGATCTGGAGGAATACCGGTGGCGAAGGCG
GCCCCCTGGACAAAGACTGACGCTCAGGTGCGAAAGCGTGGGGAGCAAACAGGATTA
GATACCCTGGTAGTCCACGCTGTAAACGATGTCGACTTGGAGGTTGTGCCCTTGAGGCG
TGGCTTCCGGAGCTAACGCGTTAAGTCGACCGCCTGGGGAGTACGGCCGCAAGGTTAA
AACTCAAATGAATTGACGGGGGCCCGCACAAGCGGTGGAGCATGTGGTTTAATTCGATG
CAACGCGAAGAACCTTACCTACTCTTGACATCCACAGAACTTAGCAGAGATGCTTCGGT
GCCTTCGGGAACTGTGAGACAGGTGCTGCATGGCTGTCGTCAGCTCGTGTTGTGAAATG
TTGGGTTAAGTCCCGCAACGAGCGCAACCCTTATCCTTTGTTGCCAGCGAGTAATGTCG
GGAACTCAAAGGAGACTGCCGGTGATAAACCGGAGGAAGGTGGGGATGACGTCAAGT
CATCATGGCCCTTACGAGTAGGGCTACACACGTGCTACAATGGCAGATACAAAGTGAAG
CGAACTCGCGAGAGCAAGCGGACCACATAAAGTCTGTCGTAGTCCGGATTGGAGTCTG
CAACTCGACTCCATGAAGTCGGAATCGCTAGTAATCGTAGATCAGAATGCTACGGTGAAT
ACGTTCCCGGGCCTTGTACACACCGCCCGTCACACCATGGGAGTGGGTTGCAAAAGAA
GTAGGTAGCTTAACCTTCGGGAGGGCGCTTACCACTTTGTGATTCATGACTGGGG
pattern 236
CGCTGGCGGCAGGCCTAACACATGCAAGTCGAGCGGCAGCGGGAAGTAGTTTACTACT
TTGCCGGCGAGCGGCGGACGGGTGAGTAATGTCTGGGAAACTGCCTGATGGGGGGGA
TAACTACTGGAAACGGTAGCTAATACCGCATAACGTCTTCGGACCAAAGTGGGGGACCT
TCGGGCCTCACGCCATCGGATGTGCCCAGATGGGATTAGCTAGTAGGTGGGGTAATGGC
TCACCTAGGCGACGATCCCTAGCTGGTCTGAGAGGATGACCAGCCACACTGGAACTGA
GACACGGTCCAGACTCCTACGGGAGGCAGCAGTGGGGAATATTGCACAATGGGCGCAA
GCCTGATGCAGCCATGCCGCGTGTGTGAAGAAGGCCTTCGGGTTGTAAAGCACTTTCAG
CGAGGAGGAAGGCATAAAGGTTAATAACCTTTGTGATTGACGTTACTCGCAGAAGAAG
CACCGGCTAACTCCGTGCCAGCAGCCGCGGTAATACGGAGGGTGCAAGCGTTAATCGG
AATTACTGGGCGTAAAGCGCACGCAGGCGGTTTGTTAAGTCAGATGTGAAATCCCCGCG
CTTAACGTGGGAACTGCATTTGAAACTGGCAAGCTAGAGTCTTGTAGAGGGGGGTAGA
ATTCCAGGTGTAGCGGTGAAATGCGTAGAGATCTGGAGGAATACCGGTGGCGAAGGCG
GCCCCCTGGACAAAGACTGACGCTCAGGTGCGAAAGCGTGGGGAGCAAACAGGATTA
GATACCCTGGTAGTCCACGCTGTAAACGATGTCGACTTGGAGGTTGTGCCCTTGAGGCG
TGGCTTCCGGAGCTAACGCGTTAAGTCGACCGCCTGGGGAGTACGGCCGCAAGGTTAA
AACTCAAATGAATTGACGGGGGCCCGCACAAGCGGTGGAGCATGTGGTTTAATTCGATG
CAACGCGAAGAACCTTACCTACTCTTGACATCCACGGAATTTAGCAGAGATGCTTTAGT
GCCTTCGGGAACCGTGAGACAGGTGCTGCATGGCTGTCGTCAGCTCGTGTTGTGAAATG
TTGGGTTAAGTCCCGCAACGAGCGCAACCCTTATCCTTTGTTGCCAGCGAGTAATGTCG
GGAACTCAAAGGAGACTGCCGGTGATAAACCGGAGGAAGGTGGGGATGACGTCAAGT
CATCATGGCCCTTACGAGTAGGGCTACACACGTGCTACAATGGCAGATACAAAGTGAAG
CGAACTCGCGAGAGCAAGCGGACCACATAAAGTCTGTCGTAGTCCGGATTGGAGTCTG

CAACTCGACTCCATGAAGTCGGAATCGCTAGTAATCGTAGATCAGAATGCTACGGTGAAT
ACGTTCCCGGGCCTTGTACACACCGCCCGTCACACCATGGGAGTGGGTTGCAAAAGAA
GTAGGTAGCTTAACCTTCGGGAGGGCGCTTACCACTTTGTGATTCATGACTGGGG
pattern 237
CGCTGGCGGCAGGCCTAACACATGCAAGTCGAGCGGCAGCGGGAAGTAGTTTACTACT
TTGCCGGCGAGCGGCGGACGGGTGAGTAATGTCTGGGAAACTGCCTGATGGGGGGGA
TAACTACTGGAAACGGTAGCTAATACCGCATAACGTCTACGGACCAAAGTGGGGGACCT
TCGGGCCTCACGCCATCGGATGTGCCCAGATGGGATTAGCTAGTAGGTGGGGTAATGGC
TCACCTAGGCGACGATCCCTAGCTGGTCTGAGAGGATGACCAGCCACACTGGAACTGA
GACACGGTCCAGACTCCTACGGGAGGCAGCAGTGGGGAATATTGCACAATGGGCGCAA
GCCTGATGCAGCCATGCCGCGTGTGTGAAGAAGGCCTTCGGGTTGTAAAGCACTTTCAG
CGAGGAGGAAGGCATAAAGGTTAATAACCTTTGTGATTGACGTTACTCGCAGAAGAAG
CACCGGCTAACTCCGTGCCAGCAGCCGCGGTAATACGGAGGGTGCAAGCGTTAATCGG
AATTACTGGGCGTAAAGCGCACGCAGGCGGTTTGTTAAGTCAGATGTGAAATCCCCGCG
CTTAACGTGGGAACTGCATTTGAAACTGGCAAGCTAGAGTCTTGTAGAGGGGGGTAGA
ATTCCAGGTGTAGCGGTGAAATGCGTAGAGATCTGGAGGAATACCGGTGGCGAAGGCG
GCCCCCTGGACAAAGACTGACGCTCAGGTGCGAAAGCGTGGGGAGCAAACAGGATTA
GATACCCTGGTAGTCCACGCTGTAAACGATGTCGACTTGGAGGTTGTGCCCTTGAGGCG
TGGCTTCCGGAGCTAACGCGTTAAGTCGACCGCCTGGGGAGTACGGCCGCAAGGTTAA
AACTCAAATGAATTGACGGGGGCCCGCACAAGCGGTGGAGCATGTGGTTTAATTCGATG
CAACGCGAAGAACCTTACCTACTCTTGACATCCACAGAACTTAGCAGAGATGCTTCGGT
GCCTTCGGGAACTGTGAGACAGGTGCTGCATGGCTGTCGTCAGCTCGTGTTGTGAAATG
TTGGGTTAAGTCCCGCAACGAGCGCAACCCTTATCCTTTGTTGCCAGCACGTCATGGTG
GGAACTCAAAGGAGACTGCCGGTGATAAACCGGAGGAAGGTGGGGATGACGTCAAGT
CATCATGGCCCTTACGAGTAGGGCTACACACGTGCTACAATGGCAGATACAAAGTGAAG
CGAACTCGCGAGAGCAAGCGGACCACATAAAGTCTGTCGTAGTCCGGATTGGAGTCTG
CAACTCGACTCCATGAAGTCGGAATCGCTAGTAATCGTAGATCAGAATGCTACGGTGAAT
ACGTTCCCGGGCCTTGTACACACCGCCCGTCACACCATGGGAGTGGGTTGCAAAAGAA
GTAGGTAGCTTAACCTTCGGGAGGGCGCTTACCACTTTGTGATTCATGACTGGGG
pattern 238
CGCTGGCGGCAGGCCTAACACATGCAAGTCGAGCGGCAGCGGGAAGTAGTTTACTACT
TTGCCGGCGAGCGGCGGACGGGTGAGTAATGTCTGGGAAACTGCCTGATGGAGGGGGA
TAACTACTGGAAACGGTAGCTAATACCGCATAACGTCTTCGGACCAAAGTGGGGGACCT
TCGGGCCTCACGCCATCGGATGTGCCCAGATGGGATTAGCTAGTAGGTGGGGTAATGGC
TCACCTAGGCGACGATCCCTAGCTGGTCTGAGAGGATGACCAGCCACACTGGAACTGA
GACACGGTCCAGACTCCTACGGGAGGCAGCAGTGGGGAATATTGCACAATGGGCGCAA
GCCTGATGCAGCCATGCCGCGTGTGTGAAGAAGGCCTTCGGGTTGTAAAGCACTTTCAG
CGAGGAGGAAGGCATAAAGGTTAATAATCTTTGTGATTGACGTTACTCGCAGAAGAAGC
ACCGGCTAACTCCGTGCCAGCAGCCGCGGTAATACGGAGGGTGCAAGCGTTAATCGGA
ATTACTGGGCGTAAAGCGCACGCAGGCGGTTTGTTAAGTCAGATGTGAAATCCCCGCGC
TTAACGTGGGAACTGCATTTGAAACTGGCAAGCTAGAGTCTTGTAGAGGGGGGTAGAA
TTCCAGGTGTAGCGGTGAAATGCGTAGAGATCTGGAGGAATACCGGTGGCGAAGGCGG
CCCCCTGGACAAAGACTGACGCTCAGGTGCGAAAGCGTGGGGAGCAAACAGGATTAG
ATACCCTGGTAGTCCACGCTGTAAACGATGTCGACTTGGAGGTTGTGCCCTTGAGGCGT

GGCTTCCGGAGCTAACGCGTTAAGTCGACCGCCTGGGGAGTACGGCCGCAAGGTTAAA
ACTCAAATGAATTGACGGGGGCCCGCACAAGCGGTGGAGCATGTGGTTTAATTCGATGC
AACGCGAAGAACCTTACCTACTCTTGACATCCACAGAACTTAGCAGAGATGCTTCGGTG
CCTTCGGGAACTGTGAGACAGGTGCTGCATGGCTGTCGTCAGCTCGTGTTGTGAAATGT
TGGGTTAAGTCCCGCAACGAGCGCAACCCTTATCCTTTGTTGCCAGCACGTCATGGTGG
GAACTCAAAGGAGACTGCCGGTGATAAACCGGAGGAAGGTGGGGATGACGTCAAGTC
ATCATGGCCCTTACGAGTAGGGCTACACACGTGCTACAATGGCAGATACAAAGTGAAGC
GAACTCGCGAGAGCAAGCGGACCACATAAAGTCTGTCGTAGTCCGGATTGGAGTCTGC
AACTCGACTCCATGAAGTCGGAATCGCTAGTAATCGTAGATCAGAATGCTACGGTGAAT
ACGTTCCCGGGCCTTGTACACACCGCCCGTCACACCATGGGAGTGGGTTGCAAAAGAA
GTAGGTAGCTTAACCTTCGGGAGGGCGCTTACCACTTTGTGATTCATGACTGGGG
pattern 239
CGCTGGCGGCAGGCCTAACACATGCAAGTCGAGCGGCAGCGGAAAGTAGCTTGCTACT
TTGCCGGCGAGCGGCGGACGGGTGAGTAATGTCTGGGGATCTGCCTGATGGAGGGGGA
TAACTACTGGAAACGGTAGCTAATACCGCATGACCTCGAAAGAGCAAAGTGGGGGACC
TTCGGGCCTCACGCCATCGGATGAACCCAGATGGGATTAGCTAGTAGGTGGGGTAATGG
CTCACCTAGGCGACGATCCCTAGCTGGTCTGAGAGGATGACCAGCCACACTGGAACTG
AGACACGGTCCAGACTCCTACGGGAGGCAGCAGTGGGGAATATTGCACAATGGGCGCA
AGCCTGATGCAGCCATGCCGCGTGTGTGAAGAAGGCCTTCGGGTTGTAAAGCACTTTCA
GCGAGGAGGAAGGCATTGTGGTTAATAACCGCAGTGATTGACGTTACTCGCAGAAGAA
GCACCGGCTAACTCCGTGCCAGCAGCCGCGGTAATACGGAGGGTGCAAGCGTTAATCG
GAATTACTGGGCGTAAAGCGCACGCAGGCGGTTTGTTAAGTCAGATGTGAAATCCCCGC
GCTTAACGTGGGAACTGCATTTGAAACTGGCAAGCTAGAGTCTTGTAGAGGGGGGTAG
AATTCCAGGTGTAGCGGTGAAATGCGTAGAGATCTGGAGGAATACCGGTGGCGAAGGC
GGCCCCCTGGACAAAGACTGACGCTCAGGTGCGAAAGCGTGGGGAGCAAACAGGATT
AGATACCCTGGTAGTCCACGCTGTAAACGATGTCGACTTGGAGGTTGTGCCCTTGAGGC
GTGGCTTCCGGAGCTAACGCGTTAAGTCGACCGCCTGGGGAGTACGGCCGCAAGGTTA
AAACTCAAATGAATTGACGGGGGCCCGCACAAGCGGTGGAGCATGTGGTTTAATTCGAT
GCAACGCGAAGAACCTTACCTACTCTTGACATCCACAGAACTTAGCAGAGATGCTTCGG
TGCCTTCGGGAACTGTGAGACAGGTGCTGCATGGCTGTCGTCAGCTCGTGTTGTGAAAT
GTTGGGTTAAGTCCCGCAACGAGCGCAACCCTTATCCTTTGTTGCCAGCACGTAATGGT
GGGAACTCAAGGGAGACTGCCGGTGACAAACCGGAGGAAGGTGGGGATGACGTCAAG
TCATCATAGCCCTTACGAGTAGGGCTACACACGTGCTACAATGGCAGATACAAAGTGAA
GCGAACTCGCGAGAGCAAGCGGACCACATAAAGTCTGTCGTAGTCCGGATTGGAGTCT
GCAACTCGACTCCATGAAGTCGGAATCGCTAGTAATCGTAGATCAGAATGCTACGGTGA
ATACGTTCCCGGGCCTTGTACACACCGCCCGTCACACCATGGGAGTGGGTTGCAAAAG
AAGTAGGTAGCTTAACCTTCGGGAGGGCGCTTACCACTTTGTGATTCATGACTGGGG
pattern 240
CGCTGGCGGCAGGCCTAACACATGCAAGTCGAGCGGCAGCGGAAAGTAGCTTGCTACT
TTGCCGGCGAGCGGCGGACGGGTGAGTAATGTCTGGGGATCTGCCTGATGGAGGGGGA
TAACTACTGGAAACGGTAGCTAATACCGCATGACCTCGAAAGAGCAAAGTGGGGGACC
TTCGGGCCTCACGCCATCGGATGAACCCAGATGGGATTAGCTAGTAGGTGGGGTAATGG
CTCACCTAGGCGACGATCCCTAGCTGGTCTGAGAGGATGACCAGCCACACTGGAACTG
AGACACGGTCCAGACTCCTACGGGAGGCAGCAGTGGGGAATATTGCACAATGGGCGCA

AGCCTGATGCAGCCATGCCGCGTGTGTGAAGAAGGCCTTCGGGTTGTAAAGCACTTTCA
GCGAGGAGGAAGGCATTGTGGTTAATAACCACAGTGATTGACGTTACTCGCAGAAGAA
GCACCGGCTAACTCCGTGCCAGCAGCCGCGGTAATACGGAGGGTGCAAGCGTTAATCG
GAATTACTGGGCGTAAAGCGCACGCAGGCGGTTTGTTAAGTCAGATGTGAAATCCCCGC
GCTTAACGTGGGAACTGCATTTGAAACTGGCAAGCTAGAGTCTTGTAGAGGGGGGTAG
AATTCCAGGTGTAGCGGTGAAATGCGTAGAGATCTGGAGGAATACCGGTGGCGAAGGC
GGCCCCCTGGACAAAGACTGACGCTCAGGTGCGAAAGCGTGGGGAGCAAACAGGATT
AGATACCCTGGTAGTCCACGCTGTAAACGATGTCGACTTGGAGGTTGTGCCCTTGAGGC
GTGGCTTCCGGAGCTAACGCGTTAAGTCGACCGCCTGGGGAGTACGGCCGCAAGGTTA
AAACTCAAATGAATTGACGGGGGCCCGCACAAGCGGTGGAGCATGTGGTTTAATTCGAT
GCAACGCGAAGAACCTTACCTACTCTTGACATCCACGGAATTTAGCAGAGATGCTTTAG
TGCCTTCGGGAACCGTGAGACAGGTGCTGCATGGCTGTCGTCAGCTCGTGTTGTGAAAT
GTTGGGTTAAGTCCCGCAACGAGCGCAACCCTTATCCTTTGTTGCCAGCACGTAATGGT
GGGAACTCAAGGGAGACTGCCGGTGACAAACCGGAGGAAGGTGGGGATGACGTCAAG
TCATCATGGCCCTTACGAGTAGGGCTACACACGTGCTACAATGGCAGATACAAAGTGAA
GCGAACTCGCGAGAGCAAGCAGACCACATAAAGTCTGTCGTAGTCCGGATTGGAGTCT
GCAACTCGACTCCATGAAGTCGGAATCGCTAGTAATCGTAGATCAGAATGCTACGGTGA
ATACGTTCCCGGGCCTTGTACACACCGCCCGTCACACCATGGGAGTGGGTTGCAAAAG
AAGTAGGTAGCTTAACCTTCGGGAGGGCGCTTACCACTTTGTGATTCATGACTGGGG
pattern 241
CGCTGGCGGCAGGCCTAACACATGCAAGTCGAGCGGCAGCGGGAAGTAGCTTGCTACT
TTGCCGGCGAGCGGCGGACGGGTGAGTAATGTCTGGGAAACTGCCTGATGGAGGGGGA
TAACTACTGGAAACGGTAGCTAATACCGCATAACGTCTACGGACCAAAGTGGGGGACCT
TCGGGCCTCACGCCATCGGATGTGCCCAGATGGGATTAGCTAGTAGGTGGGGTAATGGC
TCACCTAGGCGACGATCCCTAGCTGGTCTGAGGGGATGACCAGCCACACTGGAACTGA
GACACGGTCCAGACTCCTACGGGAGGCAGCAGTGGGGAATATTGCACAATGGGCGCAA
GCCTGATGCAGCCATGCCGCGTGTGTGAAGAAGGCCTTCGGGTTGTAAAGCACTTTCAG
CGAGGAGGAAGGCATAAAGGTTAATAACCTTTGTGATTGACGTTACTCGCAGAAGAAG
CACCGGCTAACTCCGTGCCAGCAGCCGCGGTAATACGGAGGGTGCAAGCGTTAATCGG
AATTACTGGGCGTAAAGCGCACGCAGGCGGTTTGTTAAGTCAGATGTGAAATCCCCGCG
CTTAACGTGGGAACTGCATTTGAAACTGGCAAGCTAGAGTCTTGTAGAGGGGGGTAGA
ATTCCAGGTGTAGCGGTGAAATGCGTAGAGATCTGGAGGAATACCGGTGGCGAAGGCG
GCCCCCTGGACAAAGACTGACGCTCAGGTGCGAAAGCGTGGGGAGCAAACAGGATTA
GATACCCTGGTAGTCCACGCTGTAAACGATGTCGACTTGGAGGTTGTGCCCTTGAGGCG
TGGCTTCCGGAGCTAACGCGTTAAGTCGACCGCCTGGGGAGTACGGCCGCAAGGTTAA
AACTCAAATGAATTGACGGGGGCCCGCACAAGCGGTGGAGCATGTGGTTTAATTCGATG
CAACGCGAAGAACCTTACCTACTCTTGACATCCACGGAATTTAGCAGAGATGCTTTAGT
GCCTTCGGGAACCGTGAGACAGGTGCTGCATGGCTGTCGTCAGCTCGTGTTGTGAAATG
TTGGGTTAAGTCCCGCAACGAGCGCAACCCTTATCCTTTGTTGCCAGCACGTCATGGTG
GGAACTCAAAGGAGACTGCCGGTGATAAACCGGAGGAAGGTGGGGATGACGTCAAGT
CATCATGGCCCTTACGAGTAGGGCTACACACGTGCTACAATGGCAGATACAAAGTGAAG
CGAACTCGCGAGAGCAAGCGGACCACATAAAGTCTGTCGTAGTCCGGATTGGAGTCTG
CAACTCGACTCCATGAAGTCGGAATCGCTAGTAATCGTAGATCAGAATGCTACGGTGAAT
ACGTTCCCGGGCCTTGTACACACCGCCCGTCACACCATGGGAGTGGGTTGCAAAAGAA

GTAGGTAGCTTAACCTTCGGGAGGGCGCTTACCACTTTGTGATTCATGACTGGGG
pattern 242
CGCTGGCGGCAGGCCTAACACATGCAAGTCGAGCGGCAGCGGGAAGTAGTTTACTACT
TTGCCGGCGAGCGGCGGACGGGTGAGTAATGTCTGGGAAACTGCCTGATGGAGGGGGA
TAACTACTGGAAACGGTAGCTAATACCGCATGATCTCGAAAGAGCAAAGTGGGGGACCT
TCGGGCCTCACGCCATCGGATGTGCCCAGATGGGATTAGCTAGTAGGTGGGGTAATGGC
TCACCTAGGCGACGATCCCTAGCTGGTCTGAGAGGATGACCAGCCACACTGGAACTGA
GACACGGTCCAGACTCCTACGGGAGGCAGCAGTGGGGAATATTGCACAATGGGCGCAA
GCCTGATGCAGCCATGCCGCGTGTGTGAAGAAGGCCTTCGGGTTGTAAAGCACTTTCAG
CGAGGAGGAAGGCAGTCGTGTTAATAGCACGATTGATTGACGTTACTCGCAGAAGAAG
CACCGGCTAACTCCGTGCCAGCAGCCGCGGTAATACGGAGGGTGCAAGCGTTAATCGG
AATTACTGGGCGTAAAGCGCACGCAGGCGGTTTGTTAAGTCAGATGTGAAATCCCCGCG
CTTAACGTGGGAACTGCATTTGAAACTGGCAAGCTAGAGTCTTGTAGAGGGGGGTAGA
ATTCCAGGTGTAGCGGTGAAATGCGTAGAGATCTGGAGGAATACCGGTGGCGAAGGCG
GCCCCCTGGACAAAGACTGACGCTCAGGTGCGAAAGCGTGGGGAGCAAACAGGATTA
GATACCCTGGTAGTCCACGCTGTAAACGATGTCGACTTGGAGGTTGTGCCCTTGAGGCG
TGGCTTCCGGAGCTAACGCGTTAAGTCGACCGCCTGGGGAGTACGGCCGCAAGGTTAA
AACTCAAATGAATTGACGGGGGCCCGCACAAGCGGTGGAGCATGTGGTTTAATTCGATG
CAACGCGAAGAACCTTACCTACTCTTGACATCCACAGAACTTAGCAGAGATGCTTCGGT
GCCTTCGGGAACTGTGAGACAGGTGCTGCATGGCNGTCGTCAGCTCGTGTTGTGAAAT
GTTGGGTTAAGTCCCGCAACGAGCGCAACCCTTATCCTTTGTTGCCAGCGAGTAATGTC
GGGAACTCAAAGGAGACTGCCGGTGATAAACCGGAGGAAGGTGGGGATGACGTCAAG
TCATCATGGCCCTTACGAGTAGGGCTACACACGTGCTACAATGGCAGATACAAAGTGAA
GCGAACTCGCGAGAGCAAGCGGACCACATAAAGTCTGTCGTAGTCCGGATTGGAGTCT
GCAACTCGACTCCATGAAGTCGGAATCGCTAGTAATCGTAGATCAGAATGCTACGGTGA
ATACGTTCCCGGGCCTTGTACACACCGCCCGTCACACCATGGGAGTGGGTTGCAAAAG
AAGTAGGTAGCTTAACCTTCGGGAGGGCGCTTACCACTTTGTGATTCATGACTGGGG
pattern 243
CGCTGGCGGCAGGCCTAACACATGCAAGTCGAGCGGCAGCGGGAAGTAGTTTACTACT
TTGCCGGCGAGCGGCGGACGGGTGAGTAATGTCTGGGAAACTGCCTGATGGAGGGGGA
TAACTACTGGAAACGGTAGCTAATACCGCATGACCTCGCAAGAGCAAAGTGGGGGACC
TTCGGGCCTCACGCCATCGGATGTGCCCAGATGGGATTAGCTAGTAGGTGGGGTAATGG
CTCACCTAGGCGACGATCCCTAGCTGGTCTGAGAGGATGACCAGCCACACTGGAACTG
AGACACGGTCCAGACTCCTACGGGAGGCAGCAGTGGGGAATATTGCACAATGGGCGCA
AGCCTGATGCAGCCATGCCGCGTGTGTGAAGAAGGCCTTCGGGTTGTAAAGCACTTTCA
GCGAGGAGGAAGGCCAGTAGCTTAATACGCTGTTGGATTGACGTTACTCGCAGAAGAA
GCACCGGCTAACTCCGTGCCAGCAGCCGCGGTAATACGGAGGGTGCAAGCGTTAATCG
GAATTACTGGGCGTAAAGCGCACGCAGGCGGTTTGTTAAGTCAGATGTGAAATCCCCGC
GCTTAACGTGGGAACTGCATTTGAAACTGGCAAGCTAGAGTCTTGTAGAGGGGGGTAG
AATTCCAGGTGTAGCGGTGAAATGCGTAGAGATCTGGAGGAATACCGGTGGCGAAGGC
GGCCCCCTGGACAAAGACTGACGCTCAGGTGCGAAAGCGTGGGGAGCAAACAGGATT
AGATACCCTGGTAGTCCACGCTGTAAACGATGTCGACTTGGAGGTTGTGCCCTTGAGGC
GTGGCTTCCGGAGCTAACGCGTTAAGTCGACCGCCTGGGGAGTACGGCCGCAAGGTTA
AAACTCAAATGAATTGACGGGGGCCCGCACAAGCGGTGGAGCATGTGGTTTAATTCGAT

GCAACGCGAAGAACCTTACCTACTCTTGACATCCACAGAACTTAGCAGAGATGCTTCGG
TGCCTTCGGGAACTGTGAGACAGGTGCTGCATGGCTGTCGTCAGCTCGTGTTGTGAAAT
GTTGGGTTAAGTCCCGCAACGAGCGCAACCCTTATCCTTTGTTGCCAGCACGTAATGGT
GGGAACTCAAGGGAGACTGCCGGTGACAAACCGGAGGAAGGTGGGGATGACGTCAAG
TCATCATGGCCCTTACGAGTAGGGCTACACACGTGCTACAATGGCAGATACAAAGTGAA
GCGAACTCGCGAGAGCAAGCGGACCACATAAAGTCTGTCGTAGTCCGGATTGGAGTCT
GCAACTCGACTCCATGAAGTCGGAATCGCTAGTAATCGTAGATCAGAATGCTACGGTGA
ATACGTTCCCGGGCCTTGTACACACCGCCCGTCACACCATGGGAGTGGGTTGCAAAAG
AAGTAGGTAGCTTAACCTTCGGGAGGGCGCTTACCACTTTGTGATTCATGACTGGGG
pattern 244
CGCTGGCGGCAGGCCTAACACATGCAAGTCGAGCGGCAGCGGGAAGTAGTTTACTACT
TTGCCGGCGAGCGGCGGACGGGTGAGTAATGTCTGGGAAACTGCCTGATGGAGGGGGA
TAACTACTGGAAACGGTAGCTAATACCGCATGACCTCGCAAGAGCAAAGTGGGGGACC
TTCGGGCCTCACGCCATCGGATGTGCCCAGATGGGATTAGCTAGTAGGTGGGGTAATGG
CTCACCTAGGCGACGATCCCTAGCTGGTCTGAGAGGATGACCAGCCACACTGGAACTG
AGACACGGTCCAGACTCCTACGGGAGGCAGCAGTGGGGAATATTGCACAATGGGCGCA
AGCCTGATGCAGCCATGCCGCGTGTGTGAAGAAGGCCTTCGGGTTGTAAAGCACTTTCA
GCGAGGAGGAAGGCCAATAGCTTAATACGCTGTTGGATTGACGTTACTCGCAGAAGAA
GCACCGGCTAACTCCGTGCCAGCAGCCGCGGTAATACGGAGGGTGCAAGCGTTAATCG
GAATTACTGGGCGTAAAGCGCACGCAGGCGGTTTGTTAAGTCAGATGTGAAATCCCCGC
GCTTAACGTGGGAACTGCATTTGAAACTGGCAAGCTAGAGTCTTGTAGAGGGGGGTAG
AATTCCAGGTGTAGCGGTGAAATGCGTAGAGATCTGGAGGAATACCGGTGGCGAAGGC
GGCCCCCTGGACAAAGACTGACGCTCAGGTGCGAAAGCGTGGGGAGCAAACAGGATT
AGATACCCTGGTAGTCCACGCTGTAAACGATGTCGACTTGGAGGTTGTGCCCTTGAGGC
GTGGCTTCCGGAGCTAACGCGTTAAGTCGACCGCCTGGGGAGTACGGCCGCAAGGTTA
AAACTCAAATGAATTGACGGGGGCCCGCACAAGCGGTGGAGCATGTGGTTTAATTCGAT
GCAACGCGAAGAACCTTACCTACTCTTGACATCCACAGAACTTAGCAGAGATGCTTCGG
TGCCTTCGGGAACTGTGAGACAGGTGCTGCATGGCCGTCGTCAGCTCGTGTTGTGAAAT
GTTGGGTTAAGTCCCGCAACGAGCGCAACCCTTATCCTTTGTTGCCAGCACGTAATGGT
GGGAACTCAAGGGAGACTGCCGGTGACAAACCGGAGGAAGGTGGGGATGACGTCAAG
TCATCATGGCCCTTACGAGTAGGGCTACACACGTGCTACAATGGCAGATACAAAGTGAA
GCGAACTCGCGAGAGCAAGCGGACCACATAAAGTCTGTCGTAGTCCGGATTGGAGTCT
GCAACTCGACTCCATGAAGTCGGAATCGCTAGTAATCGTAGATCAGAATGCTACGGTGA
ATACGTTCCCGGGCCTTGTACACACCGCCCGTCACACCATGGGAGTGGGTTGCAAAAG
AAGTAGGTAGCTTAACCTTCGGGAGGGCGCTTACCACTTTGTGATTCATGACTGGGG
pattern 245
CGCTGGCGGCAGGCCTAACACATGCAAGTCGAGCGGCAGCGGGAAGTAGTTTACTACT
TTGCCGGCGAGCGGCGGACGGGTGAGTAATGTCTGGGAAACTGCCTGATGGAGGGGGA
TAACTACTGGAAACGGTAGCTAATACCGCATGACCTCGCAAGAGCAAAGTGGGGGACC
TTCGGGCCTCACGCCATCGGATGTGCCCAGATGGGATTAGCTAGTAGGTGGGGTAATGG
CTCACCTAGGCGACGATCCCTAGCTGGTCTGAGAGGATGACCAGCCACACTGGAACTG
AGACACGGTCCAGACTCCTACGGGAGGCAGCAGTGGGGAATATTGCACAATGGGCGCA
AGCCTGATGCAGCCATGCCGCGTGTGTGAAGAAGGCCTTCGGGTTGTAAAGCACTTTCA
GCGAGGAGGAAGGCCAATAGCTTAATACGCTGTTGGATTGACGTTACTCGCAGAAGAA

GCACCGGCTAACTCCGTGCCAGCAGCCGCGGTAATACGGAGGGTGCAAGCGTTAATCG
GAATTACTGGGCGTAAAGCGCACGCAGGCGGTTTGTTAAGTCAGATGTGAAATCCCCGC
GCTTAACGTGGGAACTGCATTTGAAACTGGCAAGCTAGAGTCTTGTAGAGGGGGGTAG
AATTCCAGGTGTAGCGGTGAAATGCGTAGAGATCTGGAGGAATACCGGTGGCGAAGGC
GGCCCCCTGGACAAAGACTGACGCTCAGGTGCGAAAGCGTGGGGAGCAAACAGGATT
AGATACCCTGGTAGTCCACGCTGTAAACGATGTCGACTTGGAGGTTGTGCCCTTGAGGC
GTGGCTTCCGGAGCTAACGCGTTAAGTCGACCGCCTGGGGAGTACGGCCGCAAGGTTA
AAACTCAAATGAATTGACGGGGGCCCGCACAAGCGGTGGAGCATGTGGTTTAATTCGAT
GCAACGCGAAGAACCTTACCTACTCTTGACATCCACAGAACTTAGCAGAGATGCTTCGG
TGCCTTCGGGAACTGTGAGACAGGTGCTGCATGGCTGTCGTCAGCTCGTGTTGTGAAAT
GTTGGGTTAAGTCCCGCAACGAGCGCAACCCTTATCCTTTGTTGCCAGCACGTAATGGT
GGGAACTCAAGGGAGACTGCCGGTGACAAACCGGAGGAAGGTGGGGATGACGTCAAG
TCATCATGGCCCTTACGAGTAGGGCTACACACGTGCTACAATGGCAGATACAAAGTGAA
GCGAACTCGCGAGAGCAAGCGGACCACATAAAGTCTGTCGTAGTCCGGATTGGAGTCT
GCAACTCGACTCCATGAAGTCGGAATCGCTAGTAATCGTAGATCAGAATGCTACGGTGA
ATACGTTCCCGGGCCTTGTACACACCGCCCGTCACACCATGGGAGTGGGTTGCAAAAG
AAGTAGGTAGCTTAACCTTCGGGAGGGCGCTTACCACTTTGTGATTCATGACTGGGG
pattern 246
CGCTGGCGGCAGGCCTAACACATGCAAGTCGAGCGGCAGCGGGAAGTAGTTTACTACT
TTGCCGGCGAGCGGCGGACGGGTGAGTAATGTCTGGGAAACTGCCTGATGGAGGGGGA
TAACTACTGGAAACGGTAGCTAATACCGCATGACCTCGCAAGAGCAAAGTGGGGGACC
TTCGGGCCTCACGCCATCGGATGTGCCCAGATGGGATTAGCTAGTAGGTGAGGTAATGG
CTCACCTAGGCGACGATCCCTAGCTGGTCTGAGAGGATGACCAGCCACACTGGAACTG
AGACACGGTCCAGACTCCTACGGGAGGCAGCAGTGGGGAATATTGCACAATGGGCGCA
AGCCTGATGCAGCCATGCCGCGTGTGTGAAGAAGGCCTTCGGGTTGTAAAGCACTTTCA
GCGAGGAGGAAGGGTTCAGTGTTAATAGCACTGAGCATTGACGTTACTCGCAGAAGAA
GCACCGGCTAACTCCGTGCCAGCAGCCGCGGTAATACGGAGGGTGCAAGCGTTAATCG
GAATTACTGGGCGTAAAGCGCACGCAGGCGGTTTGTTAAGTCAGATGTGAAATCCCCGC
GCTTAACGTGGGAACTGCATTTGAAACTGGCAAGCTAGAGTCTTGTAGAGGGGGGTAG
AATTCCAGGTGTAGCGGTGAAATGCGTAGAGATCTGGAGGAATACCGGTGGCGAAGGC
GGCCCCCTGGACAAAGACTGACGCTCAGGTGCGAAAGCGTGGGGAGCAAACAGGATT
AGATACCCTGGTAGTCCACGCTGTAAACGATGTCGACTTGGAGGTTGTGCCCTTGAGGC
GTGGCTTCCGGAGCTAACGCGTTAAGTCGACCGCCTGGGGAGTACGGCCGCAAGGTTA
AAACTCAAATGAATTGACGGGGGCCCGCACAAGCGGTGGAGCATGTGGTTTAATTCGAT
GCAACGCGAAGAACCTTACCTACTCTTGACATCCACGGAATTTGGCAGAGATGCCTTAG
TGCCTTCGGGAACCGTGAGACAGGTGCTGCATGGCTGTCGTCAGCTCGTGTTGTGAAAT
GTTGGGTTAAGTCCCGCAACGAGCGCAACCCTTATCCTTTGTTGCCAGCACGTAATGGT
GGGAACTCAAGGGAGACTGCCGGTGACAAACCGGAGGAAGGTGGGGATGACGTCAAG
TCATCATGGCCCTTACGAGTAGGGCTACACACGTGCTACAATGGCAGATACAAAGTGAA
GCGAACTCGCGAGAGCAAGCGGACCACATAAAGTCTGTCGTAGTCCGGATTGGAGTCT
GCAACTCGACTCCATGAAGTCGGAATCGCTAGTAATCGTAGATCAGAATGCTACGGTGA
ATACGTTCCCGGGCCTTGTACACACCGCCCGTCACACCATGGGAGTGGGTTGCAAAAG
AAGTAGGTAGCTTAACCTTCGGGAGGGCGCTTACCACTTTGTGATTCATGACTGGGG
pattern 247

CGCTGGCGGCAGGCCTAACACATGCAAGTCGAGCGGCAGCGGGAAGTAGTTTACTACT
TTGCCGGCGAGCGGCGGACGGGTGAGTAATGTCTGGGAAACTGCCTGATGGAGGGGGA
TAACTACTGGAAACGGTAGCTAATACCGCATGACCTCGCAAGAGCAAAGTGGGGGACC
TTCGGGCCTCACGCCATCGGATGTGCCCAGATGGGATTAGCTAGTAGGTGAGGTAATGG
CTCACCTAGGCGACGATCCCTAGCTGGTCTGAGAGGATGACCAGCCACACTGGAACTG
AGACACGGTCCAGACTCCTACGGGAGGCAGCAGTGGGGAATATTGCACAATGGGCGCA
AGCCTGATGCAGCCATGCCGCGTGTGTGAAGAAGGCCTTCGGGTTGTAAAGCACTTTCA
GCGAGGAGGAAGGGTTCAGTGTTAATAGCACTGAGCATTGACGTTACTCGCAGAAGAA
GCACCGGCTAACTCCGTGCCAGCAGCCGCGGTAATACGGAGGGTGCAAGCGTTAATCG
GAATTACTGGGCGTAAAGCGCACGCAGGCGGTTTGTTAAGTCAGATGTGAAATCCCCGC
GCTTAACGTGGGAACTGCATTTGAAACTGGCAAGCTAGAGTCTTGTAGAGGGGGGTAG
AATTCCAGGTGTAGCGGTGAAATGCGTAGAGATCTGGAGGAATACCGGTGGCGAAGGC
GGCCCCCTGGACAAAGACTGACGCTCAGGTGCGAAAGCGTGGGGAGCAAACAGGATT
AGATACCCTGGTAGTCCACGCTGTAAACGATGTCGACTTGGAGGTTGTGCCCTTGAGGC
GTGGCTTCCGGAGCTAACGCGTTAAGTCGACCGCCTGGGGAGTACGGCCGCAAGGTTA
AAACTCAAATGAATTGACGGGGGCCCGCACAAGCGGTGGAGCATGTGGTTTAATTCGAT
GCAACGCGAAGAACCTTACCTACTCTTGACATCCACAGAATTTGGCAGAGATGCCTTAG
TGCCTTCGGGAACTGTGAGACAGGTGCTGCATGGCTGTCGTCAGCTCGTGTTGTGAAAT
GTTGGGTTAAGTCCCGCAACGAGCGCAACCCTTATCCTTTGTTGCCAGCACGTAATGGT
GGGAACTCAAGGGAGACTGCCGGTGACAAACCGGAGGAAGGTGGGGATGACGTCAAG
TCATCATGGCCCTTACGAGTAGGGCTACACACGTGCTACAATGGCAGATACAAAGTGAA
GCGAACTCGCGAGAGCAAGCGGACCACATAAAGTCTGTCGTAGTCCGGATTGGAGTCT
GCAACTCGACTCCATGAAGTCGGAATCGCTAGTAATCGTAGATCAGAATGCTACGGTGA
ATACGTTCCCGGGCCTTGTACACACCGCCCGTCACACCATGGGAGTGGGTTGCAAAAG
AAGTAGGTAGCTTAACCTTCGGGAGGGCGCTTACCACTTTGTGATTCATGACTGGGG
pattern 248
CGCTGGCGGCAGGCCTAACACATGCAAGTCGAGCGGCAGCGGGAAGTAGTTTACTACT
TTGCCGGCGAGCGGCGGACGGGTGAGTAATGTCTGGGAAACTGCCTGATGGAGGGGGA
TAACTACTGGAAACGGTAGCTAATACCGCATGACCTCGCAAGAGCAAAGTGGGGGACC
TTCGGGCCTCACGCCATCGGATGTGCCCAGATGGGATTAGCTAGTAGGTGAGGTAATGG
CTCACCTAGGCGACGATCCCTAGCTGGTCTGAGAGGATGACCAGCCACACTGGAACTG
AGACACGGTCCAGACTCCTACGGGAGGCAGCAGTGGGGAATATTGCACAATGGGCGCA
AGCCTGATGCAGCCATGCCGCGTGTGTGAAGAAGGCCTTCGGGTTGTAAAGCACTTTCA
GCGAGGAGGAAGGGTTTAGTGTTAATAGCACTGAGCATTGACGTTACTCGCAGAAGAA
GCACCGGCTAACTCCGTGCCAGCAGCCGCGGTAATACGGAGGGTGCAAGCGTTAATCG
GAATTACTGGGCGTAAAGCGCACGCAGGCGGTTTGTTAAGTCAGATGTGAAATCCCCGC
GCTTAACGTGGGAACTGCATTTGAAACTGGCAAGCTAGAGTCTTGTAGAGGGGGGTAG
AATTCCAGGTGTAGCGGTGAAATGCGTAGAGATCTGGAGGAATACCGGTGGCGAAGGC
GGCCCCCTGGACAAAGACTGACGCTCAGGTGCGAAAGCGTGGGGAGCAAACAGGATT
AGATACCCTGGTAGTCCACGCTGTAAACGATGTCGACTTGGAGGTTGTGCCCTTGAGGC
GTGGCTTCCGGAGCTAACGCGTTAAGTCGACCGCCTGGGGAGTACGGCCGCAAGGTTA
AAACTCAAATGAATTGACGGGGGCCCGCACAAGCGGTGGAGCATGTGGTTTAATTCGAT
GCAACGCGAAGAACCTTACCTACTCTTGACATCCACGGAATTTGGCAGAGATGCCTTAG
TGCCTTCGGGAACCGTGAGACAGGTGCTGCATGGCTGTCGTCAGCTCGTGTTGTGAAAT

GTTGGGTTAAGTCCCGCAACGAGCGCAACCCTTATCCTTTGTTGCCAGCACGTAATGGT
GGGAACTCAAGGGAGACTGCCGGTGACAAACCGGAGGAAGGTGGGGATGACGTCAAG
TCATCATGGCCCTTACGAGTAGGGCTACACACGTGCTACAATGGCAGATACAAAGTGAA
GCGAACTCGCGAGAGCAAGCGGACCACATAAAGTCTGTCGTAGTCCGGATTGGAGTCT
GCAACTCGACTCCATGAAGTCGGAATCGCTAGTAATCGTAGATCAGAATGCTACGGTGA
ATACGTTCCCGGGCCTTGTACACACCGCCCGTCACACCATGGGAGTGGGTTGCAAAAG
AAGTAGGTAGCTTAACCTTCGGGAGGGCGCTTACCACTTTGTGATTCATGACTGGGG
pattern 249
CGCTGGCGGCAGGCCTAACACATGCAAGTCGAGCGGCAGCGGGAAGTAGTTTACTACT
TTGCCGGCGAGCGGCGGACGGGTGAGTAATGTCTGGGAAACTGCCTGATGGAGGGGGA
TAACTACTGGAAACGGTAGCTAATACCGCATAACGTCTTCGGACCAAAGTGGGGGACCT
TCGGGCCTCACGCCATCGGATGTGCCCAGATGGGATTAGCTAGTAGGTGGGGTAATGGC
TCACCTAGGCGACGATCCCTAGCTGGTCTGAGAGGATGACCAGCCACACTGGAACTGA
GACACGGTCCAGACTCCTACGGGAGGCAGCAGTGGGGAATATTGCACAATGGGCGCAA
GCCTGATGCAGCCATGCCGCGTGTGTGAAGAAGGCCTTCGGGTTGTAAAGCACTTTCAG
CGAGGAGGAAGGCATAAAGGTTAATAACCTTTGTGATTGACGTTACTCGCAGAAGAAG
CACCGGCTAACTCCGTGCCAGCAGCCGCGGTAATACGGAGGGTGCAAGCGTTAATCGG
AATTACTGGGCGTAAAGCGCACGCAGGCGGTTTGTTAAGTCAGATGTGAAATCCCCGCG
CTTAACGTGGGAACTGCATTTGAAACTGGCAAGCTAGAGTCTTGTAGAGGGGGGTAGA
ATTCCAGGTGTAGCGGTGAAATGCGTAGAGATCTGGAGGAATACCGGTGGCGAAGGCG
GCCCCCTGGACAAAGACTGACGCTCAGGTGCGAAAGCGTGGGGAGCAAACAGGATTA
GATACCCTGGTAGTCCACGCTGTAAACGATGTCGACTTGGAGGTTGTGCCCTTGAGGCG
TGGCTTCCGGAGCTAACGCGTTAAGTCGACCGCCTGGGGAGTACGGCCGCAAGGTTAA
AACTCAAATGAATTGACGGGGGCCCGCACAAGCGGTGGAGCATGTGGTTTAATTCGATG
CAACGCGAAGAACCTTACCTACTCTTGACATCCACGGAATTTAGCAGAGATGCTTTATTA
GTGCCTTCGGGAACCGTGAGACAGGTGCTGCATGGCCGTCGTCAGCTCGTGTTGTGAA
ATGTTGGGTTAAGTCCCGCAACGAGCGCAACCCTTATCCTTTGTTGCCAGCACGTGATG
GTGGGAACTCAAAGGAGACTGCCGGTGATAAACCGGAGGAAGGTGGGGATGACGTCA
AGTCATCATGGCCCTTACGAGTAGGGCTACACACGTGCTACAATGGCAGATACAAAGTG
AAGCGAACTCGCGAGAGCAAGCGGACCACATAAAGTCTGTCGTAGTCCGGATTGGAGT
CTGCAACTCGACTCCATGAAGTCGGAATCGCTAGTAATCGTAGATCAGAATGCTACGGT
GAATACGTTCCCGGGCCTTGTACACACCGCCCGTCACACCATGGGAGTGGGTTGCAAA
AGAAGTAGGTAGCTTAACCTTCGGGAGGGCGCTTACCACTTTGTGATTCATGACTGGGG
pattern 250
CGCTGGCGGCAGGCCTAACACATGCAAGTCGAGCGGCAGCGGGAAGTAGTTTACTACT
TTTCCGGCGAGCGGCGGACGGGTGAGTAATGTCTGGGAAACTGCCTGATGGAGGGGGA
TAACTACTGGAAACGGTAGCTAATACCGCATAACGTCTTCGGACCAAAGTGGGGGACCT
TCGGGCCTCACGCCATCGGATGTGCCCAGATGGGATTAGCTAGTAGGTGGGGTAATGGC
TCACCTAGGCGACGATCCCTAGCTGGTCTGAGAGGATGACCAGCCACACTGGAACTGA
GACACGGTCCAGACTCCTACGGGAGGCAGCAGTGGGGAATATTGCACAATGGGCGCAA
GCCTGATGCAGCCATGCCGCGTGTGTGAAGAAGGCCTTCGGGTTGTAAAGCACTTTCAG
CGAGGAGGAAGGCATAAAGGTTAATAACCTTTGTGATTGACGTTACTCGCAGAAGAAG
CACCGGCTAACTCCGTGCCAGCAGCCGCGGTAATACGGAGGGTGCAAGCGTTAATCGG
AATTACTGGGCGTAAAGCGCACGCAGGCGGTTTGTTAAGTCAGATGTGAAATCCCCGCG

CTTAACGTGGGAACTGCATTTGAAACTGGCAAGCTAGAGTCTTGTAGAGGGGGGTAGA
ATTCCAGGTGTAGCGGTGAAATGCGTAGAGATCTGGAGGAATACCGGTGGCGAAGGCG
GCCCCCTGGACAAAGACTGACGCTCAGGTGCGAAAGCGTGGGGAGCAAACAGGATTA
GATACCCTGGTAGTCCACGCTGTAAACGATGTCGACTTGGAGGTTGTGCCCTTGAGGCG
TGGCTTCCGGAGCTAACGCGTTAAGTCGACCGCCTGGGGAGTACGGCCGCAAGGTTAA
AACTCAAATGAATTGACGGGGGCCCGCACAAGCGGTGGAGCATGTGGTTTAATTCGATG
CAACGCGAAGAACCTTACCTACTCTTGACATCCACGGAATTTAGCAGAGATGCTTTAGT
GCCTTCGGGAACCGTGAGACAGGTGCTGCATGGCTGTCGTCAGCTCGTGTTGTGAAATG
TTGGGTTAAGTCCCGCAACGAGCGCAACCCTTATCCTTTGTTGCCAGCACGTGATGGTG
GGAACTCAAAGGAGACTGCCGGTGATAAACCGGAGGAAGGTGGGGATGACGTCAAGT
CATCATGGCCCTTACGAGTAGGGCTACACACGTGCTACAATGGCAGATACAAAGTGAAG
CGAACTCGCGAGAGCAAGCGGACCACATAAAGTCTGTCGTAGTCCGGATTGGAGTCTG
CAACTCGACTCCATGAAGTCGGAATCGCTAGTAATCGTAGATCAGAATGCTACGGTGAAT
ACGTTCCCGGGCCTTGTACACACCGCCCGTCACACCATGGGAGTGGGTTGCAAAAGAA
GTAGGTAGCTTAACCTTCGGGAGGGCGCTTACCACTTTGTGATTCATGACTGGGG
pattern 251
CGCTGGCGGCAGGCCTAACACATGCAAGTCGAGCGGCAGCGGGAAGTAGTTTACTACT
TTGCCGGCGAGCGGCGGACGGGTGAGTAATGTCTGGGAAACTGCCTGATGGAGGGGGA
TAACTACTGGAAACGGTAGCTAATACCGCATAACGTCTTCGGACCAAAGTGGGGGACCT
TCGGGCCTCACGCCATCGGATGTGCCCAGATGGGATTAGCTAGTAGGTGGGGTAACGGC
TCACCTAGGCGACGATCCCTAGCTGGTCTGAGAGGATGACCAGCCACACTGGAACTGA
GACACGGTCCAGACTCCTACGGGAGGCAGCAGTGGGGAATATTGCACAATGGGCGCAA
GCCTGATGCAGCCATGCCGCGTGTGTGAAGAAGGCCTTCGGGTTGTAAAGCACTTTCAG
CGAGGAGGAAGGCATAAAGGTTAATAACCTTTGTGATTGACGTTACTCGCAGAAGAAG
CACCGGCTAACTCCGTGCCAGCAGCCGCGGTAATACGGAGGGTGCAAGCGTTAATCGG
AATTACTGGGCGTAAAGCGCACGCAGGCGGTTTGTTAAGTCAGATGTGAAATCCCCGCG
CTTAACGTGGGAACTGCATTTGAAACTGGCAAGCTAGAGTCTTGTAGAGGGGGGTAGA
ATTCCAGGTGTAGCGGTGAAATGCGTAGAGATCTGGAGGAATACCGGTGGCGAAGGCG
GCCCCCTGGACAAAGACTGACGCTCAGGTGCGAAAGCGTGGGGAGCAAACAGGATTA
GATACCCTGGTAGTCCACGCTGTAAACGATGTCGACTTGGAGGTTGTGCCCTTGAGGCG
TGGCTTCCGGAGCTAACGCGTTAAGTCGACCGCCTGGGGAGTACGGCCGCAAGGTTAA
AACTCAAATGAATTGACGGGGGCCCGCACAAGCGGTGGAGCATGTGGTTTAATTCGATG
CAACGCGAAGAACCTTACCTACTCTTGACATCCACGGAATTTAGCAGAGATGCTTTAGT
GCCTTCGGGAACCGTGAGACAGGTGCTGCATGGCTGTCGTCAGCTCGTGTTGTGAAATG
TTGGGTTAAGTCCCGCAACGAGCGCAACCCTTATCCTTTGTTGCTAGCACGTAATGGTG
GGAACTCAAAGGAGACTGCCGGTGATAAACCGGAGGAAGGTGGGGATGACGTCAAGT
CATCATGGCCCTTACGAGTAGGGCTACACACGTGCTACAATGGCAGATACAAAGTGAAG
CGAACTCGCGAGAGCAAGCGGACCACATAAAGTCTGTCGTAGTCCGGATTGGAGTCTG
CAACTCGACTCCATGAAGTCGGAATCGCTAGTAATCGTAGATCAGAATGCTACGGTGAAT
ACGTTCCCGGGCCTTGTACACACCGCCCGTCACACCATGGGAGTGGGTTGCAAAAGAA
GTAGGTAGCTTAACCTTCGGGAGGGCGCTTACCACTTTGTGATTCATGACTGGGG
pattern 252
CGCTGGCGGCAGGCCTAACACATGCAAGTCGAGCGGCAGCGGGAAGTAGTTTACTACT
TTGCCGGCGAGCGGCGGACGGGTGAGTAATGTCTGGGAAACTGCCTGATGGAGGGGGA

TAACTACTGGAAACGGTAGCTAATACCGCATAACGTCTTCGGACCAAAGTGGGGGACCT
TCGGGCCTCACGCCATCGGATGTGCCCAGATGGGATTAGCTAGTAGGTGGGGTAACGGC
TCACCTAGGCGACGATCCCTAGCTGGTCTGAGAGGATGACCAGCCACACTGGAACTGA
GACACGGTCCAGACTCCTACGGGAGGCAGCAGTGGGGAATATTGCACAATGGGCGCAA
GCCTGATGCAGCCATGCCGCGTGTGTGAAGAAGGCCTTCGGGTTGTAAAGCACTTTCAG
CGAGGAGGAAGGCATAAAGGTTAATAACCTTTGTGATTGACGTTACTCGCAGAAGAAG
CACCGGCTAACTCCGTGCCAGCAGCCGCGGTAATACGGAGGGTGCAAGCGTTAATCGG
AATTACTGGGCGTAAAGCGCACGCAGGCGGTTTGTTAAGTCAGATGTGAAATCCCCGCG
CTTAACGTGGGAACTGCATTTGAAACTGGCAAGCTAGAGTCTTGTAGAGGGGGGTAGA
ATTCCAGGTGTAGCGGTGAAATGCGTAGAGATCTGGAGGAATACCGGTGGCGAAGGCG
GCCCCCTGGACAAAGACTGACGCTCAGGTGCGAAAGCGTGGGGAGCAAACAGGATTA
GATACCCTGGTAGTCCACGCTGTAAACGATGTCGACTTGGAGGTTGTGCCCTTGAGGCG
TGGCTTCCGGAGCTAACGCGTTAAGTCGACCGCCTGGGGAGTACGGCCGCAAGGTTAA
AACTCAAATGAATTGACGGGGGCCCGCACAAGCGGTGGAGCATGTGGTTTAATTCGATG
CAACGCGAAGAACCTTACCTACTCTTGACATCCACAGAACTTAGCAGAGATGCTTCGGT
GCCTTCGGGAACTGTGAGACAGGTGCTGCATGGCTGTCGTCAGCTCGTGTTGTGAAATG
TTGGGTTAAGTCCCGCAACGAGCGCAACCCTTATCCTTTGTTGCCAGCACGTGATGGTG
GGAACTCAAAGGAGACTGCCGGTGATAAACCGGAGGAAGGTGGGGATGACGTCAAGT
CATCATGGCCCTTACGAGTAGGGCTACACACGTGCTACAATGGCAGATACAAAGTGAAG
CGAACTCGCGAGAGCAAGCGGACCACATAAAGTCTGTCGTAGTCCGGATTGGAGTCTG
CAACTCGACTCCATGAAGTCGGAATCGCTAGTAATCGTAGATCAGAATGCTACGGTGAAT
ACGTTCCCGGGCCTTGTACACACCGCCCGTCACACCATGGGAGTGGGTTGCAAAAGAA
GTAGGTAGCTTAACCTTCGGGAGGGCGCTTACCACTTTGTGATTCATGACTGGGG
pattern 253
CGCTGGCGGCAGGCCTAACACATGCAAGTCGAGCGGCAGCGGGAAGTAGTTTACTACT
TTGCCGGCGAGCGGCGGACGGGTGAGTAATGTCTGGGGATCTGCCTGATGGAGGGGGA
TAACTACTGGAAACGGTAGCTAATACCGCATGACCTCGCAAGAGCAAAGTGGGGGACC
TTAGGGCCTCACGCCATCGGATGAACCCAGATGGGATTAGCTAGTAGGTGGGGTAATGG
CTCACCTAGGCGACGATCCCTAGCTGGTCTGAGAGGATGACCAGCCACACTGGAACTG
AGACACGGTCCAGACTCCTACGGGAGGCAGCAGTGGGGAATATTGCACAATGGGCGCA
AGCCTGATGCAGCCATGCCGCGTGTGTGAAGAAGGCCTTCGGGTTGTAAAGCACTTTCA
GCGAGGAGGAAGGGGTTGAGTTTAATACGCTCAATCATTGACGTTACTCGCAGAAGAA
GCACCGGCTAACTCCGTGCCAGCAGCCGCGGTAATACGGAGGGTGCAAGCGTTAATCG
GAATTACTGGGCGTAAAGCGCACGCAGGCGGTTTGTTAAGTCAGATGTGAAATCCCCGC
GCTTAACGTGGGAACTGCATTTGAAACTGGCAAGCTAGAGTCTTGTAGAGGGGGGTAG
AATTCCAGGTGTAGCGGTGAAATGCGTAGAGATCTGGAGGAATACCGGTGGCGAAGGC
GGCCCCCTGGACAAAGACTGACGCTCAGGTGCGAAAGCGTGGGGAGCAAACAGGATT
AGATACCCTGGTAGTCCACGCCGTAAACGATGTCGACTTGGAGGTTGTGCCCTTGAGGC
GTGGCTTCCGGAGCTAACGCGTTAAGTCGACCGCCTGGGGAGTACGGCCGCAAGGTTA
AAACTCAAATGAATTGACGGGGGCCCGCACAAGCGGTGGAGCATGTGGTTTAATTCGAT
GCAACGCGAAGAACCTTACCTACTCTTGACATCCACAGAATTTGGCAGAGATGCTAAAG
TGCCTTCGGGAACTGTGAGACAGGTGCTGCATGGCTGTCGTCAGCTCGTGTTGTGAAAT
GTTGGGTTAAGTCCCGCAACGAGCGCAACCCTTATCCTTTGTTGCCAGCACGTAATGGT
GGGAACTCAAGGGAGACTGCCGGTGACAAACCGGAGGAAGGTGGGGATGACGTCAAG

TCATCATGGCCCTTACGAGTAGGGCTACACACGTGCTACAATGGCAGATACAAAGTGAA
GCGAACTCGCGAGAGCCAGCGGACCACATAAAGTCTGTCGTAGTCCGGATTGGAGTCT
GCAACTCGACTCCATGAAGTCGGAATCGCTAGTAATCGTAGATCAGAATGCTACGGTGA
ATACGTTCCCGGGCCTTGTACACACCGCCCGTCACACCATGGGAGTGGGTTGCAAAAG
AAGTAGGTAGCTTAACCTTCGGGAGGGCGCTTACCACTTTGTGATTCATGACTGGGG
pattern 254
CGCTGGCGGCAGGCCTAACACATGCAAGTCGAGCGGCAGCGGGAAGTAGTTTACTACT
TTGCCGGCGAGCGGCGGACGGGTGAGTAATGTCTGGGGATCTGCCTGATGGAGGGGGA
TAACTACTGGAAACGGTAGCTAATACCGCATGACCTCGCAAGAGCAAAGTGGGGGACC
TTAGGGCCTCACGCCATCGGATGAACCCAGATGGGATTAGCTAGTAGGTGGGGTAATGG
CTCACCTAGGCGACGATCCCTAGCTGGTCTGAGAGGATGACCAGCCACACTGGAACTG
AGACACGGTCCAGACTCCTACGGGAGGCAGCAGTGGGGAATATTGCACAATGGGCGCA
AGCCTGATGCAGCCATGCCGCGTGTGTGAAGAAGGCCTTCGGGTTGTAAAGCACTTTCA
GCGAGGAGGAAGGGGTTGAGTTTAATACACTCAATCATTGACGTTACTCGCAGAAGAA
GCACCGGCTAACTCCGTGCCAGCAGCCGCGGTAATACGGAGGGTGCAAGCGTTAATCG
GAATTACTGGGCGTAAAGCGCACGCAGGCGGTTTGTTAAGTCAGATGTGAAATCCCCGC
GCTTAACGTGGGAACTGCATTTGAAACTGGCAAGCTAGAGTCTTGTAGAGGGGGGTAG
AATTCCAGGTGTAGCGGTGAAATGCGTAGAGATCTGGAGGAATACCGGTGGCGAAGGC
GGCCCCCTGGACAAAGACTGACGCTCAGGTGCGAAAGCGTGGGGAGCAAACAGGATT
AGATACCCTGGTAGTCCACGCTGTAAACGATGTCGACTTGGAGGTTGTGCCCTTGAGGC
GTGGCTTCCGGAGCTAACGCGTTAAGTCGACCGCCTGGGGAGTACGGCCGCAAGGTTA
AAACTCAAATGAATTGACGGGGGCCCGCACAAGCGGTGGAGCATGTGGTTTAATTCGAT
GCAACGCGAAGAACCTTACCTACTCTTGACATCCACAGAATTTGGCAGAGATGCTAAAG
TGCCTTCGGGAACTGTGAGACAGGTGCTGCATGGCTGTCGTCAGCTCGTGTTGTGAAAT
GTTGGGTTAAGTCCCGCAACGAGCGCAACCCTTATCCTTTGTTGCCAGCACGTAATGGT
GGGAACTCAAGGGAGACTGCCGGTGACAAACCGGAGGAAGGTGGGGATGACGTCAAG
TCATCATGGCCCTTACGAGTAGGGCTACACACGTGCTACAATGGCAGATACAAAGTGAA
GCGAACTCGCGAGAGCCAGCGGACCACATAAAGTCTGTCGTAGTCCGGATTGGAGTCT
GCAACTCGACTCCATGAAGTCGGAATCGCTAGTAATCGTAGATCAGAATGCTACGGTGA
ATACGTTCCCGGGCCTTGTACACACCGCCCGTCACACCATGGGAGTGGGTTGCAAAAG
AAGTAGGTAGCTTAACCTTCGGGAGGGCGCTTACCACTTTGTGATTCATGACTGGGG
pattern 255
CGCTGGCGGCAGGCCTAACACATGCAAGTCGAGCGGCAGCGGAAAGTAGCTTGCTACT
TTGCCGGCGAGCGGCGGACGGGTGAGTAATGTCTGGGGATCTGCCTGATGGAGGGGGA
TAACTACTGGAAACGGTAGCTAATACCGCATGACCTCGAAAGAGCAAAGTGGGGGACC
TTCGGGCCTCACGCCATCGGATGAACCCAGATGGGATTAGCTAGTAGGTGGGGTAATGG
CTCACCTAGGCGACGATCCTTAGCTGGTCTGAGAGGATGACCAGCCACACTGGAACTG
AGACACGGTCCAGACTCCTACGGGAGGCAGCAGTGGGGAATATTGCACAATGGGCGCA
AGCCTGATGCAGCCATGCCGCGTGTGTGAAGAAGGCCTTCGGGTTGTAAAGCACTTTCA
GCGAGGAGGAAGGCATTGTGGTTAATAACCGCAGTGATTGACGTTACTCGCAGAAGAA
GCACCGGCTAACTCCGTGCCAGCAGCCGCGGTAATACGGAGGGTGCAAGCGTTAATCG
GAATTACTGGGCGTAAAGCGCACGCAGGCGGTTTGTTAAGTCAGATGTGAAATCCCCGC
GCTTAACGTGGGAACTGCATTTGAAACTGGCAAGCTAGAGTCTTGTAGAGGGGGGTAG
AATTCCAGGTGTAGCGGTGAAATGCGTAGAGATCTGGAGGAATACCGGTGGCGAAGGC

GGCCCCCTGGACAAAGACTGACGCTCAGGTGCGAAAGCGTGGGGAGCAAACAGGATT
AGATACCCTGGTAGTCCACGCTGTAAACGATGTCGACTTGGAGGTTGTGCCCTTGAGGC
GTGGCTTCCGGAGCTAACGCGTTAAGTCGACCGCCTGGGGAGTACGGCCGCAAGGTTA
AAACTCAAATGAATTGACGGGGGCCCGCACAAGCGGTGGAGCATGTGGTTTAATTCGAT
GCAACGCGAAGAACCTTACCTACTCTTGACATCCACAGAACTTAGCAGAGATGCTTCGG
TGCCTTCGGGAACTGTGAGACAGGTGCTGCATGGCTGTCGTCAGCTCGTGTTGTGAAAT
GTTGGGTTAAGTCCCGCAACGAGCGCAACCCTCGTCCTATGTTGCCAGCACGTTATGGT
GGGAACTCATGGGATACTGCCGTGGTCAACACGGAGGAAGGTGGGGATGACGTCAAAT
CATCATGCCCCTTATGTCTTGGGCTTCACGCATGCTACAATGGCAGATACAAAGTGAAGC
GAACTCGCGAGAGCAAGCGGACCACATAAAGTCTGTCGTAGTCCGGATTGGAGTCTGC
AACTCGACTCCATGAAGTCGGAATCGCTAGTAATCGTAGATCAGAATGCTACGGTGAAT
ACGTTCCCGGGCCTTGTACACACCGCCCGTCACACCATGGGAGTGGGTTGCAAAAGAA
GTAGGTAGCTTAACCTTCGGGAGGGCGCTTACCACTTTGTGATTCATGACTGGGG
pattern 256
CGCTGGCGGCAGGCCTAACACATGCAAGTCGAGCGGCAGCGGGAAGTAGTTTACTACT
TTGCCGGCGAGCGGCGGACGGGTGAGTAATGTCTGGGAAACTGCCTGATGGAGGGGGA
TAACTACTGGAAACGGTAGCTAATACCGCATGACCTCGTAAGAGCAAAGTGGGGGACCT
TCGGGCCTCACGCCATCGGATGTGCCCAGATGGGATTAGCTAGTAGGTGGGGTAATGGC
TCACCTAGGCGACGATCCCTAGCTGGTCTGAGAGGATGACCAGCCACACTGGAACTGA
GACACGGTCCAGACTCCTACGGGAGGCAGCAGTGGGGAATATTGCACAATGGGCGCAA
GCCTGATGCAGCCATGCCGCGTGTGTGAAGAAGGCCTTCGGGTTGTAAAGCACTTTCAG
CGAGGAGGAAGGCAGTCGTGTTAATAGCACGGTTGATTGACGTTACTCGCAGAAGAAG
CACCGGCTAACTCCGTGCCAGCAGCCGCGGTAATACGGAGGGTGCAAGCGTTAATCGG
AATTACTGGGCGTAAAGCGCACGCAGGCGGTTTGTTAAGTCAGATGTGAAATCCCCGCG
CTTAACGTGGGAACTGCATTTGAAACTGGCAAGCTAGAGTCTTGTAGAGGGGGGTAGA
ATTCCAGGTGTAGCGGTGAAATGCGTAGAGATCTGGAGGAATACCGGTGGCGAAGGCG
GCCCCCTGGACAAAGACTGACGCTCAGGTGCGAAAGCGTGGGGAGCAAACAGGATTA
GATACCCTGGTAGTCCACGCTGTAAACGATGTCGACTTGGAGGTTGTGCCCTTGAGGTG
TGGCTTCCGGAGCTAACGCGTTAAGTCGACCGCCTGGGGAGTACGGCCGCAAGGTTAA
AACTCAAATGAATTGACGGGGGCCCGCACAAGCGGTGGAGCATGTGGTTTAATTCGATG
CAACGCGAAGAACCTTACCTACTCTTGACATCCACAGAACTTAGCAGAGATGCTTCGGT
GCCTTCGGGAACTGTGAGACAGGTGCTGCATGGCTGTCGTCAGCTCGTGTTGTGAAATG
TTGGGTTAAGTCCCGCAACGAGCGCAACCCTTATCCTTTGTTGCCAGCACGTAATGGTG
GGAACTCAAGGGAGACTGCCGGTGACAAACCGGAGGAAGGTGGGGATGACGTCAAGT
CATCATGGCCCTTACGAGTAGGGCTACACACGTGCTACAATGGCAGATACAAAGTGAAG
CGAACTCGCGAGAGCAAGCGGACCACATAAAGTCTGTCGTAGTCCGGATTGGAGTCTG
CAACTCGACTCCATGAAGTCGGAATCGCTAGTAATCGTAGATCAGAATGCTACGGTGAAT
ACGTTCCCGGGCCTTGTACACACCGCCCGTCACACCATGGGAGTGGGTTGCAAAAGAA
GTAGGTAGCTTAACCTTCGGGAGGGCGCTTACCACTTTGTGATTCATGACTGGGG
pattern 257
CGCTGGCGGCAGGCCTAACACATGCAAGTCGAGCGGCAGCGGGAAGTAGTTTACTACT
TTGCCGGCGAGCGGCGGACGGGTGAGTAATGTCTGGGAAACTGCCTGATGGAGGGGGA
TAACTACTGGAAACGGTAGCTAATACCGCATAACGTCTTCGGACCAAAGTGGGGGACCT
TCGGGCCTCACGCCATCGGATGTGCCCAGATGGGATTAGCTAGTAGGTGGGGTAATGGC

TCACCTAGGCGACGATCCCTAGCTGGTCTGAGAGGATGACCAGCCACACTGGAACTGA
GACACGGTCCAGACTCCTACGGGAGGCAGCAGTGGGGAATATTGCACAATGGGCGCAA
GCCTGATGCAGCCATGCCGCGTGTGTGAAGAAGGCCTTCGGGTTGTAAAGCACTTTCAG
CGAGGAGGAAGGCATGAAGGTTAATAACCTTTGTGATTGACGTTACTCGCAGAAGAAG
CACCGGCTAACTCCGTGCCAGCAGCCGCGGTAATACGGAGGGTGCAAGCGTTAATCGG
AATTACTGGGCGTAAAGCGCACGCAGGCGGTTTGTTAAGTCAGATGTGAAATCCCCGCG
CTTAACGTGGGAACTGCATTTGAAACTGGCAAGCTAGAGTCTTGTAGAGGGGGGTAGA
ATTCCAGGTGTAGCGGTGAAATGCGTAGAGATCTGGAGGAATACCGGTGGCGAAGGCG
GCCCCCTGGACAAAGACTGACGCTCAGGTGCGAAAGCGTGGGGAGCAAACAGGATTA
GATACCCTGGTAGTCCACGCTGTAAACGATGTCGACTTGGAGGTTGTGCCCTTGAGGCG
TGGCTTCCGGAGCTAACGCGTTAAGTCGACCGCCTGGGGAGTACGGCCGCAAGGTTAA
AACTCAAATGAATTGACGGGGGCCCGCACAAGCGGTGGAGCATGTGGTTTAATTCGATG
CAACGCGAAGAACCTTACCTACTCTTGACATCCACAGAACTTAGCAGAGATGCTTCGGT
GCCTTCGGGAACTGTGAGACAGGTGCTGCATGGCTGTCGTCAGCTCGTGTTGTGAAATG
TTGGGTTAAGTCCCGCAACGAGCGCAACCCTTATCCTTTGTTGCCAGCGAGTAATGTCG
GGAACTCAAAGGAGACTGCCGGTGATAAACCGGAGGAAGGTGGGGATGACGTCAAGT
CATCATGGCCCTTACGAGTAGGGCTACACACGTGCTACAATGGCAGATACAAAGTGAAG
CGAACTCGCGAGAGCAAGCGGACCACATAAAGTCTGTCGTAGTCCGGATTGGAGTCTG
CAACTCGACTCCATGAAGTCGGAATCGCTAGTAATCGTAGATCAGAATGCTACGGTGAAT
ACGTTCCCGGGCCTTGTACACACCGCCCGTCACACCATGGGAGTGGGTTGCAAAAGAA
GTAGGTAGCTTAACCTTCGGGAGGGCGCTTACCACTTTGTGATTCATGACTGGGG
pattern 258
CGCTGGCGGCAGGCCTAACACATGCAAGTCGAGCGGCAGCGGGAAGTAGTTTACTACT
TTGCCGGCGAGCGGCGGACGGGTGAGTAATGTCTGGGAAACTGCCTGATGGAGGGGGA
TAACTACTGGAAACGGTAGCTAATACCGCATGACCTCGTAAGAGCAAAGTGGGGGACCT
TCGGGCCTCACGCCATCGGATGTGCCCAGATGGGATTAGCTAGTAGGTGGGGTAATGGC
TCACCTAGGCGACGATCCCTAGCTGGTCTGAGAGGATGACCAGCCACACTGGAACTGA
GACACGGTCCAGACTCCTACGGGAGGCAGCAGTGGGGAATATTGCACAATGGGCGCAA
GCCTGATGCAGCCATGCCGCGTGTGTGAAGAAGGCCTTCGGGTTGTAAAGCACTTTCAG
CGAGGAGGAAGGCAGTCGTGTTAATAGCACGGTTGATTGACGTTACTCGCAGAAGAAG
CACCGGCTAACTCCGTGCCAGCAGCCGCGGTAATACGGAGGGTGCAAGCGTTAATCGG
AATTACTGGGCGTAAAGCGCACGCAGGCGGTTTGTTAAGTCAGATGTGAAATCCCCGCG
CTTAACGTGGGAACTGCATTTGAAACTGGCAAGCTAGAGTCTTGTAGAGGGGGGTAGA
ATTCCAGGTGTAGCGGTGAAATGCGTAGAGATCTGGAGGAATACCGGTGGCGAAGGCG
GCCCCCTGGACAAAGACTGACGCTCAGGTGCGAAAGCGTGGGGAGCAAACAGGATTA
GATACCCTGGTAGTCCACGCTGTAAACGATGTCGACTTGGAGGTTGTGCCCTTGAGGCG
TGGCTTCCGGAGCTAACGCGTTAAGTCGACCGCCTGGGGAGTACGGCCGCAAGGTTAA
AACTCAAATGAATTGACGGGGGCCCGCACAAGCGGTGGAGCATGTGGTTTAATTCGATG
CAACGCGAAGAACCTTACCTACTCTTGACATCCACGGAATTTAGCAGAGATGCTTAGGT
GCCTTCGGGAACTGTGAGACAGGTGCTGCATGGCTGTCGTCAGCTCGTGTTGTGAAATG
TTGGGTTAAGTCCCGCAACGAGCGCAACCCTTATCCTTTGTTGCCAGCACGTAATGGTG
GGAACTCAAGGGAGACTGCCGGTGACAAACCGGAGGAAGGTGGGGATGACGTCAAGT
CATCATGGCCCTTACGAGTAGGGCTACACACGTGCTACAATGGCAGATACAAAGTGAAG
CGAACTCGCGAGAGCAAGCGGACCACATAAAGTCTGTCGTAGTCCGGATTGGAGTCTG

CAACTCGACTCCATGAAGTCGGAATCGCTAGTAATCGTAGATCAGAATGCTACGGTGAAT
ACGTTCCCGGGCCTTGTACACACCGCCCGTCACACCATGGGAGTGGGTTGCAAAAGAA
GTAGGTAGCTTAACCTTCGGGAGGGCGCTTACCACTTTGTGATTCATGACTGGGG
pattern 259
CGCTGGCGGCAGGCCTAACACATGCAAGTCGAGCGGCAGCGGGAAGTAGTTTACTACT
TTGCCGGCGAGCGGCGGACGGGTGAGTAATGTCTGGGAAACTGCCTGATGGAGGGGGA
TAACTACTGGAAACGGTAGCTAATACCGCATGACCTCGCAAGAGCAAAGTGGGGGACC
TTCGGGCCTCACGCCATCGGATGTGCCCAGATGGGATTAGCTAGTAGGTGGGGTAATGG
CTCACCTAGGCGACGATCCCTAGCTGGTCTGAGAGGATGACCAGCCACACTGGAACTG
AGACACGGTCCAGACTCCTACGGGAGGCAGCAGTGGGGAATATTGCACAATGGGCGCA
AGCCTGATGCAGCCATGCCGCGTGTGTGAAGAAGGCCTTCGGGTTGTAAAGCACTTTCA
GCGAGGAGGAAGGCAGTCGTGTTAATAGCACGGTTGATTGACGTTACTCGCAGAAGAA
GCACCGGCTAACTCCGTGCCAGCAGCCGCGGTAATACGGAGGGTGCAAGCGTTAATCG
GAATTACTGGGCGTAAAGCGCACGCAGGCGGTTTGTTAAGTCAGATGTGAAATCCCCGC
GCTTAACGTGGGAACTGCATTTGAAACTGGCAAGCTAGAGTCTTGTAGAGGGGGGTAG
AATTCCAGGTGTAGCGGTGAAATGCGTAGAGATCTGGAGGAATACCGGTGGCGAAGGC
GGCCCCCTGGACAAAGACTGACGCTCAGGTGCGAAAGCGTGGGGAGCAAACAGGATT
AGATACCCTGGTAGTCCACGCTGTAAACGATGTCGACTTGGAGGTTGTGCCCTTGAGGC
GTGGCTTCCGGAGCTAACGCGTTAAGTCGACCGCCTGGGGAGTACGGCCGCAAGGTTA
AAACTCAAATGAATTGACGGGGGCCCGCACAAGCGGTGGAGCATGTGGTTTAATTCGAT
GCAACGCGAAGAACCTTACCTACTCTTGACATCCACAGAACTTAGCAGAGATGCTTAGG
TGCCTTCGGGAACTGTGAGACAGGTGCTGCATGGCTGTCGTCAGCTCGTGTTGTGAAAT
GTTGGGTTAAGTCCCGCAACGAGCGCAACCCTTATCCTTTGTTGCCAGCACGTAATGGT
GGGAACTCAAGGGAGACTGCCGGTGACAAACCGGAGGAAGGTGGGGATGACGTCAAG
TCATCATGGCCCTTACGAGTAGGGCTACACACGTGCTACAATGGCAGATACAAAGTGAA
GCGAACTCGCGAGAGCAAGCGGACCACATAAAGTCTGTCGTAGTCCGGATTGGAGTCT
GCAACTCGACTCCATGAAGTCGGAATCGCTAGTAATCGTAGATCAGAATGCTACGGTGA
ATACGTTCCCGGGCCTTGTACACACCGCCCGTCACACCATGGGAGTGGGTTGCAAAAG
AAGTAGGTAGCTTAACCTTCGGGAGGGCGCTTACCACTTTGTGATTCATGACTGGGG
pattern 260
CGCTGGCGGCAGGCCTAACACATGCAAGTCGAGCGGCAGCGGGAAGTAGTTTACTACT
TTGCCGGCGAGCGGCGGACGGGTGAGTAATGTCTGGGAAACTGCCTGATGGAGGGGGA
TAACTACTGGAAACGGTAGCTAATACCGCATGACCTCGTAAGAGCAAAGTGGGGGACCT
TCGGGCCTCACGCCATCGGATGTGCCCAGATGGGATTAGCTAGTAGGTGAGGTAATGGC
TCACCTAGGCGACGATCCCTAGCTGGTCTGAGAGGATGACCAGCCACACTGGAACTGA
GACACGGTCCAGACTCCTACGGGAGGCAGCAGTGGGGAATATTGCACAATGGGCGCAA
GCCTGATGCAGCCATGCCGCGTGTGTGAAGAAGGCCTTCGGGTTGTAAAGCACTTTCAG
CGAGGAGGAAGGCAGTCGTGTTAATAGCACGATTGATTGACGTTACTCGCAGAAGAAG
CACCGGCTAACTCCGTGCCAGCAGCCGCGGTAATACGGAGGGTGCAAGCGTTAATCGG
AATTACTGGGCGTAAAGCGCACGCAGGCGGTTTGTTAAGTCAGATGTGAAATCCCCGCG
CTTAACGTGGGAACTGCATTTGAAACTGGCAAGCTAGAGTCTTGTAGAGGGGGGTAGA
ATTCCAGGTGTAGCGGTGAAATGCGTAGAGATCTGGAGGAATACCGGTGGCGAAGGCG
GCCCCCTGGACAAAGACTGACGCTCAGGTGCGAAAGCGTGGGGAGCAAACAGGATTA
GATACCCTGGTAGTCCACGCTGTAAACGATGTCGACTTGGAGGTTGTGCCCTTGAGGCG

TGGCTTCCGGAGCTAACGCGTTAAGTCGACCGCCTGGGGAGTACGGCCGCAAGGTTAA
AACTCAAATGAATTGACGGGGGCCCGCACAAGCGGTGGAGCATGTGGTTTAATTCGATG
CAACGCGAAGAACCTTACCTACTCTTGACATCCACAGAACTTAGCAGAGATGCTTCGGT
GCCTTCGGGAACTGTGAGACAGGTGCTGCATGGCTGTCGTCAGCTCGTGTTGTGAAATG
TTGGGTTAAGTCCCGCAACGAGCGCAACCCTTATCCTTTGTTGCCAGCACGTAATGGTG
GGAACTCAAGGGAGACTGCCGGTGACAAACCGGAGGAAGGTGGGGATGACGTCAAGT
CATCATGGCCCTTACGAGTAGGGCTACACACGTGCTACAATGGCAGATACAAAGTGAAG
CGAACTCGCGAGAGCAAGCGGACCACATAAAGTCTGTCGTAGTCCGGATTGGAGTCTG
CAACTCGACTCCATGAAGTCGGAATCGCTAGTAATCGTAGATCAGAATGCTACGGTGAAT
ACGTTCCCGGGCCTTGTACACACCGCCCGTCACACCATGGGAGTGGGTTGCAAAAGAA
GTAGGTAGCTTAACCTTCGGGAGGGCGCTTACCACTTTGTGATTCATGACTGGGG
pattern 261
CGCTGGCGGCAGGCCTAACACATGCAAGTCGAGCGGCAGCGGAAAGTAGCTTGCTACT
TTGCCGGCGAGCGGCGGACGGGTGAGTAATGTCTGGGGATCTGCCTGATGGAGGGGGA
TAACTACTGGAAACGGTAGCTAATACCGCATGACCTCGAAAGAGCAAAGTGGGGGACC
TTCGGGCCTCACGCCATCGGATGAACCCAGATGGGATTAGCTAGTAGGTGGGGTAATGG
CTCACCTAGGCGACGATCCCTAGCTGGTCTGAGAGGATGACCAGCCACACTGGAACTG
AGACACGGTCCAGACTCCTACGGGAGGCAGCAGTGGGGAATATTGCACAATGGGCGCA
AGCCTGATGCAGCCATGCCGCGTGTGTGAAGAAGGCCTTCGGGTTGTAAAGCACTTTCA
GCGAGGAGGAAGGCATTGTGGTTAATAACCGCAGTGATTGACATTACTCGCAGAAGAA
GCACCGGCTAACTCCGTGCCAGCAGCCGCGGTAATACGGAGGGTGCAAGCGTTAATCG
GAATTACTGGGCGTAAAGCGCACGCAGGCGGTTTGTTAAGTCAGATGTGAAATCCCCGC
GCTTAACGTGGGAACTGCATTTGAAACTGGCAAGCTAGAGTCTTGTAGAGGGGGGTAG
AATTCCAGGTGTAGCGGTGAAATGCGTAGAGATCTGGAGGAATACCGGTGGCGAAGGC
GGCCCCCTGGACAAAGACTGACGCTCAGGTGCGAAAGCGTGGGGAGCAAACAGGATT
AGATACCCTGGTAGTCCACGCTGTAAACGATGTCGACTTGGAGGTTGTGCCCTTGAGGC
GTGGCTTCCGGAGCTAACGCGTTAAGTCGACCGCCTGGGGAGTACGGCCGCAAGGTTA
AAACTCAAATGAATTGACGGGGGCCCGCACAAGCGGTGGAGCATGTGGTTTAATTCGAT
GCAACGCGAAGAACCTTACCTACTCTTGACATCCACGGAATTTAGCAGAGATGCTTTAG
TGCCTTCGGGAACCGTGAGACAGGTGCTGCATGGCTGTCGTCAGCTCGTGTTGTGAAAT
GTTGGGTTAAGTCCCGCAACGAGCGCAACCCTTATCCTTTGTTGCCAGCACGTAATGGT
GGGAACTCAAGGGAGACTGCCGGTGACAAACCGGAGGAAGGTGGGGATGACGTCAAG
TCATCATGGCCCTTACGAGTAGGGCTACACACGTGCTACAATGGCAGATACAAAGTGAA
GCGAACTCGCGAGAGCAAGCGGACCACATAAAGTCTGTCGTAGTCCGGATTGGAGTCT
GCAACTCGACTCCATGAAGTCGGAATCGCTAGTAATCGTAGATCAGAATGCTACGGTGA
ATACGTTCCCGGGCCTTGTACACACCGCCCGTCACACCATGGGAGTGGGTTGCAAAAG
AAGTAGGTAGCTTAACCTTCGGGAGGGCGCTTACCACTTTGTGATTCATGACTGGGG
pattern 262
CGCTGGCGGCAGGCCTAACACATGCAAGTCGAGCGGCAGCGGGAAGTAGTTTACTACT
TTGCCGGCGAGCGGCGGACGGGTGAGTAATGTCTGGGAAACTGCCTGATGGAGGGGGA
TAACTACTGGAAACGGTAGCTAATACCGCATGACCTCGCAAGAGCAAAGTGGGGGACC
TTAGGGCCTCACGCCATCGGATGTGCCCAGATGGGATTAGCTAGTAGGTGGGGTAATGG
CTCACCTAGGCGACGATCCCTAGCTGGTCTGAGAGGATGACCAGCCACACTGGAACTG
AGACACGGTCCAGACTCCTACGGGAGGCAGCAGTGGGGAATATTGCACAATGGGCGCA

AGCCTGATGCAGCCATGCCGCGTGTGTGAAGAAGGCCTTCGGGTTGTAAAGTACTTTCA
GCGAGGAGGAAGGCGATCGTGTTAATAGCACGGTTGATTGACGTTACTCGCAGAAGAA
GCACCGGCTAACTCCGTGCCAGCAGCCGCGGTAATACGGAGGGTGCAAGCGTTAATCG
GAATTACTGGGCGTAAAGCGCACGCAGGCGGTTTGTTAAGTCAGATGTGAAATCCCCGC
GCTTAACGTGGGAACTGCATTTGAAACTGGCAAGCTAGAGTCTTGTAGAGGGGGGTAG
AATTCCAGGTGTAGCGGTGAAATGCGTAGAGATCTGGAGGAATACCGGTGGCGAAGGC
GGCCCCCTGGACAAAGACTGACGCTCAGGTGCGAAAGCGTGGGGAGCAAACAGGATT
AGATACCCTGGTAGTCCACGCTGTAAACGATGTCGACTTGGAGGTTGTGCCCTTGAGGC
GTGGCTTCCGGAGCTAACGCGTTAAGTCGACCGCCTGGGGAGTACGGCCGCAAGGTTA
AAACTCAAATGAATTGACGGGGGCCCGCACAAGCGGTGGAGCATGTGGTTTAATTCGAT
GCAACGCGAAGAACCTTACCTACTCTTGACATCCACAGAACTTAGCAGAGATGCTTCGG
TGCCTTCGGGAACTGTGAGACAGGTGCTGCATGGCTGTCGTCAGCTCGTGTTGTGAAAT
GTTGGGTTAAGTCCCGCAACGAGCGCAACCCTTATCCTTTGTTGCCAGCACGTAATGGT
GGGAACTCAAGGGAGACTGCCGGTGACAAACCGGAGGAAGGTGGGGATGACGTCAAG
TCATCATGGCCCTTACGAGTAGGGCTACACACGTGCTACAATGGCAGATACAAAGTGAA
GCGAACTCGCGAGAGCAAGCGGACCACATAAAGTCTGTCGTAGTCCGGATTGGAGTCT
GCAACTCGACTCCATGAAGTCGGAATCGCTAGTAATCGTAGATCAGAATGCTACGGTGA
ATACGTTCCCGGGCCTTGTACACACCGCCCGTCACACCATGGGAGTGGGTTGCAAAAG
AAGTAGGTAGCTTAACCTTCGGGAGGGCGCTTACCACTTTGTGATTCATGACTGGGG
pattern 263
CGCTGGCGGCAGGCCTAACACATGCAAGTCGAGCGGCAGCGGGAAGTAGTTTACTACT
TTGCCGGCGAGCGGCGGACGGGTGAGTAATGTCTGGGAAACTGCCTGATGGAGGTGGA
TAACTACTGGAAACGGTAGCTAATACCGCATGACCTCGCAAGAGCAAAGTGGGGGACC
TTAGGGCCTCACGCCATCGGATGTGCCCAGATGGGATTAGCTAGTAGGTGGGGTAATGG
CTCACCTAGGCGACGATCCCTAGCTGGTCTGAGAGGATGACCAGCCACACTGGAACTG
AGACACGGTCCAGACTCCTACGGGAGGCAGCAGTGGGGAATATTGCACAATGGGCGCA
AGCCTGATGCAGCCATGCCGCGTGTGTGAAGAAGGCCTTCGGGTTGTAAAGCACTTTCA
GCGAGGAGGAAGGCGATCGTGTTAATAGCACGGTTGATTGACGTTACTCGCAGAAGAA
GCACCGGCTAACTCCGTGCCAGCAGCCGCGGTAATACGGAGGGTGCAAGCGTTAATCG
GAATTACTGGGCGTAAAGCGCACGCAGGCGGTTTGTTAAGTCAGATGTGAAATCCCCGC
GCTTAACGTGGGAACTGCATTTGAAACTGGCAAGCTAGAGTCTTGTAGAGGGGGGTAG
AATTCCAGGTGTAGCGGTGAAATGCGTAGAGATCTGGAGGAATACCGGTGGCGAAGGC
GGCCCCCTGGACAAAGACTGACGCTCAGGTGCGAAAGCGTGGGGAGCAAACAGGATT
AGATACCCTGGTAGTCCACGCTGTAAACGATGTCGACTTGGAGGTTGTGCCCTTGAGGC
GTGGCTTCCGGAGCTAACGCGTTAAGTCGACCGCCTGGGGAGTACGGCCGCAAGGTTA
AAACTCAAATGAATTGACGGGGGCCCGCACAAGCGGTGGAGCATGTGGTTTAATTCGAT
GCAACGCGAAGAACCTTACCTACTCTTGACATCCACAGAACTTAGCAGAGATGCTTCGG
TGCCTTCGGGAACTGTGAGACAGGTGCTGCATGGCTGTCGTCAGCTCGTGTTGTGAAAT
GTTGGGTTAAGTCCCGCAACGAGCGCAACCCTTATCCTTTGTTGCCAGCACGTAATGGT
GGGAACTCAAGGGAGACTGCCGGTGACAAACCGGAGGAAGGTGGGGATGACGTCAAG
TCATCATGGCCCTTACGAGTAGGGCTACACACGTGCTACAATGGCAGATACAAAGTGAA
GCGAACTCGCGAGAGCAAGCGGACCACATAAAGTCTGTCGTAGTCCGGATTGGAGTCT
GCAACTCGACTCCATGAAGTCGGAATCGCTAGTAATCGTAGATCAGAATGCTACGGTGA
ATACGTTCCCGGGCCTTGTACACACCGCCCGTCACACCATGGGAGTGGGTTGCAAAAG

AAGTAGGTAGCTTAACCTTCGGGAGGGCGCTTACCACTTTGTGATTCATGACTGGGG
pattern 264
CGCTGGCGGCAGGCCTAACACATGCAAGTCGAGCGGCAGCGGGAAGTAGTTTACTACT
TCGCCGGCGAGCGGCGGACGGGTGAGTAATGTCTGGGAAACTGCCTGATGGAGGGGGA
TAACTACTGGAAACGGTAGCTAATACCGCATGACCTCGCAAGAGCAAAGTGGGGGACC
TTAGGGCCTCACGCCATCGGATGTGCCCAGATGGGATTAGCTAGTAGGTGGGGTAATGG
CTCACCTAGGCGACGATCCCTAGCTGGTCTGAGAGGATGACCAGCCACACTGGAACTG
AGACACGGTCCAGACTCCTACGGGAGGCAGCAGTGGGGAATATTGCACAATGGGCGCA
AGCCTGATGCAGCCATGCCGCGTGTGTGAAGAAGGCCTTCGGGTTGTAAAGCACTTTCA
GCGAGGAGGAAGGCAATCGTGTTAATAGCACGGTTGATTGACGTTACTCGCAGAAGAA
GCACCGGCTAACTCCGTGCCAGCAGCCGCGGTAATACGGAGGGTGCAAGCGTTAATCG
GAATTACTGGGCGTAAAGCGCACGCAGGCGGTTTGTTAAGTCAGATGTGAAATCCCCGC
GCTTAACGTGGGAACTGCATTTGAAACTGGCAAGCTAGAGTCTTGTAGAGGGGGGTAG
AATTCCAGGTGTAGCGGTGAAATGCGTAGAGATCTGGAGGAATACCGGTGGCGAAGGC
GGCCCCCTGGACAAAGACTGACGCTCAGGTGCGAAAGCGTGGGGAGCAAACAGGATT
AGATACCCTGGTAGTCCACACTGTAAACGATGTCGACTTGGAGGTTGTGCCCTTGAGGC
GTGGCTTCCGGAGCTAACGCGTTAAGTCGACCGCCTGGGGAGTACGGCCGCAAGGTTA
AAACTCAAATGAATTGACGGGGGCCCGCACAAGCGGTGGAGCATGTGGTTTAATTCGAT
GCAACGCGAAGAACCTTACCTACTCTTGACATCCACAGAACTTAGCAGAGATGCTTCGG
TGCCTTCGGGAACTGTGAGACAGGTGCTGCATGGCTGTCGTCAGCTCGTGTTGTGAAAT
GTTGGGTTAAGTCCCGCAACGAGCGCAACCCTTATCCTTTGTTGCCAGCACGTAATGGT
GGGAACTCAAGGGAGACTGCCGGTGACAAACCGGAGGAAGGTGGGGATGACGTCAAG
TCATCATGGCCCTTACGAGTAGGGCTACACACGTGCTACAATGGCAGATACAAAGTGAA
GCGAACTCGCGAGAGCAAGCGGACCACATAAAGTCTGTCGTAGTCCGGATTGGAGTCT
GCAACTCGACTCCATGAAGTCGGAATCGCTAGTAATCGTAGATCAGAATGCTACGGTGA
ATACGTTCCCGGGCCTTGTACACACCGCCCGTCACACCATGGGAGTGGGTTGCAAAAG
AAGTAGGTAGCTTAACCTTCGGGAGGGCGCTTACCACTTTGTGATTCATGACTGGGG
pattern 265
CGCTGGCGGCAGGCCTAACACATGCAAGTCGAGCGGCAGCGGAAAGTAGTTTACTACT
TTGCCGGCGAGCGGCGGACGGGTGAGTAATGTCTGGGAAACTGCCTGATGGAGGGGGA
TAACTACTGGAAACGGTAGCTAATACCGCATGACCTCGCAAGAGCAAAGTGGGGGACC
TTCGGGCCTCACGCCATCGGATGTGCCCAGATGGGATTAGCTAGTAGGTGGGGTAATGG
CTCACCTAGGCGACGATCCCTAGCTGGTCTGAGAGGATGACCAGCCACACTGGAACTG
AGACACGGTCCAGACTCCTACGGGAGGCAGCAGTGGGGAATATTGCACAATGGGCGCA
AGCCTGATGCAGCCATGCCGCGTGTGTGAAGAAGGCCTTCGGGTTGTAAAGCACTTTCA
GCGAGGAGGAAGGCAGTCGTGTTAATAGCACGATTGATTGACGTTACTCGCAGAAGAA
GCACCGGCTAACTCCGTGCCAGCAGCCGCGGTAATACGGAGGGTGCAAGCGTTAATCG
GAATTACTGGGCGTAAAGCGCACGCAGGCGGTTTGTTAAGTCAGATGTGAAATCCCCGC
GCTTAACGTGGGAACTGCATTTGAAACTGGCAAGCTAGAGTCTTGTAGAGGGGGGTAG
AATTCCAGGTGTAGCGGTGAAATGCGTAGAGATCTGGAGGAATACCGGTGGCGAAGGC
GGCCCCCTGGACAAAGACTGACGCTCAGGTGCGAAAGCGTGGGGAGCAAACAGGATT
AGATACCCTGGTAGTCCACGCTGTAAACGATGTCGACTTGGAGGTTGTGCCCTTGAGGC
GTGGCTTCCGGAGCTAACGCGTTAAGTCGACCGCCTGGGGAGTACGGCCGCAAGGTTA
AAACTCAAATGAATTGACGGGGGCCCGCACAAGCGGTGGAGCATGTGGTTTAATTCGAT

GCAACGCGAAGAACCTTACCTACTCTTGACATCCACAGAACTTAGCAGAGATGCTTCGG
TGCCTTCGGGAACTGTGAGACAGGTGCTGCATGGCTGTCGTCAGCTCGTGTTGTGAAAT
GTTGGGTTAAGTCCCGCAACGAGCGCAACCCTTATCCTTTGTTGCCAGCACGTAATGGT
GGGAACTCAAGGGAGACTGCCGGTGACAAACCGGAGGAAGGTGGGGATGACGTCAAG
TCATCATGGCCCTTACGAGTAGGGCTACACACGTGCTACAATGGCAGATACAAAGTGAA
GCGAACTCGCGAGAGCAAGCGGACCACATAAAGTCTGTCGTAGTCCGGATTGGAGTCT
GCAACTCGACTCCATGAAGTCGGAATCGCTAGTAATCGTAGATCAGAATGCTACGGTGA
ATACGTTCCCGGGCCTTGTACACACCGCCCGTCACACCATGGGAGTGGGTTGCAAAAG
AAGTAGGTAGCTTAACCTTCGGGAGGGCGCTTACCACTTTGTGATTCATGACTGGGG
pattern 266
CGCTGGCGGCAGGCCTAACACATGCAAGTCGAGCGGCAGCGGGAAGTAGTTTACTACT
TCGCCGGCGAGCGGCGGACGGGTGAGTAATGTCTGGGAAACTGCCTGATGGAGGGGGA
TAACTACTGGAAACGGTAGCTAATACCGCATGACCTCGCAAGAGCAAAGTGGGGGACC
TTAGGGCCTCACGCCATCGGATGTGCCCAGATGGGATTAGCTAGTAGGTGGGGTAATGG
CTCACCTAGGCGACGATCCCTAGCTGGTCTGAGAGGATGACCAGCCACACTGGAACTG
AGACACGGTCCAGACTCCTACGGGAGGCAGCAGTGGGGAATATTGCACAATGGGCGCA
AGCCTGATGCAGCCATGCCGCGTGTGTGAAGAAGGCCTTCGGGTTGTAAAGCACTTTCA
GCGAGGAGGAAGGCAATCGTGTTAATAGCACGGTTGATTGACGTTACTCGCAGAAGAA
GCACCGGCTAACTCCGTGCCAGCAGCCGCGGTAATACGGAGGGTGCAAGCGTTAATCG
GAATTACTGGGCGTAAAGCGCACGCAGGCGGTTTGTTAAGTCAGATGTGAAATCCCCGC
GCTTAACGTGGGAACTGCATTTGAAACTGGCAAGCTAGAGTCTTGTAGAGGGGGGTAG
AATTCCAGGTGTAGCGGTGAAATGCGTAGAGATCTGGAGGAATACCGGTGGCGAAGGC
GGCCCCCTGGACAAAGACTGACGCTCAGGTGCGAAAGCGTGGGGAGCAAACAGGATT
AGATACCCTGGTAGTCCACGCTGTAAACGATGTCGACTTGGAGGTTGTGCCCTTGAGGC
GTGGCTTCCGGAGCTAACGCGTTAAGTCGACCGCCTGGGGAGTACGGCCGCAAGGTTA
AAACTCAAATGAATTGACGGGGGCCCGCACAAGCGGTGGAGCATGTGGTTTAATTCGAT
GCAACGCGAAGAACCTTACCTACTCTTGACATCCACAGAACTTAGCAGAGATGCTTCGG
TGCCTTCGGGAACTGTGAGACAGGTGCTGCATGGCTGTCGTCAGCTCGTGTTGTGAAAT
GTTGGGTTAAGTCCCGCAACGAGCGCAACCCTTATCCTTTGTTGCCAGCACGTAATGGT
GGGAACTCAAGGGAGACTGCCGGTGACAAACCGGAGGAAGGTGGGGATGACGTCAAG
TCATCATGGCCCTTACGAGTAGGGCTACACACGTGCTACAATGGCAGATACAAAGTGAA
GCGAACTCGCGAGAGCAAGCGGACCACATAAAGTCTGTCGTAGTCCGGATTGGAGTCT
GCAACTCGACTCCATGAAGTCGGAATCGCTAGTAATCGTAGATCAGAATGCTACGGTGA
ATACGTTCCCGGGCCTTGTACACACCGCCCGTCACACCATGGGAGTGGGTTGCAAAAG
AAGTAGGTAGCTTAACCTTAGGGAGGGCGCTTACCACTTTGTGATTCATGACTGGGG
pattern 267
CGCTGGCGGCAGGCCTAACACATGCAAGTCGAGCGGCAGCGGGAAGTAGTTTACTACT
TTGCCGGCGAGCGGCGGACGGGTGAGTAATGTCTGGGAAACTGCCTGATGGAGGGGGA
TAACTACTGGAAACGGTAGCTAATACCGCATGACCTCGCAAGAGCAAAGTGGGGGACC
TTCGGGCCTCACGCCATCGGATGTGCCCAGATGGGATTAGCTAGTAGGTGAGGTAATGG
CTCACCTAGGCGACGATCCCTAGCTGGTCTGAGAGGATGACCAGCCACACTGGAACTG
AGACACGGTCCAGACTCCTACGGGAGGCAGCAGTGGGGAATATTGCACAATGGGCGCA
AGCCTGATGCAGCCATGCCGCGTGTGTGAAGAAGGCCTTCGGGTTGTAAAGCACTTTCA
GCGAGGAGGAAGGCAGTCGTGTTAATAGCACGGTTGATTGACGTTACTCGCAGAAGAA

GCACCGGCTAACTCCGTGCCAGCAGCCGCGGTAATACGGAGGGTGCAAGCGTTAATCG
GAATTACTGGGCGTAAAGCGCACGCAGGCGGTTTGTTAAGTCAGATGTGAAATCCCCGC
GCTTAACGTGGGAACTGCATTTGAAACTGGCAAGCTAGAGTCTTGTAGAGGGGGGTAG
AATTCCAGGTGTAGCGGTGAAATGCGTAGAGATCTGGAGGAATACCGGTGGCGAAGGC
GGCCCCCTGGACAAAGACTGACGCTCAGGTGCGAAAGCGTGGGGAGCAAACAGGATT
AGATACCCTGGTAGTCCACGCTGTAAACGATGTCGACTTGGAGGTTGTGCCCTTGAGGC
GTGGCTTCCGGAGCTAACGCGTTAAGTCGACCGCCTGGGGAGTACGGCCGCAAGGTTA
AAACTCAAATGAATTGACGGGGGCCCGCACAAGCGGTGGAGCATGTGGTTTAATTCGAT
GCAACGCGAAGAACCTTACCTACTCTTGACATCCACAGAACTTAGCATAGATGCTTCGG
TGCCTTCGGGAACTGTGAGACAGGTGCTGCATGGCTGTCGTCAGCTCGTGTTGTGAAAT
GTTGGGTTAAGTCCCGCAACGAGCGCAACCCTTATCCTTTGTTGCCAGCACGTAATGGT
GGGAACTCAAGGGAGACTGCCGGTGACAAACCGGAGGAAGGTGGGGATGACGTCAAG
TCATCATGGCCCTTACGAGTAGGGCTACACACGTGCTACAATGGCAGATACAAAGTGAA
GCGAACTCGCGAGAGCAAGCGGACCACATAAAGTCTGTCGTAGTCCGGATTGGAGTCT
GCAACTCGACTCCATGAAGTCGGAATCGCTAGTAATCGTAGATCAGAATGCTACGGTGA
ATACGTTCCCGGGCCTTGTACACACCGCCCGTCACACCATGGGAGTGGGTTGCAAAAG
AAGTAGGTAGCTTAACCTTCGGGAGGGCGCTTACCACTTTGTGATTCATGACTGGGG
pattern 268
CGCTGGCGGCAGGCCTAACACATGCAAGTCGAGCGGCAGCGGGAAGTAGTTTACTACT
TTGCCGGCGAGCGGCGGACGGGTGAGTAATGTCTGGGAAACTGCCTGATGGAGGGGGA
TAACTACTGGAAACGGTAGCTAATACCGCATGACCTCGCAAGAGCAAAGTGGGGGACC
TTCGGGCCTCACGCCATCGGATGTGCCCAGATGGGATTAGCTAGTAGGTGGGGTAATGG
CTCACCTAGGCGACGATCCCTAGCTGGTCTGAGAGGATGACCAGCCACACTGGAACTG
AGACACGGTCCAGACTCCTACGGGAGGCAGCAGTGGGGAATATTGCACAATGGGCGCA
AGCCTGATGCAGCCATGCCGCGTGTGTGAAGAAGGCCTTCGGGTTGTAAAGCACTTTCA
GCGAGGAGGAAGGCAGTCGTGTTAATAGCACGATTGATTGACGTTACTCGCAGAAGAA
GCACCGGCTAACTCCGTGCCAGCAGCCGCGGTAATACGGAGGGTGCAAGCGTTAATCG
GAATTACTGGGCGTAAAGCGCACGCAGGCGGTTTGTTAAGTCAGATGTGAAATCCCCGC
GCTTAACGTGGGAACTGCATTTGAAACTGGCAAGCTAGAGTCTTGTAGAGGGGGGTAG
AATTCCAGGTGTAGCGGTGAAATGCGTAGAGATCTGGAGGAATACCGGTGGCGAAGGC
GGCCCCCTGGACAAAGACTGACGCTCAGGTGCGAAAGCGTGGGGAGCAAACAGGATT
AGATACCCTGGTAGTCCACGCTGTAAACGATGTCGACTTGGAGGTTGTGCCCTTGAGGC
GTGGCTTCCGGAGCTAACGCGTTAAGTCGACCGCCTGGGGAGTACGGCCGCAAGGTTA
AAACTCAAATGAATTGACGGGGGCCCGCACAAGCGGTGGAGCATGTGGTTTAATTCGAT
GCAACGCGAAGAACCTTACCTACTCTTGACATCCACAGAACTTAGCATAGATGCTTCGG
TGCCTTCGGGAACTGTGAGACAGGTGCTGCATGGCTGTCGTCAGCTCGTGTTGTGAAAT
GTTGGGTTAAGTCCCGCAACGAGCGCAACCCTTATCCTTTGTTGCCAGCACGTAATGGT
GGGAACTCAAGGGAGACTGCCGGTGACAAACCGGAGGAAGGTGGGGATGACGTCAAG
TCATCATGGCCCTTACGAGTAGGGCTACACACGTGCTACAATGGCAGATACAAAGTGAA
GCGAACTCGCGAGAGCAAGCGGACCACATAAAGTCTGTCGTAGTCCGGATTGGAGTCT
GCAACTCGACTCCATGAAGTCGGAATCGCTAGTAATCGTAGATCAGAATGCTACGGTGA
ATACGTTCCCGGGCCTTGTACACACCGCCCGTCACACCATGGGAGTGGGTTGCAAAAG
AAGTAGGTAGCTTAACCTTCGGGAGGGCGCTTACCACTTTGTGATTCATGACTGGGG
pattern 269

CGCTGGCGGCAGGCCTAACACATGCAAGTCGAGCGGCAGCGGAAAGTAGCTTGCTACT
TTGCCGGCGAGCGGCGGACGGGTGAGTAATGTCTGGGGATCTGCCTGATGGAGGGGGA
TAACTACTGGAAACGGTAGCTAATACCGCATGACCTCGAAAGAGCAAAGTGGGGGACC
TTCGGGCCTCACGCCATCGGATGAACCCAGATGGGATTAGCTAGTAGGTGGGGTAATGG
CTCACCTAGGCGACGATCCCTAGCTGGTCTGAGAGGATGACCAGCCACACTGGAACTG
AGACACGGTCCAGACTCCTACGGGAGGCAGCAGTGGGGAATATTGCACAATGGGCGCA
AGCCTGATGCAGCCATGCCGCGTGTGTGAAGAAGGCCTTCGGGTTGTAAAGCACTTTCA
GCGAGGAGGAAGGCATTGTGGTTAATAACCGCAGTGATTGACGTTACTCGCAGAAGAA
GCACCGGCTAACTCCGTGCCAGCAGCCGCGGTAATACGGAGGGTGCAAGCGTTAATCG
GAATTACTGGGCGTAAAGCGCACGCAGGCGGTTTGTTAAGTCAGATGTGAAATCCCCGC
GCTTAACGTGGGAACTGCATTTGAAACTGGCAAGCTAGAGTCTTGTAGAGGGGGGTAG
AATTCCAGGTGTAGCGGTGAAATGCGTAGAGATCTGGAGGAATACCGGTGGCGAAGGC
GGCCCCCTGGACAAAGACTGACGCTCAGGTGCGAAAGCGTGGGGAGCAAACAGGATT
AGATACCCTGGTAGTCCACGCTGTAAACGATGTCGACTTGGAGGTTGTGCCCTTGAGGC
GTGGCTTCCGGAGCTAACGCGTTAAGTCGACCGCCTGGGGAGTACGGCCGCAAGGTTA
AAACTCAAATGAATTGACGGGGGCCCGCACAAGCGGTGGAGCATGTGGTTTAATTCGAT
GCAACGCGAAGAACCTTACCTACTCTTGACATCCACAGAACTTAGCAGAGATGCTTCGG
TGCCTTCGGGAACTGTGAGACAGGTGCTGCATGGCTGTCGTCAGCTCGTGTTGTGAAAT
GTTGGGTTAAGTCCCGCAACGAGCGCAACCCTTATCCTTTGTTGCCAGCACGTAATGGT
GGGAACTCAAGGGAGACTGCCGGTGACAAACCGGAGGAAGGTGGGGATGACGTCAAG
TCATCATGGCCCTTACGAGTAGGGCTACACACGTGCTACAATGGCAGATACAAAGTGAA
GCGAACTCGCGAGAGCAAGCGAACCACATAAAGTCTGTCGTAGTCCGGATTGGAGTCT
GCAACTCGACTCCATGAAGTCGGAATCGCTAGTAATCGTAGATCAGAATGCTACGGTGA
ATACGTTCCCGGGCCTTGTACACACCGCCCGTCACACCATGGGAGTGGGTTGCAAAAG
AAGTAGGTAGCTTAACCTTCGGGAGGGCGCTTACCACTTTGTGATTCATGACTGGGG
pattern 270
CGCTGGCGGCAGGCCTAACACATGCAAGTCGAGCGGCAGCGGAAAGTAGCTTGCTACT
TTGCCGGCGAGCGGCGGACGGGTGAGTAATGTCTGGGGATCTGCCTGATGGAGGGGGA
TAACTACTGGAAACGGTAGCTAATACCGCATGACCTCGAAAGAGCAAAGTGGGGGACC
TTCGGGCCTCACGCCATCGGATGAACCCAGATGGGATTAGCTAGTAGGTGGGGTAATGG
CTCACCTAGGCGACGATCCCTAGCTGGTCTGAGAGGATGACCAGCCACACTGGAACTG
AGACACGGTCCAGACTCCTACGGGAGGCAGCAGTGGGGAATATTGCACAATGGGCGCA
AGCCTGATGCAGCCATGCCGCGTGTGTGAAGAAGGCCTTCGGGTTGTAAAGCACTTTCA
GCGAGGAGGAAGGCATTGTGGTTAATAACCGCAGTGATTGACGTTACTCGCAAAAGAA
GCACCGGCTAACTCCGTGCCAGCAGCCGCGGTAATACGGAGGGTGCAAGCGTTAATCG
GAATTACTGGGCGTAAAGCGCACGCAGGCGGTTTGTTAAGTCAGATGTGAAATCCCCGC
GCTTAACGTGGGAACTGCATTTGAAACTGGCAAGCTAGAGTCTTGTAGAGGGGGGTAG
AATTCCAGGTGTAGCGGTGAAATGCGTAGAGATCTGGAGGAATACCGGTGGCGAAGGC
GGCCCCCTGGACAAAGACTGACGCTCAGGTGCGAAAGCGTGGGGAGCAAACAGGATT
AGATACCCTGGTAGTCCACGCTGTAAACGATGTCGACTTGGAGGTTGTGCCCTTGAGGC
GTGGCTTCCGGAGCTAACGCGTTAAGTCGACCGCCTGGGGAGTACGGCCGCAAGGTTA
AAACTCAAATGAATTGACGGGGGCCCGCACAAGCGGTGGAGCATGTGGTTTAATTCGAT
GCAACGCGAAGAACCTTACCTACTCTTGACATCCACAGAACTTAGCAGAGATGCTTCGG
TGCCTTCGGGAACTGTGAGACAGGTGCTGCATGGCTGTCGTCAGCTCGTGTTGTGAAAT

GTTGGGTTAAGTCCCGCAACGAGCGCAACCCTTATCCTTTGTTGCCAGCACGTAATGGT
GGGAACTCAAGGGAGACTGCCGGTGACAAACCGGAGGAAGGTGGGGATGACGTCAAG
TCATCATGGCCCTTACGAGTAGGGCTACACACGTGCTACAATGGCAGATACAAAGTGAA
GCGAACTCGCGAGAGCAAGCGGACCACATAAAGTCTGTCGTAGTCCGGATTGGAGTCT
GCAACTCGACTCCATGAAGTCGGAATCGCTAGTAATCGTAGATCAGAATGCTACGGTGA
ATACGTTCCCGGGCCTTGTACACACCGCCCGTCACACCATGGGAGTGGGTTGCAAAAG
AAGTAGGTAGCTTAACCTTCGGGAGGGCTCTTACCACTTTGTGATTCATGACTGGGG
pattern 271
CGCTGGCGGCAGGCCTAACACATGCAAGTCGAGCGGCAGCGGAAAGTAGCTTGCTACT
TTGCCGGCGAGCGGCGGACGGGTGAGTAATGTCTGGGGATCTGCCTGATGGAGGGGGA
TAACTACTGGAAACGGTAGCTAATACCGCATGACCTCGAAAGAGCAAAGTGGGGGACC
TTCGGGCCTCACGCCATCGGATGAACCCAGATGGGATTAGCTAGTAGGTGGGGTAATGG
CTCACCTAGGCGACGATCCCTAGCTGGTCTGAGAGGATGACCAGCCACACTGGAACTG
AGACACGGTCCAGACTCCTACGGGAGGCAGCAGTGGGGAATATTGCACAATGGGTGCA
AGCCTGATGCAGCCATGCCGCGTGTGTGAAGAAGGCCTTCGGGTTGTAAAGCACTTTCA
GCGAGGAGGAAGGCATTGTGGTTAATAACCGCAGTGATTGACGTTACTCGCAGAAGAA
GCACCGGCTAACTCCGTGCCAGCAGCCGCGGTAATACGGAGGGTGCAAGCGTTAATCG
GAATTACTGGGCGTAAAGCGCACGCAGGCGGTTTGTTAAGTCAGATGTGAAATCCCCGC
GCTTAACGTGGGAACTGCATTTGAAACTGGCAAGCTAGAGTCTTGTAGAGGGGGGTAG
AATTCCAGGTGTAGCGGTGAAATGCGTAGAGATCTGGAGGAATACCGGTGGCGAAGGC
GGCCCCCTGGACAAAGACTGACGCTCAGGTGCGAAAGCGTGGGGAGCAAACAGGATT
AGATACCCTGGTAGTCCACGCTGTAAACGATGTCGACTTGGAGGTTGTGCCCTTGAGGC
GTGGCTTCCGGAGCTAACGCGTTAAGTCGACCGCCTGGGGAGTACGGCCGCAAGGTTA
AAACTCAAATGAATTGACGGGGGCCCGCACAAGCGGTGGAGCATGTGGTTTAATTCGAT
GCAACGCGAAGAACCTTACCTACTCTTGACATCCACAGAACTTAGCAGAGATGCTTCGG
TGCCTTCGGGAACTGTGAGACAGGTGCTGCATGGCTGTCGTCAGCTCGTGTTGTGAAAT
GTTGGGTTAAGTCCCGCAACGAGCGCAACCCTTATCCTTTGTTGCCAGCACGTAATGGT
GGGAACTCAAGGGAGACTGCCGGTGACAAACCGGAGGAAGGTGGGGATGACGTCAAG
TCATCATGGCCCTTACGAGTAGGGCTACACACGTGCTACAATGGCAGATACAAAGTGAA
GCGAACTCGCGAGAGCAAGCGGACCACATAAAGTCTGTCGTAGTCCGGATTGGAGTCT
GCAACTCGACTCCATGAAGTCGGAATCGCTAGTAATCGTAGATCAGAATGCTACGGTGA
ATACGTTCCCGGGCCTTGTACACACCGCCCGTCACACCATGGGAGTGGGTTGCAAAAG
AAGTAGGTAGCTTAACCTTCGGGAGGGCGCTTACCACTTTGTGATTCATGACTGGGG
pattern 272
CGCTGGCGGCAGGCCTAACACATGCAAGTCGAGCGGCAGCGGGAAGTAGTTTACTACT
TTGCCGGCGAGCGGCGGACGGGTGAGTAATGTCTGGGAAACTGCCTGATGGAGGGGGA
TAACTACTGGAAACGGTAGCTAATACCGCATGACCTCGCAAGAGCAAAGTGGGGGACC
TTAGGGCCTCACGCCATCGGATGTGCCCAGATGGGATTAGCTAGTAGGTGGGGTAATGG
CTCACCTAGGCGACGATCCCTAGCTGGTCTGAGAGGATGACCAGCCACACTGGAACTG
AGACACGGTCCAGACTCCTACGGGAGGCAGCAGTGGGGAATATTGCACAATGGGCGCA
AGCCTGATGCAGCCATGCCGCGTGTGTGAAGAAGGCCTTCGGGTTGTAAAGCACTTTCA
GCGAGGAGGAAGGCAGTCGTGTTAATAGCACGGTTGATTGACGTTACTCGCAGAAGAA
GCACCGGCTAACTCCGTGCCAGCAGCCGCGGTAATACGGAGGGTGCAAGCGTTAATCG
GAATTACTGGGCGTAAAGCGCACGCAGGCGGTTTGTTAAGTCAGATGTGAAATCCCCGC

GCTTAACGTGGGAACTGCATTTGAAACTGGCAAGCTAGAGTCTTGTAGAGGGGGGTAG
AATTCCAGGTGTAGCGGTGAAATGCGTAGAGATCTGGAGGAATACCGGTGGCGAAGGC
GGCCCCCTGGACAAAGACTGACGCTCAGGTGCGAAAGCGTGGGGAGCAAACAGGATT
AGATACCCTGGTAGTCCACGCTGTAAACGATGTCGACTTGGAGGTTGTGCCCTTGAGGC
GTGGCTTCCGGAGCTAACGCGTTAAGTCGACCGCCTGGGGAGTACGGCCGCAAGGTTA
AAACTCAAATGAATTGACGGGGGCCCGCACAAGCGGTGGAGCATGTGGTTTAATTCGAT
GCAACGCGAAGAACCTTACCTACTCTTGACATCCACAGAACTTAGCAGAGATGCTTCGG
TGCCTTCGGGAACTGTGAGACAGGTGCTGCATGGCTGTCGTCAGCTCGTGTTGTGAAAT
GTTGGGTTAAGTCCCGCAACGAGCGCAACCCTTATCCTTTGTTGCCAGCACGTAATGGT
GGGAACTCAAGGGAGACTGCCGGTGATAAACCGGAGGAAGGTGGGGATGACGTCAAG
TCATCATGGCCCTTACGAGTAGGGCTACACACGTGCTACAATGGCAGATACAAAGTGAA
GCGAACTCGCGAGAGCAAGCGGACCACATAAAGTCTGTCGTAGTCCGGATTGGAGTCT
GCAACTCGACTCCATGAAGTCGGAATCGCTAGTAATCGTAGATCAGAATGCTACGGTGA
ATACGTTCCCGGGCCTTGTACACACCGCCCGTCACACCATGGGAGTGGGTTGCAAAAG
AAGTAGGTAGCTTAACCTTCGGGAGGGCGCTTACCACTTTGTGATTCATGACTGGGG
pattern 273
CGCTGGCGGCAGGCCTAACACATGCAAGTCGAGCGGCAGCGGGAAGTAGTTTACTACT
TTGCCGGCGAGCGGCGGACGGGTGAGTAATGTCTGGGAAACTGCCTGATGGAGGGGGA
TAACTACTGGAAACGGTAGCTAATACCGCATGACCTCGCAAGAGCAAAGTGGGGGACC
TTAGGGCCTCACGCCATCGGATGTGCCCAGATGGGATTAGCTAGTAGGTGGGGTAATGG
CTCACCTAGGCGACGATCCCTAGCTGGTCTGAGAGGATGACCAGCCACACTGGAACTG
AGACACGGTCCAGACTCCTACGGGAGGCAGCAGTGGGGAATATTGCACAATGGGCGCA
AGCCTGATGCAGCCATGCCGCGTGTGTGAAGAAGGCCTTCGGGTTGTAAAGCACTTTCA
GCGAGGAGGAAGGCAATCGTGTTAATAGCACGGTTGATTGACGTTACTCGCAGAAGAA
GCACCGGCTAACTCCGTGCCAGCAGCCGCGGTAATACGGAGGGTGCAAGCGTTAATCG
GAATTACTGGGCGTAAAGCGCACGCAGGCGGTTTGTTAAGTCAGATGTGAAATCCCCGC
GCTTAACGTGGGAACTGCATTTGAAACTGGCAAGCTAGAGTCTTGTAGAGGGGGGTAG
AATTCCAGGTGTAGCGGTGAAATGCGTAGAGATCTGGAGGAATACCGGTGGCGAAGGC
GGCCCCCTGGACAAAGACTGACGCTCAGGTGCGAAAGCGTGGGGAGCAAACAGGATT
AGATACCCTGGTAGTCCACGCTGTAAACGATGTCGACTTGGAGGTTGTGCCCTTGAGGC
GTGGCTTCCGGAGCTAACGCGTTAAGTCGACCGCCTGGGGAGTACGGCCGCAAGGTTA
AAACTCAAATGAATTGACGGGGGCCCGCACAAGCGGTGGAGCATGTGGTTTAATTCGAT
GCAACGCGAAGAACCTTACCTACTCTTGACATCCACAGAACTTAGCAGAGATGCTTCGG
TGCCTTCGGGAACTGTGAGACAGGTGCTGCATGGCTGTCGTCAGCTCGTGTTGTGAAAT
GTTGGGTTAAGTCCCGCAACGAGCGCAACCCTTATCCTTTGTTGCCAGCACGTAATGGT
GGGAACTCAAGGGAGACTGCCGGTGATAAACCGGAGGAAGGTGGGGATGACGTCAAG
TCATCATGGCCCTTACGAGTAGGGCTACACACGTGCTACAATGGCAGATACAAAGTGAA
GCGAACTCGCGAGAGCAAGCGGACCACATAAAGTCTGTCGTAGTCCGGATTGGAGTCT
GCAACTCGACTCCATGAAGTCGGAATCGCTAGTAATCGTAGATCAGAATGCTACGGTGA
ATACGTTCCCGGGCCTTGTACACACCGCCCGTCACACCATGGGAGTGGGTTGCAAAAG
AAGTAGGTAGCTTAACCTTCGGGAGGGCGCTTACCACTTTGTGATTCATGACTGGGG
pattern 274
CGCTGGCGGCAGGCCTAACACATGCAAGTCGAGCGGCAGCGGGAAGTAGTTTACTACT
TTGCCGGCGAGCGGCGGACGGGTGAGTAATGTCTGGGAAACTGCCTGATGGAGGGGGA

TAACTACTGGAAACGGTAGCTAATACCGCATGACCTCGCAAGAGCAAAGTGGGGGACC
TTCGGGCCTCACGCCATCGGATGTGCCCAGATGGGATTAGCTAGTAGGTGGGGTAATGG
CTCACCTAGGCGACGATCCCTAGCTGGTCTGAGAGGATGACCAGCCACACTGGAACTG
AGACACGGTCCAGACTCCTACGGGAGGCAGCAGTGGGGAATATTGCACAATGGGCGCA
AGCCTGATGCAGCCATGCCGCGTGTGTGAAGAAGGCCTTCGGGTTGTAAAGCACTTTCA
GCGAGGAGGAAGGCAATCGTGTTAATAGCACGGTTGATTGACGTTACTCGCAGAAGAA
GCACCGGCTAACTCCGTGCCAGCAGCCGCGGTAATACGGAGGGTGCAAGCGTTAATCG
GAATTACTGGGCGTAAAGCGCACGCAGGCGGTTTGTTAAGTCAGATGTGAAATCCCCGC
GCTTAACGTGGGAACTGCATTTGAAACTGGCAAGCTAGAGTCTTGTAGAGGGGGGTAG
AATTCCAGGTGTAGCGGTGAAATGCGTAGAGATCTGGAGGAATACCGGTGGCGAAGGC
GGCCCCCTGGACAAAGACTGACGCTCAGGTGCGAAAGCGTGGGGAGCAAACAGGATT
AGATACCCTGGTAGTCCACGCTGTAAACGATGTCGACTTGGAGGTTGTGCCCTTGAGGC
GTGGCTTCCGGAGCTAACGCGTTAAGTCGACCGCCTGGGGAGTACGGCCGCAAGGTTA
AAACTCAAATGAATTGACGGGGGCCCGCACAAGCGGTGGAGCATGTGGTTTAATTCGAT
GCAACGCGAAGAACCTTACCTACTCTTGACATCCACAGAACTTAGCAGAGATGCTTCGG
TGCCTTCGGGAACTGTGAGACAGGTGCTGCATGGCTGTCGTCAGCTCGTGTTGTGAAAT
GTTGGGTTAAGTCCCGCAACGAGCGCAACCCTTATCCTTTGTTGCCAGCACGTAATGGT
GGGAACTCAAGGGAGACTGCCGGTGATAAACCGGAGGAAGGTGGGGATGACGTCAAG
TCATCATGGCCCTTACGAGTAGGGCTACACACGTGCTACAATGGCAGATACAAAGTGAA
GCGAACTCGCGAGAGCAAGCGGACCACATAAAGTCTGTCGTAGTCCGGATTGGAGTCT
GCAACTCGACTCCATGAAGTCGGAATCGCTAGTAATCGTAGATCAGAATGCTACGGTGA
ATACGTTCCCGGGCCTTGTACACACCGCCCGTCACACCATGGGAGTGGGTTGCAAAAG
AAGTAGGTAGCTTAACCTTCGGGAGGGCGCTTACCACTTTGTGATTCATGACTGGGG
pattern 275
CGCTGGCGGCAGGCCTAACACATGCAAGTCGAGCGGCAGCGGGAAGTAGTTTACTACT
TTGCCGGCGAGCGGCGGACGGGTGAGTAATGTCTGGGAAACTGCCTGATGGAGGGGGA
TAACTACTGGAAACGGTAGCTAATACCGCATGACCTCGCAAGAGCAAAGTGGGGGACC
TTCGGGCCTCACGCCATCGGATGTGCCCAGATGGGATTAGCTAGTAGGTGGGGTAATGG
CTCACCTAGGCGACGATCCCTAGCTGGTCTGAGAGGATGACCAGCCACACTGGAACTG
AGACACGGTCCAGACTCCTACGGGAGGCAGCAGTGGGGAATATTGCACAATGGGCGCA
AGCCTGATGCAGCCATGCCGCGTGTGTGAAGAAGGCCTTCGGGTTGTAAAGCACTTTCA
GCGAGGAGGAAGGCAGTCGTGTTAATAGCACGGTTGATTGACGTTACTCGCAGAAGAA
GCACCGGCTAACTCCGTGCCAGCAGCCGCGGTAATACGGAGGGTGCAAGCGTTAATCG
GAATTACTGGGCGTAAAGCGCACGCAGGCGGTTTGTTAAGTCAGATGTGAAATCCCCGC
GCTTAACGTGGGAACTGCATTTGAAACTGGCAAGCTAGAGTCTTGTAGAGGGGGGTAG
AATTCCAGGTGTAGCGGTGAAATGCGTAGAGATCTGGAGGAATACCGGTGGCGAAGGC
GGCCCCCTGGACAAAGACTGACGCTCAGGTGCGAAAGCGTGGGGAGCAAACAGGATT
AGATACCCTGGTAGTCCACGCTGTAAACGATGTCGACTTGGAGGTTGTGCCCTTGAGGC
GTGGCTTCCGGAGCTAACGCGTTAAGTCGACCGCCTGGGGAGTACGGCCGCAAGGTTA
AAACTCAAATGAATTGACGGGGGCCCGCACAAGCGGTGGAGCATGTGGTTTAATTCGAT
GCAACGCGAAGAACCTTACCTACTCTTGACATCCACAGAACTTAGCAGAGATGCTTCGG
TGCCTTCGGGAACTGTGAGACAGGTGCTGCATGGCTGTCGTCAGCTCGTGTTGTGAAAT
GTTGGGTTAAGTCCCGCAACGAGCGCAACCCTTATCCTTTGTTGCCAGCACGTAATGGT
GGGAACTCAAGGGAGACTGCCGGTGATAAACCGGAGGAAGGTGGGGATGACGTCAAG

TCATCATGGCCCTTACGAGTAGGGCTACACACGTGCTACAATGGCAGATACAAAGTGAA
GCGAACTCGCGAGAGCAAGCGGACCACATAAAGTCTGTCGTAGTCCGGATTGGAGTCT
GCAACTCGACTCCATGAAGTCGGAATCGCTAGTAATCGTAGATCAGAATGCTACGGTGA
ATACGTTCCCGGGCCTTGTACACACCGCCCGTCACACCATGGGAGTGGGTTGCAAAAG
AAGTAGGTAGCTTAACCTTCGGGAGGGCGCTTACCACTTTGTGATTCATGACTGGGG

pattern 276

CGCTGGCGGCAGGCCTAACACATGCAAGTCGAGCGGCAGCGGGAAGTAGTTTACTACT
TTGCCGGCGAGCGGCGGACGGGTGAGTAATGTCTGGGAAACTGCCTGATGGAGGGGGA
TAACTACTGGAAACGGTAGCTAATACCGCATAACGTCTTCGGACCAAAGTGGGGGACCT
TCGGGCCTCACGCCATCGGATGTGCCCAGATGGGATTAGCTAGTAGGTGGGGTAATGGC
TCACCTAGGCGACGATCCCTAGCTGGTCTGAGAGGATGACCAGCCACACTGGAACTGA
GACACGGTCCAGACTCCTACGGGAGGCAGCAGTGGGGAATATTGCACAATGGGCGCAA
GCCTGATGCAGCCATGCCGCGTGTGTGAAGAAGGCCTTCGGGTTGTAAAGCACTTTCAG
CGAGGAGGAAGGCATAAAGGTTAATAACCTTTGTGATTGACGTTACTCGCAGAAGAAG
CACCGGCTAACTCCGTGCCAGCAGCCGCGGTAATACGGAGGGTGCAAGCGTTAATCGG
AATTACTGGGCGTAAAGCGCACGCAGGCGGTTTGTTAAGTCAGATGTGAAATCCCCGCG
CTTAACGTGGGAACTGCATTTGAAACTGGCAAGCTAGAGTCTTGTAGAGGGGGGTAGA
ATTCCAGGTGTAGCGGTGAAATGCGTAGAGATCTGGAGGAATACCGGTGGCGAAGGCG
GCCCCCTGGACAAAGACTGACGCTCAGGTGCGAAAGCGTGGGGAGCAAACAGGATTA
GATACCCTGGTAGTCCACGCTGTAAACGATGTCGACTTGGAGGTTGTGCCCTTGAGGCG
TGGCTTCCGGAGCTAACGCGTTAAGTCGACCGCCTGGGGAGTACGGCCGCAAGGTTAA
AACTCAAATGAATTGACGGGGGCCCGCACAAGCGGTGGAGCATGTGGTTTAATTCGATG
CAACGCGAAGAACCTTACCTACTCTTGACATCCACGGAATTTAGCAGAGATGCTTTAGT
GCCTTCGGGAACCGTGAGACAGGTGCTGCATGGCTGTCGTCAGCTCGTGTTGTGAAATG
TTGGGTTAAGTCCCGCAACGAGCGCAACCCTTATCCTTTGTTGCCAGCACGTGATGGTG
GGAACTCAAAGGAGACTGCCGGTGATAAACCGGAGGAAGGTGGGGATGACGTCAAGT
CATCATGGCCCTTACGAGTAGGGCTACACACGTGCTACAATGGCAGATACAAAGTGAAG
CGAACTCGCGAGAGCAAGCGGACCACATAAAGTCTGTCGTAGTCCGGATTGGAGTCTG
CAACTCGACTCCATGAAGTCGGAATCGCTAGTAATCGTAGATCAGAATGCTACGGTGAAT
ACGTTCCCGGGCCTTGTACACACCGCCCGTCACACCATGGGAGTGGGTTGCAAAAGAA
GTAGGTAGCTTAACCTTCGGGAGGGCGCTTACCACTTTGTGATTCATGACTAGGG

pattern 277

CGCTGGCGGCAGGCCTAACACATGCAAGTCGAGCGGCAGCGGAAAGTAGCTTGCTACT
TTGCCGGCGAGCGGCGGACGGGTGAGTAATGTCTGGGAAACTGCCTGATGGAGGGGGA
TAACTACTGGAAACGGTAGCTAATACCGCATGACCTCGAAAGAGCAAAGTGGGGGACC
TTCGGGCCTCACGCCATCGGATGTGCCCAGATGGGATTAGCTAGTAGGTGGGGTAATGG
CTCACCTAGGCGACGATCCCTAGCTGGTCTGAGAGGATGACCAGCCACACTGGAACTG
AGACACGGTCCAGACTCCTACGGGAGGCAGCAGTGGGGAATATTGCACAATGGGCGCA
AGCCTGATGCAGCCATGCCGCGTGTGTGAAGAAGGCCTTCGGGTTGTAAAGCACTTTCA
GCGAGGAGGAAGGCATTTCACTTAATACGTGAAGTGATTGACGTTACTCGCAGAAGAA
GCACCGGCTAACTCCGTGCCAGCAGCCGCGGTAATACGGAGGGTGCAAGCGTTAATCG
GAATTACTGGGCGTAAAGCGCACGCAGGCGGTTTGTTAAGTCAGATGTGAAATCCCCGA
GCTTAACTTGGGAACTGTATTTGAAACTGGCAAGCTAGAGTCTTGTAGAGGGGGGTAGA
ATTCCAGGTGTAGCGGTGAAATGCGTAGAGATCTGGAGGAATACCGGTGGCGAAGGCG

GCCCCCTGGACAAAGACTGACGCTCAGGTGCGAAAGCGTGGGGAGCAAACAGGATTA
GATACCCTGGTAGTCCACGCTGTAAACGATGTCGACTTGGAGGTTGTGCCCTTGAGGCG
TGGCTTCCGGAGCTAACGCGTTAAGTCGACCGCCTGGGGAGTACGGCCGCAAGGTTAA
AACTCAAATGAATTGACGGGGGCCCGCACAAGCGGTGGAGCATGTGGTTTAATTCGATG
CAACGCGAAGAACCTTACCTACTCTTGACATCCACAGAACTTAGCAGAGATGCTTAGGT
GCCTTCGGGAACTGTGAGACAGGTGCTGCATGGCTGTCGTCAGCTCGTGTTGTGAAATG
TTGGGTTAAGTCCCGCAACGAGCGCAACCCTTATCCTTTGTTGCCAGCACGTAATGGTG
GGAACTCAAAGGAGACTGCCGGTGATAAACCGGAGGAAGGTGGGGATGACGTCAAGT
CATCATGGCCCTTACGAGTAGGGCTACACACGTGCTACAATGGCAGATACAAAGTGAAG
CGAACTCGCGAGAGCAAGCGGACCACATAAAGTCTGTCGTAGTCCGGATTGGAGTCTG
CAACTCGACTCCATGAAGTCGGAATCGCTAGTAATCGTAGATCAGAATGCTACGGTGAAT
ACGTTCCCGGGCCTTGTACACACCGCCCGTCACACCATGGGAGTGGGTTGCAAAAGAA
GTAGGTAGCTTAACCTTCGGGAGGGCGCTTACCACTTTGTGATTCATGACTGGGG
pattern 278
CGCTGGCGGCAGGCCTAACACATGCAAGTCGAGCGGCAGCGGAAAGTAGCTTGCTACT
TTGCCGGCGAGCGGCGGACGGGTGAGTAATGTCTGGGAAACTGCCTGATGGAGGGGGA
TAACTACTGGAAACGGTAGCTAATACCGCATGACCTCGAAAGAGCAAAGTGGGGGACC
TTCGGGCCTCACGCCATCGGATGTGCCCAGATGGGATTAGCTAGTAGGTGGGGTAATGG
CTCACCTAGGCGACGATCCCTAGCTGGTCTGAGAGGATGACCAGCCACACTGGAACTG
AGACACGGTCCAGACTCCTACGGGAGGCAGCAGTGGGGAATATTGCACAATGGGCGCA
AGCCTGATGCAGCCATGCCGCGTGTGTGAAGAAGGCCTTCGGGTTGTAAAGCACTTTCA
GCGAGGAGGAAGGCATTTCACTTAATACGTGAAGTGATTGACGTTACTCGCAGAAGAA
GCACCGGCTAACTCCGTGCCAGCAGCCGCGGTAATACGGAGGGTGCAAGCGTTAATCG
GAATTACTGGGCGTAAAGCGCACGCAGGCGGTTTGTTAAGTCAGATGTGAAATCCCCGA
GCTTAACTTGGGAACTGTATTTGAAACTGGCAAGCTAGAGTCTTGTAGAGGGGGGTAGA
ATTCCAGGTGTAGCGGTGAAATGCGTAGAGATCTGGAGGAATACCGGTGGCGAAGGCG
GCCCCCTGGACAAAGACTGACGCTCAGGTGCGAAAGCGTGGGGAGCAAACAGGATTA
GATACCCTGGTAGTCCACGCTGTAAACGATGTCGACTTGGAGGTTGTGCCCTTGAGGCG
TGGCTTCCGGAGCTAACGCGTTAAGTCGACCGCCTGGGGAGTACGGCCGCAAGGTTAA
AACTCAAATGAATTGACGGGGGCCCGCACAAGCGGTGGAGCATGTGGTTTAATTCGATG
CAACGCGAAGAACCTTACCTACTCTTGACATCCACAGAACTGAGCAGAGATGCTTAGGT
GCCTTCGGGAACTGTGAGACAGGTGCTGCATGGCTGTCGTCAGCTCGTGTTGTGAAATG
TTGGGTTAAGTCCCGCAACGAGCGCAACCCTTATCCTTTGTTGCCAGCACGTAATGGTG
GGAACTCAAAGGAGACTGCCGGTGATAAACCGGAGGAAGGTGGGGATGACGTCAAGT
CATCATGGCCCTTACGAGTAGGGCTACACACGTGCTACAATGGCAGATACAAAGTGAAG
CGAACTCGCGAGAGCAAGCGGACCACATAAAGTCTGTCGTAGTCCGGATTGGAGTCTG
CAACTCGACTCCATGAAGTCGGAATCGCTAGTAATCGTAGATCAGAATGCTACGGTGAAT
ACGTTCCCGGGCCTTGTACACACCGCCCGTCACACCATGGGAGTGGGTTGCAAAAGAA
GTAGGTAGCTTAACCTTCGGGAGGGCGCTTACCACTTTGTGATTCATGACTGGGG
pattern 279
CGCTGGCGGCAGGCCTAACACATGCAAGTCGAGCGGCAGCGGGAAGTAGTTTACTACT
TTGCCGGCGAGCGGCGGACGGGTGAGTAATGTCTGGGAAACTGCCTGATGGAGGGGGA
TAACTACTGGAAACGGTAGCTAATACCGCATAACGTCTTCGGACCAAAGTGGGGGACCT
TCGGGCCTCACGCCATCGGATGTGCCCAGATGGGATTAGCTAGTAGGTGGGGTAATGGC

TCACCTAGGCGACGATCCCTAGCTGGTCTGAGAGGATGACCAGCCACACTGGAACTGA
GACACGGTCCAGACTCCTACGGGAGGCAGCAGTGGGGAATATTGCACAATGGGCGCAA
GCCTGATGCAGCCATGCCGCGTGTGTGAAGAAGGCCTTCGGGTTGTAAAGCACTTTCAG
CGAGGAGGAAGGCATAAAGGTTAATAACCTTTGTGATTGACGTTACTCGCAGAAGAAG
CACCGGCTAACTCCGTGCCAGCAGCCGCGGTAATACGGAGGGTGCAAGCGTTAATCGG
AATTACTGGGCGTAAAGCGCACGCAGGCGGTTTGTTAAGTCAGATGTGAAATCCCCGCG
CTTAACGTGGGAACTGCATTTGAAACTGGCAAGCTAGAGTCTTGTAGAGGGGGGTAGA
ATTCCAGGTGTAGCGGTGAAATGCGTAGAGATCTGGAGGAATACCGGTGGCGAAGGCG
GCCCCCTGGACAAAGACTGACGCTCAGGTGCGAAAGCGTGGGGAGCAAACAGGATTA
GATACCCTGGTAGTCCACGCTGTAAACGATGTCGACTTGGAGGTTGTGCCCTTGAGGCG
TGGCTTCCGGAGCTAACGCGTTAAGTCGACCGCCTGGGGAGTACGGCCGCAAGGTTAA
AACTCAAATGAATTGACGGGGGCCCGCACAAGCGGTGGAGCATGTGGTTTAATTCGATG
CAACGCGAAGAACCTTACCTACTCTTGACATCCACAGAACTTAGCAGAGATGCTTCGGT
GCCTTCGGGAACTGTGAGACAGGTGCTGCATGGCTGTCGTCAGCTCGTGTTGTGAAATG
TTGGGTTAAGTCCCGCAACGAGCGCAACCCTTATCCTTTGTTGCCAGCACGTAATGGTG
GGAACTCAAAGGAGACTGCCGGTGATAAACCGGAGGAAGGTGGGGATGACGTCAAGT
CATCATGGCCCTTACGAGTAGGGCTACACACGTGCTACAATGGCAGATACAAAGTGAAG
CGAACTCGCGAGAGCAAGCGGACCACATAAAGTCTGTCGTAGTCCGGATTGGAGTCTG
CAACTCGACTCCATGAAGTCGGAATCGCTAGTAATCGTAGATCAGAATGCTACGGTGAAT
ACGTTCCCGGGCCTTGTACACACCGCCCGTCACACCATGGGAGTGGGTTGCAAAAGAA
GTAGGTAGCTTAACCTTCGGGAGGGCGCTTACCACTTTGTGATTCATGACTGGGG
pattern 280
CGCTGGCGGCAGGCCTAACACATGCAAGTCGAGCGGCAGCGGGAAGTAGTTTACTACT
TTGCCGGCGAGCGGCGGACGGGTGAGTAATGTCTGGGAAACTGCCTGATGGAGGGGGA
TAACTACTGGAAACGGTAGCTAATACCGCATAACGTCTACGGACCAAAGTGGGGGACCT
TCGGGCCTCACGCCATCGGATGTGCCCAGATGGGATTAGCTAGTAGGTGGGGTAATGGC
TCACCTAGGCGACGATCCCTAGCTGGTCTGAGAGGATGACCAGCCACACTGGAACTGA
GACACGGTCCAGACTCCTACGGGAGGCAGCAGTGGGGAATATTGCACAATGGGCGCAA
GCCTGATGCAGCCATGCCGCGTGTGTGAAGAAGGCCTTCGGGTTGTAAAGCACTTTCAG
CGAGGAGGAAGGCATAAAGGTTAATAACCTTTGTGATTGACGTTACTCGCAGAAGAAG
CACCGGCTAACTCCGTGCCAGCAGCCGCGGTAATACGGAGGGTGCAAGCGTTAATCGG
AATTACTGGGCGTAAAGCGCACGCAGGCGGTTTGTTAAGTCAGATGTGAAATCCCCGCG
CTTAACGTGGGAACTGCATTTGAAACTGGCAAGCTAGAGTCTTGTAGAGGGGGGTAGA
ATTCCAGGTGTAGCGGTGAAATGCGTAGAGATCTGGAGGAATACCGGTGGCGAAGGCG
GCCCCCTGGACAAAGACTGACGCTCAGGTGCGAAAGCGTGGGGAGCAAACAGGATTA
GATACCCTGGTAGTCCACGCTGTAAACGATGTCGACTTGGAGGTTGTGCCCTTGAGGCG
TGGCTTCCGGAGCTAACGCGTTAAGTCGACCGCCTGGGGAGTACGGCCGCAAGGTTAA
AACTCAAATGAATTGACGGGGGCCCGCACAAGCGGTGGAGCATGTGGTTTAATTCGATG
CAACGCGAAGAACCTTACCTACTCTTGACATCCACGGAATTTAGCAGAGATGCTTTAGT
GCCTTCGGGAACCGTGAGACAGGTGCTGCATGGCTGTCGTCAGCTCGTGTTGTGAAATG
TTGGGTTAAGTCCCGCAACGAGCGCAACCCTTATCCTTTGTTGCCAGCACGTAATGGTG
GGAACTCAAAGGAGACTGCCGGTGATAAACCGGAGGAAGGTGGGGATGACGTCAAGT
CATCATGGCCCTTACGAGTAGGGCTACACACGTGCTACAATGGCAGATACAAAGTGAAG
CGAACTCGCGAGAGCAAGCGGACCACATAAAGTCTGTCGTAGTCCGGATTGGAGTCTG

CAACTCGACTCCATGAAGTCGGAATCGCTAGTAATCGTAGATCAGAATGCTACGGTGAAT
ACGTTCCCGGGCCTTGTACACACCGCCCGTCACACCATGGGAGTGGGTTGCAAAAGAA
GTAGGTAGCTTAACCTTCGGGAGGGCGCTTACCACTTTGTGATTCATGACTGGGG
pattern 281
CGCTGGCGGCAGGCCTAACACATGCAAGTCGAGCGGCAGCGGGAAGTAGTTTACTACT
TTGCCGGCGAGCGGCGGACGGGTGAGTAATGTCTGGGAAACTGCCTGATGGAGGGGGA
TAACTACTGGAAACGGTAGCTAATACCGCATAACGTCTACGGACCAAAGTGGGGGACCT
TCGGGCCTCACGCCATCGGATGTGCCCAGATGGGATTAGCTAGTAGGTGGGGTAATGGC
TCACCTAGGCGACGATCCCTAGCTGGTCTGAGAGGATGACCAGCCACACTGGAACTGA
GACACGGTCCAGACTCCTACGGGAGGCAGCAGTGGGGAATATTGCACAATGGGCGCAA
GCCTGATGCAGCCATGCCGCGTGTGTGAAGAAGGCCTTCGGGTTGTAAAGCACTTTCAG
CGAGGAGGAAGGCATAAAGGTTAATAACCTTTGTGATTGACGTTACTCGCAGAAGAAG
CACCGGCTAACTCCGTGCCAGCAGCCGCGGTAATACGGAGGGTGCAAGCGTTAATCGG
AATTACTGGGCGTAAAGCGCACGCAGGCGGTTTGTTAAGTCAGATGTGAAATCCCCGCG
CTTAACGTGGGAACTGCATTTGAAACTGGCAAGCTAGAGTCTTGTAGAGGGGGGTAGA
ATTCCAGGTGTAGCGGTGAAATGCGTAGAGATCTGGAGGAATACCGGTGGCGAAGGCG
GCCCCCTGGACAAAGACTGACGCTCAGGTGCGAAAGCGTGGGGAGCAAACAGGATTA
GATACCCTGGTAGTCCACGCTGTAAACGATGTCGACTTGGAGGTTGTGCCCTTGAGGCG
TGGCTTCCGGAGCTAACGCGTTAAGTCGACCGCCTGGGGAGTACGGCCGCAAGGTTAA
AACTCAAATGAATTGACGGGGGCCCGCACAAGCGGTGGAGCATGTGGTTTAATTCGATG
CAACGCGAAGAACCTTACCTACTCTTGACATCCACAGAACTTAGCAGAGATGCTTCGGT
GCCTTCGGGAACTGTGAGACAGGTGCTGCATGGCTGTCGTCAGCTCGTGTTGTGAAATG
TTGGGTTAAGTCCCGCAACGAGCGCAACCCTTATCCTTTGTTGCCAGCACGTAATGGTG
GGAACTCAAAGGAGACTGCCGGTGATAAACCGGAGGAAGGTGGGGATGACGTCAAGT
CATCATGGCCCTTACGAGTAGGGCTACACACGTGCTACAATGGCAGATACAAAGTGAAG
CGAACTCGCGAGAGCAAGCGGACCACATAAAGTCTGTCGTAGTCCGGATTGGAGTCTG
CAACTCGACTCCATGAAGTCGGAATCGCTAGTAATCGTAGATCAGAATGCTACGGTGAAT
ACGTTCCCGGGCCTTGTACACACCGCCCGTCACACCATGGGAGTGGGTTGCAAAAGAA
GTAGGTAGCTTAACCTTCGGGAGGGCGCTTACCACTTTGTGATTCATGACTGGGG
pattern 282
CGCTGGCGGCAGGCCTAACACATGCAAGTCGAGCGGCAGCGGGAAGTAGTTTACTACT
TTGCCGGCGAGCGGCGGACGGGTGAGTAATGTCTGGGAAACTGCCTGATGGAGGGGGA
TAACTACTGGAAACGGTAGCTAATACCGCATAATGTCTACGGACCAAAGTGGGGGACCT
TCGGGCCTCACGCCATCGGATGTGCCCAGATGGGATTAGCTAGTAGGTGGGGTAATGGC
TCACCTAGGCGACGATCCCTAGCTGGTCTGAGAGGATGACCAGCCACACTGGAACTGA
GACACGGTCCAGACTCCTACGGGAGGCAGCAGTGGGGAATATTGCACAATGGGCGCAA
GCCTGATGCAGCCATGCCGCGTGTGTGAAGAAGGCCTTCGGGTTGTAAAGCACTTTCAG
CGAGGAGGAAGGCATAAAGGTTAATAACCTTTGTGATTGACGTTACTCGCAGAAGAAG
CACCGGCTAACTCCGTGCCAGCAGCCGCGGTAATACGGAGGGTGCAAGCGTTAATCGG
AATTACTGGGCGTAAAGCGCACGCAGGCGGTTTGTTAAGTCAGATGTGAAATCCCCGCG
CTTAACGTGGGAACTGCATTTGAAACTGGCAAGCTAGAGTCTTGTAGAGGGGGGTAGA
ATTCCAGGTGTAGCGGTGAAATGCGTAGAGATCTGGAGGAATACCGGTGGCGAAGGCG
GCCCCCTGGACAAAGACTGACGCTCAGGTGCGAAAGCGTGGGGAGCAAACAGGATTA
GATACCCTGGTAGTCCACGCTGTAAACGATGTCGACTTGGAGGTTGTGCCCTTGAGGCG

TGGCTTCCGGAGCTAACGCGTTAAGTCGACCGCCTGGGGAGTACGGCCGCAAGGTTAA
AACTCAAATGAATTGACGGGGGCCCGCACAAGCGGTGGAGCATGTGGTTTAATTCGATG
CAACGCGAAGAACCTTACCTACTCTTGACATCCACAGAACTTAGCAGAGATGCTTCGGT
GCCTTCGGGAACTGTGAGACAGGTGCTGCATGGCTGTCGTCAGCTCGTGTTGTGAAATG
TTGGGTTAAGTCCCGCAACGAGCGCAACCCTTATCCTTTGTTGCCAGCACGTAATGGTG
GGAACTCAAAGGAGACTGCCGGTGATAAACCGGAGGAAGGTGGGGATGACGTCAAGT
CATCATGGCCCTTACGAGTAGGGCTACACACGTGCTACAATGGCAGATACAAAGTGAAG
CGAACTCGCGAGAGCAAGCGGACCACATAAAGTCTGTCGTAGTCCGGATTGGAGTCTG
CAACTCGACTCCATGAAGTCGGAATCGCTAGTAATCGTAGATCAGAATGCTACGGTGAAT
ACGTTCCCGGGCCTTGTACACACCGCCCGTCACACCATGGGAGTGGGTTGCAAAAGAA
GTAGGTAGCTTAACCTTCGGGAGGGCGCTTACCACTTTGTGATTCATGACTGGGG
pattern 283
CGCTGGCGGCAGGCCTAACACATGCAAGTCGAGCGGCAGCGGGAAGTAGTTTACTACT
TTGCCGGCGAGCGGCGGACGGGTGAGTAATGTCTGGGAAACTGCCTGATGGAGGGGGA
TAACTACTGGAAACGGTAGCTAATACCGCATGACCTCGCAAGAGCAAAGTGGGGGACC
TTCGGGCCTCACGCCATCGGATGTGCCCAGATGGGATTAGCTAGTAGGTGGGGTAATGG
CTCACCTAGGCGACGATCCCTAGCTGGTCTGAGAGGATGACCAGCCACACTGGAACTG
AGACACGGTCCAGACTCCTACGGGAGGCAGCAGTGGGGAATATTGCACAATGGGCGCA
AGCCTGATGCAGCCATGCCGCGTGTGTGAAGAAGGCCTTCGGGTTGTAAAGCACTTTCA
GCGAGGAGGAAGGCAGTCGTGTTAATAGCACGATTGATTGACGTTACTCGCAGAAGAA
GCACCGGCTAACTCCGTGCCAGCAGCCGCGGTAATACGGAGGGTGCAAGCGTTAATCG
GAATTACTGGGCGTAAAGCGCACGCAGGCGGTTTGTTAAGTCAGATGTGAAATCCCCGC
GCTTAACGTGGGAACTGCATTTGAAACTGGCAAGCTAGAGTCTTGTAGAGGGGGGTAG
AATTCCAGGTGTAGCGGTGAAATGCGTAGAGATCTGGAGGAATACCGGTGGCGAAGGC
GGCCCCCTGGACAAAGACTGACGCTCAGGTGCGAAAGCGTGGGGAGCAAACAGGATT
AGATACCCTGGTAGTCCACGCTGTAAACGATGTCGACTTGGAGGTTGTGCCCTTGAGGC
GTGGCTTCCGGAGCTAACGCGTTAAGTCGACCGCCTGGGGAGTACGGCCGCAAGGTTA
AAACTCAAATGAATTGACGGGGGCCCGCACAAGCGGTGGAGCATGTGGTTTAATTCGAT
GCAACGCGAAGAACCTTACCTACTCTTGACATCCACAGAACTTAGCAGAGATGCTTCGG
TGCCTTCGGGAACTGTGAGACAGGTGCTGCATGGCTGTCGTCAGCTCGTGTTGTGAAAT
GTTGGGTTAAGTCCCGCAACGAGCGCAACCCTTATCCTTTTTTGCCAGCACGTAATGGT
GGGAACTCAAGGGAGACTGCCGGTGACAAACCGGAGGAAGGTGGGGATGACGTCAAG
TCATCATGGCCCTTACGAGTAGGGCTACACACGTGCTACAATGGCAGATACAAAGTGAA
GCGAACTCGCGAGAGCAAGCGGACCACATAAAGTCTGTCGTAGTCCGGATTGGAGTCT
GCAACTCGACTCCATGAAGTCGGAATCGCTAGTAATCGTAGATCAGAATGCTACGGTGA
ATACGTTCCCGGGCCTTGTACACACCGCCCGTCACACCATGGGAGTGGGTTGCAAAAG
AAGTAGGTAGCTTAACCTTCGGGAGGGCGCTTACCACTTTGTGATTCATGACTGGGG
pattern 284
CGCTGGCGGCAGGCCTAACACATGCAAGTCGAGCGGCAGCGGGAAGTAGTTTACTACT
TTGCCGGCGAGCGGCGGACGGGTGAGTAATGTCTGGGAAACTGCCTGATGGAGGGGGA
TAACTACTGGAAACGGTAGCTAATACCGCATGACCTCGCAAGAGCAAAGTGGGGGACC
TTCGGGCCTCACGCCATCGGATGTGCCCAGATGGGATTAGCTAGTAGGTGGGGTAATGG
CTCACCTAGGCGACGATCCCTAGCTGGTCTGAGAGGATGACCAGCCACACTGGAACTG
AGACACGGTCCAGACTCCTACGGGAGGCAGCAGTGGGGAATATTGCACAATGGGCGCA

AGCCTGATGCAGCCATGCCGCGTGTGTGAAGAAGGCCTTCGGGTTGTAAAGCACTTTCA
GCGAGGAGGAAGGGTTCAGTGTTAATAGCACTGTTCATTGACGTTACTCGCAGAAGAA
GCACCGGCTAACTCCGTGCCAGCAGCCGCGGTAATACGGAGGGTGCAAGCGTTAATCG
GAATTACTGGGCGTAAAGCGCACGCAGGCGGTTTGTTAAGTCAGATGTGAAATCCCCGC
GCTTAACGTGGGAACTGCATTTGAAACTGGCAAGCTAGAGTCTTGTAGAGGGGGGTAG
AATTCCAGGTGTAGCGGTGAAATGCGTAGAGATCTGGAGGAATACCGGTGGCGAAGGC
GGCCCCCTGGACAAAGACTGACGCTCAGGTGCGAAAGCGTGGGGAGCAAACAGGATT
AGATACCCTGGTAGTCCACGCTGTAAACGATGTCGACTTGGAGGTTGTGCCCTTGAGGC
GTGGCTTCCGGAGCTAACGCGTTAAGTCGACCGCCTGGGGAGTACGGCCGCAAGGTTA
AAACTCAAATGAATTGACGGGGGCCCGCACAAGCGGTGGAGCATGTGGTTTAATTCGAT
GCAACGCGAAGAACCTTACCTACTCTTGACATCCACAGAACTTAGCAGAGATGCTTCGG
TGCCTTCGGGAACTGTGAGACAGGTGCTGCATGGCTGTCGTCAGCTCGTGTTGTGAAAT
GTTGGGTTAAGTCCCGCAACGAGCGCAACCCTTATCCTTTTTTGCCAGCACGTAATGGT
GGGAACTCAAGGGAGACTGCCGGTGACAAACCGGAGGAAGGTGGGGATGACGTCAAG
TCATCATGGCCCTTACGAGTAGGGCTACACACGTGCTACAATGGCAGATACAAAGTGAA
GCGAACTCGCGAGAGCAAGCGGACCACATAAAGTCTGTCGTAGTCCGGATTGGAGTCT
GCAACTCGACTCCATGAAGTCGGAATCGCTAGTAATCGTAGATCAGAATGCTACGGTGA
ATACGTTCCCGGGCCTTGTACACACCGCCCGTCACACCATGGGAGTGGGTTGCAAAAG
AAGTAGGTAGCTTAACCTTCGGGAGGGCGCTTACCACTTTGTGATTCATGACTGGGG
pattern 285
CGCTGGCGGCAGGCCTAACACATGCAAGTCGAGCGGCAGCGGGAAGTAGTTTACTACT
TTGCCGGCGAGCGGCGGACGGGTGAGTAATGTCTGGGAAACTGCCTGATGGAGGGGGA
TAACTACTGGAAACGGTAGCTAATACCGCATGACCTCGCAAGAGCAAAGTGGGGGACC
TTCGGGCCTCACGCCATCGGATGTGCCCAGATGGGATTAGCTAGTAGGTGGGGTAATGG
CTCACCTAGGCGACGATCCCTAGCTGGTCTGAGAGGATGACCAGCCACACTGGAACTG
AGACACGGTCCAGACTCCTACGGGAGGCAGCAGTGGGGAATATTGCACAATGGGCGCA
AGCCTGATGCAGCCATGCCGCGTGTGTGAAGAAGGCCTTCGGGTTGTAAAGCACTTTCA
GCGAGGAGGAAGGCAGTTGTGTTAATAGCACGATTGATTGACGTTACTCGCAGAAGAA
GCACCGGCTAACTCCGTGCCAGCAGCCGCGGTAATACGGAGGGTGCAAGCGTTAATCG
GAATTACTGGGCGTAAAGCGCACGCAGGCGGTTTGTTAAGTCAGATGTGAAATCCCCGC
GCTTAACGTGGGAACTGCATTTGAAACTGGCAAGCTAGAGTCTTGTAGAGGGGGGTAG
AATTCCAGGTGTAGCGGTGAAATGCGTAGAGATCTGGAGGAATACCGGTGGCGAAGGC
GGCCCCCTGGACAAAGACTGACGCTCAGGTGCGAAAGCGTGGGGAGCAAACAGGATT
AGATACCCTGGTAGTCCACGCTGTAAACGATGTCGACTTGGAGGTTGTGCCCTTGAGGC
GTGGCTTCCGGAGCTAACGCGTTAAGTCGACCGCCTGGGGAGTACGGCCGCAAGGTTA
AAACTCAAATGAATTGACGGGGGCCCGCACAAGCGGTGGAGCATGTGGTTTAATTCGAT
GCAACGCGAAGAACCTTACCTACTCTTGACATCCACAGAACTTAGCAGAGATGCTTCGG
TGCCTTCGGGAACTGTGAGACAGGTGCTGCATGGCTGTCGTCAGCTCGTGTTGTGAAAT
GTTGGGTTAAGTCCCGCAACGAGCGCAACCCTTATCCTTTGTTGCCAGCACGTAATGGT
GGGAACTCAAGGGAGACTGCCGGTGACAAACCGGAGGAAGGTGGGGATGACGTCAAG
TCATCATGGCCCTTACGAGTAGGGCTACACACGTGCTACAATGGCAGATACAAAGTGAA
GCGAACTCGCGAGAGCAAGCGGACCACATAAAGTCTGTCGTAGTCCGGATTGGAGTCT
GCAACTCGACTCCATGAAGTCGGAATCGCTAGTAATCGTAGATCAGAATGCTACGGTGA
ATACGTTCCCGGGCCTTGTACACACCGCCCGTCACACCATGGGAGTGGGTTGCAAAAG

AAGTAGGTAGCTTAACCTTCGGGAGGGCGCTTACCACTTTGTGATTCATGACTGGGG
pattern 286
CGCTGGCGGCAGGCCTAACACATGCAAGTCGAGCGGCAGCGGGAAGTAGTTTACTACT
TTGCCGGCGAGCGGCGGACGGGTGAGTAATGTCTGGGGATCTGCCTGATGGAGGGGGA
TAACTACTGGAAACGGTAGCTAATACCGCATGACCTCGCAAGAGCAAAGTGGGGGACC
TTAGGGCCTCACGCCATCGGATGAACCCAGATGGGATTAGCTAGTAGGTGGGGTAATGG
CTCACCTAGGCGACGATCCCTAGCTGGTCTGAGAGGATGACCAGCCACACTGGAACTG
AGACACGGTCCAGACTCCTACGGGAGGCAGCAGTGGGGAATATTGCACAATGGGCGCA
AGCCTGATGCAGCCATGCCGCGTGTGTGAAGAAGGCCTTCGGGTTGTAAAGCACTTTCA
GCGAGGAGGAAGGGGTTGAGTTTAATACGCTCAATCATTGACGTTACTCGCAGAAGAA
GCACCGGCTAACTCCGTGCCAGCAGCCGCGGTAATACGGAGGGTGCAAGCGTTAATCG
GAATTACTGGGCGTAAAGCGCACGCAGGCGGTTTGTTAAGTCAGATGTGAAATCCCCGC
GCTTAACGTGGGAACTGCATTTGAAACTGGCAAGCTAGAGTCTTGTAGAGGGGGGTAG
AATTCCAGGTGTAGCGGTGAAATGCGTAGAGATCTGGAGGAATACCGGTGGCGAAGGC
GGCCCCCTGGACAAAGACTGACGCTCAGGTGCGAAAGCGTGGGGAGCAAACAGGATT
AGATACCCTGGTAGTCCACGCTGTAAACGATGTCGACTTGGAGGTTGTGCCCTTGAGGC
GTGGCTTCCGGAGCTAACGCGTTAAGTCGACCGCCTGGGGAGTACGGCCGCAAGGTTA
AAACTCAAATGAATTGACGGGGGCCCGCACAAGCGGTGGAGCATGTGGTTTAATTCGAT
GCAACGCGAAGAACCTTACCTACTCTTGACATCCACAGAATTTGGCAGAGATGCTAAAG
TGCCTTCGGGAACTGTGAGACAGGTGCTGCATGGCTGTCGTCAGCTCGTGTTGTGAAAT
GTTGGGTTAAGTCCCGCAACGAGCGCAACCCTTATCCTTTGTTGCCAGCACGTAATGGT
GGGAACTCAAGGGAGACTGCCGGTGACAAACCGGAGGAAGGTGGGGATGACGTCAAG
TCATCATGGCCCTTACGAGTAGGGCTACACACGTGCTACAATGGCAGATACAAAGTGAA
GCGAACTCGCGAGAGCCAGCGGACCACATAAAGTCTGTCGTAGTCCGGATTGGAGTCT
GCAACTCGACTCCATGAAGTCGGAATCGCTAGTAATCGTAGATCAGAATGCTACGGTGA
ATACGTTCCCGGGCCTTGTACACACCGCCCGTCACACCATGGGAGTGGGTTGCAAAAG
AAGTAGGTAGCTTAACCTTCGGGAGAGCGCTTACCACTTTGTGATTCATGACTGGGG
pattern 287
CGCTGGCGGCAGGCCTAACACATGCAAGTCGAGCGGCAGCGGGAAGTAGTTTACTACT
TTGCCGGCGAGCGGCGGACGGGTGAGTAATGTCTGGGGATCTGCCTGATGGAGGGGGA
TAACTACTGGAAACGGTAGCTAATACCGCATGACCTCGCAAGAGCAAAGTGGGGGACC
TTCGGGCCTCACGCCATCGGATGAACCCAGATGGGATTAGCTAGTAGGTGGGGTAATGG
CTCACCTAGGCGACGATCCCTAGCTGGTCTGAGAGGATGACCAGCCACACTGGAACTG
AGACACGGTCCAGACTCCTACGGGAGGCAGCAGTGGGGAATATTGCACAATGGGCGCA
AGCCTGATGCAGCCATGCCGCGTGTGTGAAGAAGGCCTTCGGGTTGTAAAGCACTTTCA
GCGAGGAGGAAGGGGTTGAGTTTAATACGCTTAATCATTGACGTTACTCGCAGAAGAAG
CACCGGCTAACTCCGTGCCAGCAGCCGCGGTAATACGGAGGGTGCAAGCGTTAATCGG
AATTACTGGGCGTAAAGCGCACGCAGGCGGTTTGTTAAGTCAGATGTGAAATCCCCGCG
CTTAACGTGGGAACTGCATTTGAAACTGGCAAGCTAGAGTCTTGTAGAGGGGGGTAGA
ATTCCAGGTGTAGCGGTGAAATGCGTAGAGATCTGGAGGAATACCGGTGGCGAAGGCG
GCCCCCTGGACAAAGACTGACGCTCAGGTGCGAAAGCGTGGGGAGCAAACAGGATTA
GATACCCTGGTAGTCCACGCTGTAAACGATGTCGACTTGGAGGTTGTGCCCTTGAGGCG
TGGCTTCCGGAGCTAACGCGTTAAGTCGACCGCCTGGGGAGTACGGCCGCAAGGTTAA
AACTCAAATGAATTGACGGGGGCCCGCACAAGCGGTGGAGCATGTGGTTTAATTCGATG

CAACGCGAAGAACCTTACCTACTCTTGACATCCACAGAATTTGGCAGAGATGCTAAAGT
GCCTTCGGGAACTGTGAGACAGGTGCTGCATGGCTGTCGTCAGCTCGTGTTGTGAAATG
TTGGGTTAAGTCCCGCAACGAGCGCAACCCTTATCCTTTGTTGCCAGCACGTAATGGTG
GGAACTCAAGGGAGACTGCCGGTGACAAACCGGAGGAAGGTGGGGATGACGTCAAGT
CATCATGGCCCTTACGAGTAGGGCTACACACGTGCTACAATGGCAGATACAAAGTGAAG
CGAACTCGCGAGAGCCAGCGGACCACATAAAGTCTGTCGTAGTCCGGATTGGAGTCTG
CAACTCGACTCCATGAAGTCGGAATCGCTAGTAATCGTAGATCAGAATGCTACGGTGAAT
ACGTTCCCGGGCCTTGTACACACCGCCCGTCACACCATGGGAGTGGGTTGCAAAAGAA
GTAGGTAGCTTAACCTTCGGGAGGGCGCTTACCACTTTGTGATTCATGACTGGGG
pattern 288
CGCTGGCGGCAGGCCTAACACATGCAAGTCGAGCGGCAGCGGGAAGTAGTTTACTACT
TTGCCGGCGAGCGGCGGACGGGTGAGTAATGTCTGGGGATCTGCCTGATGGAGGGGGA
TAACTACTGGAAACGGTAGCTAATACCGCATGACCTCGCAAGAGCAAAGTGGGGGACC
TTAGGGCCTCACGCCATCGGATGAACCCAGATGGGATTAGCTAGTAGGTGGGGTAATGG
CTCACCTAGGCGACGATCCCTAGCTGGTCTGAGAGGATGACCAGCCACACTGGAACTG
AGACACGGTCCAGACTCCTACGGGAGGCAGCAGTGGGGAATATTGCACAATGGGCGCA
AGCCTGATGCAGCCATGCCGCGTGTGTGAAGAAGGCCTTCGGGTTGTAAAGCACTTTCA
GCGAGGAGGAAGGGGTTGAGTTTAATACGCTTAATCATTGACGTTACTCGCAGAAGAAG
CACCGGCTAACTCCGTGCCAGCAGCCGCGGTAATACGGAGGGTGCAAGCGTTAATCGG
AATTACTGGGCGTAAAGCGCACGCAGGCGGTTTGTTAAGTCAGATGTGAAATCCCCGCG
CTTAACGTGGGAACTGCATTTGAAACTGGCAAGCTAGAGTCTTGTAGAGGGGGGTAGA
ATTCCAGGTGTAGCGGTGAAATGCGTAGAGATCTGGAGGAATACCGGTGGCGAAGGCG
GCCCCCTGGACAAAGACTGACGCTCAGGTGCGAAAGCGTGGGGAGCAAACAGGATTA
GATACCCTGGTAGTCCACGCTGTAAACGATGTCGACTTGGAGGTTGTGCCCTTGAGGCG
TGGCTTCCGGAGCTAACGCGTTAAGTCGACCGCCTGGGGAGTACGGCCGCAAGGTTAA
AACTCAAATGAATTGACGGGGGCCCGCACAAGCGGTGGAGCATGTGGTTTAATTCGATG
CAACGCGAAGAACCTTACCTACTCTTGACATCCACAGGATTTGGCAGAGATGCCTTAGT
GCCTTCGGGAACTGTGAGACAGGTGCTGCATGGCTGTCGTCAGCTCGTGTTGTGAAATG
TTGGGTTAAGTCCCGCAACGAGCGCAACCCTTATCCTTTGTTGCCAGCACGTAATGGTG
GGAACTCAAGGGAGACTGCCGGTGACAAACCGGAGGAAGGTGGGGATGACGTCAAGT
CATCATGGCCCTTACGAGTAGGGCTACACACGTGCTACAATGGCAGATACAAAGTGAAG
CGAACTCGCGAGAGCCAGCGGACCACATAAAGTCTGTCGTAGTCCGGATTGGAGTCTG
CAACTCGACTCCATGAAGTCGGAATCGCTAGTAATCGTAGATCAGAATGCTACGGTGAAT
ACGTTCCCGGGCCTTGTACACACCGCCCGTCACACCATGGGAGTGGGTTGCAAAAGAA
GCAGGTAGCTTAACCTTCGGGAGGGCGCTTACCACTTTGTGATTCATGACTGGGG
pattern 289
CGCTGGCGGCAGGCCTAACACATGCAAGTCGAGCGGCAGCGGGAAGTAGTTTACTACT
TTGCCGGCGAGCGGCGGACGGGTGAGTAATGTCTGGGGATCTGCCTGATGGAGGGGGA
TAACTACTGGAAACGGTAGCTAATACCGCATGACCTCGCAAGAGCAAAGTGGGGGACC
TTAGGGCCTCACGCCATCGGATGAACCCAGATGGGATTAGCTAGTAGGTGGGGTAATGG
CTCACCTAGGCGACGATCCCTAGCTGGTCTGAGAGGATGACCAGCCACACTGGAACTG
AGACACGGTCCAGACTCCTACGGGAGGCAGCAGTGGGGAATATTGCACAATGGGCGCA
AGCCTGATGCAGCCATGCCGCGTGTGTGAAGAAGGCCTTCGGGTTGTAAAGCACTTTCA
GCGAGGAGGAAGGGGTTGAGTTTAATACGCTTAATCATTGACGTTACTCGCAGAAGAAG

CACCGGCTAACTCCGTGCCAGCAGCCGCGGTAATACGGAGGGTGCAAGCGTTAATCGG
AATTACTGGGCGTAAAGCGCACGCAGGCGGTTTGTTAAGTCAGATGTGAAATCCCCGCG
CTTAACGTGGGAACTGCATTTGAAACTGGCAAGCTAGAGTCTTGTAGAGGGGGGTAGA
ATTCCAGGTGTAGCGGTGAAATGCGTAGAGATCTGGAGGAATACCGGTGGCGAAGGCG
GCCCCCTGGACAAAGACTGACGCTCAGGTGCGAAAGCGTGGGGAGCAAACAGGATTA
GATACCCTGGTAGTCCACGCTGTAAACGATGTCGACTTGGAGGTTGTGCCCTTGAGGCG
TGGCTTCCGGAGCTAACGCGTTAAGTCGACCGCCTGGGGAGTACGGCCGCAAGGTTAA
AACTCAAATGAATTGACGGGGGCCCGCACAAGCGGTGGAGCATGTGGTTTAATTCGATG
CAACGCGAAGAACCTTACCTACTCTTGACATCCACAGGATTTGGCAGAGATGCCTTAGT
GCCTTCGGGAACTGTGAGACAGGTGCTGCATGGCTGTCGTCAGCTCGTGTTGTGAAATG
TTGGGTTAAGTCCCGCAACGAGCGCAACCCTTATCCTTTGTTGCCAGCACGTAATGGTG
GGAACTCAAGGGAGACTGCCGGTGACAAACCGGAGGAAGGTGGGGATGACGTCAAGT
CATCATGGCCCTTACGAGTAGGGCTACACACGTGCTACAATGGCAGATACAAAGTGAAG
CGAACTCGCGAGAGCCAGCGGACCACATAAAGTCTGTCGTAGTCCGGATTGGAGTCTG
CAACTCGACTCCATGAAGTCGGAATCGCTAGTAATCGTAGATCAGAATGCTACGGTGAAT
ACGTTCCCGGGCCTTGTACACACCGCCCGTCACACCATGGGAGTGGGTTGCAAAAGAA
GTAGGTAGCTTAACCTTCGGGAGGGCGCTTACCACTTTGTGATTCATGACTGGGG
pattern 290
CGCTGGCGGCAGGCCTAACACATGCAAGTCGAGCGGCAGCGGGGAGTAGTTTACTACT
CTGCCGGCGAGCGGCGGACGGGTGAGTAATGTCTGGGGATCTGCCTGATGGAGGGGGA
TAACTACTGGAAACGGTAGCTAATACCGCATGACCTCGTAAGAGCAAAGTGGGGGACCT
TCGGGCCTCACGCCATCGGATGAACCCAGATGGGATTAGCTAGTAGGTGAGGTAATGGC
TCACCTAGGCGACGATCCCTAGCTGGTCTGAGAGGATGACCAGCCACACTGGAACTGA
GACACGGTCCAGACTCCTACGGGAGGCAGCAGTGGGGAATATTGCACAATGGGCGCAA
GCCTGATGCAGCCATGCCGCGTGTGTGAAGAAGGCCTTCGGGTTGTAAAGCACTTTCAG
CGAGGAGGAAGGCAGTCGTGTTAATAGCACGATTGATTGACGTTACTCGCAGAAGAAG
CACCGGCTAACTCCGTGCCAGCAGCCGCGGTAATACGGAGGGTGCAAGCGTTAATCGG
AATTACTGGGCGTAAAGCGCACGCAGGCGGTTTGTTAAGTCAGATGTGAAATCCCCGCG
CTTAACGTGGGAACTGCATTTGAAACTGGCAAGCTAGAGTCTTGTAGAGGGGGGTAGA
ATTCCAGGTGTAGCGGTGAAATGCGTAGAGATCTGGAGGAATACCGGTGGCGAAGGCG
GCCCCCTGGACAAAGACTGACGCTCAGGTGCGAAAGCGTGGGGAGCAAACAGGATTA
GATACCCTGGTAGTCCACGCTGTAAACGATGTCGACTTGGAGGTTGTGCCCTTGAGGCG
TGGCTTCCGGAGCTAACGCGTTAAGTCGACCGCCTGGGGAGTACGGCCGCAAGGTTAA
AACTCAAATGAATTGACGGGGGCCCGCACAAGCGGTGGAGCATGTGGTTTAATTCGATG
CAACGCGAAGAACCTTACCTACTCTTGACATCCACGGAATTTAGCAGAGATGCTTAAGT
GCCTTCGGGAACCGTGAGACAGGTGCTGCATGGCTGTCGTCAGCTCGTGTTGTGAAATG
TTGGGTTAAGTCCCGCAACGAGCGCAACCCTTATCCTTTGTTGCCAGCACGTAATGGTG
GGAACTCAAGGGAGACTGCCGGTGACAAACCGGAGGAAGGTGGGGATGACGTCAAGT
CATCATGGCCCTTACGAGTAGGGCTACACACGTGCTACAATGGCAGATACAAAGTGAAG
CGAACTCGCGAGAGCAAGCGGACCACATAAAGTCTGTCGTAGTCCGGATTGGAGTCTG
CAACTCGACTCCATGAAGTCGGAATCGCTAGTAATCGTAGATCAGAATGCTACGGTGAAT
ACGTTCCCGGGCCTTGTACACACCGCCCGTCACACCATGGGAGTGGGTTGCAAAAGAA
GTAGGTAGCTTAACCTTCGGGAGGGCGCTTACCACTTTGTGATTCATGACTGGGG
pattern 291

CGCTGGCGGCAGGCCTAACACATGCAAGTCGAGCGGCAGCGGAAAGTAGCTTGCTACT
TTGCCGGCGAGCGGCGGACGGGTGAGTAATGTCTGGGAAACTGCCTGATGGAGGGGGA
TAACTACTGGAAACGGTAGCTAATACCGCATGACCTCGAAAGAGCAAAGTGGGGGACC
TTTGGGCCTCACGCCATCGGATGTGCCCAGATGGGATTAGCTAGTAGGTGAGGTAATGG
CTCACCTAGGCGACGATCCCTAGCTGGTCTGAGAGGATGACCAGCCACACTGGAACTG
AGACACGGTCCAGACTCCTACGGGAGGCAGCAGTGGGGAATATTGCACAATGGGCGCA
AGCCTGATGCAGCCATGCCGCGTGTGTGAAGAAGGCCTTCGGGTTGTAAAGCACTTTCA
GCGAGGAGGAAGGCATCTTACTTAATACGTGAGGTGATTGACGTTACTCGCAGAAGAA
GCACCGGCTAACTCCGTGCCAGCAGCCGCGGTAATACGGAGGGTGCAAGCGTTAATCG
GAATTACTGGGCGTAAAGCGCACGCAGGCGGTTTGTTAAGTCAGATGTGAAATCCCCGA
GCTTAACTTGGGAACTGCATTTGAAACTGGCAAGCTAGAGTCTTGTAGAGGGGGGTAG
AATTCCAGGTGTAGCGGTGAAATGCGTAGAGATCTGGAGGAATACCGGTGGCGAAGGC
GGCCCCCTGGACAAAGACTGACGCTCAGGTGCGAAAGCGTGGGGAGCAAACAGGATT
AGATACCCTGGTAGTCCACGCTGTAAACGATGTCGACTTGGAGGTTGTGCCCTTGAGGC
GTGGCTTCCGGAGCTAACGCGTTAAGTCGACCGCCTGGGGAGTACGGCCGCAAGGTTA
AAACTCAAATGAATTGACGGGGGCCCGCACAAGCGGTGGAGCATGTGGTTTAATTCGAT
GCAACGCGAAGAACCTTACCTACTCTTGACATCCACAGAACTTAGCAGAGATGCTTAGG
TGCCTTCGGGAACTGTGAGACAGGTGCTGCATGGCTGTCGTCAGCTCGTGTTGTGAAAT
GTTGGGTTAAGTCCCGCAACGAGCGCAACCCTTATCCTTTGTTGCCAGCACGTAATGGT
GGGAACTCAAAGGAGACTGCCGGTGATAAACCGGAGGAAGGTGGGGATGACGTCAAG
TCATCATGGCCCTTACGAGTAGGGCTACACACGTGCTACAATGGCAGATACAAAGTGAA
GCGAACTCGCGAGAGCAAGCGGACCACATAAAGTCTGTCGTAGTCCGGATTGGAGTCT
GCAACTCGACTCCATGAAGTCGGAATCGCTAGTAATCGTAGATCAGAATGCTACGGTGA
ATACGTTCCCGGGCCTTGTACACACCGCCCGTCACACCATGGGAGTGGGTTGCAAAAG
AAGTAGGTAGCTTAACCTTCGGGAGGGCGCTTACCACTTTGTGATTCATGACTGGGG
pattern 292
CGCTGGCGGCAGGCCTAACACATGCAAGTCGAGCGGCAGCGGAAAGTAGCTTGCTACT
TTGCCGGCGAGCGGCGGACGGGTGAGTAATGTCTGGGAAACTGCCTGATGGAGGGGGA
TAACTACTGGAAACGGTAGCTAATACCGCATGACCTCGAAAGAGCAAAGTGGGGGACC
TTTGGGCCTCACGCCATCGGATGTGCCCAGATGGGATTAGCTAGTAGGTGAGGTAATGG
CTCACCTAGGCGACGATCCCTAGCTGGTCTGAGAGGATGACCAGCCACACTGGAACTG
AGACACGGTCCAGACTCCTACGGGAGGCAGCAGTGGGGAATATTGCACAATGGGCGCA
AGCCTGATGCAGCCATGCCGCGTGTGTGAAGAAGGCCTTCGGGTTGTAAAGCACTTTCA
GCGAGGAGGAAGGCATTTCACTTAATACGTGAAGTGATTGACGTTACTCGCAGAAGAA
GCACCGGCTAACTCCGTGCCAGCAGCCGCGGTAATACGGAGGGTGCAAGCGTTAATCG
GAATTACTGGGCGTAAAGCGCACGCAGGCGGTTTGTTAAGTCAGATGTGAAATCCCCGA
GCTTAACTTGGGAACTGCATTTGAAACTGGCAAGCTAGAGTCTTGTAGAGGGGGGTAG
AATTCCAGGTGTAGCGGTGAAATGCGTAGAGATCTGGAGGAATACCGGTGGCGAAGGC
GGCCCCCTGGACAAAGACTGACGCTCAGGTGCGAAAGCGTGGGGAGCAAACAGGATT
AGATACCCTGGTAGTCCACGCTGTAAACGATGTCGACTTGGAGGTTGTGCCCTTGAGGC
GTGGCTTCCGGAGCTAACGCGTTAAGTCGACCGCCTGGGGAGTACGGCCGCAAGGTTA
AAACTCAAATGAATTGACGGGGGCCCGCACAAGCGGTGGAGCATGTGGTTTAATTCGAT
GCAACGCGAAGAACCTTACCTACTCTTGACATCCACAGAACTTAGCAGAGATGCTTAGG
TGCCTTCGGGAACTGTGAGACAGGTGCTGCATGGCTGTCGTCAGCTCGTGTTGTGAAAT

GTTGGGTTAAGTCCCGCAACGAGCGCAACCCTTATCCTTTGTTGCCAGCACGTGATGGT
GGGAACTCAAAGGAGACTGCCGGTGATAAACCGGAGGAAGGTGGGGATGACGTCAAG
TCATCATGGCCCTTACGAGTAGGGCTACACACGTGCTACAATGGCAGATACAAAGTGAA
GCGAACTCGCGAGAGCAAGCGGACCACATAAAGTCTGTCGTAGTCCGGATTGGAGTCT
GCAACTCGACTCCATGAAGTCGGAATCGCTAGTAATCGTAGATCAGAATGCTACGGTGA
ATACGTTCCCGGGCCTTGTACACACCGCCCGTCACACCATGGGAGTGGGTTGCAAAAG
AAGTAGGTAGCTTAACCTTCGGGAGGGCGCTTACCACTTTGTGATTCATGACTGGGG

pattern 293

CGCTGGCGGCAGGCCTAACACATGCAAGTCGAGCGGCAGCGGAAAGTAGCTTGCTACT
TTGCCGGCGAGCGGCGGACGGGTGAGTAATGTCTGGGAAACTGCCTGATGGAGGGGGA
TAACTACTGGAAACGGTAGCTAATACCGCATGACCTCGAAAGAGCAAAGTGGGGGACC
TTCGGGCCTCACGCCATCGGATGTGCCCAGATGGGATTAGCTAGTAGGTGAGGTAATGG
CTCACCTAGGCGACGATCCCTAGCTGGTCTGAGAGGATGACCAGCCACACTGGAACTG
AGACACGGTCCAGACTCCTACGGGAGGCAGCAGTGGGGAATATTGCACAATGGGCGCA
AGCCTGATGCAGCCATGCCGCGTGTGTGAAGAAGGCCTTCGGGTTGTAAAGCACTTTCA
GCGAGGAGGAAGGCATCTTACTTAATACGTGAGGTGATTGACGTTACTCGCAGAAGAA
GCACCGGCTAACTCCGTGCCAGCAGCCGCGGTAATACGGAGGGTGCAAGCGTTAATCG
GAATTACTGGGCGTAAAGCGCACGCAGGCGGTTTGTTAAGTCAGATGTGAAATCCCCGA
GCTTAACTTGGGAACTGCATTTGAAACTGGCAAGCTAGAGTCTTGTAGAGGGGGGTAG
AATTCCAGGTGTAGCGGTGAAATGCGTAGAGATCTGGAGGAATACCGGTGGCGAAGGC
GGCCCCCTGGACAAAGACTGACGCTCAGGTGCGAAAGCGTGGGGAGCAAACAGGATT
AGATACCCTGGTAGTCCACGCTGTAAACGATGTCGACTTGGAGGTTGTGCCCTTGAGGC
GTGGCTTCCGGAGCTAACGCGTTAAGTCGACCGCCTGGGGAGTACGGCCGCAAGGTTA
AAACTCAAATGAATTGACGGGGGCCCGCACAAGCGGTGGAGCATGTGGTTTAATTCGAT
GCAACGCGAAGAACCTTACCTACTCTTGACATCCACAGAACTTAGCAGAGATGCTTAGG
TGCCTTCGGGAACTGTGAGACAGGTGCTGCATGGCTGTCGTCAGCTCGTGTTGTGAAAT
GTTGGGTTAAGTCCCGCAACGAGCGCAACCCTTATCCTTTGTTGCCAGCACGTAATGGT
GGGAACTCAAAGGAGACTGCCGGTGATAAACCGGAGGAAGGTGGGGATGACGTCAAG
TCATCATGGCCCTTACGAGTAGGGCTACACACGTGCTACAATGGCAGATACAAAGTGAA
GCGAACTCGCGAGAGCAAGCGGACCACATAAAGTCTGTCGTAGTCCGGATTGGAGTCT
GCAACTCGACTCCATGAAGTCGGAATCGCTAGTAATCGTAGATCAGAATGCTACGGTGA
ATACGTTCCCGGGCCTTGTACACACCGCCCGTCACACCATGGGAGTGGGTTGCAAAAG
AAGTAGGTAGCTTAACCTTCGGGAGGGCGCTTACCACTTTGTGATTCATGACTGGGG

pattern 294

CGCTGGCGGCAGGCCTAACACATGCAAGTCGAGCGGCAGCGGGAAGTAGCTTGCTACT
TTGCCGGCGAGCGGCGGACGGGTGAGTAATGTCTGGGAAACTGCCTGATGGAGGGGGA
TAACTACTGGAAACGGTAGCTAATACCGCATGACCTCGCAAGAGCAAAGTGGGGGACC
TTCGGGCCTCACGCCATCGGATGTGCCCAGATGGGATTAGCTAGTAGGTGGGGTAATGG
CTCACCTAGGCGACGATCCCTAGCTGGTCTGAGAGGATGACCAGCCACACTGGAACTG
AGACACGGTCCAGACTCCTACGGGAGGCAGCAGTGGGGAATATTGCACAATGGGCGCA
AGCCTGATGCAGCCATGCCGCGTGTGTGAAGAAGGCCTTCGGGTTGTAAAGCACTTTCA
GCGAGGAGGAAGGGGTTGAGTTTAATACGCTCAATCATTGACGTTACTCGCAGAAGAA
GCACCGGCTAACTCCGTGCCAGCAGCCGCGGTAATACGGAGGGTGCAAGCGTTAATCG
GAATTACTGGGCGTAAAGCGCACGCAGGCGGTTTGTTAAGTCAGATGTGAAATCCCCGC

GCTTAACGTGGGAACTGCATTTGAAACTGGCAAGCTAGAGTCTTGTAGAGGGGGGTAG
AATTCCAGGTGTAGCGGTGAAATGCGTAGAGATCTGGAGGAATACCGGTGGCGAAGGC
GGCCCCCTGGACAAAGACTGACGCTCAGGTGCGAAAGCGTGGGGAGCAAACAGGATT
AGATACCCTGGTAGTCCACGCTGTAAACGATGTCGACTTGGAGGTTGTGCCCTTGAGGC
GTGGCTTCCGGAGCTAACGCGTTAAGTCGACCGCCTGGGGAGTACGGCCGCAAGGTTA
AAACTCAAATGAATTGACGGGGGCCCGCACAAGCGGTGGAGCATGTGGTTTAATTCGAT
GCAACGCGAAGAACCTTACCTACTCTTGACATCCACAGAACTTAGCAGAGATGCTTAGG
TGCCTTCGGGAACTGTGAGACAGGTGCTGCATGGCTGTCGTCAGCTCGTGTTGTGAAAT
GTTGGGTTAAGTCCCGCAACGAGCGCAACCCTTATCCTTTGTTGCCAGCACGTCATGGT
GGGAACTCAAAGGAGACTGCCGGTGATAAACCGGAGGAAGGTGGGGATGACGTCAAG
TCATCATGGCCCTTACGAGTAGGGCTACACACGTGCTACAATGGCAGATACAAAGTGAA
GCGAACTCGCGAGAGCAAGCGGACCACATAAAGTCTGTCGTAGTCCGGATTGGAGTCT
GCAACTCGACTCCATGAAGTCGGAATCGCTAGTAATCGTAGATCAGAATGCTACGGTGA
ATACGTTCCCGGGCCTTGTACACACCGCCCGTCACACCATGGGAGTGGGTTGCAAAAG
AAGTAGGTAGCTTAACCTTCGGGAGGGCGCTTACCACTTTGTGATTCATGACTGGGG
pattern 295
CGCTGGCGGCAGGCCTAACACATGCAAGTCGAGCGGCAGCGGAAAGTAGCTTGCTACT
TTGCCGGCGAGCGGCGGACGGGTGAGTAATGTCTGGGAAACTGCCTGATGGAGGGGGA
TAACTACTGGAAACGGTAGCTAATACCGCATGACCTCGCAAGAGCAAAGTGGGGGACC
TTCGGGCCTCACGCCATCGGATGTGCCCAGATGGGATTAGCTAGTAGGTGGGGTAATGG
CTCACCTAGGCGACGATCCCTAGCTGGTCTGAGAGGATGACCAGCCACACTGGAACTG
AGACACGGTCCAGACTCCTACGGGAGGCAGCAGTGGGGAATATTGCACAATGGGCGCA
AGCCTGATGCAGCCATGCCGCGTGTGTGAAGAAGGCCTTCGGGTTGTAAAGCACTTTCA
GCGAGGAGGAAGGGGTTGAGTTTAATACGCTCAATCATTGACGTTACTCGCAGAAGAA
GCACCGGCTAACTCCGTGCCAGCAGCCGCGGTAATACGGAGGGTGCAAGCGTTAATCG
GAATTACTGGGCGTAAAGCGCACGCAGGCGGTTTGTTAAGTCAGATGTGAAATCCCCGC
GCTTAACGTGGGAACTGCATTTGAAACTGGCAAGCTAGAGTCTTGTAGAGGGGGGTAG
AATTCCAGGTGTAGCGGTGAAATGCGTAGAGATCTGGAGGAATACCGGTGGCGAAGGC
GGCCCCCTGGACAAAGACTGACGCTCAGGTGCGAAAGCGTGGGGAGCAAACAGGATT
AGATACCCTGGTAGTCCACGCTGTAAACGATGTCGACTTGGAGGTTGTGCCCTTGAGGC
GTGGCTTCCGGAGCTAACGCGTTAAGTCGACCGCCTGGGGAGTACGGCCGCAAGGTTA
AAACTCAAATGAATTGACGGGGGCCCGCACAAGCGGTGGAGCATGTGGTTTAATTCGAT
GCAACGCGAAGAACCTTACCTACTCTTGACATCCACGGAATTTAGCAGAGATGCTTTAG
TGCCTTCGGGAACCGTGAGACAGGTGCTGCATGGCTGTCGTCAGCTCGTGTTGTGAAAT
GTTGGGTTAAGTCCCGCAACGAGCGCAACCCTTATCCTTTGTTGCCAGCACGTCATGGT
GGGAACTCAAAGGAGACTGCCGGTGATAAACCGGAGGAAGGTGGGGATGACGTCAAG
TCATCATGGCCCTTACGAGTAGGGCTACACACGTGCTACAATGGCAGATACAAAGTGAA
GCGAACTCGCGAGAGCAAGCGGACCACATAAAGTCTGTCGTAGTCCGGATTGGAGTCT
GCAACTCGACTCCATGAAGTCGGAATCGCTAGTAATCGTAGATCAGAATGCTACGGTGA
ATACGTTCCCGGGCCTTGTACACACCGCCCGTCACACCATGGGAGTGGGTTGCAAAAG
AAGTAGGTAGCTTAACCTTCGGGAGGGCGCTTACCACTTTGTGATTCATGACTGGGG
pattern 296
CGCTGGCGGCAGGCCTAACACATGCAAGTCGAGCGGCAGCGGGAAGTAGCTTGCTACT
TTGCCGGCGAGCGGCGGACGGGTGAGTAATGTCTGGGAAACTGCCTGATGGAGGGGGA

TAACTACTGGAAACGGTAGCTAATACCGCATGACCTCGCAAGAGCAAAGTGGGGGACC
TTCGGGCCTCACGCCATCGGATGTGCCCAGATGGGATTAGCTAGTAGGTGGGGTAATGG
CTCACCTAGGCGACGATCCCTAGCTGGTCTGAGAGGATGACCAGCCACACTGGAACTG
AGACACGGTCCAGACTCCTACGGGAGGCAGCAGTGGGGAATATTGCACAATGGGCGCA
AGCCTGATGCAGCCATGCCGCGTGTGTGAAGAAGGCCTTCGGGTTGTAAAGCACTTTCA
GCGAGGAGGAAGGGGTTGAGTTTAATACGCTTAATCATTGACGTTACTCGCAGAAGAAG
CACCGGCTAACTCCGTGCCAGCAGCCGCGGTAATACGGAGGGTGCAAGCGTTAATCGG
AATTACTGGGCGTAAAGCGCACGCAGGCGGTTTGTTAAGTCAGATGTGAAATCCCCGCG
CTTAACGTGGGAACTGCATTTGAAACTGGCAAGCTAGAGTCTTGTAGAGGGGGGTAGA
ATTCCAGGTGTAGCGGTGAAATGCGTAGAGATCTGGAGGAATACCGGTGGCGAAGGCG
GCCCCCTGGACAAAGACTGACGCTCAGGTGCGAAAGCGTGGGGAGCAAACAGGATTA
GATACCCTGGTAGTCCACGCTGTAAACGATGTCGACTTGGAGGTTGTGCCCTTGAGGCG
TGGCTTCCGGAGCTAACGCGTTAAGTCGACCGCCTGGGGAGTACGGCCGCAAGGTTAA
AACTCAAATGAATTGACGGGGGCCCGCACAAGCGGTGGAGCATGTGGTTTAATTCGATG
CAACGCGAAGAACCTTACCTACTCTTGACATCCACAGAACTTAGCAGAGATGCTTAGGT
GCCTTCGGGAACTGTGAGACAGGTGCTGCATGGCTGTCGTCAGCTCGTGTTGTGAAATG
TTGGGTTAAGTCCCGCAACGAGCGCAACCCTTATCCTTTGTTGCCAGCACGTCATGGTG
GGAACTCAAAGGAGACTGCCGGTGATAAACCGGAGGAAGGTGGGGATGACGTCAAGT
CATCATGGCCCTTACGAGTAGGGCTACACACGTGCTACAATGGCAGATACAAAGTGAAG
CGAACTCGCGAGAGCAAGCGGACCACATAAAGTCTGTCGTAGTCCGGATTGGAGTCTG
CAACTCGACTCCATGAAGTCGGAATCGCTAGTAATCGTAGATCAGAATGCTACGGTGAAT
ACGTTCCCGGGCCTTGTACACACCGCCCGTCACACCATGGGAGTGGGTTGCAAAAGAA
GTAGGTAGCTTAACCTTCGGGAGGGCGCTTACCACTTTGTGATTCATGACTGGGG
pattern 297
CGCTGGCGGCAGGCCTAACACATGCAAGTCGAGCGGCAGCGGGAAGTAGCTTGCTACT
TTGCCGGCGAGCGGCGGACGGGTGAGTAATGTCTGGGAAACTGCCTGATGGAGGGGGA
TAACTACTGGAAACGGTAGCTAATACCGCATGACCTCGCAAGAGCAAAGTGGGGGACC
TTCGGGCCTCACGCCATCGGATGTGCCCAGATGGGATTAGCTAGTAGGTGGGGTAATGG
CTCACCTAGGCGACGATCCCTAGCTGGTCTGAGAGGATGACCAGCCACACTGGAACTG
AGACACGGTCCAGACTCCTACGGGAGGCAGCAGTGGGGAATATTGCACAATGGGCGCA
AGCCTGATGCAGCCATGCCGCGTGTGTGAAGAAGGCCTTCGGGTTGTAAAGCACTTTCA
GCGAGGAGGAAGGGGTTGAGTTTAATACGCTCAATCATTGACGTTACTCGCAGAAGAA
GCACCGGCTAACTCCGTGCCAGCAGCCGCGGTAATACGGAGGGTGCAAGCGTTAATCG
GAATTACTGGGCGTAAAGCGCACGCAGGCGGTTTGTTAAGTCAGATGTGAAATCCCCGC
GCTTAACGTGGGAACTGCATTTGAAACTGGCAAGCTAGAGTCTTGTAGAGGGGGGTAG
AATTCCAGGTGTAGCGGTGAAATGCGTAGAGATCTGGAGGAATACCGGTGGCGAAGGC
GGCCCCCTGGACAAAGACTGACGCTCAGGTGCGAAAGCGTGGGGAGCAAACAGGATT
AGATACCCTGGTAGTCCACGCTGTAAACGATGTCGACTTGGAGGTTGTGCCCTTGAGGC
GTGGCTTCCGGAGCTAACGCGTTAAGTCGACCGCCTGGGGAGTACGGCCGCAAGGTTA
AAACTCAAATGAATTGACGGGGGCCCGCACAAGCGGTGGAGCATGTGGTTTAATTCGAT
GCAACGCGAAGAACCTTACCTACTCTTGACATCCACGGAATTTAGCAGAGATGCTTTAG
TGCCTTCGGGAACCGTGAGACAGGTGCTGCATGGCTGTCGTCAGCTCGTGTTGTGAAAT
GTTGGGTTAAGTCCCGCAACGAGCGCAACCCTTATCCTTTGTTGCCAGCACGTCATGGT
GGGAACTCAAAGGAGACTGCCGGTGATAAACCGGAGGAAGGTGGGGATGACGTCAAG

TCATCATGGCCCTTACGAGTAGGGCTACACACGTGCTACAATGGCAGATACAAAGTGAA
GCGAACTCGCGAGAGCAAGCGGACCACATAAAGTCTGTCGTAGTCCGGATTGGAGTCT
GCAACTCGACTCCATGAAGTCGGAATCGCTAGTAATCGTAGATCAGAATGCTACGGTTAA
TACGTTCCCGGGCCTTGTACACACCGCCCGTCACACCATGGGAGTGGGTTGCAAAAGA
AGTAGGTAGCTTAACCTTCGGGAGGGCGCTTACCACTTTGTGATTCATGACTGGGG
pattern 298
CGCTGGCGGCAGGCCTAACACATGCAAGTCGAGCGGCAGCGGGAAGTAGTTTACTACT
TTGCCGGCGAGCGGCGGACGGGTGAGTAATGTCTGGGAAACTGCCTGATGGAGGGGGA
TAACTACTGGAAACGGTAGCTAATACCGCATGACCTCGTAAGAGCAAAGTGGGGGACCT
TCGGGCCTCACGCCATCGGATGTGCCCAGATGGGATTAGCTAGTAGGTGGGGTAATGGC
TCACCTAGGCGACGATCCCTAGCTGGTCTGAGAGGATGACCAGCCACACTGGAACTGA
GACACGGTCCAGACTCCTACGGGAGGCAGCAGTGGGGAATATTGCACAATGGGCGCAA
GCCTGATGCAGCCATGCCGCGTGTGTGAAGAAGGCCTTCGGGTTGTAAAGCACTTTCAG
CGAGGAGGAAGGCAGTCGTGTTAATAGCACGATTGATTGACGTTACTCGCAGAAGAAG
CACCGGCTAACTCCGTGCCAGCAGCCGCGGTAATACGGAGGGTGCAAGCGTTAATCGG
AATTACTGGGCGTAAAGCGCACGCAGGCGGTTTGTTAAGTCAGATGTGAAATCCCCGCG
CTTAACGTGGGAACTGCATTTGAAACTGGCAAGCTAGAGTCTTGTAGAGGGGGGTAGA
ATTCCAGGTGTAGCGGTGAAATGCGTAGAGATCTGGAGGAATACCGGTGGCGAAGGCG
GCCCCCTGGACAAAGACTGACGCTCAGGTGCGAAAGCGTGGGGAGCAAACAGGATTA
GATACCCTGGTAGTCCACGCTGTAAACGATGTCGACTTGGAGGTTGTGCCCTTGAGGCG
TGGCTTCCGGAGCTAACGCGTTAAGTCGACCGCCTGGGGAGTACGGCCGCAAGGTTAA
AACTCAAATGAATTGACGGGGGCCCGCACAAGCGGTGGAGCATGTGGTTTAATTCGATG
CAACGCGAAGAACCTTACCTACTCTTGACATCCACGGAATTTAGCAGAGATGCTTTAGT
GCCTTCGGGAACCGTGAGACAGGTGCTGCATGGCTGTCGTCAGCTCGTGTTGTGAAATG
TTGGGTTAAGTCCCGCAACGAGCGCAACCCTTATCCTTTGTTGCCAGCACGTAATGGTG
GGAACTCAAGGGAGACTGCCGGTGACAAACCGGAGGAAGGTGGGGATGACGTCAAGT
CATCATGGCCCTTACGAGTAGGGCTACACACGTGCTACAATGGCAGATACAAAGTGAAG
CGAACTCGCGAGAGCAAGCGGACCACATAAAGTCTGTCGCAGTCCGGATTGGAGTCTG
CAACTCGACTCCATGAAGTCGGAATCGCTAGTAATCGTAGATCAGAATGCTACGGTGAAT
ACGTTCCCGGGCCTTGTACACACCGCCCGTCACACCATGGGAGTGGGTTGCAAAAGAA
GTAGGTAGCTTAACCTTCGGGAGGGCGCTTACCACTTTGTGATTCATGACTGGGG
pattern 299
CGCTGGCGGCAGGCCTAACACATGCAAGTCGAGCGGCAGCGGAAAGTAGCTTGCTACT
TTGCCGGCGAGCGGCGGACGGGTGAGTAATGTCTGGGGATCTGCCTGATGGAGGGGGA
TAACTACTGGAAACGGTAGCTAATACCGCATGACCTCGAAAGAGCAAAGTGGGGGACC
TTCGGGCCTCACGCCATCGGATGAACCTAGATGGGATTAGCTAGTAGGTGGGGTAATGG
CTCACCTAGGCGACGATCCCTAGCTGGTCTGAGAGGATGACCAGCCACACTGGAACTG
AGACACGGTCCAGACTCCTACGGGAGGCAGCAGTGGGGAATATTGCACAATGGGCGAA
AGCCTGATGCAGCCATGCCGCGTGTGTGAAGAAGGCCTTCGGGTTGTAAAGCACTTTCA
GCGAGGAGGAAGGCATTGTGGTTAATAACCGCAGTGATTGACGTTACTCGCAGAAGAA
GCACCGGCTAACTCCGTGCCAGCAGCCGCGGTAATACGGAGGGTGCAAGCGTTAATCG
GAATTACTGGGCGTAAAGCGCACGCAGGCGGTTTGTTAAGTCAGATGTGAAATCCCCGC
GCTTAACGTGGGAACTGCATTTGAAACTGGCAAGCTAGAGTCTTGTAGAGGGGGGTAG
AATTCCAGGTGTAGCGGTGAAATGCGTAGAGATCTGGAGGAATACCGGTGGCGAAGGC

GGCCCCCTGGACAAAGACTGACGCTCAGGTGCGAAAGCGTGGGGAGCAAACAGGATT
AGATACCCTGGTAGTCCACGCTGTAAACGATGTCGACTTGGAGGTTGTGCCCTTGAGGC
GTGGCTTCCGGAGCTAACGCGTTAAGTCGACCGCCTGGGGAGTACGGCCGCAAGGTTA
AAACTCAAATGAATTGACGGGGGCCCGCACAAGCGGTGGAGCATGTGGTTTAATTCGAT
GCAACGCGAAGAACCTTACCTACTCTTGACATCCACGGAATTTAGCAGAGATGCTTTAG
TGCCTTCGGGAACCGTGAGACAGGTGCTGCATGGCTGTCGTCAGCTCGTGTTGTGAAAT
GTTGGGTTAAGTCCCGCAACGAGCGCAACCCTTATCCTTTGTTGCCAGCACGTAATGGT
GGGAACTCAAGGGAGACTGCCGGTGACAAACCGGAGGAAGGTGGGGATGACGTCAAG
TCATCATGGCCCTTACGAGTAGGGCTACACACGTGCTACAATGGCAGATACAAAGTGAA
GCGAACTCGCGAGAGCAAGCGGACCACATAAAGTCTGTCGTAGTCCGGATTGGAGTCT
GCAACTCGACTCCATGAAGTCGGAATCGCTAGTAATCGTAGATCAGAATGCTACGGTGA
ATACGTTCCCGGGCCTTGTACACACCGCCCGTCACACCATGGGAGTGGGTTGCAAAAG
AAGTAGGTAGCTTAACCTTCGGGAGGGCGCTTACCACTTTGTGATTCATGACTGGGG

pattern 300

CGCTGGCGGCAGGCCTAACACATGCAAGTCGAGCGGCAGCGGAAAGTAGCTTGCTACT
TTGCCGGCGAGCGGCGGACGGGTGAGTAATGTCTGGGGATCTGCCTGATGGAGGGGGA
TAACTACTGGAAACGGTAGCTAATACCGCATGACCTCGAAAGAGCAAAGTGGGGGACC
TTCGGGCCTCACGCCATCGGATGAACCCAGATGGGATTAGCTAGTAGGTGAGGTAATGG
CTCACCTAGGCGACGATCCCTAGCTGGTCTGAGAGGATGACCAGCCACACTGGAACTG
AGACACGGTCCAGACTCCTACGGGAGGCAGCAGTGGGGAATATTGCACAATGGGCGCA
AGCCTGATGCAGCCATGCCGCGTGTGTGAAGAAGGCCTTCGGGTTGTAAAGCACTTTCA
GCGAGGAGGAAGGCATTGTGGTTAATAACCGCAGTGATTGACGTTACTCGCAGAAGAA
GCACCGGCTAACTCCGTGCCAGCAGCCGCGGTAATACGGAGGGTGCAAGCGTTAATCG
GAATTACTGGGCGTAAAGCGCACGCAGGCGGTTTGTTAAGTCAGATGTGAAATCCCCGC
GCTTAACGTGGGAACTGCATTTGAAACTGGCAAGCTAGAGTCTTGTAGAGGGGGGTAG
AATTCCAGGTGTAGCGGTGAAATGCGTAGAGATCTGGAGGAATACCGGTGGCGAAGGC
GGCCCCCTGGACAAAGACTGACGCTCAGGTGCGAAAGCGTGGGGAGCAAACAGGATT
AGATACCCTGGTAGTCCACGCTGTAAACGATGTCGACTTGGAGGTTGTGCCCTTGAGGC
GTGGCTTCCGGAGCTAACGCGTTAAGTCGACCGCCTGGGGAGTACGGCCGCAAGGTTA
AAACTCAAATGAATTGACGGGGGCCCGCACAAGCGGTGGAGCATGTGGTTTAATTCGAT
GCAACGCGAAGAACCTTACCTACTCTTGACATCCACGGAATTTAGCAGAGATGCTTTAG
TGCCTTCGGGAACCGTGAGACAGGTGCTGCATGGCTGTCGTCAGCTCGTGTTGTGAAAT
GTTGGGTTAAGTCCCGCAACGAGCGCAACCCTTATCCTTTGTTGCCAGCACGTAATGGT
GGGAACTCAAGGGAGACTGCCGGTGACAAACCGGAGGAAGGTGGGGATGACGTCAAG
TCATCATGGCCCTTACGAGTAGGGCTACACACGTGCTACAATGGCAGATACAAAGTGAA
GCGAACTCGCGAGAGCAAGCAGACCACATAAAGTCTGTCGTAGTCCGGATTGGAGTCT
GCAACTCGACTCCATGAAGTCGGAATCGCTAGTAATCGTAGATCAGAATGCTACGGTGA
ATACGTTCCCGGGCCTTGTACACACCGCCCGTCACACCATGGGAGTGGGTTGCAAAAG
AAGTAGGTAGCTTAACCTTCGGGAGGGCGCTTACCACTTTGTGATTCATGACTGGGG

pattern 301

CGCTGGCGGCAGGCCTAACACATGCAAGTCGAGCGGCAGCGGGGAGTAGTTTGCTACT
TTGCCGGCGAGCGGCGGACGGGTGAGTAATGTCTGGGAAACTGCCTGATGGAGGGGGA
TAACTACTGGAAACGGTAGCTAATACCGCATAACGTCTTCGGACCAAAGTGGGGGACCT
TCGGGCCTCACGCCATCGGATGTGCCCAGATGGGATTAGCTAGTAGGTGGGGTAATGGC

TCACCTAGGCGACGATCCCTAGCTGGTCTGAGAGGATGACCAGCCACACTGGAACTGA
GACACGGTCCAGACTCCTACGGGAGGCAGCAGTGGGGAATATTGCACAATGGGCGCAA
GCCTGATGCAGCCATGCCGCGTGTGTGAAGAAGGCCTTCGGGTTGTAAAGCACTTTCAG
CGAGGAGGAAGGCATAAAGGTTAATAACCTTTGTGATTGACGTTACTCGCAGAAGAAG
CACCGGCTAACTCCGTGCCAGCAGCCGCGGTAATACGGAGGGTGCAAGCGTTAATCGG
AATTACTGGGCGTAAAGCGCACGCAGGCGGTTTGTTAAGTCAGATGTGAAATCCCCGCG
CTTAACGTGGGAACTGCATTTGAAACTGGCAAGCTAGAGTCTTGTAGAGGGGGGTAGA
ATTCCAGGTGTAGCGGTGAAATGCGTAGAGATCTGGAGGAATACCGGTGGCGAAGGCG
GCCCCCTGGACAAAGACTGACGCTCAGGTGCGAAAGCGTGGGGAGCAAACAGGATTA
GATACCCTGGTAGTCCACGCTGTAAACGATGTCGACTTGGAGGTTGTGCCCTTGAGGCG
TGGCTTCCGGAGCTAACGCGTTAAGTCGACCGCCTGGGGAGTACGGCCGCAAGGTTAA
AACTCAAATGAATTGACGGGGGCCCGCACAAGCGGTGGAGCATGTGGTTTAATTCGATG
CAACGCGAAGAACCTTACCTACTCTTGACATCCACAGAACTTAGCAGAGATGCTTCGGT
GCCTTCGGGAACTGTGAGACAGGTGCTGCATGGCTGTCGTCAGCTCGTGTTGTGAAATG
TTGGGTTAAGTCCCGCAACGAGCGCAACCCTTATCCTTTGTTGCCAGCACGTCATGGTG
GGAACTCAAAGGAGACTGCCGGTGACAAACCGGAGGAAGGTGGGGATGACGTCAAGT
CATCATGGCCCTTACGAGTAGGGCTACACACGTGCTACAATGGCAGATACAAAGTGAAG
CGAACTCGCGAGAGCAAGCGGACCACATAAAGTCTGTCGTAGTCCGGATTGGAGTCTG
CAACTCGACTCCATGAAGTCGGAATCGCTAGTAATCGTAGATCAGAATGCTACGGTGAAT
ACGTTCCCGGGCCTTGTACACACCGCCCGTCACACCATGGGAGTGGGTTGCAAAAGAA
GTAGGTAGCTTAACCTTCGGGAGGGCGCTTACCACTTTGTGATTCATGACTGGGG
pattern 302
CGCTGGCGGCAGGCCTAACACATGCAAGTCGAGCGGCAGCGGGAAGTAGTTTACTACT
TTGCCGGCGAGCGGCGGACGGGTGAGTAATGTCTGGGAAACTGCCTGATGGAGGGGGA
TAACTACTGGAAACGGTAGCTAATACCGCATAACGTCTACGGACCAAAGTGGGGGACCT
TCGGGCCTCACGCCATCGGATGTGCCCAGATGGGATTAGCTAGTAGGTGGGGTAATGGC
TCACCTAGGCGACGATCCCTAGCTGGTCTGAGAGGATGACCAGCCACACTGGAACTGA
GACACGGTCCAGACTCCTACGGGAGGCAGCAGTGGGGAATATTGCACAATGGGCGCAA
GCCTGATGCAGCCATGCCGCGTGTGTGAAGAAGGCCTTCGGGTTGTAAAGCACTTTCAG
CGAGGAGGAAGGCATAAAGGTTAATAACCTTTGTGATTGACGTTACTCGCAGAAGAAG
CACCGGCTAACTCCGTGCCAGCAGCCGCGGTAATACGGAGGGTGCAAGCGTTAATCGG
AATTACTGGGCGTAAAGCGCACGCAGGCGGTTTGTTAAGTCAGATGTGAAATCCCCGCG
CTTAACGTGGGAACTGCATTTGAAACTGGCAAGCTAGAGTCTTGTAGAGGGGGGTAGA
ATTCCAGGTGTAGCGGTGAAATGCGTAGAGATCTGGAGGAATACCGGTGGCGAAGGCG
GCCCCCTGGACAAAGACTGACGCTCAGGTGCGAAAGCGTGGGGAGCAAACAGGATTA
GATACCCTGGTAGTCCACGCTGTAAACGATGTCGACTTGGAGGTTGTGCCCTTGAGGCG
TGGCTTCCGGAGCTAACGCGTTAAGTCGACCGCCTGGGGAGTACGGCCGCAAGGTTAA
AACTCAAATGAATTGACGGGGGCCCGCACAAGCGGTGGAGCATGTGGTTTAATTCGATG
CAACGCGAAGAACCTTACCTACTCTTGACATCCACGGAATTTAGCAGAGATGCTTTAGT
GCCTTCGGGAACCGTGAGACAGGTGCTGCATGGCTGTCGTCAGCTCGTGTTGTGAAATG
TTGGGTTAAGTCCCGCAACGAGCGCAACCCTTATCCTTTGTTGCCAGCACGTAATGGTG
GGAACTCAAAGGAGACTGCCGGTGACAAACCGGAGGAAGGTGGGGATGACGTCAAGT
CATCATGGCCCTTACGAGTAGGGCTACACACGTGCTACAATGGCAGATACAAAGTGAAG
CGAACTCGCGAGAGCAAGCGGACCACATAAAGTCTGTCGTAGTCCGGATTGGAGTCTG

CAACTCGACTCCATGAAGTCGGAATCGCTAGTAATCGTAGATCAGAATGCTACGGTGAAT
ACGTTCCCGGGCCTTGTACACACCGCCCGTCACACCATGGGAGTGGGTTGCAAAAGAA
GTAGGTAGCTTAACCTTCGGGAGGGCGCTTACCACTTTGTGATTCATGACTGGGG
pattern 303
CGCTGGCGGCAGGCCTAACACATGCAAGTCGAGCGGCAGCGGGGAGTAGTTTACTACT
TTGCCGGCGAGCGGCGGACGGGTGAGTAATGTCTGGGAAACTGCCTGATGGAGGGGGA
TAACTACTGGAAACGGTAGCTAATACCGCATAACGTCTTCGGACCAAAGTGGGGGACCT
TCGGGCCTCACGCCATCGGATGTGCCCAGATGGGATTAGCTAGTAGGTGGGGTAATGGC
TCACCTAGGCGACGATCCCTAGCTGGTCTGAGAGGATGACCAGCCACACTGGAACTGA
GACACGGTCCAGACTCCTACGGGAGGCAGCAGTGGGGAATATTGCACAATGGGCGCAA
GCCTGATGCAGCCATGCCGCGTGTGTGAAGAAGGCCTTCGGGTTGTAAAGCACTTTCAG
CGAGGAGGAAGGCATAAAGGTTAATAACCTTTGTGATTGACGTTACTCGCAGAAGAAG
CACCGGCTAACTCCGTGCCAGCAGCCGCGGTAATACGGAGGGTGCAAGCGTTAATCGG
AATTACTGGGCGTAAAGCGCACGCAGGCGGTTTGTTAAGTCAGATGTGAAATCCCCGCG
CTTAACGTGGGAACTGCATTTGAAACTGGCAAGCTAGAGTCTTGTAGAGGGGGGTAGA
ATTCCAGGTGTAGCGGTGAAATGCGTAGAGATCTGGAGGAATACCGGTGGCGAAGGCG
GCCCCCTGGACAAAGACTGACGCTCAGGTGCGAAAGCGTGGGGAGCAAACAGGATTA
GATACCCTGGTAGTCCACGCTGTAAACGATGTCGACTTGGAGGTTGTGCCCTTGAGGCG
TGGCTTCCGGAGCTAACGCGTTAAGTCGACCGCCTGGGGAGTACGGCCGCAAGGTTAA
AACTCAAATGAATTGACGGGGGCCCGCACAAGCGGTGGAGCATGTGGTTTAATTCGATG
CAACGCGAAGAACCTTACCTACTCTTGACATCCACGGAATTTAGCAGAGATGCTTTAGT
GCCTTCGGGAACCGTGAGACAGGTGCTGCATGGCTGTCGTCAGCTCGTGTTGTGAAATG
TTGGGTTAAGTCCCGCAACGAGCGCAACCCTTATCCTTTGTTGCCAGCGAGTAATGTCG
GGAACTCAAAGGAGACTGCCGGTGATAAACCGGAGGAAGGTGGGGATGACGTCAAGT
CATCATGGCCCTTACGAGTAGGGCTACACACGTGCTACAATGGCAGATACAAAGTGAAG
CGAACTCGCGAGAGCAAGCGGACCACATAAAGTCTGTCGTAGTCCGGATTGGAGTCTG
CAACTCGACTCCATGAAGTCGGAATCGCTAGTAATCGTAGATCAGAATGCTACGGTGAAT
ACGTTCCCGGGCCTTGTACACACCGCCCGTCACACCATGGGAGTGGGTTGCAAAAGAA
GTAGGTAGCTTAACCTTCGGGAGGGCGCTTACCACTTTGTGATTCATGACTGGGG
pattern 304
CGCTGGCGGCAGGCCTAACACATGCAAGTCGAGCGGCAGCGGAAAGTAGCTTGCTACT
TTGCCGGCGAGCGGCGGACGGGTGAGTAATGTCTGGGGATCTGCCTGATGGAGGGGGA
TAACTACTGGAAACGGTAGCTAATACCGCATGACCTCGAAAGAGCAAAGTGGGGGACC
TTCGGGCCTCACGCCATCGGATGAACCCAGATGGGATTAGCTAGTAGGTGGGGTAATGG
CTCACCTAGGCGACGATCCGTAACTGGTCTGAGAGGATGATCAGTCACACTGGAACTGA
GACACGGTCCAGACTCCTACGGGAGGCAGCAGTGGGGAATATTGCACAATGGGCGCAA
GCCTGATGCAGCCATGCCGCGTGTGTGAAGAAGGCCTTCGGGTTGTAAAGCACTTTCAG
CGAGGAGGAAGGCATTGTGGTTAATAGCCACAGTGATTGACGTTACTCGCAGAAGAAG
CACCGGCTAACTCCGTGCCAGCAGCCGCGGTAATACGGAGGGTGCAAGCGTTAATCGG
AATTACTGGGCGTAAAGCGCACGCAGGCGGTTTGTTAAGTCAGATGTGAAATCCCCGCG
CTTAACGTGGGAACTGCATTTGAAACTGGCAAGCTAGAGTCTTGTAGAGGGGGGTAGA
ATTCCAGGTGTAGCGGTGAAATGCGTAGAGATCTGGAGGAATACCGGTGGCGAAGGCG
GCCCCCTGGACAAAGACTGACGCTCAGGTGCGAAAGCGTGGGGAGCAAACAGGATTA
GATACCCTGGTAGTCCACGCTGTAAACGATGTCGACTTGGAGGTTGTGCCCTTGAGGCG

TGGCTTCCGGAGCTAACGCGTTAAGTCGACCGCCTGGGGAGTACGGCCGCAAGGTTAA
AACTCAAATGAATTGACGGGGGCCCGCACAAGCGGTGGAGCATGTGGTTTAATTCGATG
CAACGCGAAGAACCTTACCTACTCTTGACATCCACAGAACTTAGCAGAGATGCTTCGGT
GCCTTCGGGAACTGTGAGACAGGTGCTGCATGGCTGTCGTCAGCTCGTGTTGTGAAATG
TTGGGTTAAGTCCCGCAACGAGCGCAACCCTTATCCTTTGTTGCCAGCACGTAATGGTG
GGAACTCAAGGGAGACTGCCGGTGACAAACCGGAGGAAGGTGGGGATGACGTCAAGT
CATCATGGCCCTTACGAGTAGGGCTACACACGTGCTACAATGGCAGATACAAAGTGAAG
CGAACTCGCGAGAGCAAGCGGACCACATAAAGTCTGTCGTAGTCCGGATTGGAGTCTG
CAACTCGACTCCATGAAGTCGGAATCGCTAGTAATCGTAGATCAGAATGCTACGGTGAAT
ACGTTCCCGGGCCTTGTACACACCGCCCGTCACACCATGGGAGTGGGTTGCAAAAGAA
GTAGGTAGCTTAACCTTCGGGAGGGCGCTTACCACTTTGTGATTCATGACTGGGG
pattern 305
CGCTGGCGGCAGGCCTAACACATGCAAGTCGAGCGGCAGCGGAAAGTAGCTTGCTACT
TTGCCGGCGAGCGGCGGACGGGTGAGTAATGTCTGGGGATCTGCCTAATGGAGGGGGA
TAACTACTGGAAACGGTAGCTAATACCGCATGACCTCGAAAGAGCAAAGTGGGGGACC
TTCGGGCCTCACGCCATCGGATGAACCCAGATGGGATTAGCTAGTAGGTGGGGTAATGG
CTCACCTAGGCGACGATCCCTAGCTGGTCTGAGAGGATGACCAGCCACACTGGAACTG
AGACACGGTCCAGACTCCTACGGGAGGCAGCAGTGGGGAATATTGCACAATGGGCGCA
AGCCTGATGCAGCCATGCCGCGTGTGTGAAGAAGGCCTTCGGGTTGTAAAGCACTTTCA
GCGAGGAGGAAGGCATTGTGGTTAATAACCACAGTGATTGACGTTACTCGCAGAAGAA
GCACCGGCTAACTCCGTGCCAGCAGCCGCGGTAATACGGAGGGTGCAAGCGTTAATCG
GAATTACTGGGCGTAAAGCGCACGCAGGCGGTTTGTTAAGTCAGATGTGAAATCCCCGC
GCTTAACGTGGGAACTGCATTTGAAACTGGCAAGCTAGAGTCTTGTAGAGGGGGGTAG
AATTCCAGGTGTAGCGGTGAAATGCGTAGAGATCTGGAGGAATACCGGTGGCGAAGGC
GGCCCCCTGGACAAAGACTGACGCTCAGGTGCGAAAGCGTGGGGAGCAAACAGGATT
AGATACCCTGGTAGTCCACGCTGTAAACGATGTCGACTTGGAGGTTGTGCCCTTGAGGC
GTGGCTTCCGGAGCTAACGCGTTAAGTCGACCGCCTGGGGAGTACGGCCGCAAGGTTA
AAACTCAAATGAATTGACGGGGGCCCGCACAAGCGGTGGAGCATGTGGTTTAATTCGAT
GCAACGCGAAGAACCTTACCTACTCTTGACATCCACGGAATTTAGCAGAGATGCTTTAG
TGCCTTCGGGAACCGTGAGACAGGTGCTGCATGGCTGTCGTCAGCTCGTGTTGTGAAAT
GTTGGGTTAAGTCCCGCAACGAGCGCAACCCTTATCCTTTGTTGCCAGCACGTAATGGT
GGGAACTCAAGGGAGACTGCCGGTGACAAACCGGAGGAAGGTGGGGATGACGTCAAG
TCATCATGGCCCTTACGAGTAGGGCTACACACGTGCTACAATGGCAGATACAAAGTGAA
GCGAACTCGCGAGAGCAAGCGGACCACATAAAGTCTGTCGTAGTCCGGATTGGAGTCT
GCAACTCGACTCCATGAAGTCGGAATCGCTAGTAATCGTAGATCAGAATGCTACGGTGA
ATACGTTCCCGGGCCTTGTACACACCGCCCGTCACACCATGGGAGTGGGTTGCAAAAG
AAGTAGGTAGCTTAACCTTCGGGAGGGCGCTTACCACTTTGTGATTCATGACTGGGG
pattern 306
CGCTGGCGGCAGGCCTAACACATGCAAGTCGAGCGGCAGCGGAAAGTAGCTTGCTACT
TTGCCGGCGAGCGGCGGACGGGTGAGTAATGTCTGGGGATCTGCCTGATGGAGGGGGA
TAACTACTGGAAACGGTAGCTAATACCGCATGACCTCGAAAGAGCAAAGTGGGGGACC
TTCGGGCCTCACGCCATCGGATGAACCCAGATGGGATTAGCTAGTAGGTGGGGTAATGG
CTCACCTAGGCGACGATCCCTAGCTGGTCTGAGAGGATGACCAGCCACACTGGAACTG
AGACACGGTCCAGACTCCTACGGGAGGCAGCAGTGGGGAATATTGCACAATGGGCGCA

AGCCTGATGCAGCCATGCCGCGTGTGTGAAGAAGGCCTTCGGGTTGTAAAGCACTTTCA
GCGAGGAGGAAGGCATTGTGGTTAATAACCGCAGTGATTGACGTTACTCGCAGAAGAA
GCACCGGCTAACTCCGTGCCAGCAGCCGCGGTAATACGGAGGGTGCAAGCGTTAATCG
GAATTACTGGGCGTAAAGCGCACGCAGGCGGTTTGTTAAGTCAGATGTGAAATCCCCGC
GCTTAACGTGGGAACTGCATTTGAAACTGGCAAGCTAGAGTCTGGTAGAGGGGGGTAG
AATTCCAGGTGTAGCGGTGAAATGCGTAGAGATCTGGAGGAATACCGGTGGCGAAGGC
GGCCCCCTGGACAAAGACTGACGCTCAGGTGCGAAAGCGTGGGGAGCAAACAGGATT
AGATACCCTGGTAGTCCACGCTGTAAACGATGTCGACTTGGAGGTTGTGCCCTTGAGGC
GTGGCTTCCGGAGCTAACGCGTTAAGTCGACCGCCTGGGGAGTACGGCCGCAAGGTTA
AAACTCAAATGAATTGACGGGGGCCCGCACAAGCGGTGGAGCATGTGGTTTAATTCGAT
GCAACGCGAAGAACCTTACCTACTCTTGACATCCACAGAACTTAGCAGAGATGCTTCGG
TGCCTTCGGGAACTGTGAGACAGGTGCTGCATGGCTGTCGTCAGCTCGTGTTGTGAAAT
GTTGGGTTAAGTCCCGCAACGAGCGCAACCCTTATCCTTTGTTGCCAGCACGTAATGGT
GGGAACTCAAGGGAGACTGCCGGTGACAAACCGGAGGAAGGTGGGGATGACGTCAAG
TCATCATGGCCCTTACGAGTAGGGCTACACACGTGCTACAATGGCAGATACAAAGTGAA
GCGAACTCGCGAGAGCAAGCGGACCACATAAAGTCTGTCGTAGTCCGGATTGGAGTCT
GCAACTCGACTCCATGAAGTCGGAATCGCTAGTAATCGTAGATCAGAATGCTACGGTGA
ATACGTTCCCGGGCCTTGTACACACCGCCCGTCACACCATGGGAGTGGGTTGCAAAAG
AAGTAGGTAGCTTAACCTTCGGGAGGGCGCTTACCACTTTGTGATTCATGACTGGGG
pattern 307
CGCTGGCGGCAGGCCTAACACATGCAAGTCGAGCGGCAGCGGAAAGTAGCTTGCTACT
TTGCCGGCGAGCGGCGGACGGGTGAGTAATGTCTGGGGATCTGCCTGATGGAGGGGGA
TAACTACTGGAAACGGTAGCTAATACCGCATGACCTCGAAAGAGCAAAGTGGGGGACC
TTCGGGCCTCACGCCATCGGATGAACCCAGATGGGATTAGCTAGTAGGTGGGGTAATGG
CTCACCTAGGCGACGATCCTTAGCTGGTCTGAGAGGATGACCAGCCACACTGGAACTG
AGACACGGTCCAGACTCCTACGGGAGGCAGCAGTGGGGAATATTGCACAATGGGCGCA
AGCCTGATGCAGCCATGCCGCGTGTGTGAAGAAGGCCTTCGGGTTGTAAAGCACTTTCA
GCGAGGAGGAAGGCATTGTGGTTAATAACCGCAGTGATTGACGTTACTCGCAGAAGAA
GCACCGGCTAACTCCGTGCCAGCAGCCGCGGTAATACGGAGGGTGCAAGCGTTAATCG
GAATTACTGGGCGTAAAGCGCACGCAGGCGGTTTGTTAAGTCAGATGTGAAATCCCCGC
GCTTAACGTGGGAACTGCATTTGAAACTGGCAAGCTAGAGTCTTGTAGAGGGGGGTAG
AATTCCAGGTGTAGCGGTGAAATGCGTAGAGATCTGGAGGAATACCGGTGGCGAAGGC
GGCCCCCTGGACAAAGACTGACGCTCAGGTGCGAAAGCGTGGGGAGCAAACAGGATT
AGATACCCTGGTAGTCCACGCTGTAAACGATGTCGACTTGGAGGTTGTGCCCTTGAGGC
GTGGCTTCCGGAGCTAACGCGTTAAGTCGACCGCCTGGGGAGTACGGCCGCAAGGTTA
AAACTCAAATGAATTGACGGGGGCCCGCACAAGCGGTGGAGCATGTGGTTTAATTCGAT
GCAACGCGAAGAACCTTACCTACTCTTGACATCCACAGAACTTAGCAGAGATGCTTCGG
TGCCTTCGGGAACTGTGAGACAGGTGCTGCATGGCTGTCGTCAGCTCGTGTTGTGAAAT
GTTGGGTTAAGTCCCGCAACGAGCGCAACCCTTATCCTTTGTTGCCAGCACGTAATGGT
GGGAACTCAAGGGAGACTGCCGGTGACAAACCGGAGGAAGGTGGGGATGACGTCAAG
TCATCATGGCCCTTACGAGTAGGGCTACACACGTGCTACAATGGCAGATACAAAGTGAA
GCGAACTCGCGAGAGCAAGCGGACCACATAAAGTCTGTCGTAGTCCGGATTGGAGTCT
GCAACTCGACTCCATGAAGTCGGAATCGCTAGTAATCGTAGATCAGAATGCTACGGTGA
ATACGTTCCCGGGCCTTGTACACACCGCCCGTCACACCATGGGAGTAGGTTGCAAAAGA

AGTAGGTAGCTTAACCTTCGGGAGGGCGCTTACCACTTTGTGATTCATGACTGGGG
pattern 308
CGCTGGCGGCAGGCCTAACACATGCAAGTCGAGCGGCAGCGGGAGGTAGTTTACTACT
TTGCCGGCGAGCGGCGGACGGGTGAGTAATGTCTGGGAAACTGCCTGATGGAGGGGGA
TAACTACTGGAAACGGTAGCTAATACCGCATGACCTCGCAAGAGCAAAGTGGGGGACC
TTCGGGCCTCACGCCATCGGATGTGCCCAGATGGGATTAGCTAGTAGGTGGGGTAATGG
CTCACCTAGGCGACGATCCCTAGCTGGTCTGAGAGGATGACCAGCCACACTGGAACTG
AGACACGGTCCAGACTCCTACGGGAGGCAGCAGTGGGGAATATTGCACAATGGGCGCA
AGCCTGATGCAGCCATGCCGCGTGTGTGAAGAAGGCCTTCGGGTTGTAAAGCACTTTCA
GCGAGGAGGAAGGGTTCAGTGTTAATAGCACTGTTCATTGACGTTACTCGCAGAAGAA
GCACCGGCTAACTCCGTGCCAGCAGCCGCGGTAATACGGAGGGTGCAAGCGTTAATCG
GAATTACTGGGCGTAAAGCGCACGCAGGCGGTTTGTTAAGTCAGATGTGAAATCCCCGC
GCTTAACGTGGGAACTGCATTTGAAACTGGCAAGCTAGAGTCTTGTAGAGGGGGGTAG
AATTCCAGGTGTAGCGGTGAAATGCGTAGAGATCTGGAGGAATACCGGTGGCGAAGGC
GGCCCCCTGGACAAAGACTGACGCTCAGGTGCGAAAGCGTGGGGAGCAAACAGGATT
AGATACCCTGGTAGTCCACGCTGTAAACGATGTCGACTTGGAGGTTGTGCCCTTGAGGC
GTGGCTTCCGGAGCTAACGCGTTAAGTCGACCGCCTGGGGAGTACGGCCGCAAGGTTA
AAACTCAAATGAATTGACGGGGGCCCGCACAAGCGGTGGAGCATGTGGTTTAATTCGAT
GCAACGCGAAGAACCTTACCTACTCTTGACATCCACAGAACTTAGCAGAGATGCTTCGG
TGCCTTCGGGAACTGTGAGACAGGTGCTGCATGGCTGTCGTCAGCTCGTGTTGTGAAAT
GTTGGGTTAAGTCCCGCAACGAGCGCAACCCTTATCCTTTGTTGCCAGCACGTAATGGT
GGGAACTCAAGGGAGACTGCCGGTGACAAACCGGAGGAAGGTGGGGATGACGTCAAG
TCATCATGGCCCTTACGAGTAGGGCTACACACGTGCTACAATGGCAGATACAAAGTGAA
GCGAACTCGCGAGAGCAAGCGGACCACATAAAGTCTGTCGTAGTCCGGATTGGAGTCT
GCAACTCGACTCCATGAAGTCGGAATCGCTAGTAATCGTAGATCAGAATGCTACGGTGA
ATACGTTCCCGGGCCTTGTACACACCGCCCGTCACACCATGGGAGTGGGTTGCAAAAG
AAGTAGGTAGCTTAACCTTCGGGAGGGCGCTTACCACTTTGTGATTCATGACTGGGG
pattern 309
CGCTGGCGGCAGGCCTAACACATGCAAGTCGAGCGGCAGCGGGAAGTAGTTTACTACT
TTGCCGGCGAGCGGCGGACGGGTGAGTAATGTCTGGGAAACTGCCTGATGGAGGGGGA
TAACTACTGGAAACGGTAGCTAATACCGCATGACCTCGCAAGAGCAAAGTGGGGGACC
TTAGGGCCTCACGCCATCGGATGTGCCCAGATGGGATTAGCTAGTAGGTGGGGTAATGG
CTCACCTAGGCGACGATCCCTAGCTGGTCTGAGAGGATGACCAGCCACACTGGAACTG
AGACACGGTCCAGACTCCTACGGGAGGCAGCAGTGGGGAATATTGCACAATGGGCGCA
AGCCTGATGCAGCCATGCCGCGTGTGTGAAGAAGGCCTTCGGGTTGTAAAGCACTTTCA
GCGAGGAGGAAGGGTTCAGTATTAATAGCACTGTTCATTGACGTTACTCGCAGAAGAAG
CACCGGCTAACTCCGTGCCAGCAGCCGCGGTAATACGGAGGGTGCAAGCGTTAATCGG
AATTACTGGGCGTAAAGCGCACGCAGGCGGTTTGTTAAGTCAGATGTGAAATCCCCGCG
CTTAACGTGGGAACTGCATTTGAAACTGGCAAGCTAGAGTCTTGTAGAGGGGGGTAGA
ATTCCAGGTGTAGCGGTGAAATGCGTAGAGATCTGGAGGAATACCGGTGGCGAAGGCG
GCCCCCTGGACAAAGACTGACGCTCAGGTGCGAAAGCGTGGGGAGCAAACAGGATTA
GATACCCTGGTAGTCCACGCTGTAAACGATGTCGACTTGGAGGTTGTGCCCTTGAGGCG
TGGCTTCCGGAGCTAACGCGTTAAGTCGACCGCCTGGGGAGTACGGCCGCAAGGTTAA
AACTCAAATGAATTGACGGGGGCCCGCACAAGCGGTGGAGCATGTGGTTTAATTCGATG

CAACGCGAAGAACCTTACCTACTCTTGACATCCACAGAACTTAGCAGAGATGCTTCGGT
GCCTTCGGGAACTGTGAGACAGGTGCTGCATGGCTGTCGTCAGCTCGTGTTGTGAAATG
TTGGGTTAAGTCCCGCAACGAGCGCAACCCTTATCCTTTGTTGCCAGCACGTAATGGTG
GGAACTCAAGGGAGACTGCCGGTGACAAACCGGAGGAAGGTGGGGATGACGTCAAGT
CATCATGGCCCTTACGAGTAGGGCTACACACGTGCTACAATGGCAGATACAAAGTGAAG
CGAACTCGCGAGAGCAAGCGGACCACATAAAGTCTGTCGTAGTCCGGATTGGAGTCTG
CAACTCGACTCCATGAAGTCGGAATCGCTAGTAATCGTAGATCAGAATGCTACGGTGAAT
ACGTTCCCGGGCCTTGTACACACCGCCCGTCACACCATGGGAGTGGGTTGCAAAAGAA
GTAGGTAGCTTAACCTTCGGGAGGGCGCTTACCACTTTGTGATTCATGACTGGGG
pattern 310
CGCTGGCGGCAGGCCTAACACATGCAAGTCGAGCGGCAGCGGGAAGTAGTTTACTACT
TTGCCGGCGAGCGGCGGACGGGTGAGTAATGTCTGGGAAACTGCCTGATGGAGGGGGA
TAACTACTGGAAACGGTAGCTAATACCGCATGACCTCGCAAGAGCAAAGTGGGGGACC
TTAGGGCCTCACGCCATCGGATGTGCCCAGATGGGATTAGCTAGTAGGTGGGGTAATGG
CTCACCTAGGCGACGATCCCTAGCTGGTCTGAGAGGATGACCAGCCACACTGGAACTG
AGACACGGTCCAGACTCCTACGGGAGGCAGCAGTGGGGAATATTGCACAATGGGCGCA
AGCCTGATGCAGCCATGCCGCGTGTGTGAAGAAGGCCTTCGGGTTGTAAAGCACTTTCA
GCGAGGAGGAAGGCAATCGTGTTAATAGCACGGTTGATTGACGTTACTCGCAGAAGAA
GCACCGGCTAACTCCGTGCCAGCAGCCGCGGTAATACGGAGGGTGCAAGCGTTAATCG
GAATTACTGGGCGTAAAGCGCACGCAGGCGGTTTGTTAAGTCAGATGTGAAATCCCCGC
GCTTAACGTGGGAACTGCATTTGAAACTGGCAAGCTAGAGTCTTGTAGAGGGGGGTAG
AATTCCAGGTGTAGCGGTGAAATGCGTAGAGATCTGGAGGAATACCGGTGGCGAAGGC
GGCCCCCTGGACAAGGACTGACGCTCAGGTGCGAAAGCGTGGGGAGCAAACAGGATT
AGATACCCTGGTAGTCCACGCTGTAAACGATGTCGACTTGGAGGTTGTGCCCTTGAGGC
GTGGCTTCCGGAGCTAACGCGTTAAGTCGACCGCCTGGGGAGTACGGCCGCAAGGTTA
AAACTCAAATGAATTGACGGGGGCCCGCACAAGCGGTGGAGCATGTGGTTTAATTCGAT
GCAACGCGAAGAACCTTACCTACTCTTGACATCCACAGAACTTAGCAGAGATGCTTCGG
TGCCTTCGGGAACTGTGAGACAGGTGCTGCATGGCTGTCGTCAGCTCGTGTTGTGAAAT
GTTGGGTTAAGTCCCGCAACGAGCGCAACCCTTATCCTTTGTTGCCAGCACGTAATGGT
GGGAACTCAAGGGAGACTGCCGGTGACAAACCGGAGGAAGGTGGGGATGACGTCAAG
TCATCATGGCCCTTACGAGTAGGGCTACACACGTGCTACAATGGCAGATACAAAGTGAA
GCGAACTCGCGAGAGCAAGCGGACCACATAAAGTCTGTCGTAGTCCGGATTGGAGTCT
GCAACTCGACTCCATGAAGTCGGAATCGCTAGTAATCGTAGATCAGAATGCTACGGTGA
ATACGTTCCCGGGCCTTGTACACACCGCCCGTCACACCATGGGAGTGGGTTGCAAAAG
AAGTAGGTAGCTTAACCTTCGGGAGGGCGCTTACCACTTTGTGATTCATGACTGGGG
pattern 311
CGCTGGCGGCAGGCCTAACACATGCAAGTCGAGCGGCAGCGGGAAGTAGTTTACTACT
TTGCCGGCGAGCGGCGGACGGGTGAGTAATGTCTGGGGATCTGCCTGATGGAGGGGGA
TAACTACTGGAAACGGTAGCTAATACCGCAGGACCTCGCAAGAGCAAAGTGGGGGACC
TTAGGGCCTCACGCCATCGGATGAACCCAGATGGGATTAGCTAGTAGGTGGGGTAATGG
CTCACCTAGGCGACGATCCCTAGCTGGTCTGAGAGGATGACCAGCCACACTGGAACTG
AGACACGGTCCAGACTCCTACGGGAGGCAGCAGTGGGGAATATTGCACAATGGGCGCA
AGCCTGATGCAGCCATGCCGCGTGTGTGAAGAAGGCCTTCGGGTTGTAAAGCACTTTCA
GCGAGGAGGAAGGGGTTGAGTTTAATACGCTCAATCATTGACGTTACTCGCAGAAGAA

GCACCGGCTAACTCCGTGCCAGCAGCCGCGGTAATACGGAGGGTGCAAGCGTTAATCG
GAATTACTGGGCGTAAAGCGCACGCAGGCGGTTTGTTAAGTCAGATGTGAAATCCCCGC
GCTTAACGTGGGAACTGCATTTGAAACTGGCAAGCTAGAGTCTTGTAGAGGGGGGTAG
AATTCCAGGTGTAGCGGTGAAATGCGTAGAGATCTGGAGGAATACCGGTGGCGAAGGC
GGCCCCCTGGACAAAGACTGACGCTCAGGTGCGAAAGCGTGGGGAGCAAACAGGATT
AGATACCCTGGTAGTCCACGCTGTAAACGATGTCGACTTGGAGGTTGTGCCCTTGAGGC
GTGGCTTCCGGAGCTAACGCGTTAAGTCGACCGCCTGGGGAGTACGGCCGCAAGGTTA
AAACTCAAATGAATTGACGGGGGCCCGCACAAGCGGTGGAGCATGTGGTTTAATTCGAT
GCAACGCGAAGAACCTTACCTACTCTTGACATCCACAGAATTTGGCAGAGATGCTAAAG
TGCCTTCGGGAACTGTGAGACAGGTGCTGCATGGCTGTCGTCAGCTCGTGTTGTGAAAT
GTTGGGTTAAGTCCCGCAACGAGCGCAACCCTTATCCTTTGTTGCCAGCACGTAATGGT
GGGAACTCAAGGGAGACTGCCGGTGACAAACCGGAGGAAGGTGGGGATGACGTCAAG
TCATCATGGCCCTTACGAGTAGGGCTACACACGTGCTACAATGGCAGATACAAAGTGAA
GCGAACTCGCGAGAGCCAGCGGACCACATAAAGTCTGTCGTAGTCCGGATTGGAGTCT
GCAACTCGACTCCATGAAGTCGGAATCGCTAGTAATCGTAGATCAGAATGCTACGGTGA
ATACGTTCCCGGGCCTTGTACACACCGCCCGTCACACCATGGGAGTGGGTTGCAAAAG
AAGTAGGTAGCTTAACCTTCGGGAGGGCGCTTACCACTTTGTGATTCATGACTGGGG
pattern 312
CGCTGGCGGCAGGCCTAACACATGCAAGTCGAGCGGCAGCGGGAAGTAGTTTACTACT
TTGCCGGCGAGCGGCGGACGGGTGAGTAATGTCTGGGAAACTGCCTGATGGAGGGGGA
TAACTACTGGAAACGGTAGCTAATACCGCATGACCTCGCAAGAGCAAAGTGGGGGACC
TTCGGGCCTCACGCCATCGGATGTGCCCAGATGGGATTAGCTAGTAGGTGGGGTAATGG
CTCACCTAGGCGACGATCCCTAGCTGGTCTGAGAGGATGACCAGCCACACTGGAACTG
AGACACGGTCCAGACTCCTACGGGAGGCAGCAGTGGGGAATATTGCACAATGGGCGCA
AGCCTGATGCAGCCATGCCGCGTGTGTGAAGAAGGCCTTCGGGTTGTAAAGCACTTTCA
GCGAGGAGGAAGGGTTCAGTGTTAATAGCACTGTACATTGACGTTACTCGCAGAAGAA
GCACCGGCTAACTCCGTGCCAGCAGCCGCGGTAATACGGAGGGTGCAAGCGTTAATCG
GAATTACTGGGCGTAAAGCGCACGCAGGCGGTTTGTTAAGTCAGATGTGAAATCCCCGC
GCTTAACGTGGGAACTGCATTTGAAACTGGCAAGCTAGAGTCTTGTAGAGGGGGGTAG
AATTCCAGGTGTAGCGGTGAAATGCGTAGAGATCTGGAGGAATACCGGTGGCGAAGGC
GGCCCCCTGGACAAAGACTGACGCTCAGGTGCGAAAGCGTGGGGAGCAAACAGGATT
AGATACCCTGGTAGTCCACGCTGTAAACGATGTCGACTTGGAGGTTGTGCCCTTGAGGC
GTGGCTTCCGGAGCTAACGCGTTAAGTCGACCGCCTGGGGAGTACGGCCGCAAGGTTA
AAACTCAAATGAATTGACGGGGGCCCGCACAAGCGGTGGAGCATGTGGTTTAATTCGAT
GCAACGCGAAGAACCTTACCTACTCTTGACATCCACAGAACTTAGCAGAGATGCTTAGG
TGCCTTCGGGAACTGTGAGACAGGTGCTGCATGGCTGTCGTCAGCTCGTGTTGTGAAAT
GTTGGGTTAAGTCCCGCAACGAGCGCAACCCTTATCCTTTGTTGCCAGCACGTAATGGT
GGGAACTCAAGGGAGACTGCCGGTGACAAACCGGAGGAAGGTGGGGATGACGTCAAG
TCATCATGGCCCTTACGAGTAGGGCTACACACGTGCTACAATGGCAGATACAAAGTGAA
GCGAACTCGCGAGAGCAAGCGGACCACATAAAGTCTGTCGTAGTCCGGATTGGAGTCT
GCAACTCGACTCCATGAAGTCGGAATCGCTAGTAATCGTAGATCAGAATGCTACGGTGA
ATACGTTCCCGGGCCTTGTACACACCGCCCGTCACACCATGGGAGTGGGTTGCAAAAG
AAGTAGGTAGCTTAACCTTCGGGAGGGCGCTTACCACTTTGTGATTCATGACTGGGG
pattern 313

CGCTGGCGGCAGGCCTAACACATGCAAGTCGAGCGGCAGCGGGAAGTAGTTTACTACT
TTGCCGGCGAGCGGCGGACGGGTGAGTAATGTCTGGGAAACTGCCTGATGGAGGGGGA
TAACTACTGGAAACGGTAGCTAATACCGCATGACCTCGCAAGAGCAAAGTGGGGGACC
TTCGGGCCTCACGCCATCGGATGTGCCCAGATGGGATTAGCTAGTAGGTGGGGTAATGG
CTCACCTAGGCGACGATCCCTAGCTGGTCTGAGAGGATGACCAGCCACACTGGAACTG
AGACACGGTCCAGACTCCTACGGGAGGCAGCAGTGGGGAATATTGCACAATGGGCGCA
AGCCTGATGCAGCCATGCCGCGTGTGTGAAGAAGGCCTTCGGGTTGTAAAGCACTTTCA
GCGAGGAGGAAGGGTTCAGTGTTAATAGCACTGTACATTGACGTTACTCGCAGAAGAA
GCACCGGCTAACTCCGTGCCAGCAGCCGCGGTAATACGGAGGGTGCAAGCGTTAATCG
GAATTACTGGGCGTAAAGCGCACGCAGGCGGTTTGTTAAGTCAGATGTGAAATCCCCGC
GCTTAACGTGGGAACTGCATTTGAAACTGGCAAGCTAGAGTCTTGTAGAGGGGGGTAG
AATTCCAGGTGTAGCGGTGAAATGCGTAGAGATCTGGAGGAATACCGGTGGCGAAGGC
GGCCCCCTGGACAAAGACTGACGCTCAGGTGCGAAAGCGTGGGGAGCAAACAGGATT
AGATACCCTGGTAGTCCACGCTGTAAACGATGTCGACTTGGAGGTTGTGCCCTTGAGGC
GTGGCTTCCGGAGCTAACGCGTTAAGTCGACCGCCTGGGGAGTACGGCCGCAAGGTTA
AAACTCAAATGAATTGACGGGGGCCCGCACAAGCGGTGGAGCATGTGGTTTAATTCGAT
GCAACGCGAAGAACCTTACCTACTCTTGACATCCACAGAACTTAGCAGAGATGCTTAGG
TGCCTTCGGGAACTGTGAGACAGGTGCTGCATGGCTGTCGTCAGCTCGTGTTGTGAAAT
GTTGGGTTAAGTCCCGCAACGAGCGCAACCCTTATCCTTTGTTGCCAGCACGTAATGGT
GGGAACTCAAGGGAGACTGCCGGTGACAAACCGGAGGAAGGTGGGGATGACGTCAAG
TCATCATGGCCCTTACGAGTAGGGCTACACACGTGCTACAATGGCAGATACAAAGTGAA
GCAAACTCGCGAGAGCAAGCGGACCACATAAAGTCTGTCGTAGTCCGGATTGGAGTCT
GCAACTCGACTCCATGAAGTCGGAATCGCTAGTAATCGTAGATCAGAATGCTACGGTGA
ATACGTTCCCGGGCCTTGTACACACCGCCCGTCACACCATGGGAGTGGGTTGCAAAAG
AAGTAGGTAGCTTAACCTTCGGGAGGGCGCTTACCACTTTGTGATTCATGACTGGGG
pattern 314
CGCTGGCGGCAGGCCTAACACATGCAAGTCGAGCGGCAGCGGGAAGTAGTTTACTACT
TTGCCGGCGAGCGGCGGACGGGTGAGTAATGTCTGGGAAACTGCCTGATGGAGGGGGA
TAACTACTGGAAACGGTAGCTAATACCGCATGACCTCGCAAGAGCAAAGTGGGGGACC
TTCGGGCCTCACGCCATCGGATGTGCCCAGATGGGATTAGCTAGTAGGTGGGGTAATGG
CTCACCTAGGCGACGATCCCTAGCTGGTCTGAGAGGATGACCAGCCACACTGGAACTG
AGACACGGTCCAGACTCCTACGGGAGGCAGCAGTGGGGAATATTGCACAATGGGCGCA
AGCCTGATGCAGCCATGCCGCGTGTGTGAAGAAGGCCTTCGGGTTGTAAAGCACTTTCA
GCGAGGAGGAAGGCCAATAGCTTAATACGCTGTTGGATTGACGTTACTCGCAGAAGAA
GCACCGGCTAACTCCGTGCCAGCAGCCGCGGTAATACGGAGGGTGCAAGCGTTAATCG
GAATTACTGGGCGTAAAGCGCACGCAGGCGGTTTGTTAAGTCAGATGTGAAATCCCCGC
GCTTAACGTGGGAACTGCATTTGAAACTGGCAAGCTAGAGTCTTGTAGAGGGGGGTAG
AATTCCAGGTGTAGCGGTGAAATGCGTAGAGATCTGGAGGAATACCGGTGGCGAAGGC
GGCCCCCTGGACAAAGACTGACGCTCAGGTGCGAAAGCGTGGGGAGCAAACAGGATT
AGATACCCTGGTAGTCCACGCTGTAAACGATGTCGACTTGGAGGTTGTGCCCTTGAGGC
GTGGCTTCCGGAGCTAACGCGTTAAGTCGACCGCCTGGGGAGTACGGCCGCAAGGTTA
AAACTCAAATGAATTGACGGGGGCCCGCACAAGCGGTGGAGCATGTGGTTTAATTCGAT
GCAACGCGAAGAACCTTACCTACTCTTGACATCCACAGAACTTAGCAGAGATGCTTAGG
TGCCTTCGGGAACTGTGAGACAGGTGCTGCATGGCTGTCGTCAGCTCGTGTTGTGAAAT

GTTGGGTTAAGTCCCGCAACGAGCGCAACCCTTATCCTTTGTTGCCAGCACGTAATGGT
GGGAACTCAAGGGAGACTGCCGGTGACAAACCGGAGGAAGGTGGGGATGACGTCAAG
TCATCATGGCCCTTACGAGTAGGGCTACACACGTGCTACAATGGCAGATACAAAGTGAA
GCGAACTCGCGAGAGCAAGCGGACCACATAAAGTCTGTCGTAGTCCGGATTGGAGTCT
GCAACTCGACTCCATGAAGTCGGAATCGCTAGTAATCGTAGATCAGAATGCTACGGTGA
ATACGTTCCCGGGCCTTGTACACACCGCCCGTCACACCATGGGAGTGGGTTGCAAAAG
AAGTAGGTAGCTTAACCTTCGGGAGGGCGCTTACCACTTTGTGATTCATGACTGGGG

pattern 315

CGCTGGCGGCAGGCCTAACACATGCAAGTCGAGCGGCAGCGGGAAGTAGCTTGCTACT
TTGCCGGCGAGCGGCGGACGGGTGAGTAATGTCTGGGAAACTGCCTGATGGAGGGGGA
TAACTACTGGAAACGGTAGCTAATACCGCATGACGTCTTCGGACCAAAGTGGGGGACCT
TCGGGCCTCACGCCATCGGATGTGCCCAGATGGGATTAGCTAGTAGGTGGGGTAACGGC
TCACCTAGGCGACGATCCCTAGCTGGTCTGAGAGGATGACCAGCCACACTGGAACTGA
GACACGGTCCAGACTCCTACGGGAGGCAGCAGTGGGGAATATTGCACAATGGGCGCAA
GCCTGATGCAGCCATGCCGCGTGTGTGAAGAAGGCCTTCGGGTTGTAAAGCACTTTCAG
CGAGGAGGAAGGCATAAAGGTTAATAACCTTTGTGATTGACGTTACTCGCAGAAGAAG
CACCGGCTAACTCCGTGCCAGCAGCCGCGGTAATACGGAGGGTGCAAGCGTTAATCGG
AATTACTGGGCGTAAAGCGCACGCAGGCGGTTTGTTAAGTCAGATGTGAAATCCCCGCG
CTTAACGTGGGAACTGCATTTGAAACTGGCAAGCTAGAGTCTTGTAGAGGGGGGTAGA
ATTCCAGGTGTAGCGGTGAAATGCGTAGAGATCTGGAGGAATACCGGTGGCGAAGGCG
GCCCCCTGGACAAAGACTGACGCTCAGGTGCGAAAGCGTGGGGAGCAAACAGGATTA
GATACCCTGGTAGTCCACGCTGTAAACGATGTCGACTTGGAGGTTGTGCCCTTGAGGCG
TGGCTTCCGGAGCTAACGCGTTAAGTCGACCGCCTGGGGAGTACGGCCGCAAGGTTAA
AACTCAAATGAATTGACGGGGGCCCGCACAAGCGGTGGAGCATGTGGTTTAATTCGATG
CAACGCGAAGAACCTTACCTACTCTTGACATCCACAGAACTTAGCAGAGATGCTTCGGT
GCCTTCGGGAACTGTGAGACAGGTGCTGCATGGCTGTCGTCAGCTCGTGTTGTGAAATG
TTGGGTTAAGTCCCGCAACGAGCGCAACCCTTATCCTTTGTTGCCAGCACGTGATGGTG
GGAACTCAAAGGAGACTGCCGGTGATAAACCGGAGGAAGGTGGGGATGACGTCAAGT
CATCATGGCCCTTACGAGTAGGGCTACACACGTGCTACAATGGCAGATACAAAGTGAAG
CGAACTCGCGAGAGCAAGCGGACCACATAAAGTCTGTCGTAGTCCGGATTGGAGTCTG
CAACTCGACTCCATGAAGTCGGAATCGCTAGTAATCGTAGATCAGAATGCTACGGTGAAT
ACGTTCCCGGGCCTTGTACACACCGCCCGTCACACCATGGGAGTGGGTTGCAAAAGAA
GTAGGTAGCTTAACCTTCGGGAGGGCGCTTACCACTTTGTGATTCATGACTGGGG

pattern 316

CGCTGGCGGCAGGCCTAACACATGCAAGTCGAGCGGCAGCGGGGAGTAGCTTGCTACT
TTGCCGGCGAGCGGCGGACGGGTGAGTAATGTCTGGGAAACTGCCTGATGGAGGGGGA
TAACTACTGGAAACGGTAGCTAATACCGCATGACGTCTTCGGACCAAAGTGGGGGACCT
TCGGGCCTCACGCCATCGGATGTGCCCAGATGGGATTAGCTAGTAGGTGGGGTAACGGC
TCACCTAGGCGACGATCCCTAGCTGGTCTGAGAGGATGACCAGCCACACTGGAACTGA
GACACGGTCCAGACTCCTACGGGAGGCAGCAGTGGGGAATATTGCACAATGGGCGCAA
GCCTGATGCAGCCATGCCGCGTGTGTGAAGAAGGCCTTCGGGTTGTAAAGCACTTTCAG
CGAGGAGGAAGGCATAAAGGTTAATAACCTTTGTGATTGACGTTACTCGCAGAAGAAG
CACCGGCTAACTCCGTGCCAGCAGCCGCGGTAATACGGAGGGTGCAAGCGTTAATCGG
AATTACTGGGCGTAAAGCGCACGCAGGCGGTTTGTTAAGTCAGATGTGAAATCCCCGCG

CTTAACGTGGGAACTGCATTTGAAACTGGCAAGCTAGAGTCTTGTAGAGGGGGGTAGA
ATTCCAGGTGTAGCGGTGAAATGCGTAGAGATCTGGAGGAATACCGGTGGCGAAGGCG
GCCCCCTGGACAAAGACTGACGCTCAGGTGCGAAAGCGTGGGGAGCAAACAGGATTA
GATACCCTGGTAGTCCACGCTGTAAACGATGTCGACTTGGAGGTTGTGCCCTTGAGGCG
TGGCTTCCGGAGCTAACGCGTTAAGTCGACCGCCTGGGGAGTACGGCCGCAAGGTTAA
AACTCAAATGAATTGACGGGGGCCCGCACAAGCGGTGGAGCATGTGGTTTAATTCGATG
CAACGCGAAGAACCTTACCTACTCTTGACATCCACAGAACTTAGCAGAGATGCTTCGGT
GCCTTCGGGAACTGTGAGACAGGTGCTGCATGGCTGTCGTCAGCTCGTGTTGTGAAATG
TTGGGTTAAGTCCCGCAACGAGCGCAACCCTTATCCTTTGTTGCCAGCACGTGATGGTG
GGAACTCAAAGGAGACTGCCGGTGATAAACCGGAGGAAGGTGGGGATGACGTCAAGT
CATCATGGCCCTTACGAGTAGGGCTACACACGTGCTACAATGGCAGATACAAAGTGAAG
CGAACTCGCGAGAGCAAGCGGACCACATAAAGTCTGTCGTAGTCCGGATTGGAGTCTG
CAACTCGACTCCATGAAGTCGGAATCGCTAGTAATCGTAGATCAGAATGCTACGGTGAAT
ACGTTCCCGGGCCTTGTACACACCGCCCGTCACACCATGGGAGTGGGTTGCAAAAGAA
GTAGGTAGCTTAACCTTCGGGAGGGCGCTTACCACTTTGTGATTCATGACTGGGG
pattern 317
CGCTGGCGGCAGGCCTAACACATGCAAGTCGAGCGGCAGCGGGAAGTAGCTTGCTACT
TTGCCGGCGAGCGGCGGACGGGTGAGTAATGTCTGGGAAACTGCCTGATGGAGGGGGA
TAACTACTGGAAACGGTAGCTAATACCGCATGACGTCTTCGGACCAAAGTGGGGGACCT
TCGGGCCTCACGCCATCGGATGTGCCCAGATGGGATTAGCTAGTAGGTGGGGTAATGGC
TCACCTAGGCGACGATCCCTAGCTGGTCTGAGAGGATGACCAGCCACACTGGAACTGA
GACACGGTCCAGACTCCTACGGGAGGCAGCAGTGGGGAATATTGCACAATGGGCGCAA
GCCTGATGCAGCCATGCCGCGTGTGTGAAGAAGGCCTTCGGGTTGTAAAGCACTTTCAG
CGAGGAGGAAGGCATAAAGGTTAATAACCTTTGTGATTGACGTTACTCGCAGAAGAAG
CACCGGCTAACTCCGTGCCAGCAGCCGCGGTAATACGGAGGGTGCAAGCGTTAATCGG
AATTACTGGGCGTAAAGCGCACGCAGGCGGTTTGTTAAGTCAGATGTGAAATCCCCGCG
CTTAACGTGGGAACTGCATTTGAAACTGGCAAGCTAGAGTCTTGTAGAGGGGGGTAGA
ATTCCAGGTGTAGCGGTGAAATGCGTAGAGATCTGGAGGAATACCGGTGGCGAAGGCG
GCCCCCTGGACAAAGACTGACGCTCAGGTGCGAAAGCGTGGGGAGCAAACAGGATTA
GATACCCTGGTAGTCCACGCTGTAAACGATGTCGACTTGGAGGTTGTGCCCTTGAGGCG
TGGCTTCCGGAGCTAACGCGTTAAGTCGACCGCCTGGGGAGTACGGCCGCAAGGTTAA
AACTCAAATGAATTGACGGGGGCCCGCACAAGCGGTGGAGCATGTGGTTTAATTCGATG
CAACGCGAAGAACCTTACCTACTCTTGACATCCACAGAACTTAGCAGAGATGCTTCGGT
GCCTTCGGGAACTGTGAGACAGGTGCTGCATGGCTGTCGTCAGCTCGTGTTGTGAAATG
TTGGGTTAAGTCCCGCAACGAGCGCAACCCTTATCCTTTGTTGCCAGCACGTGATGGTG
GGAACTCAAAGGAGACTGCCGGTGATAAACCGGAGGAAGGTGGGGATGACGTCAAGT
CATCATGGCCCTTACGAGTAGGGCTACACACGTGCTACAATGGCAGATACAAAGTGAAG
CGAACTCGCGAGAGCAAGCGGACCACATAAAGTCTGTCGTAGTCCGGATTGGAGTCTG
CAACTCGACTCCATGAAGTCGGAATCGCTAGTAATCGTAGATCAGAATGCTACGGTGAAT
ACGTTCCCGGGCCTTGTACACACCGCCCGTCACACCATGGGAGTGGGTTGCAAAAGAA
GTAGGTAGCTTAACCTTCGGGAGGGCGCTTACCACTTTGTGATTCATGACTGGGG
pattern 318
CGCTGGCGGCAGGCCTAACACATGCAAGTCGAGCGGCAGCGGGGAGTAGCTTGCTACT
TTGCCGGCGAGCGGCGGACGGGTGAGTAATGTCTGGGAAACTGCCTGATGGAGGGGGA

TAACTACTGGAAACGGTAGCTAATACCGCATGACGTCTTCGGACCAAAGTGGGGGACCT
TCGGGCCTCACGCCATCGGATGTGCCCAGATGGGATTAGCTAGTAGGTGGGGTAACGGC
TCACCTAGGCGACGATCCCTAGCTGGTCTGAGAGGATGACCAGCCACACTGGAACTGA
GACACGGTCCAGACTCCTACGGGAGGCAGCAGTGGGGAATATTGCACAATGGGCGCAA
GCCTGATGCAGCCATGCCGCGTGTGTGAAGAAGGCCTTCGGGTTGTAAAGCACTTTCAG
CGAGGAGGAAGGCATAAAGGTTAATAACCTTTGTGATTGACGTTACTCGCAGAAGAAG
CACCGGCTAACTCCGTGCCAGCAGCCGCGGTAATACGGAGGGTGCAAGCGTTAATCGG
AATTACTGGGCGTAAAGCGCACGCAGGCGGTTTGTTAAGTCAGATGTGAAATCCCCGCG
CTTAACGTGGGAACTGCATTTGAAACTGGCAAGCTAGAGTCTTGTAGAGGGGGGTAGA
ATTCCAGGTGTAGCGGTGAAATGCGTAGAGATCTGGAGGAATACCGGTGGCGAAGGCG
GCCCCCTGGACAAAGACTGACGCTCAGGTGCGAAAGCGTGGGGAGCAAACAGGATTA
GATACCCTGGTAGTCCACGCTGTAAACGATGTCGACTTGGAGGTTGTGCCCTTGAGGCG
TGGCTTCCGGAGCTAACGCGTTAAGTCGACCGCCTGGGGAGTACGGCCGCAAGGTTAA
AACTCAAATGAATTGACGGGGGCCCGCACAAGCGGTGGAGCATGTGGTTTAATTCGATG
CAACGCGAAGAACCTTACCTACTCTTGACATCCACAGAACTTAGCAGAGATGCTTCGGT
GCCTTCGGGAACTGTGAGACAGGTGCTGCATGGCTGTCGTCAGCTCGTGTTGTGAAATG
TTGGGTTAAGTCCCGCAACGAGCGCAACCCTTATCCTTTGTTGCCAGCACGTGATGGTG
GGAACTCAAAGGAGACTGCCGGTGATAAACCGGAGGAAGGTGGGGATGACGTCAAGT
CATCATGGCCCTTACGAGTAGGGCTACACACGTGCTACAATGGCAGATACAAAGTGAAG
CAAACTCGCGAGAGCAAGCGGACCACATAAAGTCTGTCGTAGTCCGGATTGGAGTCTG
CAACTCGACTCCATGAAGTCGGAATCGCTAGTAATCGTAGATCAGAATGCTACGGTGAAT
ACGTTCCCGGGCCTTGTACACACCGCCCGTCACACCATGGGAGTGGGTTGCAAAAGAA
GTAGGTAGCTTAACCTTCGGGAGGGCGCTTACCACTTTGTGATTCATGACTGGGG
pattern 319
CGCTGGCGGCAGGCCTAACACATGCAAGTCGAGCGGCAGCGGGAAGTAGTTTACTACT
TTGCCGGCGAGCGGCGGACGGGTGAGTAATGTCTGGGAAACTGCCTGATGGAGGGGGA
TAACTACTGGAAACGGTAGCTAATACCGCATAACGTCTTCGGACCAAAGTGGGGGACCT
TCGGGCCTCACGCCATCGGATGTGCCCAGATGGGATTAGCTAGTAGGTGGGGTAATGGC
TCACCTAGGCGACGATCCCTAGCTGGTCTGAGAGGATGACCAGCCACACTGGAACTGA
GACACGGTCCAGACTCCTACGGGAGGCAGCAGTGGGGAATATTGCACAATGGGCGCAA
GCCTGATGCAGCCATGCCGCGTGTGTGAAGAAGGCCTTCGGGTTGTAAAGCACTTTCAG
CGAGGAGGAAGGCATAAAGGTTAATAACCTTTGTGATTGACGTTACTCGCAGAAGAAG
CACCGGCTAACTCCGTGCCAGCAGCCGCGGTAATACGGAGGGTGCAAGCGTTAATCGG
AATTACTGGGCGTAAAGCGCACGCAGGCGGTTTGTTAAGTCAGATGTGAAATCCCCGCG
CTTAACGTGGGAACTGCATTTGAAACTGGCAAGCTAGAGTCTTGTAGAGGGGGGTAGA
ATTCCAGGTGTAGCGGTGAAATGCGTAGAGATCTGGAGGAATACCGGTGGCGAAGGCG
GCCCCCTGGACAAAGACTGACGCTCAGGTGCGAAAGCGTGGGGAGCAAACAGGATTA
GATACCCTGGTAGTCCACGCTGTAAACGATGTCGACTTGGAGGTTGTGCCCTTGAGGCG
TGGCTTCCGGAGCTAACGCGTTAAGTCGACCGCCTGGGGAGTACGGCCGCAAGGTTAA
AACTCAAATGAATTGACGGGGGCCCGCACAAGCGGTGGAGCATGTGGTTTAATTCGATG
CAACGCGAAGAACCTTACCTACTCTTGACATCCACGGAATTTAGCAGAGATGCTTTAGT
GCCTTCGGGAACCGTGAGACAGGTGCTGCATGGCTGTCGTCAGCTCGTGTTGTGAAATG
TTGGGTTAAGTCCAGCAACGAGCGCAACCCTTATCCTTTGTTGCCAGCACGTCATGGTG
GGAACTCAAAGGAGACTGCCGGTGATAAACCGGAGGAAGGTGGGGATGACGTCAAGT

CATCATGGCCCTTACGAGTAGGGCTACACACGTGCTACAATGGCAGATACAAAGTGAAG
CGAACTCGCGAGAGCAAGCGGACCACATAAAGTCTGTCGTAGTCCGGATTGGAGTCTG
CAACTCGACTCCATGAAGTCGGAATCGCTAGTAATCGTAGATCAGAATGCTACGGTGAAT
ACGTTCCCGGGCCTTGTACACACCGCCCGTCACACCATGGGAGTGGGTTGCAAAAGAA
GTAGGTAGCTTAACCTTCGGGAGGGCGCTTACCACTTTGTGATTCATGACTGGGG
pattern 320
CGCTGGCGGCAGGCCTAACACATGCAAGTCGAGCGGCAGCGGGAAGTAGTTTACTACT
TTGCCGGCGAGCGGCGGACGGGTGAGTAATGTCTGGGAAACTGCCTGATGGAGGGGGA
TAACTACTGGAAACGGTAGCTAATACCGCATAACGTCTTCGGACCAAAGTGGGGGACCT
TCGGGCCTCACGCCATCGGATGTGCCCAGATGGGATTAGCTAGTAGGTGGGGTAATGGC
TCACCTAGGCGACGATCCCTAGCTGGTCTGAGAGGATGACCAGCCACACTGGAACTGA
GACACGGTCCAGACTCCTACGGGAGGCAGCAGTGGGGAATATTGCACAATGGGCGCAA
GCCTGATGCAGCCATGCCGCGTGTGTGAAGAAGGCCTTCGGGTTGTAAAGCACTTTCAG
CGAGGAGGAAGGCATAAAGGTTAATAACCTTTGTGATTGACGTTACTCGCAGAAGAAG
CACCGGCTAACTCCGTGCCAGCAGCCGCGGTAATACGGAGGGTGCAAGCGTTAATCGG
AATTACTGGGCGTAAAGCGCACGCAGGCGGTTTGTTAAGTCAGATGTGAAATCCCCGCG
CTTAACGTGGGAACTGCATTTGAAACTGGCAAGCTAGAGTCTTGTAGAGGGGGGTAGA
ATTCCAGGTGTAGCGGTGAAATGCGTAGAGATCTGGAGGAATACCGGTGGCGAAGGCG
GCCCCCTGGACAAAGACTGACGCTCAGGTGCGAAAGCGTGGGGAGCAAACAGGATTA
GATACCCTGGTAGTCCACGCTGTAAACGATGTCGACTTGGAAGTTGTGCCCTTGAGGCG
TGGCTTCCGGAGCTAACGCGTTAAGTCGACCGCCTGGGGAGTACGGCCGCAAGGTTAA
AACTCAAATGAATTGACGGGGGCCCGCACAAGCGGTGGAGCATGTGGTTTAATTCGATG
CAACGCGAAGAACCTTACCTACTCTTGACATCCACAGAACTTAGCAGAGATGCTTCGGT
GCCTTCGGGAACTGTGAGACAGGTGCTGCATGGCTGTCGTCAGCTCGTGTTGTGAAATG
TTGGGTTAAGTCCCGCAACGAGCGCAACCCTTATCCTTTGTTGCCAGCACGTCATGGTG
GGAACTCAAAGGAGACTGCCGGTGATAAACCGGAGGAAGGTGGGGATGACGTCAAGT
CATCATGGCCCTTACGAGTAGGGCTACACACGTGCTACAATGGCAGATACAAAGTGAAG
CGAACTCGCGAGAGCAAGCGGACCACATAAAGTCTGTCGTAGTCCGGATTGGAGTCTG
CAACTCGACTCCATGAAGTCGGAATCGCTAGTAATCGTAGATCAGAATGCTACGGTGAAT
ACGTTCCCGGGCCTTGTACACACCGCCCGTCACACCATGGGAGTGGGTTGCAAAAGAA
GTAGGTAGCTTAACCTTCGGGAGGGCGCTTACCACTTTGTGATTCATGACTGGGG
pattern 321
CGCTGGCGGCAGGCCTAACACATGCAAGTCGAGCGGCAGCGGGAAGTAGTTTACTACT
TTGCCGGCGAGCGGCGGACGGGTGAGTAATGTCTGGGAAACTGCCTGATGGAGGGGGA
TAACTACTGGAAACGGTAGCTAATACCGCATAACGTCTACGGACCAAAGTGGGGGACCT
TCGGGCCTCACGCCATCGGATGTGCCCAGATGGGATTAGCTAGTAGGTGGGGTAATGGC
TCACCTAGGCGACGATCCCTAGCTGGTCTGAGAGGATGACCAGCCACACTGGAACTGA
GACACGGTCCAGACTCCTACGGGAGGCAGCAGTGGGGAATATTGCACAATGGGCGCAA
GCCTGATGCAGCCATGCCGCGTGTGTGAAGAAGGCCTTCGGGTTGTAAAGCACTTTCAG
CGAGGAGGAAGGCATGAAGGTTAATAACCTTTGTGATTGACGTTACTCGCAGAAGAAG
CACCGGCTAACTCCGTGCCAGCAGCCGCGGTAATACGGAGGGTGCAAGCGTTAATCGG
AATTACTGGGCGTAAAGCGCACGCAGGCGGTTTGTTAAGTCAGATGTGAAATCCCCGCG
CTTAACGTGGGAACTGCATTTGAAACTGGCAAGCTAGAGTCTTGTAGAGGGGGGTAGA
ATTCCAGGTGTAGCGGTGAAATGCGTAGAGATCTGGAGGAATACCGGTGGCGAAGGCG

GCCCCCTGGACAAAGACTGACGCTCAGGTGCGAAAGCGTGGGGAGCAAACAGGATTA
GATACCCTGGTAGTCCACGCTGTAAACGATGTCGACTTGGAGGTTGTGCCCTTGAGGCG
TGGCTTCCGGAGCTAACGCGTTAAGTCGACCGCCTGGGGAGTACGGCCGCAAGGTTAA
AACTCAAATGAATTGACGGGGGCCCGCACAAGCGGTGGAGCATGTGGTTTAATTCGATG
CAACGCGAAGAACCTTACCTACTCTTGACATCCACAGAACTTAGCAGAGATGCTTCGGT
GCCTTCGGGAACTGTGAGACAGGTGCTGCATGGCTGTCGTCAGCTCGTGTTGTGAAATG
TTGGGTTAAGTCCCGCAACGAGCGCAACCCTTATCCTTTGTTGCCAGCACGTCATGGTG
GGAACTCAAAGGAGACTGCCGGTGATAAACCGGAGGAAGGTGGGGATGACGTCAAGT
CATCATGGCCCTTACGAGTAGGGCTACACACGTGCTACAATGGCAGATACAAAGTGAAG
CGAACTCGCGAGAGCAAGCGGACCACATAAAGTCTGTCGTAGTCCGGATTGGAGTCTG
CAACTCGACTCCATGAAGTCGGAATCGCTAGTAATCGTAGATCAGAATGCTACGGTGAAT
ACGTTCCCGGGCCTTGTACACACCGCCCGTCACACCATGGGAGTGGGTTGCAAAAGAA
GTAGGTAGCTTAACCTTCGGGAGGGCGCTTACCACTTTGTGATTCATGACTGGGG
pattern 322
CGCTGGCGGCAGGCCTAACACATGCAAGTCGAGCGGCAGCGGGGAGTAGTTTACTACT
TTGCCGGCGAGCGGCGGACGGGTGAGTAATGTCTGGGAAACTGCCTGATGGAGGGGGA
TAACTACTGGAAACGGTAGCTAATACCGCATAACGTCTTCGGACCAAAGTGGGGGACCT
TCGGGCCTCACGCCATCGGATGTGCCCAGATGGGATTAGCTAGTAGGTGGGGTAATGGC
TCACCTAGGCGACGATCCCTAGCTGGTCTGAGAGGATGACCAGCCACACTGGAACTGA
GACACGGTCCAGACTCCTACGGGAGGCAGCAGTGGGGAATATTGCACAATGGGCGCAA
GCCTGATGCAGCCATGCCGCGTGTGTGAAGAAGGCCTTCGGGTTGTAAAGCACTTTCAG
CGAGGAGGAAGGCATAAAGGTTAATAACCTTTGTGATTGACGTTACTCGCAGAAGAAG
CACCGGCTAACTCCGTGCCAGCAGCCGCGGTAATACGGAGGGTGCAAGCGTTAATCGG
AATTACTGGGCGTAAAGCGCACGCAGGCGGTTTGTTAAGTCAGATGTGAAATCCCCGCG
CTTAACGTGGGAACTGCATTTGAAACTGGCAAGCTAGAGTCTTGTAGAGGGGGGTAGA
ATTCCAGGTGTAGCGGTGAAATGCGTAGAGATCTGGAGGAATACCGGTGGCGAAGGCG
GCCCCCTGGACAAAGACTGACGCTCAGGTGCGAAAGCGTGGGGAGCAAACAGGATTA
GATACCCTGGTAGTCCACGCTGTAAACGATGTCGACTTGGAGGTTGTGCCCTTGAGGCG
TGGCTTCCGGAGCTAACGCGTTAAGTCGACCGCCTGGGGAGTACGGCCGCAAGGTTAA
AACTCAAATGAATTGACGGGGGCCCGCACAAGCGGTGGAGCATGTGGTTTAATTCGATG
CAACGCGAAGAACCTTACCTACTCTTGACATCCACGGAATTTAGCAGAGATGCTTTAGT
GCCTTCGGGAACCGTGAGACAGGTGCTGCATGGCTGTCGTCAGCTCGTGTTGTGAAATG
TTGGGTTAAGTCCCGCAACGAGCGCAACCCTTATCCTTTGTTGCCAGCACGTCATGGTG
GGAACTCAAAGGAGACTGCCGGTGATAAACCGGAGGAAGGTGGGGATGACGTCAAGT
CATCATGGCCCTTACGAGTAGGGCTACACACGTGCTACAATGGCAGATACAAAGTGAAG
CGAACTCGCGAGAGCAAGCGGACCACATAAAGTCTGTCGTAGTCCGGATTGGAGTCTG
CAACTCGACTCCATGAAGTCGGAATCGCTAGTAATCGTAGATCAGAATGCTACGGTGAAT
ACGTTCCCGGGCCTTGTACACACCGCCCGTCACACCATGGGAGTGGGTTGCAAAAGAA
GTAGGTAGCTTAACCTTCGGGAGGGCGCTTACCACTTTGTGATTCATGACTGGGG
pattern 323
CGCTGGCGGCAGGCCTAACACATGCAAGTCGAGCGGCAGCGGGAAGTAGTTTACTACT
TTGCCGGCGAGCGGCGGACGGGTGAGTAATGTCTGGGAAACTGCCTGATGGAGGGGGA
TAACTACTGGAAACGGTAGCTAATACCGCATAACGTCTTCGGACCAAAGTGGGGGACCT
TCGGGCCTCACGCCATCGGATGTGCCCAGATGGGATTAGCTAGTAGGTGGGGTAATGGC

TCACCTAGGCGACGATCCCTAGCTGGTCTGAGAGGATGACCAGCCACACTGGAACTGA
GACACGGTCCAGACTCCTACGGGAGGCAGCAGTGGGGAATATTGCACAATGGGCGCAA
GCCTGATGCAGCCATGCCGCGTGTGTGAAGAAGGCCTTCGGGTTGTAAAGCACTTTCAG
CGAGGAGGAAGGCATAAAGGTTAATAACCTTTGTGATTGACGTTACTCGCAGAAGAAG
CACCGGCTAACTCCGTGCCAGCAGCCGCGGTAATACGGAGGGTGCAAGCGTTAATCGG
AATTACTGGGCGTAAAGCGCACGCAGGCGGTTTGTTAAGTCAGATGTGAAATCCCCGCG
CTTAACGTGGGAACTGCATTTGAAACTGGCAAGCTAGAGTCTTGTAGAGGGGGGTAGA
ATTCCAGGTGTAGCGGTGAAATGCGTAGAGATCTGGAGGAATACCGGTGGCGAAGGCG
GCCCCCTGGACAAAGACTGACGCTCAGGTGCGAAAGCGTGGGGAGCAAACAGGATTA
GATACCCTGGTAGTCCACGCTGTAAACGATGTCGACTTGGAAGTTGTGCCCTTGAGGCG
TGGCTTCCGGAGCTAACGCGTTAAGTCGACCGCCTGGGGAGTACGGCCGCAAGGTTAA
AACTCAAATGAATTGACGGGGGCCCGCACAAGCGGTGGAGCATGTGGTTTAATTCGATG
CAACGCGAAGAACCTTACCTACTCTTGACATCCACGGAATTTAGCAGAGATGCTTTAGT
GCCTTCGGGAACTGTGAGACAGGTGCTGCATGGCTGTCGTCAGCTCGTGTTGTGAAATG
TTGGGTTAAGTCCCGCAACGAGCGCAACCCTTATCCTTTGTTGCCAGCACGTCATGGTG
GGAACTCAAAGGAGACTGCCGGTGATAAACCGGAGGAAGGTGGGGATGACGTCAAGT
CATCATGGCCCTTACGAGTAGGGCTACACACGTGCTACAATGGCAGATACAAAGTGAAG
CGAACTCGCGAGAGCAAGCGGACCACATAAAGTCTGTCGTAGTCCGGATTGGAGTCTG
CAACTCGACTCCATGAAGTCGGAATCGCTAGTAATCGTAGATCAGAATGCTACGGTGAAT
ACGTTCCCGGGCCTTGTACACACCGCCCGTCACACCATGGGAGTGGGTTGCAAAAGAA
GTAGGTAGCTTAACCTTCGGGAGGGCGCTTACCACTTTGTGATTCATGACTGGGG
pattern 324
CGCTGGCGGCAGGCCTAACACATGCAAGTCGAGCGGCAGCGGGAAGTAGTTTACTACT
TTGCCGGCGAGCGGCGGACGGGTGAGTAATGTCTGGGAAACTGCCTGATGGAGGGGGA
TAACTACTGGAAACGGTAGCTAATACCGCATAACGTCTTCGGACCAAAGTGGGGGACCT
TCGGGCCTCACGCCATCAGATGTGCCCAGATGGGATTAGCTAGTAGGTGGGGTAATGGC
TCACCTAGGCGACGATCCCTAGCTGGTCTGAGAGGATGACCAGCCACACTGGAACTGA
GACACGGTCCAGACTCCTACGGGAGGCAGCAGTGGGGAATATTGCACAATGGGCGCAA
GCCTGATGCAGCCATGCCGCGTGTGTGAAGAAGGCCTTCGGGTTGTAAAGCACTTTCAG
CGAGGAGGAAGGCATAAAGGTTAATAACCTTTGTGATTGACGTTACTCGCAGAAGAAG
CACCGGCTAACTCCGTGCCAGCAGCCGCGGTAATACGGAGGGTGCAAGCGTTAATCGG
AATTACTGGGCGTAAAGCGCACGCAGGCGGTTTGTTAAGTCAGATGTGAAATCCCCGCG
CTTAACGTGGGAACTGCATTTGAAACTGGCAAGCTAGAGTCTTGTAGAGGGGGGTAGA
ATTCCAGGTGTAGCGGTGAAATGCGTAGAGATCTGGAGGAATACCGGTGGCGAAGGCG
GCCCCCTGGACAAAGACTGACGCTCAGGTGCGAAAGCGTGGGGAGCAAACAGGATTA
GATACCCTGGTAGTCCACGCTGTAAACGATGTCGACTTGGAGGTTGTGCCCTTGAGGCG
TGGCTTCCGGAGCTAACGCGTTAAGTCGACCGCCTGGGGAGTACGGCCGCAAGGTTAA
AACTCAAATGAATTGACGGGGGCCCGCACAAGCGGTGGAGCATGTGGTTTAATTCGATG
CAACGCGAAGAACCTTACCTACTCTTGACATCCACGGAATTTAGCAGAGATGCTTTAGT
GCCTTCGGGAACCGTGAGACAGGTGCTGCATGGCTGTCGTCAGCTCGTGTTGTGAAATG
TTGGGTTAAGTCCCGCAACGAGCGCAACCCTTATCCTTTGTTGCCAGCACGTCATGGTG
GGAACTCAAAGGAGACTGCCGGTGATAAACCGGAGGAAGGTGGGGATGACGTCAAGT
CATCATGGCCCTTACGAGTAGGGCTACACACGTGCTACAATGGCAGATACAAAGTGAAG
CGAACTCGCGAGAGCAAGCGGACCACATAAAGTCTGTCGTAGTCCGGATTGGAGTCTG

CAACTCGACTCCATGAAGTCGGAATCGCTAGTAATCGTAGATCAGAATGCTACGGTGAAT
ACGTTCCCGGGCCTTGTACACACCGCCCGTCACACCATGGGAGTGGGTTGCAAAAGAA
GTAGGTAGCTTAACCTTCGGGAGGGCGCTTACCACTTTGTGATTCATGACTGGGG
pattern 325
CGCTGGCGGCAGGCCTAACACATGCAAGTCGAGCGGCAGCGGGAAGTAGTTTACTACT
TTGCCGGCGAGCGGCGGACGGGTGAGTAATGTCTGGGAAACTGCCTGATGGAGGGGGA
TAACTACTGGAAACGGTAGCTAATACCGCATAACGTCTTCGGACCAAAGTGGGGGACCT
TCGGGCCTCACGCCATCGGATGTGCCCAGATGGGATTAGCTAGTAGGTGGGGTAATGGC
TCACCTAGGCGACGATCCCTAGCTGGTCTGAGAGGATGACCAGCCACACTGGAACTGA
GACACGGTCCAGACTCCTACGGGAGGCAGCAGTGGGGAATATTGCACAATGGGCGCAA
GCCTGATGCAGCCATGCCGCGTGTGTGAAGAAGGCCTTCGGGTTGTAAAGCACTTTCAG
CGAGGAGGAAGGCATAAAGGTTAATAACCTTTGTGATTGACGTTACTCGCAGAAGAAG
CACCGGCTAACTCCGTGCCAGCAGCCGCGGTAATACGGAGGGTGCAAGCGTTAATCGG
AATTACTGGGCGTAAAGCGCACGCAGGCGGTTTGTTAAGTCAGATGTGAAATCCCCGCG
CTTAACGTGGGAACTGCATTTGAAACTGGCAAGCTAGAGTCTTGTAGAGGGGGGTAGA
ATTCCAGGTGTAGCGGTGAAATGCGTAGAGATCTGGAGGAATACCGGTGGCGAAGGCG
GCCCCCTGGACAAAGACTGACGCTCAGGTGCGAAAGCGTGGGGAGCAAACAGGATTA
GATACCCTGGTAGTCCACGCTGTAAACGATGTCGACTTGGAGGTTGTGCCCTTGAGGCG
TGGCTTCCGGAGCTAACGCGTTAAGTCGACCGCCTGGGGAGTACGGCCGCAAGGTTAA
AACTCAAATGAATTGACGGGGGCCCGCACCAGCGGTGGAGCATGTGGTTTAATTCGATG
CAACGCGAAGAACCTTACCTACTCTTGACATCCACGGAATTTAGCAGAGATGCTTTAGT
GCCTTCGGGAACCGTGAGACAGGTGCTGCATGGCTGTCGTCAGCTCGTGTTGTGAAATG
TTGGGTTAAGTCCCGCAACGAGCGCAACCCTTATCCTTTGTTGCCAGCACGTCATGGTG
GGAACTCAAAGGAGACTGCCGGTGATAAACCGGAGGAAGGTGGGGATGACGTCAAGT
CATCATGGCCCTTACGAGTAGGGCTACACACGTGCTACAATGGCAGATACAAAGTGAAG
CGAACTCGCGAGAGCAAGCGGACCACATAAAGTCTGTCGTAGTCCGGATTGGAGTCTG
CAACTCGACTCCATGAAGTCGGAATCGCTAGTAATCGTAGATCAGAATGCTACGGTGAAT
ACGTTCCCGGGCCTTGTACACACCGCCCGTCACACCATGGGAGTGGGTTGCAAAAGAA
GTAGGTAGCTTAACCTTCGGGAGGGCGCTTACCACTTTGTGATTCATGACTGGGG
pattern 326
CGCTGGCGGCAGGCCTAACACATGCAAGTCGAGCGGCAGCGGGGAGTAGTTTACTACT
TTGCCGGCGAGCGGCGGACGGGTGAGTAATGTCTGGGAAACTGCCTGATGGAGGGGGA
TAACTACTGGAAACGGTAGCTAATACCGCATAACGTCTTCGGACCAAAGTGGGGGACCT
TCGGGCCTCACGCCATCAGATGTGCCCAGATGGGATTAGCTAGTAGGTGGGGTAATGGC
TCACCTAGGCGACGATCCCTAGCTGGTCTGAGAGGATGACCAGCCACACTGGAACTGA
GACACGGTCCAGACTCCTACGGGAGGCAGCAGTGGGGAATATTGCACAATGGGCGCAA
GCCTGATGCAGCCATGCCGCGTGTGTGAAGAAGGCCTTCGGGTTGTAAAGCACTTTCAG
CGAGGAGGAAGGCATAAAGGTTAATAACCTTTGTGATTGACGTTACTCGCAGAAGAAG
CACCGGCTAACTCCGTGCCAGCAGCCGCGGTAATACGGAGGGTGCAAGCGTTAATCGG
AATTACTGGGCGTAAAGCGCACGCAGGCGGTTTGTTAAGTCAGATGTGAAATCCCCGCG
CTTAACGTGGGAACTGCATTTGAAACTGGCAAGCTAGAGTCTTGTAGAGGGGGGTAGA
ATTCCAGGTGTAGCGGTGAAATGCGTAGAGATCTGGAGGAATACCGGTGGCGAAGGCG
GCCCCCTGGACAAAGACTGACGCTCAGGTGCGAAAGCGTGGGGAGCAAACAGGATTA
GATACCCTGGTAGTCCACGCTGTAAACGATGTCGACTTGGAGGTTGTGCCCTTGAGGCG

TGGCTTCCGGAGCTAACGCGTTAAGTCGACCGCCTGGGGAGTACGGCCGCAAGGTTAA
AACTCAAATGAATTGACGGGGGCCCGCACAAGCGGTGGAGCATGTGGTTTAATTCGATG
CAACGCGAAGAACCTTACCTACTCTTGACATCCACGGAATTTAGCAGAGATGCTTTAGT
GCCTTCGGGAACCGTGAGACAGGTGCTGCATGGCTGTCGTCAGCTCGTGTTGTGAAATG
TTGGGTTAAGTCCCGCAACGAGCGCAACCCTTATCCTTTGTTGCCAGCACGTCATGGTG
GGAACTCAAAGGAGACTGCCGGTGATAAACCGGAGGAAGGTGGGGATGACGTCAAGT
CATCATGGCCCTTACGAGTAGGGCTACACACGTGCTACAATGGCAGATACAAAGTGAAG
CGAACTCGCGAGAGCAAGCGGACCACATAAAGTCTGTCGTAGTCCGGATTGGAGTCTG
CAACTCGACTCCATGAAGTCGGAATCGCTAGTAATCGTAGATCAGAATGCTACGGTGAAT
ACGTTCCCGGGCCTTGTACACACCGCCCGTCACACCATGGGAGTGGGTTGCAAAAGAA
GTAGGTAGCTTAACCTTCGGGAGGGCGCTTACCACTTTGTGATTCATGACTGGGG
pattern 327
CGCTGGCGGCAGGCCTAACACATGCAAGTCGAGCGGCAGCGGGAAGTAGTTTACTACT
TTGCCGGCGAGCGGCGGACGGGTGAGTAATGTCTGGGAAACTGCCTGATGGAGGGGGA
TAACTACTGGAAACGGTAGCTAATACCGCATAACGTCTTCGGACCAAAGTGGGGGACCT
TCGGGCCTCACGCCATCGGATGTGCCCAGATGGGATTAGCTAGTAGGTGGGGTAATGGC
TCACCTAGGCGACGATCCCTAGCTGGTCTGAGAGGATGACCAGCCACACTGGAACTGA
GACACGGTCCAGACTCCTACGGGAGGCAGCAGTGGGGAATATTGCACAATGGGCGCAA
GCCTGATGCAGCCATGCCGCGTGTGTGAAGAAGGCCTTCGGGTTGTAAAGCACTTTCAG
CGAGGAGGAAGGCATAAAGGTTAATAACCTTTGTGATTGACGTTACTCGCAGAAGAAG
CACCGGCTAACTCCGTGCCAGCAGCCGCGGTAATACGGAGGGTGCAAGCGTTAATCGG
AATTACTGGGCGTAAAGCGCACGCAGGCGGTTTGTTAAGTCAGATGTGAAATCCCCGCG
CTTAACGTGGGAACTGCATTTGAAACTGGCAAGCTAGAGTCTTGTAGAGGGGGGTAGA
ATTCCAGGTGTAGCGGTGAAATGCGTAGAGATCTGGAGGAATACCGGTGGCGAAGGCG
GCCCCCTGGACAAAGACTGACGCTCAGGTGCGAAAGCGTGGGGAGCAAACAGGATTA
GATACCCTGGTAGTCCACGCTGTAAACGATGTCGACTTGGAAGTTGTGCCCTTGAGGCG
TGGCTTCCGGAGCTAACGCGTTAAGTCGACCGCCTGGGGAGTACGGCCGCAAGGTTAA
AACTCAAATGAATTGACGGGGGCCCGCACAAGCGGTGGAGCATGTGGTTTAATTCGATG
CAACGCGAAGAACCTTACCTACTCTTGACATCCACGGAATTTAGCAGAGATGCTTTAGT
GCCTTCGGGAACCGTGAGACAGGTGCTGCATGGCTGTCGTCAGCTCGTGTTGTGAAATG
TTGGGTTAAGTCCCGCAACGAGCGCAACCCTTATCCTTTGTTGCCAGCACGTCATGGTG
GGAACTCAAAGGAGACTGCCGGTGATAAACCGGAGGAAGGTGGGGATGACGTCAAGT
CATCATGGCCCTTACGAGTAGGGCTACACACGTGCTACAATGGCAGATACAAAGTGAAG
CGAACTCGCGAGAGCAAGCGGACCACATAAAGTCTGTCGTAGTCCGGATTGGAGTCTG
CAACTCGACTCCATGAAGTCGGAATCGCTAGTAATCGTAGATCAGAATGCTACGGTGAAT
ACGTTCCCGGGCCTTGTACACACCGCCCGTCACACCATGGGAGTGGGTTGCAAAAGAA
GTAGGTAGCTTAACCTTCGGGAGGGCGCTTACCACTTTGTGATTCATGACTGGGG
pattern 328
CGCTGGCGGCAGGCCTAACACATGCAAGTCGAGCGGCAGCGGGGAGTAGTTTACTACT
TTGCCGGCGAGCGGCGGACGGGTGAGTAATGTCTGGGAAACTGCCTGATGGAGGGGGA
TAACTACTGGAAACGGTAGCTAATACCGCATAACGTCTTCGGACCAAAGTGGGGGACCT
TCGGGCCTCACGCCATCGGATGTGCCCAGATGGGATTAGCTAGTAGGTGGGGTAATGGC
TCACCTAGGCGACGATCCCTAGCTGGTCTGAGAGGATGACCAGCCACACTGGAACTGA
GACACGGTCCAGACTCCTACGGGAGGCAGCAGTGGGGAATATTGCACAATGGGCGCAA

GCCTGATGCAGCCATGCCGCGTGTGTGAAGAAGGCCTTCGGGTTGTAAAGCACTTTCAG
CGAGGAGGAAGGCATAAAGGTTAATAACCTTTGTGATTGACGTTACTCGCAGAAGAAG
CACCGGCTAACTCCGTGCCAGCAGCCGCGGTAATACGGAGGGTGCAAGCGTTAATCGG
AATTACTGGGCGTAAAGCGCACGCAGGCGGTTTGTTAAGTCAGATGTGAAATCCCCGCG
CTTAACGTGGGAACTGCATTTGAAACTGGCAAGCTAGAGTCTTGTAGAGGGGGGTAGA
ATTCCAGGTGTAGCGGTGAAATGCGTAGAGATCTGGAGGAATACCGGTGGCGAAGGCG
GCCCCCTGGACAAAGACTGACGCTCAGGTGCGAAAGCGTGGGGAGCAAACAGGATTA
GATACCCTGGTAGTCCACGCTGTAAACGATGTCGACTTGGAAGTTGTGCCCTTGAGGCG
TGGCTTCCGGAGCTAACGCGTTAAGTCGACCGCCTGGGGAGTACGGCCGCAAGGTTAA
AACTCAAATGAATTGACGGGGGCCCGCACAAGCGGTGGAGCATGTGGTTTAATTCGATG
CAACGCGAAGAACCTTACCTACTCTTGACATCCACGGAATTTAGCAGAGATGCTTTAGT
GCCTTCGGGAACTGTGAGACAGGTGCTGCATGGCTGTCGTCAGCTCGTGTTGTGAAATG
TTGGGTTAAGTCCCGCAACGAGCGCAACCCTTATCCTTTGTTGCCAGCACGTCATGGTG
GGAACTCAAAGGAGACTGCCGGTGATAAACCGGAGGAAGGTGGGGATGACGTCAAGT
CATCATGGCCCTTACGAGTAGGGCTACACACGTGCTACAATGGCAGATACAAAGTGAAG
CGAACTCGCGAGAGCAAGCGGACCACATAAAGTCTGTCGTAGTCCGGATTGGAGTCTG
CAACTCGACTCCATGAAGTCGGAATCGCTAGTAATCGTAGATCAGAATGCTACGGTGAAT
ACGTTCCCGGGCCTTGTACACACCGCCCGTCACACCATGGGAGTGGGTTGCAAAAGAA
GTAGGTAGCTTAACCTTCGGGAGGGCGCTTACCACTTTGTGATTCATGACTGGGG
pattern 329
CGCTGGCGGCAGGCCTAACACATGCAAGTCGAGCGGCAGCGGAAAGTAGTTTACTACT
TTGCCGGCGAGCGGCGGACGGGTGAGTAATGTCTGGGAAACTGCCTGATGGAGGGGGA
TAACTACTGGAAACGGTAGCTAATACCGCATAACGTCTTCGGACCAAAGTGGGGGACCT
TCGGGCCTCACGCCATCGGATGTGCCCAGATGGGATTAGCTAGTAGGTGGGGTAACGGC
TCACCTAGGCGACGATCCCTAGCTGGTCTGAGAGGATGACCAGCCACACTGGAACTGA
GACACGGTCCAGACTCCTACGGGAGGCAGCAGTGGGGAATATTGCACAATGGGCGCAA
GCCTGATGCAGCCATGCCGCGTGTGTGAAGAAGGCCTTCGGGTTGTAAAGCACTTTCAG
CGAGGAGGAAGGCATAAAGGTTAATAACCTTTGTGATTGACGTTACTCGCAGAAGAAG
CACCGGCTAACTCCGTGCCAGCAGCCGCGGTAATACGGAGGGTGCAAGCGTTAATCGG
AATTACTGGGCGTAAAGCGCACGCAGGCGGTTTGTTAAGTCAGATGTGAAATCCCCGCG
CTTAACGTGGGAACTGCATTTGAAACTGGCAAGCTAGAGTCTTGTAGAGGGGGGTAGA
ATTCCAGGTGTAGCGGTGAAATGCGTAGAGATCTGGAGGAATACCGGTGGCGAAGGCG
GCCCCCTGGACAAAGACTGACGCTCAGGTGCGAAAGCGTGGGGAGCAAACAGGATTA
GATACCCTGGTAGTCCACGCTGTAAACGATGTCGACTTGGAGGTTGTGCCCTTGAGGCG
TGGCTTCCGGAGCTAACGCGTTAAGTCGACCGCCTGGGGAGTACGGCCGCAAGGTTAA
AACTCAAATGAATTGACGGGGGCCCGCACAAGCGGTGGAGCATGTGGTTTAATTCGATG
CAACGCGAAGAACCTTACCTACTCTTGACATCCACGGAATTTAGCAGAGATGCTTTAGT
GCCTTCGGGAACCGTGAGACAGGTGCTGCATGGCTGTCGTCAGCTCGTGTTGTGAAATG
TTGGGTTAAGTCCCGCAACGAGCGCAACCCTTATCCTTTGTTGCCAGCACGTCATGGTG
GGAACTCAAAGGAGACTGCCGGTGATAAACCGGAGGAAGGTGGGGATGACGTCAAGT
CATCATGGCCCTTACGAGTAGGGCTACACACGTGCTACAATGGCAGATACAAAGTGAAG
CGAACTCGCGAGAGCAAGCGGACCACATAAAGTCTGTCGTAGTCCGGATTGGAGTCTG
CAACTCGACTCCATGAAGTCGGAATCGCTAGTAATCGTAGATCAGAATGCTACGGTGAAT
ACGTTCCCGGGCCTTGTACACACCGCCCGTCACACCATGGGAGTGGGTTGCAAAAGAA

GTAGGTAGCTTAACCTTCGGGAGGGCGCTTACCACTTTGTGATTCATGACTGGGG
pattern 330
CGCTGGCGGCAGGCCTAACACATGCAAGTCGAGCGGCAGCGGGAAGTAGTTTACTACT
TTGCCGGCGAGCGGCGGACGGGTGAGTAATGTCTGGGAAACTGCCTGATGGAGGGGGA
TAACTACTGGAAACGGTAGCTAATACCGCATAACGTCTTCGGACCAAAGTGGGGGACCT
TCGGGCCTCACGCCATCGGATGTGCCCAGATGGGATTAGCTAGTAGGTGGGGTAACGGC
TCACCTAGGCGACGATCCCTAGCTGGTCTGAGAGGATGACCAGCCACACTGGAACTGA
GACACGGTCCAGACTCCTACGGGAGGCAGCAGTGGGGAATATTGCACAATGGGCGCAA
GCCTGATGCAGCCATGCCGCGTGTGTGAAGAAGGCCTTCGGGTTGTAAAGCACTTTCAG
CGAGGAGGAAGGCATAAAGGTTAATAACCTTTGTGATTGACGTTACTCGCAGAAGAAG
CACCGGCTAACTCCGTGCCAGCAGCCGCGGTAATACGGAGGGTGCAAGCGTTAATCGG
AATTACTGGGCGTAAAGCGCACGCAGGCGGTTTGTTAAGTCAGATGTGAAATCCCCGCG
CTTAACGTGGGAACTGCATTTGAAACTGGCAAGCTAGAGTCTTGTAGAGGGGGGTAGA
ATTCCAGGTGTAGCGGTGAAATGCGTAGAGATCTGGAGGAATACCGGTGGCGAAGGCG
GCCCCCTGGACAAAGACTGACGCTCAGGTGCGAAAGCGTGGGGAGCAAACAGGATTA
GATACCCTGGTAGTCCACGCTGTAAACGATGTCGACTTGGAGGTTGTGCCCTTGAGGCG
TGGCTTCCGGAGCTAACGCGTTAAGTCGACCGCCTGGGGAGTACGGCCGCAAGGTTAA
AACTCAAATGAATTGACGGGGGCCCGCACAAGCGGTGGAGCATGTGGTTTAATTCGATG
CAACGCGAAGAACCTTACCTACTCTTGACATCCACGGAATTTAGCAGAGATGCTTTAGT
GCCTTCGGGAACCGTGAGACAGGTGCTGCATGGCTGTCGTCAGCTCGTGTTGTGAAATG
TTGGGTTAAGTCCCGCAACGAGCGCAACCCTTATCCTTTGTTGCCAGCACGTCATGGTG
GGAACTCAAAGGAGACTGCCGGTGATAAACCGGAGGAAGGTGGGGATGACGTCAAGT
CATCATGGCCCTTACGAGTAGGGCTACACACGTGCTACAATGGCAGATACAAAGTGAAG
CGAACTCGCGAGAGCAAGCGGACCACATAAAGTCTGTCGTAGTCCGGATTGGAGTCTG
CAACTCGACTCCATGAAGTCGGAATCGCTAGTAATCGTAGATCAGAATGCTACGGTGAAT
ACGTTCCCGGGCCTTGTACACACCGCCCGTCACACCATGGGAGTGGGTTGCAAAAGAA
GTAGGTAGCTTAACCTTCGGGAGGGCGCTTACCACTTTGTGATTCATGACTGGGG
pattern 331
CGCTGGCGGCAGGCCTAACACATGCAAGTCGAGCGGCAGCGGAAAGTAGTTTACTACT
TTGCCGGCGAGCGGCGGACGGGTGAGTAATGTCTGGGAAACTGCCTGATGGAGGGGGA
TAACTACTGGAAACGGTAGCTAATACCGCATAACGTCTACGGACCAAAGTGGGGGACCT
TCGGGCCTCACGCCATCGGATGTGCCCAGATGGGATTAGCTAGTAGGTGGGGTAATGGC
TCACCTAGGCGACGATCCCTAGCTGGTCTGAGAGGATGACCAGCCACACTGGAACTGA
GACACGGTCCAGACTCCTACGGGAGGCAGCAGTGGGGAATATTGCACAATGGGCGCAA
GCCTGATGCAGCCATGCCGCGTGTGTGAAGAAGGCCTTCGGGTTGTAAAGCACTTTCAG
CGAGGAGGAAGGCATAAAGGTTAATAACCTTTGTGATTGACGTTACTCGCAGAAGAAG
CACCGGCTAACTCCGTGCCAGCAGCCGCGGTAATACGGAGGGTGCAAGCGTTAATCGG
AATTACTGGGCGTAAAGCGCACGCAGGCGGTTTGTTAAGTCAGATGTGAAATCCCCGCG
CTTAACGTGGGAACTGCATTTGAAACTGGCAAGCTAGAGTCTTGTAGAGGGGGGTAGA
ATTCCAGGTGTAGCGGTGAAATGCGTAGAGATCTGGAGGAATACCGGTGGCGAAGGCG
GCCCCCTGGACAAAGACTGACGCTCAGGTGCGAAAGCGTGGGGAGCAAACAGGATTA
GATACCCTGGTAGTCCACGCTGTAAACGATGTCGACTTGGAGGTTGTGCCCTTGAGGCG
TGGCTTCCGGAGCTAACGCGTTAAGTCGACCGCCTGGGGAGTACGGCCGCAAGGTTAA
AACTCAAATGAATTGACGGGGGCCCGCACAAGCGGTGGAGCATGTGGTTTAATTCGATG

CAACGCGAAGAACCTTACCTACTCTTGACATCCACAGAACTTAGCAGAGATGCTTCGGT
GCCTTCGGGAACTGTGAGACAGGTGCTGCATGGCTGTCGTCAGCTCGTGTTGTGAAATG
TTGGGTTAAGTCCCGCAACGAGCGCAACCCTTATCCTTTGTTGCCAGCACGTCATGGTG
GGAACTCAAAGGAGACTGCCGGTGATAAACCGGAGGAAGGTGGGGATGACGTCAAGT
CATCATGGCCCTTACGAGTAGGGCTACACACGTGCTACAATGGCAGATACAAAGTGAAG
CGAACTCGCGAGAGCAAGCGGACCACATAAAGTCTGTCGTAGTCCGGATTGGAGTCTG
CAACTCGACTCCATGAAGTCGGAATCGCTAGTAATCGTAGATCAGAATGCTACGGTGAAT
ACGTTCCCGGGCCTTGTACACACCGCCCGTCACACCATGGGAGTGGGTTGCAAAAGAA
GTAGGTAGCTTAACCTTCGGGAGGGCGCTTACCACTTTGTGATTCATGACTGGGG
pattern 332
CGCTGGCGGCAGGCCTAACACATGCAAGTCGAGCGGCAGCGGGAAGTAGTTTACTACT
TTGCCGGCGAGCGGCGGACGGGTGAGTAATGTCTGGGGATCTGCCTGATGGAGGGGGA
TAACTACTGGAAACGGTAGCTAATACCGCATGACCTCGCAAGAGCAAAGTGGGGGACC
TTAGGGCCTCACGCCATCGGATGAACCCAGATGGGATTAGCTAGTAGGTGGGGTAATGG
CTCACCTAGGCGACGATCCCTAGCTGGTCTGAGAGGATGACCAGCCACACTGGAACTG
AGACACGGTCCAGACTCCTACGGGAGGCAGCAGTGGGGAATATTGCACAATGGGCGCA
AGCCTGATGCAGCCATGCCGCGTGTGTGAAGAAGGCCTTCGGGTTGTAAAGCACTTTCA
GCGAGGAGGAAGGGGTTGAGTTTAATACGCTCAATCATTGACGTTACTCGCAGAAGAA
GCACCGGCTAACTCCGTGCCAGCAGCCGCGGTAATACGGAGGGTGCAAGCGTTAATCG
GAATTACTGGGCGTAAAGCGCACGCAGGCGGTTTGTTAAGTCAGATGTGAAATCCCCGC
GCTTAACGTGGGAACTGCATTTGAAACTGGCAAGCTAGAGTCTTGTAGAGGGGGGTAG
AATTCCAGGTGTAGCGGTGAAATGCGTAGAGATCTGGAGGAATACCGGTGGCGAAGGC
GGCCCCCTGGACAAAGACTGACGCTCAGGTGCGAAAGCGTGGGGAGCAAACAGGATT
AGATACCCTGGTAGTCCACGCTGTAAACGATGTCGACTTGGAGGTTGTGCCCTTGAGGC
GTGGCTTCCGGAGCTAACGCGTTAAGTCGACCGCCTGGGGAGTACGGCCGCAAGGTTA
AAACTCAAATGAATTGACGGGGGCCCGCACAAGCGGTGGAGCATGTGGTTTAATTCGAT
GCAACGCGAAGAACCTTACCTACTCTTGACATCCACAGAATTTGGCAGAGATGCTTTAG
TGCCTTCGGGAACCGTGAGACAGGTGCTGCATGGCTGTCGTCAGCTCGTGTTGTGAAAT
GTTGGGTTAAGTCCCGCAACGAGCGCAACCCTTATCCTTTGTTGCCAGCACGTAATGGT
GGGAACTCAAGGGAGACTGCCGGTGACAAACCGGAGGAAGGTGGGGATGACGTCAAG
TCATCATGGCCCTTACGAGTAGGGCTACACACGTGCTACAATGGCAGATACAAAGTGAA
GCGAACTCGCGAGAGCCAGCGGACCACATAAAGTCTGTCGTAGTCCGGATTGGAGTCT
GCAACTCGACTCCATGAAGTCGGAATCGCTAGTAATCGTAGATCAGAATGCTACGGTGA
ATACGTTCCCGGGCCTTGTACACACCGCCCGTCACACCATGGGAGTGGGTTGCAAAAG
AAGTAGGTAGCTTAACCTTCGGGAGGGCGCTTACCACTTTGTGATTCATGACTGGGG
pattern 333
CGCTGGCGGCAGGCCTAACACATGCAAGTCGAGCGGCAGCGGGAAGTAGTTTACTACT
TTGCCGGCGAGCGGCGGACGGGTGAGTAATGTCTGGGAAACTGCCTGATGGAGGGGGA
TAACTACTGGAAACGGTAGCTAATACCGCATAACGTCTTCGGACCAAAGTGGGGGACCT
TCGGGCCTCACGCCATCGGATGTGCCCAGATGGGATTAGCTAGTAGGTGGGGTAATGGC
TCACCTAGGCGACGATCCCTAGCTGGTCTGAGAGGATGACCAGCCACACTGGAACTGA
GACACGGTCCAGACTCCTACGGGAGGCAGCAGTGGGGAATATTGCACAATGGGCGCAA
GCCTGATGCAGCCATGCCGCGTGTGTGAAGAAGGCCTTCGGGTTGTAAAGCACTTTCAG
CGAGGAGGAAGGCAGTCGTGTTAATAGCACGGTTGATTGACGTTACTCGCAGAAGAAG

CACCGGCTAACTCCGTGCCAGCAGCCGCGGTAATACGGAGGGTGCAAGCGTTAATCGG
AATTACTGGGCGTAAAGCGCACGCAGGCGGTTTGTTAAGTCAGATGTGAAATCCCCGCG
CTTAACGTGGGAACTGCATTTGAAACTGGCAAGCTAGAGTCTTGTAGAGGGGGGTAGA
ATTCCAGGTGTAGCGGTGAAATGCGTAGAGATCTGGAGGAATACCGGTGGCGAAGGCG
GCCCCCTGGACAAAGACTGACGCTCAGGTGCGAAAGCGTGGGGAGCAAACAGGATTA
GATACCCTGGTAGTCCACGCTGTAAACGATGTCGACTTGGAGGTTGTGCCCTTGAGGCG
TGGCTTCCGGAGCTAACGCGTTAAGTCGACCGCCTGGGGAGTACGGCCGCAAGGTTAA
AACTCAAATGAATTGACGGGGGCCCGCACAAGCGGTGGAGCATGTGGTTTAATTCGATG
CAACGCGAAGAACCTTACCTACTCTTGACATCCACAGAACTTAGCAGAGATGCTTAGGT
GCCTTCGGGAACTGTGAGACAGGTGCTGCATGGCTGTCGTCAGCTCGTGTTGTGAAATG
TTGGGTTAAGTCCCGCAACGAGCGCAACCCTTATCCTTTGTTGCCAGCACGTAATGGTG
GGAACTCAAGGGAGACTGCCGGTGACAAACCGGAGGAAGGTGGGGATGACGTCAAGT
CATCATGGCCCTTACGAGTAGGGCTACACACATGCTACAATGGCAGATACAAAGTGAAG
CGAACTCGCGAGAGCAAGCGGACCACATAAAGTCTGTCGTAGTCCGGATTGGAGTCTG
CAACTCGACTCCATGAAGTCGGAATCGCTAGTAATCGTAGATCAGAATGCTACGGTGAAT
ACGTTCCCGGGCCTTGTACACACCGCCCGTCACACCATGGGAGTGGGTTGCAAAAGAA
GTAGGTAGCTTAACCTTCGGGAGGGCGCTTACCACTTTGTGATTCATGACTGGGG
pattern 334
CGCTGGCGGCAGGCCTAACACATGCAAGTCGAGCGGCAGCGGGAAGTAGTTTACTACT
TTGCCGGCGAGCGGCGGACGGGTGAGTAATGTCTGGGAAACTGCCTGATGGAGGGGGA
TAACTACTGGAAACGGTAGCTAATACCGCATAACGTCTTCGGACCAAAGTGGGGGACCT
TCGGGCCTCACGCCATCGGATGTGCCCAGATGGGATTAGCTAGTAGGTGGGGTAATGGC
TCACCTAGGCGACGATCCCTAGCTGGTCTGAGAGGATGACCAGCCACACTGGAACTGA
GACACGGTCCAGACTCCTACGGGAGGCAGCAGTGGGGAATATTGCACAATGGGCGCAA
GCCTGATGCAGCCATGCCGCGTGTGTGAAGAAGGCCTTCGGGTTGTAAAGCACTTTCAG
CGAGGAGGAAGGCAGTCGTGTTAATAGCACGATTGATTGACGTTACTCGCAGAAGAAG
CACCGGCTAACTCCGTGCCAGCAGCCGCGGTAATACGGAGGGTGCAAGCGTTAATCGG
AATTACTGGGCGTAAAGCGCACGCAGGCGGTTTGTTAAGTCAGATGTGAAATCCCCGCG
CTTAACGTGGGAACTGCATTTGAAACTGGCAAGCTAGAGTCTTGTAGAGGGGGGTAGA
ATTCCAGGTGTAGCGGTGAAATGCGTAGAGATCTGGAGGAATACCGGTGGCGAAGGCG
GCCCCCTGGACAAAGACTGACGCTCAGGTGCGAAAGCGTGGGGAGCAAACAGGATTA
GATACCCTGGTAGTCCACGCTGTAAACGATGTCGACTTGGAGGTTGTGCCCTTGAGGCG
TGGCTTCCGGAGCTAACGCGTTAAGTCGACCGCCTGGGGAGTACGGCCGCAAGGTTAA
AACTCAAATGAATTGACGGGGGCCCGCACAAGCGGTGGAGCATGTGGTTTAATTCGATG
CAACGCGAAGAACCTTACCTACTCTTGACATCCACGGAATTTAGCAGAGATGCTTTAGT
GCCTTCGGGAACCGTGAGACAGGTGCTGCATGGCTGTCGTCAGCTCGTGTTGTGAAATG
TTGGGTTAAGTCCCGCAACGAGCGCAACCCTTATCCTTTGTTGCCAGCACGTAATGGTG
GGAACTCAAGGGAGACTGCCGGTGACAAACCGGAGGAAGGTGGGGATGACGTCAAGT
CATCATGGCCCTTACGAGTAGGGCTACACACGTGCTACAATGGCAGATACAAAGTGAAG
CGAACTCGCGAGAGCAAGCGGACCACATAAAGTCTGTCGTAGTCCGGATTGGAGTCTG
CAACTCGACTCCATGAAGTCGGAATCGCTAGTAATCGTAGATCAGAATGCTACGGTGAAT
ACGTTCCCGGGCCTTGTACACACCGCCCGTCACACCATGGGAGTGGGTTGCAAAAGAA
GTAGGTAGCTTAACCTTCGGGAGGGCGCTTACCACTTTGTGATTCATGACTGGGG
pattern 335

CGCTGGCGGCAGGCCTAACACATGCAAGTCGAGCGGCAGCGGGAAGTAGTTTACTACT
TTGCCGGCGAGCGGCGGACGGGTGAGTAATGTCTGGGAAACTGCCTGATGGAGGGGGA
TAACTACTGGAAACGGTAGCTAATACCGCATAACGTCTTCGGACCAAAGTGGGGGACCT
TCGGGCCTCACGCCATCGGATGTGCCCAGATGGGATTAGCTAGTAGGTGGGGTAATGGC
TCACCTAGGCGACGATCCCTAGCTGGTCTGAGAGGATGACCAGCCACACTGGAACTGA
GACACGGTCCAGACTCCTACGGGAGGCAGCAGTGGGGAATATTGCACAATGGGCGCAA
GCCTGATGCAGCCATGCCGCGTGTGTGAAGAAGGCCTTCGGGTTGTAAAGCACTTTCAG
CGAGGAGGAAGGCAGTCGTGTTAATAGCACGGTTGATTGACGTTACTCGCAGAAGAAG
CACCGGCTAAACTCCGTGCCAGCAGCCGCGGTAATACGGAGGGTGCAAGCGTTAATCG
GAATTACTGGGCGTAAAGCGCACGCAGGCGGTTTGTTAAGTCAGATGTGAAATCCCCGC
GCTTAACGTGGGAACTGCATTTGAAACTGGCAAGCTAGAGTCTTGTAGAGGGGGGTAG
AATTCCAGGTGTAGCGGTGAAATGCGTAGAGATCTGGAGGAATACCGGTGGCGAAGGC
GGCCCCTGGACAAAGACTGACGCTCAGGTGCGAAAGCGTGGGGAGCAAACAGGATTA
GATACCCTGGTAGTCCACGCTGTAAACGATGTCGACTTGGAGGTTGTGCCCTTGAGGCG
TGGCTTCCGGAGCTAACGCGTTAAGTCGACCGCCTGGGGAGTACGGCCGCAAGGTTAA
AACTCAAATGAATTGACGGGGGCCCGCACAAGCGGTGGAGCATGTGGTTTAATTCGATG
CAACGCGAAGAACCTTACCTACTCTTGACATCCACAGAACTTAGCAGAGATGCTTAGGT
GCCTTCGGGAACTGTGAGACAGGTGCTGCATGGCTGTCGTCAGCTCGTGTTGTGAAATG
TTGGGTTAAGTCCCGCAACGAGCGCAACCCTTATCCTTTGTTGCCAGCACGTAATGGTG
GGAACTCAAGGGAGACTGCCGGTGACAAACCGGAGGAAGGTGGGGATGACGTCAAGT
CATCATGGCCCTTACGAGTAGGGCTACACACGTGCTACAATGGCAGATACAAAGTGAAG
CGAACTCGCGAGAGCAAGCGGACCACATAAAGTCTGTCGTAGTCCGGATTGGAGTCTG
CAACTCGACTCCATGAAGTCGGAATCGCTAGTAATCGTAGATCAGAATGCTACGGTGAAT
ACGTTCCCGGGCCTTGTACACACCGCCCGTCACACCATGGGAGTGGGTTGCAAAAGAA
GTAGGTAGCTTAACCTTCGGGAGGGCGCTTACCACTTTGTGATTCATGACTGGGG
pattern 336
CGCTGGCGGCAGGCCTAACACATGCAAGTCGAGCGGCAGCGGGAAGTAGTTTACTACT
TTGCCGGCGAGCGGCGGACGGGTGAGTAATGTCTGGGAAACTGCCTGATGGAGGGGGA
TAACTACTGGAAACGGTAGCTAATACCGCATAACGTCTTCGGACCAAAGTGGGGGACCT
TCGGGCCTCACGCCATCGGATGTGCCCAGATGGGATTAGCTAGTAGGTGGGGTAATGGC
TCACCTAGGCGACGATCCCTAGCTGGTCTGAGAGGATGACCAGCCACACTGGAACTGA
GACACGGTCCAGACTCCTACGGGAGGCAGCAGTGGGGAATATTGCACAATGGGCGCAA
GCCTGATGCAGCCATGCCGCGTGTGTGAAGAAGGCCTTCGGGTTGTAAAGCACTTTCAG
CGAGGAGGAAGGCAGTCGTGTTAATAGCACGGTTGATTGACGTTACTCGCAGAAGAAG
CACCGGCTAACTCCGTGCCAGCAGCCGCGGTAATACGGAGGGTGCAAGCGTTAATCGG
AATTACTGGGCGTAAAGCGCACGCAGGCGGTTTGTTAAGTCAGATGTGAAATCCCCGCG
CTTAACGTGGGAACTGCATTTGAAACTGGCAAGCTAGAGTCTTGTAGAGGGGGGTAGA
ATTCCAGGTGTAGCGGTGAAATGCGTAGAGATCTGGAGGAATACCGGTGGCGAAGGCG
GCCCCCTGGACAAAGACTGACGCTCAGGTGCGAAAGCGTGGGGAGCAAACAGGATTA
GATACCCTGGTAGTCCACGCTGTAAACGATGTCGACTTGGAGGTTGTGCCCTTGAGGCG
TGGCTTCCGGAGCTAACGCGTTAAGTCGACCGCCTGGGGAGTACGGCCGCAAGGTTAA
AACTCAAATGAATTGACGGGGGCCCGCACAAGCGGTGGAGCATGTGGTTTAATTCGATG
CAACGCGAAGAACCTTACCTACTCTTGACATCCACAGAACTTAGCAGAGATGCTTAGGT
GCCTTCGGGAACTGTGAGACAGGTGCTGCATGGCTGTCGTCAGCTCGTGTTGTGAAATG

TTGGGTTAAGTCCCGCAACGAGCGCAACCCTTATCCTTTGTTGCCAGCACGTAATGGTG
GGAACTCAAGGGAGACTGCCGGTGACAAACCGGAGGAAGGTGGGGATGACGTCAAGT
CATCATGGCCCTTACGAGTAGGGCTACACACGTGCTACAATGGCAGATACAAAGTGAAG
CGAACTCGCGAGAGCAAGCGGACCACATAAAGTCTGTCGTAGTCCGGATTGGAGTCTG
CAACTCGACTCCATGAAGTCGGAATCGCTAGTAATCGTAGATCAGAATGCTACGGTGAAT
ACGTTCCCGGGCCTTGTACACACCGCCCGTCACACCATGGGAGTGGGTTGCAAAAGAA
GTAGGTAGCTTAACCTTCGGGAGGGCGCTTACCACTTTGTGATTCATGACTGGGG
pattern 337
CGCTGGCGGCAGGCCTAACACATGCAAGTCGAGCGGCAGCGGGAAGTAGTTTACTACT
TTGCCGGCGAGCGGCGGACGGGTGAGTAATGTCTGGGGATCTGCCTGATGGAGGGGGA
TAACTACTGGAAACGGTAGCTAATACCGCATGACCTCGCAAGAGCAAAGTGGGGGACC
TTAGGGCCTCACGCCATCGGATGAACCCAGATGGGATTAGCTAGTAGGTGGGGTAATGG
CTCACCTAGGCGACGATCCCTAGCTGGTCTGAGAGGATGACCAGCCACACTGGAACTG
AGACACGGTCCAGACTCCTACGGGAGGCAGCAGTGGGGAATATTGCACAATGGGCGCA
AGCCTGATGCAGCCATGCCGCGTGTGTGAAGAAGGCCTTCGGGTTGTAAAGCACTTTCA
GCGAGGAGGAAGGGGTTGAGTTTAATACGCTCAATCATTGACGTTACTCGCAGAAGAA
GCACCGGCTAACTCCGTGCCAGCAGCCGCGGTAATACGGAGGGTGCAAGCGTTAATCG
GAATTACTGGGCGTAAAGCGCACGCAGGCGGTTTGTTAAGTCAGATGTGAAATCCCCGC
GCTTAACGTGGGAACTGCATTTGAAACTGGCAAGCTAGAGTCTTGTAGAGGGGGGTAG
AATTCCAGGTGTAGCGGTGAAATGCGTAGAGATCTGGAGGAATACCGGTGGCGAAGGC
GGCCCCCTGGACAAAGACTGACGCTCAGGTGCGAAAGCGTGGGGAGCAAACAGGATT
AGATACCCTGGTAGTCCACGCTGTAAACGATGTCGACTTGGAGGTTGTGCCCTTGAGGC
GTGGCTTCCGGAGCTAACGCGTTAAGTCGACCGCCTGGGGAGTACGGCCGCAAGGTTA
AAACTCAAATGAATTGACGGGGGCCCGCACAAGCGGTGGAGCATGTGGTTTAATTCGAT
GCAACGCGAAGAACCTTACCTACTCTTGACATCCACAGAATTTGGCAGAGATGCTAAAG
TGCCTTCGGGAACTGTGAGACAGGTGCTGCATGGCTGTCGTCAGCTCGTGTTGTGAAAT
GTTGGGTTAAGTCCCGCAACGAGCGCAACCCTTATCCTTTGTTGCCAGCACGTAATGGT
GGGAACTCAAGGGAGACTGCCGGTGACAAACCGGAGGAAGGTGGGGATGACGTCAAG
TCATCATGGCCCTTACGAGTAGGGCTACACACGTGCTACAATGGCAGATACAAAGTGAA
GCGAACTCGCGAGAGCCAGCGGACCACATAAAGTCTGTCGTAGTCCGGATTGGAGTCT
GAAACTCGACTCCATGAAGTCGGAATCGCTAGTAATCGTAGATCAGAATGCTACGGTGA
ATACGTTCCCGGGCCTTGTACACACCGCCCGTCACACCATGGGAGTGGGTTGCAAAAG
AAGTAGGTAGCTTAACCTTCGGGAGGGCGCTTACCACTTTGTGATTCATGACTGGGG
pattern 338
CGCTGGCGGCAGGCCTAACACATGCAAGTCGAGCGGCAGCGGGAAGTAGTTTACTACT
TTGCCGGCGAGCGGTGGACGGGTGAGTAATGTCTGGGGATCTGCCTGATGGAGGGGGA
TAACTACTGGAAACGGTGGCTAATACCGCATGACCTCGCAAGAGCAAAGTGGGGGACC
TTAGGGCCTCACGCCATCGGATGAACCCAGATGGGATTAGCTAGTAGGTGGGGTAATGG
CTCACCTAGGCGACGATCCCTAGCTGGTCTGAGAGGATGACCAGCCACACTGGAACTG
AGACACGGTCCAGACTCCTACGGGAGGCAGCAGTGGGGAATATTGCACAATGGGCGCA
AGCCTGATGCAGCCATGCCGCGTGTGTGAAGAAGGCCTTCGGGTTGTAAAGCACTTTCA
GCGAGGAGGAAGGGGTTGAGTTTAATACGCTCAATCATTGACGTTACTCGCAGAAGAA
GCACCGGCTAACTCCGTGCCAGCAGCCGCGGTAATACGGAGGGTGCAAGCGTTAATCG
GAATTACTGGGCGTAAAGCGCACGCAGGCGGTTTGTTAAGTCAGATGTGAAATCCCCGC

GCTTAACGTGGGAACTGCATTTGAAACTGGCAAGCTAGAGTCTTGTAGAGGGGGGTAG
AATTCCAGGTGTAGCGGTGAAATGCGTAGAGATCTGGAGGAATACCGGTGGCGAAGGC
GGCCCCCTGGACAAAGACTGACGCTCAGGTGCGAAAGCGTGGGGAGCAAACAGGATT
AGATACCCTGGTAGTCCACGCTGTAAACGATGTCGACTTGGAGGTTGTGCCCTTGAGGC
GTGGCTTCCGGAGCTAACGCGTTAAGTCGACCGCCTGGGGAGTACGGCCGCAAGGTTA
AAACTCAAATGAATTGACGGGGGCCCGCACAAGCGGTGGAGCATGTGGTTTAATTCGAT
GCAACGCGAAGAACCTTACCTACTCTTGACATCCACAGAATTTGGCAGAGATGCTAAAG
TGCCTTCGGGAACTGTGAGACAGGTGCTGCATGGCTGTCGTCAGCTCGTGTTGTGAAAT
GTTGGGTTAAGTCCCGCAACGAGCGCAACCCTTATCCTTTGTTGCCAGCACGTAATGGT
GGGAACTCAAGGGAGACTGCCGGTGACAAACCGGAGGAAGGTGGGGATGACGTCAAG
TCATCATGGCCCTTACGAGTAGGGCTACACACGTGCTACAATGGCAGATACAAAGTGAA
GCGAACTCGCGAGAGCCAGCGGACCACATAAAGTCTGTCGTAGTCCGGATTGGAGTCT
GCAACTCGACTCCATGAAGTCGGAATCGCTAGTAATCGTAGATCAGAATGCTACGGTGA
ATACGTTCCCGGGCCTTGTACACACCGCCCGTCACACCATGGGAGTGGGTTGCAAAAG
AAGTAGGTAGCTTAACCTTCGGGAGGGCGCTTACCACTTTGTGATTCATGACTGGGG
pattern 339
CGCTGGCGGCAGGCCTAACACATGCAAGTCGAGCGGCAGCGGGAAGTGGTTTACTACT
TTGCCGGCGAGCGGCGGACGGGTGAGTAATGTCTGGGAAACTGCCTGATGGAGGGGGA
TAACTACTGGAAACGGTAGCTAATACCGCATGACCTCGCAAGAGCAAAGTGGGGGACC
TTCGGGCCTCACGCCATCGGATGTGCCCAGATGGGATTAGCTAGTAGGTGGGGTAATGG
CTCACCTAGGCGACGATCCCTAGCTGGTCTGAGAGGATGACCAGCCACACTGGAACTG
AGACACGGTCCAGACTCCTACGGGAGGCAGCAGTGGGGAATATTGCACAATGGGCGCA
AGCCTGATGCAGCCATGCCGCGTGTGTGAAGAAGGCCTTCGGGTTGTAAAGCACTTTCA
GCGAGGAGGAAGGCAGTCGTGTTAATAGCACGATTGATTGACGTTACTCGCAGAAGAA
GCACCGGCTAACTCCGTGCCAGCAGCCGCGGTAATACGGAGGGTGCAAGCGTTAATCG
GAATTACTGGGCGTAAAGCGCACGCAGGCGGTTTGTTAAGTCAGATGTGAAATCCCCGC
GCTTAACGTGGGAACTGCATTTGAAACTGGCAAGCTAGAGTCTTGTAGAGGGGGGTAG
AATTCCAGGTGTAGCGGTGAAATGCGTAGAGATCTGGAGGAATACCGGTGGCGAAGGC
GGCCCCCTGGACAAAGACTGACGCTCAGGTGCGAAAGCGTGGGGAGCAAACAGGATT
AGATACCCTGGTAGTCCACGCTGTAAACGATGTCGACTTGGAGGTTGTGCCCTTGAGGC
GTGGCTTCCGGAGCTAACGCGTTAAGTCGACCGCCTGGGGAGTACGGCCGCAAGGTTA
AAACTCAAATGAATTGACGGGGGCCCGCACAAGCGGTGGAGCATGTGGTTTAATTCGAT
GCAACGCGAAGAACCTTACCTACTCTTGACATCCACAGAACTTAGCAGAGATGCTTCGG
TGCCTTCGGGAACTGTGAGACAGGTGCTGCATGGCTGTCGTCAGCTCGTGTTGTGAAAT
GTTGGGTTAAGTCCCGCAACGAGCGCAACCCTTATCCTTTGTTGCCAGCACGTAATGGT
GGGAACTCAAGGGAGACTGCCGGTGACAAACCGGAGGAAGGTGGGGATGACGTCAAG
TCATCATGGCCCTTACGAGTAGGGCTACACACGTGCTACAATGGCAGATACAAAGTGAA
GCGAACTCGCGAGAGCAAGCGGACCACATAAAGTCTGTCGTAGTCCGGATTGGAGTCT
GCAACTCGACTCCATGAAGTCGGAATCGCTAGTAATCGTAGATCAGAATGCTACGGTGA
ATACGTTCCCGGGCCTTGTACACACCGCCCGTCACACCATGGGAGTGGGTTGCAAAAG
AAGTAGGTAGCTTAACCTTCGGGAGGGCGCTTACCACTTTGTGATTCATGACTGGGG
pattern 340
CGCTGGCGGCAGGCCTAACACATGCAAGTCGAGCGGCAGCGGGAAGTAGTTTACTACT
TTGCCGGCGAGCGGAGGACGGGTGAGTAATGTCTGGGAAACTGCCTGATGGAGGGGGA

TAACTACTGGAAACGGTAGCTAATACCGCATGACCTCGCAAGAGCAAAGTGGGGGACC
TTAGGGCCTCACGCCATCGGATGTGCCCAGATGGGATTAGCTAGTAGGTGGGGTAATGG
CTCACCTAGGCGACGATCCCTAGCTGGTCTGAGAGGATGACCAGCCACACTGGAACTG
AGACACGGTCCAGACTCCTACGGGAGGCAGCAGTGGGGAATATTGCACAATGGGCGCA
AGCCTGATGCAGCCATGCCGCGTGTGTGAAGAAGGCCTTCGGGTTGTAAAGCACTTTCA
GCGAGGAGGAAGGCAATCGTGTTAATAGCACGGTTGATTGACGTTACTCGCAGAAGAA
GCACCGGCTAACTCCGTGCCAGCAGCCGCGGTAATACGGAGGGTGCAAGCGTTAATCG
GAATTACTGGGCGTAAAGCGCACGCAGGCGGTTTGTTAAGTCAGATGTGAAATCCCCGC
GCTTAACGTGGGAACTGCATTTGAAACTGGCAAGCTAGAGTCTTGTAGAGGGGGGTAG
AATTCCAGGTGTAGCGGTGAAATGCGTAGAGATCTGGAGGAATACCGGTGGCGAAGGC
GGCCCCCTGGACAAAGACTGACGCTCAGGTGCGAAAGCGTGGGGAGCAAACAGGATT
AGATACCCTGGTAGTCCACGCTGTAAACGATGTCGACTTGGAGGTTGTGCCCTTGAGGC
GTGGCTTCCGGAGCTAACGCGTTAAGTCGACCGCCTGGGGAGTACGGCCGCAAGGTTA
AAACTCAAATGAATTGACGGGGGCCCGCACAAGCGGTGGAGCATGTGGTTTAATTCGAT
GCAACGCGAAGAACCTTACCTACTCTTGACATCCACAGAACTTAGCAGAGATGCTTCGG
TGCCTTCGGGAACTGTGAGACAGGTGCTGCATGGCTGTCGTCAGCTCGTGTTGTGAAAT
GTTGGGTTAAGTCCCGCAACGAGCGCAACCCTTATCCTTTGTTGCCAGCACGTAATGGT
GGGAACTCAAGGGAGACTGCCGGTGACAAACCGGAGGAAGGTGGGGATGACGTCAAG
TCATCATGGCCCTTACGAGTAGGGCTACACACGTGCTACAATGGCAGATACAAAGTGAA
GCGAACTCGCGAGAGCAAGCGGACCACATAAAGTCTGTCGTAGTCCGGATTGGAGTCT
GCAACTCGACTCCATGAAGTCGGAATCGCTAGTAATCGTAGATCAGAATGCTACGGTGA
ATACGTTCCCGGGCCTTGTACACACCGCCCGTCACACCATGGGAGTGGGTTGCAAAAG
AAGTAGGTAGCTTAACCTTCGGGAGGGCGCTTACCACTTTGTGATTCATGACTGGGG
pattern 341
CGCTGGCGGCAGGCCTAACACATGCAAGTCGAGCGGCAGCGGAAAGTAGCTTGCTACT
TTGCCGGCGAGCGGCGGACGGGTGAGTAATGTCTGGGGATCTGCCTAATGGAGGGGGA
TAACTACTGGAAACGGTAGCTAATACCGCATGACCTCGAAAGAGCAAAGTGGGGGACC
TTCGGGCCTCACGCCATCGGATGAACCCAGATGGGATTAGCTAGTAGGTGGGGTAATGG
CTCACCTAGGCGACGATCCCTAGCTGGTCTGAGAGGATGACCAGCCACACTGGAACTG
AGACACGGTCCAGACTCCTACGGGAGGCAGCAGTGGGGAATATTGCACAATGGGCGCA
AGCCTGATGCAGCCATGCCGCGTGTGTGAAGAAGGCCTTCGGGTTGTAAAGCACTTTCA
GCGAGGAGGAAGGCATTGTGGTTAATAACCGCAGTGATTGACGTTACTCGCAGAAGAA
GCACCGGCTAACTCCGTGCCAGCAGCCGCGGTAATACGGAGGGTGCAAGCGTTAATCG
GAATTACTGGGCGTAAAGCGCACGCAGGCGGTTTGTTAAGTCAGATGTGAAATCCCCGC
GCTTAACGTGGGAACTGCATTTGAAACTGGCAAGCTAGAGTCTTGTAGAGGGGGGTAG
AATTCCAGGTGTAGCGGTGAAATGCGTAGAGATCTGGAGGAATACCGGTGGCGAAGGC
GGCCCCCTGGACAAAGACTGACGCTCAGGTGCGAAAGCGTGGGGAGCAAACAGGATT
AGATACCCTGGTAGTCCACGCTGTAAACGATGTCGACTTGGAGGTTGTGCCCTTGAGGC
GTGGCTTCCGGAGCTAACGCGTTAAGTCGACCGCCTGGGGAGTACGGCCGCAAGGTTA
AAACTCAAATGAATTGACGGGGGCCCGCACAAGCGGTGGAGCATGTGGTTTAATTCGAT
GCAACGCGAAGAACCTTACCTACTCTTGACATCCACGGAATTTAGCAGAGATGCTTTAG
TGCCTTCGGGAACCGTGAGACAGGTGCTGCATGGCTGTCGTCAGCTCGTGTTGTGAAAT
GTTGGGTTAAGTCCCGCAACGAGCGCAACCCTTATCCTTTGTTGCCAGCACGTAATGGT
GGGAACTCAAGGGAGACTGCCGGTGACAAACCGGAGGAAGGTGGGGATGACGTCAAG

TCATCATGGCCCTTACGAGTAGGGCTACACACGTGCTACAATGGCAGATACAAAGTGAA
GCGAACTCGCGAGAGCAAGCGGACCACATAAAGTCTGTCGTAGTCCGGATTGGAGTCT
GCAACTCGACTCCATGAAGTCGGAATCGCTAGTAATCGTAGATCAGAATGCTACGGTGA
ATACGTTCCCGGGCCTTGTACACACCGCCCGTCACACCATGGGAGTGGGTTGCAAAAG
AAGTAGGTAGCTTAACCTTCGGGAGGGCGCTTACCACTTTGTGATTCATGACTGGGG
pattern 342
CGCTGGCGGCAGGCCTAACACATGCAAGTCGAGCGGCAGCGGGAAGTAGTTTACTACT
TTGCCGGCGAGCGGCGGACGGGTGAGTAATGTCTGGGAAACTGCCTGATGGAGGGGGA
TAACTACTGGAAACGGTAGCTAATACCGCATGACCTCGCAAGAGCAAAGTGGGGGACC
TTCGGGCCTCACGCCATCGGATGTGCCCAGATGGGATTGGCTAGTAGGTGGGGTAATGG
CTCACCTAGGCGACGATCCCTAGCTGGTCTGAGAGGATGACCAGCCACACTGGAACTG
AGACACGGTCCAGACTCCTACGGGAGGCAGCAGTGGGGAATATTGCACAATGGGCGCA
AGCCTGATGCAGCCATGCCGCGTGTGTGAAGAAGGCCTTCGGGTTGTAAAGCACTTTCA
GCGAGGAGGAAGGCAGTCGTGTTAATAGCACGATTGATTGACGTTACTCGCAGAAGAA
GCACCGGCTAACTCCGTGCCAGCAGCCGCGGTAATACGGAGGGTGCAAGCGTTAATCG
GAATTACTGGGCGTAAAGCGCACGCAGGCGGTTTGTTAAGTCAGATGTGAAATCCCCGC
GCTTAACGTGGGAACTGCATTTGAAACTGGCAAGCTAGAGTCTTGTAGAGGGGGGTAG
AATTCCAGGTGTAGCGGTGAAATGCGTAGAGATCTGGAGGAATACCGGTGGCGAAGGC
GGCCCCCTGGACAAAGACTGACGCTCAGGTGCGAAAGCGTGGGGAGCAAACAGGATT
AGATACCCTGGTAGTCCACGCTGTAAACGATGTCGACTTGGAGGTTGTGCCCTTGAGGC
GTGGCTTCCGGAGCTAACGCGTTAAGTCGACCGCCTGGGGAGTACGGCCGCAAGGTTA
AAACTCAAATGAATTGACGGGGGCCCGCACAAGCGGTGGAGCATGTGGTTTAATTCGAT
GCAACGCGAAGAACCTTACCTACTCTTGACATCCACAGAACTTAGCAGAGATGCTTCGG
TGCCTTCGGGAACTGTGAGACAGGTGCTGCATGGCTGTCGTCAGCTCGTGTTGTGAAAT
GTTGGGTTAAGTCCCGCAACGAGCGCAACCCTTATCCTTTGTTGCCAGCACGTAATGGT
GGGAACTCAAGGGAGACTGCCGGTGACAAACCGGAGGAAGGTGGGGATGACGTCAAG
TCATCATGGCCCTTACGAGTAGGGCTACACACGTGCTACAATGGCAGATACAAAGTGAA
GCGAACTCGCGAGAGCAAGCGGACCACATAAAGTCTGTCGTAGTCCGGATTGGAGTCT
GCAACTCGACTCCATGAAGTCGGAATCGCTAGTAATCGTAGATCAGAATGCTACGGTGA
ATACGTTCCCGGGCCTTGTACACACCGCCCGTCACACCATGGGAGTGGGTTGCAAAAG
AAGTAGGTAGCTTAACCTTCGGGAGGGCGCTTACCACTTTGTGATTCATGACTGGGG
pattern 343
CGCTGGCGGCAGGCCTAACACATGCAAGTCGAGCGGCAGCGGAAAGTAGCTTGCTACT
TTGCCGGCGAGCGGCGGACGGGTGAGTAATGTCTGGGGATCTGCCTAATGGAGGGGGA
TAACTACTGGAAACGGTAGCTAATACCGCATGACCTCGAAAGAGCAAAGTGGGGGACC
TTCGGGCCTCACGCCATCGGATGAACCCAGATGGGATTAGCTAGTAGGTGGGGTAATGG
CTCACCTAGGCGACGATCCCTAGCTGGTCTGAGAGGATGACCAGCCACACTGGAACTG
AGACACGGTCCAGACTCCTACGGGAGGCAGCAGTGGGGAATATTGCACAATGGGCGCA
AGCCTGATGCAGCCATGCCGCGTGTGTGAAGAAGGCCTTCGGGTTGTAAAGCACTTTCA
GCGAGGAGGAAGGCATTGTGGTTAATAACCACAGTGATTGACGTTACTCGCAGAAGAA
GCACCGGCTAACTCCGTGCCAGCAGCCGCGGTAATACGGAGGGTGCAAGCGTTAATCG
GAATTACTGGGCGTAAAGCGCACGCAGGCGGTTTGTTAAGTCAGATGTGAAATCCCCGC
GCTTAACGTGGGAACTGCATTTGAAACTGGCAAGCTAGAGTCTTGTAGAGGGGGGTAG
AATTCCAGGTGTAGCGGTGAAATGCGTAGAGATCTGGAGGAATACCGGTGGCGAAGGC

GGCCCCCTGGACAAAGACTGACGCTCAGGTGCGAAAGCGTGGGGAGCAAACAGGATT
AGATACCCTGGTAGTCCACGCTGTAAACGATGTCGACTTGGAGGTTGTGCCCTTGAGGC
GTGGCTTCCGGAGCTAACGCGTTAAGTCGACCGCCTGGGGAGTACGGCCGCAAGGTTA
AAACTCAAATGAATTGACGGGGGCCCGCACAAGCGGTGGAGCATGTGGTTTAATTCGAT
GCAACGCGAAGAACCTTACCTACTCTTGACATCCACAGAACTTAGCAGAGATGCTTCGG
TGCCTTCGGGAACTGTGAGACAGGTGCTGCATGGCTGTCGTCAGCTCGTGTTGTGAAAT
GTTGGGTTAAGTCCCGCAACGAGCGCAACCCTTATCCTTTGTTGCCAGCACGTAATGGT
GGGAACTCAAGGGAGACTGCCGGTGACAAACCGGAGGAAGGTGGGGATGACGTCAAG
TCATCATGGCCCTTACGAGTAGGGCTACACACGTGCTACAATGGCAGATACAAAGTGAA
GCGAACTCGCGAGAGCAAGCGGACCACATAAAGTCTGTCGTAGTCCGGATTGGAGTCT
GCAACTCGACTCCATGAAGTCGGAATCGCTAGTAATCGTAGATCAGAATGCTACGGTGA
ATACGTTCCCGGGCCTTGTACACACCGCCCGTCACACCATGGGAGTGGGTTGCAAAAG
AAGTAGGTAGCTTAACCTTCGGGAGGGCGCTTACCACTTTGTGATTCATGACTGGGG
pattern 344
CGCTGGCGGCAGGCCTAACACATGCAAGTCGAGCGGCAGCGGAAAGTAGCTTGCTACT
TTGCCGGCGAGCGGCGGACGGGTGAGTAATGTCTGGGGATCTGCCTGATGGAGGGGGA
TAACTACTGGAAACGGTAGCTAATACCGCATGACCTCGAAAGAGCAAAGTGGGGGACC
TTCGGGCCTCACGCCATCGGATGAACCCAGATGGGATTAGCTAGTAGGTGGGGTAATGG
CTCACCTAGGCGACGATCCCTAGCTGGTCTGAGAGGATGACCAGCCACACTGGAACTG
AGACACGGTCCAGACTCCTACGGGAGGCAGCAGTGGGGAATATTGCACAATGGGCGCA
AGCCTGATGCGGCCATGCCGCGTGTGTGAAGAAGGCCTTCGGGTTGTAAAGCACTTTCA
GCGAGGAGGAAGGCATTGTGGTTAATAACCGCAGTGATTGACGTTACTCGCAGAAGAA
GCACCGGCTAACTCCGTGCCAGCAGCCGCGGTAATACGGAGGGTGCAAGCGTTAATCG
GAATTACTGGGCGTAAAGCGCACGCAGGCGGTTTGTTAAGTCAGATGTGAAATCCCCGC
GCTTAACGTGGGAACTGCATTTGAAACTGGCAAGCTAGAGTCTTGTAGAGGGGGGTAG
AATTCCAGGTGTAGCGGTGAAATGCGTAGAGATCTGGAGGAATACCGGTGGCGAAGGC
GGCCCCCTGGACAAAGACTGACGCTCAGGTGCGAAAGCGTGGGGAGCAAACAGGATT
AGATACCCTGGTAGTCCACGCTGTAAACGATGTCGACTTGGAGGTTGTGCCCTTGAGGC
GTGGCTTCCGGAGCTAACGCGTTAAGTCGACCGCCTGGGGAGTACGGCCGCAAGGTTA
AAACTCAAATGAATTGACGGGGGCCCGCACAAGCGGTGGAGCATGTGGTTTAATTCGAT
GCAACGCGAAGAACCTTACCTACTCTTGACATCCACGGAATTTAGCAGAGATGCTTTAG
TGCCTTCGGGAACCGTGAGACAGGTGCTGCATGGCTGTCGTCAGCTCGTGTTGTGAAAT
GTTGGGTTAAGTCCCGCAACGAGCGCAACCCTTATCCTTTGTTGCCAGCACGTAATGGT
GGGAACTCAAGGGAGACTGCCGGTGACAAACCGGAGGAAGGTGGGGATGACGTCAAG
TCATCATGGCCCTTACGAGTAGGGCTACACACGTGCTACAATGGCAGATACAAAGTGAA
GCGAACTCGCGAGAGCAAGCGGACCACATAAAGTCTGTCGTAGTCCGGATTGGAGTCT
GCAACTCGACTCCATGAAGTCGGAATCGCTAGTAATCGTAGATCAGAATGCTACGGTGA
ATACGTTCCCGGGCCTTGTACACACCGCCCGTCACACCATGGGAGTGGGTTGCAAAAG
AAGTAGGTAGCTTAACCTTCGGGAGGGCGCTTACCACTTTGTGATTCATGACTGGGG
pattern 345
CGCTGGCGGCAGGCCTAACACATGCAAGTCGAGCGGCAGCGGGAAGTAGTTTACTACT
TTGCCGGCGAGCGGCGGACGGGTGAGTAATGTCTGGGAAACTGCCTGATGGAGGGGGA
TAACTACTGGAAACGGTAGCTAATACCGCATGACCTCGCAAGAGCAAAGTGGGGGACC
TTCGGGCCTCACGCCATCGGATGTGCCCAGATGGGATTAGCTAGTAGGTGGGGTAATGG

CTCACCTAGGCGACGATCCCTAGCTGGTCTGAGAGGATGACCAGCCACACTGGAACTG
AGACACGGTCCAGACTCCTACGGGAGGCAGCAGTGGGGAATATTGCACAATGGGCGCA
AGCCTGATGCAGCCATGCCGCGTGTGTGAGGAAGGCCTTCGGGTTGTAAAGCACTTTCA
GCGAGGAGGAAGGCAGTCGTGTTAATAGCACGGTTGATTGACGTTACTCGCAGAAGAA
GCACCGGCTAACTCCGTGCCAGCAGCCGCGGTAATACGGAGGGTGCAAGCGTTAATCG
GAATTACTGGGCGTAAAGCGCACGCAGGCGGTTTGTTAAGTCAGATGTGAAATCCCCGC
GCTTAACGTGGGAACTGCATTTGAAACTGGCAAGCTAGAGTCTTGTAGAGGGGGGTAG
AATTCCAGGTGTAGCGGTGAAATGCGTAGAGATCTGGAGGAATACCGGTGGCGAAGGC
GGCCCCCTGGACAAAGACTGACGCTCAGGTGCGAAAGCGTGGGGAGCAAACAGGATT
AGATACCCTGGTAGTCCACGCTGTAAACGATGTCGACTTGGAGGTTGTGCCCTTGAGGC
GTGGCTTCCGGAGCTAACGCGTTAAGTCGACCGCCTGGGGAGTACGGCCGCAAGGTTA
AAACTCAAATGAATTGACGGGGGCCCGCACAAGCGGTGGAGCATGTGGTTTAATTCGAT
GCAACGCGAAGAACCTTACCTACTCTTGACATCCACAGAACTTAGCAGAGATGCTTCGG
TGCCTTCGGGAACTGTGAGACAGGTGCTGCATGGCTGTCGTCAGCTCGTGTTGTGAAAT
GTTGGGTTAAGTCCCGCAACGAGCGCAACCCTTATCCTTTGTTGCCAGCACGTAATGGT
GGGAACTCAAGGGAGACTGCCGGTGACAAACCGGAGGAAGGTGGGGATGACGTCAAG
TCATCATGGCCCTTACGAGTAGGGCTACACACGTGCTACAATGGCAGATACAAAGTGAA
GCGAACTCGCGAGAGCAAGCGGACCACATAAAGTCTGTCGTAGTCCGGATTGGAGTCT
GCAACTCGACTCCATGAAGTCGGAATCGCTAGTAATCGTAGATCAGAATGCTACGGTGA
ATACGTTCCCGGGCCTTGTACACACCGCCCGTCACACCATGGGAGTGGGTTGCAAAAG
AAGTAGGTAGCTTAACCTTCGGGAGGGCGCTTACCACTTTGTGATTCATGACTGGGG
pattern 346
CGCTGGCGGCAGGCCTAACACATGCAAGTCGAGCGGCAGCGGGAAGTAGTTTACTACT
TTGCCGGCGAGCGGCGGACGGGTGAGTAATGTCTGGGAAACTGCCTGATGGAGGGGGA
TAACTACTGGAAACGGTAGCTAATACCGCATGACCTCGCAAGAGCAAAGTGGGGGACC
TTCGGGCCTCACGCCATCGGATGTGCCCAGATGGGATTAGCTAGTAGGTGGGGTAATGG
CTCACCTAGGCTACGATCCCTAGCTGGTCTGAGAGGATGACCAGCCACACTGGAACTGA
GACACGGTCCAGACTCCTACGGGAGGCAGCAGTGGGGAATATTGCACAATGGGCGCAA
GCCTGATGCAGCCATGCCGCGTGTGTGAAGAAGGCCTTCGGGTTGTAAAGCACTTTCAG
CGAGGAGGAAGGCAGTCGTGTTAATAGCACGATTGATTGACGTTACTCGCAGAAGAAG
CACCGGCTAACTCCGTGCCAGCAGCCGCGGTAATACGGAGGGTGCAAGCGTTAATCGG
AATTACTGGGCGTAAAGCGCACGCAGGCGGTTTGTTAAGTCAGATGTGAAATCCCCGCG
CTTAACGTGGGAACTGCATTTGAAACTGGCAAGCTAGAGTCTTGTAGAGGGGGGTAGA
ATTCCAGGTGTAGCGGTGAAATGCGTAGAGATCTGGAGGAATACCGGTGGCGAAGGCG
GCCCCCTGGACAAAGACTGACGCTCAGGTGCGAAAGCGTGGGGAGCAAACAGGATTA
GATACCCTGGTAGTCCACGCTGTAAACGATGTCGACTTGGAGGTTGTGCCCTTGAGGCG
TGGCTTCCGGAGCTAACGCGTTAAGTCGACCGCCTGGGGAGTACGGCCGCAAGGTTAA
AACTCAAATGAATTGACGGGGGCCCGCACAAGCGGTGGAGCATGTGGTTTAATTCGATG
CAACGCGAAGAACCTTACCTACTCTTGACATCCACAGAACTTAGCAGAGATGCTTCGGT
GCCTTCGGGAACTGTGAGACAGGTGCTGCATGGCTGTCGTCAGCTCGTGTTGTGAAATG
TTGGGTTAAGTCCCGCAACGAGCGCAACCCTTATCCTTTGTTGCCAGCACGTAATGGTG
GGAACTCAAGGGAGACTGCCGGTGACAAACCGGAGGAAGGTGGGGATGACGTCAAGT
CATCATGGCCCTTACGAGTAGGGCTACACACGTGCTACAATGGCAGATACAAAGTGAAG
CGAACTCGCGAGAGCAAGCGGACCACATAAAGTCTGTCGTAGTCCGGATTGGAGTCTG

CAACTCGACTCCATGAAGTCGGAATCGCTAGTAATCGTAGATCAGAATGCTACGGTGAAT
ACGTTCCCGGGCCTTGTACACACCGCCCGTCACACCATGGGAGTGGGTTGCAAAAGAA
GTAGGTAGCTTAACCTTCGGGAGGGCGCTTACCACTTTGTGATTCATGACTGGGG
pattern 347
CGCTGGCGGCAGGCCTAACACATGCAAGTCGAGCGGCAGCGGAAAGTAGCTTGCTACT
TTGCCGGCGAGCGGCGGACGGGTGAGTAATGTCTGGGGATCTGCCTGATGGAGGGGGA
TAACTACTGGAAACGGTAGCTAATACCGCATGACCTCGAAAGAGCAAAGTGGGGGACC
TTCGGGCCTCACGCCATCGGATGAACCCAGATGGGATTAGCTAGTAGGTGGGGTAATGG
CTCACCTAGGCGACGATCCCTAGCTGGTCTGAGAGGATGACCAGCCACACTGGAACTG
AGACACGGTCCAGACTCCTACGGGAGGCAGCAGTGGGGAATATTGCACAATGGGCGCA
AGCCTGATGCAGCCATGCCGCGTGTGTGAAGAAGGCCTTCGGGTTGTAAAGCACTTTCA
GCGAGGAGGAAGGCATTGTGGTTAATAACCACAGTGATTGACGTTACTCGCAGAAGAA
GCACCGGCTAACTCCGTGCCAGCAGCCGCGGTAATACGGAGGGTGCAAGCGTTAATCG
GAATTACTGGGCGTAAAGCGCACGCAGGCGGTTTGTTAAGTCAGATGTGAAATCCCCGC
GCTTAACGTGGGAACTGCATTTGAAACTGGCAAGCTAGAGTCTTGTAGAGGGGGGTAG
AATTCCAGGTGTAGCGGTGAAATGCGTAGAGATCTGGAGGAATACCGGTGGCGAAGGC
GGCCCCCTGGACAAAGACTGACGCTCAGGTGCGAAAGCGTGGGGAGCAAACAGGATT
AGATACCCTGGTAGTCCACGCTGTAAACGATGTCGACTTGGAGGTTGTGCCCTTGAGGC
GTGGCTTCCGGAGCTAACGCGTTAAGTCGACCGCCTGGGGAGTACGGCCGCAAGGTTA
AAACTCAAATGAATTGACGGGGGCCCGCACAAGCGGTGGAGCATGTGGTTTAATTCGAT
GCAACGCGAAGAACCTTACCTACTCTTGACATCCACGGAATTTAGCAGAGATGCTTTAG
TGCCTTCGGGAACCGTGAGACAGGTGCTGCATGGCTGTCGTCAGCTCGTGTTGTGAAAT
GTTGGGTTAAGTCCCGCAACGAGCGCAACCCTTATCCTTTGTTGCCAGCACGTAATGGT
GGGAACTCAAGGGAGACTGCCGGTGACAAACCGGAGGAAGGTGGGGATGACGTCAAG
TCATCATGGCCCTTACGAGTAGGGCTACACACGTGCTACAATGGCAGATACAAAGTGAA
GCGAACTCGCGAGAGCAAGCAGACCACATAAAGTCTGTCGTAGTCCGGATTGGAGTCT
GCAACTCGACTCCATGAAGTCGGAATCGCTAGTAATCGTAGATCAGAATGCTACGGTGA
ATACGTTCCCGGGCCTTGTACACACCGCCCGTCACACCATGGGAGTGGGTTGCAAAAG
AAGTAGGTAGCTTAACCTTCGGGAGGGCGTTTACCACTTTGTGATTCATGACTGGGG
pattern 348
CGCTGGCGGCAGGCCTAACACATGCAAGTCGAGCGGCAGCGGGAAGTAGCTTGCTACT
TTGCCGGCGAGCGGCGGACGGGTGAGTAATGTCTGGGAAACTGCCTGATGGAGGGGGA
TAACTACTGGAAACGGTAGCTAATACCGCATGACCTCGCAAGAGCAAAGTGGGGGACC
TTCGGGCCTCACGCCATCGGATGTGCCCAGATGGGATTAGCTAGTAGGTGGGGTAATGG
CTCACCTAGGCGACGATCCCTAGCTGGTCTGAGAGGATGACCAGCCACACTGGAACTG
AGACACGGTCCAGACTCCTACGGGAGGCAGCAGTGGGGAATATTGCACAATGGGCGCA
AGCCTGATGCAGCCATGCCGCGTGTGTGAAGAAGGCCTTCGGGTTGTAAAGCACTTTCA
GCGAGGAGGAAGGGGTTGAGTTTAATACGCTCAATCATTGACGTTACTCGCAGAAGAA
GCACCGGCTAACTCCGTGCCAGCAGCCGCGGTAATACGGAGGGTGCAAGCGTTAATCG
GAATTACTGGGCGTAAAGCGCACGCAGGCGGTTTGTTAAGTCAGATGTGAAATCCCCGC
GCTTAACGTGGGAACTGCATTTGAAACTGGCAAGCTAGAGTCTTGTAGAGGGGGGTAG
AATTCCAGGTGTAGCGGTGAAATGCGTAGAGATCTGGAGGAATACCGGTGGCGAAGGC
GGCCCCCTGGACAAAGACTGACGCTCAGGTGCGAAAGCGTGGGGAGCAAACAGGATT
AGATACCCTGGTAGTCCACGCTGTAAACGATGTCGACTTGGAGGTTGTGCCCTTGAGGC

GTGGCTTCCGGAGCTAACGCGTTAAGTCGACCGCCTGGGGAGTACGGCCGCAAGGTTA
AAACTCAAATGAATTGACGGGGGCCCGCACAAGCGGTGGAGCATGTGGTTTAATTCGAT
GCAACGCGAAGAACCTTACCTACTCTTGACATCCACAGAACTTAGCAGAGATGCTTAGG
TGCCTTCGGGAACTGTGAGACAGGTGCTGCATGGCTGTCGTCAGCTCGTGTTGTGAAAT
GTTGGGTTAAGTCCCGCAACGAGCGCAACCCTTATCCTTTGTTGCCAGCACGTAATGGT
GGGAACTCAAAGGAGACTGCCGGTGATAAACCGGAGGAAGGTGGGGATGACGTCAAG
TCATCATGGCCCTTACGAGTAGGGCTACACACGTGCTACAATGGCAGATACAAAGTGAA
GCGAACTCGCGAGAGCAAGCGGACCACATAAAGTCTGTCGTAGTCCGGATTGGAGTCT
GCAACTCGACTCCATGAAGTCGGAATCGCTAGTAATCGTAGATCAGAATGCTACGGTGA
ATACGTTCCCGGGCCTTGTACACACCGCCCGTCACACCATGGGAGTGGGTTGCAAAAG
AAGTAGGTAGCTTAACCTTCGGGAGGGCGCTTACCACTTTGTGATTCATGACTGGGG
pattern 349
CGCTGGCGGCAGGCCTAACACATGCAAGTCGAGCGGCAGCGGGAAGTAGCTTGCTACT
TTGCCGGCGAGCGGCGGACGGGTGAGTAATGTCTGGGAAACTGCCTGATGGAGGGGGA
TAACTACTGGAAACGGTAGCTAATACCGCATGACCTCGCAAGAGCAAAGTGGGGGACC
TTCGGGCCTCACGCCATCGGATGTGCCCAGATGGGATTAGCTAGTAGGTGGGGTAATGG
CTCACCTAGGCGACGATCCCTAGCTGGTCTGAGAGGATGACCAGCCACACTGGAACTG
AGACACGGTCCAGACTCCTACGGGAGGCAGCAGTGGGGAATATTGCACAATGGGCGCA
AGCCTGATGCAGCCATGCCGCGTGTGTGAAGAAGGCCTTCGGGTTGTAAAGCACTTTCA
GCGAGGAGGAAGGGGTTGAGTTTAATACGCTCAATCATTGACGTTACTCGCAGAAGAA
GCACCGGCTAACTCCGTGCCAGCAGCCGCGGTAATACGGAGGGTGCAAGCGTTAATCG
GAATTACTGGGCGTAAAGCGCACGCAGGCGGTTTGTTAAGTCAGATGTGAAATCCCCGC
GCTTAACGTGGGAACTGCATTTGAAACTGGCAAGCTAGAGTCTTGTAGAGGGGGGTAG
AATTCCAGGTGTAGCGGTGAAATGCGTAGAGATCTGGAGGAATACCGGTGGCGAAGGC
GGCCCCCTGGACAAAGACTGACGCTCAGGTGCGAAAGCGTGGGGAGCAAACAGGATT
AGATACCCTGGTAGTCCACGCTGTAAACGATGTCGACTTGGAGGTTGTGCCCTTGAGGC
GTGGCTTCCGGAGCTAACGCGTTAAGTCGACCGCCTGGGGAGTACGGCCGCAAGGTTA
AAACTCAAATGAATTGACGGGGGCCCGCACAAGCGGTGGAGCATGTGGTTTAATTCGAT
GCAACGCGAAGAACCTTACCTACTCTTGACATCCACAGAACTTAGCAGAGATGCTTCGG
TGCCTTCGGGAACTGTGAGACAGGTGCTGCATGGCTGTCGTCAGCTCGTGTTGTGAAAT
GTTGGGTTAAGTCCCGCAACGAGCGCAACCCTTATCCTTTGTTGCCAGCACGTAATGGT
GGGAACTCAAAGGAGACTGCCGGTGATAAACCGGAGGAAGGTGGGGATGACGTCAAG
TCATCATGGCCCTTACGAGTAGGGCTACACACGTGCTACAATGGCAGATACAAAGTGAA
GCGAACTCGCGAGAGCAAGCGGACCACATAAAGTCTGTCGTAGTCCGGATTGGAGTCT
GCAACTCGACTCCATGAAGTCGGAATCGCTAGTAATCGTAGATCAGAATGCTACGGTGA
ATACGTTCCCGGGCCTTGTACACACCGCCCGTCACACCATGGGAGTGGGTTGCAAAAG
AAGTAGGTAGCTTAACCTTCGGGAGGGCGCTTACCACTTTGTGATTCATGACTGGGG
pattern 350
CGCTGGCGGCAGGCCTAACACATGCAAGTCGAGCGGCAGCGGAAAGTAGCTTGCTACT
TTGCCGGCGAGCGGCGGACGGGTGAGTAATGTCTGGGAAACTGCCTGATGGAGGGGGA
TAACTACTGGAAACGGTAGCTAATACCGCATGACCTCGCAAGAGCAAAGTGGGGGACC
TTCGGGCCTCACGCCATCGGATGTGCCCAGATGGGATTAGCTAGTAGGTGGGGTAATGG
CTCACCTAGGCGACGATCCCTAGCTGGTCTGAGAGGATGACCAGCCACACTGGAACTG
AGACACGGTCCAGACTCCTACGGGAGGCAGCAGTGGGGAATATTGCACAATGGGCGCA

AGCCTGATGCAGCCATGCCGCGTGTGTGAAGAAGGCCTTCGGGTTGTAAAGCACTTTCA
GCGAGGAGGAAGGGGTTGAGTTTAATACGCTCAATCATTGACGTTACTCGCAGAAGAA
GCACCGGCTAACTCCGTGCCAGCAGCCGCGGTAATACGGAGGGTGCAAGCGTTAATCG
GAATTACTGGGCGTAAAGCGCACGCAGGCGGTTTGTTAAGTCAGATGTGAAATCCCCGC
GCTTAACGTGGGAACTGCATTTGAAACTGGCAAGCTAGAGTCTTGTAGAGGGGGGTAG
AATTCCAGGTGTAGCGGTGAAATGCGTAGAGATCTGGAGGAATACCGGTGGCGAAGGC
GGCCCCCTGGACAAAGACTGACGCTCAGGTGCGAAAGCGTGGGGAGCAAACAGGATT
AGATACCCTGGTAGTCCACGCTGTAAACGATGTCGACTTGGAGGTTGTGCCCTTGAGGC
GTGGCTTCCGGAGCTAACGCGTTAAGTCGACCGCCTGGGGAGTACGGCCGCAAGGTTA
AAACTCAAATGAATTGACGGGGGCCCGCACAAGCGGTGGAGCATGTGGTTTAATTCGAT
GCAACGCGAAGAACCTTACCTACTCTTGACATCCACAGAACTTAGCAGAGATGCTTAGG
TGCCTTCGGGAACTGTGAGACAGGTGCTGCATGGCTGTCGTCAGCTCGTGTTGTGAAAT
GTTGGGTTAAGTCCCGCAACGAGCGCAACCCTTATCCTTTGTTGCCAGCACGTAATGGT
GGGAACTCAAAGGAGACTGCCGGTGATAAACCGGAGGAAGGTGGGGATGACGTCAAG
TCATCATGGCCCTTACGAGTAGGGCTACACACGTGCTACAATGGCAGATACAAAGTGAA
GCGAACTCGCGAGAGCAAGCGGACCACATAAAGTCTGTCGTAGTCCGGATTGGAGTCT
GCAACTCGACTCCATGAAGTCGGAATCGCTAGTAATCGTAGATCAGAATGCTACGGTGA
ATACGTTCCCGGGCCTTGTACACACCGCCCGTCACACCATGGGAGTGGGTTGCAAAAG
AAGTAGGTAGCTTAACCTTCGGGAGGGCGCTTACCACTTTGTGATTCATGACTGGGG
pattern 351
CGCTGGCGGCAGGCCTAACACATGCAAGTCGAGCGGCAGCGGGAAGTAGCTTGCTACT
TTGCCGGCGAGCGGCGGACGGGTGAGTAATGTCTGGGAAACTGCCTGATGGAGGGGGA
TAACTACTGGAAACGGTAGCTAATACCGCATGACCTCGCAAGAGCAAAGTGGGGGACC
TTCGGGCCTCACGCCATCGGATGTGCCCAGATGGGATTAGCTAGTAGGTGGGGTAATGG
CTCACCTAGGCGACGATCCCTAGCTGGTCTGAGAGGATGACCAGCCACACTGGAACTG
AGACACGGTCCAGACTCCTACGGGAGGCAGCAGTGGGGAATATTGCACAATGGGCGCA
AGCCTGATGCAGCCATGCCGCGTGTGTGAAGAAGGCCTTCGGGTTGTAAAGCACTTTCA
GCGAGGAGGAAGGGGTTGAGTTTAATACGCTCAATCATTGACGTTACTCGCAGAAGAA
GCACCGGCTAACTCCGTGCCAGCAGCCGCGGTAATACGGAGGGTGCAAGCGTTAATCG
GAATTACTGGGCGTAAAGCGCACGCAGGCGGTTTGTTAAGTCAGATGTGAAATCCCCGC
GCTTAACGTGGGAACTGCATTTGAAACTGGCAAGCTAGAGTCTTGTAGAGGGGGGTAG
AATTCCAGGTGTAGCGGTGAAATGCGTAGAGATCTGGAGGAATACCGGTGGCGAAGGC
GGCCCCCTGGACAAAGACTGACGCTCAGGTGCGAAAGCGTGGGGAGCAAACAGGATT
AGATACCCTGGTAGTCCACGCTGTAAACGATGTCGACTTGGAGGTTGTGCCCTTGAGGC
GTGGCTTCCGGAGCTAACGCGTTAAGTCGACCGCCTGGGGAGTACGGCCGCAAGGTTA
AAACTCAAATGAATTGACGGGGGCCCGCACAAGCGGTGGAGCATGTGGTTTAATTCGAT
GCAACGCGAAGAACCTTACCTACTCTTGACATCCACGGAATTTAGCAGAGATGCTTTAG
TGCCTTCGGGAACCGTGAGACAGGTGCTGCATGGCTGTCGTCAGCTCGTGTTGTGAAAT
GTTGGGTTAAGTCCCGCAACGAGCGCAACCCTTATCCTTTGTTGCCAGCACGTAATGGT
GGGAACTCAAAGGAGACTGCCGGTGATAAACCGGAGGAAGGTGGGGATGACGTCAAG
TCATCATGGCCCTTACGAGTAGGGCTACACACGTGCTACAATGGCAGATACAAAGTGAA
GCGAACTCGCGAGAGCAAGCGGACCACATAAAGTCTGTCGTAGTCCGGATTGGAGTCT
GCAACTCGACTCCATGAAGTCGGAATCGCTAGTAATCGTAGATCAGAATGCTACGGTGA
ATACGTTCCCGGGCCTTGTACACACCGCCCGTCACACCATGGGAGTGGGTTGCAAAAG

AAGTAGGTAGCTTAACCTTCGGGAGGGCGCTTACCACTTTGTGATTCATGACTGGGG
pattern 352
CGCTGGCGGCAGGCCTAACACATGCAAGTCGAGCGGCAGCGGAAAGTAGCTTGCTACT
TTGCCGGCGAGCGGCGGACGGGTGAGTAATGTCTGGGGATCTGCCTGATGGAGGGGGA
TAACTACTGGAAACGGTAGCTAATACCGCATGACCTCGAAAGAGCAAAGTGGGGGACC
TTCGGGCCTCACGCCATCGGATGAACCCAGATGGGATTAGCTAGTAGGTGGGGTAATGG
CTCACCTAGGCGACGATCCCTAGCTGGTCTGAGAGGATGACCAGCCACACTGGAACTG
AGACACGGTCCAGACTCCTACGGGAGGCAGCAGTGGGGAATATTGCACAATGGGCGCA
AGCCTGATGCAGCCATGCCGCGTGTGTGAAGAAGGCCTTCGGGTTGTAAAGCACTTTCA
GCGAGGAGGAAGGCATTGTGGTTAATAACCGCAGTGATTGACGTTACTCGCAGAAGAA
GCACCGGCTAACTCCGTGCCAGCAGCCGCGGTAATACGGAGGGTGCAAGCGTTAATCG
GAATTACTGGGCGTAAAGCGCACGCAGGCGGTTTGTTAAGTCAGATGTGAAATCCCCGC
GCTTAACGTGGGAACTGCATTTGAAACTGGCAAGCTAGAGTCTTGTAGAGGGGGGTAG
AATTCCAGGTGTAGCGGTGAAATGCGTAGAGATCTGGAGGAATACCGGTGGCGAAGGC
GGCCCCCTGGACAAAGACTGACGCTCAGGTGCGAAAGCGTGGGGAGCAAACAGGATT
AGATACCCTGGTAGTCCACGCTGTAAACGATGTCGACTTGGAGGTTGTGCCCTTGAGGC
GTGGCTTCCGGAGCTAACGCGTTAAGTCGACCGCCTGGGGAGTACGGCCGCAAGGTTA
AAACTCAAATGAATTGACGGGGGCCCGCACAAGCGGTGGAGCATGTGGTTTAATTCGAT
GCAACGCGAAGAACCTTACCTACTCTTGACATCCACAGAACTTAGCAGAGATGCTTCGG
TGCCTTCGGGAACCGTGAGACAGGTGCTGCATGGCTGTCGTCAGCTCGTGTTGTGAAAT
GTTGGGTTAAGTCCCGCAACGAGCGCAACCCTTATCCTTTGTTGCCAGCACGTAATGGT
GGGAACTCAAGGGAGACTGCCGGTGACAAACCGGAGGAAGGTGGGGATGACGTCAAG
TCATCATGGCCCTTACGAGTAGGGCTACACACGTGCTACAATGGCAGATACAAAGTGAA
GCGAACTCGCGAGAGCAAGCGGACCACATAAAGTCTGTCGTAGTCCGGATTGGAGTCT
GCAACTCGACTCCATGAAGTCGGAATCGCTAGTAATCGTAGATCAGAATGCTACGGTGA
ATACGTTCCCGGGCCTTGTACACACCGCCCGTCACACCATGGGAGTGGGTTGCAAAAG
AAGTAGGTAGCTTAACCTTCGGGAGGGCGCTTACCACTTTGTGATTCATGACTGGGG
pattern 353
CGCTGGCGGCAGGCCTAACACATGCAAGTCGAGCGGCAGCGGAAAGTAGCTTGCTACT
TTGCCGGCGAGCGGCGGACGGGTGAGTAATGTCTGGGGATCTGCCTGATGGAGGGGGA
TAACTACTGGAAACGGTAGCTAATACCGCATGACCTCGAAAGAGCAAAGTGGGGGACC
TTCGGGCCTCACGCCATCGGATGAACCCAGATGGGATTAGCTAGTAGGTGGGGTAATGG
CTCACCTAGGCGACGATCCCTAGCTGGTCTGAGAGGATGACCAGCCACACTGGAACTG
AGACACGGTCCAGACTCCTACGGGAGGCAGCAGTGGGGAATATTGCACAATGGGCTCA
AGCCTGATGCAGCCATGCCGCGTGTGTGAAGAAGGCCTTCGGGTTGTAAAGCACTTTCA
GCGAGGAGGAAGGCATTGTGGTTAATAACCACAGTGATTGACGTTACTCGCAGAAGAA
GCACCGGCTAACTCCGTGCCAGCAGCCGCGGTAATACGGAGGGTGCAAGCGTTAATCG
GAATTACTGGGCGTAAAGCGCACGCAGGCGGTTTGTTAAGTCAGATGTGAAATCCCCGC
GCTTAACGTGGGAACTGCATTTGAAACTGGCAAGCTAGAGTCTTGTAGAGGGGGGTAG
AATTCCAGGTGTAGCGGTGAAATGCGTAGAGATCTGGAGGAATACCGGTGGCGAAGGC
GGCCCCCTGGACAAAGACTGACGCTCAGGTGCGAAAGCGTGGGGAGCAAACAGGATT
AGATACCCTGGTAGTCCACGCTGTAAACGATGTCGACTTGGAGGTTGTGCCCTTGAGGC
GTGGCTTCCGGAGCTAACGCGTTAAGTCGACCGCCTGGGGAGTACGGCCGCAAGGTTA
AAACTCAAATGAATTGACGGGGGCCCGCACAAGCGGTGGAGCATGTGGTTTAATTCGAT

GCAACGCGAAGAACCTTACCTACTCTTGACATCCACAGAACTTAGCAGAGATGCTTCGG
TGCCTTCGGGAACTGTGAGACAGGTGCTGCATGGCTGTCGTCAGCTCGTGTTGTGAAAT
GTTGGGTTAAGTCCCGCAACGAGCGCAACCCTTATCCTTTGTTGCCAGCACGTAATGGT
GGGAACTCAAGGGAGACTGCCGGTGACAAACCGGAGGAAGGTGGGGATGACGTCAAG
TCATCATGGCCCTTACGAGTAGGGCTACACACGTGCTACAATGGCAGATACAAAGTGAA
GCGAACTCGCGAGAGCAAGCGGACCACATAAAGTCTGTCGTAGTCCGGATTGGAGTCT
GCAACTCGACTCCATGAAGTCGGAATCGCTAGTAATCGTAGATCAGAATGCTACGGTGA
ATACGTTCCCGGGCCTTGTACACACCGCCCGTCACACCATGGGAGTGGGTTGCAAAAG
AAGTAGGTAGCTTAACCTTCGGGAGGGCGCTTACCACTTTGTGATTCATGACTGGGG
pattern 354
CGCTGGCGGCAGGCCTAACACATGCAAGTCGAGCGGCAGCGGAAAGTAGCTTGCTACT
TTGCCGGCGAGCGGCGGACGGGTGAGTAATGTCTGGGGATCTGCCTGATGGAGGGGGA
TAACTACTGGAAACGGTAGCTAATACCGCATGACCTCGAAAGAGCAAAGTGGGGGACC
TTCGGGCCTCACGCCATCGGATGAACCCAGATGGGATTAGCTAGTAGGTGGGGTAATGG
CTCACCTAGGCGACGATCCCTAGCTGGTCTGAGAGGATGACCAGCCACACTGGAACTG
AGACACGGTCCAGACTCCTACGGGAGGCAGCAGTGGGGAATATTGCACAATGGGCGCA
AGCCTGATGCAGCCATGCCGCGTGTGTGAAGAAGGCCTTCGGGTTGTAAAGCACTTTCA
GCGAGGAGGAAGGCATTGTGGTTAATAACCACAGTGATTGACGTTACTCGCAGAAGAA
GCACCGGCTAACTCCGTGCCAGCAGCCGCGGTAATACGGAGGGTGCAAGCGTTAATCG
GAATTACTGGGCGTAAAGCGCACGCAGGCGGTTTGTTAAGTCAGATGTGAAATCCCCGC
GCTTAACGTGGGAACTGCATTTGAAACTGGCAAGCTAGAGTCTTGTAGAGGGGGGTAG
AATTCCAGGTGTAGCGGTGAAATGCGTAGAGATCTGGAGGAATACCGGTGGCGAAGGC
GGCCCCCTGGACAAAGACTGACGCTCAGGTGCGAAAGCGTGGGGAGCAAACAGGATT
AGATACCCTGGTAGTCCACGCTGTAAACGATGTCGACTTGGAGGTTGTGCCCTTGAGGC
GTGGCTTCCGGAGCTAACGCGTTAAGTCGACCGCCTGGGGAGTACGGCCGCAAGGTTA
AAACTCAAATGAATTGACGGGGGCCCGCACAAGCGGTGGAGCATGTGGTTTAATTCGAT
GCAACGCGAAGAACCTTACCTACTCTTGACATCCACAGAACTTAGCAGAGATGCTTCGG
TGCCTTCGGGAACCGTGAGACAGGTGCTGCATGGCTGTCGTCAGCTCGTGTTGTGAAAT
GTTGGGTTAAGTCCCGCAACGAGCGCAACCCTTATCCTTTGTTGCCAGCACGTAATGGT
GGGAACTCAAGGGAGACTGCCGGTGACAAACCGGAGGAAGGTGGGGATGACGTCAAG
TCATCATGGCCCTTACGAGTAGGGCTACACACGTGCTACAATGGCAGATACAAAGTGAA
GCGAACTCGCGAGAGCAAGCGGACCACATAAAGTCTGTCGTAGTCCGGATTGGAGTCT
GCAACTCGACTCCATGAAGTCGGAATCGCTAGTAATCGTAGATCAGAATGCTACGGTGA
ATACGTTCCCGGGCCTTGTACACACCGCCCGTCACACCATGGGAGTGGGTTGCAAAAG
AAGTAGGTAGCTTAACCTTCGGGAGGGCGCTTACCACTTTGTGATTCATGACTGGGG
pattern 355
CGCTGGCGGCAGGCCTAACACATGCAAGTCGAGCGGCAGCGGGGAGTAGTTTACTACT
TTGCCGGCGAGCGGCGGACGGGTGAGTAATGTCTGGGAAACTGCCTGATGGAGGGGGA
TAACTACTGGAAACGGTAGCTAATACCGCATAACGTCTTCGGACCAAAGTGGGGGACCT
TCGGGCCTCACGCCATCGGATGTGCCCAGATGGGATTAGCTAGTAGGTGGGGTAATGGC
TCACCTAGGCGACGATCCCTAGCTGGTCTGAGAGGATGACCAGCCACACTGGAACTGA
GACACGGTCCAGACTCCTACGGGAGGCAGCAGTGGGGAATATTGCACAATGGGCGCAA
GCCTGATGCAGCCATGCCGCGTGTGTGAAGAAGGCCTTCGGGTTGTAAAGCACTTTCAG
CGAGGAGGAAGGCATAAAGGTTAATAACCTTTGTGATTGACGTTACTCGCAGAAGAAG

CACCGGCTAACTCCGTGCCAGCAGCCGCGGTAATACGGAGGGTGCAAGCGTTAATCGG
AATTACTGGGCGTAAAGCGCACGCAGGCGGTTTGTTAAGTCAGATGTGAAATCCCCGCG
CTTAACGTGGGAACTGCATTTGAAACTGGCAAGCTAGAGTCTTGTAGAGGGGGGTAGA
ATTCCAGGTGTAGCGGTGAAATGCGTAGAGATCTGGAGGAATACCGGTGGCGAAGGCG
GCCCCCTGGACAAAGACTGACGCTCAGGTGCGAAAGCGTGGGGAGCAAACAGGATTA
GATACCCTGGTAGTCCACGCTGTAAACGATGTCGACTTGGAGGTTGTGCCCTTGAGGCG
TGGCTTCCGGAGCTAACGCGTTAAGTCGACCGCCTGGGGAGTACGGCCGCAAGGTTAA
AACTCAAATGAATTGACGGGGGCCCGCACAAGCGGTGGAGCATGTGGTTTAATTCGATG
CAACGCGAAGAACCTTACCTACTCTTGACATCCACAGAACTTAGCAGAGATGCTTCGGT
GCCTTCGGGAACTGTGAGACAGGTGCTGCATGGCTGTCGTCAGCTCGTGTTGTGAAATG
TTGGGTTAAGTCCCGCAACGAGCGCAACCCTTATCCTTTGTTGCCAGCACGTCATGGTG
GGAACTCAAAGGAGACTGCCGGTGATAAACCGGAGGAAGGTGGGGATGACGTCAAGT
CATCATGGCCCTTACGAGTAGGGCTACACACGTGCTACAATGGCAGATACAAAGTGAAG
CGAACTCGCGAGAGCAAGCGGACCACATAAAGTCTGTCGTAGTCCGGATTGGAGTCTG
CAACTCGACTCCATGAAGTCGGAATCGCTAGTAATCGTAGATCAGAATGCTACGGTGAAT
ACGTTCCCGGGCCTTGTACACACCGCCCGTCACACCATGGGAGTGGGTTGCAAAAGAA
GTAGGTAGCTTAACCTTCGGGAGGGCGCTTACCACTTTGTGATTCATGACTGGGG
pattern 356
CGCTGGCGGCAGGCCTAACACATGCAAGTCGAGCGGCAGCGGGAAGTAGTTTACTACT
TTGCCGGCGAGCGGCGGACGGGTGAGTAATGTCTGGGAAACTGCCTGATGGAGGGGGA
TAACTACTGGAAACGGTAGCTAATACCGCATAACGTCTTCGGACCAAAGTGGGGGACCT
TCGGGCCTCACGCCATCGGATGTGCCCAGATGGGATTAGCTAGTAGGTGGGGTAATGGC
TCACCTAGGCGACGATCCCTAGCTGGTCTGAGAGGATGACCAGCCACACTGGAACTGA
GACACGGTCCAGACTCCTACGGGAGGCAGCAGTGGGGAATATTGCACAATGGGCGCAA
GCCTGATGCAGCCATGCCGCGTGTGTGAAGAAGGCCTTCGGGTTGTAAAGCACTTTCAG
CGAGGAGGAAGGCATAAAGGTTAATAACCTTTATGATTGACGTTACTCGCAGAAGAAGC
ACCGGCTAACTCCGTGCCAGCAGCCGCGGTAATACGGAGGGTGCAAGCGTTAATCGGA
ATTACTGGGCGTAAAGCGCACGCAGGCGGTTTGTTAAGTCAGATGTGAAATCCCCGCGC
TTAACGTGGGAACTGCATTTGAAACTGGCAAGCTAGAGTCTTGTAGAGGGGGGTAGAA
TTCCAGGTGTAGCGGTGAAATGCGTAGAGATCTGGAGGAATACCGGTGGCGAAGGCGG
CCCCCTGGACAAAGACTGACGCTCAGGTGCGAAAGCGTGGGGAGCAAACAGGATTAG
ATACCCTGGTAGTCCACGCTGTAAACGATGTCGACTTGGAGGTTGTGCCCTTGAGGCGT
GGCTTCCGGAGCTAACGCGTTAAGTCGACCGCCTGGGGAGTACGGCCGCAAGGTTAAA
ACTCAAATGAATTGACGGGGGCCCGCACAAGCGGTGGAGCATGTGGTTTAATTCGATGC
AACGCGAAGAACCTTACCTACTCTTGACATCCACAGAACTTAGCAGAGATGCTTCGGTG
CCTTCGGGAACTGTGAGACAGGTGCTGCATGGCTGTCGTCAGCTCGTGTTGTGAAATGT
TGGGTTAAGTCCCGCAACGAGCGCAACCCTTATCCTTTGTTGCCAGCACGTCATGGTGG
GAACTCAAAGGAGACTGCCGGTGATAAACCGGAGGAAGGTGGGGATGACGTCAAGTC
ATCATGGCCCTTACGAGTAGGGCTACACACGTGCTACAATGGCAGATACAAAGTGAAGC
GAACTCGCGAGAGCAAGCGGACCACATAAAGTCTGTCGTAGTCCGGATTGGAGTCTGC
AACTCGACTCCATGAAGTCGGAATCGCTAGTAATCGTAGATCAGAATGCTACGGTGAAT
ACGTTCCCGGGCCTTGTACACACCGCCCGTCACACCATGGGAGTGGGTTGCAAAAGAA
GTAGGTAGCTTAACCTTCGGGAGGGCGCTTACCACTTTGTGATTCATGACTGGGG
pattern 357

CGCTGGCGGCAGGCCTAACACATGCAAGTCGAGCGGCAGCGGGAAGTAGTTTACTACT
TTGCCGGCGAGCGGCGGACGGGTGAGTAATGTCTGGGAAACTGCCTGATGGAGGGGGA
TAACTACTGGAAACGGTAGCTAATACCGCATGACCTCGTAAGAGCAAAGTGGGGGACCT
TCGGGCCTCACGCCATCGGATGTGCCCAGATGGGATTAGCTAGTAGGTGGGGTAATGGC
TCACCTAGGCGACGATCCCTAGCTGGTCTGAGAGGATGACCAGCCACACTGGAACTGA
GACACGGTCCAGACTCCTACGGGAGGCAGCAGTGGGGAATATTGCACAATGGGCGCAA
GCCTGATGCAGCCATGCCGCGTGTGTGAAGAAGGCCTTCGGGTTGTAAAGCACTTTCAG
CGAGGAGGAAGGCCGATAACTTAATACGTTGTCGGATTGACGTTACTCGCAGAAGAAG
CACCGGCTAACTCCGTGCCAGCAGCCGCGGTAATACGGAGGGTGCAAGCGTTAATCGG
AATTACTGGGCGTAAAGCGCACGCAGGCGGTTTGTTAAGTCAGATGTGAAATCCCCGCG
CTTAACGTGGGAACTGCATTTGAAACTGGCAAGCTAGAGTCTTGTAGAGGGGGGTAGA
ATTCCAGGTGTAGCGGTGAAATGCGTAGAGATCTGGAGGAATACCGGTGGCGAAGGCG
GCCCCCTGGACAAAGACTGACGCTCAGGTGCGAAAGCGTGGGGAGCAAACAGGATTA
GATACCCTGGTAGTCCACGCTGTAAACGATGTCGACTTGGAGGTTGTGCCCTTGAGGCG
TGGCTTCCGGAGCTAACGCGTTAAGTCGACCGCCTGGGGAGTACGGCCGCAAGGTTAA
AACTCAAATGAATTGACGGGGGCCCGCACAAGCGGTGGAGCATGTGGTTTAATTCGATG
CAACGCGAAGAACCTTACCTACTCTTGACATCCACAGAACTTAGCAGAGATGCTTCGGT
GCCTTCGGGAACTGTGAGACAGGTGCTGCATGGCTGTCGTCAGCTCGTGTTGTGAAATG
TTGGGTTAAGTCCCGCAACGAGCGCAACCCTTATCCTTTGTTGCCAGCACGTAATGGTG
GGAACTCAAGGGAGACTGCCGGTGACAAACCGGAGGAAGGTGGGGATGACGTCAAGT
CATCATGGCCCTTACGAGTAGGGCTACACACGTGCTACAATGGCAGATACAAAGTGAAG
CGAACTCGCGAGAGCAAGCGGACCACATAAAGTCTGTCGTAGTCCGGATTGGAGTCTG
CAACTCGACTCCATGAAGTCGGAATCGCTAGTAATCGTAGATCAGAATGCTACGGTGAAT
ACGTTCCCGGGCCTTGTACACACCGCCCGTCACACCATGGGAGTGGGTTGCAAAAGAA
GTAGGTAGCTTAACCTTCGGGAGGGCGCTTACCACTTTGTGATTCATGACTGGGG
pattern 358
CGCTGGCGGCAGGCCTAACACATGCAAGTCGAGCGGCAGCGGGAAGTAGTTTACTACT
TTGCCGGCGAGCGGCGGACGGGTGAGTAATGTCTGGGAAACTGCCTGATGGAGGGGGA
TAACTACTGGAAACGGTAGCTAATACCGCATGACCTCGCAAGAGCAAAGTGGGGGACC
TTCGGGCCTCACGCCATTGGATGTGCCCAGATGGGATTAGCTAGTAGGTGGGGTAATGG
CTCACCTAGGCGACGATCCCTAGCTGGTCTGAGAGGATGACCAGCCACACTGGAACTG
AGACACGGTCCAGACTCCTACGGGAGGCAGCAGTGGGGAATATTGCACAATGGGCGCA
AGCCTGATGCAGCCATGCCGCGTGTGTGAAGAAGGCCTTCGGGTTGTAAAGCACTTTCA
GCGAGGAGGAAGGCCAATAGCTTAATACGCTGTTGGATTGACGTTACTCGCAGAAGAA
GCACCGGCTAACTCCGTGCCAGCAGCCGCGGTAATACGGAGGGTGCAAGCGTTAATCG
GAATTACTGGGCGTAAAGCGCACGCAGGCGGTTTGTTAAGTCAGATGTGAAATCCCCGC
GCTTAACGTGGGAACTGCATTTGAAACTGGCAAGCTAGAGTCTTGTAGAGGGGGGTAG
AATTCCAGGTGTAGCGGTGAAATGCGTAGAGATCTGGAGGAATACCGGTGGCGAAGGC
GGCCCCCTGGACAAAGACTGACGCTCAGGTGCGAAAGCGTGGGGAGCAAACAGGATT
AGATACCCTGGTAGTCCACGCTGTAAACGATGTCGACTTGGAGGTTGTGCCCTTGAGGC
GTGGCTTCCGGAGCTAACGCGTTAAGTCGACCGCCTGGGGAGTACGGCCGCAAGGTTA
AAACTCAAATGAATTGACGGGGGCCCGCACAAGCGGTGGAGCATGTGGTTTAATTCGAT
GCAACGCGAAGAACCTTACCTACTCTTGACATCCACAGAACTTAGCAGAGATGCTTCGG
TGCCTTCGGGAACTGTGAGACAGGTGCTGCATGGCTGTCGTCAGCTCGTGTTGTGAAAT

GTTGGGTTAAGTCCCGCAACGAGCGCAACCCTTATCCTTTGTTGCCAGCACGTAATGGT
GGGAACTCAAGGGAGACTGCCGGTGACAAACCGGAGGAAGGTGGGGATGACGTCAAG
TCATCATGGCCCTTACGAGTAGGGCTACACACGTGCTACAATGGCAGATACAAAGTGAA
GCGAACTCGCGAGAGCAAGCGGACCACATAAAGTCTGTCGTAGTCCGGATTGGAGTCT
GCAACTCGACTCCATGAAGTCGGAATCGCTAGTAATCGTAGATCAGAATGCTACGGTGA
ATACGTTCCCGGGCCTTGTACACACCGCCCGTCACACCATGGGAGTGGGTTGCAAAAG
AAGTAGGTAGCTTAACCTTCGGGAGGGCGCTTACCACTTTGTGATTCATGACTGGGG
pattern 359
CGCTGGCGGCAGGCCTAACACATGCAAGTCGAGCGGCAGCGGGAAGTAGTTTACTACT
TTGCCGGCGAGCGGCGGACGGGTGAGTAATGTCTGGGAAACTGCCTGATGGAGGGGGA
TAACTACTGGAAACGGTAGCTAATACCGCATGACCTCGCAAGAGCAAAGTGGGGGACC
TTCGGGCCTCACGCCATCGGATGTGCCCAGATGGGATTAGCTAGTAGGTGGGGTAATGG
CTCACCTAGGCGACGATCCCTAGCTGGTCTGAGAGGATGACCAGCCACACTGGAACTG
AGACACGGTCCAGACTCCTACGGGAGGCAGCAGTGGGGAATATTGCACAATGGGCGCA
AGCCTGATGCAGCCATGCCGCGTGTGTGAAGAAGGCCTTCGGGTTGTAAAGCACTTTCA
GCGAGGAGGAAGGCCGATAACTTAATACGTTGTCGGATTGACGTTACTCGCAGAAGAA
GCACCGGCTAACTCCGTGCCAGCAGCCGCGGTAATACGGAGGGTGCAAGCGTTAATCG
GAATTACTGGGCGTAAAGCGCACGCAGGCGGTTTGTTAAGTCAGATGTGAAATCCCCGC
GCTTAACGTGGGAACTGCATTTGAAACTGGCAAGCTAGAGTCTTGTAGAGGGGGGTAG
AATTCCAGGTGTAGCGGTGAAATGCGTAGAGATCTGGAGGAATACCGGTGGCGAAGGC
GGCCCCCTGGACAAAGACTGACGCTCAGGTGCGAAAGCGTGGGGAGCAAACAGGATT
AGATACCCTGGTAGTCCACGCTGTAAACGATGTCGACTTGGAGGTTGTGCCCTTGAGGC
GTGGCTTCCGGAGCTAACGCGTTAAGTCGACCGCCTGGGGAGTACGGCCGCAAGGTTA
AAACTCAAATGAATTGACGGGGGCCCGCACAAGCGGTGGAGCATGTGGTTTAATTCGAT
GCAACGCGAAGAACCTTACCTACTCTTGACATCCACAGAACTTAGCAGAGATGCTTCGG
TGCCTTCGGGAACTGTGAGACAGGTGCTGCATGGCTGTCGTCAGCTCGTGTTGTGAAAT
GTTGGGTTAAGTCCCGCAACGAGCGCAACCCTTATCCTTTGTTGCCAGCACGTAATGGT
GGGAACTCAAGGGAGACTGCCGGTGACAAACCGGAGGAAGGTGGGGATGACGTCAAG
TCATCATGGCCCTTACGAGTAGGGCTACACACGTGCTACAATGGCAGATACAAAGTGAA
GCGAACTCGCGAGAGCAAGCGGACCACATAAAGTCTGTCGTAGTCCGGATTGGAGTCT
GCAACTCGACTCCATGAAGTCGGAATCGCTAGTAATCGTAGATCAGAATGCTACGGTGA
ATACGTTCCCGGGCCTTGTACACACCGCCCGTCACACCATGGGAGTGGGTTGCAAAAG
AAGTAGGTAGCTTAACCTTCGGGAGGGCGCTTACCACTTTGTGATTCATGACTGGGG
pattern 360
CGCTGGCGGCAGGCCTAACACATGCAAGTCGAGCGGCAGCGGGAAGTAGTTTACTACT
TTGCCGGCGAGCGGCGGACGGGTGAGTAATGTCTGGGAAACTGCCTGATGGAGGGGGA
TAACTACTGGAAACGGTAGCTAATACCGCATGACCTCGCAAGAGCAAAGTGGGGGACC
TTCGGGCCTCACGCCATCGGATGTGCCCAGATGGGATTAGCTAGTAGGTGGGGTAATGG
CTCACCTAGGCGACGATCCCTAGCTGGTCTGAGAGGATGACCAGCCACACTGGAACTG
AGACACGGTCCAGACTCCTACGGGAGGCAGCAGTGGGGAATATTGCACAATGGGCGTA
AGCCTGATGCAGCCATGCCGCGTGTGTGAAGAAGGCCTTCGGGTTGTAAAGCACTTTCA
GCGAGGAGGAAGGCCAATAGCTTAATACGCTGTTGGATTGACGTTACTCGCAGAAGAA
GCACCGGCTAACTCCGTGCCAGCAGCCGCGGTAATACGGAGGGTGCAAGCGTTAATCG
GAATTACTGGGCGTAAAGCGCACGCAGGCGGTTTGTTAAGTCAGATGTGAAATCCCCGC

GCTTAACGTGGGAACTGCATTTGAAACTGGCAAGCTAGAGTCTTGTAGAGGGGGGTAG
AATTCCAGGTGTAGCGGTGAAATGCGTAGAGATCTGGAGGAATACCGGTGGCGAAGGC
GGCCCCCTGGACAAAGACTGACGCTCAGGTGCGAAAGCGTGGGGAGCAAACAGGATT
AGATACCCTGGTAGTCCACGCTGTAAACGATGTCGACTTGGAGGTTGTGCCCTTGAGGC
GTGGCTTCCGGAGCTAACGCGTTAAGTCGACCGCCTGGGGAGTACGGCCGCAAGGTTA
AAACTCAAATGAATTGACGGGGGCCCGCACAAGCGGTGGAGCATGTGGTTTAATTCGAT
GCAACGCGAAGAACCTTACCTACTCTTGACATCCACAGAACTTAGCAGAGATGCTTCGG
TGCCTTCGGGAACTGTGAGACAGGTGCTGCATGGCTGTCGTCAGCTCGTGTTGTGAAAT
GTTGGGTTAAGTCCCGCAACGAGCGCAACCCTTATCCTTTGTTGCCAGCACGTAATGGT
GGGAACTCAAGGGAGACTGCCGGTGACAAACCGGAGGAAGGTGGGGATGACGTCAAG
TCATCATGGCCCTTACGAGTAGGGCTACACACGTGCTACAATGGCAGATACAAAGTGAA
GCGAACTCGCGAGAGCAAGCGGACCACATAAAGTCTGTCGTAGTCCGGATTGGAGTCT
GCAACTCGACTCCATGAAGTCGGAATCGCTAGTAATCGTAGATCAGAATGCTACGGTGA
ATACGTTCCCGGGCCTTGTACACACCGCCCGTCACACCATGGGAGTGGGTTGCAAAAG
AAGTAGGTAGCTTAACCTTCGGGAGGGCGCTTACCACTTTGTGATTCATGACTGGGG
pattern 361
CGCTGGCGGCAGGCCTAACACATGCAAGTCGAGCGGCAGCGGAAAGTAGCTTGCTACT
TTGCCGGCGAGCGGCGGACGGGTGAGTAATGTCTGGGGATCTGCCTGATGGAGGGGGA
TAACTACTGGAAACGGTAGCTAATACCGCATGACCTCGAAAGAGCAAAGTGGGGGACC
TTCGGGCCTCACGCCATCGGATGAACCCAGATGGGATTAGCTAGTAGGTGGGGTAATGG
CTCACCTAGGCGACGATCCCTAGCTGGTCTGAGAGGATGACCAGCCACACTGGAACTG
AGACACGGTCCAGACTCCTACGGGAGGCAGCAGTGGGGAATATTGCACAATGGGCGCA
AGCCTGATGCAGCCATGCCGCGTGTGTGAAGAAGGCCTTCGGGTTGTAAAGCACTTTCA
GCGAGGAGGAAGGCATTGTGGTTAATAGCCACAGTGATTGACGTTACTCGCAGAAGAA
GCACCGGCTAACTCCGTGCCAGCAGCCGCGGTAATACGGAGGGTGCAAGCGTTAATCG
GAATTACTGGGCGTAAAGCGCACGCAGGCGGTTTGTTAAGTCAGATGTGAAATCCCCGC
GCTTAACGTGGGAACTGCATTTGAAACTGGCAAGCTAGAGTCTTGTAGAGGGGGGTAG
AATTCCAGGTGTAGCGGTGAAATGCGTAGAGATCTGGAGGAATACCGGTGGCGAAGGC
GGCCCCCTGGACAAAGACTGACGCTCAGGTGCGAAAGCGTGGGGAGCAAACAGGATT
AGATACCCTGGTAGTCCACGCTGTAAACGATGTCGACTTGGAGGTTGTGCCCTTGAGGC
GTGGCTTCCGGAGCTAACGCGTTAAGTCGACCGCCTGGGGAGTACGGCCGCAAGGTTA
AAACTCAAATGAATTGACGGGGGCCCGCACAAGCGGTGGAGCATGTGGTTTAATTCGAT
GCAACGCGAAGAACCTTACCTACTCTTGACATCCACAGAACTTAGCAGAGATGCTTCGG
TGCCTTCGGGAACTGTGAGACAGGTGCTGCATGGCTGTCGTCAGCTCGTGTTGTGAAAT
GTTGGGTTAAGTCCCGCAACGAGCGCAACCCTTATCCTTTGTTGCCAGCACGTAATGGT
GGGAACTCAAGGGAGACTGCCGGTGACAAACCGGAGGAAGGTGGGGATGACGTCAAG
TCATCATGGCCCTTACGAGTAGGGCTACACACGTGCTACAATGGCAGATACAAAGTGAA
GCGAACTCGCGAGAGCAAGCGGACCACATAAAGTCTGTCGTAGTCCGGATTGGAGTCT
GCAACTCGACTCCATGAAGTCGGAATCGCTAGTAATCGTAGATCAGAATGCTACGGTGA
ATACGTTCCCGGGCCTTGTACACACCGCCCGTCACACCATGGGAGTGGGTTGCAAAAG
AAGTAGGTAGCTTAACCTTCGGGAGGGCGCTTACCACTTTGTGATTCATGACTGGGG
pattern 362
CGCTGGCGGCAGGCCTAACACATGCAAGTCGAGCGGCAGCGGGAAGTAGTTTACTACT
TTGCCGGCGAGCGGCGGACGGGTGAGTAATGTCTGGGAAACTGCCTGATGGAGGGGGA

TAACTACTGGAAACGGTAGCTAATACCGCATGACCTCGAAAGAGCAAAGTGGGGGACC
TTCGGGCCTCACGCCATCGGATGTGCCCAGATGGGATTAGCTAGTAGGTGGGGTAACGG
CTCACCTAGGCGACGATCCCTAGCTGGTCTGAGAGGATGACCAGCCACACTGGAACTG
AGACACGGTCCAGACTCCTACGGGAGGCAGCAGTGGGGAATATTGCACAATGGGCGCA
AGCCTGATGCAGCCATGCCGCGTGTGTGAAGAAGGCCTTCGGGTTGTAAAGCACTTTCA
GCGAGGAGGAAGGCAGTCGTGTTAATAGCACGATTGATTGACGTTACTCGCAGAAGAA
GCACCGGCTAACTCCGTGCCAGCAGCCGCGGTAATACGGAGGGTGCAAGCGTTAATCG
GAATTACTGGGCGTAAAGCGCACGCAGGCGGTTTGTTAAGTCAGATGTGAAATCCCCGC
GCTTAACGTGGGAACTGCATTTGAAACTGGCAAGCTAGAGTCTTGTAGAGGGGGGTAG
AATTCCAGGTGTAGCGGTGAAATGCGTAGAGATCTGGAGGAATACCGGTGGCGAAGGC
GGCCCCCTGGACAAAGACTGACGCTCAGGTGCGAAAGCGTGGGGAGCAAACAGGATT
AGATACCCTGGTAGTCCACGCTGTAAACGATGTCGACTTGGAGGTTGTGCCCTTGAGGC
GTGGCTTCCGGAGCTAACGCGTTAAGTCGACCGCCTGGGGAGTACGGCCGCAAGGTTA
AAACTCAAATGAATTGACGGGGGCCCGCACAAGCGGTGGAGCATGTGGTTTAATTCGAT
GCAACGCGAAGAACCTTACCTACTCTTGACATCCACAGAACTTAGCAGAGATGCTTCGG
TGCCTTCGGGAACTGTGAGACAGGTGCTGCATGGCTGTCGTCAGCTCGTGTTGTGAAAT
GTTGGGTTAAGTCCCGCAACGAGCGCAACCCTTATCCTTTGTTGCCAGCGAGTAATGTC
GGGAACTCAAAGGAGACTGCCGGTGATAAACCGGAGGAAGGTGGGGATGACGTCAAG
TCATCATGGCCCTTACGAGTAGGGCTACACACGTGCTACAATGGCAGATACAAAGTGAA
GCGAACTCGCGAGAGCAAGCGGACCACATAAAGTCTGTCGTAGTCCGGATTGGAGTCT
GCAACTCGACTCCATGAAGTCGGAATCGCTAGTAATCGTAGATCAGAATGCTACGGTGA
ATACGTTCCCGGGCCTTGTACACACCGCCCGTCACACCATGGGAGTGGGTTGCAAAAG
AAGTAGGTAGCTTAACCTTCGGGAGGGCGCTTACCACTTTGTGATTCATGACTGGGG
pattern 363
CGCTGGCGGCAGGCCTAACACATGCAAGTCTAGCGGCAGCGGAAAGTAGCTTGCTACT
TTGCCGGCGAGCGGCGGACGGGTGAGTAATGTCTGGGGATCTGCCTGATGGAGGGGGA
TAACTACTGGAAACGGTAGCTAATACCGCATGACCTCGAAAGAGCAAAGTGGGGGACC
TTCGGGCCTCACGCCATCGGATGAACCCAGATGGGATTAGCTAGTAGGTGGGGTAATGG
CTCACCTAGGCGACGATCCCTAGCTGGTCTGAGAGGATGACCAGCCACACTGGAACTG
AGACACGGTCCAGACTCCTACGGGAGGCAGCAGTGGGGAATATTGCACAATGGGCGCA
AGCCTGATGCAGCCATGCCGCGTGTGTGAAGAAGGCCTTCGGGTTGTAAAGCACTTTCA
GCGAGGAGGAAGGCATTGTGGTTAATAACCGCAGTGATTGACGTTACTCGCAGAAGAA
GCACCGGCTAACTCCGTGCCAGCAGCCGCGGTAATACGGAGGGTGCAAGCGTTAATCG
GAATTACTGGGCGTAAAGCGCACGCAGGCGGTTTGTTAAGTCAGATGTGAAATCCCCGC
GCTTAACGTGGGAACTGCATTTGAAACTGGCAAGCTAGAGTCTTGTAGAGGGGGGTAG
AATTCCAGGTGTAGCGGTGAAATGCGTAGAGATCTGGAGGAATACCGGTGGCGAAGGC
GGCCCCCTGGACAAAGACTGACGCTCAGGTGCGAAAGCGTGGGGAGCAAACAGGATT
AGATACCCTGGTAGTCCACGCTGTAAACGATGTCGACTTGGAGGTTGTGCCCTTGAGGC
GTGGCTTCCGGAGCTAACGCGTTAAGTCGACCGCCTGGGGAGTACGGCCGCAAGGTTA
AAACTCAAATGAATTGACGGGGGCCCGCACAAGCGGTGGAGCATGTGGTTTAATTCGAT
GCAACGCGAAGAACCTTACCTACTCTTGACATCCACAGAACTTAGCAGAGATGCTTCGG
TGCCTTCGGGAACTGTGAGACAGGTGCTGCATGGCTGTCGTCAGCTCGTGTTGTGAAAT
GTTGGGTTAAGTCCCGCAACGAGCGCAACCCTTATCCTTTGTTGCCAGCACGTAATGGT
GGGAACTCAAGGGAGACTGCCGGTGACAAACCGGAGGAAGGTGGGGATGACGTCAAG

TCATCATGGCCCTTACGAGTAGGGCTACACACGTGCTACAATGGCAGATACAAAGTGAA
GCGAACTCGCGAGAGCAAGCGGACCACATAAAGTCTGTCGTAGTCCGGATTGGAGTCT
GCAACTCGACTCCATGAAGTCGGAATCGCTAGTAATCGTAGATCAGAATGCTACGGTGA
ATACGTTCCCGGGCCTTGTACACACCGCCCGTCACACCATGGGAGTGGGTTGCAAAAG
AAGTAGGTAGCTTAACCTTCGGGAGGGCGCTTACCACTTTGTGATTCATGACTGGGG
pattern 364
CGCTGGCGGCAGGCCTAACACATGCAAGTCGAGCGGCAGCGGAAAGTAGCTTGCTACT
TTGCCGGCGAGCGGCGGACGGGTGAGTAATGTCTGGGAAACTGCCTGATGGAGGGGGA
TAACTACTGGAAACGGTAGCTAATACCGCATGACCTCGAAAGAGCAAAGTGGGGGACC
TTCGGGCCTCACGCCATCGGATGTGCCCAGATGGGATTAGCTAGTAGGTGAGGTAATGG
CTCACCTAGGCGACGATCCCTAGCTGGTCTGAGAGGATGACCAGCCACACTGGAACTG
AGACACGGTCCAGACTCCTACGGGAGGCAGCAGTGGGGAATATTGCACAATGGGCGCA
AGCCTGATGCAGCCATGCCGCGTGTGTGAAGAAGGCCTTCGGGTTGTAAAGCACTTTCA
GCGAGGAGGAAGGCATTTCACTTAATACGTGAGGTGATTGACGTTACTCGCAGAAGAA
GCACCGGCTAACTCCGTGCCAGCAGCCGCGGTAATACGGAGGGTGCAAGCGTTAATCG
GAATTACTGGGCGTAAAGCGCACGCAGGCGGTTTGTTAAGTCAGATGTGAAATCCCCGA
GCTTAACTTGGGAACTGCATTTGAAACTGGCAAGCTAGAGTCTTGTAGAGGGGGGTAG
AATTCCAGGTGTAGCGGTGAAATGCGTAGAGATCTGGAGGAATACCGGTGGCGAAGGC
GGCCCCCTGGACAAAGACTGACGCTCAGGTGCGAAAGCGTGGGGAGCAAACAGGATT
AGATACCCTGGTAGTCCACGCTGTAAACGATGTCGACTTGGAGGTTGTGCCCTTGAGGC
GTGGCTTCCGGAGCTAACGCGTTAAGTCGACCGCCTGGGGAGTACGGCCGCAAGGTTA
AAACTCAAATGAATTGACGGGGGCCCGCACAAGCGGTGGAGCATGTGGTTTAATTCGAT
GCAACGCGAAGAACCTTACCTACTCTTGACATCCACAGAACTGAGCAGAGATGCTTAG
GTGCCTTCGGGAACTGTGAGACAGGTGCTGCATGGCTGTCGTCAGCTCGTGTTGTGAAA
TGTTGGGTTAAGTCCCGCAACGAGCGCAACCCTTATCCTTTGTTGCCAGCACGTAATGG
TGGGAACTCAAAGGAGACTGCCGGTGATAAACCGGAGGAAGGTGGGGATGACGTCAA
GTCATCATGGCCCTTACGAGTAGGGCTACACACGTGCTACAATGGCAGATACAAAGTGA
AGCGAACTCGCGAGAGCAAGCGGACCACATAAAGTCTGTCGTAGTCCGGATTGGAGTC
TGCAACTCGACTCCATGAAGTCGGAATCGCTAGTAATCGTAGATCAGAATGCTACGGTG
AATACGTTCCCGGGCCTTGTACACACCGCCCGTCACACCATGGGAGTGGGTTGCAAAA
GAAGTAGGTAGCTTAACCTTCGGGAGGGCGCTTACCACTTTGTGATTCATGACTGGGG
pattern 365
CGCTGGCGGCAGGCCTAACACATGCAAGTCGAGCGGCAGCGGAAAGTAGCTTGCTACT
TTGCCGGCGAGCGGCGGACGGGTGAGTAATGTCTGGGGATCTGCCTGATGGAGGGGGA
TAACTACTGGAAACGGTAGCTAATACCGCATGACCTCGAAAGAGCAAAGTGGGGGACC
TTCGGGCCTCACGCCATCGGATGAACCCAGATGGGATTAGCTAGTAGGTGGGGTAATGG
CTCACCTAGGCGACGATCCCTAGCTGGTCTGAGAGGATGACCAGCCACACTGGAACTG
AGACACGGTCCAGACTCCTACGGGAGGCAGCAGTGGGGAATATTGCACAATGGGCGCA
AGCCTGATGCAGCCATGCCGCGTGTGTGAAGAAGGCCTTCGGGTTGTAAAGCACTTTCA
GCGAGGAGGAAGGCATTGTGGTTAATAACCGCAGTGATTGACGTTACTCGCAGAAGAA
GCACCGGCTAACTCCGTGCCAGCAGCCGCGGTAATACGGAGGGTGCAAGCGTTAATCG
GAATTACTGGGCGTAAAGCGCACGCAGGCGGTTTGTTAAGTCAGATGTGAAATCCCCGC
GCTTAACGTGGGAACTGCATTTGAAACTGGCAAGCTAGAGTCTTGTAGAGGGGGGTGG
AATTCCAGGTGTAGCGGTGAAATGCGTAGAGATCTGGAGGAATACCGGTGGCGAAGGC

GGCCCCCTGGACAAAGACTGACGCTCAGGTGCGAAAGCGTGGGGAGCAAACAGGATT
AGATACCCTGGTAGTCCACGCTGTAAACGATGTCGACTTGGAGGTTGTGCCCTTGAGGC
GTGGCTTCCGGAGCTAACGCGTTAAGTCGACCGCCTGGGGAGTACGGCCGCAAGGTTA
AAACTCAAATGAATTGACGGGGGCCCGCACAAGCGGTGGAGCATGTGGTTTAATTCGAT
GCAACGCGAAGAACCTTACCTACTCTTGACATCCACGGAATTTAGCAGAGATGCTTCGG
TGCCTTCGGGAACTGTGAGACAGGTGCTGCATGGCTGTCGTCAGCTCGTGTTGTGAAAT
GTTGGGTTAAGTCCCGCAACGAGCGCAACCCTTATCCTTTGTTGCCAGCACGTAATGGT
GGGAACTCAAGGGAGACTGCCGGTGACAAACCGGAGGAAGGTGGGGATGACGTCAAG
TCATCATGGCCCTTACGAGTAGGGCTACACACGTGCTACAATGGCAGATACAAAGTGAA
GCGAACTCGCGAGAGCAAGCGGACCACATAAAGTCTGTCGTAGTCCGGATTGGAGTCT
GCAACTCGACTCCATGAAGTCGGAATCGCTAGTAATCGTAGATCAGAATGCTACGGTGA
ATACGTTCCCGGGCCTTGTACACACCGCCCGTCACACCATGGGAGTGGGTTGCAAAAG
AAGTAGGTAGCTTAACCTTCGGGAGGGCGCTTACCACTTTGTGATTCATGACTGGGG
pattern 366
CGCTGGCGGCAGGCCTAACACATGCAAGTCGAGCGGCAGCGGGAAGTAGTTTACTACT
TTGCCGGCGAGCGGCGGACGGGTGAGTAATGTCTGGGAAACTGCCTGATGGAGGGGGA
TAACTACTGGAAACGGTAGCTAATACCGCATGACCTCGCAAGAGCAAAGTGGGGGACC
TTCGGGCCTCACGCCATCGGATGTGCCCAGATGGGATTAGCTAGTAGGTGGGGTAATGG
CTCACCTAGGCGACGATCCCTAGCTGGTCTGAGAGGATGACCAGCCACACTGGAACTG
AGACACGGTCCAGACTCCTACGGGAGGCAGCAGTGGGGAATATTGCACAATGGGCGCA
AGCCTGATGCAGCCATGCCGCGTGTGTGAAGAAGGCCTTCGGGTTGTAAAGCACTTTCA
GCGAGGAGGAAGGCAGTCGTGTTAATAGCACGGTTGATTGACGTTACTCGCAGAAGAA
GCACCGGCTAACTCCGTGCCAGCAGCCGCGGTAATACGGAGGGTGCAAGCGTTAATCG
GAATTACTGGGCGTAAAGCGCACGCAGGCGGTTTGTTAAGTCAGATGTGAAATCCCCGC
GCTTAACGTGGGAACTGCATTTGAAACTGGCAAGCTAGAGTCTTGTAGAGGGGGGTAG
AATTCCAGGTGTAGCGGTGAAATGCGTAGAGATCTGGAGGAATACCGGTGGCGAAGGC
GGCCCCCTGGACAAAGACTGACGCTCAGGTGCGAAAGCGTGGGGAGCAAACAGGATT
AGATACCCTGGTAGTCCACGCTGTAAACGATGTCGACTTGGAGGTTGTGCCCTTGAGGC
GTGGCTTCCGGAGCTAACGCGTTAAGTCGACCGCCTGGGGAGTACGGCCGCAAGGTTA
AAACTCAAATGAATTGACGGGGGCCCGCACAAGCGGTGGAGCATGTGGTTTAATTCGAT
GCAACGCGAAGAACCTTACCTACTCTTGACATCCACAGAACTTAGCAGAGATGCTTCGG
TGCCTTCGGGAACTGTGAGACAGGTGCTGCATGGCTGTCGTCAGCTCGTGTTGTGAAAT
GTTGGGTTAAGTCCCGCAACGAGCGCAACCCTTATCCTTTGTTGCCAGCACGTAATGGT
GGGAACTCAAGGGAGACTGCCGGTGACAAATCGGAGGAAGGTGGGGATGACGTCAAG
TCATCATGGCCCTTACGAGTAGGGCTACACACGTGCTACAATGGCAGATACAAAGTGAA
GCGAACTCGCGAGAGCAAGCGGACCACATAAAGTCTGTCGTAGTCCGGATTGGAGTCT
GCAACTCGACTCCATGAAGTCGGAATCGCTAGTAATCGTAGATCAGAATGCTACGGTGA
ATACGTTCCCGGGCCTTGTACACACCGCCCGTCACACCATGGGAGTGGGTTGCAAAAG
AAGTAGGTAGCTTAACCTTCGGGAGGGCGCTTACCACTTTGTGATTCATGACTGGGG
pattern 367
CGCTGGCGGCAGGCCTAACACATGCAAGTCGAGCGGCAGCGGGAAGTAGTTTACTACT
TTGCCGGCGAGCGGCGGACGGGTGAGTAATGTCTGGGAAACTGCCTGATGGAGGGGGA
TAACTACTGGAAACGGTAGCTAATACCGCATGACCTCGCAAGAGCAAAGTGGGGGACC
TTCGGGCCTCACGCCATCGGATGTGCCCAGATGGGATTAGCTAGTAGGTGGGGTAATGG

CTCACCTAGGCGACGATCCCTAGCTGGTCTGAGAGGATGACCAGCCACACTGGAACTG
AGACACGGTCCAGACTCCTACGGGAGGCAGCAGTGGGGAATATTGCACAATGGGCGCA
AGCCTGATGCAGCCATGCCGCGTGTGTGAAGAAGGCCTTCGGGTTGTAAAGCACTTTCA
GCGAGGAGGAAGGCAGTCGTGTTAATAGCACGGTTGATTGACATTACTCGCAGAAGAA
GCACCGGCTAACTCCGTGCCAGCAGCCGCGGTAATACGGAGGGTGCAAGCGTTAATCG
GAATTACTGGGCGTAAAGCGCACGCAGGCGGTTTGTTAAGTCAGATGTGAAATCCCCGC
GCTTAACGTGGGAACTGCATTTGAAACTGGCAAGCTAGAGTCTTGTAGAGGGGGGTAG
AATTCCAGGTGTAGCGGTGAAATGCGTAGAGATCTGGAGGAATACCGGTGGCGAAGGC
GGCCCCCTGGACAAAGACTGACGCTCAGGTGCGAAAGCGTGGGGAGCAAACAGGATT
AGATACCCTGGTAGTCCACGCTGTAAACGATGTCGACTTGGAGGTTGTGCCCTTGAGGC
GTGGCTTCCGGAGCTAACGCGTTAAGTCGACCGCCTGGGGAGTACGGCCGCAAGGTTA
AAACTCAAATGAATTGACGGGGGCCCGCACAAGCGGTGGAGCATGTGGTTTAATTCGAT
GCAACGCGAAGAACCTTACCTACTCTTGACATCCACAGAACTTAGCAGAGATGCTTCGG
TGCCTTCGGGAACTGTGAGACAGGTGCTGCATGGCTGTCGTCAGCTCGTGTTGTGAAAT
GTTGGGTTAAGTCCCGCAACGAGCGCAACCCTTATCCTTCGTTGCCAGCACGTAATGGT
GGGAACTCAAGGGAGACTGCCGGTGACAAATCGGAGGAAGGTGGGGATGACGTCAAG
TCATCATGGCCCTTACGAGTAGGGCTACACACGTGCTACAATGGCAGATACAAAGTGAA
GCGAACTCGCGAGAGCAAGCGGACCACATAAAGTCTGTCGTAGTCCGGATTGGAGTCT
GCAACTCGACTCCATGAAGTCGGAATCGCTAGTAATCGTAGATCAGAATGCTACGGTGA
ATACGTTCCCGGGCCTTGTACACACCGCCCGTCACACCATGGGAGTGGGTTGCAAAAG
AAGTAGGTAGCTTAACCTTCGGGAGGGCGCTTACCACTTTGTGATTCATGACTGGGG
pattern 368
CGCTGGCGGCAGGCCTAACACATGCAAGTCGAGCGGCAGCGGGAAGTAGTTTACTACT
TTGCCGGCGAGCGGCGGACGGGTGAGTAATGTCTGGGAAACTGCCTGATGGAGGGGGA
TAACTACTGGAAACGGTAGCTAATACCGCATGACCTCGCAAGAGCAAAGTGGGGGACC
TTCGGGCCTCACGCCATCGGATGTGCCCAGATGGGATTAGCTAGTAGGTGGGGTAATGG
CTCACCTAGGCGACGATCCCTAGCTGGTCTGAGAGGATGACCAGCCACACTGGAACTG
AGACACGGTCCAGACTCCTACGGGAGGCAGCAGTGGGGAATATTGCACAATGGGCGCA
AGCCTGATGCAGCCATGCCGCGTGTGTGAAGAAGGCCTTCGGGTTGTAAAGCACTTTCA
GCGAGGAGGAAGGCAGTCGTGTTAATAGCACGGTTGATTGACATTACTCGCAGAAGAA
GCACCGGCTAACTCCGTGCCAGCAGCCGCGGTAATACGGAGGGTGCAAGCGTTAATCG
GAATTACTGGGCGTAAAGCGCACGCAGGCGGTTTGTTAAGTCAGATGTGAAATCCCCGC
GCTTAACGTGGGAACTGCATTTGAAACTGGCAAGCTAGAGTCTTGTAGAGGGGGGTAG
AATTCCAGGTGTAGCGGTGAAATGCGTAGAGATCTGGAGGAATACCGGTGGCGAAGGC
GGCCCCCTGGACAAAGACTGACGCTCAGGTGCGAAAGCGTGGGGAGCAAACAGGATT
AGATACCCTGGTAGTCCACGCTGTAAACGATGTCGACTTGGAGGTTGTGCCCTTGAGGC
GTGGCTTCCGGAGCTAACGCGTTAAGTCGACCGCCTGGGGAGTACGGCCGCAAGGTTA
AAACTCAAATGAATTGACGGGGGCCCGCACAAGCGGTGGAGCATGTGGTTTAATTCGAT
GCAACGCGAAGAACCTTACCTACTCTTGACATCCACAGAACTTAGCAGAGATGCTTCGG
TGCCTTCGGGAACTGTGAGACAGGTGCTGCATGGCTGTCGTCAGCTCGTGTTGTGAAAT
GTTGGGTTAAGTCCCGCAACGAGCGCAACCCTTATCCTTTGTTGCCAGCACGTAATGGT
GGGAACTCAAGGGAGACTGCCGGTGACAAACCGGAGGAAGGTGGGGATGACGTCAAG
TCATCATGGCCCTTACGAGTAGGGCTACACACGTGCTACAATGGCAGATACAAAGTGAA
GCGAACTCGCGAGAGCAAGCGGACCACATAAAGTCTGTCGTAGTCCGGATTGGAGTCT

GCAACTCGACTCCATGAAGTCGGAATCGCTAGTAATCGTAGATCAGAATGCTACGGTGA
ATACGTTCCCGGGCCTTGTACACACCGCCCGTCACACCATGGGAGTGGGTTGCAAAAG
AAGTAGGTAGCTTAACCTTCGGGAGGGCGCTTACCACTTTGTGATTCATGACTGGGG
pattern 369
CGCTGGCGGCAGGCCTAACACATGCAAGTCGAGCGGCAGCGGGAAGTAGTTTACTACT
TTGCCGGCGAGCGGCGGACGGGTGAGTAATGTCTGGGAAACTGCCTGATGGAGGGGGA
TAACTACTGGAAACGGTAGCTAATACCGCATAACGTCTTCGGACCAAAGTGGGGGACCT
TCGGGCCTCACGCCATCAGATGTGCCCAGATGGGATTAGCTAGTAGGTGGGGTAATGGC
TCACCTAGGCGACGATCCCTAGCTGGTCTGAGAGGATGACCAGCCACACTGGAACTGA
GACACGGTCCAGACTCCTACGGGAGGCAGCAGTGGGGAATATTGCACAATGGGCGCAA
GCCTGATGCAGCCATGCCGCGTGTGTGAAGAAGGCCTTCGGGTTGTAAAGCACTTTCAG
CGAGGAGGAAGGCATAAAGGTTAATAACCTTTGTGATTGACGTTACTCGCAGAAGAAG
CACCGGCTAACTCCGTGCCAGCAGCCGCGGTAATACGGAGGGTGCAAGCGTTAATCGG
AATTACTGGGCGTAAAGCGCACGCAGGCGGTTTGTTAAGTCAGATGTGAAATCCCCGCG
CTTAACGTGGGAACTGCATTTGAAACTGGCAAGCTAGAGTCTTGTAGAGGGGGGTAGA
ATTCCAGGTGTAGCGGTGAAATGCGTAGAGATCTGGAGGAATACCGGTGGCGAAGGCG
GCCCCCTGGACAAAGACTGACGCTCAGGTGCGAAAGCGTGGGGAGCAAACAGGATTA
GATACCCTGGTAGTCCACGCTGTAAACGATGTCGACTTGGAGGTTGTGCCCTTGAGGCG
TGGCTTCCGGAGCTAACGCGTTAAGTCGACCGCCTGGGGAGTACGGCCGCAAGGTTAA
AACTCAAATGAATTGACGGGGGCCCGCACCAGCGGTGGAGCATGTGGTTTAATTCGATG
CAACGCGAAGAACCTTACCTACTCTTGACATCCACGGAATTTAGCAGAGATGCTTTAGT
GCCTTCGGGAACCGTGAGACAGGTGCTGCATGGCTGTCGTCAGCTCGTGTTGTGAAATG
TTGGGTTAAGTCCCGCAACGAGCGCAACCCTTATCCTTTGTTGCCAGCACGTCATGGTG
GGAACTCAAAGGAGACTGCCGGTGATAAACCGGAGGAAGGTGGGGATGACGTCAAGT
CATCATGGCCCTTACGAGTAGGGCTACACACGTGCTACAATGGCAGATACAAAGTGAAG
CGAACTCGCGAGAGCAAGCGGACCACATAAAGTCTGTCGTAGTCCGGATTGGAGTCTG
CAACTCGACTCCATGAAGTCGGAATCGCTAGTAATCGTAGATCAGAATGCTACGGTGAAT
ACGTTCCCGGGCCTTGTACACACCGCCCGTCACACCATGGGAGTGGGTTGCAAAAGAA
GTAGGTAGCTTAACCTTCGGGAGGGCGCTTACCACTTTGTGATTCATGACTGGGG
pattern 370
CGCTGGCGGCAGGCCTAACACATGCAAGTCGAGCGGCAGCGGGAAGTAGTTTACTACT
TTGCCGGCGAGCGGCGGACGGGTGAGTAATGTCTGGGAAACTGCCTGATGGAGGGGGA
TAACTACTGGAAACGGTAGCTAATACCGCATAACGTCTTCGGACCAAAGTGGGGGACCT
TCGGGCCTCACGCCATCGGATGTGCCCAGATGGGATTAGCTAGTAGGTGGGGTAATGGC
TCACCTAGGCGACGATCCCTAGCTGGTCTGAGAGGATGACCAGCCACACTGGAACTGA
GACACGGTCCAGACTCCTACGGGAGGCAGCAGTGGGGAATATTGCACAATGGGCGCAA
GCCTGATGCAGCCATGCCGCGTGTGTGAAGAAGGCCTTCGGGTTGTAAAGCACTTTCAG
CGAGGAGGAAGGCATAAAGGTTAATAACCTTTGTGATTGACGTTACTCGCAGAAGAAG
CACCGGCTAACTCCGTGCCAGCAGCCGCGGTAATACGGAGGGTGCAAGCGTTAATCGG
AATTACTGGGCGTAAAGCGCACGCAGGCGGTTTGTTAAGTCAGATGTGAAATCCCCGCG
CTTAACGTGGGAACTGCATTTGAAACTGGCAAGCTAGAGTCTTGTAGAGGGGGGTAGA
ATTCCAGGTGTAGCGGTGAAATGCGTAGAGATCTGGAGGAATACCGGTGGCGAAGGCG
GCCCCCTGGACAAAGACTGACGCTCAGGTGCGAAAGCGTGGGGAGCAAACAGGATTA
GATACCCTGGTAGTCCACGCTGTAAACGATGTCGACTTGGAGGTTGTGCCCTTGAGGCG

TGGCTTCCGGAGCTAACGCGTTAAGTCGACCGCCTGGGGAGTACGGCCGCAAGGTTAA
AACTCAAATGAATTGACGGGGGCCCGCACAAGCGGTGGAGCATGTGGTTTAATTCGATG
CAACGCGAAGAACCTTACCTACTCTTGACATCCACGGAATTTAGCAGAGATGCTTTAGT
GCCTTCGGGAACTGTGAGACAGGTGCTGCATGGCTGTCGTCAGCTCGTGTTGTGAAATG
TTGGGTTAAGTCCCGCAACGAGCGCAACCCTTATCCTTTGTTGCCAGCACGTGATGGTG
GGAACTCAAAGGAGACTGCCGGTGATAAACCGGAGGAAGGTGGGGATGACGTCAAGT
CATCATGGCCCTTACGAGTAGGGCTACACACGTGCTACAATGGCAGATACAAAGTGAAG
CGAACTCGCGAGAGCAAGCGGACCACATAAAGTCTGTCGTAGTCCGGATTGGAGTCTG
CAACTCGACTCCATGAAGTCGGAATCGCTAGTAATCGTAGATCAGAATGCTACGGTGAAT
ACGTTCCCGGGCCTTGTACACACCGCCCGTCACACCATGGGAGTGGGTTGCAAAAGAA
GTAGGTAGCTTAACCTTCGGGAGGGCGCTTACCACTTTGTGATTCATGACTGGGG
pattern 371
CGCTGGCGGCAGGCCTAACACATGCAAGTCGAGCGGCAGCGGGAAGTAGTTTACTACT
TTGCCGGCGAGCGGCGGACGGGTGAGTAATGTCTGGGAAACTGCCTGATGGAGGGGGA
TAACTACTGGAAACGGTAGCTAATACCGCATGACCTCGTAAGAGCAAAGTGGGGGACCT
TCGGGCCTCACGCCATCGGATGTGCCCAGATGGGATTAGCTAGTAGGTGGGGTAATGGC
TCACCTAGGCGACGATCCCTAGCTGGTCTGAGAGGATGACCAGCCACACTGGAACTGA
GACACGGTCCAGACTCCTACGGGAGGCAGCAGTGGGGAATATTGCACAATGGGCGCAA
GCCTGATGCAGCCATGCCGCGTGTGTGAAGAAGGCCTTCGGGTTGTAAAGCACTTTCAG
CGAGGAGGAAGGCAGTCGTGTTAATAGCACGATTGATTGACGTTACTCGCAGAAGAAG
CACCGGCTAACTCCGTGCCAGCAGCCGCGGTAATACGGAGGGTGCAAGCGTTAATCGG
AATTACTGGGCGTAAAGCGCACGCAGGCGGTTTGTTAAGTCAGATGTGAAATCCCCGCG
CTTAACGTGGGAACTGCATTTGAAACTGGCAAGCTAGAGTCTTGTAGAGGGGGGTAGA
ATTCCAGGTGTAGCGGTGAAATGCGTAGAGATCTGGAGGAATACCGGTGGCGAAGGCG
GCCCCCTGGACAAAGACTGACGCTCAGGTGCGAAAGCGTGGGGAGCAAACAGGATTA
GATACCCTGGTAGTCCACGCTGTAAACGATGTCGACTTGGAGGTTGTGCCCTTGAGGCG
TGGCTTCCGGAGCTAACGCGTTAAGTCGACCGCCTGGGGAGTACGGCCGCAAGGTTAA
AACTCAAATGAATTGACGGGGGCCCGCACAAGCGGTGGAGCATGTGGTTTAATTCGATG
CAACGCGAAGAACCTTACCTACTCTTGACATCCACAGAACTTAGCAGAGATGCTTCAGT
GCCTTCGGGAACCGTGAGACAGGTGCTGCATGGCTGTCGTCAGCTCGTGTTGTGAAATG
TTGGGTTAAGTCCCGCAACGAGCGCAACCCTTATCCTTTGTTGCCAGCACGTAATGGTG
GGAACTCAAGGGAGACTGCCGGTGACAAACCGGAGGAAGGTGGGGATGACGTCAAGT
CATCATGGCCCTTACGAGTAGGGCTACACACGTGCTACAATGGCAGATACAAAGTGAAG
CGAACTCGCGAGAGCAAGCGGACCACATAAAGTCTGTCGTAGTCCGGATTGGAGTCTG
CAACTCGACTCCATGAAGTCGGAATCGCTAGTAATCGTAGATCAGAATGCTACGGTGAAT
ACGTTCCCGGGCCTTGTACACACCGCCCGTCACACCATGGGAGTGGGTTGCAAAAGAA
GTAGGTAGCTTAACCTTCGGGAGGGCGCTTACCACTTTGTGATTCATGACTGGGG
pattern 372
CGCTGGCGGCAGGCCTAACACATGCAAGTCGAGCGGCAGCGGGAAGTAGTTTACTACT
TTGCCGGCGAGCGGCGGACGGGTGAGTAATGTCTGGGGATCTGCCTGATGGAGGGGGA
TAACTACTGGAAACGGTGGCTAATACCGCATGACCTCGCAAGAGCAAAGTGGGGGACC
TTAGGGCCTCACGCCATCGGATGAACCCAGATGGGATTAGCTAGTAGGTGGGGTAATGG
CTCACCTAGGCGACGATCCCTAGCTGGTCTGAGAGGATGACCAGCCACACTGGAACTG
AGACACGGTCCAGACTCCTACGGGAGGCAGCAGTGGGGAATATTGCACAATGGGCGCA

AGCCTGATGCAGCCATGCCGCGTGTGTGAAGAAGGCCTTCGGGTTGTAAAGCACTTTCA
GCGAGGAGGAAGGGGTTGAGTTTAATACGCTCAATCATTGACGTTACTCGCAGAAGAA
GCACCGGCTAACTCCGTGCCAGCAGCCGCGGTAATACGGAGGGTGCAAGCGTTAATCG
GAATTACTGGGCGTAAAGCGCACGCAGGCGGTTTGTTAAGTCAGATGTGAAATCCCCGC
GCTTAACGTGGGAACTGCATTTGAAACTGGCAAGCTAGAGTCTTGTAGAGGGGGGTAG
AATTCCAGGTGTAGCGGTGAAATGCGTAGAGATCTGGAGGAATACCGGTGGCGAAGGC
GGCCCCCTGGACAAAGACTGACGCTCAGGTGCGAAAGCGTGGGGAGCAAACAGGATT
AGATACCCTGGTAGTCCACGCTGTAAACGATGTCGACTTGGAGGTTGTGCCCTTGAGGC
GTGGCTTCCGGAGCTAACGCGTTAAGTCGACCGCCTGGGGAGTACGGCCGCAAGGTTA
AAACTCAAATGAATTGACGGGGGCCCGCACAAGCGGTGGAGCATGTGGTTTAATTCGAT
GCAACGCGAAGAACCTTACCTACTCTTGACATCCACGGAATTTAGCAGAGATGCTTTAG
TGCCTTCGGGAACCGTGAGACAGGTGCTGCATGGCTGTCGTCAGCTCGTGTTGTGAAAT
GTTGGGTTAAGTCCCGCAACGAGCGCAACCCTTATCCTTTGTTGCCAGCACGTAATGGT
GGGAACTCAAGGGAGACTGCCGGTGACAAACCGGAGGAAGGTGGGGATGACGTCAAG
TCATCATGGCCCTTACGAGTAGGGCTACACACGTGCTACAATGGCAGATACAAAGTGAA
GCGAACTCGCGAGAGCCAGCGGACCACATAAAGTCTGTCGTAGTCCGGATTGGAGTCT
GCAACTCGACTCCATGAAGTCGGAATCGCTAGTAATCGTAGATCAGAATGCTACGGTGA
ATACGTTCCCGGGCCTTGTACACACCGCCCGTCACACCATGGGAGTGGGTTGCAAAAG
AAGTAGGTAGCTTAACCTTCGGGAGGGCGTTTACCACTTTGTGATTCATGACTGGGG
pattern 373
CGCTGGCGGCAGGCCTAACACATGCAAGTCGAGCGGCAGCGGGAAGTAGTTTACTACT
TTGCCGGCGAGCGGCGGACGGGTGAGTAATGTCTGGGAAACTGCCTGATGGAGGGGGA
TAACTACTGGAAACGGTAGCTAATACCGCATAACGTCTTCGGACCAAAGTGGGGGACCT
TCTGGCCTCACGCCATCGGATGTGCCCAGATGGGATTAGCTAGTAGGTGGGGTAATGGC
TCACCTAGGCGACGATCCCTAGCTGGTCTGAGAGGATGACCAGCCACACTGGAACTGA
GACACGGTCCAGACTCCTACGGGAGGCAGCAGTGGGGAATATTGCACAATGGGCGCAA
GCCTGATGCAGCCATGCCGCGTGTGTGAAGAAGGCCTTCGGGTTGTAAAGCACTTTCAG
CGAGGAGGAAGGCATAAAGGTTAATAACCTTTGTGATTGACGTTACTCGCAGAAGAAG
CACCGGCTAACTCCGTGCCAGCAGCCGCGGTAATACGGAGGGTGCAAGCGTTAATCGG
AATTACTGGGCGTAAAGCGCACGCAGGCGGTTTGTTAAGTCAGATGTGAAATCCCCGCG
CTTAACGTGGGAACTGCATTTGAAACTGGCAAGCTAGAGTCTTGTAGAGGGGGGTAGA
ATTCCAGGTGTAGCGGTGAAATGCGTAGAGATCTGGAGGAATACCGGTGGCGAAGGCG
GCCCCCTGGACAAAGACTGACGCTCAGGTGCGAAAGCGTGGGGAGCAAACAGGATTA
GATACCCTGGTAGTCCACGCTGTAAACGATGTCGACTTGGAGGTTGTGCCCTTGAGGCG
TGGCTTCCGGAGCTAACGCGTTAAGTCGACCGCCTGGGGAGTACGGCCGCAAGGTTAA
AACTCAAATGAATTGACGGGGGCCCGCACAAGCGGTGGAGCATGTGGTTTAATTCGATG
CAACGCGAAGAACCTTACCTACTCTTGACATCCACAGAACTTAGCAGAGATGCTTCGGT
GCCTTCGGGAACTGTGAGACAGGTGCTGCATGGCTGTCGTCAGCTCGTGTTGTGAAATG
TTGGGTTAAGTCCCGCAACGAGCGCAACCCTTATCCTTTGTTGCCAGCACGTGATGGTG
GGAACTCAAAGGAGACTGCCGGTGATAAACCGGAGGAAGGTGGGGATGACGTCAAGT
CATCATGGCCCTTACGAGTAGGGCTACACACGTGCTACAATGGCAGATACAAAGTGAAG
CGAACTCGCGAGAGCAAGCGGACCACATAAAGTCTGTCGTAGTCCGGATTGGAGTCTG
CAACTCGACTCCATGAAGTCGGAATCGCTAGTAATCGTAGATCAGAATGCTACGGTGAAT
ACGTTCCCGGGCCTTGTACACACCGCCCGTCACACCATGGGAGTGGGTTGCAAAAGAA

GTAGGTAGCTTAACCTTCGGGAGGGCGCTTACCACTTTGTGATTCATGACTGGGG
pattern 374
CGCTGGCGGCAGGCCTAACACATGCAAGTCGAGCGGCAGCGGGAAGTAGTTTACTACT
TTGCCGGCGAGCGGCGGACGGGTGAGTAATGTCTGGGAAACTGCCTGATGGAGGGGGA
TAACTACTGGAAACGGTAGCTAATACCGCATAACGTCTTCGGACCAAAGTGGGGGACCT
TCGGGCCTCACGCCATCGGATGTGCCCAGATGGGATTAGCTAGTAGGTGGGGTAATGGC
TCACCTAGGCGACGATCCCTAGCTGGTCTGAGAGGATGACCAGCCACACTGGAACTGA
GACACGGTCCAGACTCCTACGGGAGGCAGCAGTGGGGAATATTGCACAATGGGCGCAA
GCCTGATGCAGCCATGCCGCGTGTGTGAAGAAGGCCTTCGGGTTGTAAAGCACTTTCAG
CGAGGAGGAAGGCATAAAGGTTAATAACCTTTGTGATTGACGTTACTCGCAGAAGAAG
CACCGGCTAACTCCGTGCCAGCAGCCGCGGTAATACGGAGGGTGCAAGCGTTAATCGG
AATTACTGGGCGTAAAGCGCACGCAGGCGGTTTGTTAAGTCAGATGTGAAATCCCCGCG
CTTAACGTGGGAACTGCATTTGAAACTGGCAAGCTAGAGTCTTGTAGAGGGGGGTAGA
ATTCCAGGTGTAGCGGTGAAATGCGTAGAGATCTGGAGGAATACCGGTGGCGAAGGCG
GCCCCCTGGACAAAGACTGACGCTCAGGTGCGAAAGCGTGGGGAGCAAACAGGATTA
GATACCCTGGTAGTCCACGCTGTAAACGATGTCGACTTGGAGGTTGTGCCCTTGAGGCG
TGGCTTCCGGAGCTAACGCGTTAAGTCGACCGCCTGGGGAGTACGGCCGCAAGGTTAA
AACTCAAATGAATTGACGGGGGCCCGCACAAGCGGTGGAGCATGTGGTTTAATTCGATG
CAACGCGAAGAACCTTACCTACTCTTGACATCCACGGAATTTAGCAGAGATGCTTTAGT
GCCTTCGGGAACCGTGAGACAGGTGCTGCATGGCTGTCGTCAGCTCGTGTTGTGAAATG
TTGGGTTAAGTCCCGCAACGAGCGCAACCCTTATCCTTTGTTGCCAGCACGTGATGGTG
GGAACTCAAAGGAGACTGCCGGTGATAAACCGGAGGAAGGTGGGGATGACGTCAAGT
CATCATGGCCCTTACGAGTAGGGCTACACACGTGCTACAATGGCAGATACAAAGTGAAG
CGAACTCGCGAGAGTAAGCGGACCACATAAAGTCTGTCGTAGTCCGGATTGGAGTCTG
CAACTCGACTCCATGAAGTCGGAATCGCTAGTAATCGTAGATCAGAATGCTACGGTGAAT
ACGTTCCCGGGCCTTGTACACACCGCCCGTCACACCATGGGAGTGGGTTGCAAAAGAA
GTAGGTAGCTTAACCTTCGGGAGGGCGCTTACCACTTTGTGATTCATGACTGGGG
pattern 375
CGCTGGCGGCAGGCCTAACACATGCAAGTCGAGCGGCAGCGGAAAGTAGCTTGCTACT
TTGCCGGCGAGCGGCGGACGGGTGAGTAATGTCTGGGGATCTGCCTGATGGAGGGGGA
TAACTACTGGAAACGGTAGCTAATACCGCATGACCTCGAAAGAGCAAAGTGGGGGACC
TTCGGGCCTCACGCCATCGGATGAACCCAGATGGGATTAGCTAGTAGGTGATGTAATGG
CTCACCTAGGCGACGATCCCTAGCTGGTCTGAGAGGATGACCAGCCACACTGGAACTG
AGACACGGTCCAGACTCCTACGGGAGGCAGCAGTGGGGAATATTGCACAATGGGCGCA
AGCCTGATGCAGCCATGCCGCGTGTGTGAAGAAGGCCTTCGGGTTGTAAAGCACTTTCA
GCGAGGAGGAAGGCATTGTGGTTAATAACCGCAGTGATTGACGTTACTCGCAGAAGAA
GCACCGGCTAACTCCGTGCCAGCAGCCGCGGTAATACGGAGGGTGCAAGCGTTAATCG
GAATTACTGGGCGTAAAGCGCACGCAGGCGGTTTGTTAAGTCAGATGTGAAATCCCCGC
GCTTAACGTGGGAACTGCATTTGAAACTGGCAAGCTAGAGTCTTGTAGAGGGGGGTAG
AATTCCAGGTGTAGCGGTGAAATGCGTAGAGATCTGGAGGAATACCGGTGGCGAAGGC
GGCCCCCTGGACAAAGACTGACGCTCAGGTGCGAAAGCGTGGGGAGCAAACAGGATT
AGATACCCTGGTAGTCCACGCTGTAAACGATGTCGACTTGGAGGTTGTGCCCTTGAGGC
GTGGCTTCCGGAGCTAACGCGTTAAGTCGACCGCCTGGGGAGTACGGCCGCAAGGTTA
AAACTCAAATGAATTGACGGGGGCCCGCACAAGCGGTGGAGCATGTGGTTTAATTCGAT

GCAACGCGAAGAACCTTACCTACTCTTGACATCCACGGAATTTAGCAGAGATGCTTTAG
TGCCTTCGGGAACCGTGAGACAGGTGCTGCATGGCTGTCGTCAGCTCGTGTTGTGAAAT
GTTGGGTTAAGTCCCGCAACGAGCGCAACCCTTATCCTTTGTTGCCAGCACGTAATGGT
GGGAACTCAAGGGAGACTGCCGGTGACAAACCGGAGGAAGGTGGGGATGACGTCAAG
TCATCATGGCCCTTACGAGTAGGGCTACACACGTGCTACAATGGCAGATACAAAGTGAA
GCGAACTCGCGAGAGCAAGCGGACCACATAAAGTCTGTCGTAGTCCGGATTGGAGTCT
GCAACTCGACTCCATGAAGTCGGAATCGCTAGTAATCGTAGATCAGAATGCTACGGTGA
ATACGTTCCCGGGCCTTGTACACACCGCCCGTCACACCATGGGAGTGGGTTGCAAAAG
AAGTAGGTAGCTTAACCTTCGGGAGGGCGCTTACCACTTTGTGATTCATGACTGGGG
pattern 376
CGCTGGCGGCAGGCCTAACACATGCAAGTCGAGCGGCAGCGGGAAGTAGTTTACTACT
TTGCCGGCGAGCGGCGGACGGGTGAGTAATGTCTGGGAAACTGCCTGATGGAGGGGGA
TAACTACTGGAAACGGTAGCTAATACCGCATGACCTCGCCAGAGCAAAGTGGGGGACC
TTCGGGCCTCACGCCATCGGATGTGCCCAGATGGGATTAGCTAGTAGGTGGGGTAATGG
CTCACCTAGGCGACGATCCCTAGCTGGTCTGAGAGGATGACCAGCCACACTGGAACTG
AGACACGGTCCAGACTCCTACGGGAGGCAGCAGTGGGGAATATTGCACAATGGGCGCA
AGCCTGATGCAGCCATGCCGCGTGTGTGAAGAAGGCCTTCGGGTTGTAAAGCACTTTCA
GCGAGGAGGAAGGCATACGGGTTAATAGCCCGTGTGATTGACGTTACTCGCAGAAGAA
GCACCGGCTAACTCCGTGCCAGCAGCCGCGGTAATACGGAGGGTGCAAGCGTTAATCG
GAATTACTGGGCGTAAAGCGCACGCAGGCGGTTTGTTAAGTCAGATGTGAAATCCCCGC
GCTTAACGTGGGAACTGCATTTGAAACTGGCAAGCTAGAGTCTTGTAGAGGGGGGTAG
AATTCCAGGTGTAGCGGTGAAATGCGTAGAGATCTGGAGGAATACCGGTGGCGAAGGC
GGCCCCCTGGACAAAGACTGACGCTCAGGTGCGAAAGCGTGGGGAGCAAACAGGATT
AGATACCCTGGTAGTCCACGCTGTAAACGATGTCGACTTGGAGGTTGTGCCCTTGAGGC
GTGGCTTCCGGAGCTAACGCGTTAAGTCGACCGCCTGGGGAGTACGGCCGCAAGGTTA
AAACTCAAATGAATTGACGGGGGCCCGCACAAGCGGTGGAGCATGTGGTTTAATTCGAT
GCAACGCGAAGAACCTTACCTACTCTTGACATCCACAGAACTTAGCAGAGATGCTTCAG
TGCCTTCGGGAACTGTGAGACAGGTGCTGCATGGCTGTCGTCAGCTCGTGTTGTGAAAT
GTTGGGTTAAGTCCCGCAACGAGCGCAACCCTTATCCTTTGTTGCCAGCACGTAATGGT
GGGAACTCAAGGGAGACTGCCGGTGACAAACCGGAGGAAGGTGGGGATGACGTCAAG
TCATCATGGCCCTTACGAGTAGGGCTACACACGTGCTACAATGGCAGATACAAAGTGAA
GCGAACTCGCGAGAGCAAGCGGACCACATAAAGTCTGTCGTAGTCCGGATTGGAGTCT
GCAACTCGACTCCATGAAGTCGGAATCGCTAGTAATCGTAGATCAGAATGCTACGGTGA
ATACGTTCCCGGGCCTTGTACACACCGCCCGTCACACCATGGGAGTGGGTTGCAAAAG
AAGTAGGTAGCTTAACCTTCGGGAGGGCGCTTACCACTTTGTGATTCATGACTGGGG
pattern 377
CGCTGGCGGCAGGCCTAACACATGCAAGTCGAGCGGCAGCGGGAAGTAGTTTACTACT
TTGCCGGCGAGCGGCGGACGGGTGAGTAATGTCTGGGAAACTGCCTGATGGAGGGGGA
TAACTACTGGAAACGGTAGCTAATACCGCATGACCTCGCAAGAGCAAAGTGGGGGACC
TTCGGGCCTCACGCCATCGGATGTGCCCAGATGGGATTAGCTAGTAGGTGGGGTAATGG
CTCACCTAGGCGACGATCCCTAGCTGGTCTGAGAGGATGACCAGCCACACTGGAACTG
AGACACGGTCCAGACTCCTACGGGAGGCAGCAGTGGGGAATATTGCACAATGGGCGCA
AGCCTGATGCAGCCATGCCGCGTGTGTGAAGAAGGCCTTCGGGTTGTAAAGCACTTTCA
GCGAGGAGGAAGGCAGTCGTGTTAATAGCACGGTTGATTGACGTTACTCGCAGAAGAA

GCACCGGCTAACTCCGTGCCAGCAGCCGCGGTAATACGGAGGGTGCAAGCGTTAATCG
GAATTACTGGGCGTAAAGCGCACGCAGGCGGTTTGTTAAGTCAGATGTGAAATCCCCGC
GCTTAACGTGGGAACTGCATTTGAAACTGGCAAGCTAGAGTCTTGTAGAGGGGGGTAG
AATTCCAGGTGTAGCGGTGAAATGCGTAGAGATCTGGAGGAATACCGGTGGCGAAGGC
GGCCCCCTGGACAAAGACTGACGCTCAGGTGCGAAAGCGTGGGGAGCAAACAGGATT
AGATACCCTGGTAGTCCACGCTGTAAACGATGTCGACTTGGAGGTTGTGCCCTTGAGGC
GTGGCTTCCGGAGCTAACGCGTTAAGTCGACCGCCTGGGGAGTACGGCCGCAAGGTTA
AAACTCAAATGAATTGACGGGGGCCCGCACAAGCGGTGGAGCATGTGGTTTAATTCGAT
GCAACGCGAAGAACCTTACCTACTCTTGACATCCACAGAACTTAGCAGAGATGCTTCGG
TGCCTTCGGGAACTGTGAGACAGGTGCTGCATGGCTGTCGTCAGCTCGTGTTGTGAAAT
GTTGGGTTAAGTCCCGCAACGAGCGCAACCCTTATCCTTTGTTGCCAGCACGTAATGGT
GGGAACTCAAGGGAGACTGCCGGTGACAAACCGGAGGAAGGTGGGGATGACGTCAAG
TCATCATGGCCCTTACGAGTAGGGCTACACACGTGCTACAATGGCAGGTACAAAGTGAA
GCGAACTCGCGAGAGCAAGCGGACCACATAAAGTCTGTCGTAGTCCGGATTGGAGTCT
GCAACTCGACTCCATGAAGTCGGAATCGCTAGTAATCGTAGATCAGAATGCTACGGTGA
ATACGTTCCCGGGCCTTGTACACACCGCCCGTCACACCATGGGAGTGGGTTGCAAAAG
AAGTAGGTAGCTTAACCTTCGGGAGGGCGCTTACCACTTTGTGATTCATGACTGGGG
pattern 378
CGCTGGCGGCAGGCCTAACACATGCAAGTCGAGCGGCAGCGGGAAGTAGTTTACTACT
TTGCCGGCGAGCGGCGGACGGGTGAGTAATGTCTGGGAAACTGCCTGATGGAGGGGGA
TAACTACTGGAAACGGTAGCTAATACCGCATGACCTCGCAAGAGCAAAGTGGGGGACC
TTCGGGCCTCACGCCATCGGATGTGCCCAGATGGGATTAGCTAGTAGGTGGGGTAATGG
CTCACCTAGGCGACGATCCCTAGCTGGTCTGAGAGGATGACCAGCCACACTGGAACTG
AGACACGGTCCAGACTCCTACGGGAGGCAGCAGTGGGGAATATTGCACAATGGGCGCA
AGCCTGATGCAGCCATGCCGCGTGTGTGAAGAAGGCCTTCGGGTTGTAAAGCACTTTCA
GCGAGGAGGAAGGGTTCAGTGTTAATAGCACTGTTCATTGACGTTACTCGCAGAAGAA
GCACCGGCTAACTCCGTGCCAGCAGCCGCGGTAATACGGAGGGTGCAAGCGTTAATCG
GAATTACTGGGCGTAAAGCGCACGCAGGCGGTTTGTTAAGTCAGATGTGAAATCCCCGC
GCTTAACGTGGGAACTGCATTTGAAACTGGCAAGCTAGAGTCTTGTAGAGGGGGGTAG
AATTCCAGGTGTAGCGGTGAAATGCGTAGAGATCTGGAGGAATACCGGTGGCGAAGGC
GGCCCCCTGGACAAAGACTGACGCTCAGGTGCGAAAGCGTGGGGAGCAAACAGGATT
AGATACCCTGGTAGTCCACGCTGTAAACGATGTCGACTTGGAGGTTGTGCCCTTGAGGC
GTGGCTTCCGGAGCTAACGCGTTAAGTCGACCGCCTGGGGAGTACGGCCGCAAGGTTA
AAACTCAAATGAATTGACGGGGGCCCGCACAAGCGGTGGAGCATGTGGTTTAATTCGAT
GCAACGCGAAGAACCTTACCTACTCTTGACATCCACAGAACTTAGCAGAGATGCTTCGG
TGCCTTCGGGAACTGTGAGACAGGTGCTGCATGGCTGTCGTCAGCTCGTGTTGTGAAAT
GTTGGGTTAAGTCCCGCAACGAGCGCAACCCTTATCCTTTGTTGCCAGCACGTAATGGT
GGGAACTCAAGGGAGACTGCCGGTGACAAACCGGAGGAAGGTGGGGATGACGTCAAG
GCATCATGGCCCTTACGAGTAGGGCTACACACGTGCTACAATGGCAGATACAAAGTGAA
GCGAACTCGCGAGAGCAAGCGGACCACATAAAGTCTGTCGTAGTCCGGATTGGAGTCT
GCAACTCGACTCCATGAAGTCGGAATCGCTAGTAATCGTAGATCAGAATGCTACGGTGA
ATACGTTCCCGGGCCTTGTACACACCGCCCGTCACACCATGGGAGTGGGTTGCAAAAG
AAGTAGGTAGCTTAACCTTCGGGAGGGCGCTTACCACTTTGTGATTCATGACTGGGG
pattern 379

CGCTGGCGGCAGGCCTAACACATGCAAGTCGAGCGGCAGCGGGAAGTAGTTTACTACT
TCGCCGGCGAGCGGCGGACGGGTGAGTAATGTCTGGGAAACTGCCTGATGGAGGGGGA
TAACTACTGGAAACGGTAGCTAATACCGCATGACCTCGCAAGAGCAAAGTGGGGGACC
TTCGGGCCTCACGCCATCGGATGTGCCCAGATGGGATTAGCTAGTAGGTGGGGTAATGG
CTCACCTAGGCGACGATCCCTAGCTGGTCTGAGAGGATGACCAGCCACACTGGAACTG
AGACACGGTCCAGACTCCTACGGGAGGCAGCAGTGGGGAATATTGCACAATGGGCGCA
AGCCTGATGCAGCCATGCCGCGTGTGTGAAGAAGGCCTTCGGGTTGTAAAGCACTTTCA
GCGAGGAGGAAGGGTTCAGTGTTAATAGCACTGTACATTGACGTTACTCGCAGAAGAA
GCACCGGCTAACTCCGTGCCAGCAGCCGCGGTAATACGGAGGGTGCAAGCGTTAATCG
GAATTACTGGGCGTAAAGCGCACGCAGGCGGTTTGTTAAGTCAGATGTGAAATCCCCGC
GCTTAACGTGGGAACTGCATTTGAAACTGGCAAGCTAGAGTCTTGTAGAGGGGGGTAG
AATTCCAGGTGTAGCGGTGAAATGCGTAGAGATCTGGAGGAATACCGGTGGCGAAGGC
GGCCCCCTGGACAAAGACTGACGCTCAGGTGCGAAAGCGTGGGGAGCAAACAGGATT
AGATACCCTGGTAGTCCACGCTGTAAACGATGTCGACTTGGAGGTTGTGCCCTTGAGGC
GTGGCTTCCGGAGCTAACGCGTTAAGTCGACCGCCTGGGGAGTACGGCCGCAAGGTTA
AAACTCAAATGAATTGACGGGGGCCCGCACAAGCGGTGGAGCATGTGGTTTAATTCGAT
GCAACGCGAAGAACCTTACCTACTCTTGACATCCACAGAACTTAGCAGAGATGCTTAGG
TGCCTTCGGGAACTGTGAGACAGGTGCTGCATGGCTGTCGTCAGCTCGTGTTGTGAAAT
GTTGGGTTAAGTCCCGCAACGAGCGCAACCCTTATCCTTTGTTGCCAGCACGTAATGGT
GGGAACTCAAGGGAGACTGCCGGTGACAAACCGGAGGAAGGTGGGGATGACGTCAAG
TCATCATGGCCCTTACGAGTAGGGCTACACACGTGCTACAATGGCAGATACAAAGTGAA
GCGAACTCGCGAGAGCAAGCGGACCACATAAAGTCTGTCGTAGTCCGGATTGGAGTCT
GCAACTCGACTCCATGAAGTCGGAATCGCTAGTAATCGTAGATCAGAATGCTACGGTGA
ATACGTTCCCGGGCCTTGTACACACCGCCCGTCACACCATGGGAGTGGGTTGCAAAAG
AAGTAGGTAGCTTAACCTTCGGGAGGGCGCTTACCACTTTGTGATTCATGACTGGGG
pattern 380
CGCTGGCGGCAGGCCTAACACATGCAAGTCGAGCGGCAGCGGGAAGTAGTTTACTACT
TTGCCGGCGAGCGGCGGACGGGTGAGTAATGTCTGGGAAACTGCCTGATGGAGGGGGA
TAACTACTGGAAACGGTAGCTAATACCGCATGACCTCGCAAGAGCAAAGTGGGGGACC
TTCGGGCCTCACGCCATCGGATGTGCCCAGATGGGATTAGCTAGTAGGTGAGGTAATGG
CTCACCTAGGCGACGATCCCTAGCTGGTCTGAGAGGATGACCAGCCACACTGGAACTG
AGACACGGTCCAGACTCCTACGGGAGGCAGCAGTGGGGAATATTGCACAATGGGCGCA
AGCCTGATGCAGCCATGCCGCGTGTGTGAAGAAGGCCTTCGGGTTGTAAAGCACTTTCA
GCGAGGAGGAAGGGTTTAGTGTTAATAGCACTGAGCATTGACGTTACTCGCAGAAGAA
GCACCGGCTAACTCCGTGCCAGCAGCCGCGGTAATACGGAGGGTGCAAGCGTTAATCG
GAATTACTGGGCGTAAAGCGCACGCAGGCGGTTTGTTAAGTCAGATGTGAAATCCCCGC
GCTTAACGTGGGAACTGCATTTGAAACTGGCAAGCTAGAGTCTTGTAGAGGGGGGTAG
AATTCCAGGTGTAGCGGTGAAATGCGTAGAGATCTGGAGGAATACCGGTGGCGAAGGC
GGCCCCCTGGACAAAGACTGACGCTCAGGTGCGAAAGCGTGGGGAGCAAACAGGATT
AGATACCCTGGTAGTCCACGCTGTAAACGATGTCGACTTGGAGGTTGTGCCCTTGAGGC
GTGGCTTCCGGAGCTAACGCGTTAAGTCGACCGCCTGGGGAGTACGGCCGCAAGGTTA
AAACTCAAATGAATTGACGGGGGCCCGCACAAGCGGTGGAGCATGTGGTTTAATTCGAT
GCAACGCGAAGAACCTTACCTACTCTTGACATCCACAGAATTTGGCAGAGATGCCTTAG
TGCCTTCGGGAACTGTGAGACAGGTGCTGCATGGCTGTCGTCAGCTCGTGTTGTGAAAT

GTTGGGTTAAGTCCCGCAACGAGCGCAACCCTTATCCTTTGTTGCCAGCACGTAATGGT
GGGAACTCAAGGGAGACTGCCGGTGACAAACCGGAGGAAGGTGGGGATGACGTCAAG
TCATCATGGCCCTTACGAGTAGGGCTACACACGTGCTACAATGGCAGATACAAAGTGAA
GCGAACTCGCGAGAGCAAGCGGACCACATAAAGTCTGTCGTAGTCCGGATTGGAGTCT
GCAACTCGACTCCATGAAGTCGGAATCGCTAGTAATCGTAGATCAGAATGCTACGGTGA
ATACGTTCCCGGGCCTTGTACACACCGCCCGTCACACCATGGGAGTGGGTTGCAAAAG
AAGTAGGTAGCTTAACCTTCGGGAGGGCGCTTACCACTTTGTGATTCATGACTGGGG
pattern 381
CGCTGGCGGCAGGCCTAACACATGCAAGTCGAGCGGCAGCGGGAAGTAGTTTACTACT
TTGCCGGCGAGTGGCGGACGGGTGAGTAATGTCTGGGAAACTGCCTGATGGAGGGGGA
TAACTACTGGAAACGGTAGCTAATACCGCATGACCTCGCAAGAGCAAAGTGGGGGACC
TTCGGGCCTCACGCCATCGGATGTGCCCAGATGGGATTAGCTAGTAGGTGAGGTAATGG
CTCACCTAGGCGACGATCCCTAGCTGGTCTGAGAGGATGACCAGCCACACTGGAACTG
AGACACGGTCCAGACTCCTACGGGAGGCAGCAGTGGGGAATATTGCACAATGGGCGCA
AGCCTGATGCAGCCATGCCGCGTGTGTGAAGAAGGCCTTCGGGTTGTAAAGCACTTTCA
GCGAGGAGGAAGGGTTCAGTGTTAATAGCACTGAGCATTGACGTTACTCGCAGAAGAA
GCACCGGCTAACTCCGTGCCAGCAGCCGCGGTAATACGGAGGGTGCAAGCGTTAATCG
GAATTACTGGGCGTAAAGCGCACGCAGGCGGTTTGTTAAGTCAGATGTGAAATCCCCGC
GCTTAACGTGGGAACTGCATTTGAAACTGGCAAGCTAGAGTCTTGTAGAGGGGGGTAG
AATTCCAGGTGTAGCGGTGAAATGCGTAGAGATCTGGAGGAATACCGGTGGCGAAGGC
GGCCCCCTGGACAAAGACTGACGCTCAGGTGCGAAAGCGTGGGGAGCAAACAGGATT
AGATACCCTGGTAGTCCACGCTGTAAACGATGTCGACTTGGAGGTTGTGCCCTTGAGGC
GTGGCTTCCGGAGCTAACGCGTTAAGTCGACCGCCTGGGGAGTACGGCCGCAAGGTTA
AAACTCAAATGAATTGACGGGGGCCCGCACAAGCGGTGGAGCATGTGGTTTAATTCGAT
GCAACGCGAAGAACCTTACCTACTCTTGACATCCACGGAATTTGGCAGAGATGCCTTAG
TGCCTTCGGGAACCGTGAGACAGGTGCTGCATGGCTGTCGTCAGCTCGTGTTGTGAAAT
GTTGGGTTAAGTCCCGCAACGAGCGCAACCCTTATCCTTTGTTGCCAGCACGTAATGGT
GGGAACTCAAGGGAGACTGCCGGTGACAAACCGGAGGAAGGTGGGGATGACGTCAAG
TCATCATGGCCCTTACGAGTAGGGCTACACACGTGCTACAATGGCAGATACAAAGTGAA
GCGAACTCGCGAGAGCAAGCGGACCACATAAAGTCTGTCGTAGTCCGGATTGGAGTCT
GCAACTCGACTCCATGAAGTCGGAATCGCTAGTAATCGTAGATCAGAATGCTACGGTGA
ATACGTTCCCGGGCCTTGTACACACCGCCCGTCACACCATGGGAGTGGGTTGCAAAAG
AAGTAGGTAGCTTAACCTTCGGGAGGGCGCTTACCACTTTGTGATTCATGACTGGGG
pattern 382
CGCTGGCGGCAGGCCTAACACATGCAAGTCGAGCGGCAGCGGGAAGTAGTTTACTACT
TTGCCGGCGAGTGGCGGACGGGTGAGTAATGTCTGGGAAACTGCCTGATGGAGGGGGA
TAACTACTGGAAACGGTAGCTAATACCGCATGACCTCGCAAGAGCAAAGTGGGGGACC
TTCTGGCCTCACGCCATCGGATGTGCCCAGATGGGATTAGCTAGTAGGTGAGGTAATGG
CTCACCTAGGCGACGATCCCTAGCTGGTCTGAGAGGATGACCAGCCACACTGGAACTG
AGACACGGTCCAGACTCCTACGGGAGGCAGCAGTGGGGAATATTGCACAATGGGCGCA
AGCCTGATGCAGCCATGCCGCGTGTGTGAAGAAGGCCTTCGGGTTGTAAAGCACTTTCA
GCGAGGAGGAAGGGTTTAGTGTTAATAGCACTGAGCATTGACGTTACTCGCAGAAGAA
GCACCGGCTAACTCCGTGCCAGCAGCCGCGGTAATACGGAGGGTGCAAGCGTTAATCG
GAATTACTGGGCGTAAAGCGCACGCAGGCGGTTTGTTAAGTCAGATGTGAAATCCCCGC

GCTTAACGTGGGAACTGCATTTGAAACTGGCAAGCTAGAGTCTTGTAGAGGGGGGTAG
AATTCCAGGTGTAGCGGTGAAATGCGTAGAGATCTGGAGGAATACCGGTGGCGAAGGC
GGCCCCCTGGACAAAGACTGACGCTCAGGTGCGAAAGCGTGGGGAGCAAACAGGATT
AGATACCCTGGTAGTCCACGCTGTAAACGATGTCGACTTGGAGGTTGTGCCCTTGAGGC
GTGGCTTCCGGAGCTAACGCGTTAAGTCGACCGCCTGGGGAGTACGGCCGCAAGGTTA
AAACTCAAATGAATTGACGGGGGCCCGCACAAGCGGTGGAGCATGTGGTTTAATTCGAT
GCAACGCGAAGAACCTTACCTACTCTTGACATCCACAGAATTTGGCAGAGATGCCTTAG
TGCCTTCGGGAACTGTGAGACAGGTGCTGCATGGCTGTCGTCAGCTCGTGTTGTGAAAT
GTTGGGTTAAGTCCCGCAACGAGCGCAACCCTTATCCTTTGTTGCCAGCACGTAATGGT
GGGAACTCAAGGGAGACTGCCGGTGACAAACCGGAGGAAGGTGGGGATGACGTCAAG
TCATCATGGCCCTTACGAGTAGGGCTACACACGTGCTACAATGGCAGATACAAAGTGAA
GCGAACTCGCGAGAGCAAGCGGACCACATAAAGTCTGTCGTAGTCCGGATTGGAGTCT
GCAACTCGACTCCATGAAGTCGGAATCGCTAGTAATCGTAGATCAGAATGCTACGGTGA
ATACGTTCCCGGGCCTTGTACACACCGCCCGTCACACCATGGGAGTGGGTTGCAAAAG
AAGTAGGTAGCTTAACCTTCGGGAGGGCGCTTACCACTTTGTGATTCATGACTGGGG
pattern 383
CGCTGGCGGCAGGCCTAACACATGCAAGTCGAGCGGCAGCGGGAAGTAGTTTACTACT
TTGCCGGCGAGCGGCGGACGGGTGAGTAATGTCTGGGAAACTGCCTGATGGAGGGGGA
TAACTACTGGAAACGGTAGCTAATACCGCATGACCTCGCAAGAGCAAAGTGGGGGACC
TTCGGGCCTCACGCCATCGGATGTGCCCAGATGGGATTAGCTAGTAGGTGAGGTAATGG
CTCACCTAGGCGACGATCCCTAGCTGGTCTGAGAGGATGACCAGCCACACTGGAACTG
AGACACGGTCCAGACTCCTACGGGAGGCAGCAGTGGGGAATATTGCACAATGGGCGCA
AGCCTGATGCAGCCATGCCGCGTGTGTGAAGAAGGCCTTCGGGTTGTAAAGCACTTTCA
GCGAGGAGGAAGGGTTCAGTGTTAATAGCACTGAGCATTGACGTTACTCGCAGAAGAA
GCACCGGCTAACTCCGTGCCAGCAGCCGCGGTAATACGGAGGGTGCAAGCGTTAATCG
GAATTACTGGGCGTAAAGCGCACGCAGGCGGTTTGTTAAGTCAGATGTGAAATCCCCGC
GCTTAACGTGGGAACTGCATTTGAAACTGGCAAGCTAGAGTCTTGTAGAGGGGGGTAG
AATTCCAGGTGTAGCGGTGAAATGCGTAGAGATCTGGAGGAATACCGGTGGCGAAGGC
GGCCCCCTGGACAAAGACTGACGCTCAGGTGCGAAAGCGTGGGGAGCAAACAGGATT
AGATACCCTGGTAGTCCACGCTGTAAACGATGTCGACTTGGAGGTTGTGCCCTTGAGGC
GTGGCTTCCGGAGCTAACGCGTTAAGTCGACCGCCTGGGGAGTACGGCCGCAAGGTTA
AAACTCAAATGAATTGACGGGGGCCCGCACAAGCGGTGGAGCATGTGGTTTAATTCGAT
GCAACGCGAAGAACCTTACCTACTCTTGACATCCACGGGATTTGGCAGAGATGCCTTAG
TGCCTTCGGGAACCGTGAGACAGGTGCTGCATGGCTGTCGTCAGCTCGTGTTGTGAAAT
GTTGGGTTAAGTCCCGCAACGAGCGCAACCCTTATCCTTTGTTGCCAGCACGTAATGGT
GGGAACTCAAGGGAGACTGCCGGTGACAAACCGGAGGAAGGTGGGGATGACGTCAAG
TCATCATGGCCCTTACGAGTAGGGCTACACACGTGCTACAATGGCAGATACAAAGTGAA
GCGAACTCGCGAGAGCAAGCGGACCACATAAAGTCTGTCGTAGTCCGGATTGGAGTCT
GCAACTCGACTCCATGAAGTCGGAATCGCTAGTAATCGTAGATCAGAATGCTACGGTGA
ATACGTTCCCGGGCCTTGTACACACCGCCCGTCACACCATGGGAGTGGGTTGCAAAAG
AAGTAGGTAGCTTAACCTTCGGGAGGGCGCTTACCACTTTGTGATTCATGACTGGGG
pattern 384
CGCTGGCGGCAGGCCTAACACATGCAAGTCGAGCGGCAGCGGGAAGTAGTTTACTACT
TTGCCGGCGAGCGGCGGACGGGTGAGTAATGTCTGGGGATCTGCCTGATGGAGGGGGA

TAACTACTGGAAACGGTAGCTAATACCGCATGACCTCGCAAGAGCAAAGTGGGGGACC
TTAGGGCCTCACGCCATCGGATGAACCCAGATGGGATTAGCTAGTAGGTGGGGTAATGG
CTCACCTAGGCGACGATCCCTAGCTGGTCTGAGAGGATGACCAGCCACACTGGAACTG
AGACACGGTCCAGACTCCTACGGGAGGCAGCAGTGGGGAATATTGCACAATGGGCGCA
AGCCTGATGCAGCCATGCCGCGTGTGTGAAGAAGGCCTTCGGGTTGTAAAGCACTTTCA
GCGAGGAGGAAGGGGTTGAGTTTAATACGCTCAATCATTGACGTTACTCGCAGAAGAA
GCACCGGCTAACTCCGTGCCAGCAGCCGCGGTAATACGGAGGGTGCAAGCGTTAATCG
GAATTACTGGGCGTAAAGCGCACGCAGGCGGTTTGTTAAGTCAGATGTGAAATCCCCGC
GCTTAACGTGGGAACTGCATTTGAAACTGGCAAGCTAGAGTCTTGTAGAGGGGGGTAG
AATTCCAGGTGTAGCGGTGAAATGCGTAGAGATCTGGAGGAATACCGGTGGCGAAGGC
GGCCCCCTGGACAAAGACTGACGCTCAGGTGCGAAAGCGTGGGGAGCAAACAGGATT
AGATACCCTGGTAGTCCACGCTGTAAACGATGTCGACTTGGAGGTTGTGCCCTTGAGGC
GTGGCTTCCGGAGCTAACGCGTTAAGTCGACCGCCTGGGGAGTACGGCCGCAAGGTTA
AAACTCAAATGAATTGACGGGGGCCCGCACAAGCGGTGGAGCATGTGGTTTAATTCGAT
GCAACGCGAAGAACCTTACCTACTCTTGACATCCACAGAATTTGGCAGAGATGTTAAAG
TGCCTTCGGGAACTGTGAGACAGGTGCTGCATGGCTGTCGTCAGCTCGTGTTGTGAAAT
GTTGGGTTAAGTCCCGCAACGAGCGCAACCCTTATCCTTTGTTGCCAGCACGTAATGGT
GGGAACTCAAGGGAGACTGCCGGTGACAAACCGGAGGAAGGTGGGGATGACGTCAAG
TCATCATGGCCCTTACGAGTAGGGCTACACACGTGCTACAATGGCAGATACAAAGTGAA
GCGAACTCGCGAGAGTCAGCGGACCACATAAAGTCTGTCGTAGTCCGGATTGGAGTCT
GCAACTCGACTCCATGAAGTCGGAATCGCTAGTAATCGTAGATCAGAATGCTACGGTGA
ATACGTTCCCGGGCCTTGTACACACCGCCCGTCACACCATGGGAGTGGGTTGCAAAAG
AAGTAGGTAGCTTAACCTTCGGGAGGGCGCTTACCACTTTGTGATTCATGACTGGGG
pattern 385
CGCTGGCGGCAGGCCTAACACATGCAAGTCGAGCGGCAGCGGGAAGTAGTTTACTACT
TTGCCGGCGAGCGGCGGACGGGTGAGTAATGTCTGGGGATCTGCCTGATGGAGGGGGA
TAACTACTGGAAACGGTAGCTAATACCGCATGACCTCGCAAGAGCAAAGTGGGGGACC
TTAGGGCCTCACGCCATCGGATGAACCCAGATGGGATTAGCTAGTAGGTGGGGTAATGG
CTCACCTAGGCGACGATCCCTAGCTGGTCTGAGAGGATGACCAGCCACACTGGAACTG
AGACACGGTCCAGACTCCTACGGGAGGCAGCAGTGGGGAATATTGCACAATGGGCGCA
AGCCTGATGCAGCCATGCCGCGTGTGTGAAGAAGGCCTTCGGGTTGTAAAGCACTTTCA
GCGAGGAGGAAGGGGTTGAGTTTAATACGCTCAATCATTGACGTTACTCGCAGAAGAA
GCACCGGCTAACTCCGTGCCAGCAGCCGCGGTAATACGGAGGGTGCAAGCGTTAATCG
GAATTACTGGGCGTAAAGCGCACGCAGGCGGTTTGTTAAGTCAGATGTGAAATCCCCGC
GCTTAACGTGGGAACTGCATTTGAAACTGGCAAGCTAGAGTCTTGTAGAGGGGGGTAG
AATTCCAGGTGTAGCGGTGAAATGCGTAGAGATCTGGAGGAATACCGGTGGCGAAGGC
GGCCCCCTGGACAAAGACTGACGCTCAGGTGCGAAAGCGTGGGGAGCAAACAGGATT
AGATACCCTGGTAGTCCACGCTGTAAACGATGTCGACTTGGAGGTTGTGCCCTTGAGGC
GTGGCTTCCGGAGCTAACGCGTTAAGTCGACCGCCTGGGGAGTACGGCCGCAAGGTTA
AAACTCAAATGAATTGACGGGGGCCCGCACAAGCGGTGGAGCATGTGGTTTAATTCGAT
GCAACGCGAAGAACCTTACCTACTCTTGACATCCACAGAATTTGGCAGAGATGCTAAAG
TGCCTTCGGGAACTGTGAGACAGGTGCTGCATGGCCGTCGTCAGCTCGTGTTGTGAAAT
GTTGGGTTAAGTCCCGCAACGAGCGCAACCCTTATCCTTTGTTGCCAGCACGTAATGGT
GGGAACTCAAGGGAGACTGCCGGTGACAAACCGGAGGAAGGTGGGGATGACGTCAAG

TCATCATGGCCCTTACGAGTAGGGCTACACACGTGCTACAATGGCAGATACAAAGTGAA
GCGAACTCGCGAGAGCCAGCGGACCACATAAAGTCTGTCGTAGTCCGGATTGGAGTCT
GCAACTCGACTCCATGAAGTCGGAATCGCTAGTAATCGTAGATCAGAATGCTACGGTGA
ATACGTTCCCGGGCCTTGTACACACCGCCCGTCACACCATGGGAGTGGGTTGCAAAAG
AAGTAGGTAGCTTAACCTTCGGGAGGGCGCTTACCACTTTGTGATTCATGACTGGGG
pattern 386
CGCTGGCGGCAGGCCTAACACATGCAAGTCGAGCGGCAGCGGGAAGTAGTTTACTACT
TTGCCGGCGAGCGGCGGACGGGTGAGTAATGTCTGGGGATCTGCCTGATGGAGGGGGA
TAACTACTGGAAACGGTAGCTAATACCGCATGACCTCGCAAGAGCAAAGTGGGGGACC
TTAGGGCCTCACGCCATCGGATGAACCCAGATGGGATTAGCTAGTAGGTGGGGTAATGG
CTCACCTAGGCGACGATCCCTAGCTGGTCTGAGAGGATGACCAGCCACACTGGAACTG
AGACACGGTCCAGACTCCTACGGGAGGCAGCAGTGGGGAATATTGCACAATGGGCGCA
AGCCTGATGCAGCCATGCCGCGTGTGTGAAGAAGGCCTTCGGGTTGTAAAGCACTTTCA
GCGAGGAGGAAGGGGTTGAGTTTAATACGCTCAATCATTGACGTTACTCGCAGAAGAA
GCACCGGCTAACTCCGTGCCAGCAGCCGCGGTAATACGGAGGGTGCAAGCGTTAATCG
GAATTACTGGGCGTAAAGCGCACGCAGGCGGTTTGTTAAGTCAGATGTGAAATCCCCGC
GCTTAACGTGGGAACTGCATTTGAAACTGGCAAGCTAGAGTCTTGTAGAGGGGGGTAG
AATTCCAGGTGTAGCGGTGAAATGCGTAGAGATCTGGAGGAATACCGGTGGCGAAGGC
GGCCCCCTGGACAAAGACTGACGCTCAGGTGCGAAAGCGTGGGGAGCAAACAGGATT
AGATACCCTGGTAGTCCACGCTGTAAACGATGTCGACTTGGAGGTTGTGCCCTTGAGGC
GTGGCTTCCGGAGCTAACGCGTTAAGTCGACCGCCTGGGGAGTACGGCCGCAAGGTTA
AAACTCAAATGAATTGACGGGGGCCCGCACCAGCGGTGGAGCATGTGGTTTAATTCGAT
GCAACGCGAAGAACCTTACCTACTCTTGACATCCACAGAATTTGGCAGAGATGCTAAAG
TGCCTTCGGGAACTGTGAGACAGGTGCTGCATGGCTGTCGTCAGCTCGTGTTGTGAAAT
GTTGGGTTAAGTCCCGCAACGAGCGCAACCCTTATCCTTTGTTGCCAGCACGTAATGGT
GGGAACTCAAGGGAGACTGCCGGTGACAAACCGGAGGAAGGTGGGGATGACGTCAAG
TCATCATGGCCCTTACGAGTAGGGCTACACACGTGCTACAATGGCAGATACAAAGTGAA
GCGAACTCGCGAGAGCCAGCGGACCACATAAAGTCTGTCGTAGTCCGGATTGGAGTCT
GCAACTCGACTCCATGAAGTCGGAATCGCTAGTAATCGTAGATCAGAATGCTACGGTGA
ATACGTTCCCGGGCCTTGTACACACCGCCCGTCACACCATGGGAGTGGGTTGCAAAAG
AAGTAGGTAGCTTAACCTTCGGGAGGGCGCTTACCACTTTGTGATTCATGACTGGGG
pattern 387
CGCTGGCGGCAGGCCTAACACATGCAAGTCGAGCGGCAGCGGAAAGTAGCTTGCTACT
TTGCCGGCGAGCGGCGGACGGGTGAGTAATGTCTGGGGATCTGCCTGATGGAGGGGGA
TAACTACTGGAAACGGTAGCTAATACCGCATGACCTCGAAAGAGCAAAGTGGGGGACC
TTCGGGCCTCACGCCATCGGATGAACCCAGATGGGATTAGCTAGTAGGTGGGGTAATGG
CTCACCTAGGCGACGATCCCTAGCTGGTCTGAGAGGATGACCAGCCACACTGGAACTG
AGACACGGTCCAGACTCCTACGGGAGGCAGCAGTGGGGAATATTGCACAATGGGCGCA
AGCCTGATGCAGCCATGCCGCGTGTGTGAAGAAGGCCTTCGGGTTGTAAAGCACTTTCA
GCGAGGAGGAAGGCATTGTGGTTAATAACCGCAGTGATTGACGTTACTCGCAGAAGAA
GCACCGGCTAACTCCGTGCCAGCAGCCGCGGTAATACGGAGGGTGCAAGCGTTAATCG
GAATTACTGGGCGTAAAGCGCACGCAGGCGGTTTGTTAAGTCAGATGTGAAATCCCCGC
GCTTAACGTGGGAACTGCATTTGAAACTGGCAAGCTAGAGTCTTGTAGAGGGGGGTAG
AATTCCAGGTGTAGCGGTGAAATGCGTAGAGATCTGGAGGAATACCGGTGGCGAAGGC

GGCCCCCTGGACAAAGACTGACGCTCAGGTGCGAAAGCGTGGGGAGCAAACAGGATT
AGATACCCTGGTAGTCCACGCTGTAAACGATGTCGACTTGGAGGTTGTGCCCTTGAGGC
GTGGCTTCCGGAGCTAACGCGTTAAGTCGACCGCCTGGGGAGTACGGCCGCAAGGTTA
AAACTCAAATGAATTGACGGGGGCCCGCACAAGCGGTGGAGCATGTGGTTTAATTCGAT
GCAACGCGAAGAACCTTACCTACTCTTGACATCCACAGAACTTAGCAGAGATGCTTCGG
TGCCTTCGGGAACTGTGAGACAGGTGCTGCATGGCTGTCGTCAGCTCGTGTTGTGAAAT
GTTGGGTTAAGTCCCGCAACGAGCGCAACCCTTATCCTTTGTTGCCAGCACGTAATGGT
GGGAACTCAAGGGAGACTGCCGGTGACAAACCGGAGGAAGGTGGGGATGACGTCAAG
TCATCATGGCCCTTACGAGTAGGGCTACACACGTGCTACAATGGCAGATACAAAGTGAA
GCGAACTCGCGAGAGCAAGCGGACCACATAAAGTCTGTCGTAGTCCGGATTGGAGTCT
GCAACTCGACTCCATGAAGTCGGAATCGCTAGTAATCGTAGATCAGAATGCTACGGTGA
ATACGTTCCCGGGCCTTGTACACACCACCCGTCACACCATGGGAGTGGGTTGCAAAAG
AAGTAGGTAGCTTAACCTTCGGGAGGGCGCTTACCACTTTGTGATTCATGACTGGGG
pattern 388
CGCTGGCGGCAGGCCTAACACATGCAAGTCGAGCGGCAGCGGAAAGTAGCTTGCTACT
TTGCCGGCGAGCGGCGGACGGGTGAGTAATGTCTGGGGATCTGCCTGATGGAGGGGGA
TAACTACTGGAAACGGTAGCTAATACCGCATGACCTCGAAAGAGCAAAGTGGGGGACC
TTCGGGCCTCACGCCATCGGATGAACCCAGATGGGATTAGCTAGTAGGTGGGGTAATGG
CTCACCTAGGCGACGATCCCTAGCTGGTCTGAGAGGATGACCAGCCACACTGGAACTG
AGACACGGTCCAGACTCCTACGGGAGGCAGCAGTGGGGAATATTGCACAATGGGCGCA
AGCCTGATGCAGCCATGCCGCGTGTGTGAAGAAGGCCTTCGGGTTGTAAAGCACTTTCA
GCGAGGAGGAAGGCATTGTGGTTAATAACCGCAGTGATTGACGTTACTCGCAGAAGAA
GCACCGGCTAACTCCGTGCCAGCAGCCGCGGTAATACGGAGGGTGCAAGCGTTAATCG
GAATTACTGGGCGTAAAGCGCACGCAGGCGGTTTGTTAAGTCAGATGTGAAATCCCCGC
GCTTAACGTGGGAACTGCATTTGAAACTGGCAAGCTAGAGTCTTGTAGAGGGGGGTGG
AATTCCAGGTGTAGCGGTGAAATGCGTAGAGATCTGGAGGAATACCGGTGGCGAAGGC
GGCCCCCTGGACAAAGACTGACGCTCAGGTGCGAAAGCGTGGGGAGCAAACAGGATT
AGATACCCTGGTAGTCCACGCTGTAAACGATGTCGACTTGGAGGTTGTGCCCTTGAGGC
GTGGCTTCCGGAGCTAACGCGTTAAGTCGACCGCCTGGGGAGTACGGCCGCAAGGTTA
AAACTCAAATGAATTGACGGGGGCCCGCACAAGCGGTGGAGCATGTGGTTTAATTCGAT
GCAACGCGAAGAACCTTACCTACTCTTGACATCCACAGAACTTAGCAGAGATGCTTCGG
TGCCTTCGGGAACTGTGAGACAGGTGCTGCATGGCTGTCGTCAGCTCGTGTTGTGAAAT
GTTGGGTTAAGTCCCGCAACGAGCGCAACCCTTATCCTTTGTTGCCAGCACGTAATGGT
GGGAACTCAAGGGAGACTGCCGGTGACAAACCGGAGGAAGGTGGGGATGACGTCAAG
TCATCATGGCCCTTACGAGTAGGGCTACACACGTGCTACAATGGCAGATACAAAGTGAA
GCGAACTCGCGAGAGCAAGCGGACCACATAAAGTCTGTCGTAGTCCGGATTGGAGTCT
GCAACTCGACTCCATGAAGTCGGAATCGCCAGTAATCGTAGATCAGAATGCTACGGTGA
ATACGTTCCCGGGCCTTGTACACACCGCCCGTCACACCATGGGAGTGGGTTGCAAAAG
AAGTAGGTAGCTTAACCTTCGGGAGGGCGCTTACCACTTTGTGATTCATGACTGGGG
pattern 389
CGCTGGCGGCAGGCCTAACACATGCAAGTCGAGCGGCAGCGGAAAGTAGCTTGCTACT
TTGCCGGCGAGCGGCGGACGGGTGAGTAATGTCTGGGAAACTGCCTGATGGAGGGGGA
TAACTACTGGAAACGGTAGCTAATACCGCATGACCTCGAAAGAGCAAAGTGGGGGACC
TTCGGGCCTCACGCCATCGGATGAACCCAGATGGGATTAGCTAGTAGGTGAGGTAATGG

CTCACCTAGGCGACGATCCCTAGCTGGTCTGAGAGGATGACCAGCCACACTGGAACTG
AGACACGGTCCAGACTCCTACGGGAGGCAGCAGTGGGGAATATTGCACAATGGGCGCA
AGCCTGATGCAGCCATGCCGCGTGTGTGAAGAAGGCCTTAGGGTTGTAAAGCACTTTCA
GCGAGGAGGAAGGCATCACACTTAATACGTGTGGTGATTGACGTTACTCGCAGAAGAA
GCACCGGCTAACTCCGTGCCAGCAGCCGCGGTAATACGGAGGGTGCAAGCGTTAATCG
GAATTACTGGGCGTAAAGCGCACGCAGGCGGTTTGTTAAGTCAGATGTGAAATCCCCGC
GCTTAACGTGGGAACTGCATTTGAAACTGGCAAGCTAGAGTCTTGTAGAGGGGGGTAG
AATTCCAGGTGTAGCGGTGAAATGCGTAGAGATCTGGAGGAATACCGGTGGCGAAGGC
GGCCCCCTGGACAAAGACTGACGCTCAGGTGCGAAAGCGTGGGGAGCAAACAGGATT
AGATACCCTGGTAGTCCACGCTGTAAACGATGTCGACTTGGAGGTTGTGCCCTTGAGGC
GTGGCTTCCGGAGCTAACGCGTTAAGTCGACCGCCTGGGGAGTACGGCCGCAAGGTTA
AAACTCAAATGAATTGACGGGGGCCCGCACAAGCGGTGGAGCATGTGGTTTAATTCGAT
GCAACGCGAAGAACCTTACCTACTCTTGACATCCACGGAATTCGCCAGAGATGGCTTAG
TGCCTTCGGGAACCGTGAGACAGGTGCTGCATGGCTGTCGTCAGCTCGTGTTGTGAAAT
GTTGGGTTAAGTCCCGCAACGAGCGCAACCCTTATCCTTTGTTGCCAGCACGTAATGGT
GGGAACTCAAGGGAGACTGCCGGTGACAAACCGGAGGAAGGTGGGGATGACGTCAAG
TCATCATGGCCCTTACGAGTAGGGCTACACACGTGCTACAATGGCAGATACAAAGTGAA
GCGAACTCGCGAGAGCAAGCGGACCACATAAAGTCTGTCGTAGTCCGGATTGGAGTCT
GCAACTCGACTCCATGAAGTCGGAATCGCTAGTAATCGTAGATCAGAATGCTACGGTGA
ATACGTTCCCGGGCCTTGTACACACCGCCCGTCACACCATGGGAGTGGGTTGCAAAAG
AAGTAGGTAGCTTAACCTTCGGGAGGGCGCTTACCACTTTGTGATTCATGACTGGGG
pattern 390
CGCTGGCGGCAGGCCTAACACATGCAAGTCGAGCGGCAGCGGAAAGTAGCTTGCTACT
TTGCCGGCGAGCGGCGGACGGGTGAGTAATGTCTGGGGATCTGCCTGATGGAGGGGGA
TAACTACTGGAAACGGTAGCTAATACCGCATGACCTCGAAAGAGCAAAGTGGGGGACC
TTCGGGCCTCACGCCATCGGATGAACCCAGATGGGATTAGCTAGTAGGTGGGGTAATGG
CTCACCTAGGCGACGATCCCTAGCTGGTCTGAGAGGATGACCAGCCACACTGGAACTG
AGACACGGTCCAGACTCCTACGGGAGGCAGCAGTGGGGAATATTGCACAATGGGCGCA
AGCCTGATGCAGCCATGCCGCGTGTGTGAAGAAGGCCTTAGGGTTGTAAAGCACTTTCA
GCGAGGAGGAAGGCATCACACTTAATACGTGTGGTGATTGACGTTACTCGCAGAAGAA
GCACCGGCTAACTCCGTGCCAGCAGCCGCGGTAATACGGAGGGTGCAAGCGTTAATCG
GAATTACTGGGCGTAAAGCGCACGCAGGCGGTTTGTTAAGTCAGATGTGAAATCCCCGC
GCTTAACGTGGGAACTGCATTTGAAACTGGCAAGCTAGAGTCTTGTAGAGGGGGGTAG
AATTCCAGGTGTAGCGGTGAAATGCGTAGAGATCTGGAGGAATACCGGTGGCGAAGGC
GGCCCCCTGGACAAAGACTGACGCTCAGGTGCGAAAGCGTGGGGAGCAAACAGGATT
AGATACCCTGGTAGTCCACGCTGTAAACGATGTCGACTTGGAGGTTGTGCCCTTGAGGC
GTGGCTTCCGGAGCTAACGCGTTAAGTCGACCGCCTGGGGAGTACGGCCGCAAGGTTA
AAACTCAAATGAATTGACGGGGGCCCGCACAAGCGGTGGAGCATGTGGTTTAATTCGAT
GCAACGCGAAGAACCTTACCTACTCTTGACATCCACAGAACTTAGCAGAGATGCTTCGG
TGCCTTCGGGAACTGTGAGACAGGTGCTGCATGGCTGTCGTCAGCTCGTGTTGTGAAAT
GTTGGGTTAAGTCCCGCAACGAGCGCAACCCTTATCCTTTGTTGCCAGCACGTAATGGT
GGGAACTCAAGGGAGACTGCCGGTGACAAACCGGAGGAAGGTGGGGATGACGTCAAG
TCATCATGGCCCTTACGAGTAGGGCTACACACGTGCTACAATGGCAGATACAAAGTGAA
GCGAACTCGCGAGAGCAAGCGGACCACATAAAGTCTGTCGTAGTCCGGATTGGAGTCT

GCAACTCGACTCCATGAAGTCGGAATCGCTAGTAATCGTAGATCAGAATGCTACGGTGA
ATACGTTCCCGGGCCTTGTACACACCGCCCGTCACACCATGGGAGTGGGTTGCAAAAG
AAGTAGGTAGCTTAACCTTCGGGAGGGCGCTTACCACTTTGTGATTCATGACTGGGG
pattern 391
CGCTGGCGGCAGGCCTAACACATGCAAGTCGAGCGGCAGCGGGAAGTAGTTTACTACT
TCGCCGGCGAGCGGCGGACGGGTGAGTAATGTCTGGGAAACTGCCTGATGGAGGGGGA
TAACTACTGGAAACGGTAGCTAATACCGCATGACCTCGCAAGAGCAAAGTGGGGGACC
TTAGGGCCTCACGCCATCGGATGTGCCCAGATGGGATTAGCTAGTAGGTGGGGTAATGG
CTCACCTAGGCGACGATCCCTAGCTGGTCTGAGAGGATGACCAGCCACACTGGAACTG
AGACACGGTCCAGACTCCTACGGGAGGCAGCAGTGGGGAATATTGCACAATGGGCGCA
AGCCTGATGCAGCCATGCCGCGTGTGTGAAGAAGGCCTTCGGGTTGTAAAGCACTTTCA
GCGAGGAGGAAGGCAATCGTGTTAATAGCACGGTTGATTGACGTTACTCGCAGAAGAA
GCACCGGCTAACTCCGTGCCAGCAGCCGCGGTAATACGGAGGGTGCAAGCGTTAATCG
GAATTACTGGGCGTAAATCGCACGCAGGCGGTTTGTTAAGTCAGATGTGAAATCCCCGC
GCTTAACGTGGGAACTGCATTTGAAACTGGCAAGCTAGAGTCTTGTAGAGGGGGGTAG
AATTCCAGGTGTAGCGGTGAAATGCGTAGAGATCTGGAGGAATACCGGTGGCGAAGGC
GGCCCCCTGGACAAAGACTGACGCTCAGGTGCGAAAGCGTGGGGAGCAAACAGGATT
AGATACCCTGGTAGTCCACGCTGTAAACGATGTCGACTTGGAGGTTGTGCCCTTGAGGC
GTGGCTTCCGGAGCTAACGCGTTAAGTCGACCGCCTGGGGAGTACGGCCGCAAGGTTA
AAACTCAAATGAATTGACGGGGGCCCGCACAAGCGGTGGAGCATGTGGTTTAATTCGAT
GCAACGCGAAGAACCTTACCTACTCTTGACATCCACAGAACTTAGCAGAGATGCTTCGG
TGCCTTCGGGAACTGTGAGACAGGTGCTGCATGGCTGTCGTCAGCTCGTGTTGTGAAAT
GTTGGGTTAAGTCCCGCAACGAGCGCAACCCTTATCCTTTGTTGCCAGCACGTAATGGT
GGGAACTCAAGGGAGACTGCCGGTGACAAACCGGAGGAAGGTGGGGATGACGTCAAG
TCATCATGGCCCTTACGAGTAGGGCTACACACGTGCTACAATGGCAGATACAAAGTGAA
GCGAACTCGCGAGAGCAAGCGGACCACATAAAGTCTGTCGTAGTCCGGATTGGAGTCT
GCAACTCGACTCCATGAAGTCGGAATCGCTAGTAATCGTAGATCAGAATGCTACGGTGA
ATACGTTCCCGGGCCTTGTACACACCGCCCGTCACACCATGGGAGTGGGTTGCAAAAG
AAGTAGGTAGCTTAACCTTCGGGAGGGCGCTTACCACTTTGTGATTCATGACTGGGG
pattern 392
CGCTGGCGGCAGGCCTAACACATGCAAGTCGAGCGGCAGCGGAAAGTAGCTTGCTACT
TTGCCGGCGAGCGGCGGACGGGTGAGTAATGTCTGGGAAACTGCCTGATGGGGGGGGA
TAACTACTGGAAACGGTAGCTAATACCGCATGACCTCGCAAGAGCAAAGTGGGGGACC
TTAGGGCCTCACGCCATCGGATGTGCCCAGATGGGATTAGCTAGTAGGTGGGGTAATGG
CTCACCTAGGCGACGATCCCTAGCTGGTCTGAGAGGATGACCAGCCACACTGGAACTG
AGACACGGTCCAGACTCCTACGGGAGGCAGCAGTGGGGAATATTGCACAATGGGCGCA
AGCCTGATGCAGCCATGCCGCGTGTGTGAAGAAGGCCTTCGGGTTGTAAAGCACTTTCA
GCGAGGAGGAAGGCAATCGTGTTAATAGCACGGTTGATTGACGTTACTCGCAGAAGAA
GCACCGGCTAACTCCGTGCCAGCAGCCGCGGTAATACGGAGGGTGCAAGCGTTAATCG
GAATTACTGGGCGTAAAGCGCACGCAGGCGGTTTGTTAAGTCAGATGTGAAATCCCCGC
GCTTAACGTGGGAACTGCATTTGAAACTGGCAAGCTAGAGTCTTGTAGAGGGGGGTAG
AATTCCAGGTGTAGCGGTGAAATGCGTAGAGATCTGGAGGAATACCGGTGGCGAAGGC
GGCCCCCTGGACAAAGACTGACGCTCAGGTGCGAAAGCGTGGGGAGCAAACAGGATT
AGATACCCTGGTAGTCCACGCTGTAAACGATGTCGACTTGGAGGTTGTGCCCTTGAGGC

GTGGCTTCCGGAGCTAACGCGTTAAGTCGACCGCCTGGGGAGTACGGCCGCAAGGTTA
AAACTCAAATGAATTGACGGGGGCCCGCACAAGCGGTGGAGCATGTGGTTTAATTCGAT
GCAACGCGAAGAACCTTACCTACTCTTGACATCCACAGAACTTAGCAGAGATGCTTCGG
TGCCTTCGGGAACTGTGAGACAGGTGCTGCATGGCTGTCGTCAGCTCGTGTTGTGAAAT
GTTGGGTTAAGTCCCGCAACGAGCGCAACCCTTATCCTTTGTTGCCAGCACGTAATGGT
GGGAACTCAAGGGAGACTGCCGGTGACAAACCGGAGGAAGGTGGGGATGACGTCAAG
TCATCATGGCCCTTACGAGTAGGGCTACACACGTGCTACAATGGCAGATACAAAGTGAA
GCGAACTCGCGAGAGCAAGCGGACCACATAAAGTCTGTCGTAGTCCGGATTGGAGTCT
GCAACTCGACTCCATGAAGTCGGAATCGCTAGTAATCGTAGATCAGAATGCTACGGTGA
ATACGTTCCCGGGCCTTGTACACACCGCCCGTCACACCATGGGAGTGGGTTGCAAAAG
AAGTAGGTAGCTTAACCTTCGGGAGGGCGCTTACCACTTTGTGATTCATGACTGGGG
pattern 393
CGCTGGCGGCAGGCCTAACACATGCAAGTCGAGCGGCAGCGGGAAGTAGTTTACTACT
TTGCCGGCGAGCGGCGGACGGGTGAGTAATGTCTGGGAAACTGCCTGATGGAGGGGGA
TAACTACTGGAAACGGTAGCTAATACCGCAGGACCTCGCAAGAGCAAAGTGGGGGACC
TTCGGGCCTCACGCCATCGGATGTGCCCAGATGGGATTAGCTAGTAGGTGGGGTAATGG
CTCACCTAGGCGACGATCCCTAGCTGGTCTGAGAGGATGACCAGCCACACTGGAACTG
AGACACGGTCCAGACTCCTACGGGAGGCAGCAGTGGGGAATATTGCACAATGGGCGCA
AGCCTGATGCAGCCATGCCGCGTGTGTGAAGAAGGCCTTCGGGTTGTAAAGCACTTTCA
GCGAGGAGGAAGGCAGTCGTGTTAATAGCACGATTGATTGACGTTACTCGCAGAAGAA
GCACCGGCTAACTCCGTGCCAGCAGCCGCGGTAATACGGAGGGTGCAAGCGTTAATCG
GAATTACTGGGCGTAAAGCGCACGCAGGCGGTTTGTTAAGTCAGATGTGAAATCCCCGC
GCTTAACGTGGGAACTGCATTTGAAACTGGCAAGCTAGAGTCTTGTAGAGGGGGGTAG
AATTCCAGGTGTAGCGGTGAAATGCGTAGAGATCTGGAGGAATACCGGTGGCGAAGGC
GGCCCCCTGGACAAAGACTGACGCTCAGGTGCGAAAGCGTGGGGAGCAAACAGGATT
AGATACCCTGGTAGTCCACGCTGTAAACGATGTCGACTTGGAGGTTGTGCCCTTGAGGC
GTGGCTTCCGGAGCTAACGCGTTAAGTCGACCGCCTGGGGAGTACGGCCGCAAGGTTA
AAACTCAAATGAATTGACGGGGGCCCGCACAAGCGGTGGAGCATGTGGTTTAATTCGAT
GCAACGCGAAGAACCTTACCTACTCTTGACATCCACAGAACTTAGCAGAGATGCTTTGG
TGCCTTCGGGAACTGTGAGACAGGTGCTGCATGGCTGTCGTCAGCTCGTGTTGTGAAAT
GTTGGGTTAAGTCCCGCAACGAGCGCAACCCTTATCCTTTGTTGCCAGCACGTAATGGT
GGGAACTCAAGGGAGACTGCCGGTGACAAACCGGAGGAAGGTGGGGATGACGTCAAG
TCATCATGGCCCTTACGAGTAGGGCTACACACGTGCTACAATGGCAGATACAAAGTGAA
GCGAACTCGCGAGAGCAAGCGGACCACATAAAGTCTGTCGTAGTCCGGATTGGAGTCT
GCAACTCGACTCCATGAAGTCGGAATCGCTAGTAATCGTAGATCAGAATGCTACGGTGA
ATACGTTCCCGGGCCTTGTACACACCGCCCGTCACACCATGGGAGTGGGTTGCAAAAG
AAGTAGGTAGCTTAACCTTCGGGAGGGCGCTTACCACTTTGTGATTCATGACTGGGG
pattern 394
CGCTGGCGGCAGGCCTAACACATGCAAGTCGAGCGGCAGCGGGAAGTAGTTTACTACT
TTGCCGGCGAGCGGCGGACGGGTGAGTAATGTCTGGGAAACTGCCTGATGGAGGGGGA
TAACTACTGGAAACGGTAGCTAATACCGCATGACCTCGCAAGAGCAAAGTGGGGGACC
TTAGGGCCTCACGCCATCGGATGTGCCCAGATGGGATTAGCTAGTAGGTGGGGTAATGG
CTCACCTAGGCGACGATCCCTAGCTGGTCTGAGAGGATGACCAGCCACACTGGAACTG
AGACACGGTCCAGACTCCTACGGGAGGCAGCAGTGGGGAATATTGCACAATGGGCGCA

AGCCTGATGCAGCCATGCCGCGTGTGTGAAGAAGGCCTTCGGGTTGTAAAGCACTTTCA
GCGAGGAGGAAGGCAATCGTGTTAATAGCACGGTTGATTGACGTTACTCGCAGAAGAA
GCACCGGCTAACTCCGTGCCAGCAGCCGCGGTAATACGGAGGGTGCAAGCGTTAATCG
GAATTACTGGGCGTAAAGCGCACGCAGGCGGTTTGTTAAGTCAGATGTGAAATCCCCGC
GCTTAACGTGGGAACTGCATTTGAAACTGGCAAGCTAGAGTCTTGTAGAGGGGGGTAG
AATTCCAGGTGTAGCGGTGAAATGCGTAGAGATCTGGAGGAATACCGGTGGCGAAGGC
GGCCCCCTGGACAAAGACTGACGCTCAGGTGCGAAAGCGTGGGGAGCAAACAGGATT
AGATACCCTGGTAGTCCACGCTGTAAACGATGTCGACTTGGAGGTTGTGCCCTTGAGGC
GTGGCTTCCGGAGCTAACGCGTTAAGTCGACCGCCTGGGGAGTACGGCCGCAAGGTTA
AAACTCAAATGAATTGACGGGGGCCCGCACAAGCGGTGGAGCATGTGGTTTAATTCGAT
GCAACGCGAAGAACCTTACCTACTCTTGACATCCACAGAACTTAGCAGAGATGCTTCGG
TGCCTTCGGGAACTGTGAGACAGGTGCTGCATGGCTGTCGTCAGCTCGTGTTGTGAAAT
GTTGGGTTAAGTCCCGCAACGAGCGCAACCCTTATCCTTTGTTGCCAGCACGTAATGGT
GGGAACTCAAGGGAGACTGTCGGTGACAAACCGGAGGAAGGTGGGGATGACGTCAAG
TCATCATGGCCCTTACGAGTAGGGCTACACACGTGCTACAATGGCAGATACAAAGTGAA
GCGAACTCGCGAGAGCAAGCGGACCACATAAAGTCTGTCGTAGTCCGGATTGGAGTCT
GCAACTCGACTCCATGAAGTCGGAATCGCTAGTAATCGTAGATCAGAATGCTACGGTGA
ATACGTTCCCGGGCCTTGTACACACCGCCCGTCACACCATGGGAGTGGGTTGCAAAAG
AAGTAGGTAGCTTAACCTTCGGGAGGGCGCTTACCACTTTGTGATTCATGACTGGGG
pattern 395
CGCTGGCGGCAGGCCTAACACATGCAAGTCGAGCGGCAGCGGGAAGTAGTTTACTACT
TTGCCGGCGAGCGGCGGACGGGTGAGTAATGTCTGGGGATCTGCCTGATGGAGGGGGA
TAACTACTGGAAACGGTAGCTAATACCGCATGACCTCGCAAGAGCAAAGTGGGGGACC
TTAGGGCCTCACGCCATCGGATGAACCCAGATGGGATTAGCTAGTAGGTGGGGTAATGG
CTCACCTAGGCGACGATCCCTAGCTGGTCTGAGAGGATGACCAGCCACACTGGAACTG
AGACACGGTCCAGACTCCTACGGGAGGCAGCAGTGGGGAATATTGCACAATGGGCGCA
AGCCTGATGCAGCCATGCCGCGTGTGTGAAGAAGGCCTTCGGGTTGTAAAGCACTTTCA
GCGAGGAGGAAGGGGTTGAGTTTAATACGCTCAATCATTGACGTTACTCGCAGAAGAA
GCACCGGCTAACTCCGTGCCAGCAGCCGCGGTAATACGGAGGGTGCAAGCGTTAATCG
GAATTACTGGGCGTAAAGCGCACGCAGGCGGTTTGTTAAGTCAGATGTGAAATCCCCGC
GCTTAACGTGGGAACTGCATTTGAAACTGGCAAGCTAGAGTCTTGTAGAGGGGGGTAG
AATTCCAGGTGTAGCGGTGAAATGCGTAGAGATCTGGAGGAATACCGGTGGCGAAGGC
GGCCCCCTGGACAAAGACTGACGCTCAGGTGCGAAAGCGTGGGGAGCAAACAGGATT
AGATACCCTGGTAGTCCACGCTGTAAACGATGTCGACTTGGAGGTTGTGCCCTTGAGGC
GTGGCTTCCGGAGCTAACGCGTTAAGTCGACCGCCTGGGGAGTACGGCCGCAAGGTTA
AAACTCAAATGAATTGACGGGGGCCCGCACAAGCGGTGGAGCATGTGGTTTAATTCGAT
GCAACGCGAAGAACCTTACCTACTCTTGACATCCACAGAATTTGGCAGAGATGCTAAAG
TGCCTTCGGGAACTGTGAGACAGGTGCTGCATGGCTGTCGTCAGCTCGTGTTGTGAAAT
GTTGGGTTAAGTCCCGCAACGAGCGCAACCCTTATCCTTTGTTGCCAGCACGTAATGGT
GGGAACTCAAGGGAGACTGCCGGTGACAAACCGGAGGAAGGTGGGGATGACGTCAAG
TCATCATGGCCCTTACGAGTAGGGCTACACACGTGCTACAATGGCAGATACAAAGTGAA
GCGAACTCGCGAGAGCCAGCGGACCACATAAAGTCTGTCGTAGTCCGGATTGGAGTCT
GCAACTCGACTCCATGAAGTCGGAATCGCTAGTAATCGTAGATCAGAATGCTACGGTGA
ATACGTTCCCGGGCCTTGTACACACCGCCCGTCACACCATGGGAGTGGGTTGCAAAAG

AAGTAGGTAGCTTAACCTTTGGGAGGGCGCTTACCACTTTGTGATTCATGACTGGGG
pattern 396
CGCTGGCGGCAGGCCTAACACATGCAAGTCGAGCGGCAGCGGGAAGTAGTTTACTACT
TTGCCGGCGAGCGGCGGACGGGTGAGTAATGTCTGGGGATCTGCCTGATGGAGGGGGA
TAACTACTGGAAACGGTGGCTAATACCGCATGACCTCGCAAGAGCAAAGTGGGGGACC
TTAGGGCCTCACGCCATCGGATGAACCCAGATGGGATTAGCTAGTAGGTGGGGTAATGG
CTCACCTAGGCGACGATCCCTAGCTGGTCTGAGAGGATGACCAGCCACACTGGAACTG
AGACACGGTCCAGACTCCTACGGGAGGCAGCAGTGGGGAATATTGCACAATGGGCGCA
AGCCTGATGCAGCCATGCCGCGTGTGTGAAGAAGGCCTTCGGGTTGTAAAGCACTTTCA
GCGAGGAGGAAGGGGTTGAGTTTAATACGCTCAATCATTGACGTTACTCGCAGAAGAA
GCACCGGCTAACTCCGTGCCAGCAGCCGCGGTAATACGGAGGGTGCAAGCGTTAATCG
GAATTACTGGGCGTAAAGCGCACGCAGGCGGTTTGTTAAGTCAGATGTGAAATCCCCGC
GCTTAACGTGGGAACTGCATTTGAAACTGGCAAGCTAGAGTCTTGTAGAGGGGGGTAG
AATTCCAGGTGTAGCGGTGAAATGCGTAGAGATCTGGAGGAATACCGGTGGCGAAGGC
GGCCCCCTGGACAAAGACTGACGCTCAGGTGCGAAAGCGTGGGGAGCAAACAGGATT
AGATACCCTGGTAGTCCACGCTGTAAACGATGTCGACTTGGAGGTTGTGCCCTTGAGGC
GTGGCTTCCGGAGCTAACGCGTTAAGTCGACCGCCTGGGGAGTACGGCCGCAAGGTTA
AAACTCAAATGAATTGACGGGGGCCCGCACAAGCGGTGGAGCATGTGGTTTAATTCGAT
GCAACGCGAAGAACCTTACCTACTCTTGACATCCACAGAATTTGGCAGAGATGCTAAAG
TGCCTTCGGGAACTGTGAGACAGGTGCTGCATGGCTGTCGTCAGCTCGTGTTGTGAAAT
GTTGGGTTAAGTCCCGCAACGAGCGCAACCCTTATCCTTTGTTGCCAGCACGTAATGGT
GGGAACTCAAGGGAGACTGCCGGTGACAAACCGGAGGAAGGTGGGGATGACGTCAAG
TCATCATGGCCCTTACGAGTAGGGCTACACACGTGCTACAATGGCAGATACAAAGTGAA
GCGAACTCGCGAGAGCCAGCGGACCACATAAAGTCTGTCGTAGTCCGGATTGGAGTCT
GCAACTCGACTCCATGAAGTCGGAATCGCTAGTAATCGTAGATCAGAATGCTACGGTGA
ATACGTTCCCGGGCCTTGTACACACCGCCCGTCACACCATGGGAGTGGGTTGCATAAAG
AAGTAGGTAGCTTAACCTTCGGGAGGGCGCTTACCACTTTGTGATTCATGACTGGGG
pattern 397
CGCTGGCGGCAGGCCTAACACATGCAAGTCGAGCGGCAGCGGGAAGTAGTTTACTACT
TTGCCGGCGAGCGGCGGACGGGTGAGTAATGTCTGGGGATCTGCCTGATGGAGGGGGA
TAACTACTGGAAACGGTAGCTAATACCGCATGACCTCGCAAGAGCAAAGTGGGGGACC
TTAGGGCCTCACGCCATCGGATGAACCCAGATGGGATTAGCTAGTAGGTGGGGTAATGG
CTCACCTAGGCGACGATCCCTAGCTGGTCTGAGAGGATGACCAGCCACACTGGAACTG
AGACACGGTCCAGACTCCTACGGGAGGCAGCAGTGGGGAATATTGCACAATGGGCGCA
AGCCTGATGCAGCCATGCCGCGTGTGTGAAGAAGGCCTTCGGGTTGTAAAGCACTTTCA
GCGAGGAGGAAGGGGTTGAGTTTAATACGCTCAATCATTGACGTTACTCGCAGAAGAA
GCACCGGCTAACTCCGTGCCAGCAGCCGCGGTAATACGGAGGGTGCAAGCGTTAATCG
GAATTACTGGGCGTAAAGCGCACGCAGGCGGTTTGTTAAGTCAGATGTGAAATCCCCGC
GCTTAACGTGGGAACTGCATTTGAAACTGGCAAGCTAGAGTCTTGTAGAGGGGGGTAG
AATTCCAGGTGTAGCGGTGAAATGCGTAGAGATCTGGAGGAATACCGGTGGCGAAGGC
GGCCCCCTGGACAAAGACTGACGCTCAGGTGCGAAAGCGTGGGGAGCAAACAGGATT
AGATACCCTGGTAGTCCACGCTGTAAACGATGTCGACTTGGAGGTTGTGCCCTTGAGGC
GTGGCTTCCGGAGCTAACGCGTTAAGTCGACCGCCTGGGGAGTACGGCCGCAAGGTTA
AAACTCAAATGAATTGACGGGGGCCCGCACAAGCGGTGGAGCATGTGGTTTAATTCGAT

GCAACGCGAAGAACCTTACCTACTCTTGACATCCACAGAATTTGGCAGAGATGCTAAAG
TGCCTTCGGGAACTGTGAGACAGGTGCTGCATGGCTGTCGTCAGCTCGTGTTGTGAAAT
GTTGGGTTAAGTCCCGCAACGAGCGCAACCCTTATCCTTTGTTGCCAGCACGTAATGGT
GGGAACTCAAGGGAGACTGCCGGTGACAAACCGGAGGAAGGTGGGGATGACGTCAAG
TCATCATGGCCCTTACGAGTAGGGCTACACACGTGCTACAATGGCAGATACAAAGTGAA
GCGAACTCGCGAGAGTCAGCGGACCACATAAAGTCTGTCGTAGTCCGGATTGGAGTCT
GCAACTCGACTCCATGAAGTCGGAATCGCTAGTAATCGTAGATCAGAATGCTACGGTGA
ATACGTTCCCGGGCCTTGTACACACCGCCCGTCACACCATGGGAGTGGGTTGCATAAAG
AAGTAGGTAGCTTAACCTTCGGGAGGGCGCTTACCACTTTGTGATTCATGACTGGGG
pattern 398
CGCTGGCGGCAGGCCTAACACATGCAAGTCGAGCGGCAGCGGGAAGTAGTTTACTACT
TTGCCGGCGAGCGGCGGACGGGTGAGTAATGTCTGGGGATCTGCCTGATGGAGGGGGA
TAACTACTGGAAACGGTAGCTAATACCGCATGACCTCGCAAGAGCAAAGTGGGGGACC
TTAGGGCCTCACGCCATCGGATGAACCCAGATGGGATTAGCTAGTAGGTGGGGTAATGG
CTCACCTAGGCGACGATCCCTAGCTGGTCTGAGAGGATGACCAGCCACACTGGAACTG
AGACACGGTCCAGACTCCTACGGGAGGCAGCAGTGGGGAATATTGCACAATGGGCGCA
AGCCTGATGCAGCCATGCCGCGTGTGTGAAGAAGGCCTTCGGGTTGTAAAGCACTTTCA
GCGAGGAGGAAGGGGTTGAGTTTAATACGCTCAATCATTGACGTTACTCGCAGAAGAA
GCACCGGCTAACTCCGTGCCAGCAGCCGCGGTAATACGGAGGGTGCAAGCGTTAATCG
GAATTACTGGGCGTAAAGCGCACGCAGGCGGTTTGTTAAGTCAGATGTGAAATCCCCGC
GCTTAACGTGGGAACTGCATTTGAAACTGGCAAGCTAGAGTCTTGTAGAGGGGGGTAG
AATTCCAGGTGTAGCGGTGAAATGCGTAGAGATCTGGAGGAATACCGGTGGCGAAGGC
GGCCCCCTGGACAAAGACTGACGCTCAGGTGCGAAAGCGTGGGGAGCAAACAGGATT
AGATACCCTGGTAGTCCACGCTGTAAACGATGTCGACTTGGAGGTTGTGCCCTTGAGGC
GTGGCTTCCGGAGCTAACGCGTTAAGTCGACCGCCTGGGGAGTACGGCCGCAAGGTTA
AAACTCAAATGAATTGACGGGGGCCCGCACAAGCGGTGGAGCATGTGGTTTAATTCGAT
GCAACGCGAAGAACCTTACCTACTCTTGACATCCACGGAATTTAGCAGAGATGCTTTAG
TGCCTTCGGGAACCGTGAGACAGGTGCTGCATGGCTGTCGTCAGCTCGTGTTGTGAAAT
GTTGGGTTAAGTCCCGCAACGAGCGCAACCCTTATCCTTTGTTGCCAGCACGTAATGGT
GGGAACTCAAGGGAGACTGCCGGTGACAAACCGGAGGAAGGTGGGGATGACGTCAAG
TCATCATGGCCCTTACGAGTAGGGCTACACACGTGCTACAATGGCAGATACAAAGTGAA
GCGAACTCGCGAGAGCCAGCGGACCACATAAAGTCTGTCGTAGTCCGGATTGGAGTCT
GCAACTCGACTCCATGAAGTCGGAATCGCTAGTAATCGTAGATCAGAATGCTACGGTGA
ATACGTTCCCGGGCCTTGTACACACCGCCCGTCACACCATGGGAGTGGGTTGCAAAAG
AAGTAGGTAGCTTAACCTTTGGGAGGGCGCTTACCACTTTGTGATTCATGACTGGGG
pattern 399
CGCTGGCGGCAGGCCTAACACATGCAAGTCGAGCGGCAGCGGAAAGTAGCTTGCTACT
TTGCCGGCGAGCGGCGGACGGGTGAGTAATGTCTGGGGATCTGCCTGATGGAGGGGGA
TAACTACTGGAAACGGTAGCTAATACCGCATGACCTCGAAAGAGCAAAGTGGGGGACC
TTCGGGCCTCACGCCATCGGATGAACCCAGATGGGATTAGCTAGTAGGTGGGGTAATGG
CTCACCTAGGCGACGATCCCTAGCTGGTCTGAGAGGATGACCAGCCACACTGGAACTG
AGACACGGTCCAGACTCCTACGGGAGGCAGCAGTGGGGAATATTGCACAATGGGCGCA
AGCCTGATGCAGCCATGCCGCGTGTGTGAAGAAGGCCTTCGGGTTGTAAAGCACTTTCA
GCGAGGAGGAAGGCATTGTGGTTAATAACCACAGTGATTGACGTTACTCGCAGAAGAA

GCACCGGCTAACTCCGTGCCAGCAGCCGCGGTAATACGGAGGGTGCAAGCGTTAATCG
GAATTACTGGGCGTAAAGCGCACGCAGGCGGTTTGTTAAGTCAGATGTGAAATCCCCGC
GCTTAACGTGGGAACTGCATTTGAAACTGGCAAGCTAGAGTCTTGTAGAGGGGGGTAG
AATTCCAGGTGTAGCGGTGAAATGCGTAGAGATCTGGAGGAATACCGGTGGCGAAGGC
GGCCCCCTGGACAAAGACTGACGCTCAGGTGCGAAAGCGTGGGGAGCAAACAGGATT
AGATACCCTGGTAGTCCACGCTGTAAACGATGTCGACTTGGAGGTTGTGCCCTTGAGGT
GTGGCTTCCGGAGCTAACGCGTTAAGTCGACCGCCTGGGGAGTACGGCCGCAAGGTTA
AAACTCAAATGAATTGACGGGGGCCCGCACAAGCGGTGGAGCATGTGGTTTAATTCGAT
GCAACGCGAAGAACCTTACCTACTCTTGACATCCACGGAATTTAGCAGAGATGCTTTAG
TGCCTTCGGGAACCGTGAGACAGGTGCTGCATGGCTGTCGTCAGCTCGTGTTGTGAAAT
GTTGGGTTAAGTCCCGCAACGAGCGCAACCCTTATCCTTTGTTGCCAGCACGTAATGGT
GGGAACTCAAGGGAGACTGCCGGTGACAAACCGGAGGAAGGTGGGGATGACGTCAAG
TCATCATGGCCCTTACGAGTAGGGCTACACACGTGCTACAATGGCAGATACAAAGTGAA
GCGAACTCGCGAGAGCAAGCGGACCACATAAAGTCTGTCGTAGTCCGGATTGGAGTCT
GCAACTCGACTCCATGAAGTCGGAATCGCTAGTAATCGTAGATCAGAATGCTACGGTGA
ATACGTTCCCGGGCCTTGTACACACCGCCCGTCACACCATGGGAGTGGGTTGCAAAAG
AAGTAGGTAGCTTAACCTTCGGGAGGGCGCTTACCACTTTGTGATTCATGACTGGGG
pattern 400
CGCTGGCGGCAGGCCTAACACATGCAAGTCGAGCGGCAGCGGGAAGTAGTTTACTACT
TTGCCGGCGAGCGGCGGACGGGTGAGTAATGTCTGGGAAACTGCCTGATGGAGGGGGA
TAACTACTGGAAACGGTAGCTAATACCGCATGACCTCGCAAGAGCAAAGTGGGGGACC
TTCGGGCCTCACGCCATCGGATGTGCCCAGATGGGATTAGCTAGTAGGTGAGGTAATGG
CTCACCTAGGCGACGATCCCTAGCTGGTCTGAGAGGATGACCAGCCACACTGGAACTG
AGACACGGTCCAGACTCCTACGGGAGGCAGCAGTGGGGAATATTGCACAATGGGCGCA
AGCCTGATGCAGCCATGCCGCGTGTGTGAAGAAGGCCTTCGGGTTGTAAAGCACTTTCA
GCGAGGAGGAAGGCAGTCGTGTTAATAGCACGATTGATTGACGTTACTCGCAGAAGAA
GCACCGGCTAACTCCGTGCCAGCAGCCGCGGTAATACGGAGGGTGCAAGCGTTAATCG
GAATTACTGGGCGTAAAGCGCACGCAGGCGGTTTGTTAAGTCAGATGTGAAATCCCCGC
GCTTAACGTGGGAACTGCATTTGAAACTGGCAAGCTAGAGTCTTGTAGAGGGGGGTAG
AATTCCAGGTGTAGCGGTGAAATGCGTAGAGATCTGGAGGAATACCGGTGGCGAAGGC
GGCCCCCTGGACAAAGACTGACGCTCAGGTGCGAAAGCGTGGGGAGCAAACAGGATT
AGATACCCTGGTAGTCCACGCTGTAAACGATGTCGACTTGGAGGTTGTGCCCTTGAGGC
GTGGCTTCCGGAGCTAACGCGTTAAGTCGACCGCCTGGGGAGTACGGCCGCAAGGTTA
AAACTCAAATGAATTGACGGGGGCCCGCACAAGCGGTGGAGCATGTGGTTTAATTCGAT
GCAACGCGAAGAACCTTACCTACTCTTGACATCCACAGAACTTAGCAGAGATGCTTTAG
TGCCTTCGGGAACTGTGAGACAGGTGCTGCATGGCTGTCGTCAGCTCGTGTTGTGAAAT
GTTGGGTTAAGTCCCGCAACGAGCGCAACCCTTATCCTTTGTTGCCAGCACGTAATGGT
GGGAACTCAAGGGAGACTGCCGGTGACAAACCGGAGGAAGGTGGGGATGACGTCAAG
TCATCATGGCCCTTACGAGTAGGGCTACACACGTGCTACAATGGCAGATACAAAGTGAA
GCGAACTCGCGAGAGCAAGCGGACCACATAAAGTCTGTCGTAGTCCGGATTGGAGTCT
GCAACTCGACTCCATGAAGTCGGAATCGCTAGTAATCGTAGATCAGAATGCTACGGTGA
ATACGTTCCCGGGCCTTGTACACACCGCCCGTCACACCATGGGAGTGGGTTGCAAAAG
AAGTAGGTAGCTTAACCTTCGGGAGGGCGCTTACCACTTTGTGATTCATGACTGGGG
pattern 401

CGCTGGCGGCAGGCCTAACACATGCAAGTCGAGCGGCAGCGGAAAGTAGCTTGCTACT
TTGCCGGCGAGCGGCGGACGGGTGAGTAATGTCTGGGGATCTGCCTGATGGAGGGGGA
TAACTACTGGAAACGGTAGCTAATACCGCATGACCTCGAAAGAGCAAAGTGGGGGACC
TTCGGGCCTCACGCCATCGGATGAACCCAGATGGGATTAGCTAGTAGGTGGGGTAATGG
CTCACCTAGGCGACGATCCTTAGCTGGTCTGAGAGGATGACCAGCCACACTGGAACTG
AGACACGGTCCAGACTCCTACGGGAGGCAGCAGTGGGGAATATTGCACAATGGGCGCA
AGCCTGATGCAGCCATGCCGCGTGTGTGAAGAAGGCCTTCGGGTTGTAAAGCACTTTCA
GCGAGGAGGAAGGCATTGTGGTTAATAACCGCAGTGATTGACGTTACTCGCAGAAGAA
GCACCGGCTAACTCCGTGCCAGCAGCCGCGGTAATACGGAGGGTGCAAGCGTTAATCG
GAATTACTGGGCGTAAAGCGCACGCAGGCGGTTTGTTAAGTCAGATGTGAAATCCCCGC
GCTTAACGTGGGAACTGCATTTGAAACTGGCAAGCTAGAGTCTTGTAGAGGGGGGTAG
AATTCCAGGTGTAGCGGTGAAATGCGTAGAGATCTGGAGGAATACCGGTGGCGAAGGC
GGCCCCCTGGACAAAGACTGACGCTCAGGTGCGAAAGCGTGGGGAGCAAACAGGATT
AGATACCCTGGTAGTCCACGCTGTAAACGATGTCGACTTGGAGGTTGTGCCCTTGAGGC
GTGGCTTCCGGAGCTAACGCGTTAAGTCGTCCGCCTGGGGAGTACGGCCGCAAGGTTA
AAACTCAAATGAATTGACGGGGGCCCGCACAAGCGGTGGAGCATGTGGTTTAATTCGAT
GCAACGCGAAGAACCTTACCTACTCTTGACATCCACAGAACTTAGCAGAGATGCTTCGG
TGCCTTCGGGAACTGTGAGACAGGTGCTGCATGGCTGTCGTCAGCTCGTGTTGTGAAAT
GTTGGGTTAAGTCCCGCAACGAGCGCAACCCTTATCCTTTGTTGCCAGCACGTAATGGT
GGGAACTCAAGGGAGACTGCCGGTGACAAACCGGAGGAAGGTGGGGATGACGTCAAG
TCATCATGGCCCTTACGAGTAGGGCTACACACGTGCTACAATGGCAGATACAAAGTGAA
GCGAACTCGCGAGAGCAAGCGGACCACATAAAGTCTGTCGTAGTCCGGATTGGAGTCT
GCAACTCGACTCCATGAAGTCGGAATCGCTAGTAATCGTAGATCAGAATGCTACGGTGA
ATACGTTCCCGGGCCTTGTACACACCGCCCGTCACACCATGGGAGTAGGTTGCAAAAGA
AGTAGGTAGCTTAACCTTCGGGAGGGCGCTTACCACTTTGTGATTCATGACTGGGG
pattern 402
CGCTGGCGGCAGGCCTAACACATGCAAGTCGAGCGGCAGCGGAAAGTAGCTTGCTACT
TTGCCGGCGAGCGGCGGACGGGTGAGTAATGTCTGGGGATCTGCCTGATGGAGGGGGA
TAACTACTGGAAACGGTAGCTAATACCGCATGACCTCGAAAGAGCAAAGTGGGGGACC
TTCGGGCCTCACGCCATCGGATGAACCCAGATGGGATTAGCTAGTAGGTGGGGTAATGG
CTCACCTAGGCGACGATCCCTAGCTGGTCTGAGAGGATGACCAGCCACACTGGAACTG
AGACACGGTCCAGACTCCTACGGGAGGCAGCAGTGGGGAATATTGCACAATGGGCGCA
AGCCTGATGCAGCCATGCCGCGTGTGTGAAGAAGGCCTTCGGGTTGTAAAGCACTTTCA
GCGAGGAGGAAGGCATTGTGGTTAATAACCGCAGTGATTGACGTTACTCGCAGAAGAA
GCACCGGCTAACTCCGTGCCAGCAGCCGCGGTAATACGGAGGGTGCAAGCGTTAATCG
GAATTACTGGGCGTAAAGCGCACGCAGGCGGTTTGTTAAGTCAGATGTGAAATCCCCGC
GCTTAACGTGGGAACTGCATTTGAAACTGGCAAGCTAGAGTCTTGTAGAGGGGGGTAG
AATTCCAGGTGTAGCGGTGAAATGCGTAGAGATCTGGAGGAATACCGGTGGCGAAGGC
GGCCCCCCGGACAAAGACTGACGCTCAGGTGCGAAAGCGTGGGGAGCAAACAGGATT
AGATACCCTGGTAGTCCACGCTGTAAACGATGTCGACTTGGAGGTTGTGCCCTTGAGGC
GTGGCTTCCGGAGCTAACGCGTTAAGTCGACCGCCTGGGGAGTACGGCCGCAAGGTTA
AAACTCAAATGAATTGACGGGGGCCCGCACAAGCGGTGGAGCATGTGGTTTAATTCGAT
GCAACGCGAAGAACCTTACCTACTCTTGACATCCACAGAACTTAGCAGAGATGCTTCGG
TGCCTTCGGGAACTGTGAGACAGGTGCTGCATGGCTGTCGTCAGCTCGTGTTGTGAAAT

GTTGGGTTAAGTCCCGCAACGAGCGCAACCCTTATCCTTTGTTGCCAGCACGTAATGGT
GGGAACTCAAGGGAGACTGCCGGTGACAAACCGGAGGAAGGTGGGGATGACGTCAAG
TCATCATGGCCCTTACGAGTAGGGCTACACACGTGCTACAATGGCAGATACAAAGTGAA
GCGAACTCGCGAGAGCAAGCGGACCACATAAAGTCTGTCGTAGTCCGGATTGGAGTCT
GCAACTCGACTCCATGAAGTCGGAATCGCTAGTAATCGTAGATCAGAATGCTACGGTGA
ATACGTTCCCGGGCCTTGTACACACCGCCCGTCACACCATGGGAGTGGGTTGCAAAAG
AAGTAGGTAGCTTAACCTTCGGGAGGGCGCTTACCACTTTGTGATTCATGACTGGGG
pattern 403
CGCTGGCGGCAGGCCTAACACATGCAAGTCGAGCGGCAGCGGGAAGTAGCTTGCTACT
TTGCCGGCGAGCGGCGGACGGGTGAGTAATGTCTGGGAAACTGCCTGATGGAGGGGGA
TAACTACTGGAAACGGTAGCTAATACCGCATGACCTCGCAAGAGCAAAGTGGGGGACC
TTCGGGCCTCACGCCATCGGATGTGCCCAGATGGGATTAGCTAGTAGGTGGGGTAATGG
CTCACCTAGGCGACGATCCCTAGCTGGTCTGAGAGGATGACCAGCCACACTGGAACTG
AGACACGGTCCAGACTCCTACGGGAGGCAGCAGTGGGGAATATTGCACAATGGGCGCA
AGCCTGATGCAGCCATGCCGCGTGTGTGAAGAAGGCCTTCGGGTTGTAAAGCACTTTCA
GCGAGGAGGAAGGGGTTGAGTTTAATACGCTCAATCATTGACGTTACTCGCAGAAGAA
GCACCGGCTAACTCCGTGCCAGCAGCCGCGGTAATACGGAGGGTGCAAGCGTTAATCG
GAATTACTGGGCGTAAAGCGCACGCAGGCGGTTTGTTAAGTCAGATGTGAAATCCCCGC
GCTTAACGTGGGAACTGCATTTGAAACTGGCAAGCTAGAGTCTTGTAGAGGGGGGTAG
AATTCCAGGTGTAGCGGTGAAATGCGTAGAGATCTGGAGGAATACCGGTGGCGAAGGC
GGCCCCCTGGACAAAGACTGACGCTCAGGTGCGAAAGCGTGGGGAGCAAACAGGATT
AGATACCCTGGTAGTCCACGCTGTAAACGATGTCGACTTGGAGGTTGTGCCCTTGAGGC
GTGGCTTCCGGAGCTAACGCGTTAAGTCGACCGCCTGGGGAGTACGGCCGCAAGGTTA
AAACTCAAATGAATTGACGGGGGCCCGCACAAGCGGTGGAGCATGTGGTTTAATTCGAT
GCAACGCGAAGAACCTTACCTACTCTTGACATCCACGGAATTTAGCAGAGATGCTTTAG
TGCCTTCGGGAACCGTGAGACAGGTGCTGCATGGCTGTCGTCAGCTCGTGTTGTGAAAT
GTTGGGTTAAGTCCCGCAACGAGCGCAACCCTTATCCTTTGTTGCCAGCACGTCATGGT
GGGAACTCAAAGGAGACTGCCGGTGATAAACCGGAGGAAGGTGGGGATGACGTCAAG
TCATCATGGCCCTTACGAGTAGGGCTACACACGTGCTACAATGGCAGATACAAAGTGAA
GCGAACTCGCGAGAGCAAGCGGACCACATAAAGTCTGTCGTAGTCCGGATTGGAGTCT
GCAACTCGACTCCATGAAGTCGGAATCGCTAGTAATCGTAGATCAGAATGCTACGGTGA
ATACGTTCCCGGGCCTTGTACACACCGCCCGTCACACCATGGGAGTGGGTTGCAAAAG
AAGTAGGTAGCTTAACCTTCGGGAGGGCGCTTACCACTTTGTGATTCATGACTGGGG
pattern 404
CGCTGGCGGCAGGCCTAACACATGCAAGTCGAGCGGCAGCGGGAAGTAGTTTACTACT
TTGCCGGCGAGCGGCGGACGGGTGAGTAATGTCTGGGAAATTGCCTGATGGAGGGGGA
TAACTACTGGAAACGGTAGCTAATACCGCATGACCTCGTAAGAGCAAAGTGGGGGACCT
TCGGGCCTCACGCCATCGGATGTGCCCAGATGGGATTAGCTAGTAGGTGGGGTAATGGC
TCACCTAGGCGACGATCCCTAGCTGGTCTGAGAGGATGACCAGCCACACTGGAACTGA
GACACGGTCCAGACTCCTACGGGAGGCAGCAGTGGGGAATATTGCACAATGGGCGCAA
GCCTGATGCAGCCATGCCGCGTGTGTGAAGAAGGCCTTCGGGTTGTAAAGCACTTTCAG
CGAGGAGGAAGGCAGTCGTGTTAATAGCACGATTGATTGACGTTACTCGCAGAAGAAG
CACCGGCTAACTCCGTGCCAGCAGCCGCGGTAATACGGAGGGTGCAAGCGTTAATCGG
AATTACTGGGCGTAAAGCGCACGCAGGCGGTTTGTTAAGTCAGATGTGAAATCCCCGCG

CTTAACGTGGGAACTGCATTTGAAACTGGCAAGCTAGAGTCTTGTAGAGGGGGGTAGA
ATTCCAGGTGTAGCGGTGAAATGCGTAGAGATCTGGAGGAATACCGGTGGCGAAGGCG
GCCCCCTGGACAAAGACTGACGCTCAGGTGCGAAAGCGTGGGGAGCAAACAGGATTA
GATACCCTGGTAGTCCACGCTGTAAACGATGTCGACTTGGAGGTTGTGCCCTTGAGGCG
TGGCTTCCGGAGCTAACGCGTTAAGTCGACCGCCTGGGGAGTACGGCCGCAAGGTTAA
AACTCAAATGAATTGACGGGGGCCCGCACAAGCGGTGGAGCATGTGGTTTAATTCGATG
CAACGCGAAGAACCTTACCTACTCTTGACATCCACGGAATTTAGCAGAGATGCTTTAGT
GCCTTCGGGAACCGTGAGACAGGTGCTGCATGGCTGTCGTCAGCTCGTGTTGTGAAATG
TTGGGTTAAGTCCCGCAACGAGCGCAACCCTTATCCTTTGTTGCCAGCACGTAATGGTG
GGAACTCAAGGGAGACTGCCGGTGACAAACCGGAGGAAGGTGGGGATGACGTCAAGT
CATCATGGCCCTTACGAGTAGGGCTACACACGTGCTACAATGGCAGATACAAAGTGAAG
CGAACTCGCGAGAGCAAGCGGACCACATAAAGTCTGTCGTAGTCCGGATTGGAGTCTG
CAACTCGACTCCATGAAGTCGGAATCGCTAGTAATCGTAGATCAGAATGCTACGGTGAAT
ACGTTCCCGGGCCTTGTACACACCGCCCGTCACACCATGGGAGTGGGTTGCAAAAGAA
GTAGGTAGCTTAACCTTCGGGAGGGCGCTTACCACTTTGTGATTCATGACTGGGG
pattern 405
CGCTGGCGGCAGGCCTAACACATGCAAGTCGAGCGGCAGCGGGAAGTAGTTTACTACT
TTGCCGGCGAGCGGCGGACGGGTGAGTAATGTCTGGGAAACTGCCTGATGGAGGGGGA
TAACTACTGGAAACGGTAGCTAATACCGCATGACCTCGTAAGAGCAAAGTGGGGGACCT
TCGGGCCTCACGCCATCGGATGTGCCCAGATGGGATTAGCTAGTAGGTGGGGTAATGGC
TCACCTAGGCGACGATCCCTAGCTGGTCTGAGAGGATGACCAGCCACACTGGAACTGA
GACACGGTCCAGACTCCTACGGGAGGCAGCAGTGGGGAATATTGCACAATGGGCGCAA
GCCTGATGCAGCCATGCCGCGTGTGTGAAGAAGGCCTTCGGGTTGTAAAGCACTTTCAG
CGAGGAGGAAGGCAGTCGTGTTAATAGCACGGTTGATTGACATTACTCGCAGAAGAAG
CACCGGCTAACTCCGTGCCAGCAGCCGCGGTAATACGGAGGGTGCAAGCGTTAATCGG
AATTACTGGGCGTAAAGCGCACGCAGGCGGTTTGTTAAGTCAGATGTGAAATCCCCGCG
CTTAACGTGGGAACTGCATTTGAAACTGGCAAGCTAGAGTCTTGTAGAGGGGGGTAGA
ATTCCAGGTGTAGCGGTGAAATGCGTAGAGATCTGGAGGAATACCGGTGGCGAAGGCG
GCCCCCTGGACAAAGACTGACGCTCAGGTGCGAAAGCGTGGGGAGCAAACAGGATTA
GATACCCTGGTAGTCCACGCTGTAAACGATGTCGACTTGGAGGTTGTGCCCTTGAGGCG
TGGCTTCCGGAGCTAACGCGTTAAGTCGACCGCCTGGGGAGTACGGCCGCAAGGTTAA
AACTCAAATGAATTGACGGGGGCCCGCACAAGCGGTGGAGCATGTGGTTTAATTCGATG
CAACGCGAAGAACCTTACCTACTCTTGACATCCACAGAACTTAGCAGAGATGCTTCGGT
GCCTTCGGGAACTGTGAGACAGGTGCTGCATGGCTGTCGTCAGCTCGTGTTGTGAAATG
TTGGGTTAAGTCCCGCAACGAGCGCAACCCTTATCCTTTGTTGCCAGCACGTAATGGTG
GGAACTCAAGGGAGACTGCCGGTGACAAATCGGAGGAAGGTGGGGATGACGTCAAGT
CATCATGGCCCTTACGAGTAGGGCTACACACGTGCTACAATGGCAGATACAAAGTGAAG
CGAACTCGCGAGAGCAAGCGGACCACATAAAGTCTGTCGTAGTCCGGATTGGAGTCTG
CAACTCGACTCCATGAAGTCGGAATCGCTAGTAATCGTAGATCAGAATGCTACGGTGAAT
ACGTTCCCGGGCCTTGTACACACCGCCCGTCACACCATGGGAGTGGGTTGCAAAAGAA
GTAGGTAGCTTAACCTTCGGGAGGGCGCTTACCACTTTGTGATTCATGACTGGGG
pattern 406
CGCTGGCGGCAGGCCTAACACATGCAAGTCGAGCGGCAGCGGGAAGTAGTTTACTACT
TTGCCGGCGAGCGGCGGACGGGTGAGTAATGTCTGGGAAACTGCCTGATGGAGGGGGA

TAACTACTGGAAACGGTAGCTAATACCGCATAACGTCTACGGACCAAAGTGGGGGACCT
TCGGGCCTCACGCCATCGGATGTGCCCAGATGGGATTAGCTAGTAGGTGGGGTAATGGC
TCACCTAGGCGACGATCCCTAGCTGGTCTGAGAGGATGACCAGCCACACTGGAACTGA
GACACGGTCCAGACTCCTACGGGAGGCAGCAGTGGGGAATATTGCACAATGGGCGCAA
GCCTGATGCAGCCATGCCGCGTGTGTGAAGAAGGCCTTCGGGTTGTAAAGCACTTTCAG
CGAGGAGGAAGGCATAAAGGTTAATAACCTTTGTGATTGACGTTACTCGCAGAAGAAG
CACCGGCTAACTCCGTGCCAGCAGCCGCGGTAATACGGAGGGTGCAAGCGTTAATCGG
AATTACTGGGCGTAAAGCGCACGCAGGCGGTTTGTTAAGTCAGATGTGAAATCCCCGCG
CTTAACGTGGGAACTGCATTTGAAACTGGCAAGCTAGAGTCTTGTAGAGGGGGGTAGA
ATTCCAGGTGTAGCGGTGAAATGCGTAGAGATCTGGAGGAATACCGGTGGCGAAGGCG
GCCCCCTGGACAAAGACTGACGCTCAGGTGCGAAAGCGTGGGGAGCAAACAGGATTA
GATACCCTGGTAGTCCACGCTGTAAACGATGTCGACTTGGAGGTTGTGCCCTTGAGGCG
TGGCTTCCGGAGCTAACGCGTTAAGTCGACCGCCTGGGGAGTACGGCCGCAAGGTTAA
AACTCAAATGAATTGACGGGGGCCCGCACAAGCGGTGGAGCATGTGGTTTAATTCGATG
CAACGCGAAGAACCTTACCTACTCTTGACATCCACAGAACTTAGCAGAGATGCTTCGGT
GCCTTCGGGAACTGTGAGACAGGTGCTGCATGGCTGTCGTCAGCTCGTGTTGTGAAATG
TTGGGTTAAGTCCCGCAACGAGCGCAACCCTTATCCTTTGTTGCCAGCACGTAATGGTG
GGAACTCAAGGGAGACTGCCGGTGACAAACCGGAGGAAGGTGGGGATGACGTCAAGT
CATCATGGCCCTTACGAGTAGGGCTACACACGTGCTACAATGGCAGATACAAAGTGAAG
CGAACTCGCGAGAGCAAGCGGACCACATAAAGTCTGTCGTAGTCCGGATTGGAGTCTG
CAACTCGACTCCATGAAGTCGGAATCGCTAGTAATCGTAGATCAGAATGCTACGGTGAAT
ACGTTCCCGGGCCTTGTACACACCGCCCGTCACACCATGGGAGTGGGTTGCAAAAGAA
GTAGGTAGCTTAACCTTCGGGAGGGCGCTTACCACTTTGTGATTCATGACTGGGG
pattern 407
CGCTGGCGGCAGGCCTAACACATGCAAGTCGAGCGGCAGCGGGGAGTAGTTTACTACT
CTGCCGGCGAGCGGCGGACGGGTGAGTAATGTCTGGGGATCTGCCTGATGGAGGGGGA
TAACTACTGGAAACGGTAGCTAATACCGCATGACCTCGCAAGAGCAAAGTGGGGGACC
TTCGGGCCTCACGCCATCGGATGAACCCAGATGGGATTAGCTAGTAGGTGAGGTAATGG
CTCACCTAGGCGACGATCCCTAGCTGGTCTGAGAGGATGACCAGCCACACTGGAACTG
AGACACGGTCCAGACTCCTACGGGAGGCAGCAGTGGGGAATATTGCACAATGGGCGCA
AGCCTGATGCAGCCATGCCGCGTGTGTGAAGAAGGCCTTCGGGTTGTAAAGCACTTTCA
GCGAGGAGGAAGGCAGTCGTGTTAATAGCACGATTGATTGACGTTACTCGCAGAAGAA
GCACCGGCTAACTCCGTGCCAGCAGCCGCGGTAATACGGAGGGTGCAAGCGTTAATCG
GAATTACTGGGCGTAAAGCGCACGCAGGCGGTTTGTTAAGTCAGATGTGAAATCCCCGC
GCTTAACGTGGGAACTGCATTTGAAACTGGCAAGCTAGAGTCTTGTAGAGGGGGGTAG
AATTCCAGGTGTAGCGGTGAAATGCGTAGAGATCTGGAGGAATACCGGTGGCGAAGGC
GGCCCCCTGGACAAAGACTGACGCTCAGGTGCGAAAGCGTGGGGAGCAAACAGGATT
AGATACCCTGGTAGTCCACGCTGTAAACGATGTCGACTTGGAGGTTGTGCCCTTGAGGC
GTGGCTTCCGGAGCTAACGCGTTAAGTCGACCGCCTGGGGAGTACGGCCGCAAGGTTA
AAACTCAAATGAATTGACGGGGGCCCGCACAAGCGGTGGAGCATGTGGTTTAATTCGAT
GCAACGCGAAGAACCTTACCTACTCTTGACATCCACGGAATTTAGCAGAGATGCTTAAG
TGCCTTCGGGAACCGTGAGACAGGTGCTGCATGGCTGTCGTCAGCTCGTGTTGTGAAAT
GTTGGGTTAAGTCCCGCAACGAGCGCAACCCTTATCCTTTGTTGCCAGCACGTAATGGT
GGGAACTCAAGGGAGACTGCCGGTGACAAACCGGAGGAAGGTGGGGATGACGTCAAG

TCATCATGGCCCTTACGAGTAGGGCTACACACGTGCTACAATGGCAGATACAAAGTGAA
GCGAACTCGCGAGAGCAAGCGGACCACATAAAGTCTGTCGTAGTCCGGATTGGAGTCT
GCAACTCGACTCCATGAAGTCGGAATCGCTAGTAATCGTAGATCAGAATGCTACGGTGA
ATACGTTCCCGGGCCTTGTACACACCGCCCGTCACACCATGGGAGTGGGTTGCAAAAG
AAGTAGGTAGCTTAACCTTCGGGAGGGCGCTTACCACTTTGTGATTCATGACTGGGG

pattern 408

CGCTGGCGGCAGGCCTAACACATGCAAGTCGAGCGGCAGCGGGAAGTAGTTTACTACT
TTGCCGGCGAGCGGCGGACGGGTGAGTAATGTCTGGGAAACTGCCTGATGGAGGGGGA
TAACTACTGGAAACGGTAGCTAATACCGCATAACGTCTTCGGACCAAAGTGGGGGACCT
TCGGGCCTCACGCCATCGGATGTGCCCAGATGGGATTAGCTAGTAGGTGGGGTAATGGC
TCACCTAGGCGACGATCCCTAGCTGGTCTGAGAGGATGACCAGCCACACTGGAACTGA
GACACGGTCCAGACTCCTACGGGAGGCAGCAGTGGGGAATATTGCACAATGGGCGCAA
GCCTGATGCAGCCATGCCGCGTGTGTGAAGAAGGCCTTCGGGTTGTAAAGCACTTTCAG
CGAGGAGGAAGGCATAAAGGTTAATAACCTTTGTGATTGACGTTACTCGCAGAAGAAG
CACCGGCTAACTCCGTGCCAGCAGCCGCGGTAATACGGAGGGTGCAAGCGTTAATCGG
AATTACTGGGCGTAAAGCGCACGCAGGCGGTTTGTTAAGTCAGATGTGAAATCCCCGCG
CTTAACGTGGGAACTGCATTTGAAACTGGCAAGCTAGAGTCTTGTAGAGGGGGGTAGA
ATTCCAGGTGTAGCGGTGAAATGCGTAGAGATCTGGAGGAATACCGGTGGCGAAGGCG
GCCCCCTGGACAAAGACTGACGCTCAGGTGCGAAAGCGTGGGGAGCAAACAGGATTA
GATACCCTGGTAGTCCACGCTGTAAACGATGTCGACTTGGAGGTTGTGCCCTTGAGGCG
TGGCTTCCGGAGCTAACGCGTTAAGTCGACCGCCTGGGGAGTACGGCCGCAAGGTTAA
AACTCAAATGAATTGACGGGGGCCCGCACAAGCGGTGGAGCATGTGGTTTAATTCGATG
CAACGCGAAGAACCTTACCTACTCTTGACATCCACGGAATTTAGCAGAGATGCTTTATTA
GTGCCTTCGGGAACCGTGAGACAGGTGCTGCATGGCTGTCGTCAGCTCGTGTTGTGAAA
TGTTGGGTTAAGTCCCGCAACGAGCGCAACCCTTATCCTTTGTTGCCAGCACGTGATGG
TGGGAACTCAAAGGAGACTGCTGGTGATAAACCGGAGGAAGGTGGGGATGACGTCAA
GTCATCATGGCCCTTACGAGTAGGGCTACACACGTGCTACAATGGCAGATACAAAGTGA
AGCGAACTCGCGAGAGCAAGCGGACCACATAAAGTCTGTCGTAGTCCGGATTGGAGTC
TGCAACTCGACTCCATGAAGTCGGAATCGCTAGTAATCGTAGATCAGAATGCTACGGTG
AATACGTTCCCGGGCCTTGTACACACCGCCCGTCACACCATGGGAGTGGGTTGCAAAA
GAAGTAGGTAGCTTAACCTTCGGGAGGGCGCTTACCACTTTGTGATTCATGACTGGGG

pattern 409

CGCTGGCGGCAGGCCTAACACATGCAAGTCGAGCGGCAGCGGGAAGTAGTTTACTACT
TTGCCGGCGAGCGGCGGACGGGTGAGTAATGTCTGGGAAACTGCCTGATGGAGGGGGA
TAACTACTGGAAACGGTAGCTAATACCGCATAACGTCTTCGGACCAAAGTGGGGGACCT
TCGGGCCTCACGCCATCGGATGTGCCCAGATGGGATTAGCTAGTAGGTGGGGTAATGGC
TCACCTAGGCGACGATCCCTAGCTGGTCTGAGAGGATGACCAGCCACACTGGAACTGA
GACACGGTCCAGACTCCTACGGGAGGCAGCAGTGGGGAATATTGCACAATGGGCGCAA
GCCTGATGCAGCCATGCCGCGTGTGTGAAGAAGGCCTTCGGGTTGTAAAGCACTTTCAG
CGAGGAGGAAGGCATAAAGGTTAATAACCTTTGTGATTGACGTTACTCGCAGAAGAAG
CACCGGCTAACTCCGTGCCAGCAGCCGCGGTAATACGGAGGGTGCAAGCGTTAATCGG
AATTACTGGGCGTAAAGCGCACGCAGGCGGTTTGTTAAGTCAGATGTGAAATCCCCGCG
CTTAACGTGGGAACTGCATTTGAAACTGGCAAGCTAGAGTCTTGTAGAGGGGGGTAGA
ATTCCAGGTGTAGCGGTGAAATGCGTAGAGATCTGGAGGAATACCGGTGGCGAAGGCG

GCCCCCTGGACAAAGACTGACGCTCAGGTGCGAAAGCGTGGGGAGCAAACAGGATTA
GATACCCTGGTAGTCCACGCTGTAAACGATGTCGACTTGGAGGTTGTGCCCTTGAGGCG
TGGCTTCCGGAGCTAACGCGTTAAGTCGACCGCCTGGGGAGTACGGCCGCAAGGTTAA
AACTCAAATGAATTGACGGGGGCCCGCACAAGCGGTGGAGCATGTGGTTTAATTCGATG
CAACGCGAAGAACCTTACCTACTCTTGACATCCACGGAATTTAGCAGAGATGCTTTATTA
GTGCCTTCGGGAACCGTGAGACAGGTGCTGCATGGCTGTCGTCAGCTCGTGTTGTGAAA
TGTTGGGTTAAGTCCCGCAACGAGCGCAACCCTTATCCTTTGTTGCCAGCACGTGATGG
TGGGAACTCAAAGGAGACTGCCGGTGATAAACCGGAGGAAGGTGGGGATGACGTCAA
GTCATCATGGCCCTTACGAGTAGGGCTACACACGTGCTACAATGGCAGATACAAAGTGA
AGCGAACTCGCGAGAGCAAGCGGACCACATAAAGTCTGTCGTAGTCCGGATTGGAGTC
TGCAACTCGACTCCATGAAGTCGGAATCGCTAGTAATCGTAGATCAGAATGCTACGGTG
AATACGTTCCCGGGCCTTGTACACACCGCCCGTCACACCATGGGAGTGGGTTGCAAAA
GAAGTAGGTAGCTTAACCTTCGGGAGGGCGCTTACCACTTTGTGATTCATGACTGGGG
pattern 410
CGCTGGCGGCAGGCCTAACACATGCAAGTCGAGCGGCAGCGGGAAGTAGTTTACTACT
TTGCCGGCGAGCGGCGGACGGGTGAGTAATGTCTGGGAAACTGCCTGATGGAGGGGGA
TAACTACTGGAAACGGTAGCTAATACCGCATAACGTCTTCGGACCAAAGTGGGGGACCT
TCGGGCCTCACGCCATCGGATGTGCCCAGATGGGATTAGCTAGTAGGTGGGGTAATGGC
TCACCTAGGCGACGATCCCTAGCTGGTCTGAGAGGATGACCAGCCACACTGGAACTGA
GACACGGTCCAGACTCCTACGGGAGGCAGCAGTGGGGAATATTGCACAATGGGCGCAA
GCCTGATGCAGCCATGCCGCGTGTGTGAAGAAGGCCTTCGGGTTGTAAAGCACTTTCAG
CGAGGAGGAAGGCATAAAGGTTAATAACCTTTGTGATTGACGTTACTCGCAGAAGAAG
CACCGGCTAACTCCGTGCCAGCAGCCGCGGTAATACGGAGGGTGCAAGCGTTAATCGG
AATTACTGGGCGTAAAGCGCACGCAGGCGGTTTGTTAAGTCAGATGTGAAATCCCCGCG
CTTAACGTGGGAACTGCATTTGAAACTGGCAAGCTAGAGTCTTGTAGAGGGGGGTAGA
ATTCCAGGTGTAGCGGTGAAATGCGTAGAGATCTGGAGGAATACCGGTGGCGAAGGCG
GCCCCCTGGACAAAGACTGACGCTCAGGTGCGAAAGCGTGGGGAGCAAACAGGATTA
GATACCCTGGTAGTCCACGCTGTAAACGATGTCGACTTGGAGGTTGTGCCCTTGAGGCG
TGGCTTCCGGAGCTAACGCGTTAAGTCGACCGCCTGGGGAGTACGGCCGCAAGGTTAA
AACTCAAATGAATTGACGGGGGCCCGCACAAGCGGTGGAGCATGTGGTTTAATTCGATG
CAACGCGAAGAACCTTACCTACTCTTGACATCCACGGAATTTAGCAGAGATGCTTTAGT
GCCTTCGGGAACCGTGAGACAGGTGCTGCATGGCTGTCGTCAGCTCGTGTTGTGAAATG
TTGGGTTAAGTCCCGCAACGAGCGCAACCCTTATCCTTTGTTGCCAGCACGTGATGGTG
GGAACTCAAAGGAGACTGCCGGTGATAAACCGGAGGAAGGTGGGGATGACGTCAAGT
CATCATGGCCCTTACGAGTAGGGCTACACACGTGCTACAATGGCAGATACAAAGTGAAG
CGAACTCGCGAGAGCAAGCGGACCACATAAAGTCTGTCGTAGTCCGGATTGGAGTCTG
CAACTCGAGTCCATGAAGTCGGAATCGCTAGTAATCGTAGATCAGAATGCTACGGTGAA
TACGTTCCCGGGCCTTGTACACACCGCCCGTCACACCATGGGAGTGGGTTGCAAAAGA
AGTAGGTAGCTTAACCTTCGGGAGGGCGCTTACCACTTTGTGATTCATGACTGGGG
pattern 411
CGCTGGCGGCAGGCCTAACACATGCAAGTCGAGCGGCAGCGGGAAGTAGCTTGCTACT
TTGCCGGCGAGCGGCGGACGGGTGAGTAATGTCTGGGAAATTGCCTGATGGAGGGGGA
TAACTACTGGAAACGGTAGCTAATACCGCATGACCTCGAAAGAGCAAAGTGGGGGACC
TTCGGGCCTCACGCCATCGGATGTGCCCAGATGGGATTAGCTAGTAGGTGGGGTAATGG

CTCACCTAGGCGACGATCCCTAGCTGGTCTGAGAGGATGACCAGCCACACTGGAACTG
AGACACGGTCCAGACTCCTACGGGAGGCAGCAGTGGGGAATATTGCACAATGGGCGCA
AGCCTGATGCAGCCATGCCGCGTGTGTGAAGAAGGCCTTCGGGTTGTAAAGCACTTTCA
GCGAGGAGGAAGGCATTTCACTTAATACGTGAGGTGATTGACGTTACTCGCAGAAGAA
GCACCGGCTAACTCCGTGCCAGCAGCCGCGGTAATACGGAGGGTGCAAGCGTTAATCG
GAATTACTGGGCGTAAAGCGCACGCAGGCGGTTTGTTAAGTCAGATGTGAAATCCCCGA
GCTTAACTTGGGAACTGCATTTGAAACTGGCAAGCTAGAGTCTTGTAGAGGGGGGTAG
AATTCCAGGTGTAGCGGTGAAATGCGTAGAGATCTGGAGGAATACCGGTGGCGAAGGC
GGCCCCCTGGACAAAGACTGACGCTCAGGTGCGAAAGCGTGGGGAGCAAACAGGATT
AGATACCCTGGTAGTCCACGCTGTAAACGATGTCGACTTGGAGGTTGTGCCCTTGAGGC
GTGGCTTCCGGAGCTAACGCGTTAAGTCGACCGCCTGGGGAGTACGGCCGCAAGGTTA
AAACTCAAATGAATTGACGGGGGCCCGCACAAGCGGTGGAGCATGTGGTTTAATTCGAT
GCAACGCGAAGAACCTTACCTACTCTTGACATCCACAGAACTTAGCAGAGATGCTTAGG
TGCCTTCGGGAACTGTGAGACAGGTGCTGCATGGCTGTCGTCAGCTCGTGTTGTGAAAT
GTTGGGTTAAGTCCCGCAACGAGCGCAACCCTTATCCTTTGTTGCCAGCACGTAATGGT
GGGAACTCAAAGGAGACTGCCGGTGATAAACCGGAGGAAGGTGGGGATGACGTCAAG
TCATCATGGCCCTTACGAGTAGGGCTACACACGTGCTACAATGGCAGATACAAAGTGAA
GCGAACTCGCGAGAGCAAGCGGACCACATAAAGTCTGTCGTAGTCCGGATTGGAGTCT
GCAACTCGACTCCATGAAGTCGGAATCGCTAGTAATCGTAGATCAGAATGCTACGGTGA
ATACGTTCCCGGGCCTTGTACACACCGCCCGTCACACCATGGGAGTGGGTTGCAAAAG
AAGTAGGTAGCTTAACCTTCGGGAGGGCGCTTACCACTTTGTGATTCATGACTGGGG
pattern 412
CGCTGGCGGCAGGCCTAACACATGCAAGTCGAGCGGCAGCGGGAAGTAGTTTACTACT
TTGCCGGCGAGCGGCGGACGGGTGAGTAATGTCTGGGAAACTGCCTGATGGAGGGGGA
TAACTACTGGAAACGGTAGCTAATACCGCATGACCTCGTAAGAGCAAAGTGGGGGACCT
TCGGGCCTCACGCCATCGGATGTGCCCAGATGGGATTAGCTAGTAGGTGGGGTAATGGC
TCACCTAGGCGACGATCCCTAGCTGGTCTGAGAGGATGACCAGCCACACTGGAACTGA
GACACGGTCCAGACTCCTACGGGAGGCAGCAGTGGGGAATATTGCACAATGGGCGCAA
GCCTGATGCAGCCATGCCGCGTGTGTGAAGAAGGCCTTCGGGTTGTAAAGCACTTTCAG
CGAGGAGGAAGGCAGTCGTGTTAATAGCACGATTGATTGACGTTACTCGCAGAAGAAG
CACCGGCTAACTCCGTGCCAGCAGCCGCGGTAATACGGAGGGTGCAAGCGTTAATCGG
AATTACTGGGCGTAAAGCGCACGCAGGCGGTTTGTTAAGTCAGATGTGAAATCCCCGCG
CTTAACGTGGGAACTGCATTTGAAACTGGCAAGCTAGAGTCTTGTAGAGGGGGGTAGA
ATTCCAGGTGTAGCGGTGAAATGCGTAGAGATCTGGAGGAATACCGGTGGCGAAGGCG
GCCCCCTGGACAAAGACTGACGCTCAGGTGCGAAAGCGTGGGGAGCAAACAGGATTA
GATACCCTGGTAGTCCACGCTGTAAACGATGTCGACTTGGAGGTTGTGCCCTTGAGGCG
TGGCTTCCGGAGCTAACGCGTTAAGTCGACCGCCTGGGGAGTACGGCCGCAAGGTTAA
AACTCAAATGAATTGACGGGGGCCCGCACAAGCGGTGGAGCATGTGGTTTAATTCGATG
CAACGCGAAGAACCTTACCTACTCTTGACATCCACGGAATTTAGCAGAGATGCTTTAGT
GCCTTCGGGAACCGTGAGACAGGTGCTGCATGGCTGTCGTCAGCTCGTGTTGTGAAATG
TTGGGTTAAGTCCCGCAACGAGCGCAACCCTTATCCTTTGTTGCCAGCACGTAATGGTG
GGAACTCAAGGGAGACTGCCGGTGACAAACCGGAGGAAGGTGGGGATGACGTCAAGT
CATCATGGCCCTTACGAGTAGGGCTACACACGTGCTACAATGGCAGATACAAAGTGAAG
CGAACTCGCGAGAGCAAGCGGACCACATAAAGTCTGTCGTAGTCCGGATAGGAGTCTG

CAACTCGACTCCATGAAGTCGGAATCGCTAGTAATCGTAGATCAGAATGCTACGGTGAAT
ACGTTCCCGGGCCTTGTACACACCGCCCGTCACACCATGGGAGTGGGTTGCAAAAGAA
GTAGGTAGCTTAACCTTCGGGAGGGCGCTTACCACTTTGTGATTCATGACTGGGG
pattern 413
CGCTGGCGGCAGGCCTAACACATGCAAGTCGAGCGGCAGCGGAAAGTAGCTTGCTACT
TTGCCGGCGAGCGGCGGACGGGTGAGTAATGTCTGGGAAACTGCCTGATGGAGGGGGA
TAACTACTGGAAACGGTAGCTAATACCGCATGACCTCGCAAGAGCAAAGTGGGGGACC
TTAGGGCCTCACGCCATCGGATGTGCCCAGATGGGATTAGCTAGTAGGTGGGGTAATGG
CTCACCTAGGCGACGATCCCTAGCTGGTCTGAGAGGATGACCAGCCACACTGGAACTG
AGACACGGTCCAGACTCCTACGGGAGGCAGCAGTGGGGAATATTGCACAATGGGCGCA
AGCCTGATGCAGCCATGCCGCGTGTGTGAAGAAGGCCTTCGGGTTGTAAAGCACTTTCA
GCGAGGAGGAAGGCAGTCGTGTTAATAGCACGATTGATTGACGTTACTCGCAGAAGAA
GCACCGGCTAACTCCGTGCCAGCAGCCGCGGTAATACGGAGGGTGCAAGCGTTAATCG
GAATTACTGGGCGTAAAGCGCACGCAGGCGGTTTGTTAAGTCAGATGTGAAATCCCCGC
GCTTAACGTGGGAACTGCATTTGAAACTGGCAAGCTAGAGTCTTGTAGAGGGGGGTAG
AATTCCAGGTGTAGCGGTGAAATGCGTAGAGATCTGGAGGAATACCGGTGGCGAAGGC
GGCCCCCTGGACAAAGACTGACGCTCAGGTGCGAAAGCGTGGGGAGCAAACAGGATT
AGATACCCTGGTAGTCCACGCTGTAAACGATGTCGACTTGGAGGTTGTGCCCTTGAGGC
GTGGCTTCCGGAGCTAACGCGTTAAGTCGACCGCCTGGGGAGTACGGCCGCAAGGTTA
AAACTCAAATGAATTGACGGGGGCCCGCACAAGCGGTGGAGCATGTGGTTTAATTCGAT
GCAACGCGAAGAACCTTACCTACTCTTGACATCCACAGAACTTAGCAGAGATGCTTCGG
TGCCTTCGGGAACTGTGAGACAGGTGCTGCATGGCTGTCGTCAGCTCGTGTTGTGAAAT
GTTGGGTTAAGTCCCGCAACGAGCGCAACCCTTATCCTTTGTTGCCAGCACGTAATGGT
GGGAACTCAAGGGAGACTGCCGGTGACAAACCGGAGGAAGGTGGGGATGACGTCAAG
TCATCATGGCCCTTACGAGTAGGGCTACACACGTGCTACAATGGCAGATACAAAGTGAA
GCGAACTCGCGAGAGTCAGCGGACCACATAAAGTCTGTCGTAGTCCGGATTGGAGTCT
GCAACTCGACTCCATGAAGTCGGAATCGCTAGTAATCGTAGATCAGAATGCTACGGTGA
ATACGTTCCCGGGCCTTGTACACACCGCCCGTCACACCATGGGAGTGGGTTGCAAAAG
AAGTAGGTAGCTTAACCTTCGGGAGGGCGCTTACCACTTTGTGATTCATGACTGGGG
pattern 414
CGCTGGCGGCAGGCCTAACACATGCAAGTCGAGCGGCAGCGGAAAGTAGTTTACTACT
TTGCCGGCGAGCGGCGGACGGGTGAGTAATGTCTGGGAAACTGCCTGATGGAGGGGGA
TAACTACTGGAAACGGTAGCTAATACCGCATGACCTCGCAAGAGCAAAGTGGGGGACC
TTAGGGCCTCACGCCATCGGATGTGCCCAGATGGGATTAGCTAGTAGGTGGGGTAATGG
CTCACCTAGGCGACGATCCCTAGCTGGTCTGAGAGGATGACCAGCCACACTGGAACTG
AGACACGGTCCAGACTCCTACGGGAGGCAGCAGTGGGGAATATTGCACAATGGGCGCA
AGCCTGATGCAGCCATGCCGCGTGTGTGAAGAAGGCCTTCGGGTTGTAAAGCACTTTCA
GCGAGGAGGAAGGCAGTCGTGTTAATAGCACGATTGATTGACGTTACTCGCAGAAGAA
GCACCGGCTAACTCCGTGCCAGCAGCCGCGGTAATACGGAGGGTGCAAGCGTTAATCG
GAATTACTGGGCGTAAAGCGCACGCAGGCGGTTTGTTAAGTCAGATGTGAAATCCCCGC
GCTTAACGTGGGAACTGCATTTGAAACTGGCAAGCTAGAGTCTTGTAGAGGGGGGTAG
AATTCCAGGTGTAGCGGTGAAATGCGTAGAGATCTGGAGGAATACCGGTGGCGAAGGC
GGCCCCCTGGACAAAGACTGACGCTCAGGTGCGAAAGCGTGGGGAGCAAACAGGATT
AGATACCCTGGTAGTCCACGCTGTAAACGATGTCGACTTGGAGGTTGTGCCCTTGAGGC

GTGGCTTCCGGAGCTAACGCGTTAAGTCGACCGCCTGGGGAGTACGGCCGCAAGGTTA
AAACTCAAATGAATTGACGGGGGCCCGCACAAGCGGTGGAGCATGTGGTTTAATTCGAT
GCAACGCGAAGAACCTTACCTACTCTTGACATCCACAGAACTTAGCAGAGATGCTTCGG
TGCCTTCGGGAACTGTGAGACAGGTGCTGCATGGCTGTCGTCAGCTCGTGTTGTGAAAT
GTTGGGTTAAGTCCCGCAACGAGCGCAACCCTTATCCTTTGTTGCCAGCACGTAATGGT
GGGAACTCAAGGGAGACTGCCGGTGACAAACCGGAGGAAGGTGGGGATGACGTCAAG
TCATCATGGCCCTTACGAGTAGGGCTACACACGTGCTACAATGGCAGATACAAAGTGAA
GCGAACTCGCGAGAGTCAGCGGACCACATAAAGTCTGTCGTAGTCCGGATTGGAGTCT
GCAACTCGACTCCATGAAGTCGGAATCGCTAGTAATCGTAGATCAGAATGCTACGGTGA
ATACGTTCCCGGGCCTTGTACACACCGCCCGTCACACCATGGGAGTGGGTTGCAAAAG
AAGTAGGTAGCTTAACCTTCGGGAGGGCGCTTACCACTTTGTGATTCATGACTGGGG

pattern 415

CGCTGGCGGCAGGCCTAACACATGCAAGTCGAGCGGCAGCGGAAAGTAGTTTACTACT
TTGCCGGCGAGCGGCGGACGGGTGAGTAATGTCTGGGAAACTGCCTGATGGAGGGGGA
TAACTACTGGAAACGGTAGCTAATACCGCATGACCTCGCAAGAGCAAAGTGGGGGACC
TTCGGGCCTCACGCCATCGGATGTGCCCAGATGGGATTAGCTAGTAGGTGGGGTAATGG
CTCACCTAGGCGACGATCCCTAGCTGGTCTGAGAGGATGACCAGCCACACTGGAACTG
AGACACGGTCCAGACTCCTACGGGAGGCAGCAGTGGGGAATATTGCACAATGGGCGCA
AGCCTGATGCAGCCATGCCGCGTGTGTGAAGAAGGCCTTCGGGTTGTAAAGCACTTTCA
GCGAGGAGGAAGGCAGTCGTGTTAATAGCACGATTGATTGACGTTACTCGCAGAAGAA
GCACCGGCTAACTCCGTGCCAGCAGCCGCGGTAATACGGAGGGTGCAAGCGTTAATCG
GAATTACTGGGCGTAAAGCGCACGCAGGCGGTTTGTTAAGTCAGATGTGAAATCCCCGC
GCTTAACGTGGGAACTGCATTTGAAACTGGCAAGCTAGAGTCTTGTAGAGGGGGGTAG
AATTCCAGGTGTAGCGGTGAAATGCGTAGAGATCTGGAGGAATACCGGTGGCGAAGGC
GGCCCCCTGGACAAAGACTGACGCTCAGGTGCGAAAGCGTGGGGAGCAAACAGGATT
AGATACCCTGGTAGTCCACGCTGTAAACGATGTCGACTTGGAGGTTGTGCCCTTGAGGC
GTGGCTTCCGGAGCTAACGCGTTAAGTCGACCGCCTGGGGAGTACGGCCGCAAGGTTA
AAACTCAAATGAATTGACGGGGGCCCGCACAAGCGGTGGAGCATGTGGTTTAATTCGAT
GCAACGCGAAGAACCTTACCTACTCTTGACATCCACAGAACTTAGCAGAGATGCTTCGG
TGCCTTCGGGAACTGTGAGACAGGTGCTGCATGGCTGTCGTCAGCTCGTGTTGTGAAAT
GTTGGGTTAAGTCCCGCAACGAGCGCAACCCTTATCCTTTGTTGCCAGCACGTAATGGT
GGGAACTCAAGGGAGACTGCCGGTGACAAACCGGAGGAAGGTGGGGATGACGTCAAG
TCATCATGGCCCTTACGAGTAGGGCTACACACGTGCTACAATGGCAGATACAAAGTGAA
GCGAACTCGCGAGAGTCAGCGGACCACATAAAGTCTGTCGTAGTCCGGATTGGAGTCT
GCAACTCGACTCCATGAAGTCGGAATCGCTAGTAATCGTAGATCAGAATGCTACGGTGA
ATACGTTCCCGGGCCTTGTACACACCGCCCGTCACACCATGGGAGTGGGTTGCAAAAG
AAGTAGGTAGCTTAACCTTCGGGAGGGCGCTTACCACTTTGTGATTCATGACTGGGG

pattern 416

CGCTGGCGGCAGGCCTAACACATGCAAGTCGAGCGGCAGCGGGAAGTAGCTTGCTACT
TTGCCGGCGAGCGGCGGACGGGTGAGTAATGTCTGGGAAACTGCCTGATGGAGGGGGG
TAACTACTGGAAACGGTAGCTAATACCGCATGACGTCTTCGGACCAAAGTGGGGGACCT
TCGGGCCTCACGCCATCGGATGTGCCCAGATGGGATTAGCTAGTAGGTGGGGTAACGGC
TCACCTAGGCGACGATCCCTAGCTGGTCTGAGAGGATGACCAGCCACACTGGAACTGA
GACACGGTCCAGACTCCTACGGGAGGCAGCAGTGGGGAATATTGCACAATGGGCGCAA

GCCTGATGCAGCCATGCCGCGTGTGTGAAGAAGGCCTTCGGGTTGTAAAGCACTTTCAG
CGAGGAGGAAGGCATAAAGGTTAATAACCTTTGTGATTGACGTTACTCGCAGAAGAAG
CACCGGCTAACTCCGTGCCAGCAGCCGCGGTAATACGGAGGGTGCAAGCGTTAATCGG
AATTACTGGGCGTAAAGCGCACGCAGGCGGTTTGTTAAGTCAGATGTGAAATCCCCGCG
CTTAACGTGGGAACTGCATTTGAAACTGGCAAGCTAGAGTCTTGTAGAGGGGGGTAGA
ATTCCAGGTGTAGCGGTGAAATGCGTAGAGATCTGGAGGAATACCGGTGGCGAAGGCG
GCCCCCTGGACAAAGACTGACGCTCAGGTGCGAAAGCGTGGGGAGCAAACAGGATTA
GATACCCTGGTAGTCCACGCTGTAAACGATGTCGACTTGGAGGTTGTGCCCTTGAGGCG
TGGCTTCCGGAGCTAACGCGTTAAGTCGACCGCCTGGGGAGTACGGCCGCAAGGTTAA
AACTCAAATGAATTGACGGGGGCCCGCACAAGCGGTGGAGCATGTGGTTTAATTCGATG
CAACGCGAAGAACCTTACCTACTCTTGACATCCACAGAACTTAGCAGAGATGCTTCGGT
GCCTTCGGGAACTGTGAGACAGGTGCTGCATGGCTGTCGTCAGCTCGTGTTGTGAAATG
TTGGGTTAAGTCCCGCAACGAGCGCAACCCTTATCCTTTGTTGCCAGCGCGTAATGGTG
GGAACTCAAAGGAGACTGCCGGTGATAAACCGGAGGAAGGTGGGGATGACGTCAAGT
CATCATGGCCCTTACGAGTAGGGCTACACACGTGCTACAATGGCAGATACAAAGTGAAG
CAAACTCGCGAGAGCAAGCGGACCACATAAAGTCTGTCGTAGTCCGGATTGGAGTCTG
CAACTCGACTCCATGAAGTCGGAATCGCTAGTAATCGTAGATCAGAATGCTACGGTGAAT
ACGTTCCCGGGCCTTGTACACACCGCCCGTCACACCATGGGAGTGGGTTGCAAAAGAA
GTAGGTAGCTTAACCTTCGGGAGGGCGCTTACCACTTTGTGATTCATGACTGGGG
pattern 417
CGCTGGCGGCAGGCCTAACACATGCAAGTCGAGCGGCAGCGGGGAGTAGCTTGCTACT
TTGCCGGCGAGCGGCGGACGGGTGAGTAATGTCTGGGAAACTGCCTGATGGAGGGGGA
TAACTACTGGAAACGGTAGCTAATACCGCATGACGTCTTCGGACCAAAGTGGGGGACCT
TCGGGCCTCACGCCATCGGATGTGCCCAGATGGGATTAGCTAGTAGGTGGGGTAACGGC
TCACCTAGGCGACGATCCCTAGCTGGTCTGAGAGGATGACCAGCCACACTGGAACTGA
GACACGGTCCAGACTCCTACGGGAGGCAGCAGTGGGGAATATTGCACAATGGGCGCAA
GCCTGATGCAGCCATGCCGCGTGTGTGAAGAAGGCCTTCGGGTTGTAAAGCACTTTCAG
CGAGGAGGAAGGCATAAAGGTTAATAACCTTTGTGATTGACGTTACTCGCAGAAGAAG
CACCGGCTAACTCCGTGCCAGCAGCCGCGGTAATACGGAGGGTGCAAGCGTTAATCGG
AATTACTGGGCGTAAAGCGCACGCAGGCGGTTTGTTAAGTCAGATGTGAAATCCCCGCG
CTTAACGTGGGAACTGCATTTGAAACTGGCAAGCTAGAGTCTTGTAGAGGGGGGTAGA
ATTCCAGGTGTAGCGGTGAAATGCGTAGAGATCTGGAGGAATACCGGTGGCGAAGGCG
GCCCCCTGGACAAAGACTGACGCTCAGGTGCGAAAGCGTGGGGAGCAAACAGGATTA
GATACCCTGGTAGTCCACGCTGTAAACGATGTCGACTTGGAGGTTGTGCCCTTGAGGCG
TGGCTTCCGGAGCTAACGCGTTAAGTCGACCGCCTGGGGAGTACGGCCGCAAGGTTAA
AACTCAAATGAATTGACGGGGGCCCGCACAAGCGGTGGAGCATGTGGTTTAATTCGATG
CAACGCGAAGAACCTTACCTACTCTTGACATCCACAGAACTTAGCAGAGATGCTTCGGT
GCCTTCGGGAACTGTGAGACAGGTGCTGCATGGCTGTCGTCAGCTCGTGTTGTGAAATG
TTGGGTTAAGTCCCGCAACGAGCGCAACCCTTATCCTTTGTTGCCAGCGCGTAATGGTG
GGAACTCAAAGGAGACTGCCGGTGATAAACCGGAGGAAGGTGGGGATGACGTCAAGT
CATCATGGCCCTTACGAGTAGGGCTACACACGTGCTACAATGGCAGATACAAAGTGAAG
CAAACTCGCGAGAGCAAGCGGACCACATAAAGTCTGTCGTAGTCCGGATTGGAGTCTG
CAACTCGACTCCATGAAGTCGGAATCGCTAGTAATCGTAGATCAGAATGCTACGGTGAAT
ACGTTCCCGGGCCTTGTACACACCGCCCGTCACACCATGGGAGTGGGTTGCAAAAGAA

GTAGGTAGCTTAACCTTCGGGAGGGCGCTTACCACTTTGTGATTCATGACTGGGG
pattern 418
CGCTGGCGGCAGGCCTAACACATGCAAGTCGAGCGGCAGCGGGAAGTAGCTTGCTACT
TTGCCGGCGAGCGGCGGACGGGTGAGTAATGTCTGGGAAACTGCCTGATGGAGGGGGA
TAACTACTGGAAACGGTAGCTAATACCGCATGACGTCTTCGGACCAAAGTGGGGGACCT
TCGGGCCTCACGCCATCGGATGTGCCCAGATGGGATTAGCTAGTAGGTGGGGTAATGGC
TCACCTAGGCGACGATCCCTAGCTGGTCTGAGAGGATGACCAGCCACACTGGAACTGA
GACACGGTCCAGACTCCTACGGGAGGCAGCAGTGGGGAATATTGCACAATGGGCGCAA
GCCTGATGCAGCCATGCCGCGTGTGTGAAGAAGGCCTTCGGGTTGTAAAGCACTTTCAG
CGAGGAGGAAGGCATAAAGGTTAATAACCTTTGTGATTGACGTTACTCGCAGAAGAAG
CACCGGCTAACTCCGTGCCAGCAGCCGCGGTAATACGGAGGGTGCAAGCGTTAATCGG
AATTACTGGGCGTAAAGCGCACGCAGGCGGTTTGTTAAGTCAGATGTGAAATCCCCGCG
CTTAACGTGGGAACTGCATTTGAAACTGGCAAGCTAGAGTCTTGTAGAGGGGGGTAGA
ATTCCAGGTGTAGCGGTGAAATGCGTAGAGATCTGGAGGAATACCGGTGGCGAAGGCG
GCCCCCTGGACAAAGACTGACGCTCAGGTGCGAAAGCGTGGGGAGCAAACAGGATTA
GATACCCTGGTAGTCCACGCTGTAAACGATGTCGACTTGGAGGTTGTGCCCTTGAGGCG
TGGCTTCCGGAGCTAACGCGTTAAGTCGACCGCCTGGGGAGTACGGCCGCAAGGTTAA
AACTCAAATGAATTGACGGGGGCCCGCACAAGCGGTGGAGCATGTGGTTTAATTCGATG
CAACGCGAAGAACCTTACCTACTCTTGACATCCACAGAACTTAGCAGAGATGCTTCGGT
GCCTTCGGGAACTGTGAGACAGGTGCTGCATGGCTGTCGTCAGCTCGTGTTGTGAAATG
TTGGGTTAAGTCCCGCAACGAGCGCAACCCTTATCCTTTGTTGCCAGCGCGTAATGGTG
GGAACTCAAAGGAGACTGCCGGTGATAAACCGGAGGAAGGTGGGGATGACGTCAAGT
CATCATGGCCCTTACGAGTAGGGCTACACACGTGCTACAATGGCAGATACAAAGTGAAG
CAAACTCGCGAGAGCAAGCGGACCACATAAAGTCTGTCGTAGTCCGGATTGGAGTCTG
CAACTCGACTCCATGAAGTCGGAATCGCTAGTAATCGTAGATCAGAATGCTACGGTGAAT
ACGTTCCCGGGCCTTGTACACACCGCCCGTCACACCATGGGAGTGGGTTGCAAAAGAA
GTAGGTAGCTTAACCTTCGGGAGGGCGCTTACCACTTTGTGATTCATGACTGGGG
pattern 419
CGCTGGCGGCAGGCCTAACACATGCAAGTCGAGCGGCAGCGGGGAGTAGCTTGCTACT
TTGCCGGCGAGCGGCGGACGGGTGAGTAATGTCTGGGAAACTGCCTGATGGAGGGGGA
TAACTACTGGAAACGGTAGCTAATACCGCATGACGTCTTCGGACCAAAGTGGGGGACCT
TCGGGCCTCACGCCATCGGATGTGCCCAGATGGGATTAGCTAGTAGGTGGGGTAACGGC
TCACCTAGGCGACGATCCCTAGCTGGTCTGAGAGGATGACCAGCCACACTGGAACTGA
GACACGGTCCAGACTCCTACGGGAGGCAGCAGTGGGGAATATTGCACAATGGGCGCAA
GCCTGATGCAGCCATGCCGCGTGTGTGAAGAAGGCCTTCGGGTTGTAAAGCACTTTCAG
CGAGGAGGAAGGCATAAAGGTTAATAACCTTTGTGATTGACGTTACTCGCAGAAGAAG
CACCGGCTAACTCCGTGCCAGCAGCCGCGGTAATACGGAGGGTGCAAGCGTTAATCGG
AATTACTGGGCGTAAAGCGCACGCAGGCGGTTTGTTAAGTCAGATGTGAAATCCCCGCG
CTTAACGTGGGAACTGCATTTGAAACTGGCAAGCTAGAGTCTTGTAGAGGGGGGTAGA
ATTCCAGGTGTAGCGGTGAAATGCGTAGAGATCTGGAGGAATACCGGTGGCGAAGGCG
GCCCCCTGGACAAAGACTGACGCTCAGGTGCGAAAGCGTGGGGAGCAAACAGGATTA
GATACCCTGGTAGTCCACGCTGTAAACGATGTCGACTTGGAGGTTGTGCCCTTGAGGCG
TGGCTTCCGGAGCTAACGCGTTAAGTCGACCGCCTGGGGAGTACGGCCGCAAGGTTAA
AACTCAAATGAATTGACGGGGGCCCGCACAAGCGGTGGAGCATGTGGTTTAATTCGATG

CAACGCGAAGAACCTTACCTACTCTTGACATCCACAGAACTTAGCAGAGATGCTTCGGT
GCCTTCGGGAACTGTGAGGCAGGTGCTGCATGGCTGTCGTCAGCTCGTGTTGTGAAATG
TTGGGTTAAGTCCCGCAACGAGCGCAACCCTTATCCTTTGTTGCCAGCACGTGATGGTG
GGAACTCAAAGGAGACTGCCGGTGATAAACCGGAGGAAGGTGGGGATGACGTCAAGT
CATCATGGCCCTTACGAGTAGGGCTACACACGTGCTACAATGGCAGATACAAAGTGAAG
CAAACTCGCGAGAGCAAGCGGACCACATAAAGTCTGTCGTAGTCCGGATTGGAGTCTG
CAACTCGACTCCATGAAGTCGGAATCGCTAGTAATCGTAGATCAGAATGCTACGGTGAAT
ACGTTCCCGGGCCTTGTACACACCGCCCGTCACACCATGGGAGTGGGTTGCAAAAGAA
GTAGGTAGCTTAACCTTCGGGAGGGCGCTTACCACTTTGTGATTCATGACTGGGG
pattern 420
CGCTGGCGGCAGGCCTAACACATGCAAGTCGAGCGGCAGCGGGAAGTAGCTTGCTACT
TTGCCGGCGAGCGGCGGACGGGTGAGTAATGTCTGGGAAACTGCCTGATGGAGGGGGA
TAACTACTGGAAACGGTAGCTAATACCGCATGACGTCTTCGGACCAAAGTGGGGGACCT
TCGGGCCTCACGCCATCGGATGTGCCCAGATGGGATTAGCTAGTAGGTGGGGTAATGGC
TCACCTAGGCGACGATCCCTAGCTGGTCTGAGAGGATGACCAGCCACACTGGAACTGA
GACACGGTCCAGACTCCTACGGGAGGCAGCAGTGGGGAATATTGCACAATGGGCGCAA
GCCTGATGCAGCCATGCCGCGTGTGTGAAGAAGGCCTTCGGGTTGTAAAGCACTTTCAG
CGAGGAGGAAGGCATAAAGGTTAATAACCTTTGTGATTGACGTTACTCGCAGAAGAAG
CACCGGCTAACTCCGTGCCAGCAGCCGCGGTAATACGGAGGGTGCAAGCGTTAATCGG
AATTACTGGGCGTAAAGCGCACGCAGGCGGTTTGTTAAGTCAGATGTGAAATCCCCGCG
CTTAACGTGGGAACTGCATTTGAAACTGGCAAGCTAGAGTCTTGTAGAGGGGGGTAGA
ATTCCAGGTGTAGCGGTGAAATGCGTAGAGATCTGGAGGAATACCGGTGGCGAAGGCG
GCCCCCTGGACAAAGACTGACGCTCAGGTGCGAAAGCGTGGGGAGCAAACAGGATTA
GATACCCTGGTAGTCCACGCTGTAAACGATGTCGACTTGGAGGTTGTGCCCTTGAGGCG
TGGCTTCCGGAGCTAACGCGTTAAGTCGACCGCCTGGGGAGTACGGCCGCAAGGTTAA
AACTCAAATGAATTGACGGGGGCCCGCACAAGCGGTGGAGCATGTGGTTTAATTCGATG
CAACGCGAAGAACCTTACCTACTCTTGACATCCACAGAACTTAGCAGAGATGCTTCGGT
GCCTTCGGGAACTGTGAGACAGGTGCTGCATGGCTGTCGTCAGCTCGTGTTGTGAAATG
TTGGGTTAAGTCCCGCAACGAGCGCAACCCTTATCCTTTGTTGCCAGCACGTGATGGTG
GGAACTCAAAGGAGACTGCCGGTGATAAACCGGAGGAAGGTGGGGATGACGTCAAGT
CATCATGGCCCTTACGAGTAGGGCTACACACGTGCTACAATGGCAGATACAAAGTGAAG
CAAACTCGCGAGAGCAAGCGGACCACATAAAGTCTGTCGTAGTCCGGATTGGAGTCTG
CAACTCGACTCCATGAAGTCGGAATCGCTAGTAATCGTAGATCAGAATGCTACGGTGAAT
ACGTTCCCGGGCCTTGTACACACCGCCCGTCACACCATGGGAGTGGGTTGCAAAAGAA
GTAGGTAGCTTAACCTTCGGGAGGGCGCTTACCACTTTGTGATTCATGACTGGGG
pattern 421
CGCTGGCGGCAGGCCTAACACATGCAAGTCGAGCGGCAGCGGGGAGTAGCTTGCTACT
TTGCCGGCGAGCGGCGGACGGGTGAGTAATGTCTGGGAAACTGCCTGATGGAGGGGGA
TAACTACTGGAAACGGTAGCTAATACCGCATGACGTCTTCGGACCAAAGTGGGGGACCT
TCGGGCCTCACGCCATCGGATGTGCCCAGATGGGATTAGCTAGTAGGTGGGGTAACGGC
TCACCTAGGCGACGATCCCTAGCTGGTCTGAGAGGATGACCAGCCACACTGGAACTGA
GACACGGTCCAGACTCCTACGGGAGGCAGCAGTGGGGAATATTGCACAATGGGCGCAA
GCCTGATGCAGCCATGCCGCGTGTGTGAAGAAGGCCTTCGGGTTGTAAAGCACTTTCAG
CGAGGAGGAAGGCATAAAGGTTAATAACCTTTGTGATTGACGTTACTCGCAGAAGAAG

CACCGGCTAACTCCGTGCCAGCAGCCGCGGTAATACGGAGGGTGCAAGCGTTAATCGG
AATTACTGGGCGTAAAGCGCACGCAGGCGGTTTGTTAAGTCAGATGTGAAATCCCCGCG
CTTAACGTGGGAACTGCATTTGAAACTGGCAAGCTAGAGTCTTGTAGAGGGGGGTAGA
ATTCCAGGTGTAGCGGTGAAATGCGTAGAGATCTGGAGGAATACCGGTGGCGAAGGCG
GCCCCCTGGACAAAGACTGACGCTCAGGTGCGAAAGCGTGGGGAGCAAACAGGATTA
GATACCCTGGTAGTCCACGCTGTAAACGATGTCGACTTGGAGGTTGTGCCCTTGAGGCG
TGGCTTCCGGAGCTAACGCGTTAAGTCGACCGCCTGGGGAGTACGGCCGCAAGGTTAA
AACTCAAATGAATTGACGGGGGCCCGCACAAGCGGTGGAGCATGTGGTTTAATTCGATG
CAACGCGAAGAACCTTACCTACTCTTGACATCCACAGAACTTAGCAGAGATGCTTCGGT
GCCTTCGGGAACTGTGAGGCAGGTGCTGCATGGCTGTCGTCAGCTCGTGTTGTGAAATG
TTGGGTTAAGTCCCGCAACGAGCGCAACCCTTATCCTTTGTTGCCAGCACGTGATGGTG
GGAACTCAAAGGAGACTGCCGGTGATAAACCGGAGGAAGGTGGGGATGACGTCAAGT
CATCATGGCCCTTACGAGTAGGGCTACACACGTGCTACAATGGCAGATACAAAGTGAAG
CGAACTCGCGAGAGCAAGCGGACCACATAAAGTCTGTCGTAGTCCGGATTGGAGTCTG
CAACTCGACTCCATGAAGTCGGAATCGCTAGTAATCGTAGATCAGAATGCTACGGTGAAT
ACGTTCCCGGGCCTTGTACACACCGCCCGTCACACCATGGGAGTGGGTTGCAAAAGAA
GTAGGTAGCTTAACCTTCGGGAGGGCGCTTACCACTTTGTGATTCATGACTGGGG
pattern 422
CGCTGGCGGCAGGCCTAACACATGCAAGTCGAGCGGCAGCGGAAAGTAGCTTGCTACT
TTGCCGGCGAGCGGCGGACGGGTGAGTAATGTCTGGGAAACTGCCTGATGGAGGGGGA
TAACTACTGGAAACGGTAGCTAATACCGCATGACCTCGCAAGAGCAAAGTGGGGGACC
TTCGGGCCTCACGCCATCGGATGTGCCCAGATGGGATTAGCTAGTAGGTGAGGTAATGG
CTCACCTAGGCGACGATCCCTAGCTGGTCTGAGAGGATGACCAGCCACACTGGAACTG
AGACACGGTCCAGACTCCTACGGGAGGCAGCAGTGGGGAATATTGCACAATGGGCGCA
AGCCTGATGCAGCCATGCCGCGTGTGTGAAGAAGGCCTTCGGGTTGTAAAGCACTTTCA
GCGAGGAGGAAGGCAGTCGTGTTAATAGCACGATTGATTGACGTTACTCGCAGAAGAA
GCACCGGCTAACTCCGTGCCAGCAGCCGCGGTAATACGGAGGGTGCAAGCGTTAATCG
GAATTACTGGGCGTAAAGCGCACGCAGGCGGTTTGTTAAGTCAGATGTGAAATCCCCGC
GCTTAACGTGGGAACTGCATTTGAAACTGGCAAGCTAGAGTCTTGTAGAGGGGGGTAG
AATTCCAGGTGTAGCGGTGAAATGCGTAGAGATCTGGAGGAATACCGGTGGCGAAGGC
GGCCCCCTGGACAAAGACTGACGCTCAGGTGCGAAAGCGTGGGGAGCAAACAGGATT
AGATACCCTGGTAGTCCACGCTGTAAACGATGTCGACTTGGAGGTTGTGCCCTTGAGGT
GTGGCTTCCGGAGCTAACGCGTTAAGTCGACCGCCTGGGGAGTACGGCCGCAAGGTTA
AAACTCAAATGAATTGACGGGGGCCCGCACAAGCGGTGGAGCATGTGGTTTAATTCGAT
GCAACGCGAAGAACCTTACCTACTCTTGACATCCACAGAACTTAGCAGAGATGCTTCGG
TGCCTTCGGGAACTGTGAGACAGGTGCTGCATGGCTGTCGTCAGCTCGTGTTGTGAAAT
GTTGGGTTAAGTCCCGCAACGAGCGCAACCCTTATCCTTTGTTGCCAGCACGTAATGGT
GGGAACTCAAGGGAGACTGCCGGTGACAAACCGGAGGAAGGTGGGGATGACGTCAAG
TCATCATGGCCCTTACGAGTAGGGCTACACACGTGCTACAATGGCAGATACAAAGTGAA
GCGAACTCGCGAGAGCCAGCGGACCACATAAAGTCTGTCGTAGTCCGGATTGGAGTCT
GCAACTCGACTCCATGAAGTCGGAATCGCTAGTAATCGTAGATCAGAATGCTACGGTGA
ATACGTTCCCGGGCCTTGTACACACCGCCCGTCACACCATGGGAGTGGGTTGCAAAAG
AAGTAGGTAGCTTAACCTTCGGGAGGGCGCTTACCACTTTGTGATTCATGACTGGGG
pattern 423

CGCTGGCGGCAGGCCTAACACATGCAAGTCGAGCGGCAGCGGAAAGTAGCTTGCTACT
TTGCCGGCGAGCGGCGGACGGGTGAGTAATGTCTGGGAAACTGCCTGATGGAGGGGGA
TAACTACTGGAAACGGTAGCTAATACCGCATGACCTCGCAAGAGCAAAGTGGGGGACC
TTCGGGCCTCACGCCATCGGATGTGCCCAGATGGGATTAGCTAGTAGGTGAGGTAATGG
CTCACCTAGGCGACGATCCCTAGCTGGTCTGAGAGGATGACCAGCCACACTGGAACTG
AGACACGGTCCAGACTCCTACGGGAGGCAGCAGTGGGGAATATTGCACAATGGGCGCA
AGCCTGATGCAGCCATGCCGCGTGTGTGAAGAAGGCCTTCGGGTTGTAAAGCACTTTCA
GCGAGGAGGAAGGCAGTCGTGTTAATAGCACGATTGATTGACGTTACTCGCAGAAGAA
GCACCGGCTAACTCCGTGCCAGCAGCCGCGGTAATACGGAGGGTGCAAGCGTTAATCG
GAATTACTGGGCGTAAAGCGCACGCAGGCGGTTTGTTAAGTCAGATGTGAAATCCCCGC
GCTTAACGTGGGAACTGCATTTGAAACTGGCAAGCTAGAGTCTTGTAGAGGGGGGTAG
AATTCCAGGTGTAGCGGTGAAATGCGTAGAGATCTGGAGGAATACCGGTGGCGAAGGC
GGCCCCCTGGACAAAGACTGACGCTCAGGTGCGAAAGCGTGGGGAGCAAACAGGATT
AGATACCCTGGTAGTCCACGCTGTAAACGATGTCGACTTGGAGGTTGTGCCCTTGAGGC
GTGGCTTCCGGAGCTAACGCGTTAAGTCGACCGCCTGGGGAGTACGGCCGCAAGGTTA
AAACTCAAATGAATTGACGGGGGCCCGCACAAGCGGTGGAGCATGTGGTTTAATTCGAT
GCAACGCGAAGAACCTTACCTACTCTTGACATCCACAGAACTTAGCAGAGATGCTTCGG
TGCCTTCGGGAACTGTGAGACAGGTGCTGCATGGCTGTCGTCAGCTCGTGTTGTGAAAT
GTTGGGTTAAGTCCCGCAACGAGCGCAACCCTTATCCTTTGTTGCCAGCACGTAATGGT
GGGAACTCAAGGGAGACTGCCGGTGACAAACCGGAGGAAGGTGGGGATGACGTCAAG
TCATCATGGCCCTTACGAGTAGGGCTACACACGTGCTACAATGGCAGATACAAAGTGAA
GCGAACTCGCGAGAGCCAGCGGACCACATAAAGTCTGTCGTAGTCCGGATTGGAGTCT
GCAACTCGACTCCATGAAGTCGGAATCGCTAGTAATCGTAGATCAGAATGCTACGGTGA
ATACGTTCCCGGGCCTTGTACACACCGCCCGTCACACCATGGGAGTGGGTTGCAAAAG
AAGTAGGTAGCTTAACCTTCGGGAGGGCGCTTACCACTTTGTGATTCATGACTGGGG
pattern 424
CGCTGGCGGCAGGCCTAACACATGCAAGTCGAGCGGCAGCGGGAAGTAGTTTACTACT
TTGCCGGCGAGCGGCGGACGGGTGAGTAATGTCTGGGAAACTGCCTGATGGAGGGGGA
TAACTACTGGAAACGGTAGCTAATACCGCATGACCTCGCAAGAGCAAAGTGGGGGACC
TTCGGGCCTCACGCCATCGGATGTGCCCAGATGGGATTAGCTAGTAGGTGGGGTAATGG
CTCACCTAGGCGACGATCCCTAGCTGGTCTGAGAGGATGACCAGCCACACTGGAACTG
AGACACGGTCCAGACTCCTACGGGAGGCAGCAGTGGGGAATATTGCACAATGGGCGCA
AGCCTGATGCAGCCATGCCGCGTGTGTGAAGAAGGCCTTCGGGTTGTAAAGCACTTTCA
GCGAGGAGGAAGGCAGTCGTGTTAATAGCACGATTGATTGACGTTACTCGCAGAAGAA
GCACCGGCTAACTCCGTGCCAGCAGCCGCGGTAATACGGAGGGTGCAAGCGTTAATCG
GAATTACTGGGCGTAAAGCGCACGCAGGCGGTTTGTTAAGTCAGATGTGAAATCCCCGC
GCTTAACGTGGGAACTGCATTTGAAACTGGCAAGCTAGAGTCTTGTAGAGGGGGGTAG
AATTCCAGGTGTAGCGGTGAAATGCGTAGAGATCTGGAGGAATACCGGTGGCGAAGGC
GGCCCCCTGGACAAAGACTGACGCTCAGGCGCGAAAGCGTGGGGAGCAAACAGGATT
AGATACCCTGGTAGTCCACGCTGTAAACGATGTCGACTTGGAGGTTGTGCCCTTGAGGC
GTGGCTTCCGGAGCTAACGCGTTAAGTCGACCGCCTGGGGAGTACGGCCGCAAGGTTA
AAACTCAAATGAATTGACGGGGGCCCGCACAAGCGGTGGAGCATGTGGTTTAATTCGAT
GCAACGCGAAGAACCTTACCTACTCTTGACATCCACAGAACTTAGCAGAGATGCTTCGG
TGCCTTCGGGAACTGTGAGACAGGTGCTGCATGGCTGTCGTCAGCTCGTGTTGTGAAAT

GTTGGGTTAAGTCCCGCAACGAGCGCAACCCTTATCCTTTGTTGCCAGCACGTAATGGT
GGGAACTCAAGGGAGACTGCCGGTGACAAACCGGAGGAAGGTGGGGATGACGTCAAG
TCATCATGGCCCTTACGAGTAGGGCTACACACGTGCTACAATGGCAGATACAAAGTGAA
GCGAACTCGCGAGAGCAAGCGGACCACATAAAGTCTGTCGTAGTCCGGATTGGAGTCT
GCAACTCGACTCCATGAAGTCGGAATCGCTAGTAATCGTAGATCAGAATGCTACGGTGA
ATACGTTCCCGGGCCTTGTACACACCGCCCGTCACACCATGGGAGTGGGTTGCAAAAG
AAGTAGGTAGCTTAACCTTCGGGAGGGCGCTTACCACTTTGTGATTCATGACTGGGG
pattern 425
CGCTGGCGGCAGGCCTAACACATGCAAGTCGAGCGGCAGCGGGAAGTAGTTTACTACT
TTGCCGGCGAGCGGCGGACGGGTGAGTAATGTCTGGGAAACTGCCTGATGGAGGGGGA
TAACTACTGGAAACGGTAGCTAATACCGCATGACCTCGCAAGAGCAAAGTGGGGGACC
TTCGGGCCTCACGCCATCGGATGTGCCCAGATGGGATTAGCTAGTAGGTGGGGTAATGG
CTCACCTAGGCGACGATCCCTAGCTGGTCTGAGAGGATGACCAGCCACACTGGAACTG
AGACACGGTCCAGACTCCTACGGGAGGCAGCAGTGGGGAATATTGCACAATGGGCGCA
AGCCTGATGCAGCCATGCCGCGTGTGTGAAGAAGGCCTTCGGGTTGTAAAGCACTTTCA
GCGAGGAGGAAGGCCAATAGCTTAATACGCTGTTGGATTGACGTTACTCGCAGAAGAA
GCACCGGCTAACTCCGTGCCAGCAGCCGCGGTAATACGGAGGGTGCAAGCGTTAATCG
GAATTACTGGGCGTAAAGCGCACGCAGGCGGTTTGTTAAGTCAGATGTGAAATCCCCGC
GCTTAACGTGGGAACTGCATTTGAAACTGGCAAGCTAGAGTCTTGTAGAGGGGGGTAG
AATTCCAGGTGTAGCGGTGAAATGCGTAGAGATCTGGAGGAATACCGGTGGCGAAGGC
GGCCCCCTGGACAAAGACTGACGCTCAGGTGCGAAAGCGTGGGGAGCAAACAGGATT
AGATACCCTGGTAGTCCACGCTGTAAACGATGTCGACTTGGAGGTTGTGCCCTTGAGGC
GTGGCTTCCGGAGCTAACGCGTTAAGTCGACCGCCTGGGGAGTACGGCCGCAAGGTTA
AAACTCAAATGAATTGACGGGGGCCCGCACAAGCGGTGGAGCATGTGGTTTAATTCGAT
GCAACGCGAAGAACCTTACCTACTCTTGACATCCACAGAACTTAGCAGAGATGCTTCGG
TGCCTTCGGGAACTGTGAGACAGGTGCTGCATGGCTGTCGTCAGCTCGTGTTGTGAAAT
GTTGGGTTAAGTCCCGCAACGAGCGCAACCCTTATCCTTTGTTGCCAGCACGTAATGGT
GGGAACTCAAGGGAGACTGCCGGTGACAAACCGGAGGAAGGTGGGGATGACGTCAAG
TCATCATGGCCCTTACGAGTAGGGCTACACACGTGCTACAATGGCAGATACAAAGTGAA
GCGAACTCGCGAGAGCAAGCGGACCACATAAAGTCTGTCGTAGTCCGGATTGGAGTCT
GCAACTCGACTCCATGAAGTCGGAATCGCTAGTAATCGTAGATCAGAATGCTACGGTGA
ATACGTTCCCGGGCCTTGTACACACCGCCCGTCACACCATGGGAGTGGGTTGCAAAAG
AGTAGGTAGCTTAACCTTCGGGAGGCGCTTACCACTTTGTGATTCATGACTGGGG
pattern 426
CGCTGGCGGCAGGCCTAACACATGCAAGTCGAGCGGCAGCGGGAAGTAGTTTACTACT
TTGCCGGCGAGCGGCGGACGGGTGAGTAATGTCTGGGAAACTGCCTGATGGAGGGGGA
TAACTACTGGAAACGGTAGCTAATACCGCATGACCTCGCAAGAGCAAAGTGGGGGACC
TTCGGGCCTCACGCCATCGGATGTGCCCAGATGGGATTAGCTAGTAGGTGGGGTAATGG
CTCACCTAGGCGACGATCCCTAGCTGGTCTGAGAGGATGACCAGCCACACTGGAACTG
AGACACGGTCCAGACTCCTACGGGAGGCAGCAGTGGGGAATATTGCACAATGGGCGCA
AGCCTGATGCAGCCATGCCGCGTGTGTGAAGAAGGCCTTCGGGTTGTAAAGCACTTTCA
GCGAGGAGGAAGGCCAATAGCTTAATACGCTGTTGGATTGACGTTACTCGCAGAAGAA
GCACCGGCTAACTCCGTGCCAGCAGCCGCGGTAATACGGAGGGTGCAAGCGTTAATCG
GAATTACTGGGCGTAAAGCGCACGCAGGCGGTTTGTTAAGTCAGATGTGAAATCCCCGC

GCTTAACGTGGGAACTGCATTTGAAACTGGCAAGCTAGAGTCTTGTAGAGGGGGTAGA
ATTCCAGGTGTAGCGGTGAAATGCGTAGAGATCTGGAGGAATACCGGTGGCGAAGGCG
GCCCCCTGGACAAAGACTGACGCTCAGGTGCGAAAGCGTGGGGAGCAAACAGGATTA
GATACCCTGGTAGTCCACGCTGTAAACGATGTCGACTTGGAGGTTGTGCCCTTGAGGCG
TGGCTTCCGGAGCTAACGCGTTAAGTCGACCGCCTGGGGAGTACGGCCGCAAGGTTAA
AACTCAAATGAATTGACGGGGGCCCGCACAAGCGGTGGAGCATGTGGTTTAATTCGATG
CAACGCGAAGAACCTTACCTACTCTTGACATCCACAGAACTTAGCAGAGATGCTTCGGT
GCCTTCGGGAACTGTGAGACAGGTGCTGCATGGCTGTCGTCAGCTCGTGTTGTGAAATG
TTGGGTTAAGTCCCGCAACGAGCGCAACCCTTATCCTTTGTTGCCAGCACGTAATGGTG
GGAACTCAAGGGAGACTGCCGGTGACAAACCGGAGGAAGGTGGGGATGACGTCAAGT
CATCATGGCCCTTACGAGTAGGGCTACACACGTGCTACAATGGCAGATACAAAGTGAAG
CGAACTCGCGAGAGCAAGCGGACCACATAAAGTCTGTCGTAGTCCGGATTGGAGTCTG
CAACTCGACTCCATGAAGTCGGAATCGCTAGTAATCGTAGATCAGAATGCTACGGTGAAT
ACGTTCCCGGGCCTTGTACACACCGCCCGTCACACCATGGGAGTGGGTTGCAAAAGAA
GTAGGTAGCTTAACCTTCGGGAGGGCGCTTACCACTTTGTGATTCATGACTGGGG
pattern 427
CGCTGGCGGCAGGCCTAACACATGCAAGTCGAGCGGCAGCGGAAAGTAGCTTGCTACT
TTGCCGGCGAGCGGCGGACGGGTGAGTAATGTCTGGGGATCTGCCTAATGGAGGGGGA
TAACTACTGGAAACGGTAGCTAATACCGCATGACCTCGAAAGAGCAAAGTGGGGGACC
TTCGGGCCTCACGCCATCGGATGAACCCAGATGGGATTAGCTAGTAGGTGGGGTAATGG
CTCACCTAGGCGACGATCCCTAGCTGGTCTGAGAGGATGACCAGCCACACTGGAACTG
AGACACGGTCCAGACTCCTACGGGAGGCAGCAGTGGGGAATATTGCACAATGGGCGCA
AGCCTGATGCAGCCATGCCGCGTGTGTGAAGAAGGCCTTCGGGTTGTAAAGCACTTTCA
GCGAGGAGGAAGGCATTGTGGTTAATAACCGCAGTGATTGACGTTACTCGCAGAAGAA
GCACCGGCTAACTCCGTGCCAGCAGCCGCGGTAATACGGAGGGTGCAAGCGTTAATCG
GAATTACTGGGCGTAAAGCGCACGCAGGCGGTTTGTTAAGTCAGATGTGAAATCCCCGC
GCTTAACGTGGGAACTGCATTTGAAACTGGCAAGCTAGAGTCTTGTAGAGGGGGGTAG
AATTCCAGGTGTAGCGGTGAAATGCGTAGAGATCTGGAGGAATACCGGTGGCGAAGGC
GGCCCCCTGGACAAAGACTGACGCTCAGGTGCGAAAGCGTGGGGAGCAAACAGGATT
AGATACCCTGGTAGTCCACGCTGTAAACGATGTCGACTTGGAGGTTGTGCCCTTGAGGT
GTGGCTTCCGGAGCTAACGCGTTAAGTCGACCGCCTGGGGAGTACGGCCGCAAGGTTA
AAACTCAAATGAATTGACGGGGGCCCGCACAAGCGGTGGAGCATGTGGTTTAATTCGAT
GCAACGCGAAGAACCTTACCTACTCTTGACATCCACAGAACTTAGCAGAGATGCTTCGG
TGCCTTCGGGAACTGTGAGACAGGTGCTGCATGGCTGTCGTCAGCTCGTGTTGTGAAAT
GTTGGGTTAAGTCCCGCAACGAGCGCAACCCTTATCCTTTGTTGCCAGCACGTAATGGT
GGGAACTCAAGGGAGACTGCCGGTGACAAACCGGAGGAAGGTGGGGATGACGTCAAG
TCATCATGGCCCTTACGAGTAGGGCTACACACGTGCTACAATGGCAGATACAAAGTGAA
GCGAACTCGCGAGAGCAAGCGGACCACATAAAGTCTGTCGTAGTCCGGATTGGAGTCT
GCAACTCGACTCCATGAAGTCGGAATCGCTAGTAATCGTAGATCAGAATGCTACGGTGA
ATACGTTCCCGGGCCTTGTACACACCGCCCGTCACACCATGGGAGTGGGTTGCAAAAG
AAGTAGGTAGCTTAACCTTCGGGAGGGCGCTTACCACTTTGTGATTCATGACTGGGG
pattern 428
CGCTGGCGGCAGGCCTAACACATGCAAGTCGAGCGGCAGCGGAAAGTAGCTTGCTACT
TTGCCGGCGAGCGGCGGACGGGTGAGTAATGTCTGGGGATCTGCCTGATGGAGGGGGA

TAACTACTGGAAACGGTAGCTAATACCGCATGACCTCGAAAGAGCAAAGTGGGGGACC
TTCGGGCCTCACGCCATCGGATGAACCCAGATGGGATTAGCTAGTAGGTGGGGTAATGG
CTCACCTAGGCGACGATCCCTAGCTGGTCTGAGAGGATGACCAGCCACACTGGAACTG
AGACACGGTCCAGACTCCTACGGGAGGCAGCAGTGGGGAATATTGCACAATGGGCGCA
AGCCTGATGCAGCCATGCCGCGTGTGTGAAGAAGGCCTTCGGGTTGTAAAGCACTTTCA
GCGAGGAGGAAGGCATTGTGGTTAATAACCGCAGTGATTGACGTTACTCGCAGAAGAA
GCACCGGCTAACTCCGTGCCAGCAGCCGCGGTAATACGGAGGGTGCAAGCGTTAATCG
GAATTACTGGGCGTAAAGCGCACGCAGGCGGTTTGTTAAGTCAGATGTGAAATCCCCGC
GCTTAACGTGGGAACTGCATTTGAAACTGGCAAGCTAGAGTCTTGTAGAGGGGGGTAG
AATTCCAGGTGTAGCGGTGAAATGCGTAGAGATCTGGAGGAATACCGGTGGCGAAGGC
GGCCCCCTGGACAAAGACTGACGCTCAGGTGCGAAAGCGTGGGGAGCAAACAGGATT
AGATACCCTGGTAGTCCACGCTGTAAACGATGTCGACTTGGAGGTTGTGCCCTTGAGGC
GTGGCTTCCGGAGCTAACGCGTTAAGTCGACCGCCTGGGGAGTACGGCCGCAAGGTTA
AAACTCAAATGAATTGACGGGGGCCCGCACAAGCGGTGGAGCATGTGGTTTAATTCGAT
GCAACGCGAAGAACCTTACCTACTCTTGACATCCACGGAATTTAGCAGAAATGCTTTAG
TGCCTTCGGGAACCGTGAGACAGGTGCTGCATGGCTGTCGTCAGCTCGTGTTGTGAAAT
GTTGGGTTAAGTCCCGCAACGAGCGCAACCCTTATCCTTTGTTGCCAGCACGTAATGGT
GGGAACTCAAGGGAGACTGCCGGTGACAAACCGGAGGAAGGTGGGGATGACGTCAAG
TCATCATGGCCCTTACGAGTAGGGCTACACACGTGCTACAATGGCAGATACAAAGTGAA
GCGAACTCGCGAGAGCAAGCGGACCACATAAAGTCTGTCGTAGTCCGGATTGGAGTCT
GCAACTCGACTCCATGAAGTCGGAATCGCTAGTAATCGTAGATCAGAATGCTACGGTGA
ATACGTTCCCGGGCCTTGTACACACCGCCCGTCACACCATGGGAGTGGGTTGCAAAAG
AAGTAGGTAGCTTAACCTTCGGGAGGGCGCTTACCACTTTGTGATTCATGACTGGGG

pattern 429

CGCTGGCGGCAGGCCTAACACATGCAAGTCGAGCGGCAGCGGAAAGTAGCTTGCTACT
TTGCCGGCGAGCGGCGGACGGGTGAGTAATGTCTGGGGATCTGCCTGATGGAGGGGGA
TAACTACTGGAAACGGTAGCTAATACCGCATGACCTCGAAAGAGCAAAGTGGGGGACC
TTCGGGCCTCACGCCATCGGATGAACCCAGATGGGATTAGCTAGTAGGTGGGGTAATGG
CTCACCTAGGCGACGATCCCTAGCTGGTCTGAGAGGATGACCAGCCACACTGGAACTG
AGACACGGTCCAGACTCCTACGGGAGGCAGCAGTGGGGAATATTGCACAATGGGCGCA
AGCCTGATGCAGCCATGCCGCGTGTGTGAAGAAGGCCTTCGGGTTGTAAAGCACTTTCA
GCGAGGAGGAAGGCATTGTGGTTAATAACCGCAGTGATTGACGTTACTCGCAGAAGAA
GCACCGGCTAACTCCGTGCCAGCAGCCGCGGTAATACGGAGGGTGCAAGCGTTAATCG
GAATTACTGGGCGTAAAGCGCACGCAGGCGGTTTGTTAAGTCAGATGTGAAATCCCCGC
GCTTAACGTGGGAACTGCATTTGAAACTGGCAAGCTAGAGTCTTGTAGAGGGGGGTAG
AATTCCAGGTGTAGCGGTGAAATGCGTAGAGATCTGGAGGAATACCGGTGGCGAAGGC
GGCCCCCTGGACAAAGACTGACGCTCAGGTGCGAAAGCGTGGGGAGCAAACAGGATT
AGATACCCTGGTAGTCCACGCTGTAAACGATGTCGACTTGGAGGTTGTGCCCTTGAGGC
GTGGCTTCCGGAGCTAACGCGTTAAGTCGACCGCCTGGGGAGTACGGCCGCAAGGTTA
AAACTCAAATGAATTGACGGGGGCCCGCACAAGCGGTGGAGCATGTGGTTTAATTCGAT
GCAACGCGAAGAACCTTACCTACTCTTGACATCCACGGAATTTAGCAGAGATGCTTTAG
TGCCTTCGGGAACTGTGAGACAGGTGCTGCATGGCTGTCGTCAGCTCGTGTTGTGAAAT
GTTGGGTTAAGTCCCGCAACGAGCGCAACCCTTATCCTTTGTTGCCAGCACGTAATGGT
GGGAACTCAAGGGAGACTGCCGGTGACAAACCGGAGGAAGGTGGGGATGACGTCAAG

TCATCATGGCCCTTACGAGTAGGGCTACACACGTGCTACAATGGCAGATACAAAGTGAA
GCGAACTCGCGAGAGCAAGCGGACCACATAAAGTCTGTCGTAGTCCGGATTGGAGTCT
GCAACTCGACTCCATGAAGTCGGAATCGCTAGTAATCGTAGATCAGAATGCTACGGTGA
ATACGTTCCCGGGCCTTGTACACACCGCCCGTCACACCATGGGAGTGGGTTGCAAAAG
AAGTAGGTAGCTTAACCTTCGGGAGGGCGCTTACCACTTTGTGATTCATGACTGGGG
pattern 430
CGCTGGCGGCAGGCCTAACACATGCAAGTCGAGCGGCAGCGGGAAGTAGTTTACTACT
TTGCCGGCGAGCGGCGGACGGGTGAGTAATGTCTGGGAAACTGCCTGATGGAGGGGGA
TAACTACTGGAAACGGTAGCTAATACCGCATGACCTCGTAAGAGCAAAGTGGGGGACCT
TCGGGCCTCACGCCATCGGATGTGCCCAGATGGGATTAGCTAGTAGGTGGGGTAATGGC
TCACCTAGGCGACGATCCCTAGCTGGTCTGAGAGGATGACCAGCCACACTGGAACTGA
GACACGGTCCAGACTCCTACGGGAGGCAGCAGTGGGGAATATTGCACAATGGGCGCAA
GCCTGATGCAGCCATGCCGCGTGTGTGAAGAAGGCCTTCGGGTTGTAAAGCACTTTCAG
CGAGGAGGAAGGGGTTGAGTTTAATACGCTCAATCATTGACGTTACTCGCAGAAGAAG
CACCGGCTAACTCCGTGCCAGCAGCCGCGGTAATACGGAGGGTGCAAGCGTTAATCGG
AATTACTGGGCGTAAAGCGCACGCAGGCGGTTTGTTAAGTCAGATGTGAAATCCCCGCG
CTTAACGTGGGAACTGCATTTGAAACTGGCAAGCTAGAGTCTTGTAGAGGGGGGTAGA
ATTCCAGGTGTAGCGGTGAAATGCGTAGAGATCTGGAGGAATACCGGTGGCGAAGGCG
GCCCCCTGGACAAAGACTGACGCTCAGGTGCGAAAGCGTGGGGAGCAAACAGGATTA
GATACCCTGGTAGTCCACGCTGTAAACGATGTCGACTTGGAGGTTGTGCCCTTGAGGCG
TGGCTTCCGGAGCTAACGCGTTAAGTCGACCGCCTGGGGAGTACGGCCGCAAGGTTAA
AACTCAAATGAATTGACGGGGGCCCGCACAAGCGGTGGAGCATGTGGTTTAATTCGATG
CAACGCGAAGAACCTTACCTACTCTTGACATCCACAGAACTTAGCAGAGATGCTTCGGT
GCCTTCGGGAACTGTGAGACAGGTGCTGCATGGCTGTCGTCAGCTCGTGTTGTGAAATG
TTGGGTTAAGTCCCGCAACGAGCGCAACCCTTATCCTTTGTTGCCAGCACGTAATGGTG
GGAACTCAAAGGAGACTGCCGGTGACAAACCGGAGGAAGGTGGGGATGACGTCAAGT
CATCATGGCCCTTACGAGTAGGGCTACACACGTGCTACAATGGCAGATACAAAGTGAAG
CGAACTCGCGAGAGCAAGCGGACCACATAAAGTCTGTCGTAGTCCGGATTGGAGTCTG
CAACTCGACTCCATGAAGTCGGAATCGCTAGTAATCGTAGATCAGAATGCTACGGTGAAT
ACGTTCCCGGGCCTTGTACACACCGCCCGTCACACCATGGGAGTGGGTTGCAAAAGAA
GTAGGTAGCTTAACCTTCGGGAGGGCGCTTACCACTTTGTGATTCATGACTGGGG
pattern 431
CGCTGGCGGCAGGCCTAACACATGCAAGTCGAGCGGCAGCGGGGAGTAGTTTACTACT
TTGCCGGCGAGCGGCGGACGGGTGAGTAATGTCTGGGAAACTGCCTGATGGAGGGGGA
TAACTACTGGAAACGGTAGCTAATACCGCATAACGTCTTCGGACCAAAGTGGGGGACCT
TCGGGCCTCACGCCATCGGATGTGCCCAGATGGGATTAGCTAGTAGGTGGGGTAATGGC
TCACCTAGGCGACGATCCCTAGCTGGTCTGAGAGGATGACCAGCCACACTGGAACTGA
GACACGGTCCAGACTCCTACGGGAGGCAGCAGTGGGGAATATTGCACAATGGGCGCAA
GCCTGATGCAGCCATGCCGCGTGTGTGAAGAAGGCCTTCGGGTTGTAAAGCACTTTCAG
CGAGGAGGAAGGCATAAAGGTTAATAACCTTTATGATTGACGTTACTCGCAGAAGAAGC
ACCGGCTAACTCCGTGCCAGCAGCCGCGGTAATACGGAGGGTGCAAGCGTTAATCGGA
ATTACTGGGCGTAAAGCGCACGCAGGCGGTTTGTTAAGTCAGATGTGAAATCCCCGCGC
TTAACGTGGGAACTGCATTTGAAACTGGCAAGCTAGAGTCTTGTAGAGGGGGGTAGAA
TTCCAGGTGTAGCGGTGAAATGCGTAGAGATCTGGAGGAATACCGGTGGCGAAGGCGG

CCCCCTGGACAAAGACTGACGCTCAGGTGCGAAAGCGTGGGGAGCAAACAGGATTAG
ATACCCTGGTAGTCCACGCTGTAAACGATGTCGACTTGGAGGTTGTGCCCTTGAGGCGT
GGCTTCCGGAGCTAACGCGTTAAGTCGACCGCCTGGGGAGTACGGCCGCAAGGTTAAA
ACTCAAATGAATTGACGGGGGCCCGCACAAGCGGTGGAGCATGTGGTTTAATTCGATGC
AACGCGAAGAACCTTACCTACTCTTGACATCCACAGAACTTAGCAGAGATGCTTCGGTG
CCTTCGGGAACTGTGAGACAGGTGCTGCATGGCTGTCGTCAGCTCGTGTTGTGAAATGT
TGGGTTAAGTCCCGCAACGAGCGCAACCCTTATCCTTTGTTGCCAGCACGTCATGGTGG
GAACTCAAAGGAGACTGCCGGTGATAAACCGGAGGAAGGTGGGGATGACGTCAAGTC
ATCATGGCCCTTACGAGTAGGGCTACACACGTGCTACAATGGCAGATACAAAGTGAAGC
GAACTCGCGAGAGCAAGCGGACCACATAAAGTCTGTCGTAGTCCGGATTGGAGTCTGC
AACTCGACTCCATGAAGTCGGAATCGCTAGTAATCGTAGATCAGAATGCTACGGTGAAT
ACGTTCCCGGGCCTTGTACACACCGCCCGTCACACCATGGGAGTGGGTTGCAAAAGAA
GTAGGTAGCTTAACCTTCGGGAGGGCGCTTACCACTTTGTGATTCATGACTGGGG
pattern 432
CGCTGGCGGCAGGCCTAACACATGCAAGTCGAGCGGCAGCGGGAAGTAGTTTACTACT
TTGCCGGCGAGCGGCGGACGGGTGAGTAATGTCTGGGAAACTGCCTGATGGAGGGGGA
TAACTACTGGAAACGGTAGCTAATACCGCATGACCTCGTAAGAGCAAAGTGGGGGACCT
TCGGGCCTCACGCCATCGGATGTGCCCAGATGGGATTAGCTAGTAGGTGGGGTAATGGC
TCACCTAGGCGACGATCCCTAGCTGGTCTGAGAGGATGACCAGCCACACTGGAACTGA
GACACGGTCCAGACTCCTACGGGAGGCAGCAGTGGGGAATATTGCACAATGGGCGCAA
GCCTGATGCAGCCATGCCGCGTGTGTGAAGAAGGCCTTCGGGTTGTAAAGCACTTTCAG
CGAGGAGGAAGGCAGTCGTGTTAATAGCACGGTTGATTGACGTTACTCGCAGAAGAAG
CACCGGCTAACTCCGTGCCAGCAGCCGCGGTAATACGGAGGGTGCAAGCGTTAATCGG
AATTACTGGGCGTAAAGCGCACGCAGGCGGTTTGTTAAGTCAGATGTGAAATCCCCGCG
CTTAACGTGGGAACTGCATTTGAAACTGGCAAGCTAGAGTCTTGTAGAGGGGGGTAGA
ATTCCAGGTGTAGCGGTGAAATGCGTAGAGATCTGGAGGAATACCGGTGGCGAAGGCG
GCCCCCTGGACAAAGACTGACGCTCAGGTGCGAAAGCGTGGGGAGCAAACAGGATTA
GATACCCTGGTAGTCCACGCTGTAAACGATGTCGACTTGGAGGTTGTGCCCTTGAGGCG
TGGCTTCCGGAGCTAACGCGTTAAGTCGACCGCCTGGGGAGTACGGCCGCAAGGTTAA
AACTCAAATGAATTGACGGGGGCCCGCACAAGCGGTGGAGCATGTGGTTTAATTCGATG
CAACGCGAAGAACCTTACCTACTCTTGACATCCACGGAATTTAGCAGAGATGCTTCGGT
GCCTTCGGGAACTGTGAGACAGGTGCTGCATGGCTGTCGTCAGCTCGTGTTGTGAAATG
TTGGGTTAAGTCCCGCAACGAGCGCAACCCTTATCCTTTGTTGCCAGCACGTAATGGTG
GGAACTCAAGGGAGACTGCCGGTGACAAACCGGAGGAAGGTGGGGATGACGTCAAGT
CATCATGGCCCTTACGAGTAGGGCTACACACGTGCTACAATGGCAGATACAAAGTGAAG
CGAACTCGCGAGAGCAAGCGGACCACATAAAGTCTGTCGTAGTCCGGATTGGAGTCTG
CAACTCGACTCCATGAAGTCGGAATCGCTAGTAATCGTAGATCAGAATGCTACGGTGAAT
ACGTTCCCGGGCCTTGTACACACCGCCCGTCACACCATGGGAGTGGGTTGCAAAAGAA
GTAGGTAGCTTAACCTTCGGGAGGGCGCTTACCACTTTGTGATTCATGACTGGGG
pattern 433
CGCTGGCGGCAGGCCTAACACATGCAAGTCGAGCGGCAGCGGAAAGTAGCTTGCTACT
TTGCCGGCGAGCGGCGGACGGGTGAGTAATGTCTGGGAAACTGCCTGATGGAGGGGGA
TAACTACTGGAAACGGTAGCTAATACCGCATGACCTCGAAAGAGCAAAGTGGGGGACC
TTCGGGCCTCACGCCATCGGATGTGCCCAGATGGGATTAGCTAGTAGGTGAGGTAATGG

CTCACCTAGGCGACGATCCCTAGCTGGTCTGAGAGGATGACCAGCCACACTGGAACTG
AGACACGGTCCAGACTCCTACGGGAGGCAGCAGTGGGGAATATTGCACAATGGGCGCA
AGCCTGATGCAGCCATGCCGCGTGTGTGAAGAAGGCCTTCGGGTTGTAAAGCACTTTCA
GCGAGGAGGAAGGCATTTCACTTAATACGTGAAGTGATTGACGTTACTCGCAGAAGAA
GCACCGGCTAACTCCGTGCCAGCAGCCGCGGTAATACGGAGGGTGCAAGCGTTAATCG
GAATTACTGGGCGTAAAGCGCACGCAGGCGGTTTGTTAAGTCAGATGTGAAATCCCCGA
GCTTAACTTGGGAACTGCATTTGAAACTGGCAAGCTAGAGTCTTGTAGAGGGGGGTAG
AATTCCAGGTGTAGCGGTGAAATGCGTAGAGATCTGGAGGAATACCGGTGGCGAAGGC
GGCCCCCTGGACAAAGACTGACGCTCAGGTGCGAAAGCGTGGGGAGCAAACAGGATT
AGATACCCTGGTAGTCCACGCTGTAAACGATGTCGACTTGGAGGTTGTGCCCTTGAGGC
GTGGCTTCCGGAGCTAACGCGTTAAGTCGACCGCCTGGGGAGTACGGCCGCAAGGTTA
AAACTCAAATGAATTGACGGGGGCCCGCACAAGCGGTGGAGCATGTGGTTTAATTCGAT
GCAACGCGAAGAACCTTACCTACTCTTGACATCCACAGAACTTAGCAGAGATGCTTAGG
TGCCTTCGGGAACTGTGAGACAGGTGCTGCATGGCTGTCGTCAGCTCGTGTTGTGAAAT
GTTGGGTTAAGTCCCGCAACGAGCGCAACCCTTATCCTTTGTTGCCAGCACGTAATGGT
GGGAACTCAAAGGAGACTGCCGGTGATAAACTGGAGGAAGGTGGGGATGACGTCAAG
TCATCATGGCCCTTACGAGTAGGGCTACACACGTGCTACAATGGCAGATACAAAGTGAA
GCGAACTCGCGAGAGCAAGCGGACCACATAAAGTCTGTCGTAGTCCGGATTGGAGTCT
GCAACTCGACTCCATGAAGTCGGAATCGCTAGTAATCGTAGATCAGAATGCTACGGTGA
ATACGTTCCCGGGCCTTGTACACACCGCCCGTCACACCATGGGAGTGGGTTGCAAAAG
AAGTAGGTAGCTTAACCTTCGGGAGGGCGCTTACCACTTTGTGATTCATGACTGGGG
pattern 434
CGCTGGCGGCAGGCCTAACACATGCAAGTCGAGCGGCAGCGGAAAGTAGCTTGCTACT
TTGCCGGCGAGCGGCGGACGGGTGAGTAATGTCTGGGAAACTGCCTGATGGAGGGGGA
TAACTACTGGAAACGGTAGCTAATACCGCATGACCTCGAAAGAGCAAAGTGGGGGACC
TTCGGGCCTCACGCCATCGGATGTGCCCAGATGGGATTAGCTAGTAGGTGGGGTAATGG
CTCACCTAGGCGACGATCCCTAGCTGGTCTGAGAGGATGACCAGCCACACTGGAACTG
AGACACGGTCCAGACTCCTACGGGAGGCAGCAGTGGGGAATATTGCACAATGGGCGCA
AGCCTGATGCAGCCATGCCGCGTGTGTGAAGAAGGCCTTCGGGTTGTAAAGCACTTTCA
GCGAGGAGGAAGGCATTTCACTTAATACGTGAAGTGATTGACGTTACTCGCAGAAGAA
GCACCGGCTAACTCCGTGCCAGCAGCCGCGGTAATACGGAGGGTGCAAGCGTTAATCG
GAATTACTGGGCGTAAAGCGCACGCAGGCGGTTTGTTAAGTCAGATGTGAAATCCCCGA
GCTTAACTTGGGAACTGCATTTGAAACTGGCAAGCTAGAGTCTTGTAGAGGGGGGTAG
AATTCCAGGTGTAGCGGTGAAATGCGTAGAGATCTGGAGGAATACCGGTGGCGAAGGC
GGCCCCCTGGACAAAGACTGACGCTCAGGTGCGAAAGCGTGGGGAGCAAACAGGATT
AGATACCCTGGTAGTCCACGCTGTAAACGATGTCGACTTGGAGGTTGTGCCCTTGAGGC
GTGGCTTCCGGAGCTAACGCGTTAAGTCGACCGCCTGGGGAGTACGGCCGCAAGGTTA
AAACTCAAATGAATTGACGGGGGCCCGCACAAGCGGTGGAGCATGTGGTTTAATTCGAT
GCAACGCGAAGAACCTTACCTACTCTTGACATCCACAGAACTTAGCAGAGATGCTTAGG
TGCCTTCAGGAACTGTGAGACAGGTGCTGCATGGCTGTCGTCAGCTCGTGTTGTGAAAT
GTTGGGTTAAGTCCCGCAACGAGCGCAACCCTTATCCTTTGTTGCCAGCACGTAATGGT
GGGAACTCAAAGGAGACTGCCGGTGATAAACCGGAGGAAGGTGGGGATGACGTCAAG
TCATCATGGCCCTTACGAGTAGGGCTACACACGTGCTACAATGGCAGATACAAAGTGAA
GCGAACTCGCGAGAGCAAGCGGACCACATAAAGTCTGTCGTAGTCCGGATTGGAGTCT

GCAACTCGACTCCATGAAGTCGGAATCGCTAGTAATCGTAGATCAGAATGCTACGGTGA
ATACGTTCCCGGGCCTTGTACACACCGCCCGTCACACCATGGGAGTGGGTTGCAAAAG
AAGTAGGTAGCTTAACCTTCGGGAGGGCGCTTACCACTTTGTGATTCATGACTGGGG
pattern 435
CGCTGGCGGCAGGCCTAACACATGCAAGTCGAGCGGCAGCGGAAAGTAGCTTGCTACT
TTGCCGGCGAGCGGCGGACGGGTGAGTAATGTCTGGGAAACTGCCTGATGGAGGGGGA
TAACTACTGGAAACGGTAGCTAATACCGCATGACCTCGCAAGAGCAAAGTGGGGGACC
TTCGGGCCTCACGCCATCGGATGTGCCCAGATGGGATTAGCTAGTAGGTGGGGTAATGG
CTCACCTAGGCGACGATCCCTAGCTGGTCTGAGAGGATGACCAGCCACACTGGAACTG
AGACACGGTCCAGACTCCTACGGGAGGCAGCAGTGGGGAATATTGCACAATGGGCGCA
AGCCTGATGCAGCCATGCCGCGTGTGTGAAGAAGGCCTTCGGGTTGTAAAGCACTTTCA
GCGAGGAGGAAGGGGTTGAGTTTAATACGCTTAATCATTGACGTTACTCGCAGAAGAAG
CACCGGCTAACTCCGTGCCAGCAGCCGCGGTAATACGGAGGGTGCAAGCGTTAATCGG
AATTACTGGGCGTAAAGCGCACGCAGGCGGTTTGTTAAGTCAGATGTGAAATCCCCGCG
CTTAACGTGGGAACTGCATTTGAAACTGGCAAGCTAGAGTCTTGTAGAGGGGGGTAGA
ATTCCAGGTGTAGCGGTGAAATGCGTAGAGATCTGGAGGAATACCGGTGGCGAAGGCG
GCCCCCTGGACAAAGACTGACGCTCAGGTGCGAAAGCGTGGGGAGCAAACAGGATTA
GATACCCTGGTAGTCCACGCTGTAAACGATGTCGACTTGGAGGTTGTGCCCTTGAGGCG
TGGCTTCCGGAGCTAACGCGTTAAGTCGACCGCCTGGGGAGTACGGCCGCAAGGTTAA
AACTCAAATGAATTGACGGGGGCCCGCACAAGCGGTGGAGCATGTGGTTTAATTCGATG
CAACGCGAAGAACCTTACCTACTCTTGACATCCACAGAACTTAGCAGAGATGCTTAGGT
GCCTTCGGGAACTGTGAGACAGGTGCTGCATGGCTGTCGTCAGCTCGTGTTGTGAAATG
TTGGGTTAAGTCCCGCAACGAGCGCAACCCTTATCCTTTGTTGCCAGCACGTCATGGTG
GGAACTCAAAGGAGACTGCCGGTGATAAACCGGAGGAAGGTGGGGATGACGTCAAGT
CATCATGGCCCTTACGAGTAGGGCTACACACGTGCTACAATGGCAGATACAAAGTGAAG
CGAACTCGCGAGAGCAAGCGGACCACATAAAGTCTGTCGTAGTCCGGATTGGAGTCTG
CAACTCGACTCCATGAAGTCGGAATCGCTAGTAATCGTAGATCAGAATGCTACGGTGAAT
ACGTTCCCGGGCCTTGTACACACCGCCCGTCACACCATGGGAGTGGGTTGCAAAAGAA
GTAGGTAGCTTAACCTTCGGGAGGGCGCTTACCACTTTGTGATTCATGACTGGGG
pattern 436
CGCTGGCGGCAGGCCTAACACATGCAAGTCGAGCGGCAGCGGAAAGTAGCTTGCTACT
TTGCCGGCGAGCGGCGGACGGGTGAGTAATGTCTGGGAAACTGCCTGATGGAGGGGGA
TAACTACTGGAAACGGTAGCTAATACCGCATGACCTCGCAAGAGCAAAGTGGGGGACC
TTCGGGCCTCACGCCATCGGATGTGCCCAGATGGGATTAGCTAGTAGGTGGGGTAATGG
CTCACCTAGGCGACGATCCCTAGCTGGTCTGAGAGGATGACCAGCCACACTGGAACTG
AGACACGGTCCAGACTCCTACGGGAGGCAGCAGTGGGGAATATTGCACAATGGGCGCA
AGCCTGATGCAGCCATGCCGCGTGTGTGAAGAAGGCCTTCGGGTTGTAAAGCACTTTCA
GCGAGGAGGAAGGGGTTGAGTTTAATACGCTCAATCATTGACGTTACTCGCAGAAGAA
GCACCGGCTAACTCCGTGCCAGCAGCCGCGGTAATACGGAGGGTGCAAGCGTTAATCG
GAATTACTGGGCGTAAAGCGCACGCAGGCGGTTTGTTAAGTCAGATGTGAAATCCCCGC
GCTTAACGTGGGAACTGCATTTGAAACTGGCAAGCTAGAGTCTTGTAGAGGGGGGTAG
AATTCCAGGTGTAGCGGTGAAATGCGTAGAGATCTGGAGGAATACCGGTGGCGAAGGC
GGCCCCCTGGACAAAGACTGACGCTCAGGTGCGAAAGCGTGGGGAGCAAACAGGATT
AGATACCCTGGTAGTCCACGCTGTAAACGATGTCGACTTGGAGGTTGTGCCCTTGAGGC

GTGGCTTCCGGAGCTAACGCGTTAAGTCGACCGCCTGGGGAGTACGGCCGCAAGGTTA
AAACTCAAATGAATTGACGGGGGCCCGCACAAGCGGTGGAGCATGTGGTTTAATTCGAT
GCAACGCGAAGAACCTTACCTACTCTTGACATCCACGGAATTTAGCAGAGATGCTTTAG
TGCCTTCGGGAACCGTGAGACAGGTGCTGCATGGCTGTCGTCAGCTCGTGTTGTGAAAT
GTTGGGTTAAGTCCCGCAACGAGCGCAACCCTTATCCTTTGTTGCCAGCACGTAATGGT
GGGAACTCAAAGGAGACTGCCGGTGATAAACCGGAGGAAGGTGGGGATGACGTCAAG
TCATCATGGCCCTTACGAGTAGGGCTACACACGTGCTACAATGGCAGATACAAAGTGAA
GCGAACTCGCGAGAGCAAGCGGACCACATAAAGTCTGTCGTAGTCCGGATTGGAGTCT
GCAACTCGACTCCATGAAGTCGGAATCGCTAGTAATCGTAGATCAGAATGCTACGGTGA
ATACGTTCCCGGGCCTTGTACACACCGCCCGTCACACCATGGGAGTGGGTTGCAAAAG
AAGTAGGTAGCTTAACCTTCGGGAGGGCGCTTACCACTTTGTGATTCATGACTGGGG
pattern 437
CGCTGGCGGCAGGCCTAACACATGCAAGTCGAGCGGCAGCGGAAAGTAGCTTGCTACT
TTGCCGGCGAGCGGCGGACGGGTGAGTAATGTCTGGGGATCTGCCTGATGGAGGGGGA
TAACTACTGGAAACGGTAGCTAATACCGCATGACCTCGAAAGAGCAAAGTGGGGGACC
TTCGGGCCTCACGCCATCGGATGAACCCAGATGGGATTAGCTAGTAGGTGGGGTAATGG
CTCACCTAGGCGACGATCCCTAGCTGGTCTGAGAGGATGACCAGCCACACTGGAACTG
AGACACGGTCCAGACTCCTACGGGAGGCAGCAGTGGGGAATATTGCACAATGGGCGCA
AGCCTGATGCAGCCATGCCGCGTGTGTGAAGAAGGCCTTCGGGTTGTAAAGCACTTTCA
GCGAGGAGGAAGGCATTGTGGTTAATAACCGCAGTGATTGACGTTACTCGCAGAAGAA
GCACCGGCTAACTCCGTGCCAGCAGCCGCGGTAATACGGAGGGTGCAAGCGTTAATCG
GAATTACTGGGCGTAAAGCGCACGCAGGCGGTTTGTTAAGTCAGATGTGAAATCCCCGC
GCTTAACGTGGGAACTGCATTTGAAACTGGCAAGCTAGAGTCTTGTAGAGGGGGGTAG
AATTCCAGGTGTAGCGGTGAAATGCGTAGAGATCTGGAGGAATACCGGTGGCGAAGGC
GGCCCCCTGGACAAAGACTGACGCTCAGGTGCGAAAGCGTGGGGAGCAAACAGGATT
AGATACCCTGGTAGTCCACGCTGTAAACGATGTCGACTTGGAGGTTGTGCCCTTGAGGT
GTGGCTTCCGGAGCTAACGCGTTAAGTCGACCGCCTGGGGAGTACGGCCGCAAGGTTA
AAACTCAAATGAATTGACGGGGGCCCGCACAAGCGGTGGAGCATGTGGTTTAATTCGAT
GCAACGCGAAGAACCTTACCTACTCTTGACATCCACAGAACTTAGCAGAGATGCTTCGG
TGCCTTCGGGAACTGTGAGACAGGTGCTGCATGGCTGTCGTCAGCTCGTGTTGTGAAAT
GTTGGGTTAAGTCCCGCAACGAGCGCAACCCTTATCCTTTGTTGCCAGCACGTAATGGT
GGGAACTCAAGAGAGACTGCCGGTGACAAACCGGAGGAAGGTGGGGATGACGTCAAG
TCATCATGGCCCTTACGAGTAGGGCTACACACGTGCTACAATGGCAGATACAAAGTGAA
GCGAACTCGCGAGAGCAAGCGGACCACATAAAGTCTGTCGTAGTCCGGATTGGAGTCT
GCAACTCGACTCCATGAAGTCGGAATCGCTAGTAATCGTAGATCAGAATGCTACGGTGA
ATACGTTCCCGGGCCTTGTACACACCGCCCGTCACACCATGGGAGTGGGTTGCAAAAG
AAGTAGGTAGCTTAACCTTCGGGAGGGCGCTTACCACTTTGTGATTCATGACTGGGG
pattern 438
CGCTGGCGGCAGGCCTAACACATGCAAGTCGAGCGGCAGCGGAAAGTAGCTTGCTACT
TTGCCGGCGAGCGGCGGACGGGTGAGTAATGTCTGGGGATCTGCCTGATGGAGGGGGA
TAACTACTGGAAACGGTAGCTAATACCGCATGACCTCGAAAGAGCAAAGTGGGGGACC
TTCGGGCCTCACGCCATCGGATGAACCCAGATGGGATTAGCTAGTAGGTGGGGTAACGG
CTCACCTAGGCGACGATCCCTAGCTGGTCTGAGAGGATGACCAGCCACACTGGAACTG
AGACACGGTCCAGACTCCTACGGGAGGCAGCAGTGGGGAATATTGCACAATGGGCGCA

AGCCTGATGCAGCCATGCCGCGTGTGTGAAGAAGGCCTTCGGGTTGTAAAGCACTTTCA
GCGAGGAGGAAGGCATTGTGGTTAATAACCACAGTGATTGACGTTACTCGCAGAAGAA
GCACCGGCTAACTCCGTGCCAGCAGCCGCGGTAATACGGAGGGTGCAAGCGTTAATCG
GAATTACTGGGCGTAAAGCGCACGCAGGCGGTTTGTTAAGTCAGATGTGAAATCCCCGC
GCTTAACGTGGGAACTGCATTTGAAACTGGCAAGCTAGAGTCTTGTAGAGGGGGGTAG
AATTCCAGGTGTAGCGGTGAAATGCGTAGAGATCTGGAGGAATACCGGTGGCGAAGGC
GGCCCCCTGGACAAAGACTGACGCTCAGGTGCGAAAGCGTGGGGAGCAAACAGGATT
AGATACCCTGGTAGTCCACGCTGTAAACGATGTCGACTTGGAGGTTGTGCCCTTGAGGC
GTGGCTTCCGGAGCTAACGCGTTAAGTCGACCGCCTGGGGAGTACGGCCGCAAGGTTA
AAACTCAAATGAATTGACGGGGGCCCGCACAAGCGGTGGAGCATGTGGTTTAATTCGAT
GCAACGCGAAGAACCTTACCTACTCTTGACATCCACAGAACTTAGCAGAGATGCTTAGG
TGCCTTCGGGAACTGTGAGACAGGTGCTGCATGGCTGTCGTCAGCTCGTGTTGTGAAAT
GTTGGGTTAAGTCCCGCAACGAGCGCAACCCTTATCCTTTGTTGCCAGCACGTAATGGT
GGGAACTCAAAGGAGACTGCCGGTGATAAACCGGAGGAAGGTGGGGATGACGTCAAG
TCATCATGGCCCTTACGAGTAGGGCTACACACGTGCTACAATGGCAGATACAAAGTGAA
GCGAACTCGCGAGAGCAAGCGGACCACATAAAGTCTGTCGTAGTCCGGATTGGAGTCT
GCAACTCGACTCCATGAAGTCGGAATCGCTAGTAATCGTAGATCAGAATGCTACGGTGA
ATACGTTCCCGGGCCTTGTACACACCGCCCGTCACACCATGGGAGTGGGTTGCAAAAG
AAGTAGGTAGCTTAACCTTCGGGAGGGCGCTTACCACTTTGTGATTCATGACTGGGG
